# TRiP: Tracking Rhythms in Plants, an automated leaf movement analysis program for circadian period estimation

**DOI:** 10.1186/s13007-015-0075-5

**Published:** 2015-05-03

**Authors:** Kathleen Greenham, Ping Lou, Sara E Remsen, Hany Farid, C Robertson McClung

**Affiliations:** Department of Biological Sciences, Dartmouth College, 78 College Street, Hanover, 03755 USA; Department of Computer Science, Dartmouth College, 6211 Sudikoff Lab, Hanover, 03755 USA

**Keywords:** Leaf movement, Circadian period, Motion estimation, Imaging

## Abstract

**Background:**

A well characterized output of the circadian clock in plants is the daily rhythmic movement of leaves. This process has been used extensively in Arabidopsis to estimate circadian period in natural accessions as well as mutants with known defects in circadian clock function. Current methods for estimating circadian period by leaf movement involve manual steps throughout the analysis and are often limited to analyzing one leaf or cotyledon at a time.

**Results:**

In this study, we describe the development of TRiP (Tracking Rhythms in Plants), a new method for estimating circadian period using a motion estimation algorithm that can be applied to whole plant images. To validate this new method, we apply TRiP to a Recombinant Inbred Line (RIL) population in Arabidopsis using our high-throughput imaging platform. We begin imaging at the cotyledon stage and image through the emergence of true leaves. TRiP successfully tracks the movement of cotyledons and leaves without the need to select individual leaves to be analyzed.

**Conclusions:**

TRiP is a program for analyzing leaf movement by motion estimation that enables high-throughput analysis of large populations of plants. TRiP is also able to analyze plant species with diverse leaf morphologies. We have used TRiP to estimate period for 150 Arabidopsis RILs as well as 5 diverse plant species, highlighting the broad applicability of this new method.

**Electronic supplementary material:**

The online version of this article (doi:10.1186/s13007-015-0075-5) contains supplementary material, which is available to authorized users.

## Background

The genomics era is transforming the way we form and test biological questions. With the decreasing cost of Next Generation Sequencing (NGS) technology the use of high-throughput experimentation on large plant populations is possible. This shift towards expanded genetic and phenotypic analysis has led to next generation mapping populations which include Nested Association Mapping (NAM) populations [1] and Multiparent Advanced Generation Inter-Cross (MAGIC) lines [2] for enhanced gene mapping and trait discovery. The availability of genome sequencing and the advancements in de novo genome assembly have stimulated research in important crop plants and the development of better model systems for studying biofuel production, photosynthesis, abiotic stress response and the impacts of climate change on yield [3]. Many of the current techniques used for phenotyping are extremely labor intensive and often not feasible for the study of large populations. New methods for high-throughput phenotyping [4,5] are being developed to catch up with the mass of NGS data that is being generated.

It is well established that an output ofv the circadian clock in plants is the daily rhythmic movements of their leaves [6]. This rhythmic movement can be used to estimate the period of the internal clock. To determine the timing of leaf movement, time-lapse photography is used to image every 10-20 min over a window of 5-10 days under constant light conditions. This generates large image series that are then analyzed for rhythmicity by tracking the position of the cotyledons or leaves in each image. Several methods have been developed to perform this analysis; however, they all require user input at several steps during the analysis [7-9]. For example, one commonly used method relies on MetaMorph®; software in combination with the Biological Rhythms Analysis Software System (BRASS), which analyzes individual cotyledon movement and fits period, phase and amplitude data using a Fast Fourier Transform Nonlinear Least Squares (FFT-NLLS) method [8]. The input data for BRASS is generated in MetaMorph®;, or an equivalent image analysis software, and this step is a major bottleneck to the analysis. In MetaMorph®;, the region tool is used to select the region surrounding individual leaves. This region must be drawn large enough to surround the leaf across the image stack as it grows and moves over the course of the time series. The coordinates of the leaf are then recorded across the stack and exported to Excel for analyses with BRASS. The need to process each plant individually makes the analysis of a large population extremely labor intensive and time consuming. Another drawback to using a single cotyledon is that the movement of the cotyledon is dependent on active growth of the petiole and once growth ceases the movement dampens dramatically causing unreliable period detection [6]. A more automated method was used to analyze leaf movement on *Brassica oleracea* seedlings; however it required glueing polystyrene balls to each cotyledon blade in order to track the movement in MetaMorph®; [10,11]. To overcome these constraints, we have developed a motion estimation algorithm [12] called Tracking Rhythms in Plants (TRiP) that tracks leaf movement of cotyledons and true leaves simultaneously.

## Results and discussion

### Ground truth

**Figure 1:**
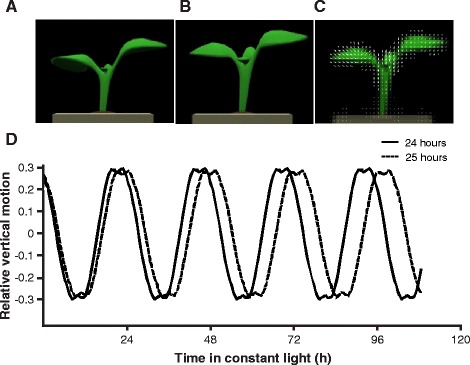
TRiP successfully detects leaf movement from CG plant model with known period. Images of growing Arabidopsis seedlings were digitized and used to animate a time series of leaf movement. **(A)** A trough (T =12, subjective dusk) and **(B)** a peak image (T = 24, subjective dawn) keyframe were each used to produce two animations that demonstrated plant leaf movement with defined circadian periods, which were assessed using TRiP. **(C)** Visualization of motion field at T =24. **(D)** Traces of simulated leaf movement with periods of 24 h or 25 h measured with TRiP.

**Figure 2:**
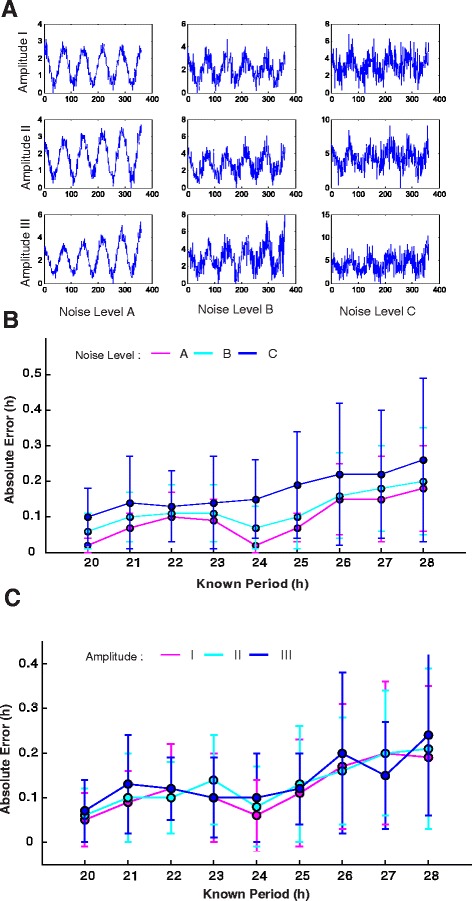
TRiP successfully estimates circadian period from simulated period data.**(A)** Examples of simulations generated for 3 amplitude trends and 3 levels of noise (A = 0.2 amplitude, B = 0.6 amplitude, C = 1 amplitude) **(B)** Error surrounding TRiP period estimates for the 3 levels of noise. **(C)** Error surrounding TRiP period estimates at the 3 amplitude trends with known period. Period and standard deviation data for all simulations can be found in Table 1.

To validate this new method we first simulated time series data with a 3-D computer generated (CG) model of a plant with a 24 h and 25 h period (Figure 1). This 3-D model was animated with a time series based on the manually estimated motion of an Arabidopsis Col-0 seedling. We used TRiP to analyze these simulated video sequences, and obtained 24 h and 25 h periods from the CG model thereby validating the motion detection algorithm. To further test the performance of the circadian period estimation we created simulated traces with periods ranging from 20 h to 28 h. For each period, we introduced 3 different amplitude trends and 3 noise levels that approximate the traces generated from leaf movement data (Figure 2A; Table 1). TRiP accurately estimated the correct period for all simulated traces at all noise levels (Figure 2B). The amplitude trends did not have much of an effect on the model output, which is consistent with previous analysis of FFT-NLLS methods [13]. It should be noted that the motion detection and circadian period estimation are separate steps in the analysis. The motion detection output can be used as input into other circadian period estimation algorithms provided on other platforms such as BioDare [13,14].

**Table 1:** **Period estimation using TRiP to analyze simulated data with different amplitude trends and noise levels**

**Known**	**Amplitude** ^**2**^	**Noise level A** ^**3**^	**Noise level B** ^**3**^	**Noise level C** ^**3**^
**Period** ^**1**^		**Period**	**Period**	**Period**
**(h)**		**(h; mean ± sd)**	**(h; mean ± sd)**	**(h; mean ± sd)**
20	I	20.00 ± 0.02	19.98 ± 0.06	19.99 ± 0.12
21	I	21.04 ± 0.07	21.07 ± 0.09	21.07 ± 0.12
22	I	22.08 ± 0.07	22.11 ± 0.09	22.10 ± 0.18
23	I	23.08 ± 0.07	23.09 ± 0.07	23.07 ± 0.18
24	I	23.99 ± 0.02	24.00 ± 0.04	24.06 ± 0.15
25	I	25.05 ± 0.06	25.05 ± 0.11	25.05 ± 0.26
26	I	26.09 ± 0.14	26.08 ± 0.24	25.98 ± 0.26
27	I	27.11 ± 0.12	27.20 ± 0.12	27.10 ± 0.34
28	I	28.13 ± 0.16	28.12 ± 0.22	28.14 ± 0.27
20	II	20.00 ± 0.03	20.00 ± 0.12	19.97 ± 0.10
21	II	21.06 ± 0.06	21.05 ± 0.09	21.06 ± 0.21
22	II	22.08 ± 0.09	22.10 ± 0.07	22.04 ± 0.14
23	II	23.08 ± 0.07	23.12 ± 0.12	23.05 ± 0.22
24	II	24.00 ± 0.04	23.98 ± 0.09	24.06 ± 0.19
25	II	25.07 ± 0.06	25.06 ± 0.13	25.07 ± 0.28
26	II	26.15 ± 0.11	26.07 ± 0.09	26.08 ± 0.27
27	II	27.16 ± 0.14	27.14 ± 0.14	26.99 ± 0.31
28	II	28.17 ± 0.15	28.19 ± 0.20	28.17 ± 0.31
20	III	20.00 ± 0.03	20.00 ± 0.07	20.06 ± 0.15
21	III	21.07 ± 0.05	21.08 ± 0.14	21.12 ± 0.20
22	III	22.09 ± 0.09	22.12 ± 0.09	22.02 ± 0.16
23	III	23.08 ± 0.08	23.04 ± 0.12	23.04 ± 0.18
24	III	24.00 ± 0.02	23.96 ± 0.12	24.08 ± 0.20
25	III	25.07 ± 0.05	25.08 ± 0.15	24.97 ± 0.19
26	III	26.18 ± 0.11	26.12 ± 0.19	26.22 ± 0.29
27	III	27.15 ± 0.14	27.18 ± 0.14	27.01 ± 0.17
28	III	28.18 ± 0.12	28.17 ± 0.17	28.16 ± 0.38

^1^A cosine of known frequency.

^2^Three levels of amplitude trends, defined as the rate at which the amplitude envelope of the signal decays, were applied: I = 0, II = 0.001, III = 0.002.

^3^Noise levels (A =0.2 amplitude, B =0.6 amplitude, C =1 amplitude).

The mean and standard deviation were calculated from 10 repeated simulations. Circadian periods plotted in Figure 2 were calculated using TRiP.

**Figure 3:**
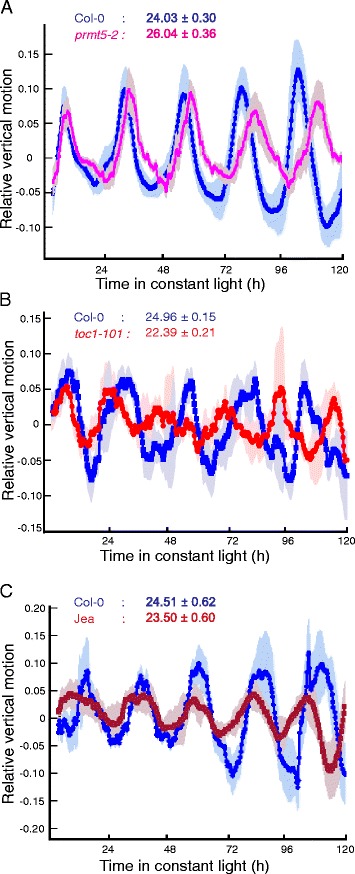
TRiP analysis of agar and soil grown Arabidopsis seedlings.**(A)** TRiP motion traces for the long period mutant *prmt5-2* and Col-0 grown on agar. **(B)** TRiP motion traces for the short period mutant *toc1-101* and Col-0 grown in soil **(C)** TRiP motion traces for Col-0 and the Jea accession grown in soil. Relative vertical motion traces are an average of 10 individual plants for *prmt5-2* and Jea and 5 plants for *toc1-101*. Shading indicates the standard deviation.

We next wanted to test TRiP on live plant images using growth conditions that have previously been used for the leaf movement analysis in Arabidopsis. To test the ability of TRiP to detect period differences on agar grown seedlings, we grew Col-0 and the long period mutant *prmt5-2* [15,16]. Seedlings were imaged for 5 days and analyzed using TRiP; output traces are shown in Figure 3. TRiP calculated a period of 24 h for wild type and 26 h for *prmt5-2*, consistent with published results using the MetaMorph®; and BRASS method described above (Figure 3A). To test the ability of TRiP to analyze leaf movement during emergence of true leaves we grew Col-0, a collection of previously characterized circadian clock mutants and the natural accession Jea on soil and imaged seedlings for 5 days during which time true leaves emerged. Leaf movement was successfully detected and resulted in period estimates consistent with published data (Figure 3B-C, Table 2). These results confirm the functionality of this new method for the analysis of leaf movement in Arabidopsis. An important advantage to the motion estimation algorithm applied in TRiP is that the cotyledon/leaf movement captured in the image is processed to generate one waveform for each plant. This method also alleviates common problems with leaf movement analyses such as overlapping leaves. Even as true leaves emerge and interfere with the cotyledons the movement is still captured.

**Table 2:** **Circadian period of leaf movement on Arabidopsis clock mutants estimated using TRiP**

**Mutant**	**N**	**Period**
		**(h; mean** ***±*** ** sem)**
Col-0	34	24.96 ± 0.15
*cca1-1lhy-20* ^1^	4	19.92 ± 0.20
*toc1-101* ^2^	5	22.39 ± 0.21
*prr5-1* ^3^	4	23.09 ± 0.15
*ztl-4fkf1-2* ^4^	10	31.81 ± 0.87
*prr5-1prr7-3prr9-1* ^5^	8	arrhythmic

^1^Previously described in [17].

^2^Previously described in [18].

^3^Previously described in [19].

^4^Previously described in [20].

^5^Triple mutant generated using alleles described in [19].

Arrhythmicity is consistent with the triple mutant described in [21].

### Applying TRiP to an Arabidopsis RIL population

**Figure 4:**
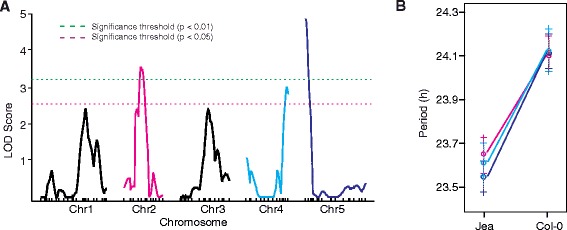
Genetic mapping of circadian period in Col-0 x Jea RIL population.**(A)** QTL likelihood map was generated in R/qtl for each chromosome. Horizontal dashed lines indicate significance levels. **(B)** Effects plot for the QTL above a significance threshold of 0.05. Colors correspond to the QTL in panel **A**.

To apply our leaf movement system to a high-throughput experiment, we analyzed a 150 line RIL population derived from a cross between the Arabidopsis accessions Col-0 and Jea [22]. The ability to image cotyledons and true leaves reduces the complications around germination and growth rate differences within the population. As leaves emerge, TRiP continues to capture the motion in the entire frame. Our current imaging platform allows us to image 1652 plants in one week (Additional file 1). To estimate period in the RIL population we implemented a randomized block design to account for camera and position effects. The resulting mean period values (Additional file 2) were used to map quantitative trait loci (QTL) in this population using available SNP marker data [22]. We identified 3 putative and 2 suggestive QTL for circadian period, with one on each of the 5 chromosomes (Figure 4, Table 3). QTL on the top of chromosome 5 have been identified for circadian period in other studies [19,23]. Candidate clock genes in this region include *PSEUDO-RESPONSE REGULATOR 7* (*PRR7*) [24] and *REVEILLE1* (*RVE1*) [25]. The QTL identified on chromosome 2 includes *EARLY FLOWERING3* (*ELF3*) [26] that has been identified as a QTL for period in a Bay-0 x Shakdara RIL population [27]. Finally, the QTL on chromosome 4 includes *PROTEIN ARGININE METHYLTRANSFERASE 5* (*PRMT5*) [15,16]. We did not detect any significant interactions between the identified QTL. These results demonstrate the utility and sensitivity of TRiP for assessing natural variation in the circadian clock in large plant populations.

**Table 3:** **Summary of circadian period QTL detected in Jea x Col-0 RIL population**

**QTL**	**CHR**	**LOD**	**INT/POS** ^**1**^	**ADD** ^**2**^	**VAR** ^**3**^	**Candidate Genes**
Jea	2	2.33	13.80-33.50 (24)	0.43	7.11	*ELF3, CCR2, XCT, FIO1, LIP1, PHYB, LKP2*
Jea	4	3.01	51.80-60.40 (59)	0.51	5.61	*PRMT5, bHLH69*
Jea	5	4.68	0.00-4.50 (0)	0.56	9.34	*PRR7, RVE1*

^1^INT/POS: 1-LOD QTL interval with peak position, cM.

^2^ADD: Additive effects of the QTL, hours.

^3^VAR: Percent of the variation explained.

### Applying TRiP to diverse plant species

**Figure 5:**
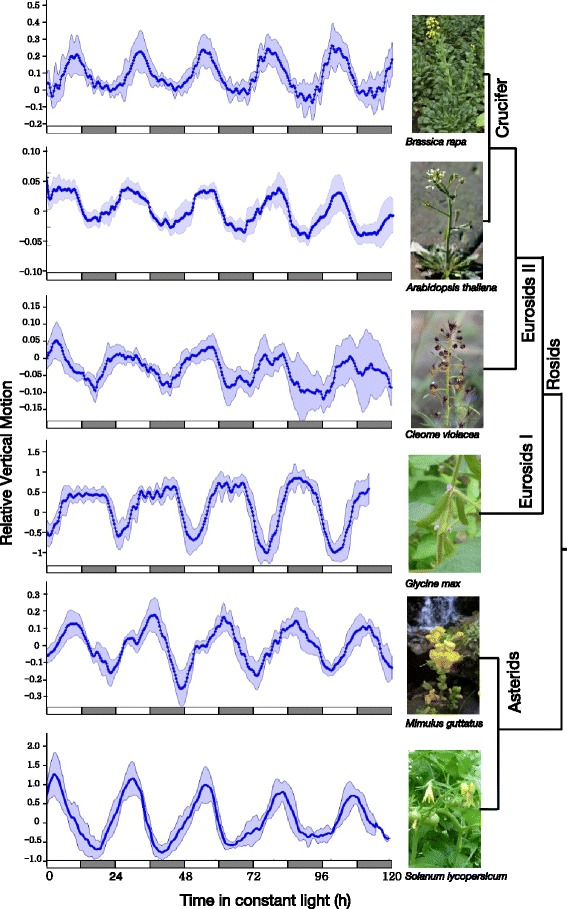
TRiP can be applied to a wide range of plant species with varying leaf morphologies. (From top to bottom: *Brassica rapa*, *Arabidopsis thaliana*, *Cleome violacea*, *Glycine max*, *Mimulus guttatus*, and *Solanum lycopersicum*. Plants were imaged every 20 minutes for 5 days under constant light and temperature at 20 ^∘^C except *Glycine max*, which was imaged at 25 ^∘^C. For each species, the relative vertical motion traces are an average of 8 individual plants (except *Solanum lycopersicum*, where n = 5) analyzed over 5 days. Shading indicates the standard deviation. White and gray bars below each trace indicate subjective day and subjective night, respectively, defined by the entraining photocycle. The phylogenetic relationships among the species are indicated at the right.

An important goal while developing a new method for leaf movement detection was to be able to apply this method to a range of plant species with varying leaf morphology. To test the versatility of TRiP on different plant species we took a phylogenetic approach and selected flowering plant species from diverse clades that included the model species Arabidopsis as well as important crop species including *Brassica rapa* and Soybean. We successfully estimated circadian period from leaf movement data on *Brassica rapa*, *Arabidopsis thaliana*, *Glycine max*, *Cleome violacea*, *Solanum lycopersicum*, and *Mimulus guttatus* (Figure 5; Table 4). Additional video sequences for each species show the leaf movement with and without the TRiP motion vectors (Additional files 3, 4, 5, 6, 7, 8, 9, 10, 11, 12, 13 and 14). This highlights the broad applicability of TRiP to model and non-model species and the feasibility of analyzing large populations of plants in a reasonable amount of time with few hands-on steps during the analysis process.

**Table 4:** **Circadian period of cotyledon or leaf movement across diverse plant species**

**Species**	**N**	**Period**
		**(h; mean** ***±*** ** sd)**
*Brassica rapa*	8	23.58 ± 0.76
*Arabidopsis thaliana*	8	22.51 ± 0.54
*Cleome violacea*	8	25.67 ± 0.94
*Glycine max*	8	24.61 ± 0.25
*Mimulus guttatus*	8	25.02 ± 0.47
*Solanum lycopersicum*	5	25.76 ± 0.64

TRiP was used to estimate circadian period for the six plant species shown in Figure 3.

## Conclusions

The presence of circadian rhythms in plants was first documented in 1729 by the French astronomer Jean Jacques Ortous de Mairan following his observations of the daily leaf movements of the heliotrope plant (*Mimosa*) that persisted in constant darkness [28]. This innate diurnal periodicity was measured a century later by de Candolle and others and found to be approximately 24 h in length [29]. Darwin characterized and quantified these movements extensively in the 1880s [30], paving the way for the emergence of circadian biology. The development of transgenic technologies and the use of fluorescent reporter systems have increased the sensitivity and feasibility of more elaborate circadian clock studies in plants, in particular the model plant Arabidopsis [31,32]. However, with the advances in NGS technology and reduction in cost, the field of molecular ecology is transforming. The ability to sequence natural populations facilitates more directed study for evolutionary and ecological questions such as the genetic basis of local adaptation, speciation, species composition and species interactions [33]. To complement these NGS studies, high-throughput phenotyping methods will need to be developed that can be applied to these natural populations. Understanding the genetic contributions to changes in flowering time in response to photoperiod, temperature and precipitation is critical towards expanding the geographical distribution of crops as well as their adaptability to the changing environment [34,35]. The circadian clock is an important integrator of environmental cues that coordinates the physiological response of the plant through a complex genetic network [36]. The ability to asses circadian clock function and variation in these natural populations will lead to significant advances in our understanding of the interactions between the circadian clock and plant fitness. The automated nature of TRiP, as well as its utility on non-model organisms as demonstrated in this study, makes it an excellent platform for addressing these questions.

## Material and methods

### TRiP program

TRiP is a Matlab-based program. The source code can be run on the open source Octave software with slight modifications outlined in the readme file provided with the TRiP package. The TRiP code has been provided as a supplemental file (Additional file 15) and can also be found on GitHub (http://github.com/KTgreenham/TRiP). The first step of the TRiP analysis is generating individually cropped images of each plant. We have applied a grid-based cropping function that takes each camera image stack as input and crops the images using the grid coordinates given and outputs the cropped image files in a separate directory. We generate the grid coordinates in Matlab of each box drawn around the plant. It is important that the cells are drawn based on the first and last image of the time series to ensure that the entire plant is captured in the crop. Additional notes regarding the grid coordinates can be found in the readme file. Once the grid has been designed, all subsequent experiments can use the same crop function and requires no manual image processing.

#### Motion estimation

Within the Computer Vision and Image Processing communities, differential motion estimation has proven highly effective at computing fine-grained and large-scale motion in video sequences [12,37,38]. We describe one such standard motion estimation algorithm.

To begin, the motion between two sequential frames, *f*(*x*,*y*,*t*) and *f*(*x*,*y*,*t*−1) is modeled with a simple 2-D translation motion vector at each pixel location:

(1) $$\begin{array}{@{}rcl@{}} f(x,y,t) & = & f(x+v_{x}, y+v_{y},t-1), \end{array} $$

where *v*_*x*_ and *v*_*y*_ are the horizontal and vertical motions. That is, the image (or an image patch) is assumed to translate uniformly between times *t* and *t*−1. In order to estimate the motion, we define the following quadratic error function to be minimized:

(2) $$\begin{array}{@{}rcl@{}} {}E(v_{x},v_{y}) & = & \sum\limits_{x,y \in \Omega} [f(x,y,t) - f(x+v_{x}, y+v_{y},t-1)]^{2}, \end{array} $$

where *Ω* denotes a user specified region of interest (ROI) in the image over which the motion is estimated. Minimizing this error function can be difficult and computationally demanding because it is non-linear in the unknown motion parameters. The minimization can be simplified by approximating the error function using a first-order truncated Taylor series expansion:

(3) $$\begin{array}{@{}rcl@{}} E(v_{x},v_{y}) & \approx & \sum\limits_{x,y \in \Omega} [f - (f + v_{x}\,f_{x} + v_{y}f_{y} - f_{t})]^{2} \\ & \approx & \sum\limits_{x,y \in \Omega} [f_{t} - v_{x}\,f_{x} - v_{y\,}f_{y}]^{2} \\ & \approx & \sum\limits_{x,y \in \Omega} \left[f_{t} - \left(f_{x} \quad f_{y}\right) {v_{x}\choose v_{y}} \right]^{2}\\ & \approx & \sum\limits_{x,y \in \Omega} \left[f_{t} - \vec{f_{s}}^{T}\vec{v} \right]^{2}, \end{array} $$

where, *f*_*x*_, *f*_*y*_, and *f*_*t*_ are the spatial and temporal image derivatives and where, for notational convenience, the spatial/temporal parameters on *f* and its derivatives are dropped.

This quadratic error function is now linear in the motion parameters $\vec {v}$ and can therefore be minimized analytically by differentiating with respect to $\vec {v}$:

(4) $$\begin{array}{@{}rcl@{}} \frac{dE}{d\vec{v}} & = & \sum\limits_{x,y \in \Omega} -2\vec{f_{s}} \left[ f_{t} - \vec{f_{s}}^{T}\vec{v} \right], \end{array} $$

seeting the result equal to zero and solving for $\vec {v}$:

(5) $$\begin{array}{@{}rcl@{}} \frac{dE}{d\vec{v}} & = & 0  \\ \sum\limits_{x,y \in \Omega} -2\vec{f_{s}} \left[ f_{t} - \vec{f_{s}}^{T}\vec{v} \right] & = & 0  \\ \sum\limits_{x,y \in \Omega} -2\vec{f_{s}} f_{t} - \sum\limits_{x,y \in \Omega} -2\vec{f_{s}}\vec{f_{s}}^{T}\vec{v} & = & 0 \\ \sum\limits_{x,y \in \Omega} \vec{f_{s}}\vec{f_{s}}^{T}\vec{v} & = & \sum\limits_{x,y \in \Omega} \vec{f_{s}} f_{t}  \\ \vec{v} & = & \left[ \sum\limits_{x,y \in \Omega} \vec{f_{s}}\vec{f_{s}}^{T} \right]^{-1} \sum\limits_{x,y \in \Omega} \vec{f_{s}} f_{t} \\ \vec{v} & = & M^{-1} \vec{b}. \end{array} $$

This solution assumes that the 2×2 matrix *M* is invertible. This can usually be guaranteed by integrating over a large enough ROI *Ω* with sufficient image content.

Given a pair of frames *f*(*x*,*y*,*t*) and *f*(*x*,*y*,*t*−1), the spatial and temporal derivatives are numerically approximated as follows:

(6) $${} \begin{aligned} f_{x}(x,y,t) & = (0.5f(x,y,t) + 0.5f(x,y,t-1)) \star d(x) \star p(y) \\ f_{y}(x,y,t) & = (0.5f(x,y,t) + 0.5f(x,y,t-1)) \star p(x) \star d(y) \\ f_{t}(x,y,t) & = (0.5f(x,y,t) - 0.5f(x,y,t-1)) \star p(x) \star p(y), \end{aligned}  $$

where ⋆ denotes the convolution operator and *d* and *p* are 1-D separable filters:

(7) $$ d(x) = (0.5 -0.5) \quad \text{and} \quad p(x) = (0.5 \quad 0.5),  $$

and where *d*(*y*) and *p*(*y*) are the same filters oriented vertically instead of horizontally.

#### Circadian period estimation

The circadian period is estimated using a two-step process. Denote the plant’s vertical leaf motion over time as *v*_*y*_(*t*). In the first step, this time series is detrended to remove any linear trend. The Fourier transform of *v*_*y*_(*t*) is then computed and the circadian period *τ*_0_ is taken to be the frequency with the maximal amplitude. In the second step, an iterative Nelder-Mead optimization is used to refine this estimate by searching for the frequency, phase and amplitude that best, in the root mean square sense, fits the motion data *v*_*y*_(*t*). This simple approach is similar to employing FFT-NLLS with only a single frequency. We have found that because the motion estimation is fairly accurate, a model based on only a single frequency suffices to extract accurate estimates of circadian period.

### 3-D Computer generated plant model

A 3-D computer generated (CG) model of a plant with a realistic and precisely known motion was used to validate TRiP. Top, front and side views of an Arabidopsis Col-0 seedling were taken every 10 minutes over a 5 day period under constant light conditions and 20 ^∘^C. This time series was used to build and animate a 3-D CG plant model (Figure 1). The modeling, texturing, and animation were done in Autodesk Maya®;. To verify the motion estimation algorithm of TRiP with known motion we used the first day of the 3-D CG model to generate simulated traces with a period of 24 h and 25 h. The resulting rendered video sequence could then be supplied to TRiP for validation of the motion estimation and circadian period estimation. We have also provided the raw images that were selected as key frames across the 5 day imaging along with movie files for the 24 h and 25 h simulations and the full 5 day model (Additional files 16, 17, 18, 19, 20, 21, 22, 23).

### Camera and imaging set up

Our imaging system uses 14 cameras, both Canon PowerShot ELPH 300s and A2300 IS models, employing the CHDK (Canon Hacker Development Kit) software to set interval shooting to take a picture every 20 min. The CHDK software is installed on 4GB SIM cards that have been formatted to FAT32. The Ultimate Intervalometer script is used to run the time interval shooting. Details of the CHDK installation and use can be found on the CHDK wiki. A 4GB memory card can hold images from 3-4 weeks of 20 min interval shooting depending on the camera and image resolution. There are other methods for setting interval shooting on other camera platforms that have been described in previous studies [7,8,39]. Any of these camera systems can be used to generate the images; the new method described in this study was designed for any sequence of jpeg-formatted images. The cameras were mounted with a fixed focus and minimum per plant pixel count of 10,000 (100 × 100 pixels). Plants are placed in front of a black background for contrast. For all plant species tested except *Glycine max*, we built a step shaped structure to maximize the number of plants in one image frame. Each wood frame (L 18 cm × W 12 cm × H 6 cm) supports 6 shelves made of steel hollow sections cut in half lengthwise (L 24 cm × W 1.75 cm × H 0.75 cm). The edges were filed down and covered with electrical tape. Pieces of Plexiglass were glued to the ends using Aquarium safe silicone. The plants are placed in the stands with the tips of the cotyledons or true leaves pointing to either side, the first row holds 18 plants and the remaining rows have 20 for a total of 118 plants/camera (Additional file 1). Larger plants cannot be imaged on every shelf so we limit the imaging to 3 rows of 20 plants for each camera. The plants were watered daily to maintain soil saturation and prevent wilting or movement from soil swelling. To image *Glycine max*, we placed 10 plants in a plexiglass stand (L 45 cm × W 2.75 cm × H 2.5 cm) with 800 mL of water at the start of imaging and watered every day. Imaging for all plants began 24 h following transfer to constant light conditions.

### QTL mapping and analysis

A total of 150 lines in the Col-0 x Jea population were assayed for leaf movement and circadian period estimation using TRiP. Model fit traces that gave period values above 32 h and below 18 h were removed. Standard error of the mean (SEM) was calculated for each line and lines with an SEM above 0.50 (corresponding to 30 min) were removed from the analysis (Additional file 2). Mean period values were used for QTL mapping. The markers and construction of the genetic map were previously described [22]. Composite interval mapping (CIM) was performed with R/qtl [40] using 3 marker covariates and a window size of 20 cM to detect QTL. LOD threshold was calculated based on the averaged LOD following 1000 permutations. A two dimensional genome scan was performed using the “scantwo” function in R/qtl to test for QTL interactions. No significant interactions were detected.

### Plant growth conditions

All plants were grown in Sunshine Redi-earth under ∼90 *μ*mol s ^−^^1^ m ^−^^2^ light unless otherwise stated. All plant species described except *Glycine max* were grown in 0.5" pvc coupling purchased from Home Depot. The pots/pvc couplings were filled with damp soil wet with water. A day after transferring plants to the imaging chamber they were watered once with a 20-20-20 fertilizer. Plants were watered daily to prevent any movement due to water loss or uptake. Soil saturation must be maintained throughout the imaging.

#### Arabidopsis thaliana

Arabidopsis seeds were stratified in H _2_O for 3 days at 4 ^∘^C in the dark. Seeds were germinated in soil and put in a 12 h light : 12 h dark (12L:12D) entrainment chamber at 20 ^∘^C for 7 days. On day 4 of entrainment the lights were turned off 4 h after dawn for 20 h to promote hypocotyl elongation and then returned to 12L:12D for 2 additional days. Following entrainment, seedlings were transferred to 24 h constant light (LL) and temperature (HH) for imaging.

#### Brassica rapa

Dry seeds were sown directly on soil. Plants were entrained for 7 days in a growth chamber at 20 ^∘^C under 12L:12D conditions and high light (∼350 *μ*mol s ^−^^1^ m ^−^^2^) to limit hypocotyl elongation. Once cotyledons had expanded (7 days), plants were transferred to LLHH conditions for imaging.

#### Cleome violacea

Dry seeds were sown directly onto damp soil and entrained to 12L:12D at 20 ^∘^C until true leaves emerged. Plants were imaged in LLHH at 20 ^∘^C for 5 days.

#### Solanum lycopersicum

Dry seeds were sown directly onto damp soil and entrained to 12L:12D at 20 ^∘^C for 7 days under low light. Cotyledons were imaged in LLHH at 20 ^∘^C for 5 days.

#### Glycine max

Dry seeds were sown directly onto damp soil in 2.25" square pots and put in a growth chamber at 12L:12D with a daytime temperature of 25 ^∘^C and night time temperature of 18 ^∘^C. Following emergence of the first trifoliate leaves, plants were transferred to LLHH at 25 ^∘^C for imaging.

#### Mimulus guttatus

Seeds were stratified in the dark at 4 ^∘^C in water for 1 week. Seeds were planted in soil and germinated in the entrainment chamber at 12L:12D with a daytime temperature of 20 ^∘^C and night time temperature of 16 ^∘^C. We observed more robust leaf movement from true leaves. Plants were moved into the imaging room at the emergence of the first set of true leaves and imaged in LLHH at 20 ^∘^C.

## Additional files

Additional file 1
**Figure S1.** Leaf movement camera setup. **(A)** Image from one camera with 118 Arabidopsis seedlings. **(B)** Image of the full camera setup showing the step-shaped platform designed to hold 20 seedlings per row.

Additional file 2
**Table S1.** Data file with the mean period values for each line in the Jea x Col-0 RIL population, the lines highlighted in grey were removed due to SEM values above 0.50.

Additional file 3
**Movie S1.** *Brassica rapa* seedling imaged every 20 min over 5 days.

Additional file 4
**Movie S2.** *Brassica rapa* seedling imaged every 20 min over 5 days. Red arrows represent the TRiP motion vectors.

Additional file 5
**Movie S3.** *Arabidopsis thaliana* seedling imaged every 20 min over 5 days.

Additional file 6
**Movie S4.** *Arabidopsis thaliana* seedling imaged every 20 min over 5 days. Red arrows represent the TRiP motion vectors.

Additional file 7
**Movie S5.** *Cleome violacea* seedling imaged every 20 min over 5 days.

Additional file 8
**Movie S6.** *Cleome violacea* seedling imaged every 20 min over 5 days. Red arrows represent the TRiP motion vectors.

Additional file 9
**Movie S7.** *Glycine max* seedling imaged every 20 min over 5 days.

Additional file 10
**Movie S8.** *Glycine max* seedling imaged every 20 min over 5 days. Red arrows represent the TRiP motion vectors.

Additional file 11
**Movie S9.** *Mimulus guttatus* seedling imaged every 20 min over 5 days.

Additional file 12
**Movie S10.** *Mimulus guttatus* seedling imaged every 20 min over 5 days. Red arrows represent the TRiP motion vectors.

Additional file 13
**Movie S11.** *Solanum lycopersicum* seedling imaged every 20 min over 5 days.

Additional file 14
**Movie S12.** *Solanum lycopersicum* seedling imaged every 20 min over 5 days. Red arrows represent the TRiP motion vectors.

Additional file 15
**TRiP.** Compressed folder containing the TRiP code including a ReadMe file and sample image data.

Additional file 16
**Movie S13.** CG plant model simulating a 24 h period.

Additional file 17
**Movie S14.** CG plant model simulating a 25 h period.

Additional file 18
**Movie S15.** CG plant model over 5 day time course.

Additional file 19
**Col-0 Side View Images for 3-D Model.** Images of Col-0 captured every 10 min for 5 days from the side view for the 3-D CG model. **Table S2** lists the images used as key frames in the model.

Additional file 20
**Col-0 Front View Images for 3-D Model.** Images of Col-0 captured every 10 min for 5 days from the front view for the 3-D CG model. **Table S2** lists the images used as key frames in the model.

Additional file 21
**Col-0 Top View Images for 3-D Model.** First half of images of Col-0 captured every 10 min for 5 days from the top view for the 3-D CG model. **Table S2** lists the images used as key frames in the model.

Additional file 22
**Col-0 Top View Images for 3-D Model.** Second half of images of Col-0 captured every 10 min for 5 days from the top view for the 3-D CG model. **Table S2** lists the images used as key frames in the model.

Additional file 23
**Table S2.** List of the images used as key frames for the 3-D CG model animation.
