# Supplementary figures and images for: TRiP: Tracking Rhythms in Plants, an automated leaf movement analysis program for circadian period estimation (part 1 of 10)
Source: Plant Methods. 2015 May 3;11:33. doi: 10.1186/s13007-015-0075-5 (PMC4445800; doi:10.1186/s13007-015-0075-5)

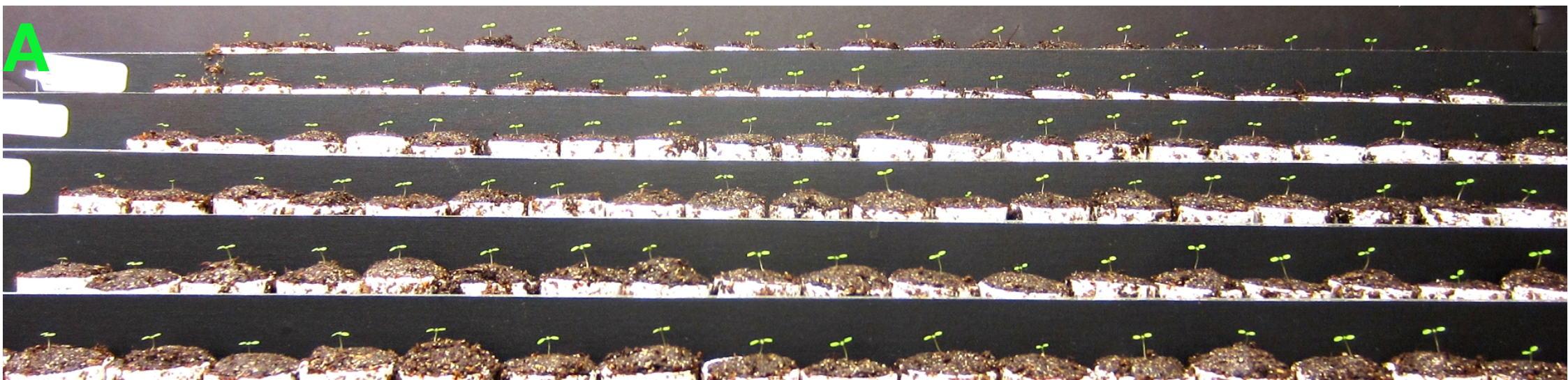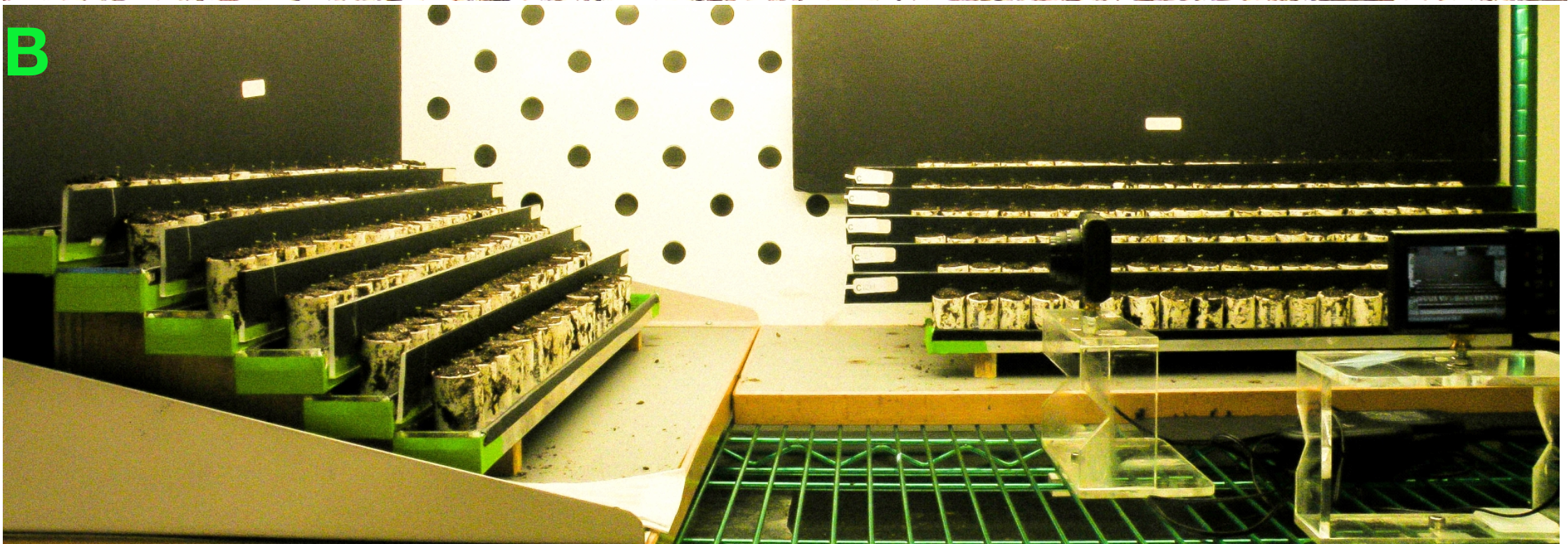

Supplement: Additional file 1 — Figure S1. Leaf movement camera setup. (A) Image from one camera with 118 Arabidopsis seedlings. (B) Image of the full camera setup showing the step-shaped platform designed to hold 20 seedlings per row. [file 13007_2015_75_MOESM1_ESM.pdf]

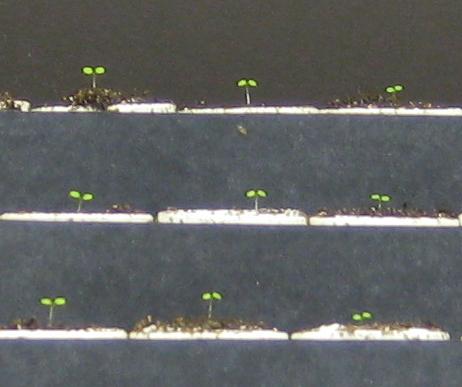

Supplement: Additional file 15 — TRiP. Compressed folder containing the TRiP code including a ReadMe file and sample image data. [file 13007_2015_75_MOESM15_ESM.zip › TRiP/input/C010001.jpg]

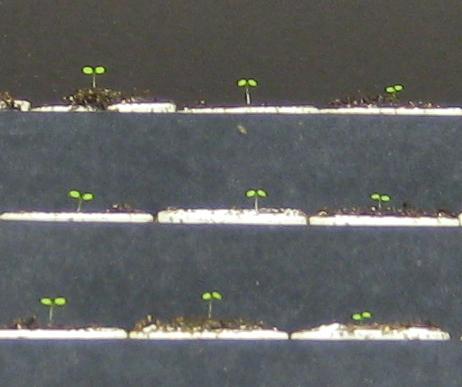

Supplement: Additional file 15 — TRiP. Compressed folder containing the TRiP code including a ReadMe file and sample image data. [file 13007_2015_75_MOESM15_ESM.zip › TRiP/input/C010002.jpg]

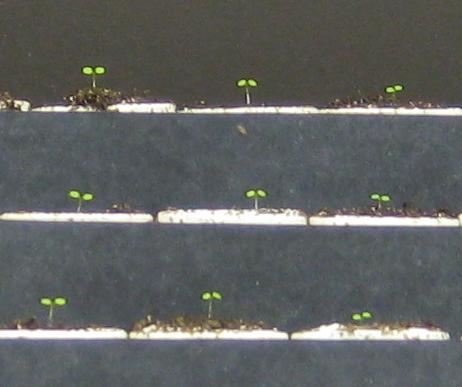

Supplement: Additional file 15 — TRiP. Compressed folder containing the TRiP code including a ReadMe file and sample image data. [file 13007_2015_75_MOESM15_ESM.zip › TRiP/input/C010003.jpg]

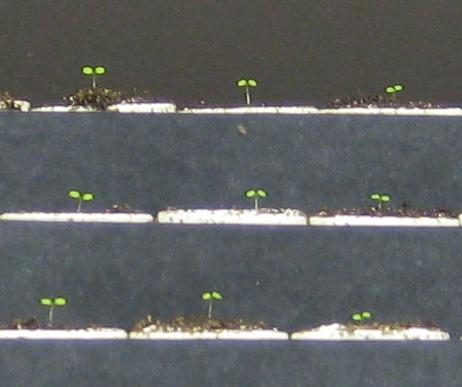

Supplement: Additional file 15 — TRiP. Compressed folder containing the TRiP code including a ReadMe file and sample image data. [file 13007_2015_75_MOESM15_ESM.zip › TRiP/input/C010004.jpg]

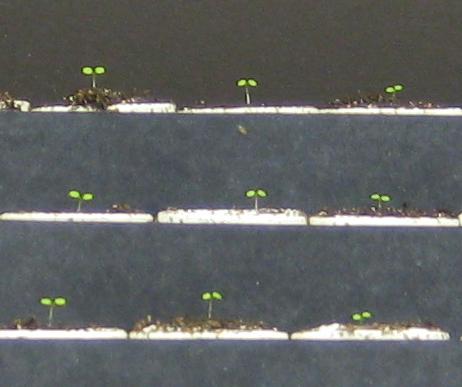

Supplement: Additional file 15 — TRiP. Compressed folder containing the TRiP code including a ReadMe file and sample image data. [file 13007_2015_75_MOESM15_ESM.zip › TRiP/input/C010005.jpg]

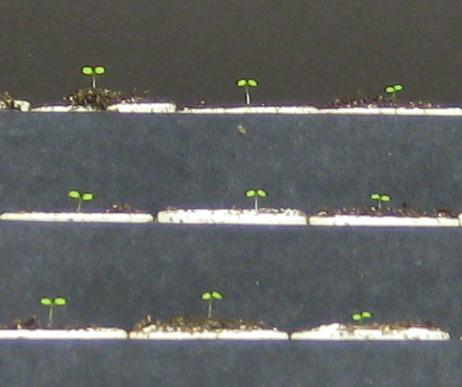

Supplement: Additional file 15 — TRiP. Compressed folder containing the TRiP code including a ReadMe file and sample image data. [file 13007_2015_75_MOESM15_ESM.zip › TRiP/input/C010006.jpg]

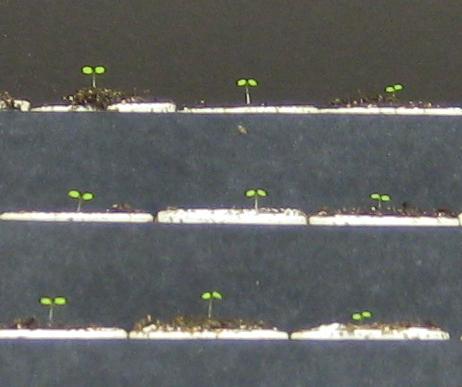

Supplement: Additional file 15 — TRiP. Compressed folder containing the TRiP code including a ReadMe file and sample image data. [file 13007_2015_75_MOESM15_ESM.zip › TRiP/input/C010007.jpg]

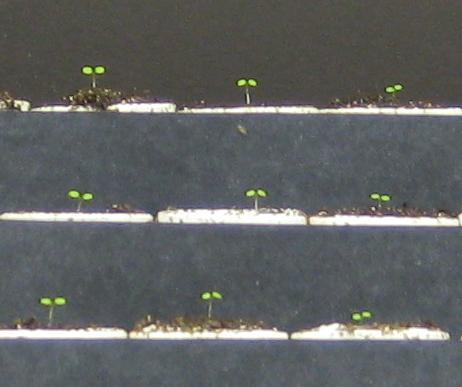

Supplement: Additional file 15 — TRiP. Compressed folder containing the TRiP code including a ReadMe file and sample image data. [file 13007_2015_75_MOESM15_ESM.zip › TRiP/input/C010008.jpg]

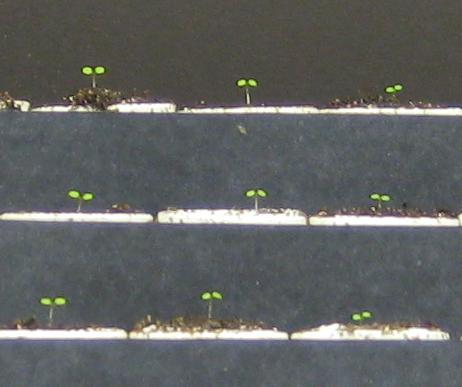

Supplement: Additional file 15 — TRiP. Compressed folder containing the TRiP code including a ReadMe file and sample image data. [file 13007_2015_75_MOESM15_ESM.zip › TRiP/input/C010009.jpg]

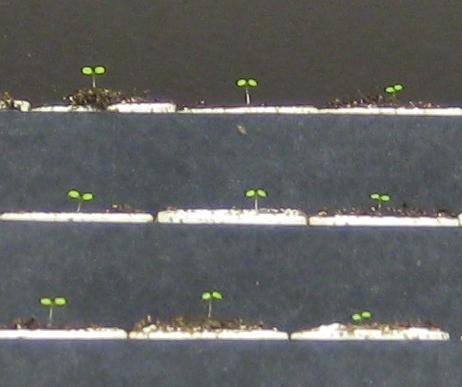

Supplement: Additional file 15 — TRiP. Compressed folder containing the TRiP code including a ReadMe file and sample image data. [file 13007_2015_75_MOESM15_ESM.zip › TRiP/input/C010010.jpg]

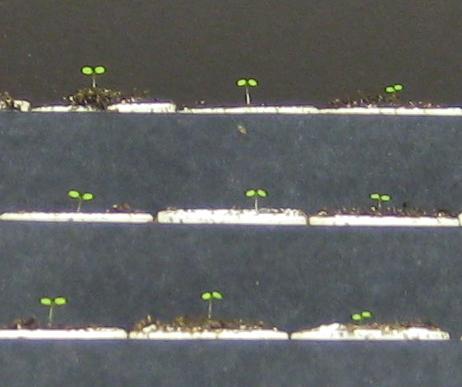

Supplement: Additional file 15 — TRiP. Compressed folder containing the TRiP code including a ReadMe file and sample image data. [file 13007_2015_75_MOESM15_ESM.zip › TRiP/input/C010011.jpg]

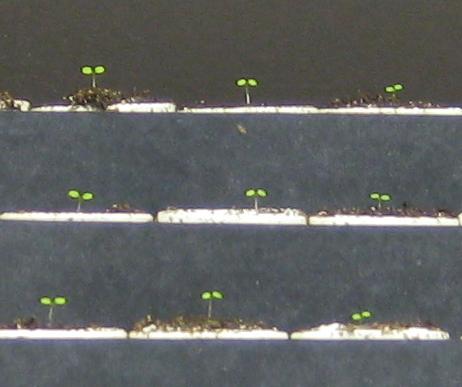

Supplement: Additional file 15 — TRiP. Compressed folder containing the TRiP code including a ReadMe file and sample image data. [file 13007_2015_75_MOESM15_ESM.zip › TRiP/input/C010012.jpg]

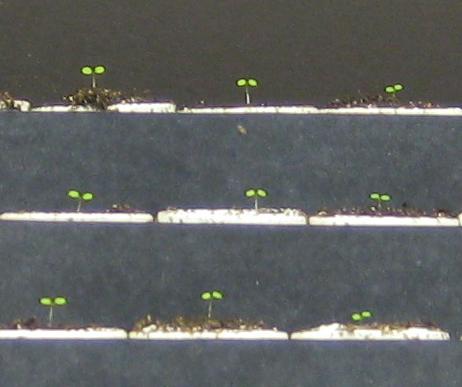

Supplement: Additional file 15 — TRiP. Compressed folder containing the TRiP code including a ReadMe file and sample image data. [file 13007_2015_75_MOESM15_ESM.zip › TRiP/input/C010013.jpg]

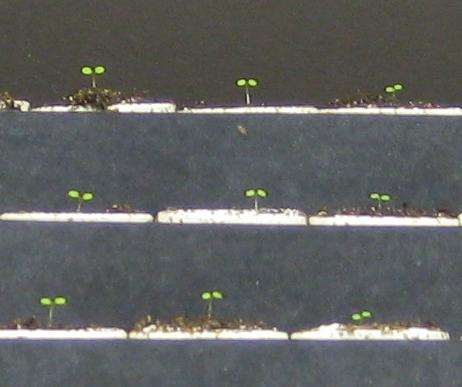

Supplement: Additional file 15 — TRiP. Compressed folder containing the TRiP code including a ReadMe file and sample image data. [file 13007_2015_75_MOESM15_ESM.zip › TRiP/input/C010014.jpg]

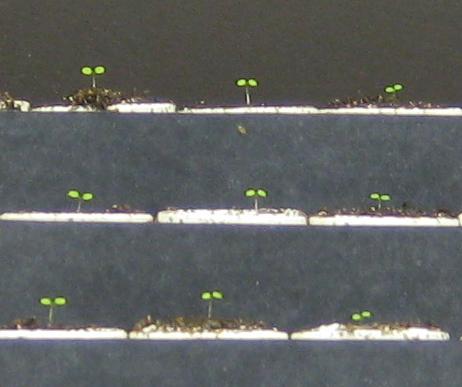

Supplement: Additional file 15 — TRiP. Compressed folder containing the TRiP code including a ReadMe file and sample image data. [file 13007_2015_75_MOESM15_ESM.zip › TRiP/input/C010015.jpg]

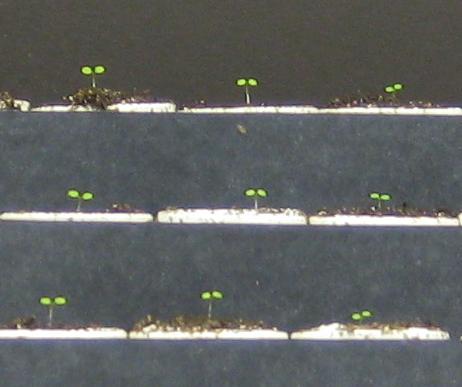

Supplement: Additional file 15 — TRiP. Compressed folder containing the TRiP code including a ReadMe file and sample image data. [file 13007_2015_75_MOESM15_ESM.zip › TRiP/input/C010016.jpg]

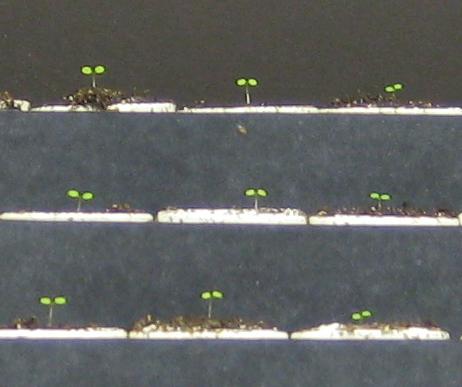

Supplement: Additional file 15 — TRiP. Compressed folder containing the TRiP code including a ReadMe file and sample image data. [file 13007_2015_75_MOESM15_ESM.zip › TRiP/input/C010017.jpg]

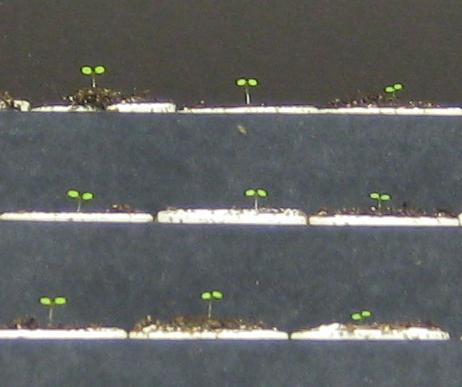

Supplement: Additional file 15 — TRiP. Compressed folder containing the TRiP code including a ReadMe file and sample image data. [file 13007_2015_75_MOESM15_ESM.zip › TRiP/input/C010018.jpg]

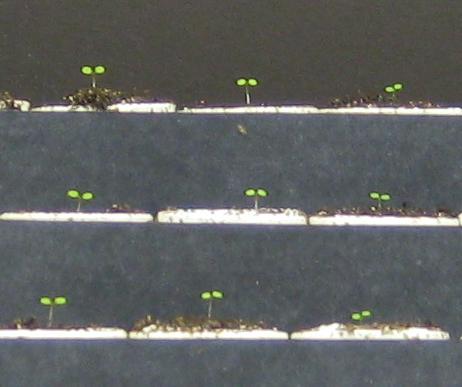

Supplement: Additional file 15 — TRiP. Compressed folder containing the TRiP code including a ReadMe file and sample image data. [file 13007_2015_75_MOESM15_ESM.zip › TRiP/input/C010019.jpg]

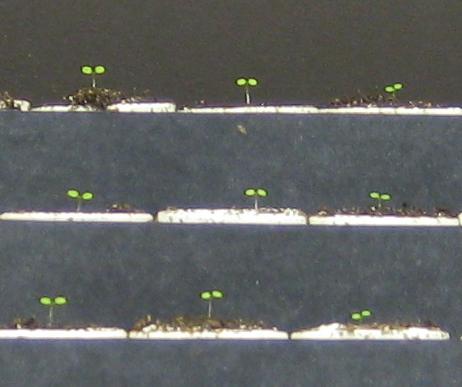

Supplement: Additional file 15 — TRiP. Compressed folder containing the TRiP code including a ReadMe file and sample image data. [file 13007_2015_75_MOESM15_ESM.zip › TRiP/input/C010020.jpg]

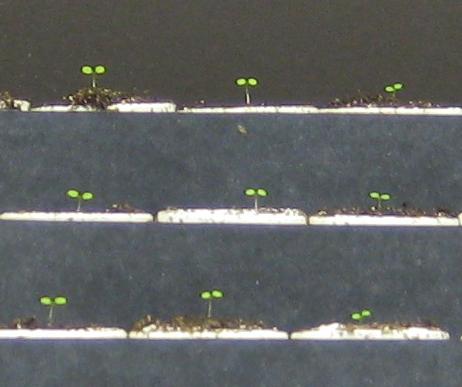

Supplement: Additional file 15 — TRiP. Compressed folder containing the TRiP code including a ReadMe file and sample image data. [file 13007_2015_75_MOESM15_ESM.zip › TRiP/input/C010021.jpg]

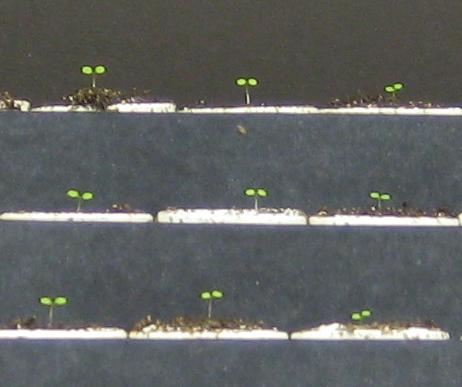

Supplement: Additional file 15 — TRiP. Compressed folder containing the TRiP code including a ReadMe file and sample image data. [file 13007_2015_75_MOESM15_ESM.zip › TRiP/input/C010022.jpg]

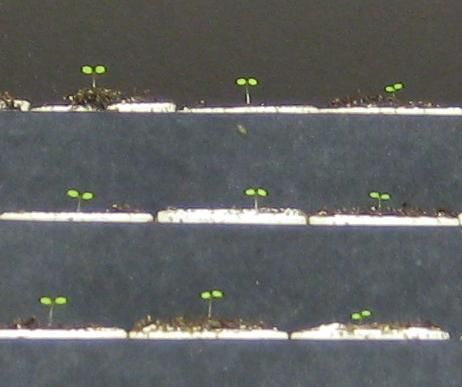

Supplement: Additional file 15 — TRiP. Compressed folder containing the TRiP code including a ReadMe file and sample image data. [file 13007_2015_75_MOESM15_ESM.zip › TRiP/input/C010023.jpg]

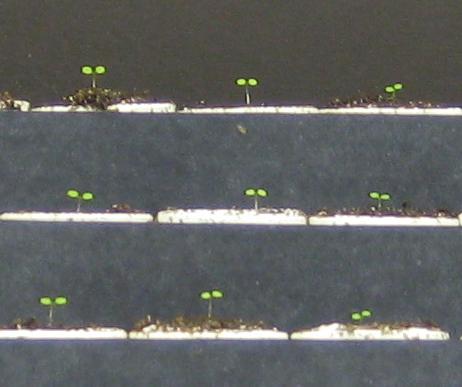

Supplement: Additional file 15 — TRiP. Compressed folder containing the TRiP code including a ReadMe file and sample image data. [file 13007_2015_75_MOESM15_ESM.zip › TRiP/input/C010024.jpg]

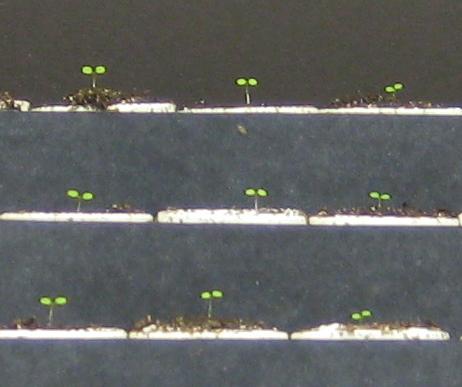

Supplement: Additional file 15 — TRiP. Compressed folder containing the TRiP code including a ReadMe file and sample image data. [file 13007_2015_75_MOESM15_ESM.zip › TRiP/input/C010025.jpg]

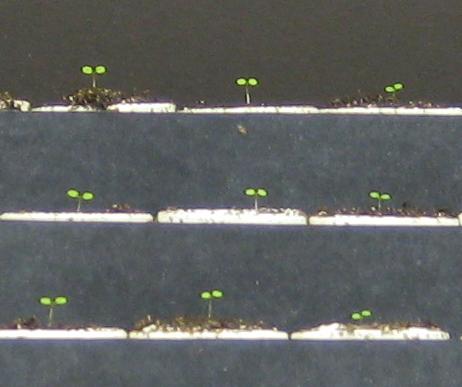

Supplement: Additional file 15 — TRiP. Compressed folder containing the TRiP code including a ReadMe file and sample image data. [file 13007_2015_75_MOESM15_ESM.zip › TRiP/input/C010026.jpg]

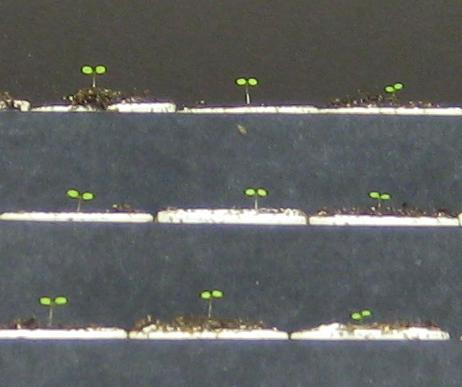

Supplement: Additional file 15 — TRiP. Compressed folder containing the TRiP code including a ReadMe file and sample image data. [file 13007_2015_75_MOESM15_ESM.zip › TRiP/input/C010027.jpg]

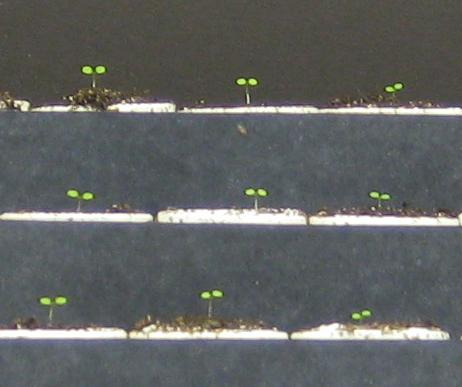

Supplement: Additional file 15 — TRiP. Compressed folder containing the TRiP code including a ReadMe file and sample image data. [file 13007_2015_75_MOESM15_ESM.zip › TRiP/input/C010028.jpg]

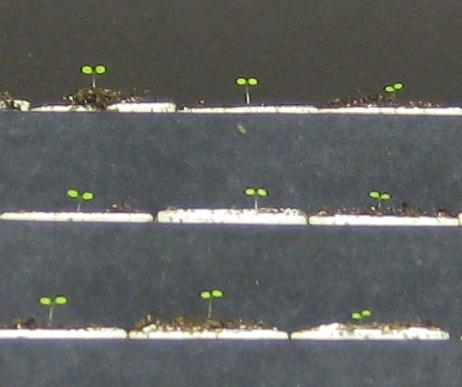

Supplement: Additional file 15 — TRiP. Compressed folder containing the TRiP code including a ReadMe file and sample image data. [file 13007_2015_75_MOESM15_ESM.zip › TRiP/input/C010029.jpg]

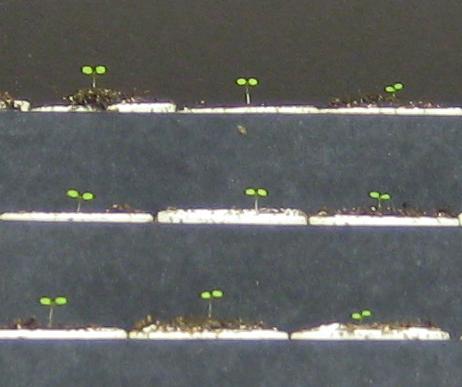

Supplement: Additional file 15 — TRiP. Compressed folder containing the TRiP code including a ReadMe file and sample image data. [file 13007_2015_75_MOESM15_ESM.zip › TRiP/input/C010030.jpg]

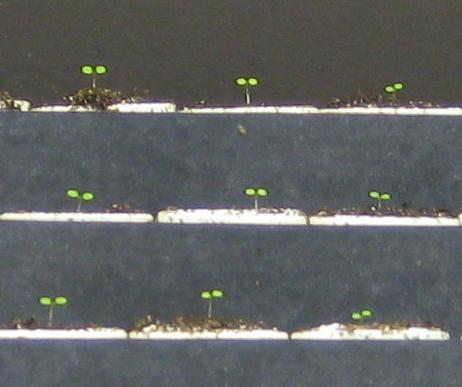

Supplement: Additional file 15 — TRiP. Compressed folder containing the TRiP code including a ReadMe file and sample image data. [file 13007_2015_75_MOESM15_ESM.zip › TRiP/input/C010031.jpg]

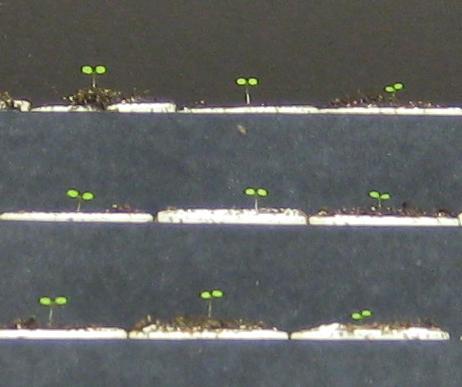

Supplement: Additional file 15 — TRiP. Compressed folder containing the TRiP code including a ReadMe file and sample image data. [file 13007_2015_75_MOESM15_ESM.zip › TRiP/input/C010032.jpg]

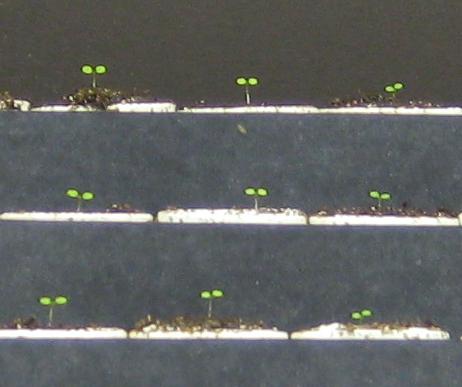

Supplement: Additional file 15 — TRiP. Compressed folder containing the TRiP code including a ReadMe file and sample image data. [file 13007_2015_75_MOESM15_ESM.zip › TRiP/input/C010033.jpg]

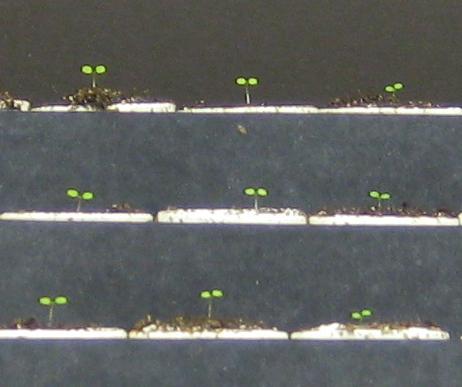

Supplement: Additional file 15 — TRiP. Compressed folder containing the TRiP code including a ReadMe file and sample image data. [file 13007_2015_75_MOESM15_ESM.zip › TRiP/input/C010034.jpg]

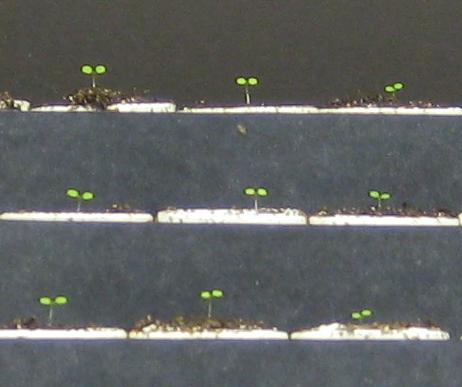

Supplement: Additional file 15 — TRiP. Compressed folder containing the TRiP code including a ReadMe file and sample image data. [file 13007_2015_75_MOESM15_ESM.zip › TRiP/input/C010035.jpg]

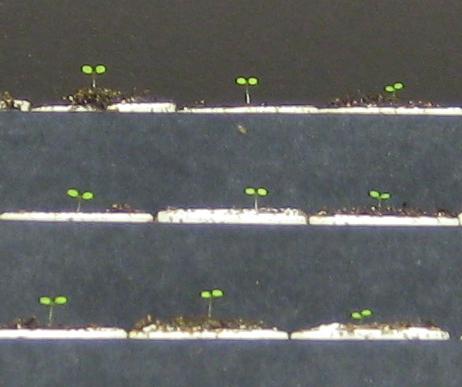

Supplement: Additional file 15 — TRiP. Compressed folder containing the TRiP code including a ReadMe file and sample image data. [file 13007_2015_75_MOESM15_ESM.zip › TRiP/input/C010036.jpg]

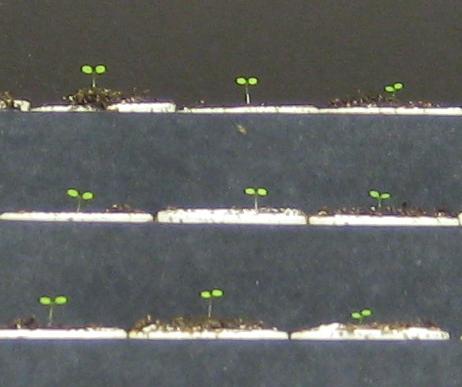

Supplement: Additional file 15 — TRiP. Compressed folder containing the TRiP code including a ReadMe file and sample image data. [file 13007_2015_75_MOESM15_ESM.zip › TRiP/input/C010037.jpg]

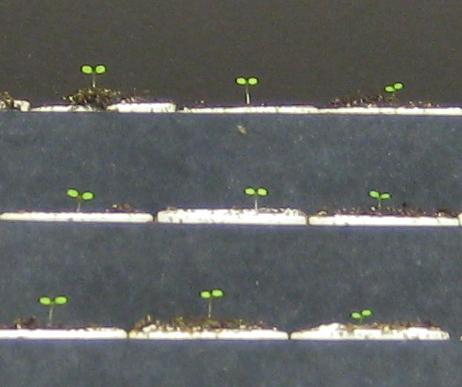

Supplement: Additional file 15 — TRiP. Compressed folder containing the TRiP code including a ReadMe file and sample image data. [file 13007_2015_75_MOESM15_ESM.zip › TRiP/input/C010038.jpg]

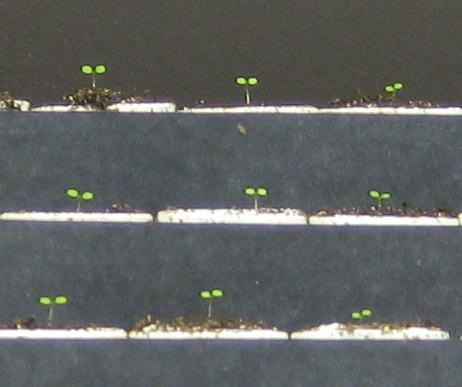

Supplement: Additional file 15 — TRiP. Compressed folder containing the TRiP code including a ReadMe file and sample image data. [file 13007_2015_75_MOESM15_ESM.zip › TRiP/input/C010039.jpg]

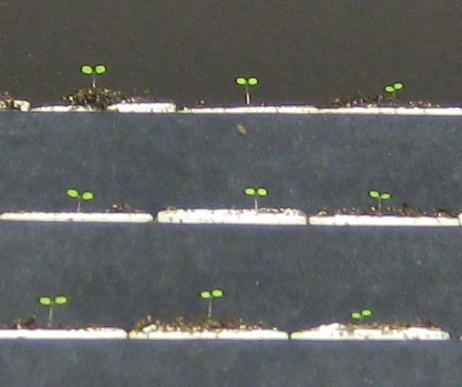

Supplement: Additional file 15 — TRiP. Compressed folder containing the TRiP code including a ReadMe file and sample image data. [file 13007_2015_75_MOESM15_ESM.zip › TRiP/input/C010040.jpg]

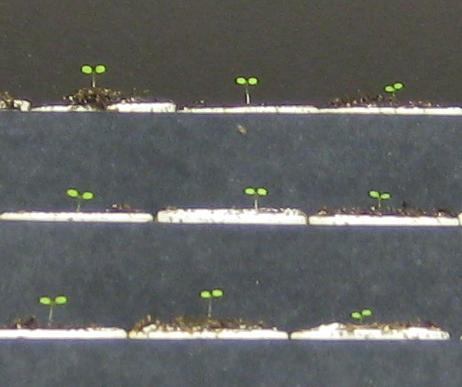

Supplement: Additional file 15 — TRiP. Compressed folder containing the TRiP code including a ReadMe file and sample image data. [file 13007_2015_75_MOESM15_ESM.zip › TRiP/input/C010041.jpg]

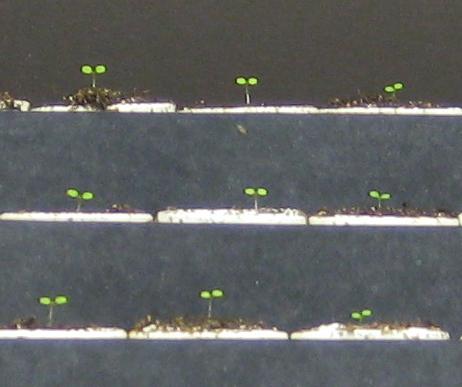

Supplement: Additional file 15 — TRiP. Compressed folder containing the TRiP code including a ReadMe file and sample image data. [file 13007_2015_75_MOESM15_ESM.zip › TRiP/input/C010042.jpg]

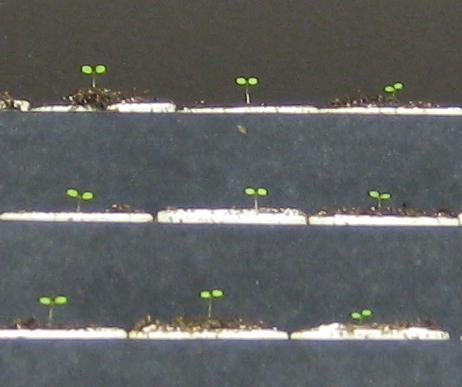

Supplement: Additional file 15 — TRiP. Compressed folder containing the TRiP code including a ReadMe file and sample image data. [file 13007_2015_75_MOESM15_ESM.zip › TRiP/input/C010043.jpg]

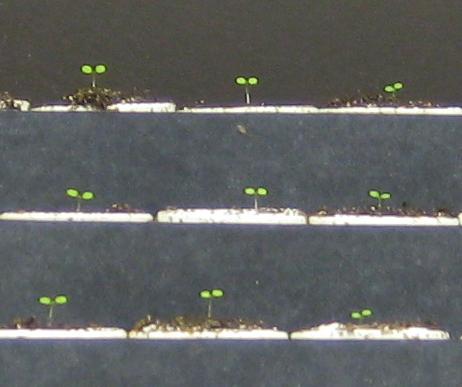

Supplement: Additional file 15 — TRiP. Compressed folder containing the TRiP code including a ReadMe file and sample image data. [file 13007_2015_75_MOESM15_ESM.zip › TRiP/input/C010044.jpg]

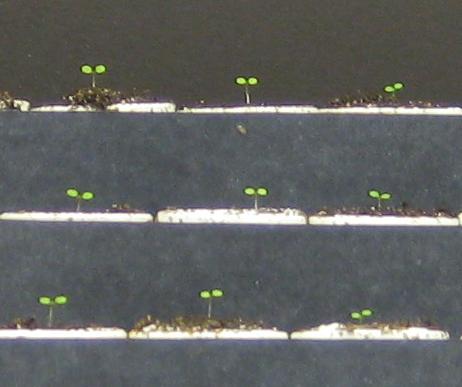

Supplement: Additional file 15 — TRiP. Compressed folder containing the TRiP code including a ReadMe file and sample image data. [file 13007_2015_75_MOESM15_ESM.zip › TRiP/input/C010045.jpg]

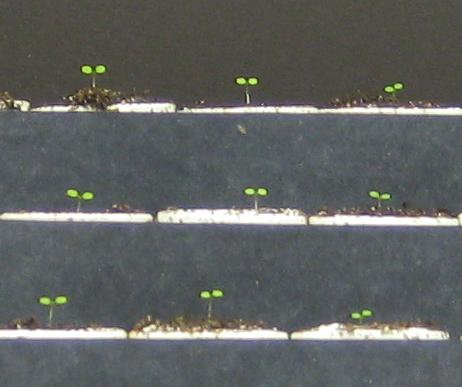

Supplement: Additional file 15 — TRiP. Compressed folder containing the TRiP code including a ReadMe file and sample image data. [file 13007_2015_75_MOESM15_ESM.zip › TRiP/input/C010046.jpg]

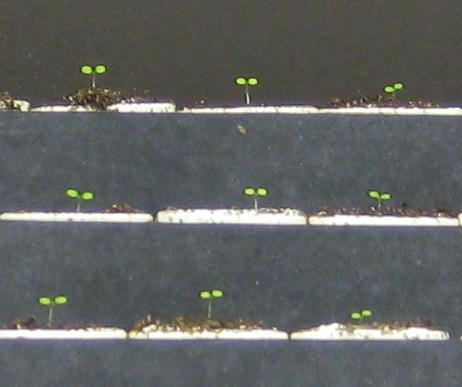

Supplement: Additional file 15 — TRiP. Compressed folder containing the TRiP code including a ReadMe file and sample image data. [file 13007_2015_75_MOESM15_ESM.zip › TRiP/input/C010047.jpg]

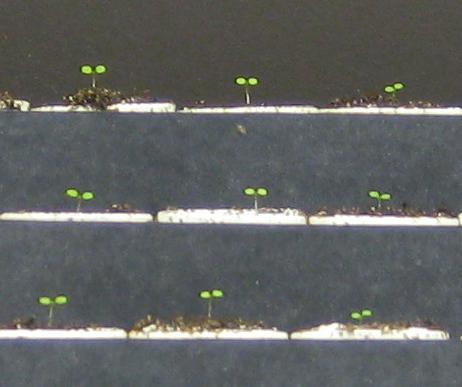

Supplement: Additional file 15 — TRiP. Compressed folder containing the TRiP code including a ReadMe file and sample image data. [file 13007_2015_75_MOESM15_ESM.zip › TRiP/input/C010048.jpg]

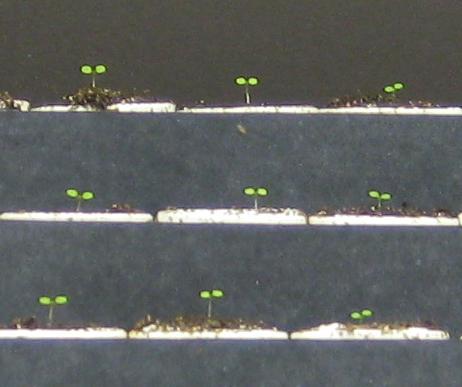

Supplement: Additional file 15 — TRiP. Compressed folder containing the TRiP code including a ReadMe file and sample image data. [file 13007_2015_75_MOESM15_ESM.zip › TRiP/input/C010049.jpg]

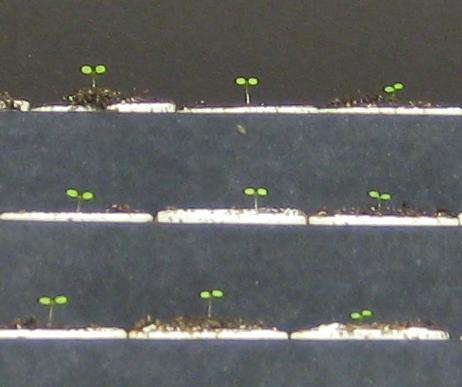

Supplement: Additional file 15 — TRiP. Compressed folder containing the TRiP code including a ReadMe file and sample image data. [file 13007_2015_75_MOESM15_ESM.zip › TRiP/input/C010050.jpg]

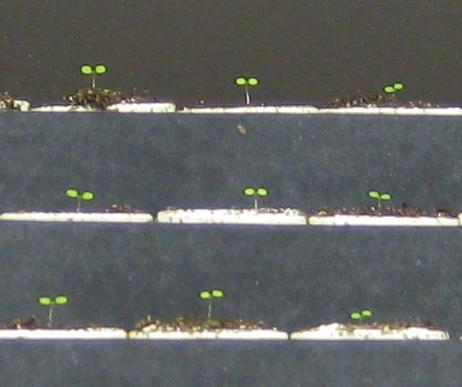

Supplement: Additional file 15 — TRiP. Compressed folder containing the TRiP code including a ReadMe file and sample image data. [file 13007_2015_75_MOESM15_ESM.zip › TRiP/input/C010051.jpg]

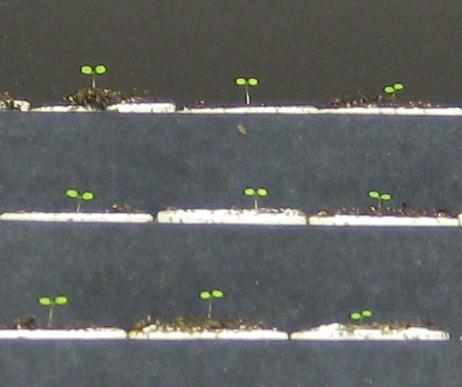

Supplement: Additional file 15 — TRiP. Compressed folder containing the TRiP code including a ReadMe file and sample image data. [file 13007_2015_75_MOESM15_ESM.zip › TRiP/input/C010052.jpg]

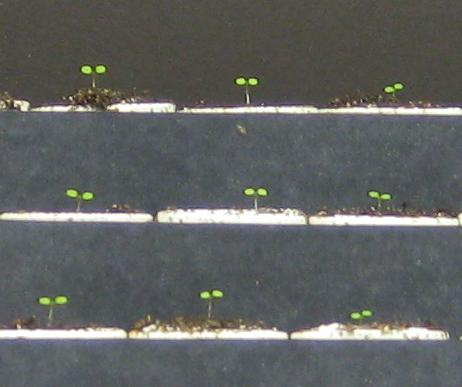

Supplement: Additional file 15 — TRiP. Compressed folder containing the TRiP code including a ReadMe file and sample image data. [file 13007_2015_75_MOESM15_ESM.zip › TRiP/input/C010053.jpg]

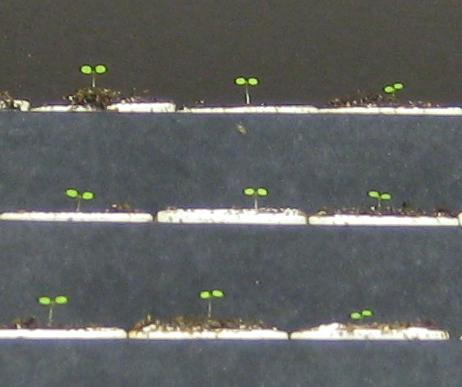

Supplement: Additional file 15 — TRiP. Compressed folder containing the TRiP code including a ReadMe file and sample image data. [file 13007_2015_75_MOESM15_ESM.zip › TRiP/input/C010054.jpg]

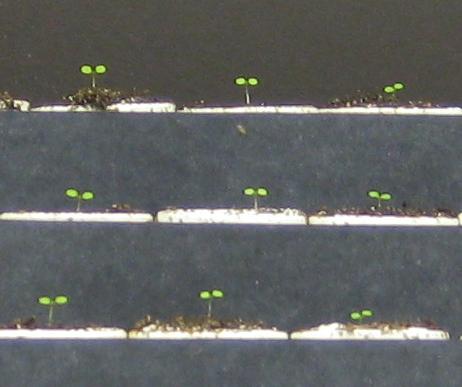

Supplement: Additional file 15 — TRiP. Compressed folder containing the TRiP code including a ReadMe file and sample image data. [file 13007_2015_75_MOESM15_ESM.zip › TRiP/input/C010055.jpg]

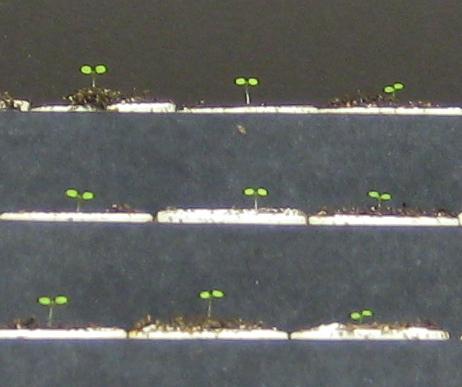

Supplement: Additional file 15 — TRiP. Compressed folder containing the TRiP code including a ReadMe file and sample image data. [file 13007_2015_75_MOESM15_ESM.zip › TRiP/input/C010056.jpg]

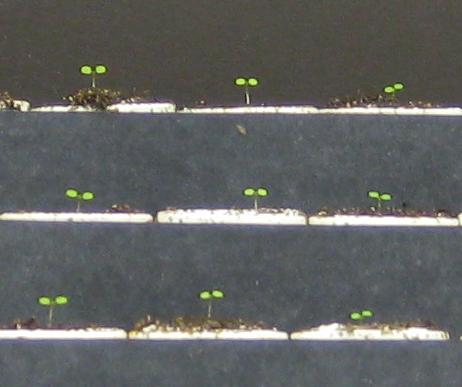

Supplement: Additional file 15 — TRiP. Compressed folder containing the TRiP code including a ReadMe file and sample image data. [file 13007_2015_75_MOESM15_ESM.zip › TRiP/input/C010057.jpg]

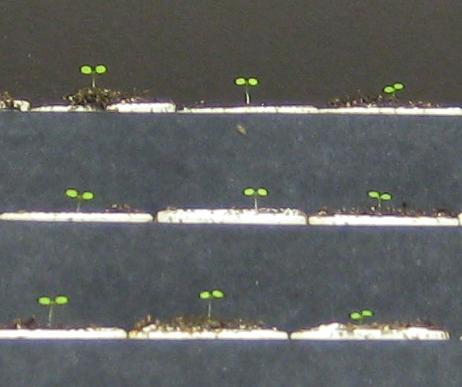

Supplement: Additional file 15 — TRiP. Compressed folder containing the TRiP code including a ReadMe file and sample image data. [file 13007_2015_75_MOESM15_ESM.zip › TRiP/input/C010058.jpg]

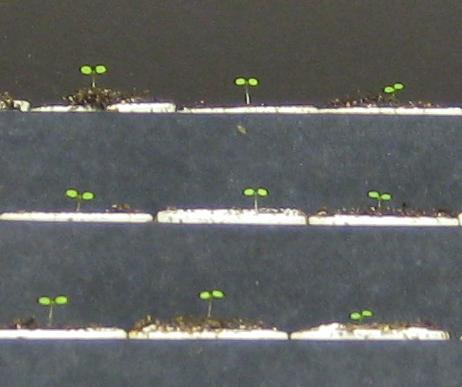

Supplement: Additional file 15 — TRiP. Compressed folder containing the TRiP code including a ReadMe file and sample image data. [file 13007_2015_75_MOESM15_ESM.zip › TRiP/input/C010059.jpg]

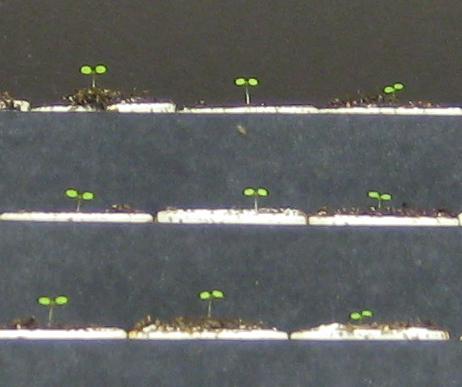

Supplement: Additional file 15 — TRiP. Compressed folder containing the TRiP code including a ReadMe file and sample image data. [file 13007_2015_75_MOESM15_ESM.zip › TRiP/input/C010060.jpg]

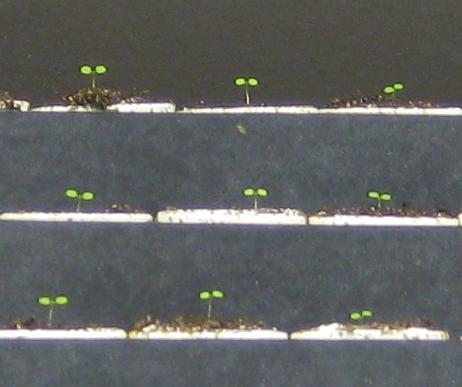

Supplement: Additional file 15 — TRiP. Compressed folder containing the TRiP code including a ReadMe file and sample image data. [file 13007_2015_75_MOESM15_ESM.zip › TRiP/input/C010061.jpg]

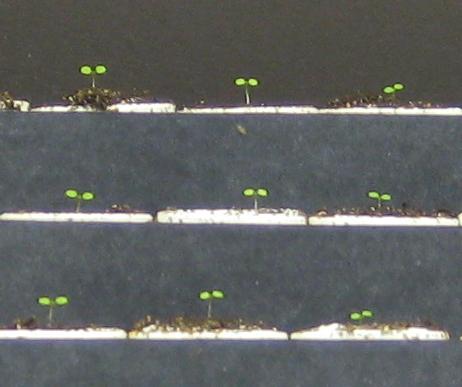

Supplement: Additional file 15 — TRiP. Compressed folder containing the TRiP code including a ReadMe file and sample image data. [file 13007_2015_75_MOESM15_ESM.zip › TRiP/input/C010062.jpg]

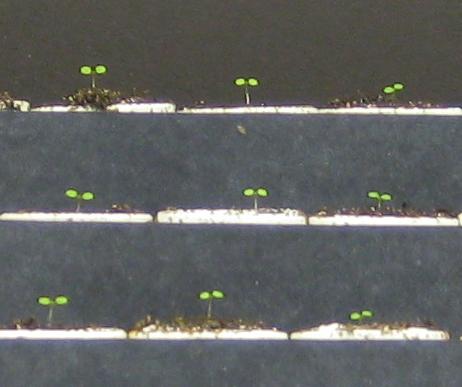

Supplement: Additional file 15 — TRiP. Compressed folder containing the TRiP code including a ReadMe file and sample image data. [file 13007_2015_75_MOESM15_ESM.zip › TRiP/input/C010063.jpg]

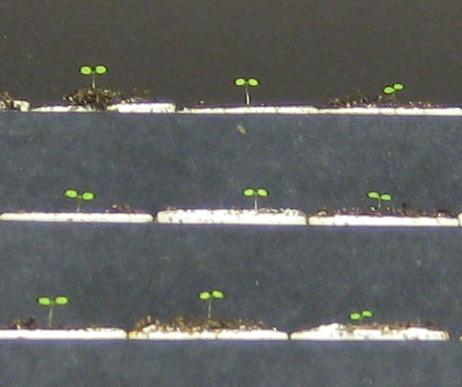

Supplement: Additional file 15 — TRiP. Compressed folder containing the TRiP code including a ReadMe file and sample image data. [file 13007_2015_75_MOESM15_ESM.zip › TRiP/input/C010064.jpg]

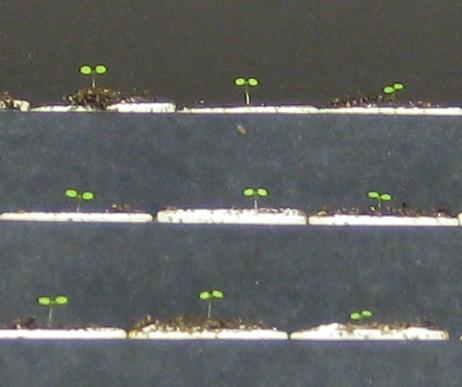

Supplement: Additional file 15 — TRiP. Compressed folder containing the TRiP code including a ReadMe file and sample image data. [file 13007_2015_75_MOESM15_ESM.zip › TRiP/input/C010065.jpg]

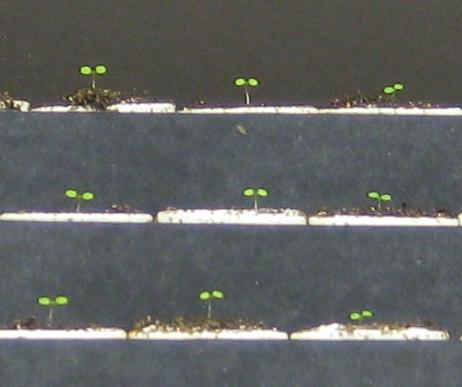

Supplement: Additional file 15 — TRiP. Compressed folder containing the TRiP code including a ReadMe file and sample image data. [file 13007_2015_75_MOESM15_ESM.zip › TRiP/input/C010066.jpg]

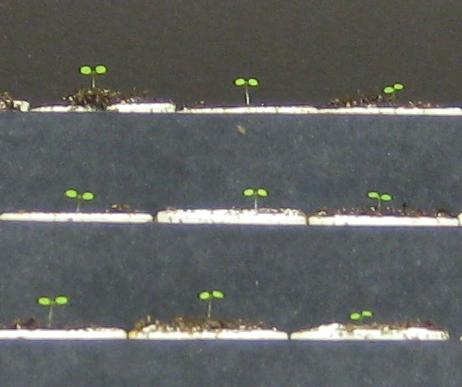

Supplement: Additional file 15 — TRiP. Compressed folder containing the TRiP code including a ReadMe file and sample image data. [file 13007_2015_75_MOESM15_ESM.zip › TRiP/input/C010067.jpg]

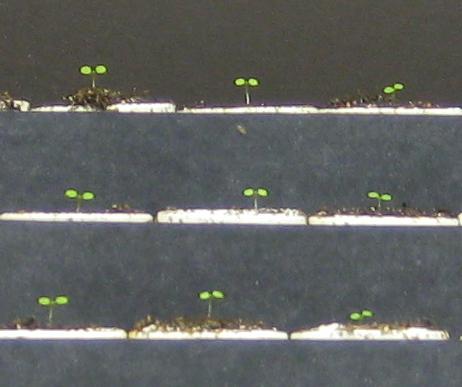

Supplement: Additional file 15 — TRiP. Compressed folder containing the TRiP code including a ReadMe file and sample image data. [file 13007_2015_75_MOESM15_ESM.zip › TRiP/input/C010068.jpg]

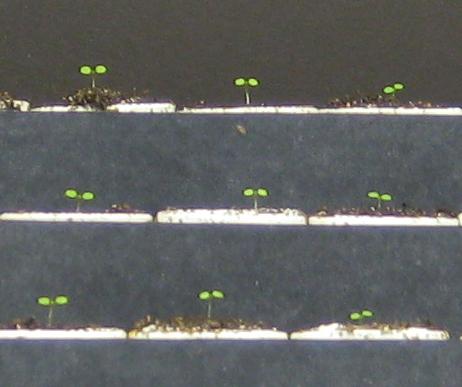

Supplement: Additional file 15 — TRiP. Compressed folder containing the TRiP code including a ReadMe file and sample image data. [file 13007_2015_75_MOESM15_ESM.zip › TRiP/input/C010069.jpg]

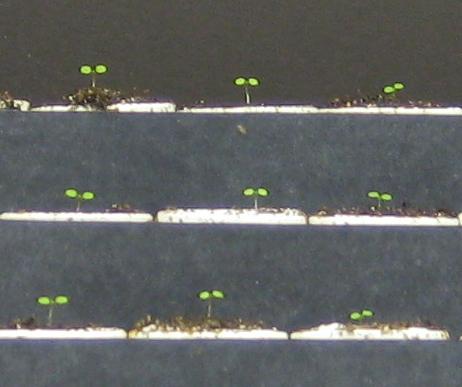

Supplement: Additional file 15 — TRiP. Compressed folder containing the TRiP code including a ReadMe file and sample image data. [file 13007_2015_75_MOESM15_ESM.zip › TRiP/input/C010070.jpg]

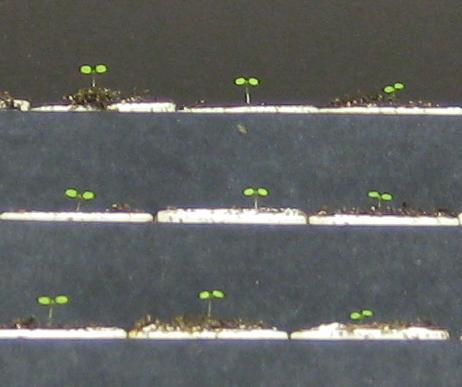

Supplement: Additional file 15 — TRiP. Compressed folder containing the TRiP code including a ReadMe file and sample image data. [file 13007_2015_75_MOESM15_ESM.zip › TRiP/input/C010071.jpg]

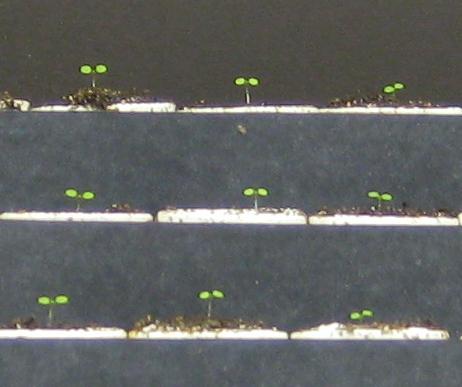

Supplement: Additional file 15 — TRiP. Compressed folder containing the TRiP code including a ReadMe file and sample image data. [file 13007_2015_75_MOESM15_ESM.zip › TRiP/input/C010072.jpg]

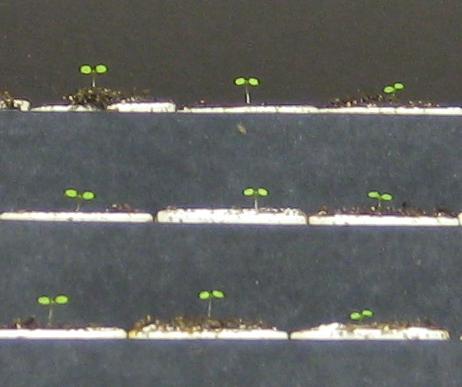

Supplement: Additional file 15 — TRiP. Compressed folder containing the TRiP code including a ReadMe file and sample image data. [file 13007_2015_75_MOESM15_ESM.zip › TRiP/input/C010073.jpg]

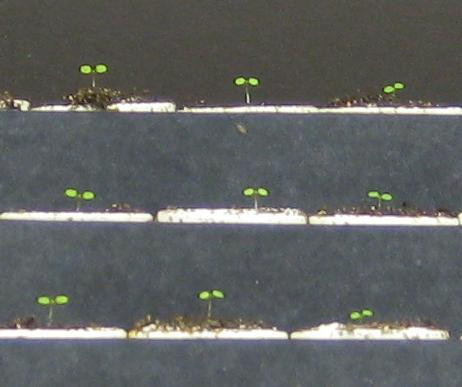

Supplement: Additional file 15 — TRiP. Compressed folder containing the TRiP code including a ReadMe file and sample image data. [file 13007_2015_75_MOESM15_ESM.zip › TRiP/input/C010074.jpg]

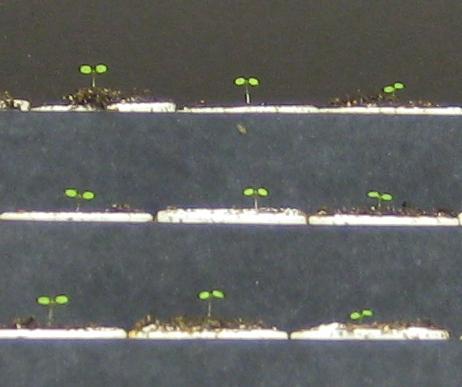

Supplement: Additional file 15 — TRiP. Compressed folder containing the TRiP code including a ReadMe file and sample image data. [file 13007_2015_75_MOESM15_ESM.zip › TRiP/input/C010075.jpg]

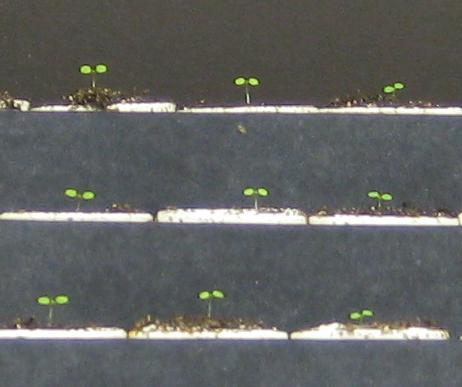

Supplement: Additional file 15 — TRiP. Compressed folder containing the TRiP code including a ReadMe file and sample image data. [file 13007_2015_75_MOESM15_ESM.zip › TRiP/input/C010076.jpg]

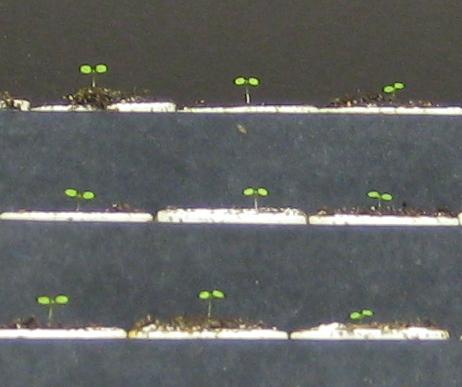

Supplement: Additional file 15 — TRiP. Compressed folder containing the TRiP code including a ReadMe file and sample image data. [file 13007_2015_75_MOESM15_ESM.zip › TRiP/input/C010077.jpg]

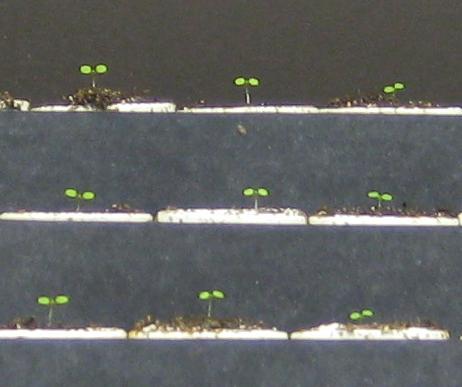

Supplement: Additional file 15 — TRiP. Compressed folder containing the TRiP code including a ReadMe file and sample image data. [file 13007_2015_75_MOESM15_ESM.zip › TRiP/input/C010078.jpg]

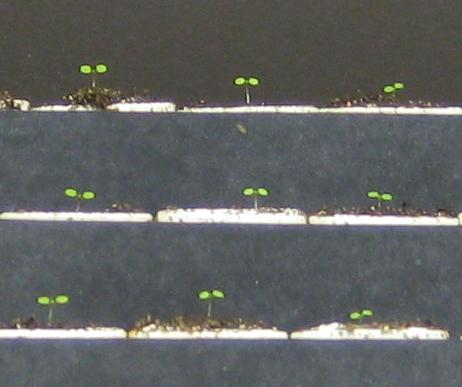

Supplement: Additional file 15 — TRiP. Compressed folder containing the TRiP code including a ReadMe file and sample image data. [file 13007_2015_75_MOESM15_ESM.zip › TRiP/input/C010079.jpg]

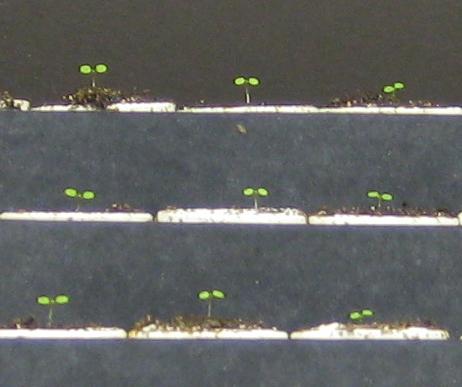

Supplement: Additional file 15 — TRiP. Compressed folder containing the TRiP code including a ReadMe file and sample image data. [file 13007_2015_75_MOESM15_ESM.zip › TRiP/input/C010080.jpg]

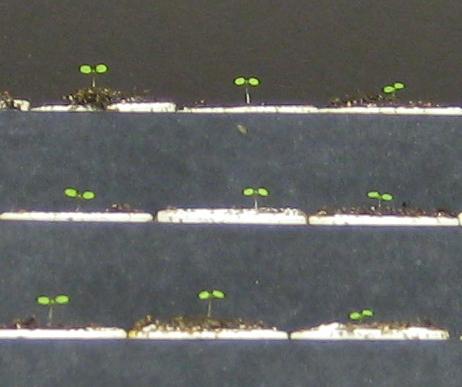

Supplement: Additional file 15 — TRiP. Compressed folder containing the TRiP code including a ReadMe file and sample image data. [file 13007_2015_75_MOESM15_ESM.zip › TRiP/input/C010081.jpg]

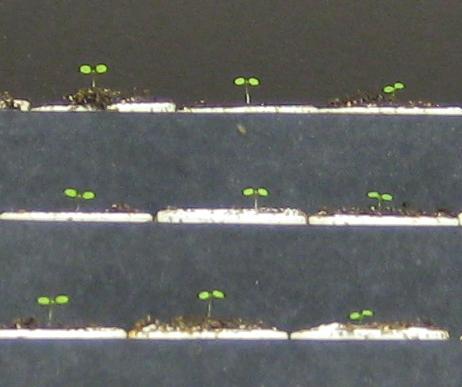

Supplement: Additional file 15 — TRiP. Compressed folder containing the TRiP code including a ReadMe file and sample image data. [file 13007_2015_75_MOESM15_ESM.zip › TRiP/input/C010082.jpg]

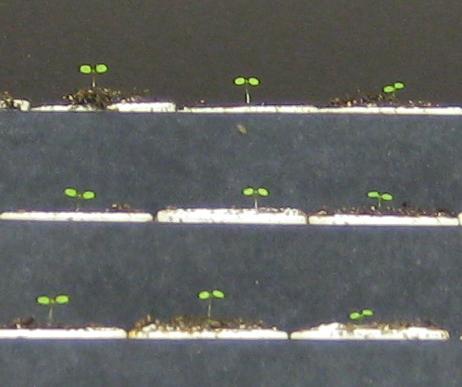

Supplement: Additional file 15 — TRiP. Compressed folder containing the TRiP code including a ReadMe file and sample image data. [file 13007_2015_75_MOESM15_ESM.zip › TRiP/input/C010083.jpg]

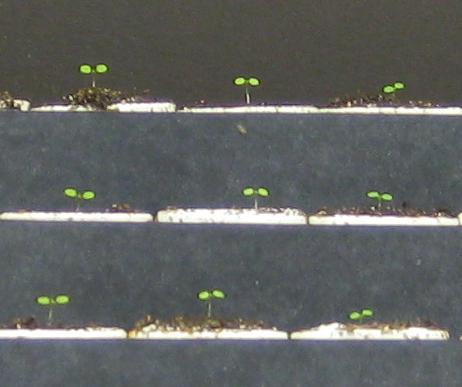

Supplement: Additional file 15 — TRiP. Compressed folder containing the TRiP code including a ReadMe file and sample image data. [file 13007_2015_75_MOESM15_ESM.zip › TRiP/input/C010084.jpg]

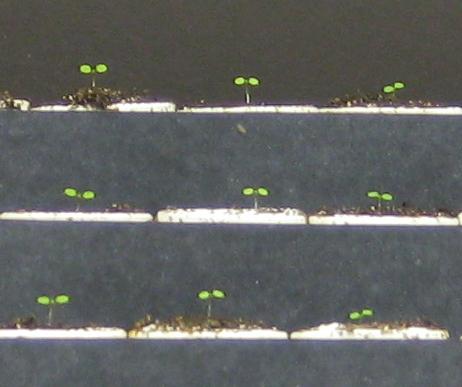

Supplement: Additional file 15 — TRiP. Compressed folder containing the TRiP code including a ReadMe file and sample image data. [file 13007_2015_75_MOESM15_ESM.zip › TRiP/input/C010085.jpg]

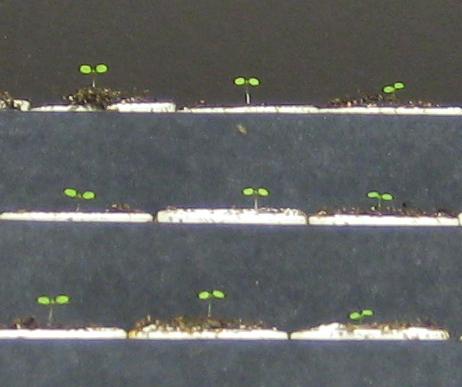

Supplement: Additional file 15 — TRiP. Compressed folder containing the TRiP code including a ReadMe file and sample image data. [file 13007_2015_75_MOESM15_ESM.zip › TRiP/input/C010086.jpg]

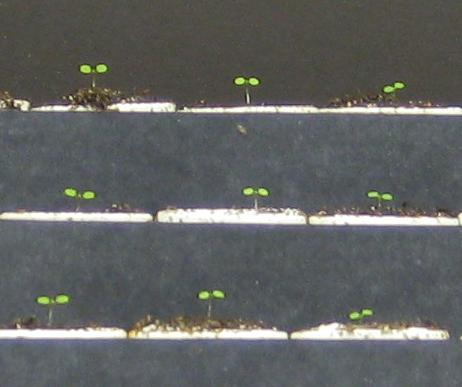

Supplement: Additional file 15 — TRiP. Compressed folder containing the TRiP code including a ReadMe file and sample image data. [file 13007_2015_75_MOESM15_ESM.zip › TRiP/input/C010087.jpg]

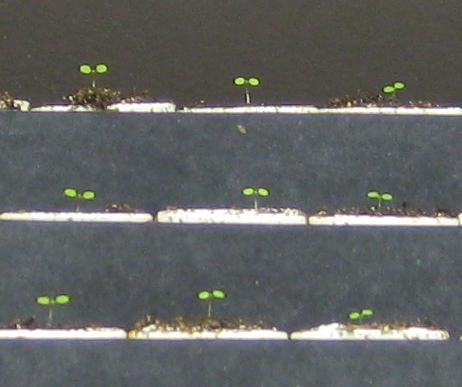

Supplement: Additional file 15 — TRiP. Compressed folder containing the TRiP code including a ReadMe file and sample image data. [file 13007_2015_75_MOESM15_ESM.zip › TRiP/input/C010088.jpg]

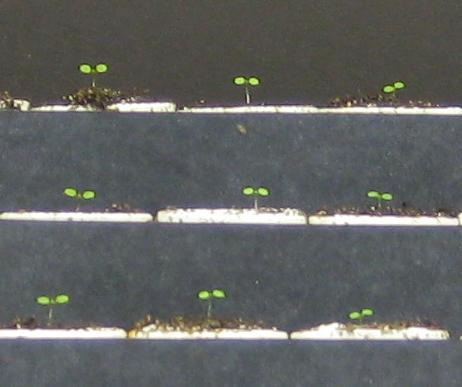

Supplement: Additional file 15 — TRiP. Compressed folder containing the TRiP code including a ReadMe file and sample image data. [file 13007_2015_75_MOESM15_ESM.zip › TRiP/input/C010089.jpg]

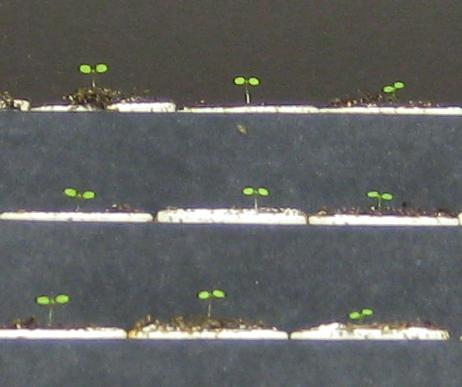

Supplement: Additional file 15 — TRiP. Compressed folder containing the TRiP code including a ReadMe file and sample image data. [file 13007_2015_75_MOESM15_ESM.zip › TRiP/input/C010090.jpg]

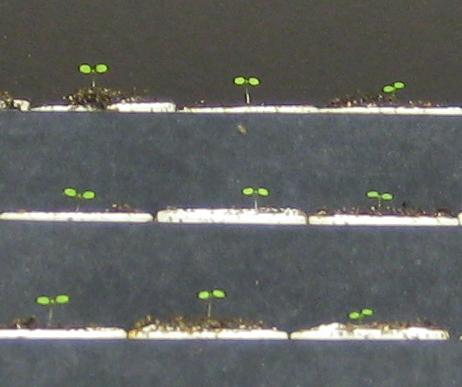

Supplement: Additional file 15 — TRiP. Compressed folder containing the TRiP code including a ReadMe file and sample image data. [file 13007_2015_75_MOESM15_ESM.zip › TRiP/input/C010091.jpg]

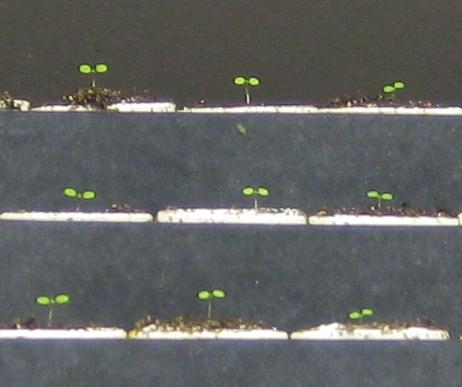

Supplement: Additional file 15 — TRiP. Compressed folder containing the TRiP code including a ReadMe file and sample image data. [file 13007_2015_75_MOESM15_ESM.zip › TRiP/input/C010092.jpg]

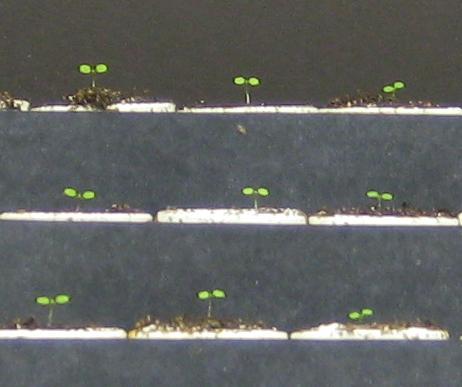

Supplement: Additional file 15 — TRiP. Compressed folder containing the TRiP code including a ReadMe file and sample image data. [file 13007_2015_75_MOESM15_ESM.zip › TRiP/input/C010093.jpg]

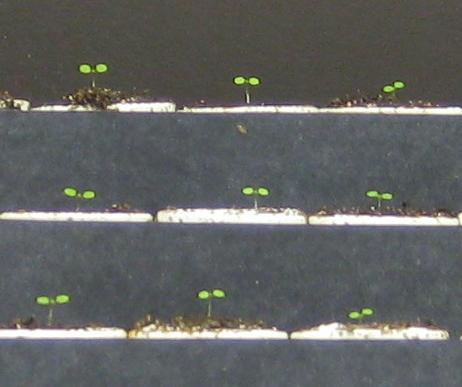

Supplement: Additional file 15 — TRiP. Compressed folder containing the TRiP code including a ReadMe file and sample image data. [file 13007_2015_75_MOESM15_ESM.zip › TRiP/input/C010094.jpg]

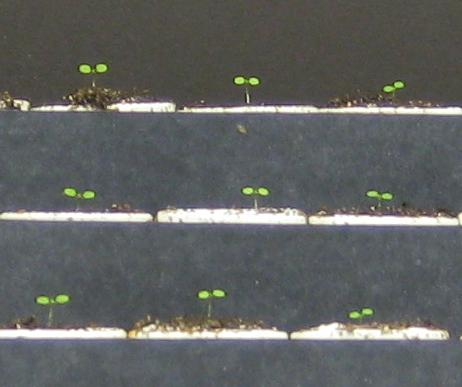

Supplement: Additional file 15 — TRiP. Compressed folder containing the TRiP code including a ReadMe file and sample image data. [file 13007_2015_75_MOESM15_ESM.zip › TRiP/input/C010095.jpg]

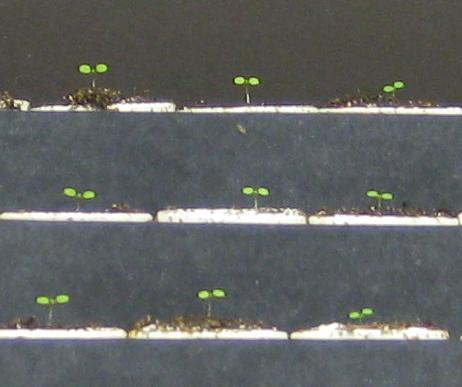

Supplement: Additional file 15 — TRiP. Compressed folder containing the TRiP code including a ReadMe file and sample image data. [file 13007_2015_75_MOESM15_ESM.zip › TRiP/input/C010096.jpg]

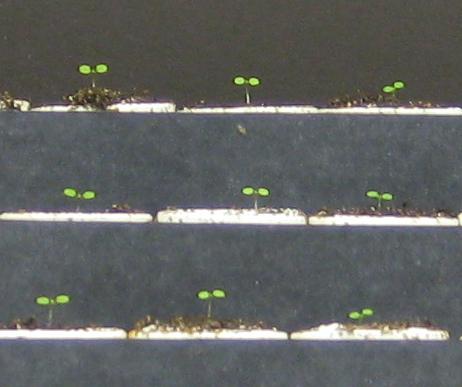

Supplement: Additional file 15 — TRiP. Compressed folder containing the TRiP code including a ReadMe file and sample image data. [file 13007_2015_75_MOESM15_ESM.zip › TRiP/input/C010097.jpg]

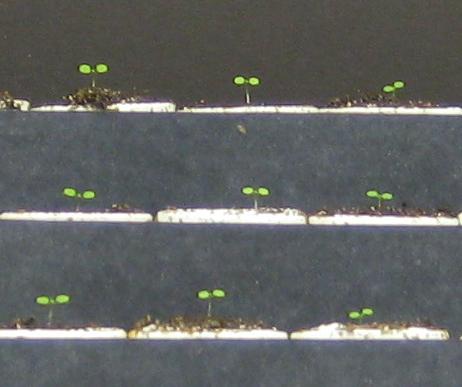

Supplement: Additional file 15 — TRiP. Compressed folder containing the TRiP code including a ReadMe file and sample image data. [file 13007_2015_75_MOESM15_ESM.zip › TRiP/input/C010098.jpg]

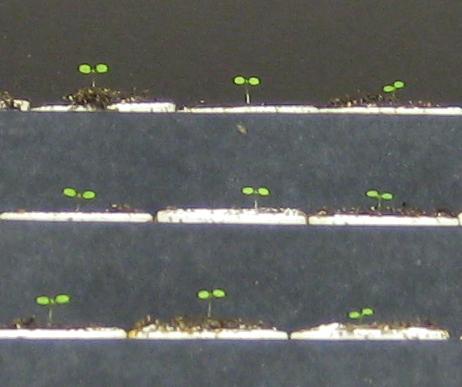

Supplement: Additional file 15 — TRiP. Compressed folder containing the TRiP code including a ReadMe file and sample image data. [file 13007_2015_75_MOESM15_ESM.zip › TRiP/input/C010099.jpg]
