# Supplementary figures and images for: TRiP: Tracking Rhythms in Plants, an automated leaf movement analysis program for circadian period estimation (part 2 of 10)
Source: Plant Methods. 2015 May 3;11:33. doi: 10.1186/s13007-015-0075-5 (PMC4445800; doi:10.1186/s13007-015-0075-5)

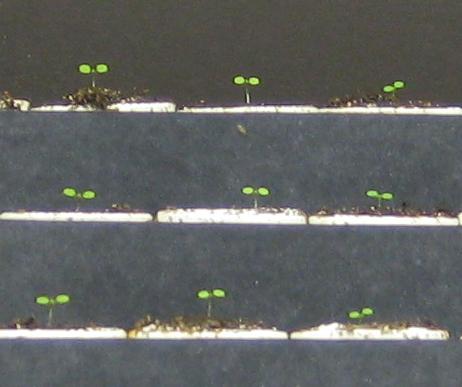

Supplement: Additional file 15 — TRiP. Compressed folder containing the TRiP code including a ReadMe file and sample image data. [file 13007_2015_75_MOESM15_ESM.zip › TRiP/input/C010100.jpg]

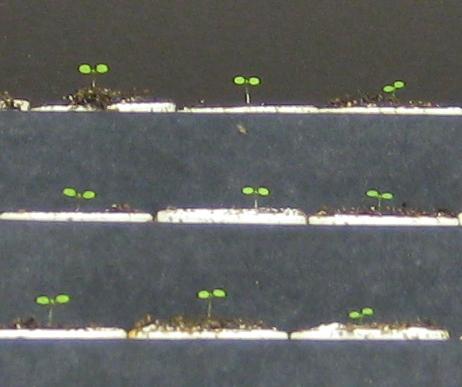

Supplement: Additional file 15 — TRiP. Compressed folder containing the TRiP code including a ReadMe file and sample image data. [file 13007_2015_75_MOESM15_ESM.zip › TRiP/input/C010101.jpg]

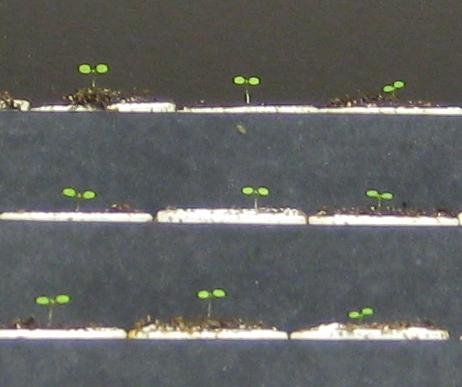

Supplement: Additional file 15 — TRiP. Compressed folder containing the TRiP code including a ReadMe file and sample image data. [file 13007_2015_75_MOESM15_ESM.zip › TRiP/input/C010102.jpg]

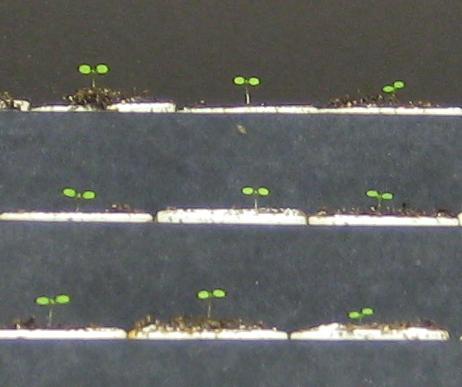

Supplement: Additional file 15 — TRiP. Compressed folder containing the TRiP code including a ReadMe file and sample image data. [file 13007_2015_75_MOESM15_ESM.zip › TRiP/input/C010103.jpg]

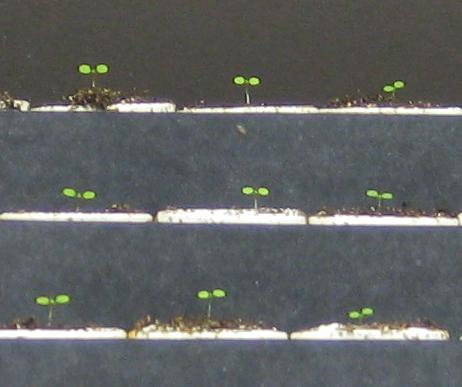

Supplement: Additional file 15 — TRiP. Compressed folder containing the TRiP code including a ReadMe file and sample image data. [file 13007_2015_75_MOESM15_ESM.zip › TRiP/input/C010104.jpg]

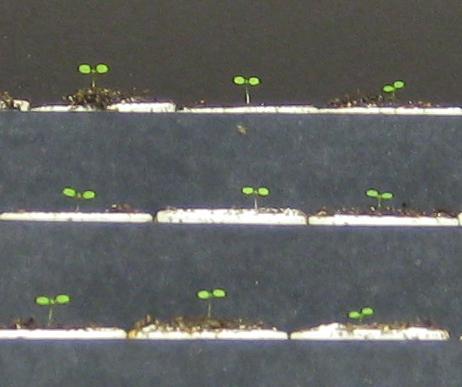

Supplement: Additional file 15 — TRiP. Compressed folder containing the TRiP code including a ReadMe file and sample image data. [file 13007_2015_75_MOESM15_ESM.zip › TRiP/input/C010105.jpg]

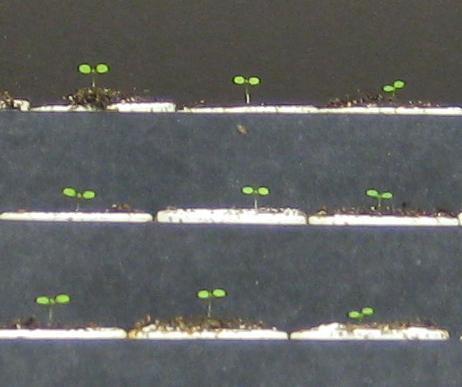

Supplement: Additional file 15 — TRiP. Compressed folder containing the TRiP code including a ReadMe file and sample image data. [file 13007_2015_75_MOESM15_ESM.zip › TRiP/input/C010106.jpg]

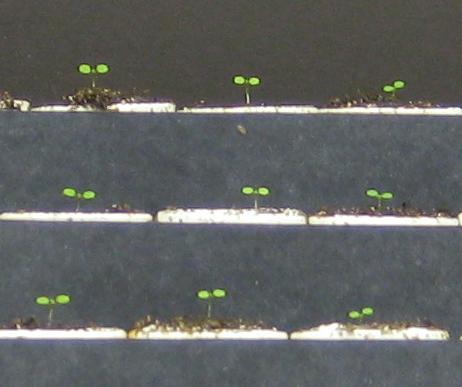

Supplement: Additional file 15 — TRiP. Compressed folder containing the TRiP code including a ReadMe file and sample image data. [file 13007_2015_75_MOESM15_ESM.zip › TRiP/input/C010107.jpg]

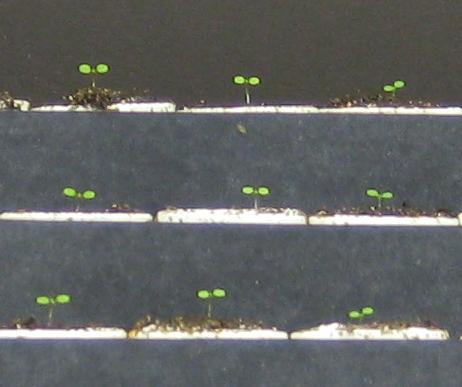

Supplement: Additional file 15 — TRiP. Compressed folder containing the TRiP code including a ReadMe file and sample image data. [file 13007_2015_75_MOESM15_ESM.zip › TRiP/input/C010108.jpg]

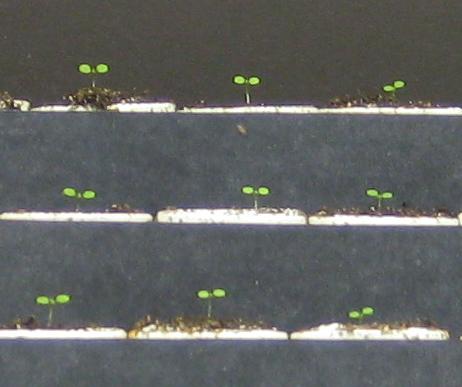

Supplement: Additional file 15 — TRiP. Compressed folder containing the TRiP code including a ReadMe file and sample image data. [file 13007_2015_75_MOESM15_ESM.zip › TRiP/input/C010109.jpg]

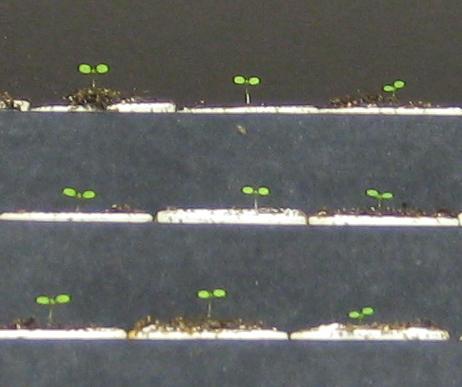

Supplement: Additional file 15 — TRiP. Compressed folder containing the TRiP code including a ReadMe file and sample image data. [file 13007_2015_75_MOESM15_ESM.zip › TRiP/input/C010110.jpg]

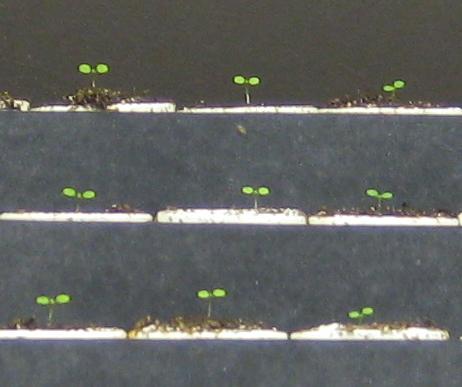

Supplement: Additional file 15 — TRiP. Compressed folder containing the TRiP code including a ReadMe file and sample image data. [file 13007_2015_75_MOESM15_ESM.zip › TRiP/input/C010111.jpg]

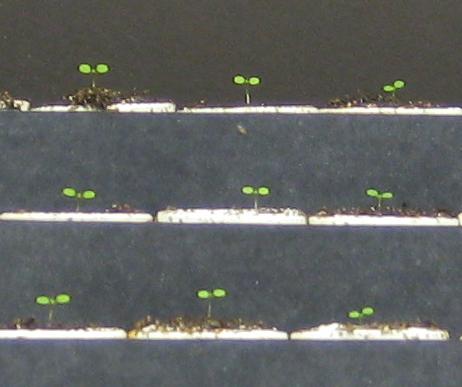

Supplement: Additional file 15 — TRiP. Compressed folder containing the TRiP code including a ReadMe file and sample image data. [file 13007_2015_75_MOESM15_ESM.zip › TRiP/input/C010112.jpg]

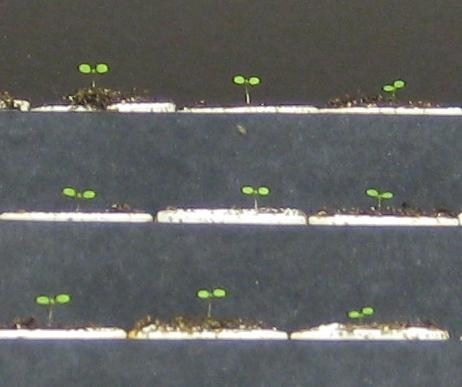

Supplement: Additional file 15 — TRiP. Compressed folder containing the TRiP code including a ReadMe file and sample image data. [file 13007_2015_75_MOESM15_ESM.zip › TRiP/input/C010113.jpg]

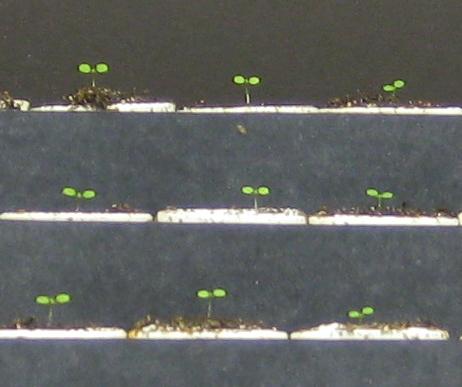

Supplement: Additional file 15 — TRiP. Compressed folder containing the TRiP code including a ReadMe file and sample image data. [file 13007_2015_75_MOESM15_ESM.zip › TRiP/input/C010114.jpg]

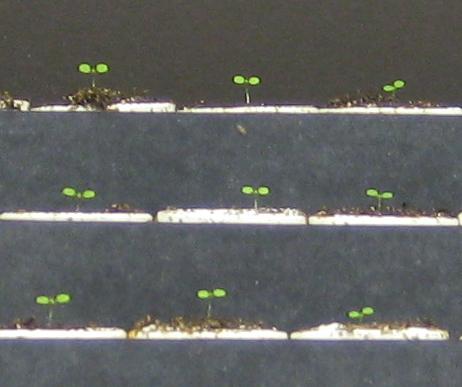

Supplement: Additional file 15 — TRiP. Compressed folder containing the TRiP code including a ReadMe file and sample image data. [file 13007_2015_75_MOESM15_ESM.zip › TRiP/input/C010115.jpg]

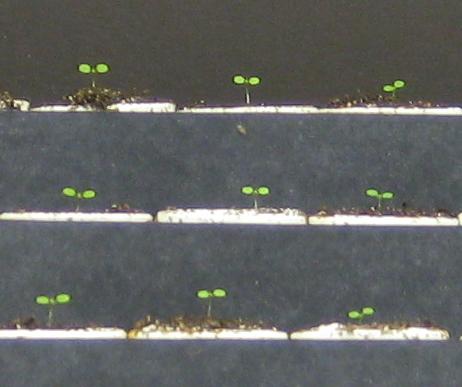

Supplement: Additional file 15 — TRiP. Compressed folder containing the TRiP code including a ReadMe file and sample image data. [file 13007_2015_75_MOESM15_ESM.zip › TRiP/input/C010116.jpg]

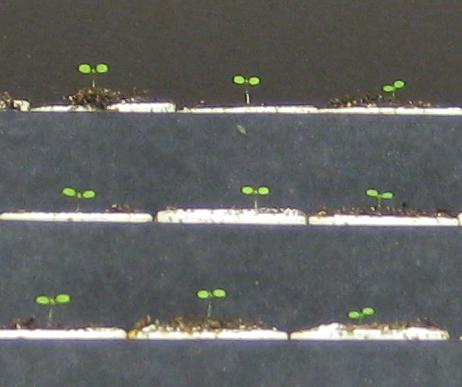

Supplement: Additional file 15 — TRiP. Compressed folder containing the TRiP code including a ReadMe file and sample image data. [file 13007_2015_75_MOESM15_ESM.zip › TRiP/input/C010117.jpg]

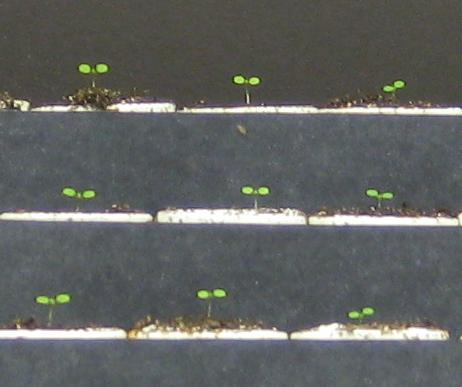

Supplement: Additional file 15 — TRiP. Compressed folder containing the TRiP code including a ReadMe file and sample image data. [file 13007_2015_75_MOESM15_ESM.zip › TRiP/input/C010118.jpg]

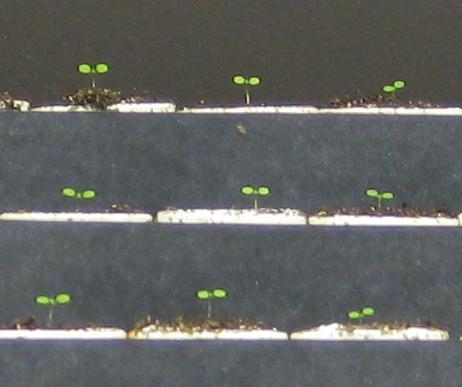

Supplement: Additional file 15 — TRiP. Compressed folder containing the TRiP code including a ReadMe file and sample image data. [file 13007_2015_75_MOESM15_ESM.zip › TRiP/input/C010119.jpg]

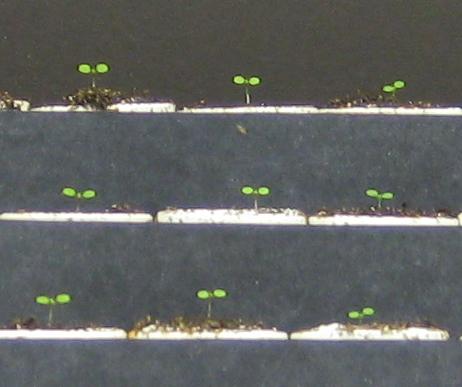

Supplement: Additional file 15 — TRiP. Compressed folder containing the TRiP code including a ReadMe file and sample image data. [file 13007_2015_75_MOESM15_ESM.zip › TRiP/input/C010120.jpg]

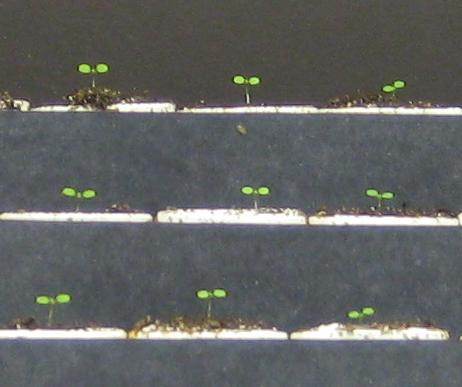

Supplement: Additional file 15 — TRiP. Compressed folder containing the TRiP code including a ReadMe file and sample image data. [file 13007_2015_75_MOESM15_ESM.zip › TRiP/input/C010121.jpg]

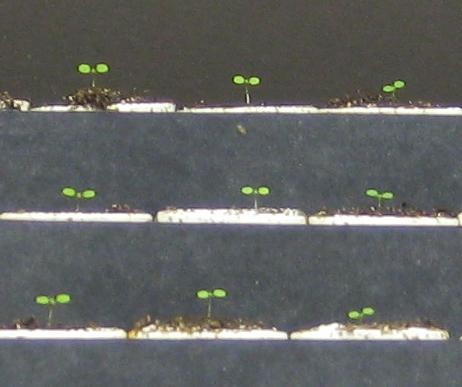

Supplement: Additional file 15 — TRiP. Compressed folder containing the TRiP code including a ReadMe file and sample image data. [file 13007_2015_75_MOESM15_ESM.zip › TRiP/input/C010122.jpg]

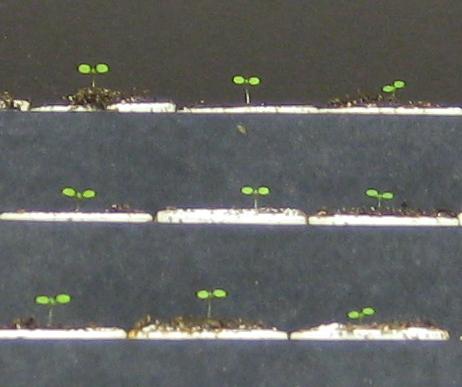

Supplement: Additional file 15 — TRiP. Compressed folder containing the TRiP code including a ReadMe file and sample image data. [file 13007_2015_75_MOESM15_ESM.zip › TRiP/input/C010123.jpg]

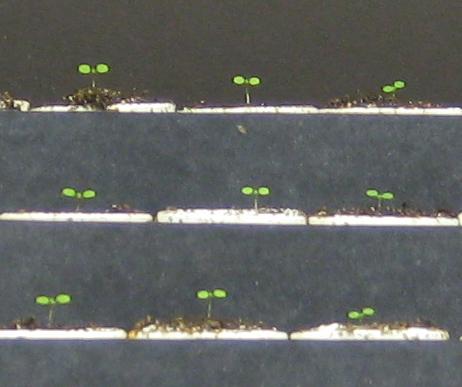

Supplement: Additional file 15 — TRiP. Compressed folder containing the TRiP code including a ReadMe file and sample image data. [file 13007_2015_75_MOESM15_ESM.zip › TRiP/input/C010124.jpg]

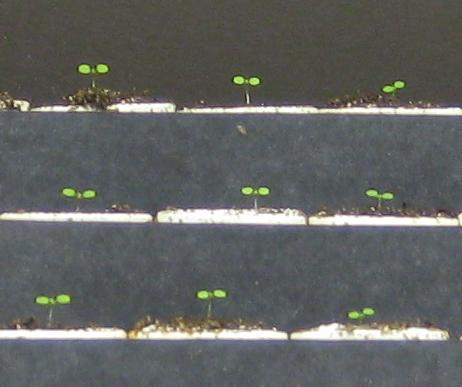

Supplement: Additional file 15 — TRiP. Compressed folder containing the TRiP code including a ReadMe file and sample image data. [file 13007_2015_75_MOESM15_ESM.zip › TRiP/input/C010125.jpg]

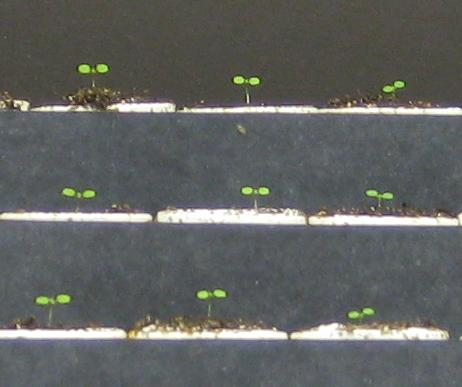

Supplement: Additional file 15 — TRiP. Compressed folder containing the TRiP code including a ReadMe file and sample image data. [file 13007_2015_75_MOESM15_ESM.zip › TRiP/input/C010126.jpg]

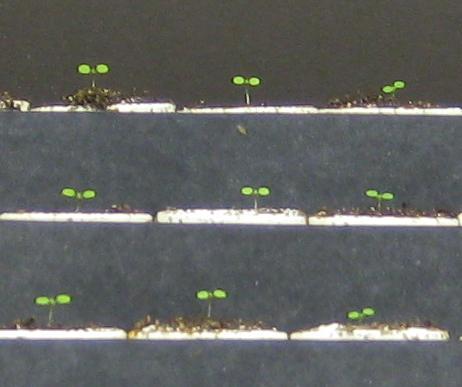

Supplement: Additional file 15 — TRiP. Compressed folder containing the TRiP code including a ReadMe file and sample image data. [file 13007_2015_75_MOESM15_ESM.zip › TRiP/input/C010127.jpg]

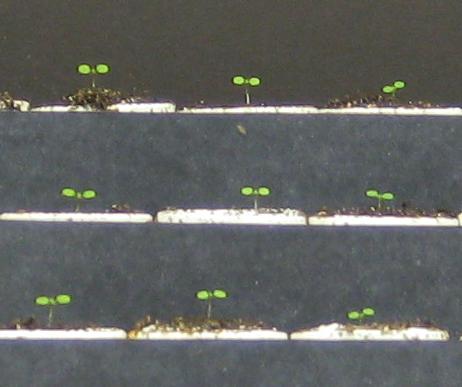

Supplement: Additional file 15 — TRiP. Compressed folder containing the TRiP code including a ReadMe file and sample image data. [file 13007_2015_75_MOESM15_ESM.zip › TRiP/input/C010128.jpg]

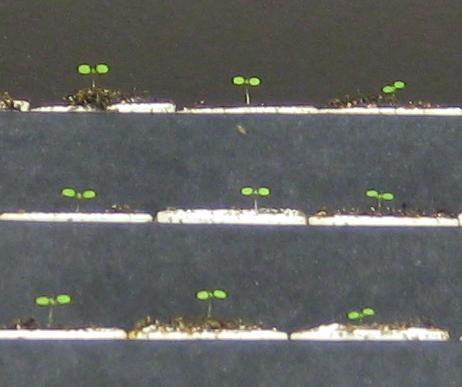

Supplement: Additional file 15 — TRiP. Compressed folder containing the TRiP code including a ReadMe file and sample image data. [file 13007_2015_75_MOESM15_ESM.zip › TRiP/input/C010129.jpg]

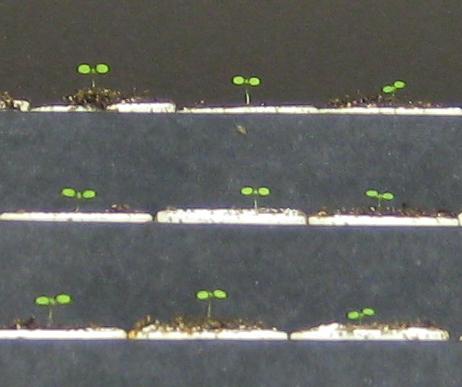

Supplement: Additional file 15 — TRiP. Compressed folder containing the TRiP code including a ReadMe file and sample image data. [file 13007_2015_75_MOESM15_ESM.zip › TRiP/input/C010130.jpg]

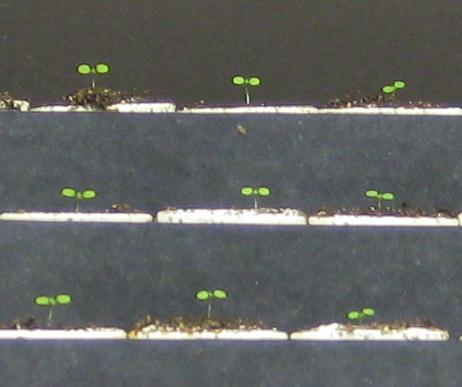

Supplement: Additional file 15 — TRiP. Compressed folder containing the TRiP code including a ReadMe file and sample image data. [file 13007_2015_75_MOESM15_ESM.zip › TRiP/input/C010131.jpg]

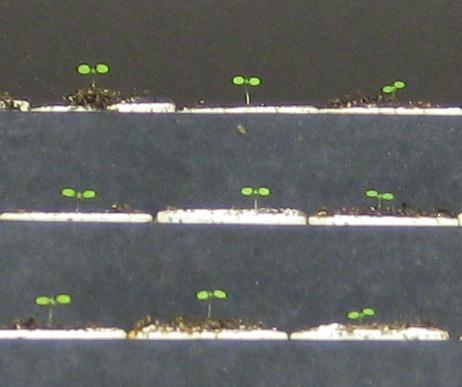

Supplement: Additional file 15 — TRiP. Compressed folder containing the TRiP code including a ReadMe file and sample image data. [file 13007_2015_75_MOESM15_ESM.zip › TRiP/input/C010132.jpg]

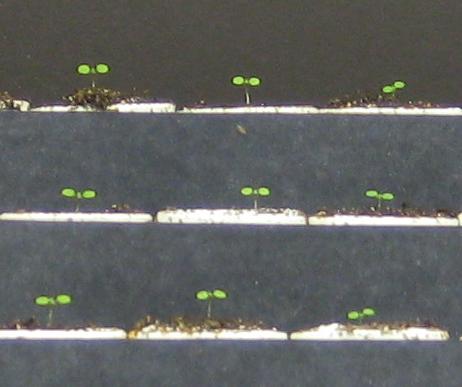

Supplement: Additional file 15 — TRiP. Compressed folder containing the TRiP code including a ReadMe file and sample image data. [file 13007_2015_75_MOESM15_ESM.zip › TRiP/input/C010133.jpg]

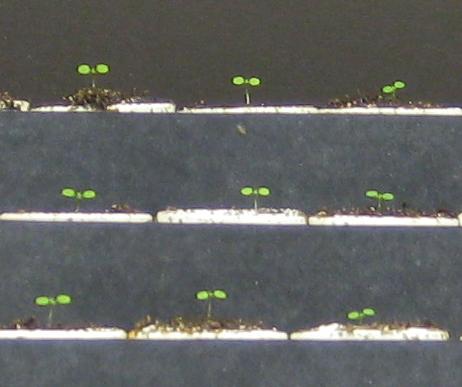

Supplement: Additional file 15 — TRiP. Compressed folder containing the TRiP code including a ReadMe file and sample image data. [file 13007_2015_75_MOESM15_ESM.zip › TRiP/input/C010134.jpg]

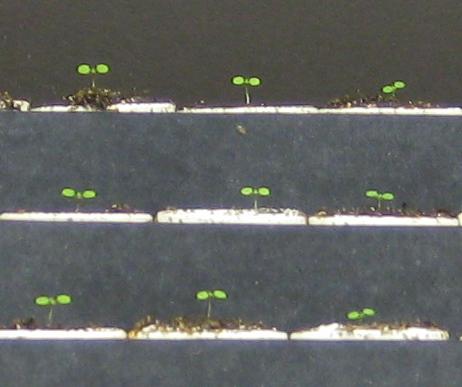

Supplement: Additional file 15 — TRiP. Compressed folder containing the TRiP code including a ReadMe file and sample image data. [file 13007_2015_75_MOESM15_ESM.zip › TRiP/input/C010135.jpg]

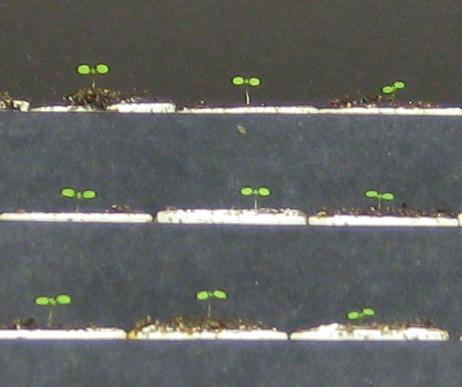

Supplement: Additional file 15 — TRiP. Compressed folder containing the TRiP code including a ReadMe file and sample image data. [file 13007_2015_75_MOESM15_ESM.zip › TRiP/input/C010136.jpg]

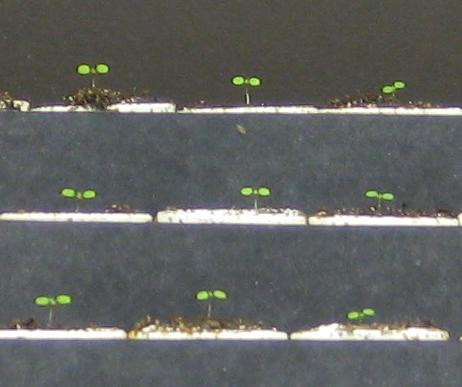

Supplement: Additional file 15 — TRiP. Compressed folder containing the TRiP code including a ReadMe file and sample image data. [file 13007_2015_75_MOESM15_ESM.zip › TRiP/input/C010137.jpg]

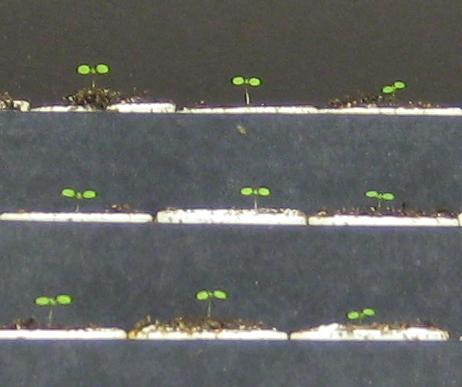

Supplement: Additional file 15 — TRiP. Compressed folder containing the TRiP code including a ReadMe file and sample image data. [file 13007_2015_75_MOESM15_ESM.zip › TRiP/input/C010138.jpg]

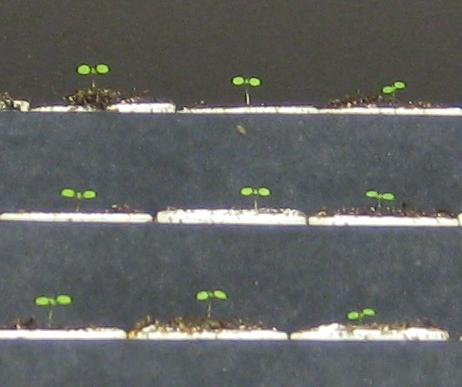

Supplement: Additional file 15 — TRiP. Compressed folder containing the TRiP code including a ReadMe file and sample image data. [file 13007_2015_75_MOESM15_ESM.zip › TRiP/input/C010139.jpg]

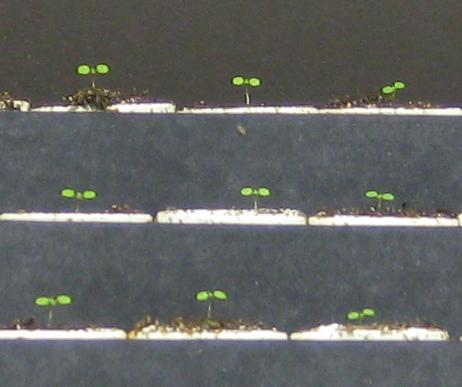

Supplement: Additional file 15 — TRiP. Compressed folder containing the TRiP code including a ReadMe file and sample image data. [file 13007_2015_75_MOESM15_ESM.zip › TRiP/input/C010140.jpg]

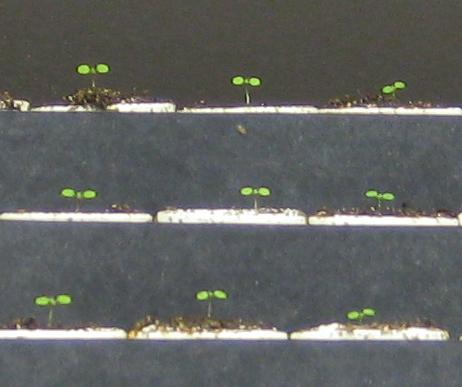

Supplement: Additional file 15 — TRiP. Compressed folder containing the TRiP code including a ReadMe file and sample image data. [file 13007_2015_75_MOESM15_ESM.zip › TRiP/input/C010141.jpg]

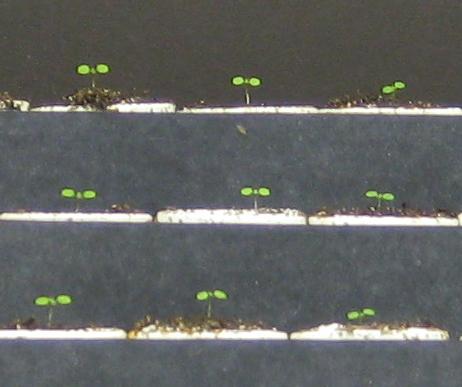

Supplement: Additional file 15 — TRiP. Compressed folder containing the TRiP code including a ReadMe file and sample image data. [file 13007_2015_75_MOESM15_ESM.zip › TRiP/input/C010142.jpg]

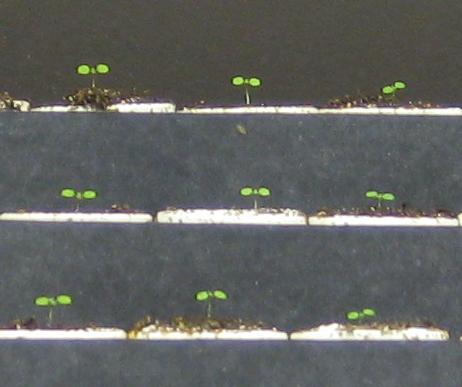

Supplement: Additional file 15 — TRiP. Compressed folder containing the TRiP code including a ReadMe file and sample image data. [file 13007_2015_75_MOESM15_ESM.zip › TRiP/input/C010143.jpg]

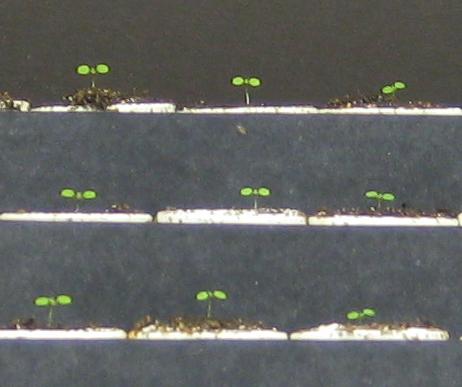

Supplement: Additional file 15 — TRiP. Compressed folder containing the TRiP code including a ReadMe file and sample image data. [file 13007_2015_75_MOESM15_ESM.zip › TRiP/input/C010144.jpg]

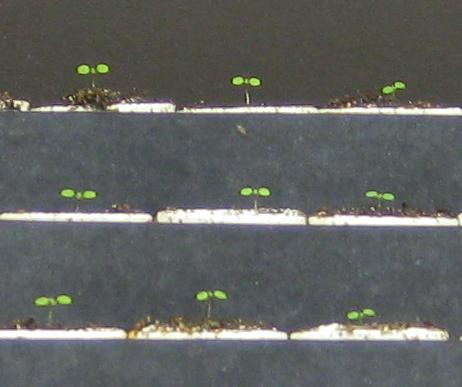

Supplement: Additional file 15 — TRiP. Compressed folder containing the TRiP code including a ReadMe file and sample image data. [file 13007_2015_75_MOESM15_ESM.zip › TRiP/input/C010145.jpg]

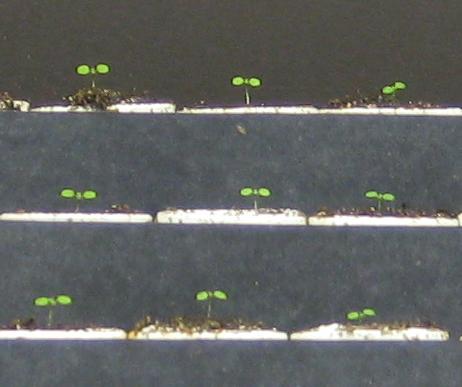

Supplement: Additional file 15 — TRiP. Compressed folder containing the TRiP code including a ReadMe file and sample image data. [file 13007_2015_75_MOESM15_ESM.zip › TRiP/input/C010146.jpg]

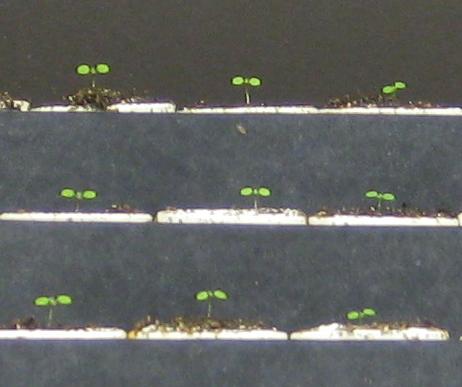

Supplement: Additional file 15 — TRiP. Compressed folder containing the TRiP code including a ReadMe file and sample image data. [file 13007_2015_75_MOESM15_ESM.zip › TRiP/input/C010147.jpg]

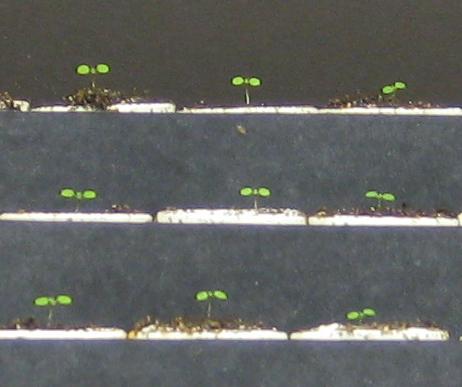

Supplement: Additional file 15 — TRiP. Compressed folder containing the TRiP code including a ReadMe file and sample image data. [file 13007_2015_75_MOESM15_ESM.zip › TRiP/input/C010148.jpg]

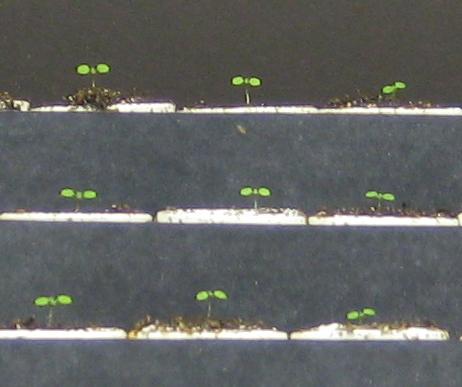

Supplement: Additional file 15 — TRiP. Compressed folder containing the TRiP code including a ReadMe file and sample image data. [file 13007_2015_75_MOESM15_ESM.zip › TRiP/input/C010149.jpg]

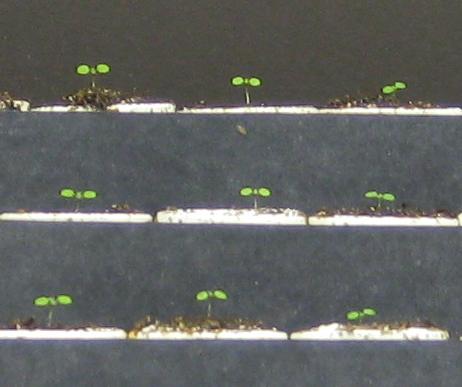

Supplement: Additional file 15 — TRiP. Compressed folder containing the TRiP code including a ReadMe file and sample image data. [file 13007_2015_75_MOESM15_ESM.zip › TRiP/input/C010150.jpg]

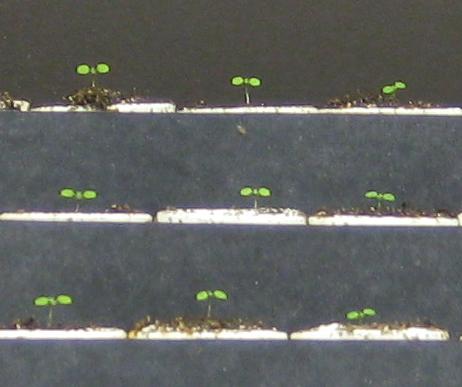

Supplement: Additional file 15 — TRiP. Compressed folder containing the TRiP code including a ReadMe file and sample image data. [file 13007_2015_75_MOESM15_ESM.zip › TRiP/input/C010151.jpg]

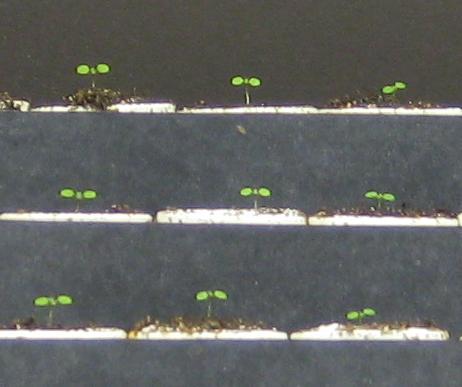

Supplement: Additional file 15 — TRiP. Compressed folder containing the TRiP code including a ReadMe file and sample image data. [file 13007_2015_75_MOESM15_ESM.zip › TRiP/input/C010152.jpg]

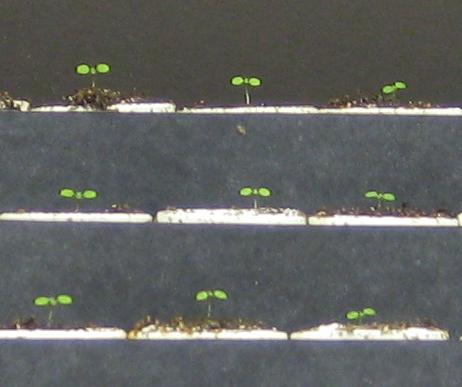

Supplement: Additional file 15 — TRiP. Compressed folder containing the TRiP code including a ReadMe file and sample image data. [file 13007_2015_75_MOESM15_ESM.zip › TRiP/input/C010153.jpg]

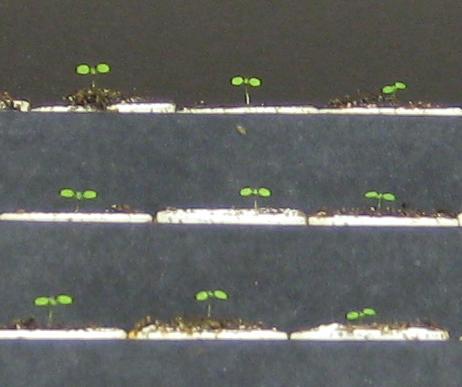

Supplement: Additional file 15 — TRiP. Compressed folder containing the TRiP code including a ReadMe file and sample image data. [file 13007_2015_75_MOESM15_ESM.zip › TRiP/input/C010154.jpg]

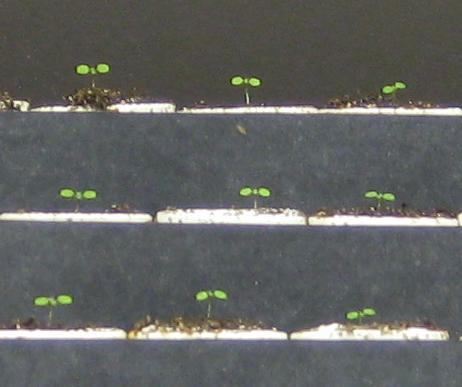

Supplement: Additional file 15 — TRiP. Compressed folder containing the TRiP code including a ReadMe file and sample image data. [file 13007_2015_75_MOESM15_ESM.zip › TRiP/input/C010155.jpg]

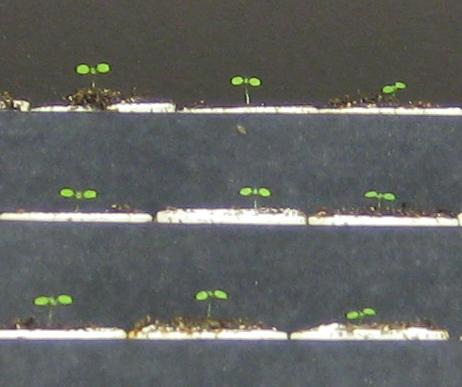

Supplement: Additional file 15 — TRiP. Compressed folder containing the TRiP code including a ReadMe file and sample image data. [file 13007_2015_75_MOESM15_ESM.zip › TRiP/input/C010156.jpg]

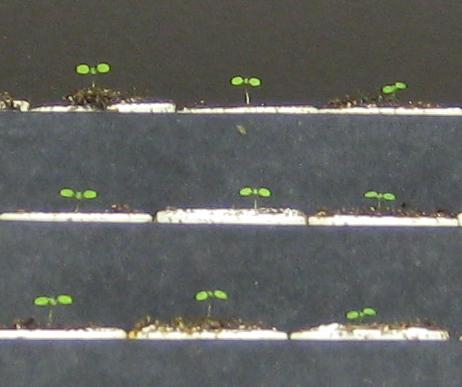

Supplement: Additional file 15 — TRiP. Compressed folder containing the TRiP code including a ReadMe file and sample image data. [file 13007_2015_75_MOESM15_ESM.zip › TRiP/input/C010157.jpg]

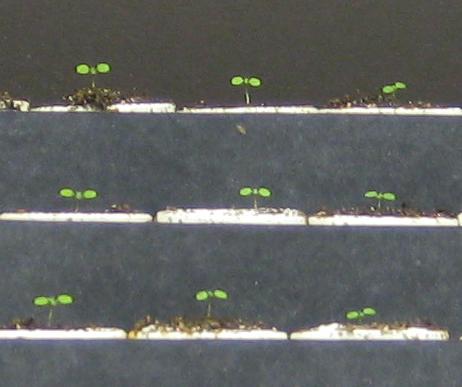

Supplement: Additional file 15 — TRiP. Compressed folder containing the TRiP code including a ReadMe file and sample image data. [file 13007_2015_75_MOESM15_ESM.zip › TRiP/input/C010158.jpg]

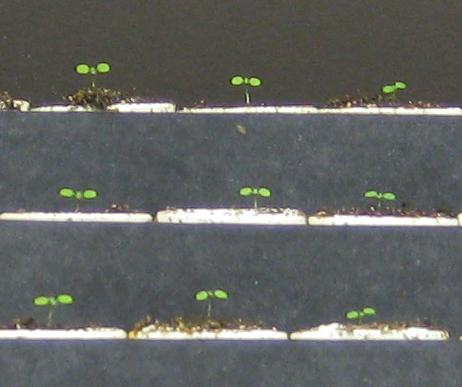

Supplement: Additional file 15 — TRiP. Compressed folder containing the TRiP code including a ReadMe file and sample image data. [file 13007_2015_75_MOESM15_ESM.zip › TRiP/input/C010159.jpg]

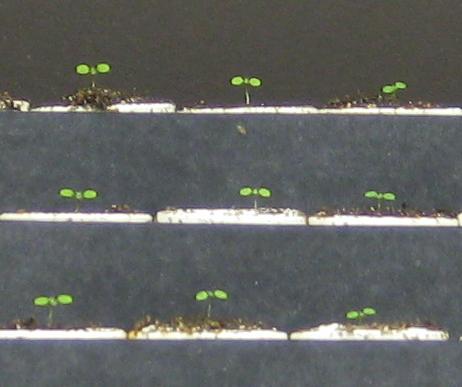

Supplement: Additional file 15 — TRiP. Compressed folder containing the TRiP code including a ReadMe file and sample image data. [file 13007_2015_75_MOESM15_ESM.zip › TRiP/input/C010160.jpg]

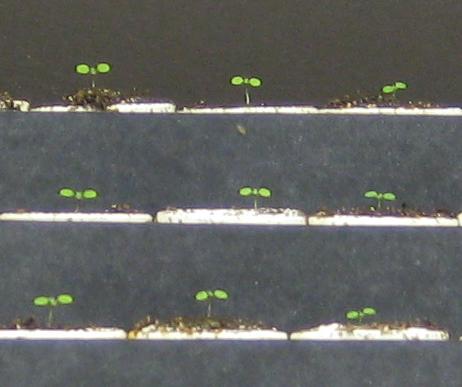

Supplement: Additional file 15 — TRiP. Compressed folder containing the TRiP code including a ReadMe file and sample image data. [file 13007_2015_75_MOESM15_ESM.zip › TRiP/input/C010161.jpg]

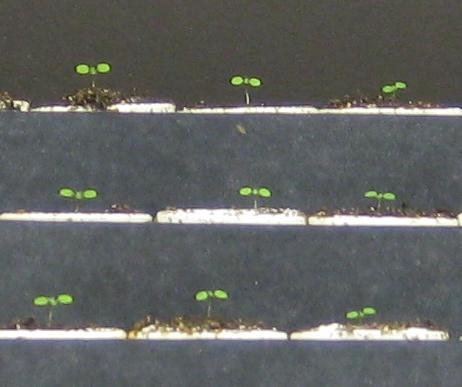

Supplement: Additional file 15 — TRiP. Compressed folder containing the TRiP code including a ReadMe file and sample image data. [file 13007_2015_75_MOESM15_ESM.zip › TRiP/input/C010162.jpg]

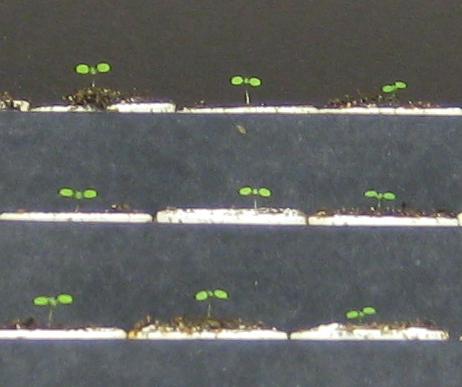

Supplement: Additional file 15 — TRiP. Compressed folder containing the TRiP code including a ReadMe file and sample image data. [file 13007_2015_75_MOESM15_ESM.zip › TRiP/input/C010163.jpg]

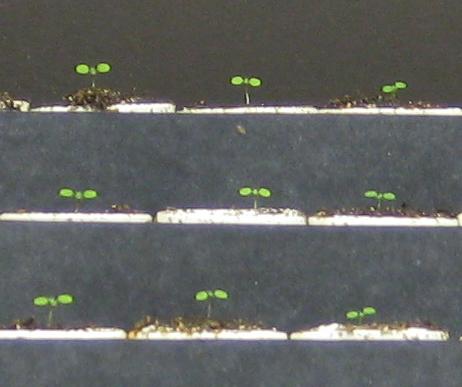

Supplement: Additional file 15 — TRiP. Compressed folder containing the TRiP code including a ReadMe file and sample image data. [file 13007_2015_75_MOESM15_ESM.zip › TRiP/input/C010164.jpg]

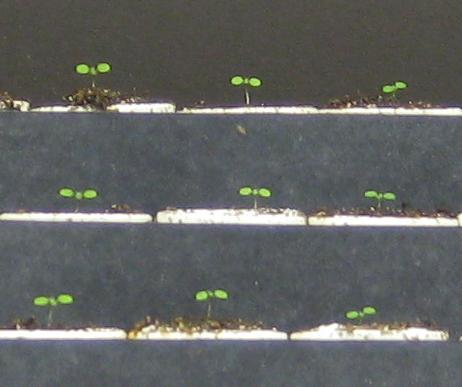

Supplement: Additional file 15 — TRiP. Compressed folder containing the TRiP code including a ReadMe file and sample image data. [file 13007_2015_75_MOESM15_ESM.zip › TRiP/input/C010165.jpg]

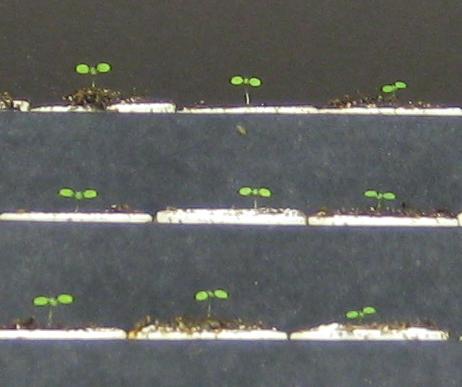

Supplement: Additional file 15 — TRiP. Compressed folder containing the TRiP code including a ReadMe file and sample image data. [file 13007_2015_75_MOESM15_ESM.zip › TRiP/input/C010166.jpg]

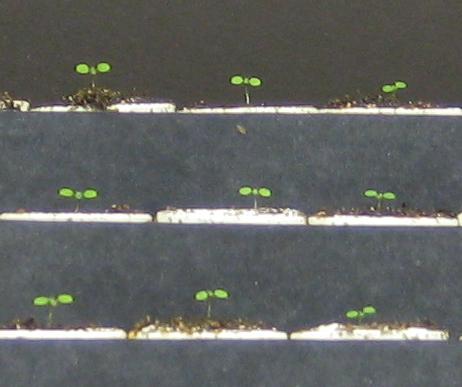

Supplement: Additional file 15 — TRiP. Compressed folder containing the TRiP code including a ReadMe file and sample image data. [file 13007_2015_75_MOESM15_ESM.zip › TRiP/input/C010167.jpg]

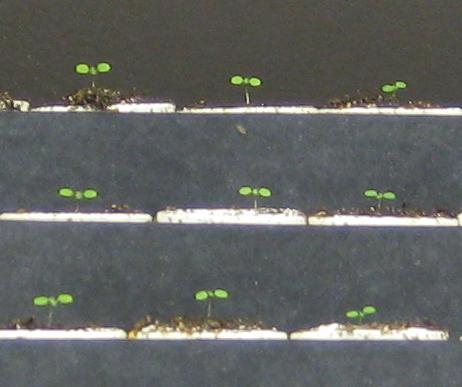

Supplement: Additional file 15 — TRiP. Compressed folder containing the TRiP code including a ReadMe file and sample image data. [file 13007_2015_75_MOESM15_ESM.zip › TRiP/input/C010168.jpg]

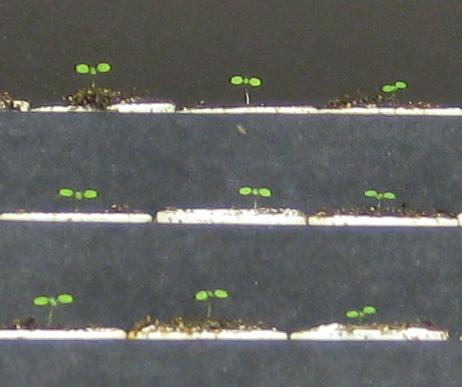

Supplement: Additional file 15 — TRiP. Compressed folder containing the TRiP code including a ReadMe file and sample image data. [file 13007_2015_75_MOESM15_ESM.zip › TRiP/input/C010169.jpg]

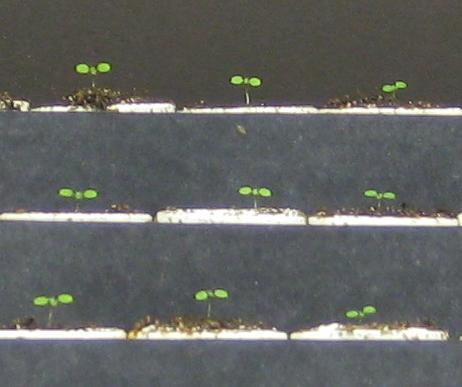

Supplement: Additional file 15 — TRiP. Compressed folder containing the TRiP code including a ReadMe file and sample image data. [file 13007_2015_75_MOESM15_ESM.zip › TRiP/input/C010170.jpg]

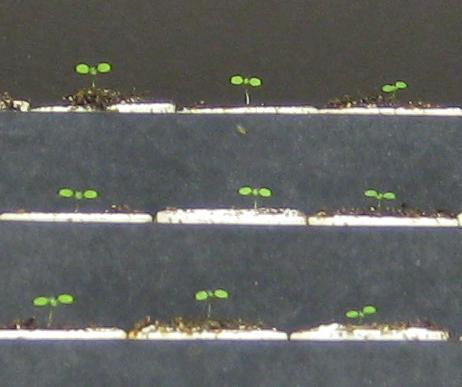

Supplement: Additional file 15 — TRiP. Compressed folder containing the TRiP code including a ReadMe file and sample image data. [file 13007_2015_75_MOESM15_ESM.zip › TRiP/input/C010171.jpg]

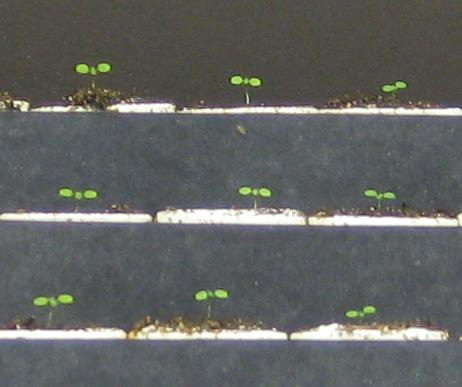

Supplement: Additional file 15 — TRiP. Compressed folder containing the TRiP code including a ReadMe file and sample image data. [file 13007_2015_75_MOESM15_ESM.zip › TRiP/input/C010172.jpg]

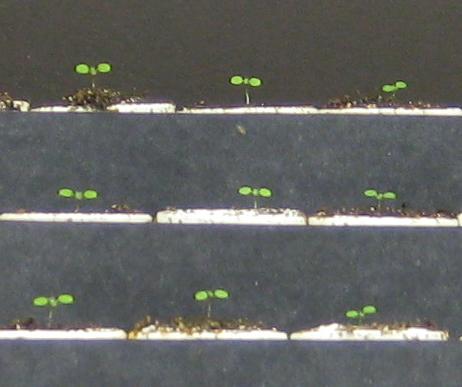

Supplement: Additional file 15 — TRiP. Compressed folder containing the TRiP code including a ReadMe file and sample image data. [file 13007_2015_75_MOESM15_ESM.zip › TRiP/input/C010173.jpg]

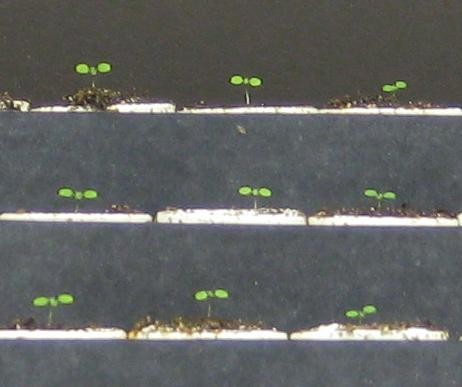

Supplement: Additional file 15 — TRiP. Compressed folder containing the TRiP code including a ReadMe file and sample image data. [file 13007_2015_75_MOESM15_ESM.zip › TRiP/input/C010174.jpg]

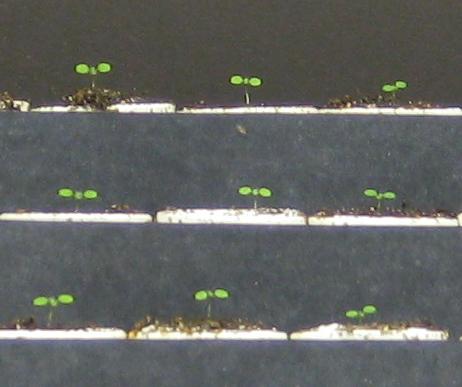

Supplement: Additional file 15 — TRiP. Compressed folder containing the TRiP code including a ReadMe file and sample image data. [file 13007_2015_75_MOESM15_ESM.zip › TRiP/input/C010175.jpg]

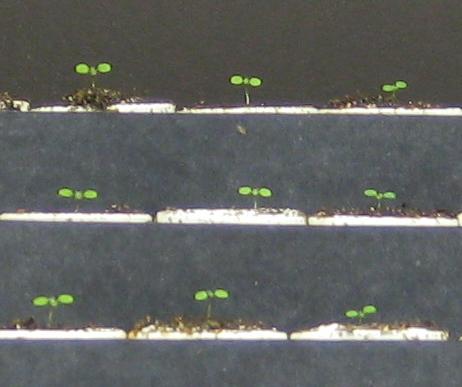

Supplement: Additional file 15 — TRiP. Compressed folder containing the TRiP code including a ReadMe file and sample image data. [file 13007_2015_75_MOESM15_ESM.zip › TRiP/input/C010176.jpg]

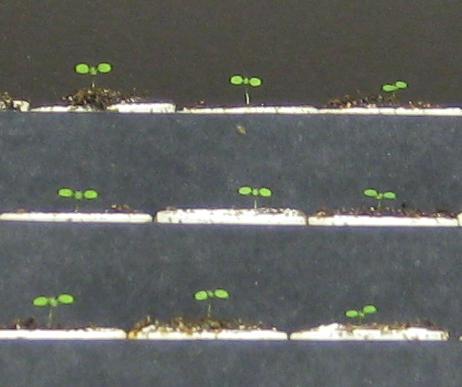

Supplement: Additional file 15 — TRiP. Compressed folder containing the TRiP code including a ReadMe file and sample image data. [file 13007_2015_75_MOESM15_ESM.zip › TRiP/input/C010177.jpg]

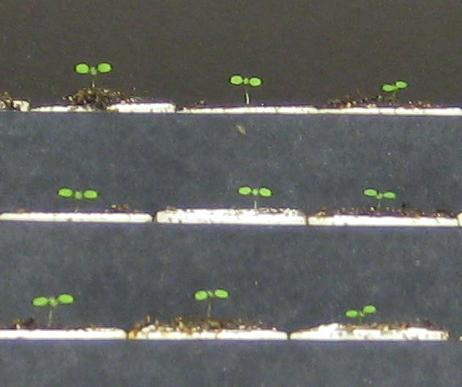

Supplement: Additional file 15 — TRiP. Compressed folder containing the TRiP code including a ReadMe file and sample image data. [file 13007_2015_75_MOESM15_ESM.zip › TRiP/input/C010178.jpg]

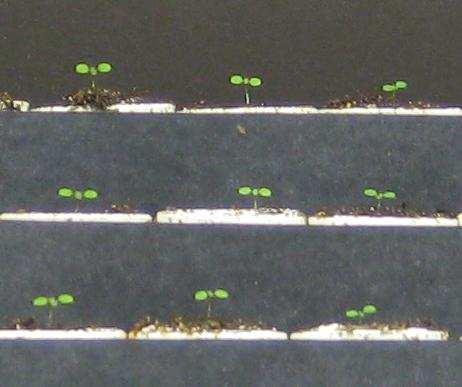

Supplement: Additional file 15 — TRiP. Compressed folder containing the TRiP code including a ReadMe file and sample image data. [file 13007_2015_75_MOESM15_ESM.zip › TRiP/input/C010179.jpg]

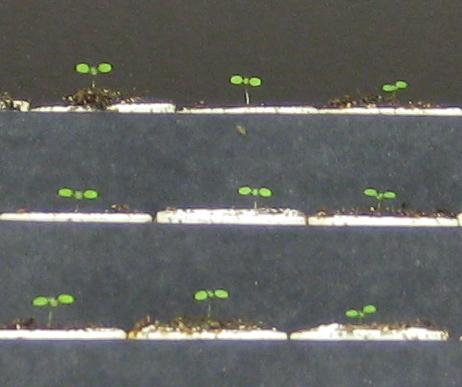

Supplement: Additional file 15 — TRiP. Compressed folder containing the TRiP code including a ReadMe file and sample image data. [file 13007_2015_75_MOESM15_ESM.zip › TRiP/input/C010180.jpg]

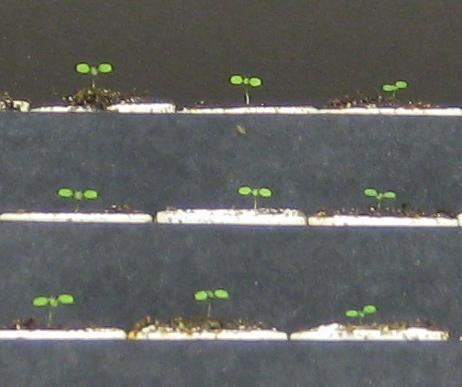

Supplement: Additional file 15 — TRiP. Compressed folder containing the TRiP code including a ReadMe file and sample image data. [file 13007_2015_75_MOESM15_ESM.zip › TRiP/input/C010181.jpg]

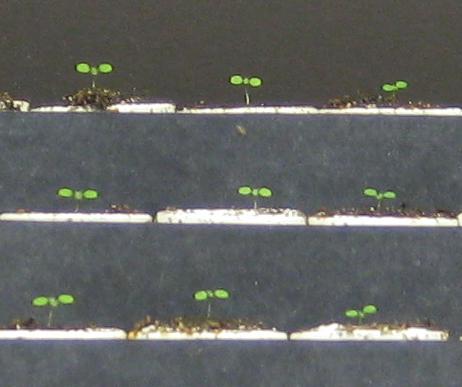

Supplement: Additional file 15 — TRiP. Compressed folder containing the TRiP code including a ReadMe file and sample image data. [file 13007_2015_75_MOESM15_ESM.zip › TRiP/input/C010182.jpg]

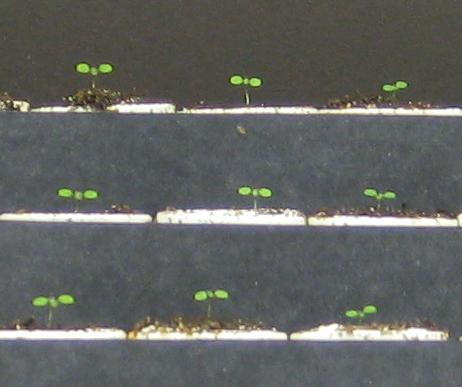

Supplement: Additional file 15 — TRiP. Compressed folder containing the TRiP code including a ReadMe file and sample image data. [file 13007_2015_75_MOESM15_ESM.zip › TRiP/input/C010183.jpg]

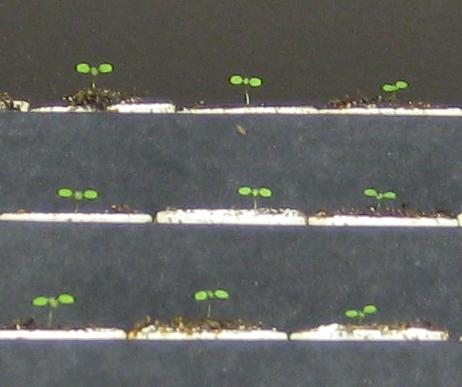

Supplement: Additional file 15 — TRiP. Compressed folder containing the TRiP code including a ReadMe file and sample image data. [file 13007_2015_75_MOESM15_ESM.zip › TRiP/input/C010184.jpg]

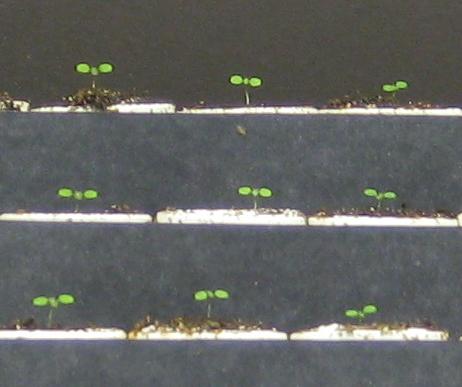

Supplement: Additional file 15 — TRiP. Compressed folder containing the TRiP code including a ReadMe file and sample image data. [file 13007_2015_75_MOESM15_ESM.zip › TRiP/input/C010185.jpg]

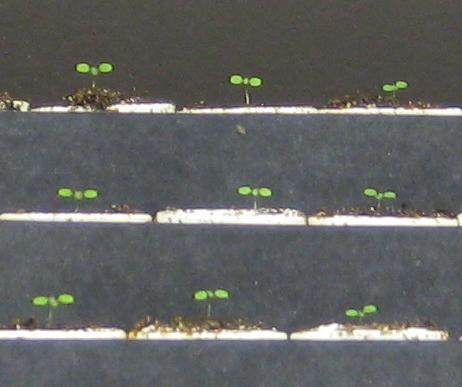

Supplement: Additional file 15 — TRiP. Compressed folder containing the TRiP code including a ReadMe file and sample image data. [file 13007_2015_75_MOESM15_ESM.zip › TRiP/input/C010186.jpg]

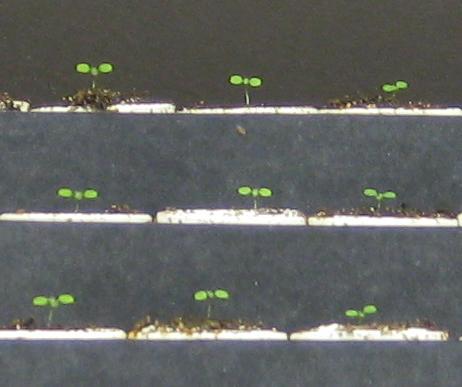

Supplement: Additional file 15 — TRiP. Compressed folder containing the TRiP code including a ReadMe file and sample image data. [file 13007_2015_75_MOESM15_ESM.zip › TRiP/input/C010187.jpg]

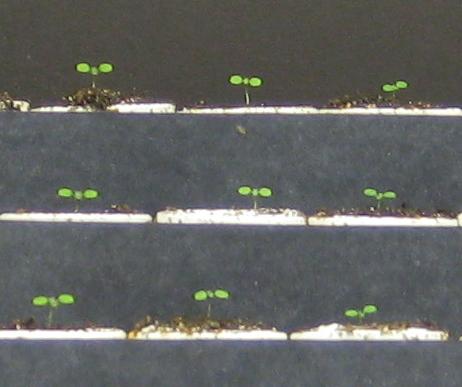

Supplement: Additional file 15 — TRiP. Compressed folder containing the TRiP code including a ReadMe file and sample image data. [file 13007_2015_75_MOESM15_ESM.zip › TRiP/input/C010188.jpg]

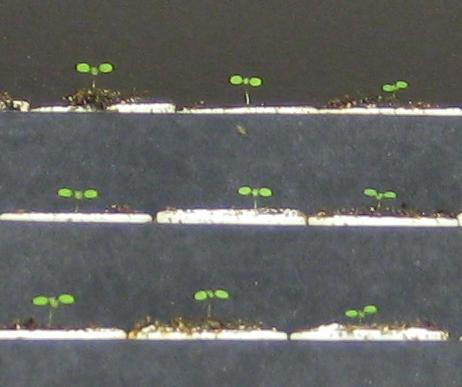

Supplement: Additional file 15 — TRiP. Compressed folder containing the TRiP code including a ReadMe file and sample image data. [file 13007_2015_75_MOESM15_ESM.zip › TRiP/input/C010189.jpg]

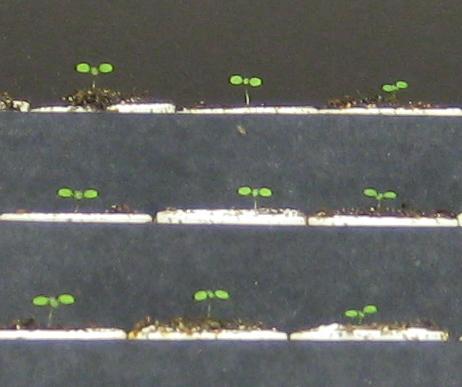

Supplement: Additional file 15 — TRiP. Compressed folder containing the TRiP code including a ReadMe file and sample image data. [file 13007_2015_75_MOESM15_ESM.zip › TRiP/input/C010190.jpg]

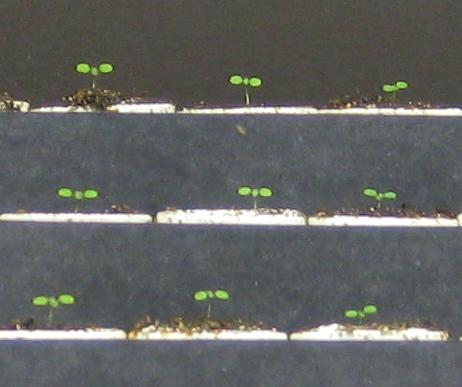

Supplement: Additional file 15 — TRiP. Compressed folder containing the TRiP code including a ReadMe file and sample image data. [file 13007_2015_75_MOESM15_ESM.zip › TRiP/input/C010191.jpg]

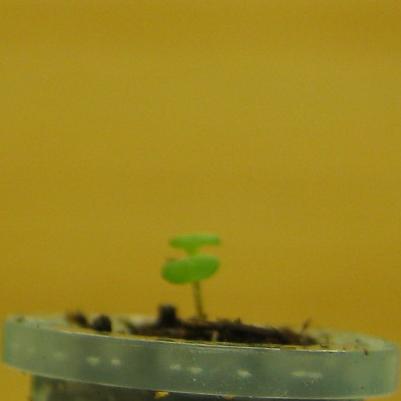

Supplement: Additional file 19 — Col-0 Side View Images for 3-D Model. Images of Col-0 captured every 10 min for 5 days from the side view for the 3-D CG model. Table S2 lists the images used as key frames in the model. [file 13007_2015_75_MOESM19_ESM.zip › side_view/side3_0001.jpg]

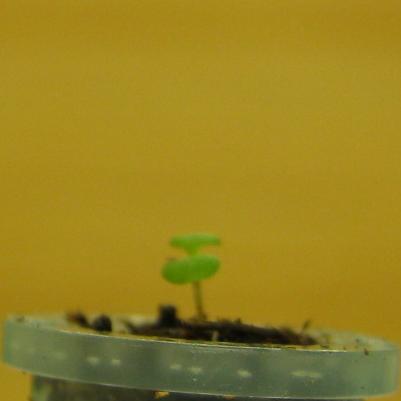

Supplement: Additional file 19 — Col-0 Side View Images for 3-D Model. Images of Col-0 captured every 10 min for 5 days from the side view for the 3-D CG model. Table S2 lists the images used as key frames in the model. [file 13007_2015_75_MOESM19_ESM.zip › side_view/side3_0002.jpg]

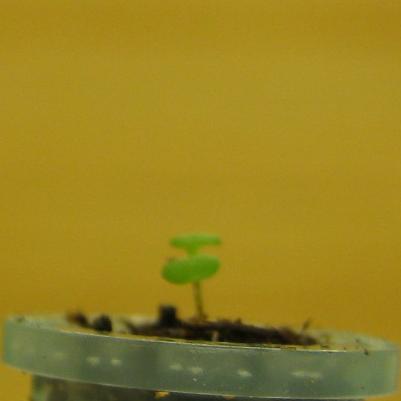

Supplement: Additional file 19 — Col-0 Side View Images for 3-D Model. Images of Col-0 captured every 10 min for 5 days from the side view for the 3-D CG model. Table S2 lists the images used as key frames in the model. [file 13007_2015_75_MOESM19_ESM.zip › side_view/side3_0003.jpg]

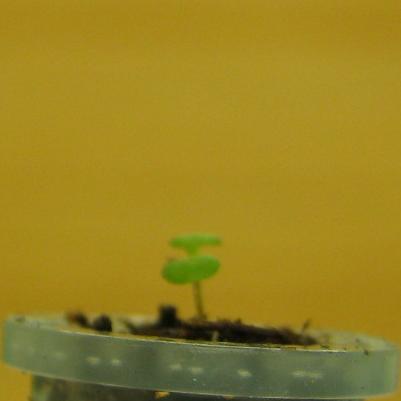

Supplement: Additional file 19 — Col-0 Side View Images for 3-D Model. Images of Col-0 captured every 10 min for 5 days from the side view for the 3-D CG model. Table S2 lists the images used as key frames in the model. [file 13007_2015_75_MOESM19_ESM.zip › side_view/side3_0004.jpg]

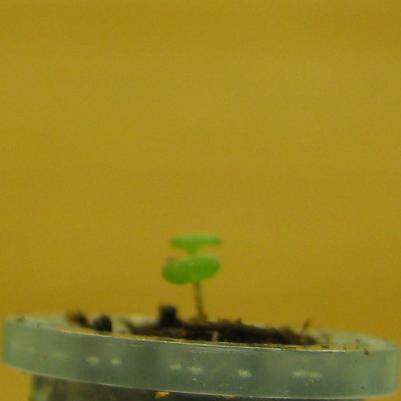

Supplement: Additional file 19 — Col-0 Side View Images for 3-D Model. Images of Col-0 captured every 10 min for 5 days from the side view for the 3-D CG model. Table S2 lists the images used as key frames in the model. [file 13007_2015_75_MOESM19_ESM.zip › side_view/side3_0005.jpg]

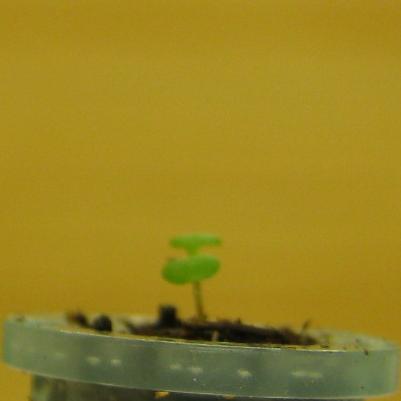

Supplement: Additional file 19 — Col-0 Side View Images for 3-D Model. Images of Col-0 captured every 10 min for 5 days from the side view for the 3-D CG model. Table S2 lists the images used as key frames in the model. [file 13007_2015_75_MOESM19_ESM.zip › side_view/side3_0006.jpg]

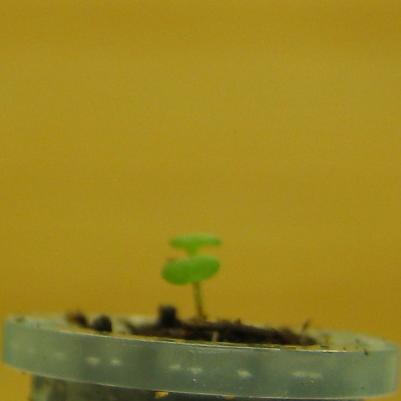

Supplement: Additional file 19 — Col-0 Side View Images for 3-D Model. Images of Col-0 captured every 10 min for 5 days from the side view for the 3-D CG model. Table S2 lists the images used as key frames in the model. [file 13007_2015_75_MOESM19_ESM.zip › side_view/side3_0007.jpg]

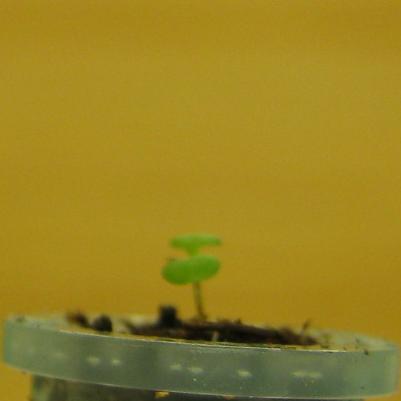

Supplement: Additional file 19 — Col-0 Side View Images for 3-D Model. Images of Col-0 captured every 10 min for 5 days from the side view for the 3-D CG model. Table S2 lists the images used as key frames in the model. [file 13007_2015_75_MOESM19_ESM.zip › side_view/side3_0008.jpg]
