# Supplementary figures and images for: TRiP: Tracking Rhythms in Plants, an automated leaf movement analysis program for circadian period estimation (part 4 of 10)
Source: Plant Methods. 2015 May 3;11:33. doi: 10.1186/s13007-015-0075-5 (PMC4445800; doi:10.1186/s13007-015-0075-5)

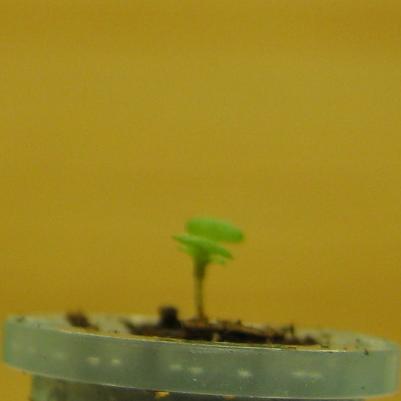

Supplement: Additional file 19 — Col-0 Side View Images for 3-D Model. Images of Col-0 captured every 10 min for 5 days from the side view for the 3-D CG model. Table S2 lists the images used as key frames in the model. [file 13007_2015_75_MOESM19_ESM.zip › side_view/side3_0109.jpg]

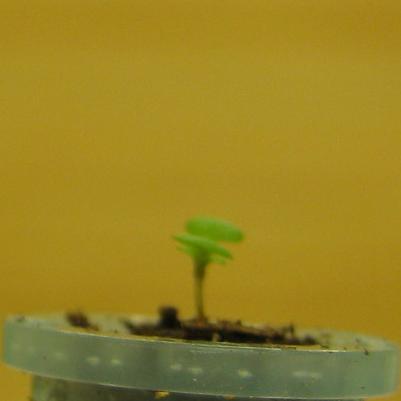

Supplement: Additional file 19 — Col-0 Side View Images for 3-D Model. Images of Col-0 captured every 10 min for 5 days from the side view for the 3-D CG model. Table S2 lists the images used as key frames in the model. [file 13007_2015_75_MOESM19_ESM.zip › side_view/side3_0110.jpg]

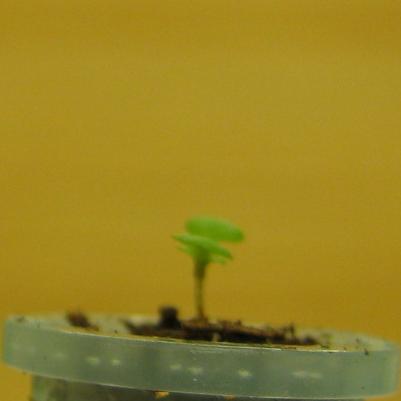

Supplement: Additional file 19 — Col-0 Side View Images for 3-D Model. Images of Col-0 captured every 10 min for 5 days from the side view for the 3-D CG model. Table S2 lists the images used as key frames in the model. [file 13007_2015_75_MOESM19_ESM.zip › side_view/side3_0111.jpg]

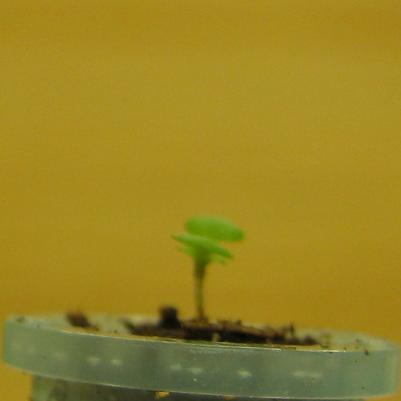

Supplement: Additional file 19 — Col-0 Side View Images for 3-D Model. Images of Col-0 captured every 10 min for 5 days from the side view for the 3-D CG model. Table S2 lists the images used as key frames in the model. [file 13007_2015_75_MOESM19_ESM.zip › side_view/side3_0112.jpg]

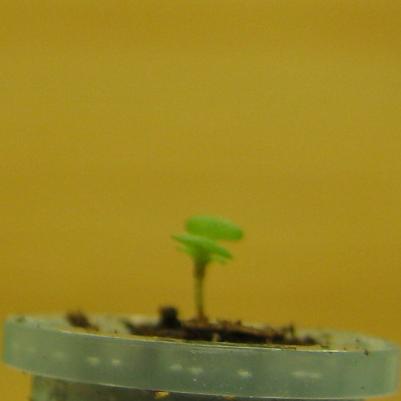

Supplement: Additional file 19 — Col-0 Side View Images for 3-D Model. Images of Col-0 captured every 10 min for 5 days from the side view for the 3-D CG model. Table S2 lists the images used as key frames in the model. [file 13007_2015_75_MOESM19_ESM.zip › side_view/side3_0113.jpg]

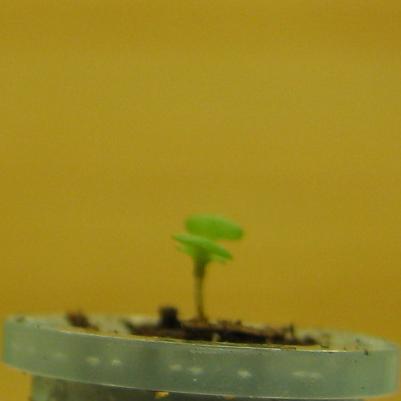

Supplement: Additional file 19 — Col-0 Side View Images for 3-D Model. Images of Col-0 captured every 10 min for 5 days from the side view for the 3-D CG model. Table S2 lists the images used as key frames in the model. [file 13007_2015_75_MOESM19_ESM.zip › side_view/side3_0114.jpg]

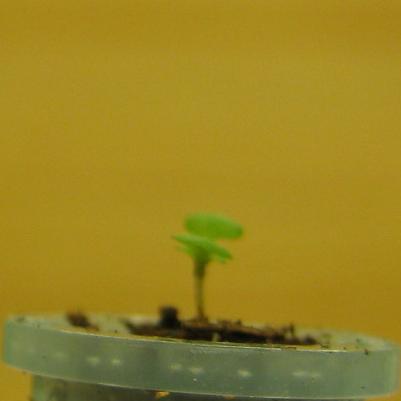

Supplement: Additional file 19 — Col-0 Side View Images for 3-D Model. Images of Col-0 captured every 10 min for 5 days from the side view for the 3-D CG model. Table S2 lists the images used as key frames in the model. [file 13007_2015_75_MOESM19_ESM.zip › side_view/side3_0115.jpg]

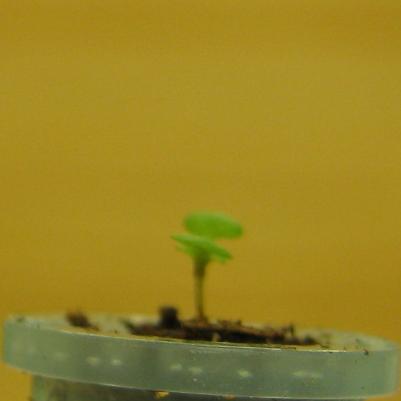

Supplement: Additional file 19 — Col-0 Side View Images for 3-D Model. Images of Col-0 captured every 10 min for 5 days from the side view for the 3-D CG model. Table S2 lists the images used as key frames in the model. [file 13007_2015_75_MOESM19_ESM.zip › side_view/side3_0116.jpg]

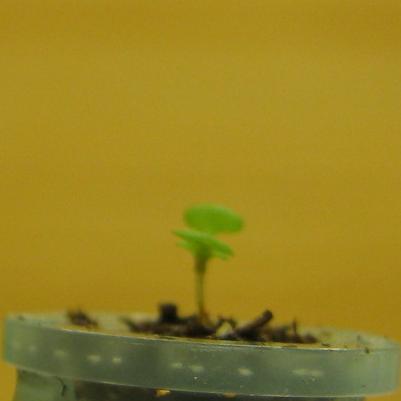

Supplement: Additional file 19 — Col-0 Side View Images for 3-D Model. Images of Col-0 captured every 10 min for 5 days from the side view for the 3-D CG model. Table S2 lists the images used as key frames in the model. [file 13007_2015_75_MOESM19_ESM.zip › side_view/side3_0117.jpg]

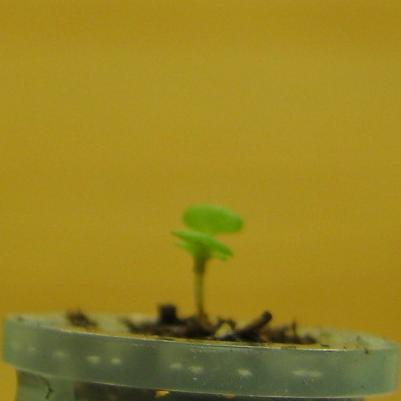

Supplement: Additional file 19 — Col-0 Side View Images for 3-D Model. Images of Col-0 captured every 10 min for 5 days from the side view for the 3-D CG model. Table S2 lists the images used as key frames in the model. [file 13007_2015_75_MOESM19_ESM.zip › side_view/side3_0118.jpg]

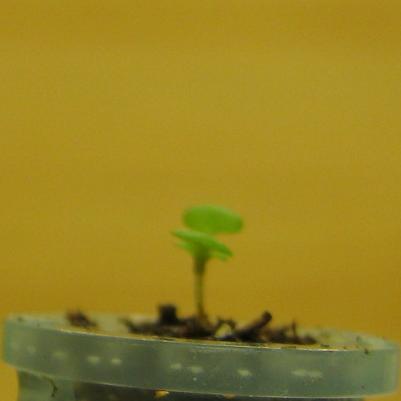

Supplement: Additional file 19 — Col-0 Side View Images for 3-D Model. Images of Col-0 captured every 10 min for 5 days from the side view for the 3-D CG model. Table S2 lists the images used as key frames in the model. [file 13007_2015_75_MOESM19_ESM.zip › side_view/side3_0119.jpg]

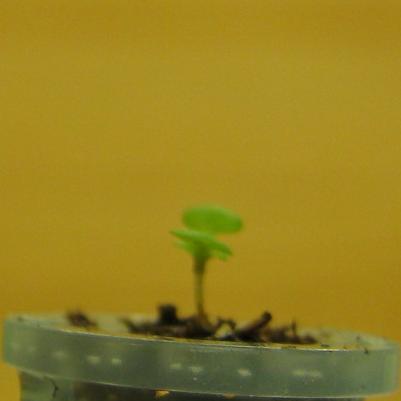

Supplement: Additional file 19 — Col-0 Side View Images for 3-D Model. Images of Col-0 captured every 10 min for 5 days from the side view for the 3-D CG model. Table S2 lists the images used as key frames in the model. [file 13007_2015_75_MOESM19_ESM.zip › side_view/side3_0120.jpg]

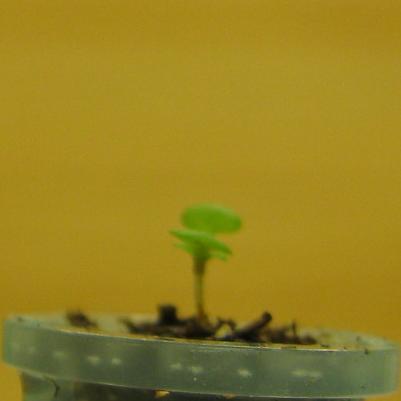

Supplement: Additional file 19 — Col-0 Side View Images for 3-D Model. Images of Col-0 captured every 10 min for 5 days from the side view for the 3-D CG model. Table S2 lists the images used as key frames in the model. [file 13007_2015_75_MOESM19_ESM.zip › side_view/side3_0121.jpg]

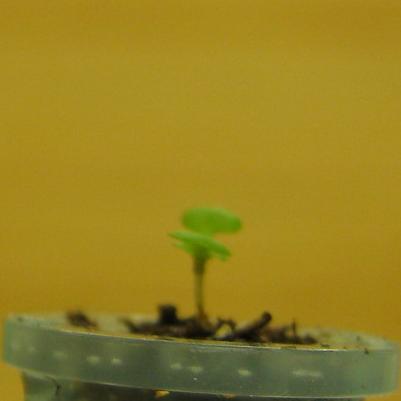

Supplement: Additional file 19 — Col-0 Side View Images for 3-D Model. Images of Col-0 captured every 10 min for 5 days from the side view for the 3-D CG model. Table S2 lists the images used as key frames in the model. [file 13007_2015_75_MOESM19_ESM.zip › side_view/side3_0122.jpg]

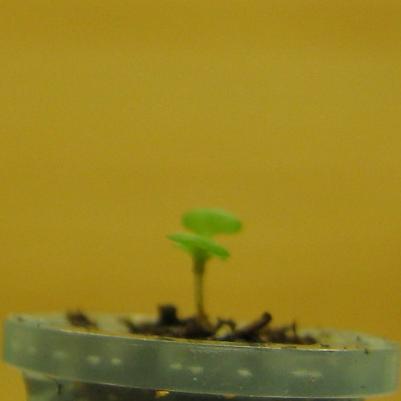

Supplement: Additional file 19 — Col-0 Side View Images for 3-D Model. Images of Col-0 captured every 10 min for 5 days from the side view for the 3-D CG model. Table S2 lists the images used as key frames in the model. [file 13007_2015_75_MOESM19_ESM.zip › side_view/side3_0123.jpg]

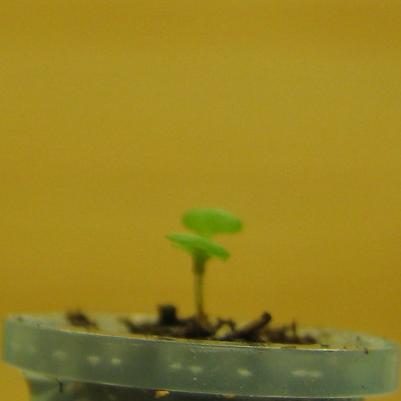

Supplement: Additional file 19 — Col-0 Side View Images for 3-D Model. Images of Col-0 captured every 10 min for 5 days from the side view for the 3-D CG model. Table S2 lists the images used as key frames in the model. [file 13007_2015_75_MOESM19_ESM.zip › side_view/side3_0124.jpg]

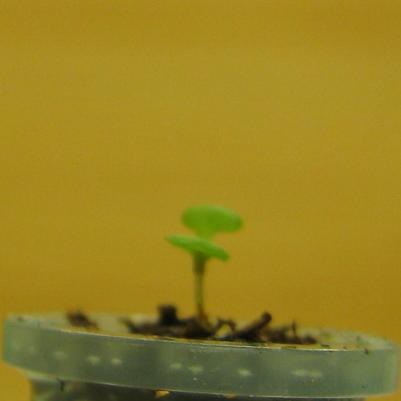

Supplement: Additional file 19 — Col-0 Side View Images for 3-D Model. Images of Col-0 captured every 10 min for 5 days from the side view for the 3-D CG model. Table S2 lists the images used as key frames in the model. [file 13007_2015_75_MOESM19_ESM.zip › side_view/side3_0125.jpg]

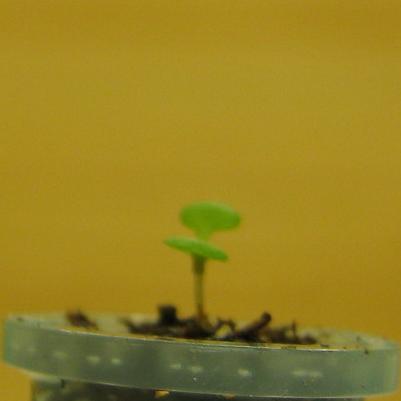

Supplement: Additional file 19 — Col-0 Side View Images for 3-D Model. Images of Col-0 captured every 10 min for 5 days from the side view for the 3-D CG model. Table S2 lists the images used as key frames in the model. [file 13007_2015_75_MOESM19_ESM.zip › side_view/side3_0126.jpg]

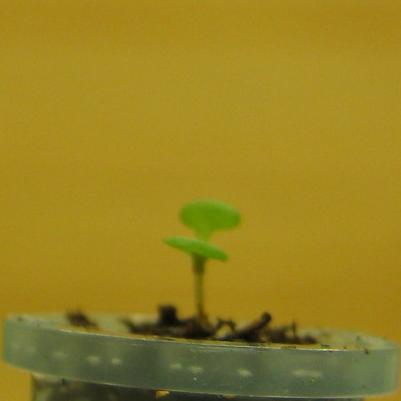

Supplement: Additional file 19 — Col-0 Side View Images for 3-D Model. Images of Col-0 captured every 10 min for 5 days from the side view for the 3-D CG model. Table S2 lists the images used as key frames in the model. [file 13007_2015_75_MOESM19_ESM.zip › side_view/side3_0127.jpg]

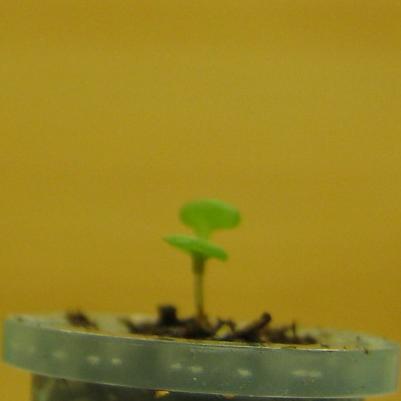

Supplement: Additional file 19 — Col-0 Side View Images for 3-D Model. Images of Col-0 captured every 10 min for 5 days from the side view for the 3-D CG model. Table S2 lists the images used as key frames in the model. [file 13007_2015_75_MOESM19_ESM.zip › side_view/side3_0128.jpg]

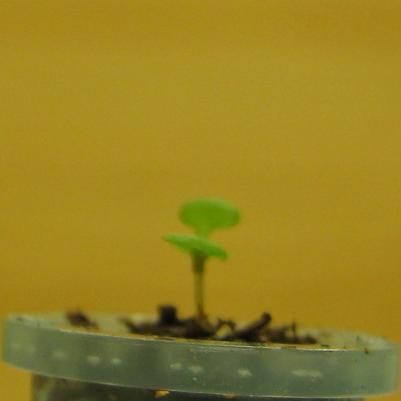

Supplement: Additional file 19 — Col-0 Side View Images for 3-D Model. Images of Col-0 captured every 10 min for 5 days from the side view for the 3-D CG model. Table S2 lists the images used as key frames in the model. [file 13007_2015_75_MOESM19_ESM.zip › side_view/side3_0129.jpg]

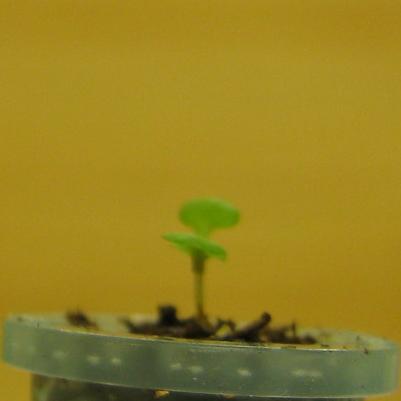

Supplement: Additional file 19 — Col-0 Side View Images for 3-D Model. Images of Col-0 captured every 10 min for 5 days from the side view for the 3-D CG model. Table S2 lists the images used as key frames in the model. [file 13007_2015_75_MOESM19_ESM.zip › side_view/side3_0130.jpg]

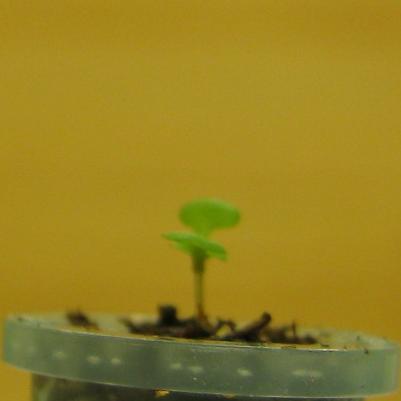

Supplement: Additional file 19 — Col-0 Side View Images for 3-D Model. Images of Col-0 captured every 10 min for 5 days from the side view for the 3-D CG model. Table S2 lists the images used as key frames in the model. [file 13007_2015_75_MOESM19_ESM.zip › side_view/side3_0131.jpg]

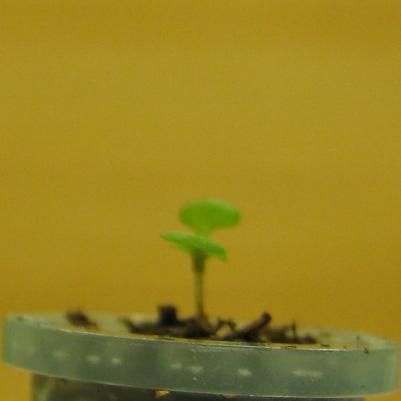

Supplement: Additional file 19 — Col-0 Side View Images for 3-D Model. Images of Col-0 captured every 10 min for 5 days from the side view for the 3-D CG model. Table S2 lists the images used as key frames in the model. [file 13007_2015_75_MOESM19_ESM.zip › side_view/side3_0132.jpg]

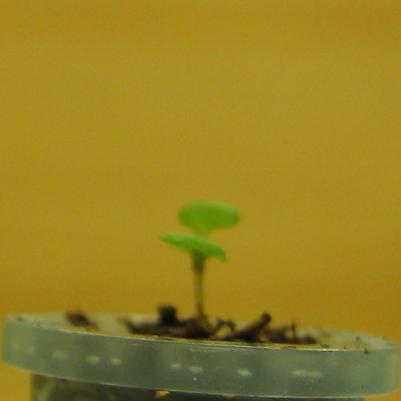

Supplement: Additional file 19 — Col-0 Side View Images for 3-D Model. Images of Col-0 captured every 10 min for 5 days from the side view for the 3-D CG model. Table S2 lists the images used as key frames in the model. [file 13007_2015_75_MOESM19_ESM.zip › side_view/side3_0133.jpg]

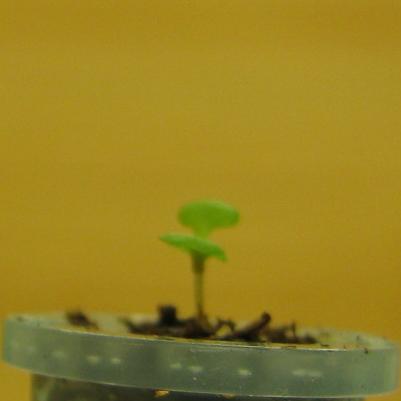

Supplement: Additional file 19 — Col-0 Side View Images for 3-D Model. Images of Col-0 captured every 10 min for 5 days from the side view for the 3-D CG model. Table S2 lists the images used as key frames in the model. [file 13007_2015_75_MOESM19_ESM.zip › side_view/side3_0134.jpg]

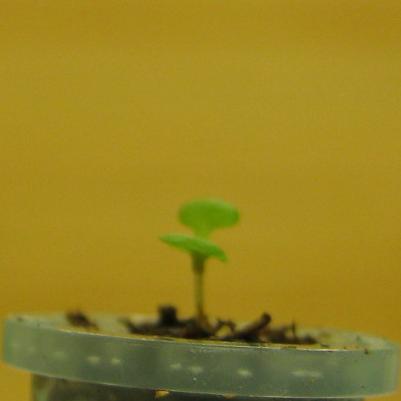

Supplement: Additional file 19 — Col-0 Side View Images for 3-D Model. Images of Col-0 captured every 10 min for 5 days from the side view for the 3-D CG model. Table S2 lists the images used as key frames in the model. [file 13007_2015_75_MOESM19_ESM.zip › side_view/side3_0135.jpg]

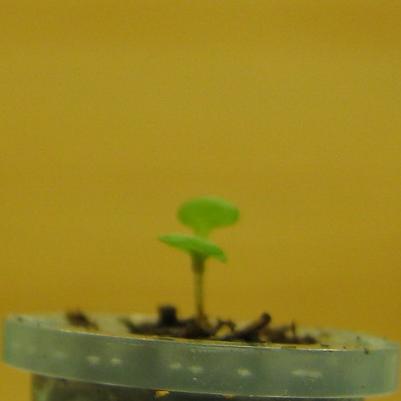

Supplement: Additional file 19 — Col-0 Side View Images for 3-D Model. Images of Col-0 captured every 10 min for 5 days from the side view for the 3-D CG model. Table S2 lists the images used as key frames in the model. [file 13007_2015_75_MOESM19_ESM.zip › side_view/side3_0136.jpg]

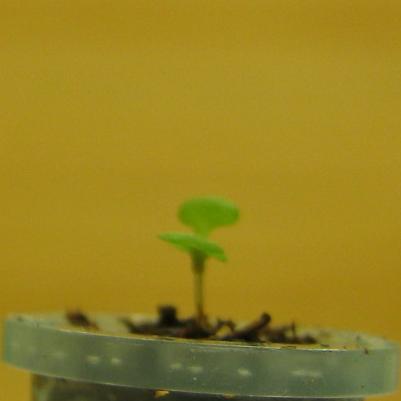

Supplement: Additional file 19 — Col-0 Side View Images for 3-D Model. Images of Col-0 captured every 10 min for 5 days from the side view for the 3-D CG model. Table S2 lists the images used as key frames in the model. [file 13007_2015_75_MOESM19_ESM.zip › side_view/side3_0137.jpg]

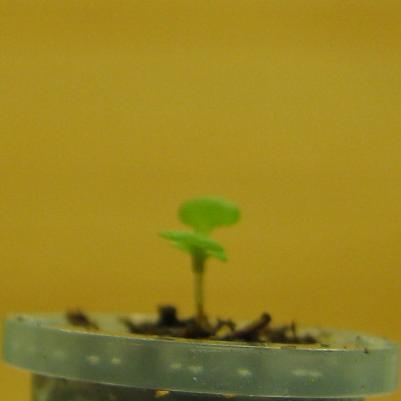

Supplement: Additional file 19 — Col-0 Side View Images for 3-D Model. Images of Col-0 captured every 10 min for 5 days from the side view for the 3-D CG model. Table S2 lists the images used as key frames in the model. [file 13007_2015_75_MOESM19_ESM.zip › side_view/side3_0138.jpg]

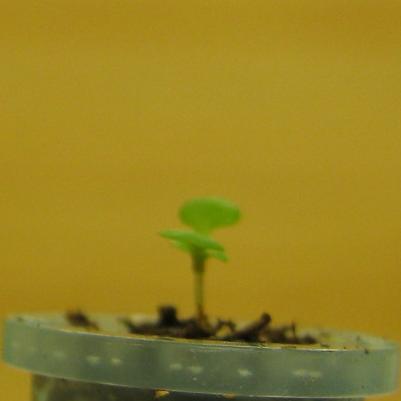

Supplement: Additional file 19 — Col-0 Side View Images for 3-D Model. Images of Col-0 captured every 10 min for 5 days from the side view for the 3-D CG model. Table S2 lists the images used as key frames in the model. [file 13007_2015_75_MOESM19_ESM.zip › side_view/side3_0139.jpg]

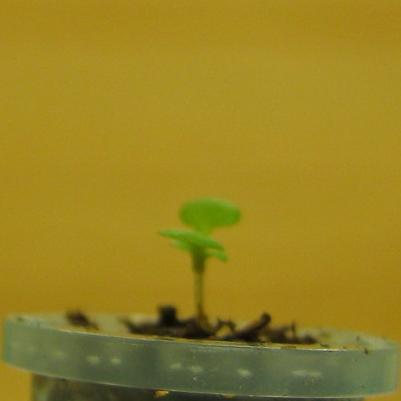

Supplement: Additional file 19 — Col-0 Side View Images for 3-D Model. Images of Col-0 captured every 10 min for 5 days from the side view for the 3-D CG model. Table S2 lists the images used as key frames in the model. [file 13007_2015_75_MOESM19_ESM.zip › side_view/side3_0140.jpg]

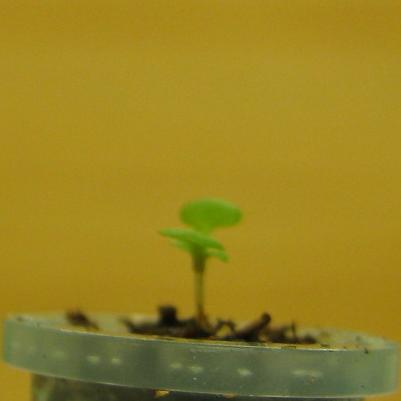

Supplement: Additional file 19 — Col-0 Side View Images for 3-D Model. Images of Col-0 captured every 10 min for 5 days from the side view for the 3-D CG model. Table S2 lists the images used as key frames in the model. [file 13007_2015_75_MOESM19_ESM.zip › side_view/side3_0141.jpg]

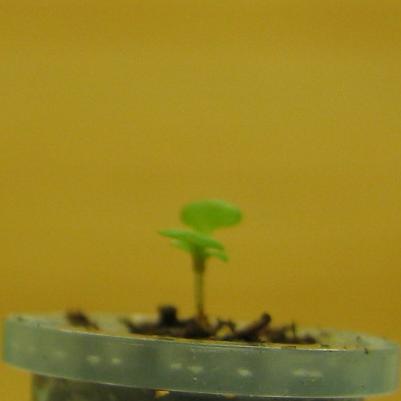

Supplement: Additional file 19 — Col-0 Side View Images for 3-D Model. Images of Col-0 captured every 10 min for 5 days from the side view for the 3-D CG model. Table S2 lists the images used as key frames in the model. [file 13007_2015_75_MOESM19_ESM.zip › side_view/side3_0142.jpg]

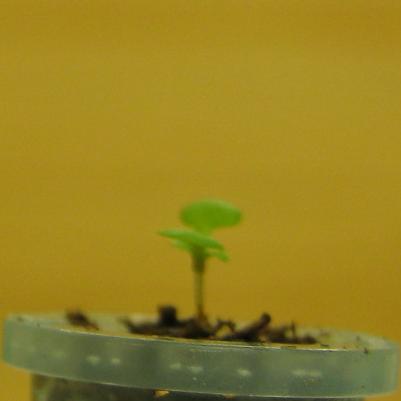

Supplement: Additional file 19 — Col-0 Side View Images for 3-D Model. Images of Col-0 captured every 10 min for 5 days from the side view for the 3-D CG model. Table S2 lists the images used as key frames in the model. [file 13007_2015_75_MOESM19_ESM.zip › side_view/side3_0143.jpg]

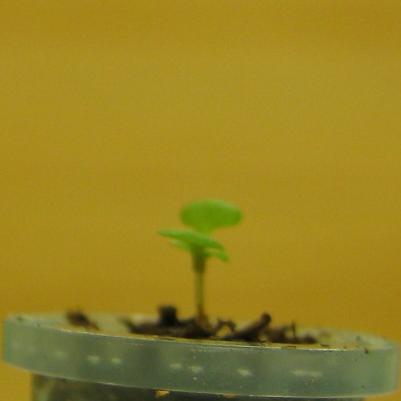

Supplement: Additional file 19 — Col-0 Side View Images for 3-D Model. Images of Col-0 captured every 10 min for 5 days from the side view for the 3-D CG model. Table S2 lists the images used as key frames in the model. [file 13007_2015_75_MOESM19_ESM.zip › side_view/side3_0144.jpg]

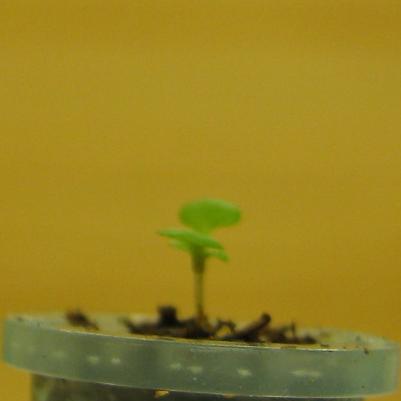

Supplement: Additional file 19 — Col-0 Side View Images for 3-D Model. Images of Col-0 captured every 10 min for 5 days from the side view for the 3-D CG model. Table S2 lists the images used as key frames in the model. [file 13007_2015_75_MOESM19_ESM.zip › side_view/side3_0145.jpg]

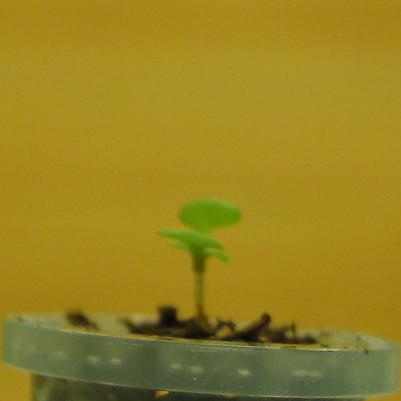

Supplement: Additional file 19 — Col-0 Side View Images for 3-D Model. Images of Col-0 captured every 10 min for 5 days from the side view for the 3-D CG model. Table S2 lists the images used as key frames in the model. [file 13007_2015_75_MOESM19_ESM.zip › side_view/side3_0146.jpg]

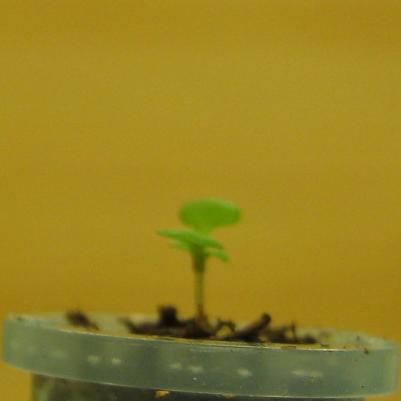

Supplement: Additional file 19 — Col-0 Side View Images for 3-D Model. Images of Col-0 captured every 10 min for 5 days from the side view for the 3-D CG model. Table S2 lists the images used as key frames in the model. [file 13007_2015_75_MOESM19_ESM.zip › side_view/side3_0147.jpg]

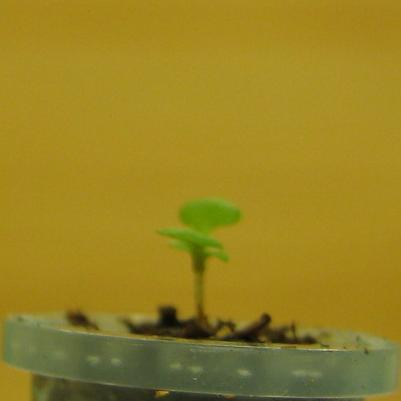

Supplement: Additional file 19 — Col-0 Side View Images for 3-D Model. Images of Col-0 captured every 10 min for 5 days from the side view for the 3-D CG model. Table S2 lists the images used as key frames in the model. [file 13007_2015_75_MOESM19_ESM.zip › side_view/side3_0148.jpg]

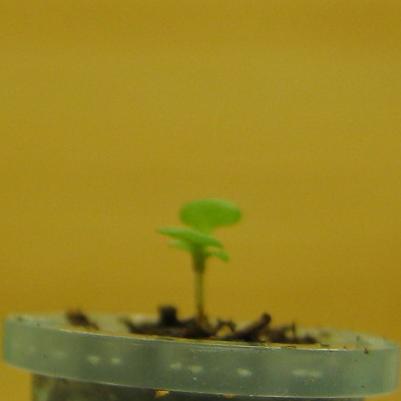

Supplement: Additional file 19 — Col-0 Side View Images for 3-D Model. Images of Col-0 captured every 10 min for 5 days from the side view for the 3-D CG model. Table S2 lists the images used as key frames in the model. [file 13007_2015_75_MOESM19_ESM.zip › side_view/side3_0149.jpg]

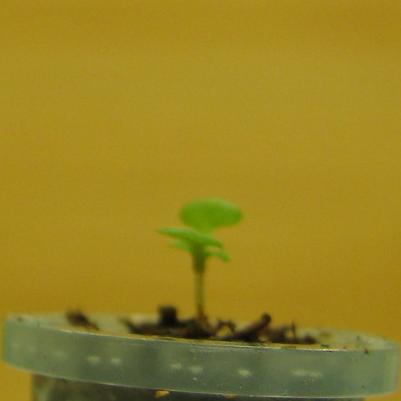

Supplement: Additional file 19 — Col-0 Side View Images for 3-D Model. Images of Col-0 captured every 10 min for 5 days from the side view for the 3-D CG model. Table S2 lists the images used as key frames in the model. [file 13007_2015_75_MOESM19_ESM.zip › side_view/side3_0150.jpg]

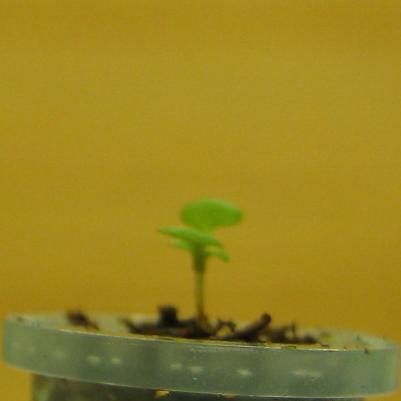

Supplement: Additional file 19 — Col-0 Side View Images for 3-D Model. Images of Col-0 captured every 10 min for 5 days from the side view for the 3-D CG model. Table S2 lists the images used as key frames in the model. [file 13007_2015_75_MOESM19_ESM.zip › side_view/side3_0151.jpg]

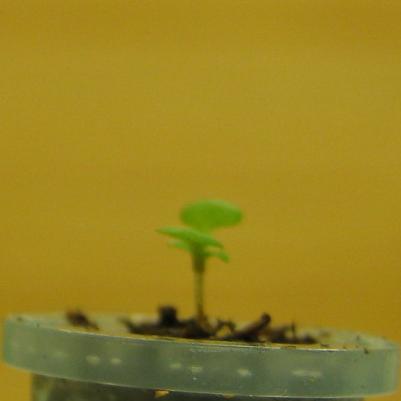

Supplement: Additional file 19 — Col-0 Side View Images for 3-D Model. Images of Col-0 captured every 10 min for 5 days from the side view for the 3-D CG model. Table S2 lists the images used as key frames in the model. [file 13007_2015_75_MOESM19_ESM.zip › side_view/side3_0152.jpg]

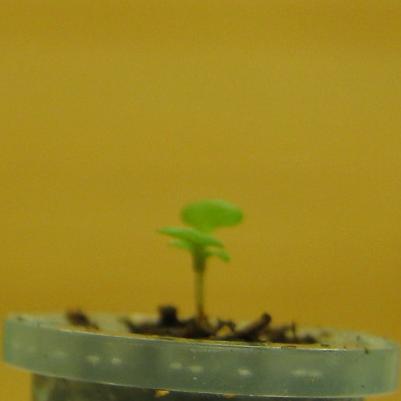

Supplement: Additional file 19 — Col-0 Side View Images for 3-D Model. Images of Col-0 captured every 10 min for 5 days from the side view for the 3-D CG model. Table S2 lists the images used as key frames in the model. [file 13007_2015_75_MOESM19_ESM.zip › side_view/side3_0153.jpg]

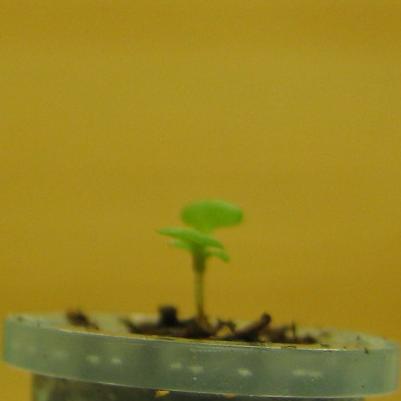

Supplement: Additional file 19 — Col-0 Side View Images for 3-D Model. Images of Col-0 captured every 10 min for 5 days from the side view for the 3-D CG model. Table S2 lists the images used as key frames in the model. [file 13007_2015_75_MOESM19_ESM.zip › side_view/side3_0154.jpg]

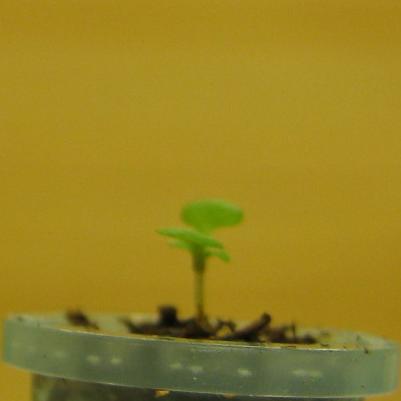

Supplement: Additional file 19 — Col-0 Side View Images for 3-D Model. Images of Col-0 captured every 10 min for 5 days from the side view for the 3-D CG model. Table S2 lists the images used as key frames in the model. [file 13007_2015_75_MOESM19_ESM.zip › side_view/side3_0155.jpg]

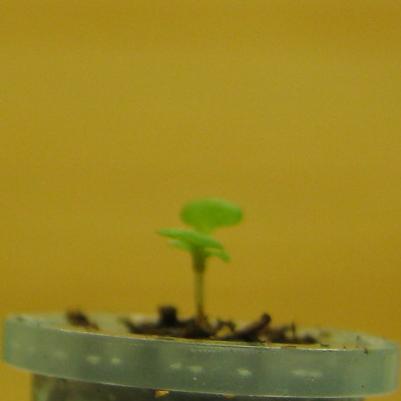

Supplement: Additional file 19 — Col-0 Side View Images for 3-D Model. Images of Col-0 captured every 10 min for 5 days from the side view for the 3-D CG model. Table S2 lists the images used as key frames in the model. [file 13007_2015_75_MOESM19_ESM.zip › side_view/side3_0156.jpg]

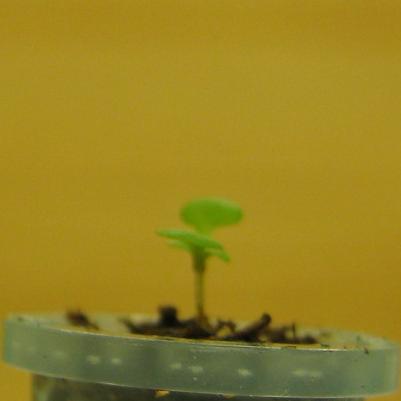

Supplement: Additional file 19 — Col-0 Side View Images for 3-D Model. Images of Col-0 captured every 10 min for 5 days from the side view for the 3-D CG model. Table S2 lists the images used as key frames in the model. [file 13007_2015_75_MOESM19_ESM.zip › side_view/side3_0157.jpg]

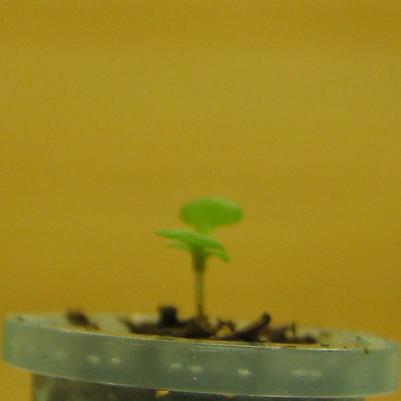

Supplement: Additional file 19 — Col-0 Side View Images for 3-D Model. Images of Col-0 captured every 10 min for 5 days from the side view for the 3-D CG model. Table S2 lists the images used as key frames in the model. [file 13007_2015_75_MOESM19_ESM.zip › side_view/side3_0158.jpg]

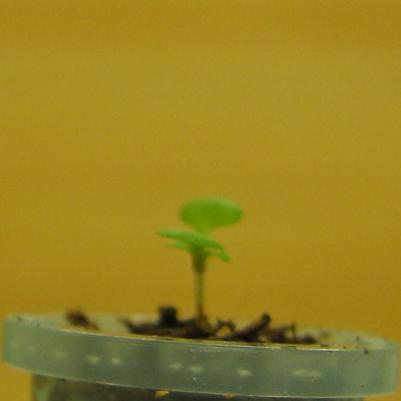

Supplement: Additional file 19 — Col-0 Side View Images for 3-D Model. Images of Col-0 captured every 10 min for 5 days from the side view for the 3-D CG model. Table S2 lists the images used as key frames in the model. [file 13007_2015_75_MOESM19_ESM.zip › side_view/side3_0159.jpg]

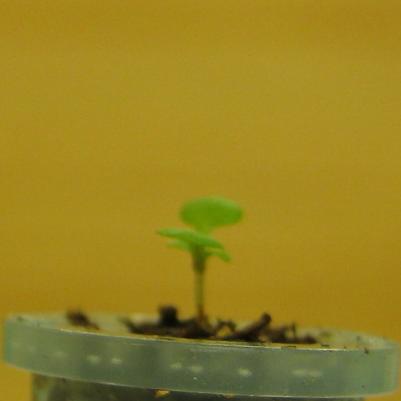

Supplement: Additional file 19 — Col-0 Side View Images for 3-D Model. Images of Col-0 captured every 10 min for 5 days from the side view for the 3-D CG model. Table S2 lists the images used as key frames in the model. [file 13007_2015_75_MOESM19_ESM.zip › side_view/side3_0160.jpg]

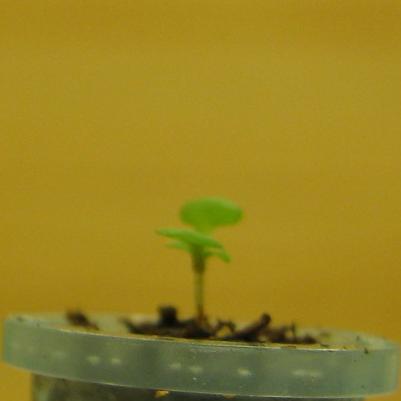

Supplement: Additional file 19 — Col-0 Side View Images for 3-D Model. Images of Col-0 captured every 10 min for 5 days from the side view for the 3-D CG model. Table S2 lists the images used as key frames in the model. [file 13007_2015_75_MOESM19_ESM.zip › side_view/side3_0161.jpg]

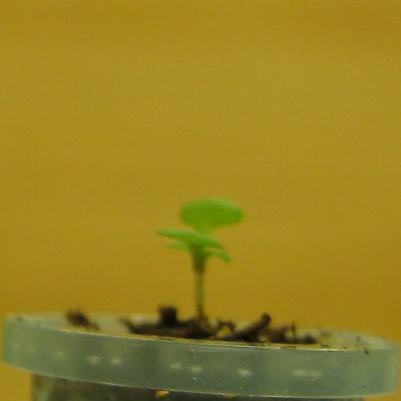

Supplement: Additional file 19 — Col-0 Side View Images for 3-D Model. Images of Col-0 captured every 10 min for 5 days from the side view for the 3-D CG model. Table S2 lists the images used as key frames in the model. [file 13007_2015_75_MOESM19_ESM.zip › side_view/side3_0162.jpg]

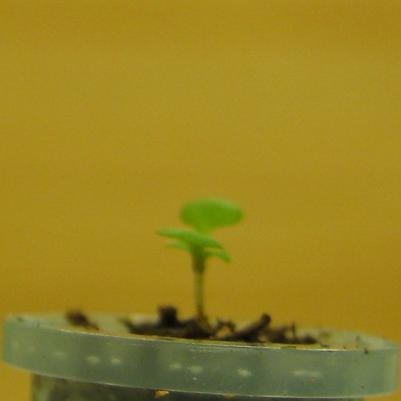

Supplement: Additional file 19 — Col-0 Side View Images for 3-D Model. Images of Col-0 captured every 10 min for 5 days from the side view for the 3-D CG model. Table S2 lists the images used as key frames in the model. [file 13007_2015_75_MOESM19_ESM.zip › side_view/side3_0163.jpg]

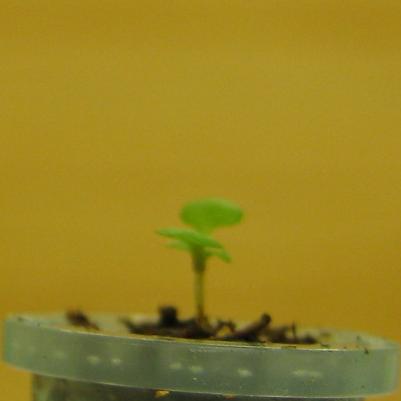

Supplement: Additional file 19 — Col-0 Side View Images for 3-D Model. Images of Col-0 captured every 10 min for 5 days from the side view for the 3-D CG model. Table S2 lists the images used as key frames in the model. [file 13007_2015_75_MOESM19_ESM.zip › side_view/side3_0164.jpg]

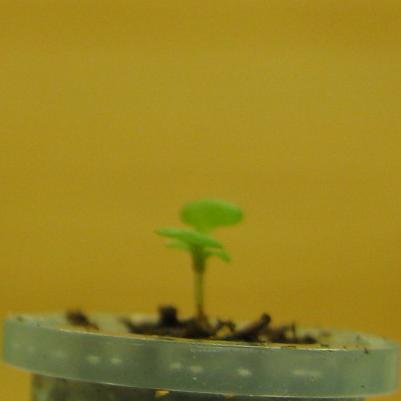

Supplement: Additional file 19 — Col-0 Side View Images for 3-D Model. Images of Col-0 captured every 10 min for 5 days from the side view for the 3-D CG model. Table S2 lists the images used as key frames in the model. [file 13007_2015_75_MOESM19_ESM.zip › side_view/side3_0165.jpg]

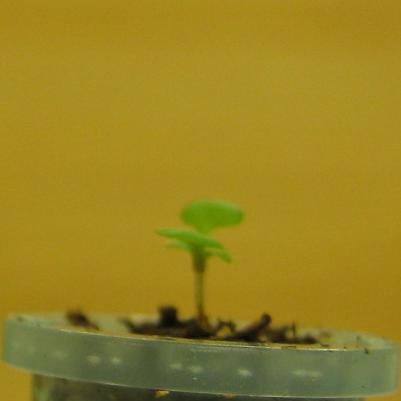

Supplement: Additional file 19 — Col-0 Side View Images for 3-D Model. Images of Col-0 captured every 10 min for 5 days from the side view for the 3-D CG model. Table S2 lists the images used as key frames in the model. [file 13007_2015_75_MOESM19_ESM.zip › side_view/side3_0166.jpg]

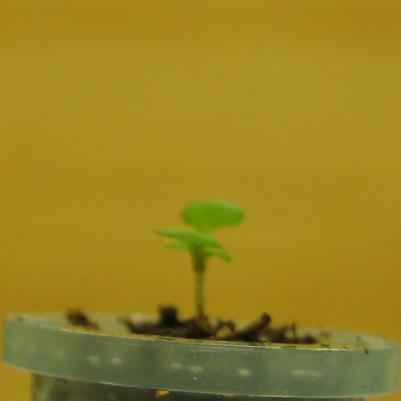

Supplement: Additional file 19 — Col-0 Side View Images for 3-D Model. Images of Col-0 captured every 10 min for 5 days from the side view for the 3-D CG model. Table S2 lists the images used as key frames in the model. [file 13007_2015_75_MOESM19_ESM.zip › side_view/side3_0167.jpg]

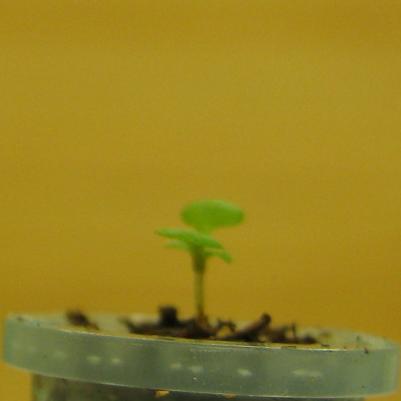

Supplement: Additional file 19 — Col-0 Side View Images for 3-D Model. Images of Col-0 captured every 10 min for 5 days from the side view for the 3-D CG model. Table S2 lists the images used as key frames in the model. [file 13007_2015_75_MOESM19_ESM.zip › side_view/side3_0168.jpg]

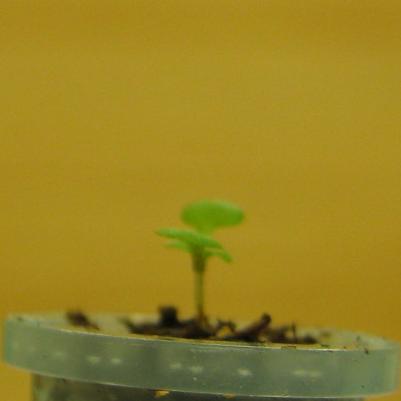

Supplement: Additional file 19 — Col-0 Side View Images for 3-D Model. Images of Col-0 captured every 10 min for 5 days from the side view for the 3-D CG model. Table S2 lists the images used as key frames in the model. [file 13007_2015_75_MOESM19_ESM.zip › side_view/side3_0169.jpg]

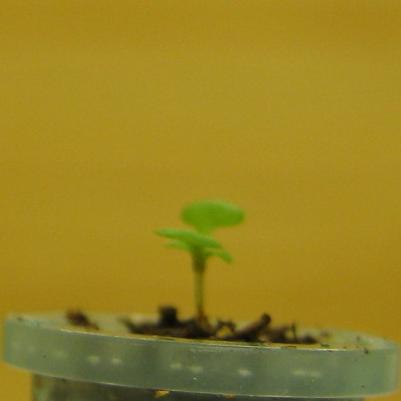

Supplement: Additional file 19 — Col-0 Side View Images for 3-D Model. Images of Col-0 captured every 10 min for 5 days from the side view for the 3-D CG model. Table S2 lists the images used as key frames in the model. [file 13007_2015_75_MOESM19_ESM.zip › side_view/side3_0170.jpg]

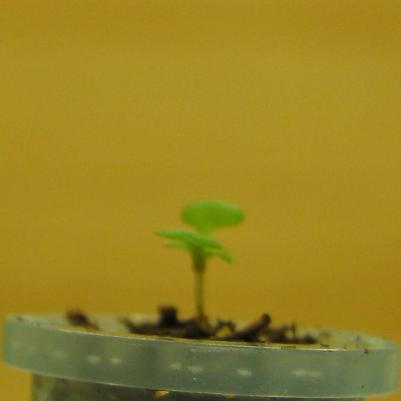

Supplement: Additional file 19 — Col-0 Side View Images for 3-D Model. Images of Col-0 captured every 10 min for 5 days from the side view for the 3-D CG model. Table S2 lists the images used as key frames in the model. [file 13007_2015_75_MOESM19_ESM.zip › side_view/side3_0171.jpg]

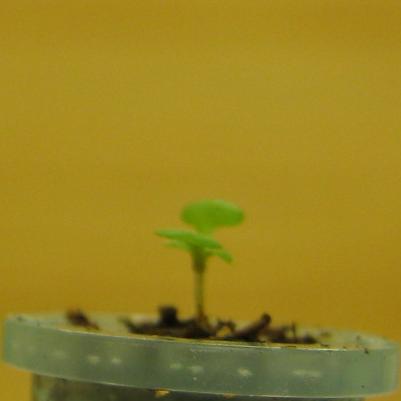

Supplement: Additional file 19 — Col-0 Side View Images for 3-D Model. Images of Col-0 captured every 10 min for 5 days from the side view for the 3-D CG model. Table S2 lists the images used as key frames in the model. [file 13007_2015_75_MOESM19_ESM.zip › side_view/side3_0172.jpg]

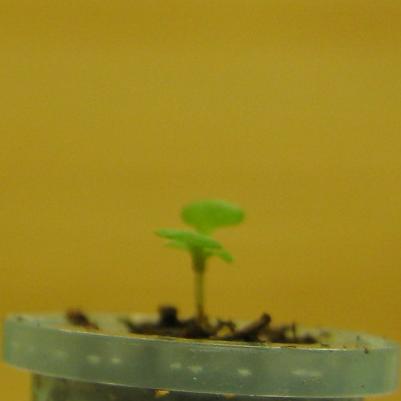

Supplement: Additional file 19 — Col-0 Side View Images for 3-D Model. Images of Col-0 captured every 10 min for 5 days from the side view for the 3-D CG model. Table S2 lists the images used as key frames in the model. [file 13007_2015_75_MOESM19_ESM.zip › side_view/side3_0173.jpg]

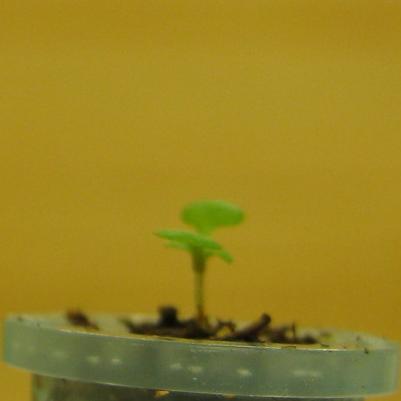

Supplement: Additional file 19 — Col-0 Side View Images for 3-D Model. Images of Col-0 captured every 10 min for 5 days from the side view for the 3-D CG model. Table S2 lists the images used as key frames in the model. [file 13007_2015_75_MOESM19_ESM.zip › side_view/side3_0174.jpg]

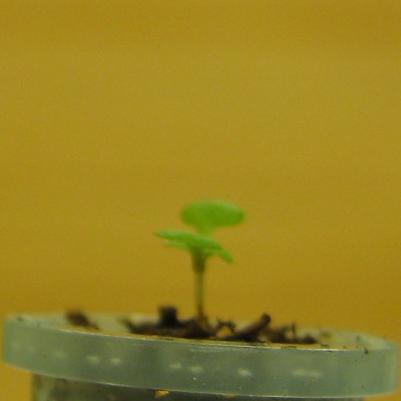

Supplement: Additional file 19 — Col-0 Side View Images for 3-D Model. Images of Col-0 captured every 10 min for 5 days from the side view for the 3-D CG model. Table S2 lists the images used as key frames in the model. [file 13007_2015_75_MOESM19_ESM.zip › side_view/side3_0175.jpg]

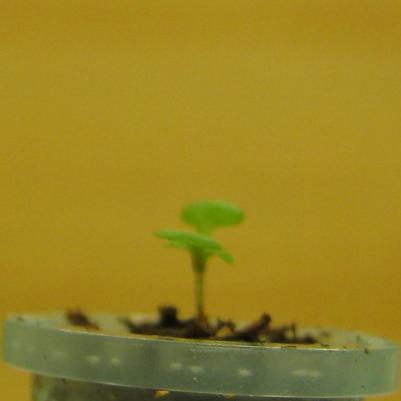

Supplement: Additional file 19 — Col-0 Side View Images for 3-D Model. Images of Col-0 captured every 10 min for 5 days from the side view for the 3-D CG model. Table S2 lists the images used as key frames in the model. [file 13007_2015_75_MOESM19_ESM.zip › side_view/side3_0176.jpg]

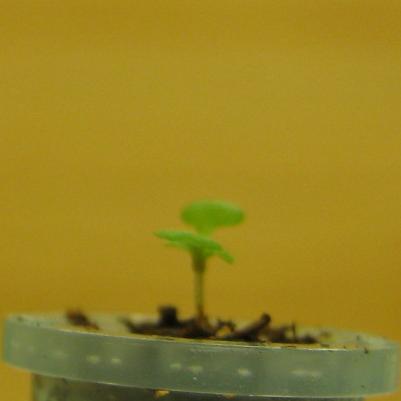

Supplement: Additional file 19 — Col-0 Side View Images for 3-D Model. Images of Col-0 captured every 10 min for 5 days from the side view for the 3-D CG model. Table S2 lists the images used as key frames in the model. [file 13007_2015_75_MOESM19_ESM.zip › side_view/side3_0177.jpg]

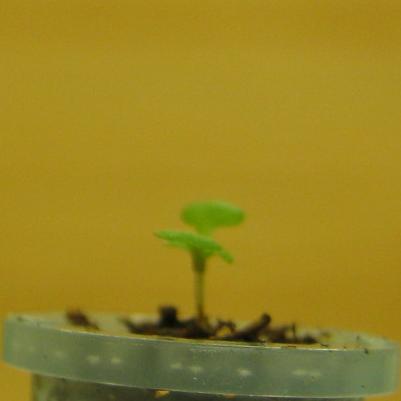

Supplement: Additional file 19 — Col-0 Side View Images for 3-D Model. Images of Col-0 captured every 10 min for 5 days from the side view for the 3-D CG model. Table S2 lists the images used as key frames in the model. [file 13007_2015_75_MOESM19_ESM.zip › side_view/side3_0178.jpg]

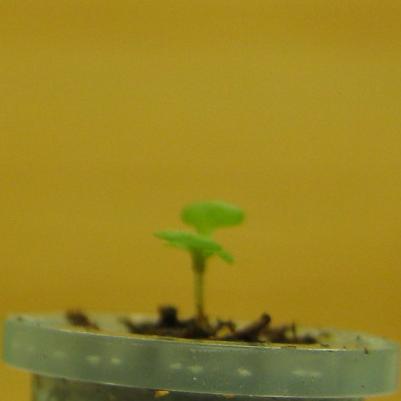

Supplement: Additional file 19 — Col-0 Side View Images for 3-D Model. Images of Col-0 captured every 10 min for 5 days from the side view for the 3-D CG model. Table S2 lists the images used as key frames in the model. [file 13007_2015_75_MOESM19_ESM.zip › side_view/side3_0179.jpg]

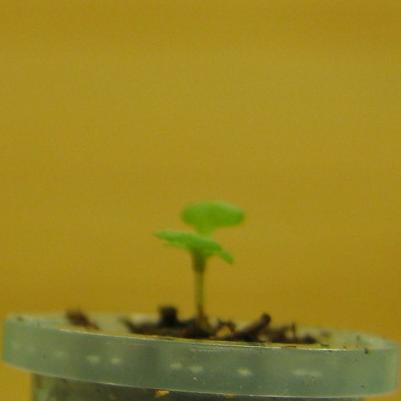

Supplement: Additional file 19 — Col-0 Side View Images for 3-D Model. Images of Col-0 captured every 10 min for 5 days from the side view for the 3-D CG model. Table S2 lists the images used as key frames in the model. [file 13007_2015_75_MOESM19_ESM.zip › side_view/side3_0180.jpg]

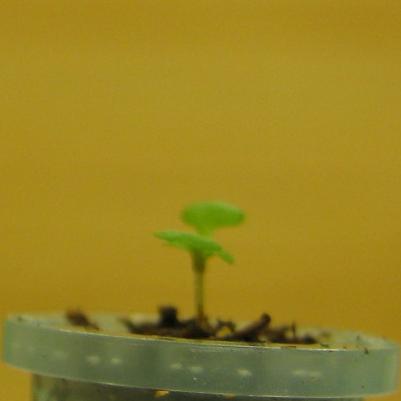

Supplement: Additional file 19 — Col-0 Side View Images for 3-D Model. Images of Col-0 captured every 10 min for 5 days from the side view for the 3-D CG model. Table S2 lists the images used as key frames in the model. [file 13007_2015_75_MOESM19_ESM.zip › side_view/side3_0181.jpg]

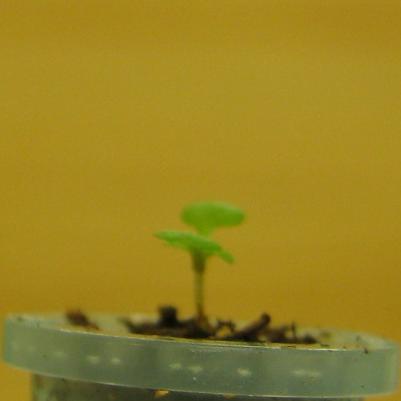

Supplement: Additional file 19 — Col-0 Side View Images for 3-D Model. Images of Col-0 captured every 10 min for 5 days from the side view for the 3-D CG model. Table S2 lists the images used as key frames in the model. [file 13007_2015_75_MOESM19_ESM.zip › side_view/side3_0182.jpg]

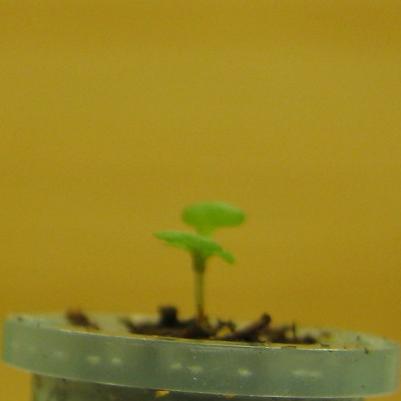

Supplement: Additional file 19 — Col-0 Side View Images for 3-D Model. Images of Col-0 captured every 10 min for 5 days from the side view for the 3-D CG model. Table S2 lists the images used as key frames in the model. [file 13007_2015_75_MOESM19_ESM.zip › side_view/side3_0183.jpg]

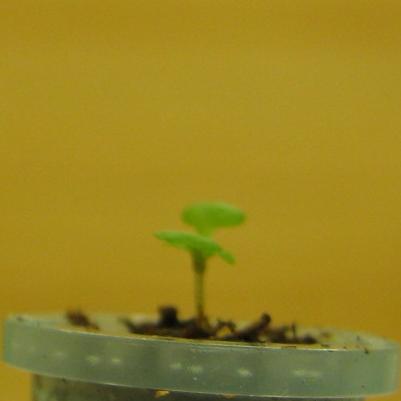

Supplement: Additional file 19 — Col-0 Side View Images for 3-D Model. Images of Col-0 captured every 10 min for 5 days from the side view for the 3-D CG model. Table S2 lists the images used as key frames in the model. [file 13007_2015_75_MOESM19_ESM.zip › side_view/side3_0184.jpg]

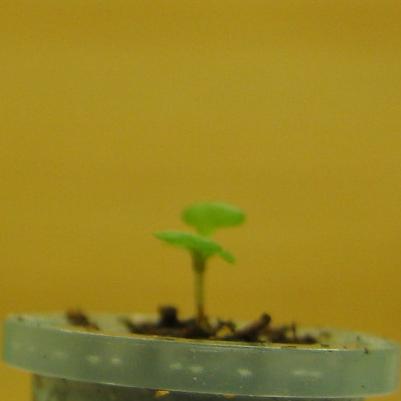

Supplement: Additional file 19 — Col-0 Side View Images for 3-D Model. Images of Col-0 captured every 10 min for 5 days from the side view for the 3-D CG model. Table S2 lists the images used as key frames in the model. [file 13007_2015_75_MOESM19_ESM.zip › side_view/side3_0185.jpg]

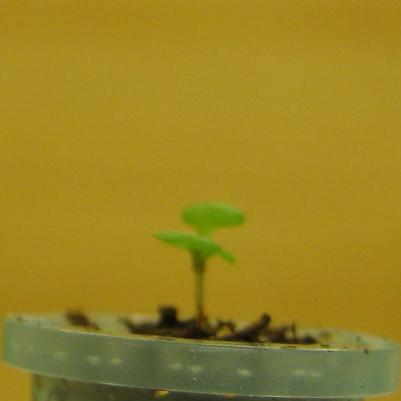

Supplement: Additional file 19 — Col-0 Side View Images for 3-D Model. Images of Col-0 captured every 10 min for 5 days from the side view for the 3-D CG model. Table S2 lists the images used as key frames in the model. [file 13007_2015_75_MOESM19_ESM.zip › side_view/side3_0186.jpg]

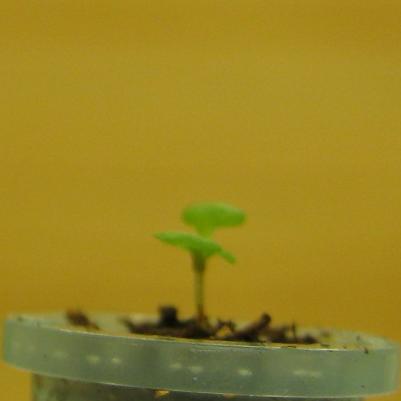

Supplement: Additional file 19 — Col-0 Side View Images for 3-D Model. Images of Col-0 captured every 10 min for 5 days from the side view for the 3-D CG model. Table S2 lists the images used as key frames in the model. [file 13007_2015_75_MOESM19_ESM.zip › side_view/side3_0187.jpg]

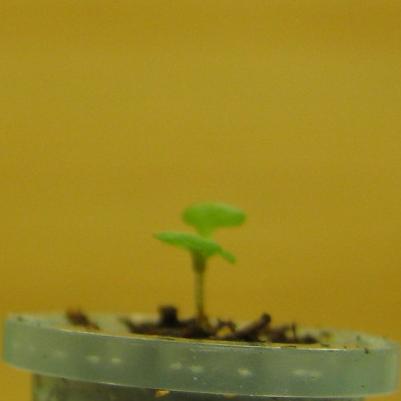

Supplement: Additional file 19 — Col-0 Side View Images for 3-D Model. Images of Col-0 captured every 10 min for 5 days from the side view for the 3-D CG model. Table S2 lists the images used as key frames in the model. [file 13007_2015_75_MOESM19_ESM.zip › side_view/side3_0188.jpg]

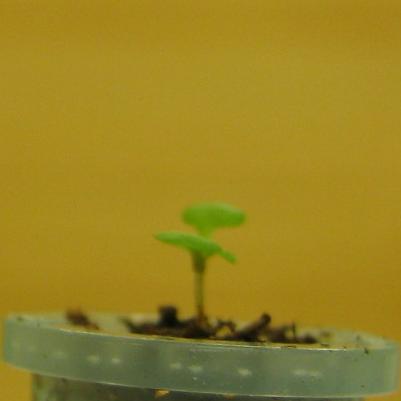

Supplement: Additional file 19 — Col-0 Side View Images for 3-D Model. Images of Col-0 captured every 10 min for 5 days from the side view for the 3-D CG model. Table S2 lists the images used as key frames in the model. [file 13007_2015_75_MOESM19_ESM.zip › side_view/side3_0189.jpg]

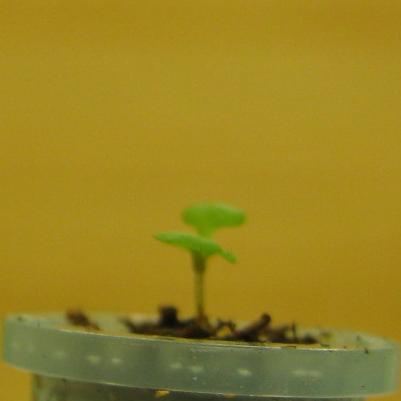

Supplement: Additional file 19 — Col-0 Side View Images for 3-D Model. Images of Col-0 captured every 10 min for 5 days from the side view for the 3-D CG model. Table S2 lists the images used as key frames in the model. [file 13007_2015_75_MOESM19_ESM.zip › side_view/side3_0190.jpg]

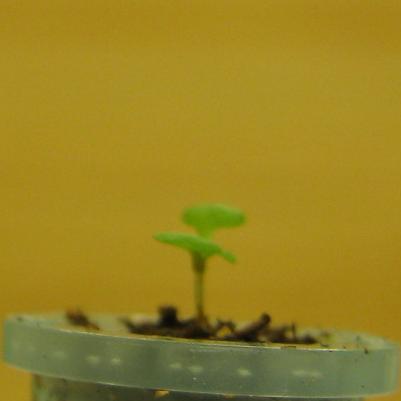

Supplement: Additional file 19 — Col-0 Side View Images for 3-D Model. Images of Col-0 captured every 10 min for 5 days from the side view for the 3-D CG model. Table S2 lists the images used as key frames in the model. [file 13007_2015_75_MOESM19_ESM.zip › side_view/side3_0191.jpg]

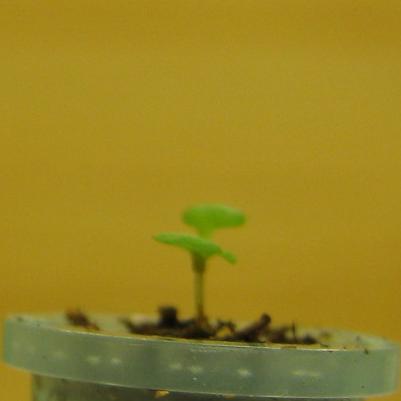

Supplement: Additional file 19 — Col-0 Side View Images for 3-D Model. Images of Col-0 captured every 10 min for 5 days from the side view for the 3-D CG model. Table S2 lists the images used as key frames in the model. [file 13007_2015_75_MOESM19_ESM.zip › side_view/side3_0192.jpg]

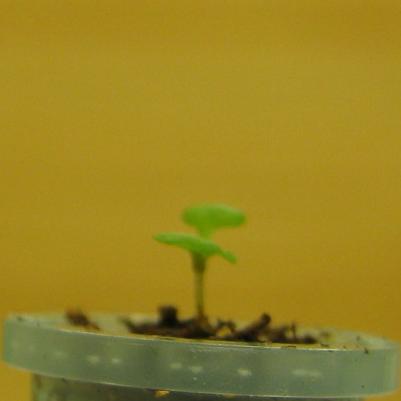

Supplement: Additional file 19 — Col-0 Side View Images for 3-D Model. Images of Col-0 captured every 10 min for 5 days from the side view for the 3-D CG model. Table S2 lists the images used as key frames in the model. [file 13007_2015_75_MOESM19_ESM.zip › side_view/side3_0193.jpg]

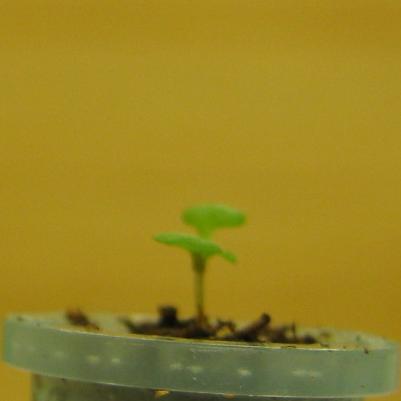

Supplement: Additional file 19 — Col-0 Side View Images for 3-D Model. Images of Col-0 captured every 10 min for 5 days from the side view for the 3-D CG model. Table S2 lists the images used as key frames in the model. [file 13007_2015_75_MOESM19_ESM.zip › side_view/side3_0194.jpg]

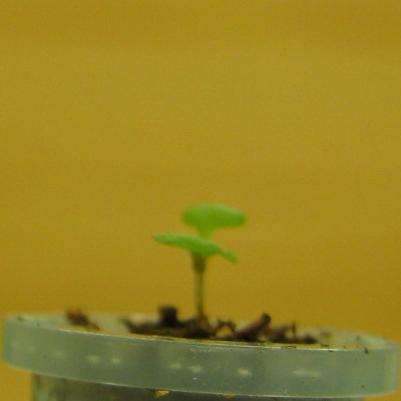

Supplement: Additional file 19 — Col-0 Side View Images for 3-D Model. Images of Col-0 captured every 10 min for 5 days from the side view for the 3-D CG model. Table S2 lists the images used as key frames in the model. [file 13007_2015_75_MOESM19_ESM.zip › side_view/side3_0195.jpg]

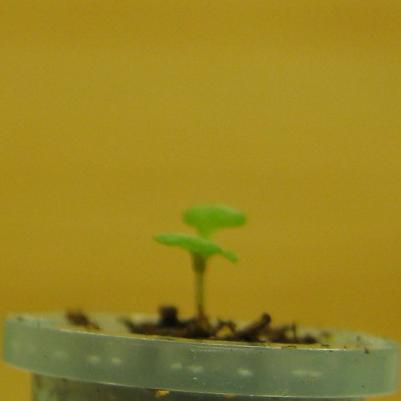

Supplement: Additional file 19 — Col-0 Side View Images for 3-D Model. Images of Col-0 captured every 10 min for 5 days from the side view for the 3-D CG model. Table S2 lists the images used as key frames in the model. [file 13007_2015_75_MOESM19_ESM.zip › side_view/side3_0196.jpg]

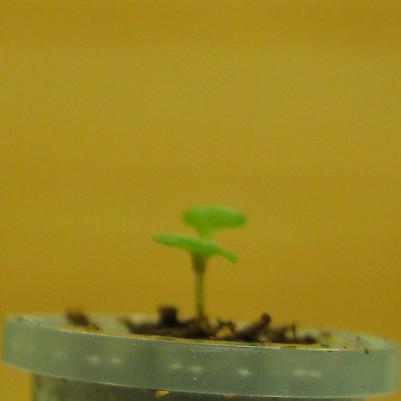

Supplement: Additional file 19 — Col-0 Side View Images for 3-D Model. Images of Col-0 captured every 10 min for 5 days from the side view for the 3-D CG model. Table S2 lists the images used as key frames in the model. [file 13007_2015_75_MOESM19_ESM.zip › side_view/side3_0197.jpg]

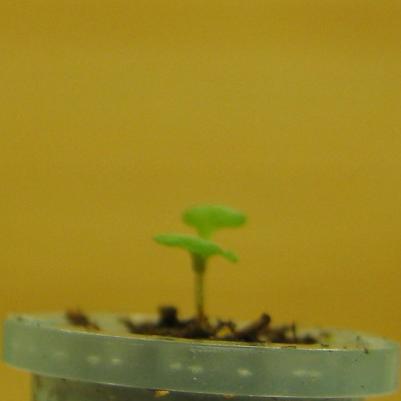

Supplement: Additional file 19 — Col-0 Side View Images for 3-D Model. Images of Col-0 captured every 10 min for 5 days from the side view for the 3-D CG model. Table S2 lists the images used as key frames in the model. [file 13007_2015_75_MOESM19_ESM.zip › side_view/side3_0198.jpg]

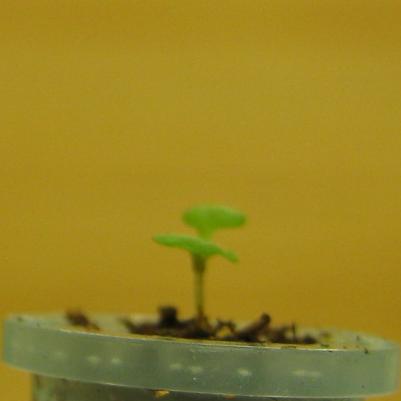

Supplement: Additional file 19 — Col-0 Side View Images for 3-D Model. Images of Col-0 captured every 10 min for 5 days from the side view for the 3-D CG model. Table S2 lists the images used as key frames in the model. [file 13007_2015_75_MOESM19_ESM.zip › side_view/side3_0199.jpg]

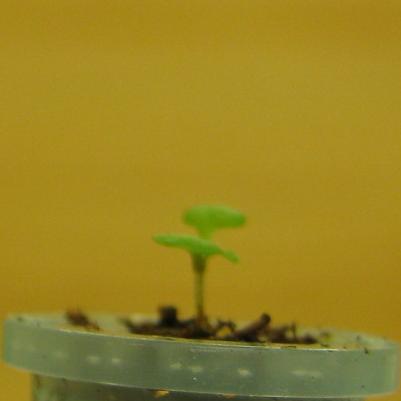

Supplement: Additional file 19 — Col-0 Side View Images for 3-D Model. Images of Col-0 captured every 10 min for 5 days from the side view for the 3-D CG model. Table S2 lists the images used as key frames in the model. [file 13007_2015_75_MOESM19_ESM.zip › side_view/side3_0200.jpg]

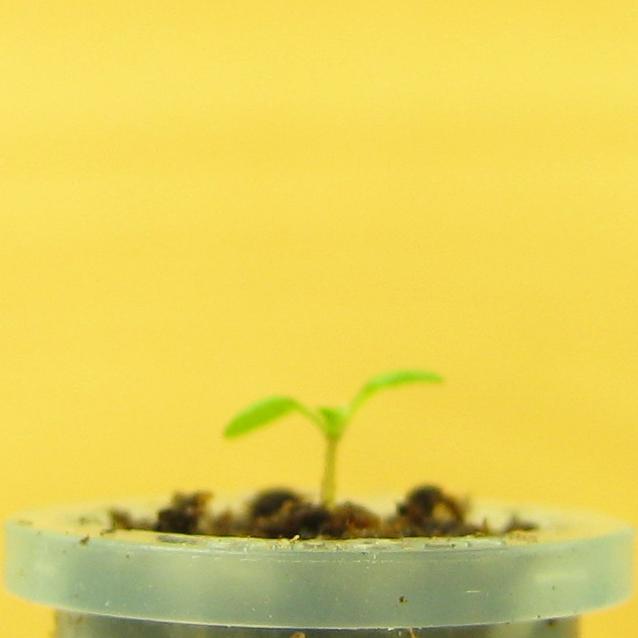

Supplement: Additional file 20 — Col-0 Front View Images for 3-D Model. Images of Col-0 captured every 10 min for 5 days from the front view for the 3-D CG model. Table S2 lists the images used as key frames in the model. [file 13007_2015_75_MOESM20_ESM.zip › front_view/side12_0001.jpg]

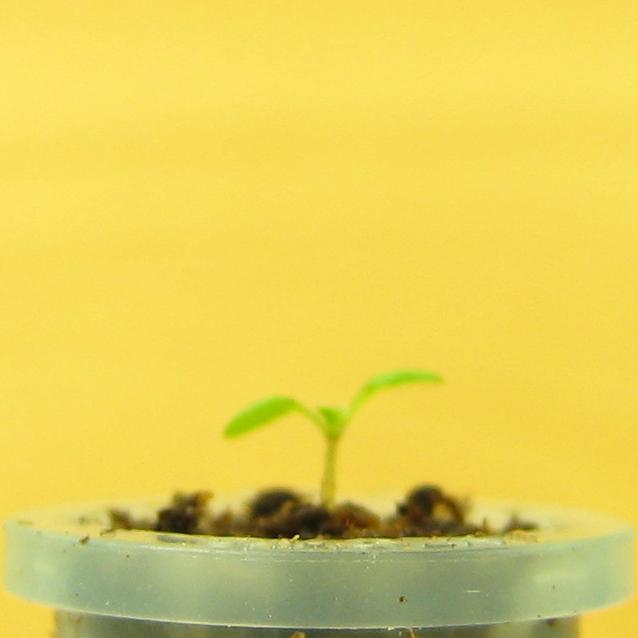

Supplement: Additional file 20 — Col-0 Front View Images for 3-D Model. Images of Col-0 captured every 10 min for 5 days from the front view for the 3-D CG model. Table S2 lists the images used as key frames in the model. [file 13007_2015_75_MOESM20_ESM.zip › front_view/side12_0002.jpg]

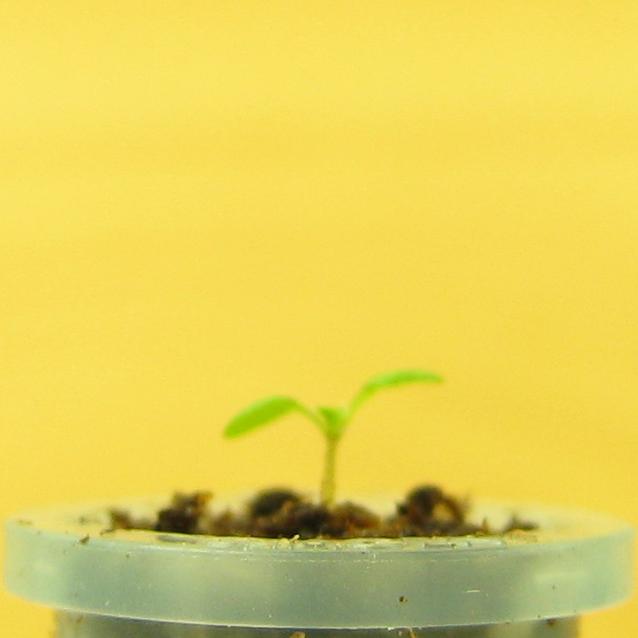

Supplement: Additional file 20 — Col-0 Front View Images for 3-D Model. Images of Col-0 captured every 10 min for 5 days from the front view for the 3-D CG model. Table S2 lists the images used as key frames in the model. [file 13007_2015_75_MOESM20_ESM.zip › front_view/side12_0003.jpg]

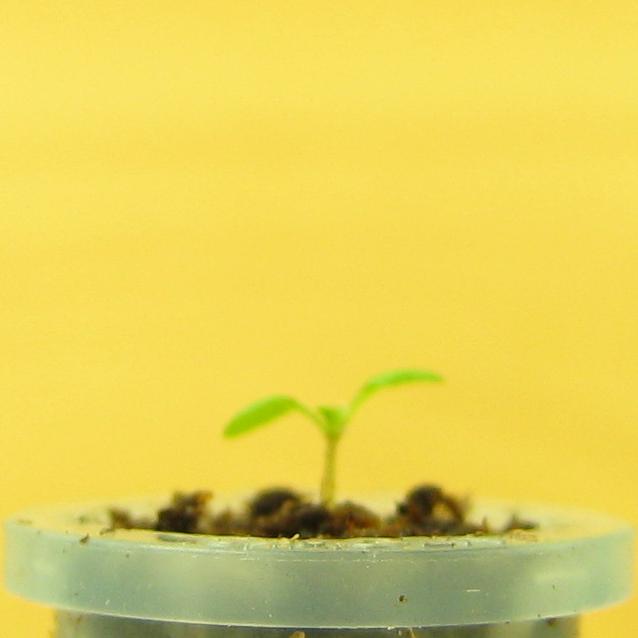

Supplement: Additional file 20 — Col-0 Front View Images for 3-D Model. Images of Col-0 captured every 10 min for 5 days from the front view for the 3-D CG model. Table S2 lists the images used as key frames in the model. [file 13007_2015_75_MOESM20_ESM.zip › front_view/side12_0004.jpg]

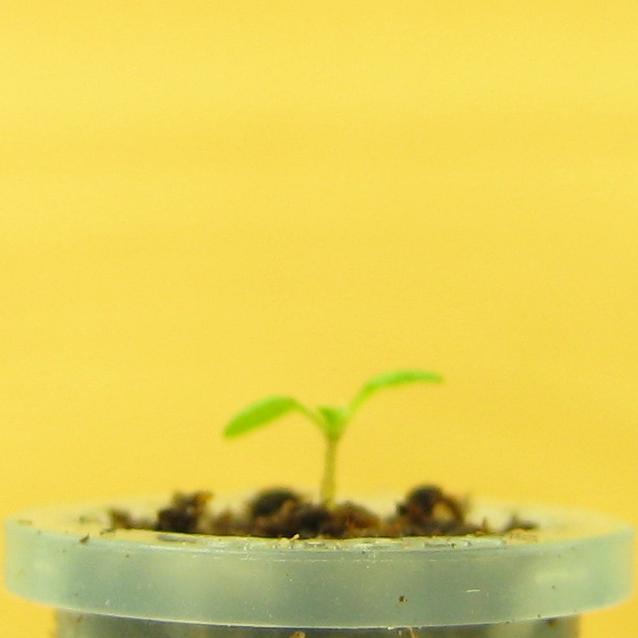

Supplement: Additional file 20 — Col-0 Front View Images for 3-D Model. Images of Col-0 captured every 10 min for 5 days from the front view for the 3-D CG model. Table S2 lists the images used as key frames in the model. [file 13007_2015_75_MOESM20_ESM.zip › front_view/side12_0005.jpg]

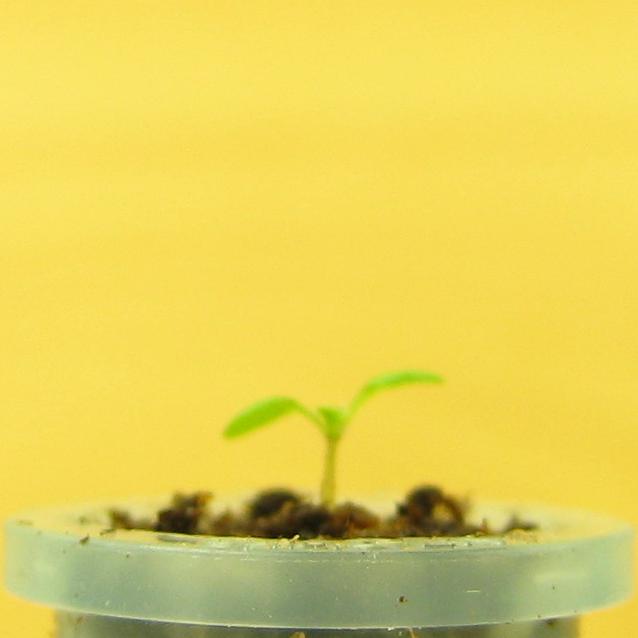

Supplement: Additional file 20 — Col-0 Front View Images for 3-D Model. Images of Col-0 captured every 10 min for 5 days from the front view for the 3-D CG model. Table S2 lists the images used as key frames in the model. [file 13007_2015_75_MOESM20_ESM.zip › front_view/side12_0006.jpg]

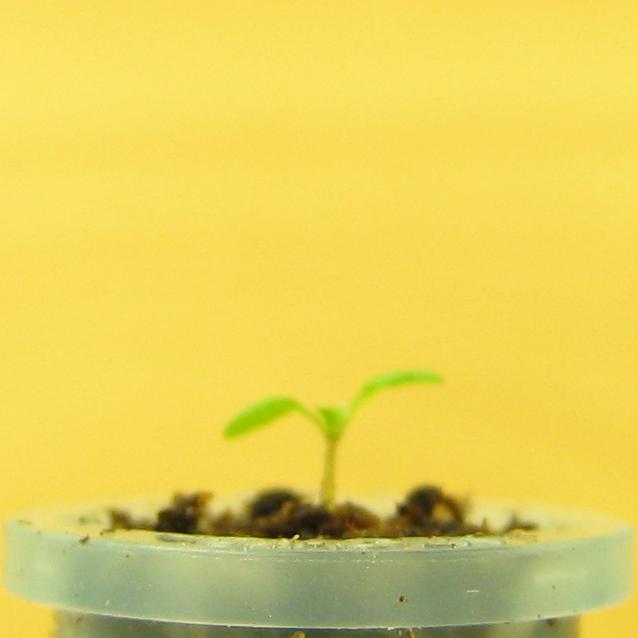

Supplement: Additional file 20 — Col-0 Front View Images for 3-D Model. Images of Col-0 captured every 10 min for 5 days from the front view for the 3-D CG model. Table S2 lists the images used as key frames in the model. [file 13007_2015_75_MOESM20_ESM.zip › front_view/side12_0007.jpg]

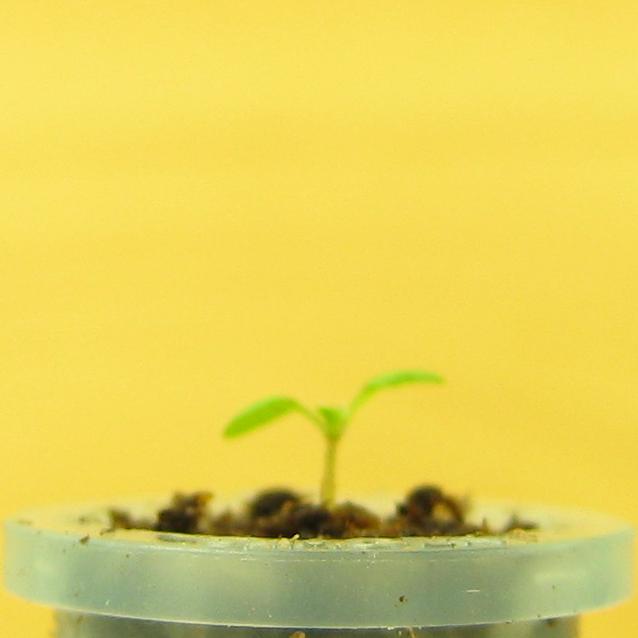

Supplement: Additional file 20 — Col-0 Front View Images for 3-D Model. Images of Col-0 captured every 10 min for 5 days from the front view for the 3-D CG model. Table S2 lists the images used as key frames in the model. [file 13007_2015_75_MOESM20_ESM.zip › front_view/side12_0008.jpg]
