# Supplementary figures and images for: TRiP: Tracking Rhythms in Plants, an automated leaf movement analysis program for circadian period estimation (part 3 of 10)
Source: Plant Methods. 2015 May 3;11:33. doi: 10.1186/s13007-015-0075-5 (PMC4445800; doi:10.1186/s13007-015-0075-5)

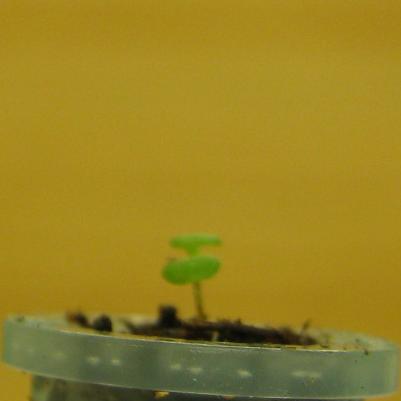

Supplement: Additional file 19 — Col-0 Side View Images for 3-D Model. Images of Col-0 captured every 10 min for 5 days from the side view for the 3-D CG model. Table S2 lists the images used as key frames in the model. [file 13007_2015_75_MOESM19_ESM.zip › side_view/side3_0009.jpg]

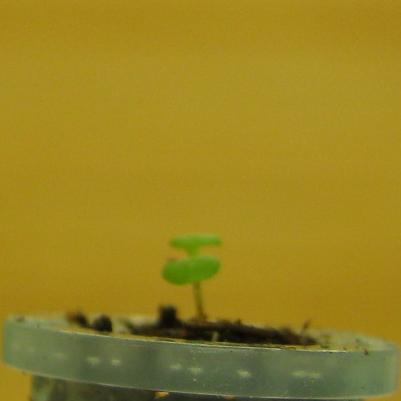

Supplement: Additional file 19 — Col-0 Side View Images for 3-D Model. Images of Col-0 captured every 10 min for 5 days from the side view for the 3-D CG model. Table S2 lists the images used as key frames in the model. [file 13007_2015_75_MOESM19_ESM.zip › side_view/side3_0010.jpg]

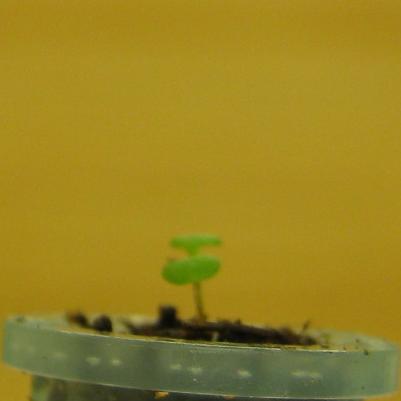

Supplement: Additional file 19 — Col-0 Side View Images for 3-D Model. Images of Col-0 captured every 10 min for 5 days from the side view for the 3-D CG model. Table S2 lists the images used as key frames in the model. [file 13007_2015_75_MOESM19_ESM.zip › side_view/side3_0011.jpg]

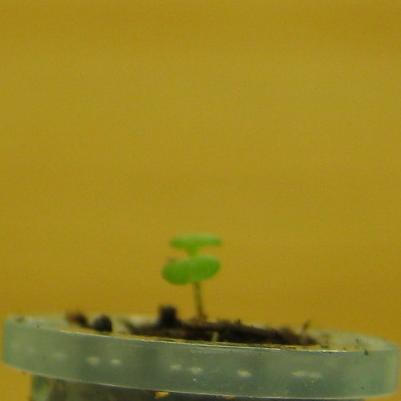

Supplement: Additional file 19 — Col-0 Side View Images for 3-D Model. Images of Col-0 captured every 10 min for 5 days from the side view for the 3-D CG model. Table S2 lists the images used as key frames in the model. [file 13007_2015_75_MOESM19_ESM.zip › side_view/side3_0012.jpg]

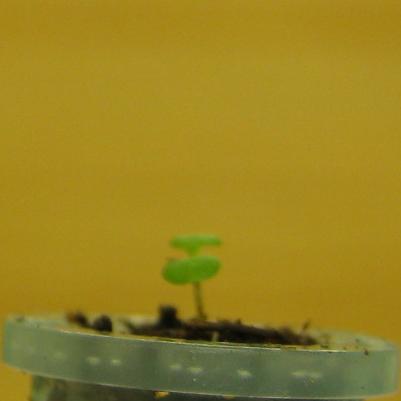

Supplement: Additional file 19 — Col-0 Side View Images for 3-D Model. Images of Col-0 captured every 10 min for 5 days from the side view for the 3-D CG model. Table S2 lists the images used as key frames in the model. [file 13007_2015_75_MOESM19_ESM.zip › side_view/side3_0013.jpg]

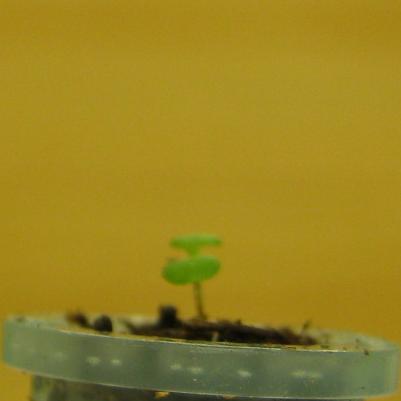

Supplement: Additional file 19 — Col-0 Side View Images for 3-D Model. Images of Col-0 captured every 10 min for 5 days from the side view for the 3-D CG model. Table S2 lists the images used as key frames in the model. [file 13007_2015_75_MOESM19_ESM.zip › side_view/side3_0014.jpg]

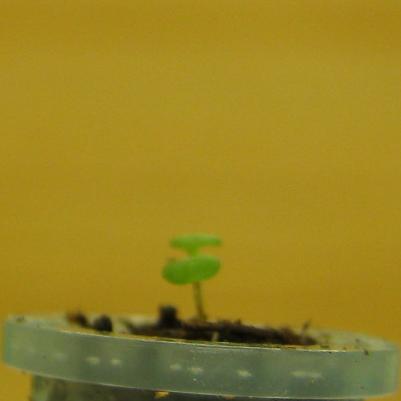

Supplement: Additional file 19 — Col-0 Side View Images for 3-D Model. Images of Col-0 captured every 10 min for 5 days from the side view for the 3-D CG model. Table S2 lists the images used as key frames in the model. [file 13007_2015_75_MOESM19_ESM.zip › side_view/side3_0015.jpg]

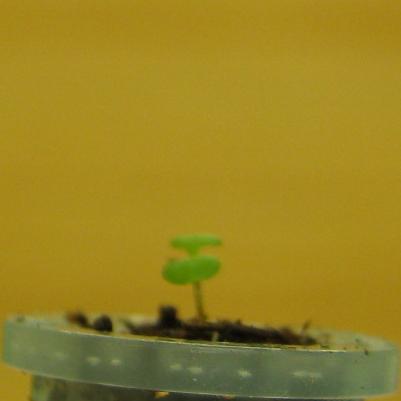

Supplement: Additional file 19 — Col-0 Side View Images for 3-D Model. Images of Col-0 captured every 10 min for 5 days from the side view for the 3-D CG model. Table S2 lists the images used as key frames in the model. [file 13007_2015_75_MOESM19_ESM.zip › side_view/side3_0016.jpg]

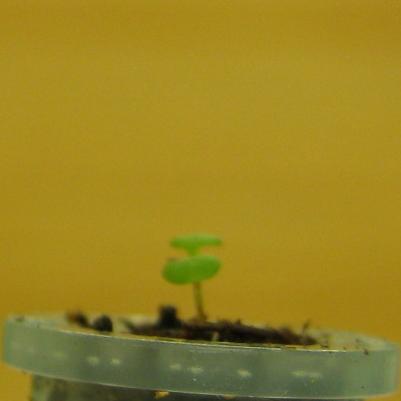

Supplement: Additional file 19 — Col-0 Side View Images for 3-D Model. Images of Col-0 captured every 10 min for 5 days from the side view for the 3-D CG model. Table S2 lists the images used as key frames in the model. [file 13007_2015_75_MOESM19_ESM.zip › side_view/side3_0017.jpg]

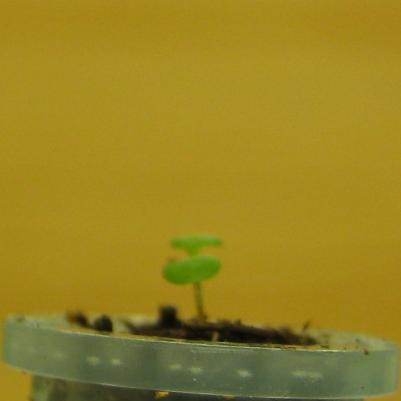

Supplement: Additional file 19 — Col-0 Side View Images for 3-D Model. Images of Col-0 captured every 10 min for 5 days from the side view for the 3-D CG model. Table S2 lists the images used as key frames in the model. [file 13007_2015_75_MOESM19_ESM.zip › side_view/side3_0018.jpg]

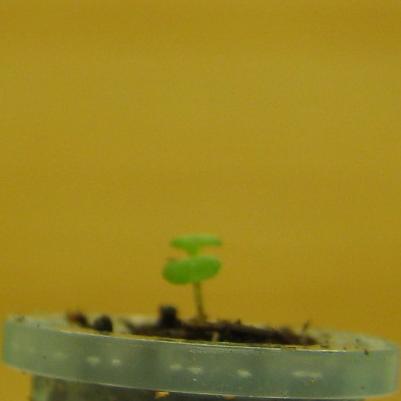

Supplement: Additional file 19 — Col-0 Side View Images for 3-D Model. Images of Col-0 captured every 10 min for 5 days from the side view for the 3-D CG model. Table S2 lists the images used as key frames in the model. [file 13007_2015_75_MOESM19_ESM.zip › side_view/side3_0019.jpg]

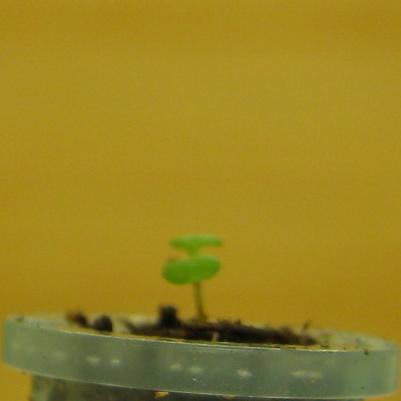

Supplement: Additional file 19 — Col-0 Side View Images for 3-D Model. Images of Col-0 captured every 10 min for 5 days from the side view for the 3-D CG model. Table S2 lists the images used as key frames in the model. [file 13007_2015_75_MOESM19_ESM.zip › side_view/side3_0020.jpg]

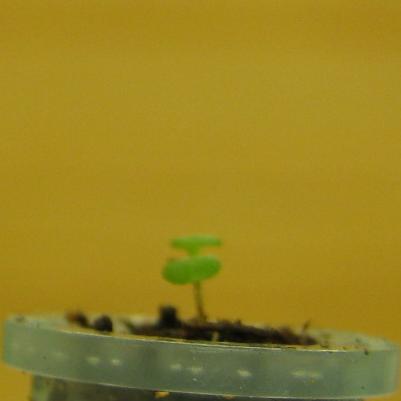

Supplement: Additional file 19 — Col-0 Side View Images for 3-D Model. Images of Col-0 captured every 10 min for 5 days from the side view for the 3-D CG model. Table S2 lists the images used as key frames in the model. [file 13007_2015_75_MOESM19_ESM.zip › side_view/side3_0021.jpg]

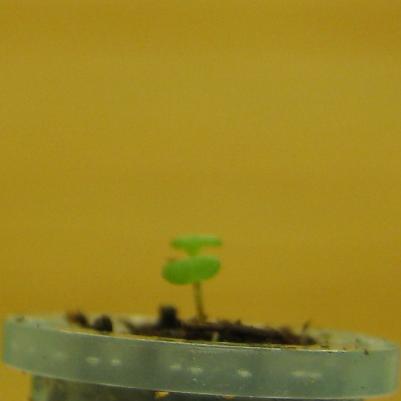

Supplement: Additional file 19 — Col-0 Side View Images for 3-D Model. Images of Col-0 captured every 10 min for 5 days from the side view for the 3-D CG model. Table S2 lists the images used as key frames in the model. [file 13007_2015_75_MOESM19_ESM.zip › side_view/side3_0022.jpg]

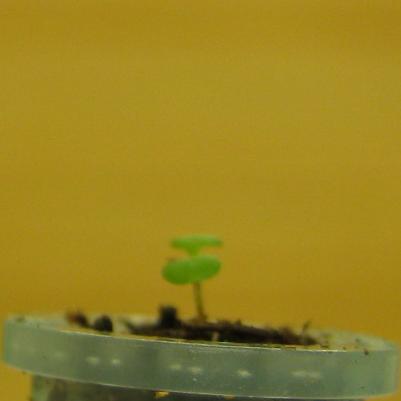

Supplement: Additional file 19 — Col-0 Side View Images for 3-D Model. Images of Col-0 captured every 10 min for 5 days from the side view for the 3-D CG model. Table S2 lists the images used as key frames in the model. [file 13007_2015_75_MOESM19_ESM.zip › side_view/side3_0023.jpg]

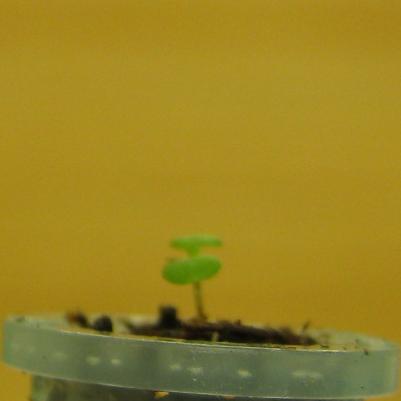

Supplement: Additional file 19 — Col-0 Side View Images for 3-D Model. Images of Col-0 captured every 10 min for 5 days from the side view for the 3-D CG model. Table S2 lists the images used as key frames in the model. [file 13007_2015_75_MOESM19_ESM.zip › side_view/side3_0024.jpg]

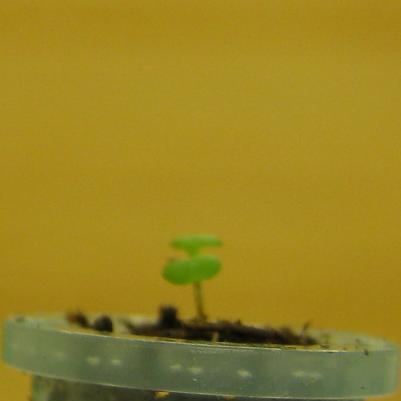

Supplement: Additional file 19 — Col-0 Side View Images for 3-D Model. Images of Col-0 captured every 10 min for 5 days from the side view for the 3-D CG model. Table S2 lists the images used as key frames in the model. [file 13007_2015_75_MOESM19_ESM.zip › side_view/side3_0025.jpg]

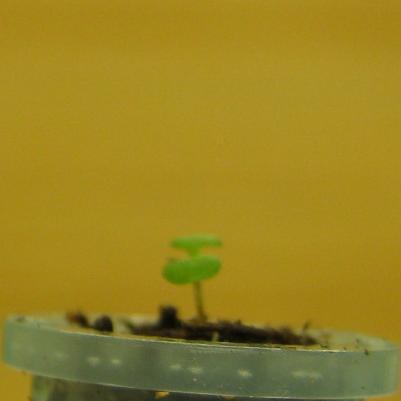

Supplement: Additional file 19 — Col-0 Side View Images for 3-D Model. Images of Col-0 captured every 10 min for 5 days from the side view for the 3-D CG model. Table S2 lists the images used as key frames in the model. [file 13007_2015_75_MOESM19_ESM.zip › side_view/side3_0026.jpg]

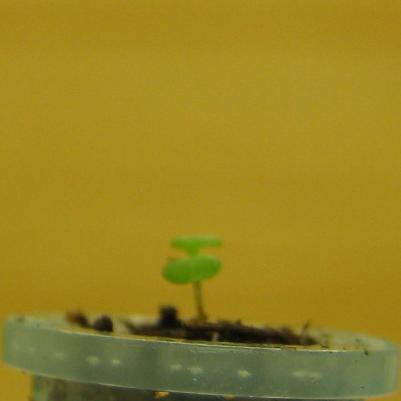

Supplement: Additional file 19 — Col-0 Side View Images for 3-D Model. Images of Col-0 captured every 10 min for 5 days from the side view for the 3-D CG model. Table S2 lists the images used as key frames in the model. [file 13007_2015_75_MOESM19_ESM.zip › side_view/side3_0027.jpg]

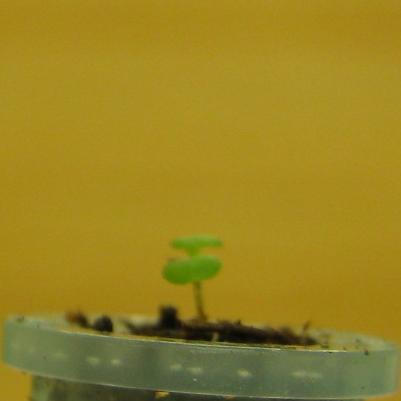

Supplement: Additional file 19 — Col-0 Side View Images for 3-D Model. Images of Col-0 captured every 10 min for 5 days from the side view for the 3-D CG model. Table S2 lists the images used as key frames in the model. [file 13007_2015_75_MOESM19_ESM.zip › side_view/side3_0028.jpg]

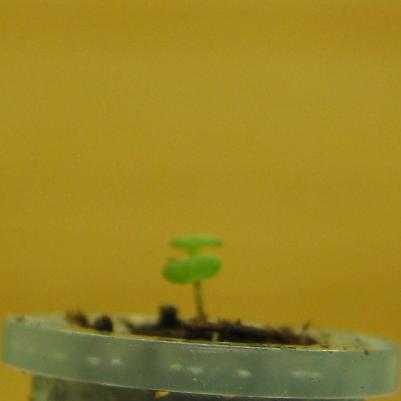

Supplement: Additional file 19 — Col-0 Side View Images for 3-D Model. Images of Col-0 captured every 10 min for 5 days from the side view for the 3-D CG model. Table S2 lists the images used as key frames in the model. [file 13007_2015_75_MOESM19_ESM.zip › side_view/side3_0029.jpg]

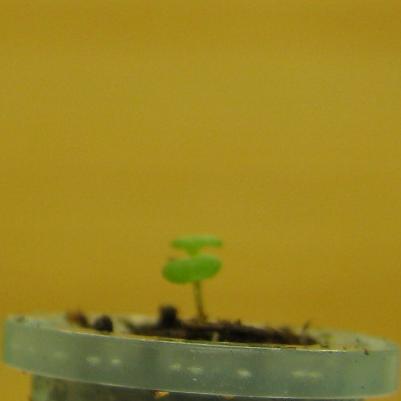

Supplement: Additional file 19 — Col-0 Side View Images for 3-D Model. Images of Col-0 captured every 10 min for 5 days from the side view for the 3-D CG model. Table S2 lists the images used as key frames in the model. [file 13007_2015_75_MOESM19_ESM.zip › side_view/side3_0030.jpg]

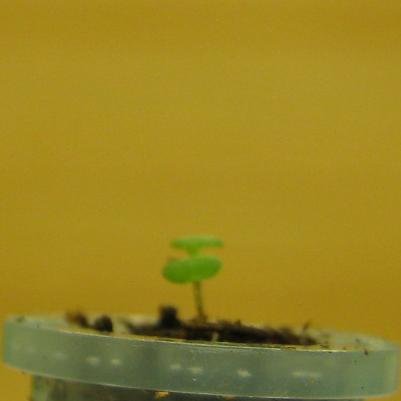

Supplement: Additional file 19 — Col-0 Side View Images for 3-D Model. Images of Col-0 captured every 10 min for 5 days from the side view for the 3-D CG model. Table S2 lists the images used as key frames in the model. [file 13007_2015_75_MOESM19_ESM.zip › side_view/side3_0031.jpg]

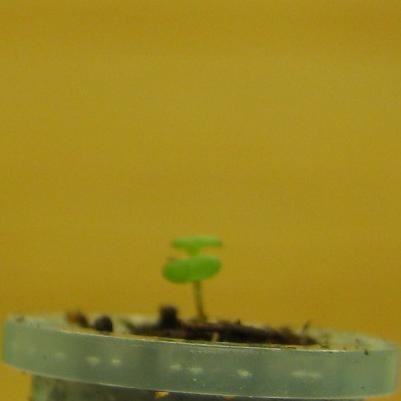

Supplement: Additional file 19 — Col-0 Side View Images for 3-D Model. Images of Col-0 captured every 10 min for 5 days from the side view for the 3-D CG model. Table S2 lists the images used as key frames in the model. [file 13007_2015_75_MOESM19_ESM.zip › side_view/side3_0032.jpg]

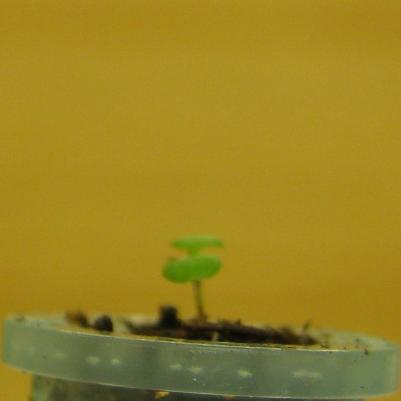

Supplement: Additional file 19 — Col-0 Side View Images for 3-D Model. Images of Col-0 captured every 10 min for 5 days from the side view for the 3-D CG model. Table S2 lists the images used as key frames in the model. [file 13007_2015_75_MOESM19_ESM.zip › side_view/side3_0033.jpg]

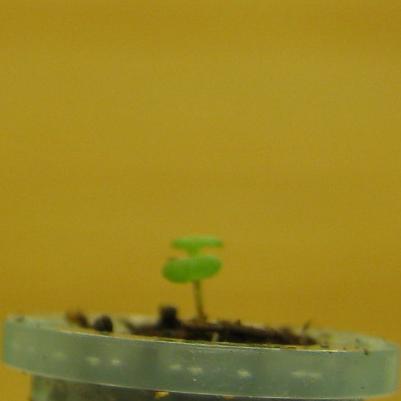

Supplement: Additional file 19 — Col-0 Side View Images for 3-D Model. Images of Col-0 captured every 10 min for 5 days from the side view for the 3-D CG model. Table S2 lists the images used as key frames in the model. [file 13007_2015_75_MOESM19_ESM.zip › side_view/side3_0034.jpg]

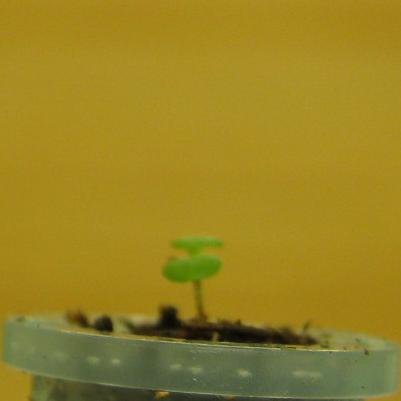

Supplement: Additional file 19 — Col-0 Side View Images for 3-D Model. Images of Col-0 captured every 10 min for 5 days from the side view for the 3-D CG model. Table S2 lists the images used as key frames in the model. [file 13007_2015_75_MOESM19_ESM.zip › side_view/side3_0035.jpg]

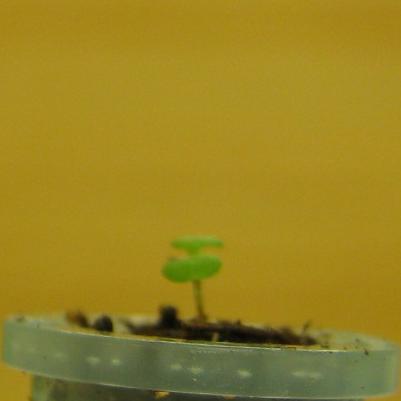

Supplement: Additional file 19 — Col-0 Side View Images for 3-D Model. Images of Col-0 captured every 10 min for 5 days from the side view for the 3-D CG model. Table S2 lists the images used as key frames in the model. [file 13007_2015_75_MOESM19_ESM.zip › side_view/side3_0036.jpg]

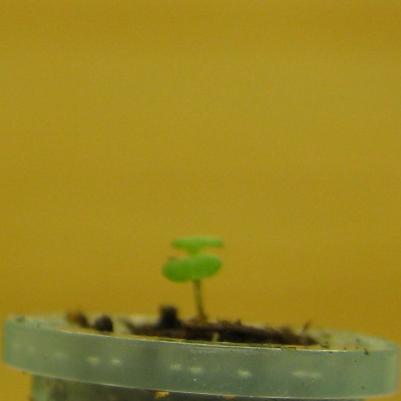

Supplement: Additional file 19 — Col-0 Side View Images for 3-D Model. Images of Col-0 captured every 10 min for 5 days from the side view for the 3-D CG model. Table S2 lists the images used as key frames in the model. [file 13007_2015_75_MOESM19_ESM.zip › side_view/side3_0037.jpg]

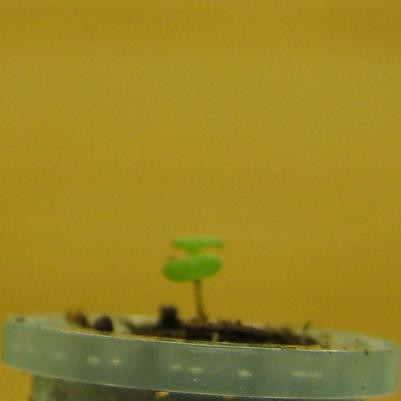

Supplement: Additional file 19 — Col-0 Side View Images for 3-D Model. Images of Col-0 captured every 10 min for 5 days from the side view for the 3-D CG model. Table S2 lists the images used as key frames in the model. [file 13007_2015_75_MOESM19_ESM.zip › side_view/side3_0038.jpg]

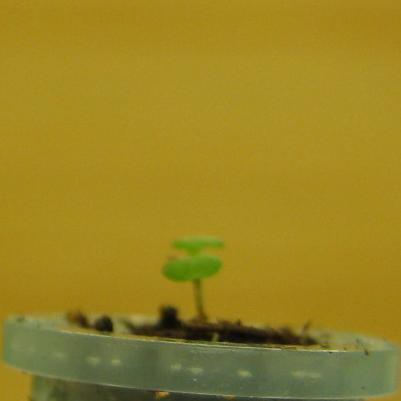

Supplement: Additional file 19 — Col-0 Side View Images for 3-D Model. Images of Col-0 captured every 10 min for 5 days from the side view for the 3-D CG model. Table S2 lists the images used as key frames in the model. [file 13007_2015_75_MOESM19_ESM.zip › side_view/side3_0039.jpg]

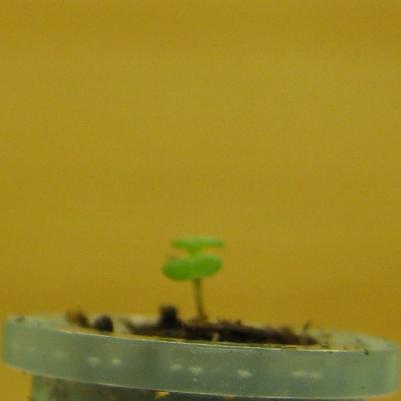

Supplement: Additional file 19 — Col-0 Side View Images for 3-D Model. Images of Col-0 captured every 10 min for 5 days from the side view for the 3-D CG model. Table S2 lists the images used as key frames in the model. [file 13007_2015_75_MOESM19_ESM.zip › side_view/side3_0040.jpg]

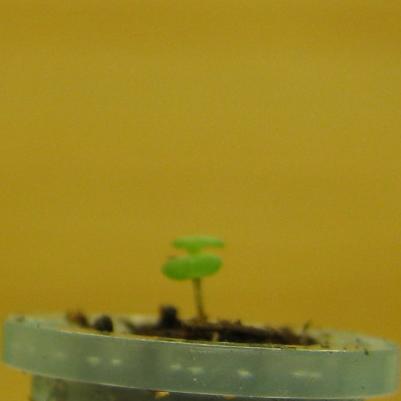

Supplement: Additional file 19 — Col-0 Side View Images for 3-D Model. Images of Col-0 captured every 10 min for 5 days from the side view for the 3-D CG model. Table S2 lists the images used as key frames in the model. [file 13007_2015_75_MOESM19_ESM.zip › side_view/side3_0041.jpg]

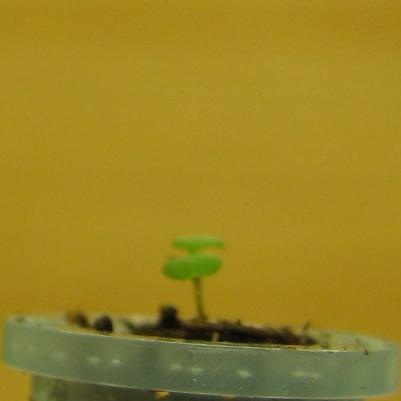

Supplement: Additional file 19 — Col-0 Side View Images for 3-D Model. Images of Col-0 captured every 10 min for 5 days from the side view for the 3-D CG model. Table S2 lists the images used as key frames in the model. [file 13007_2015_75_MOESM19_ESM.zip › side_view/side3_0042.jpg]

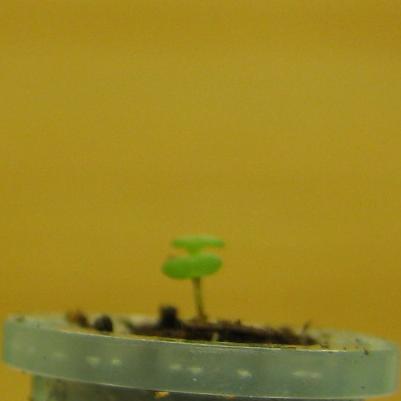

Supplement: Additional file 19 — Col-0 Side View Images for 3-D Model. Images of Col-0 captured every 10 min for 5 days from the side view for the 3-D CG model. Table S2 lists the images used as key frames in the model. [file 13007_2015_75_MOESM19_ESM.zip › side_view/side3_0043.jpg]

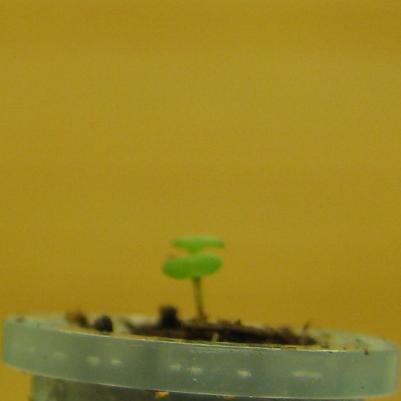

Supplement: Additional file 19 — Col-0 Side View Images for 3-D Model. Images of Col-0 captured every 10 min for 5 days from the side view for the 3-D CG model. Table S2 lists the images used as key frames in the model. [file 13007_2015_75_MOESM19_ESM.zip › side_view/side3_0044.jpg]

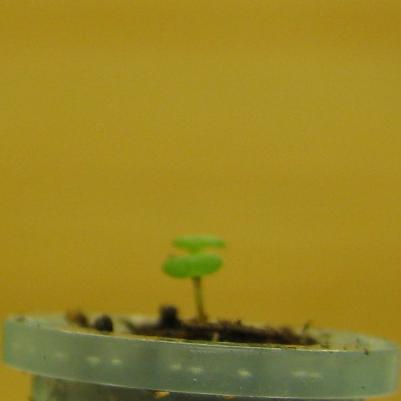

Supplement: Additional file 19 — Col-0 Side View Images for 3-D Model. Images of Col-0 captured every 10 min for 5 days from the side view for the 3-D CG model. Table S2 lists the images used as key frames in the model. [file 13007_2015_75_MOESM19_ESM.zip › side_view/side3_0045.jpg]

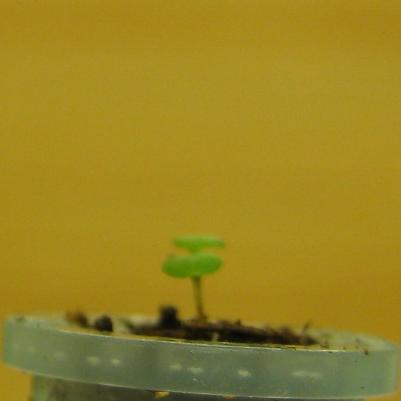

Supplement: Additional file 19 — Col-0 Side View Images for 3-D Model. Images of Col-0 captured every 10 min for 5 days from the side view for the 3-D CG model. Table S2 lists the images used as key frames in the model. [file 13007_2015_75_MOESM19_ESM.zip › side_view/side3_0046.jpg]

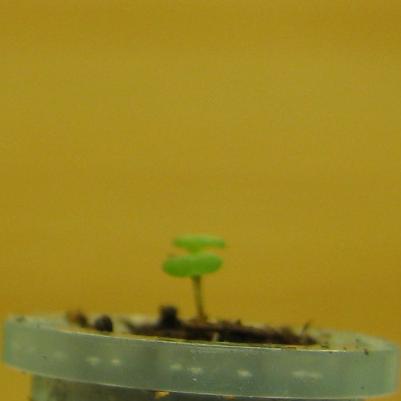

Supplement: Additional file 19 — Col-0 Side View Images for 3-D Model. Images of Col-0 captured every 10 min for 5 days from the side view for the 3-D CG model. Table S2 lists the images used as key frames in the model. [file 13007_2015_75_MOESM19_ESM.zip › side_view/side3_0047.jpg]

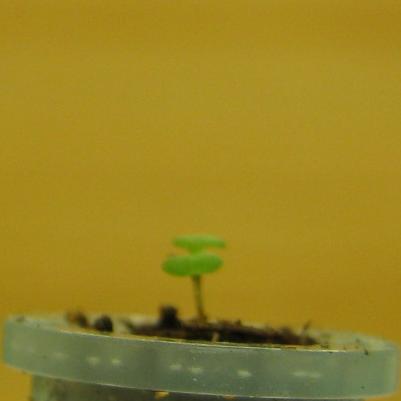

Supplement: Additional file 19 — Col-0 Side View Images for 3-D Model. Images of Col-0 captured every 10 min for 5 days from the side view for the 3-D CG model. Table S2 lists the images used as key frames in the model. [file 13007_2015_75_MOESM19_ESM.zip › side_view/side3_0048.jpg]

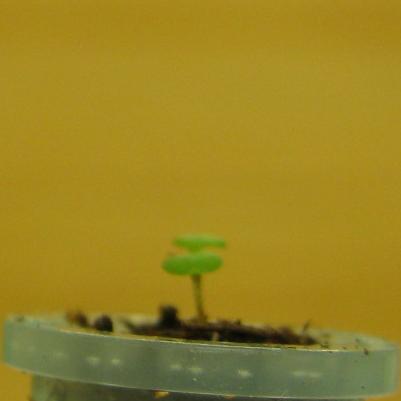

Supplement: Additional file 19 — Col-0 Side View Images for 3-D Model. Images of Col-0 captured every 10 min for 5 days from the side view for the 3-D CG model. Table S2 lists the images used as key frames in the model. [file 13007_2015_75_MOESM19_ESM.zip › side_view/side3_0049.jpg]

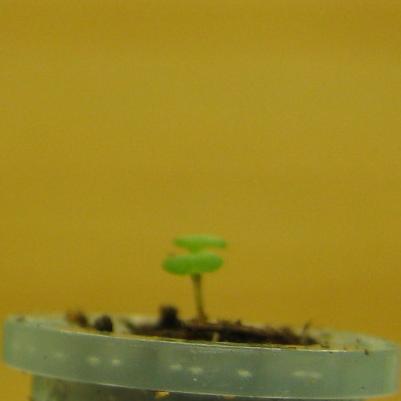

Supplement: Additional file 19 — Col-0 Side View Images for 3-D Model. Images of Col-0 captured every 10 min for 5 days from the side view for the 3-D CG model. Table S2 lists the images used as key frames in the model. [file 13007_2015_75_MOESM19_ESM.zip › side_view/side3_0050.jpg]

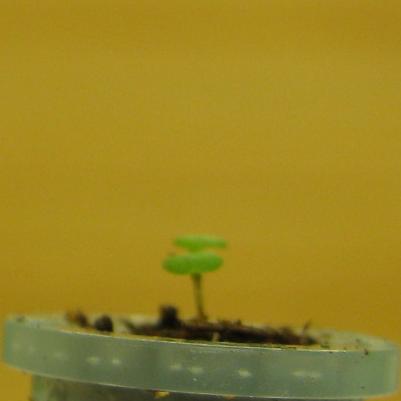

Supplement: Additional file 19 — Col-0 Side View Images for 3-D Model. Images of Col-0 captured every 10 min for 5 days from the side view for the 3-D CG model. Table S2 lists the images used as key frames in the model. [file 13007_2015_75_MOESM19_ESM.zip › side_view/side3_0051.jpg]

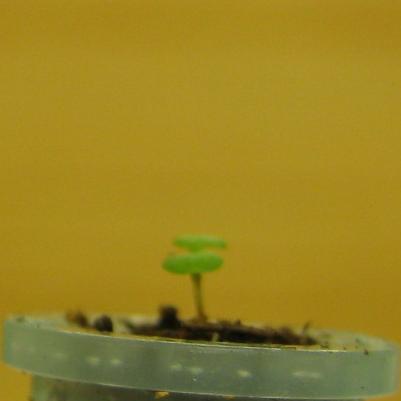

Supplement: Additional file 19 — Col-0 Side View Images for 3-D Model. Images of Col-0 captured every 10 min for 5 days from the side view for the 3-D CG model. Table S2 lists the images used as key frames in the model. [file 13007_2015_75_MOESM19_ESM.zip › side_view/side3_0052.jpg]

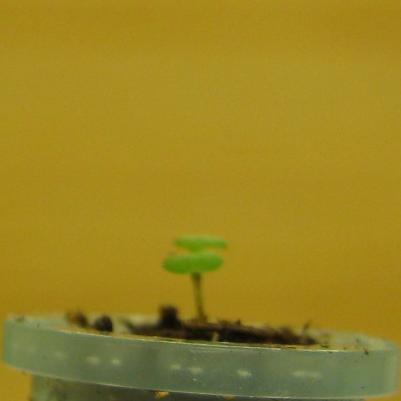

Supplement: Additional file 19 — Col-0 Side View Images for 3-D Model. Images of Col-0 captured every 10 min for 5 days from the side view for the 3-D CG model. Table S2 lists the images used as key frames in the model. [file 13007_2015_75_MOESM19_ESM.zip › side_view/side3_0053.jpg]

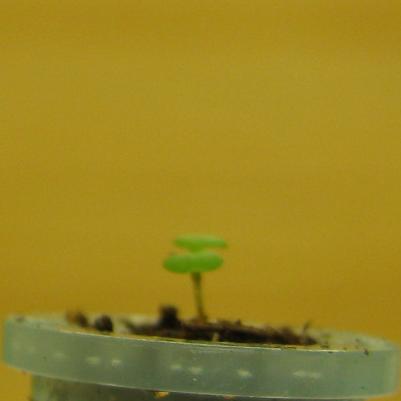

Supplement: Additional file 19 — Col-0 Side View Images for 3-D Model. Images of Col-0 captured every 10 min for 5 days from the side view for the 3-D CG model. Table S2 lists the images used as key frames in the model. [file 13007_2015_75_MOESM19_ESM.zip › side_view/side3_0054.jpg]

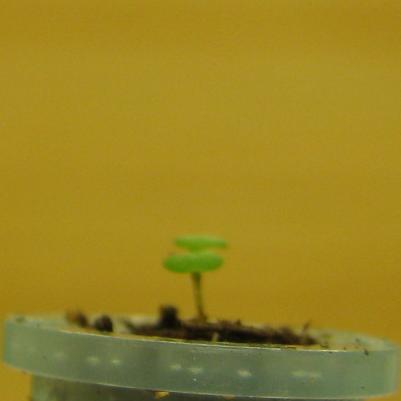

Supplement: Additional file 19 — Col-0 Side View Images for 3-D Model. Images of Col-0 captured every 10 min for 5 days from the side view for the 3-D CG model. Table S2 lists the images used as key frames in the model. [file 13007_2015_75_MOESM19_ESM.zip › side_view/side3_0055.jpg]

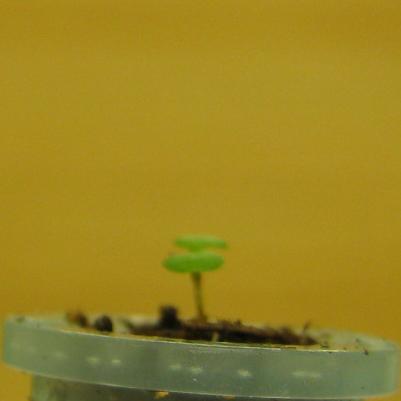

Supplement: Additional file 19 — Col-0 Side View Images for 3-D Model. Images of Col-0 captured every 10 min for 5 days from the side view for the 3-D CG model. Table S2 lists the images used as key frames in the model. [file 13007_2015_75_MOESM19_ESM.zip › side_view/side3_0056.jpg]

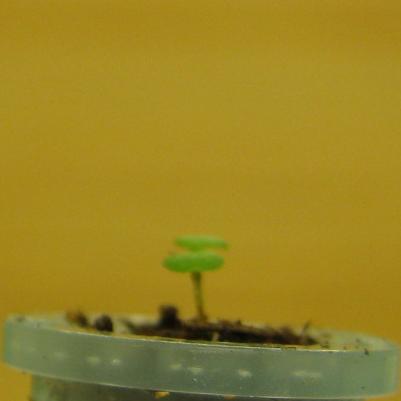

Supplement: Additional file 19 — Col-0 Side View Images for 3-D Model. Images of Col-0 captured every 10 min for 5 days from the side view for the 3-D CG model. Table S2 lists the images used as key frames in the model. [file 13007_2015_75_MOESM19_ESM.zip › side_view/side3_0057.jpg]

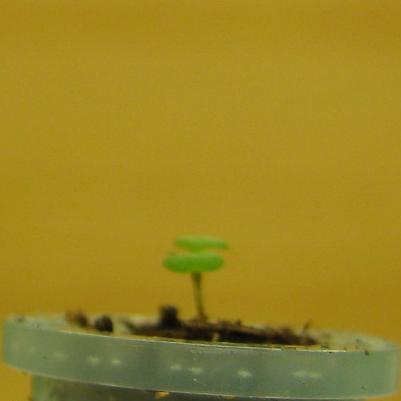

Supplement: Additional file 19 — Col-0 Side View Images for 3-D Model. Images of Col-0 captured every 10 min for 5 days from the side view for the 3-D CG model. Table S2 lists the images used as key frames in the model. [file 13007_2015_75_MOESM19_ESM.zip › side_view/side3_0058.jpg]

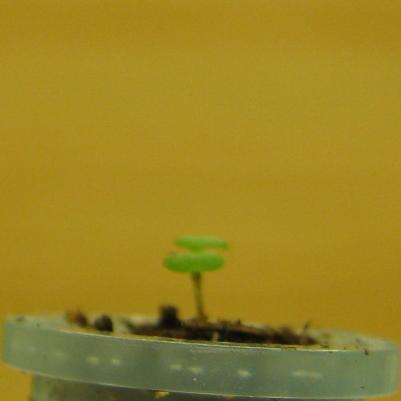

Supplement: Additional file 19 — Col-0 Side View Images for 3-D Model. Images of Col-0 captured every 10 min for 5 days from the side view for the 3-D CG model. Table S2 lists the images used as key frames in the model. [file 13007_2015_75_MOESM19_ESM.zip › side_view/side3_0059.jpg]

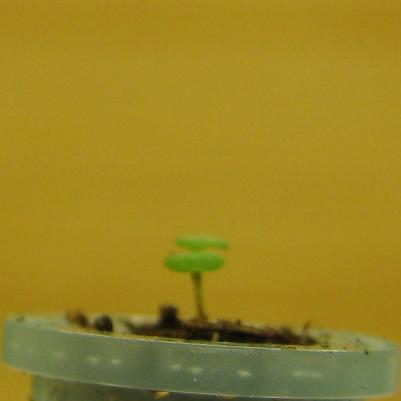

Supplement: Additional file 19 — Col-0 Side View Images for 3-D Model. Images of Col-0 captured every 10 min for 5 days from the side view for the 3-D CG model. Table S2 lists the images used as key frames in the model. [file 13007_2015_75_MOESM19_ESM.zip › side_view/side3_0060.jpg]

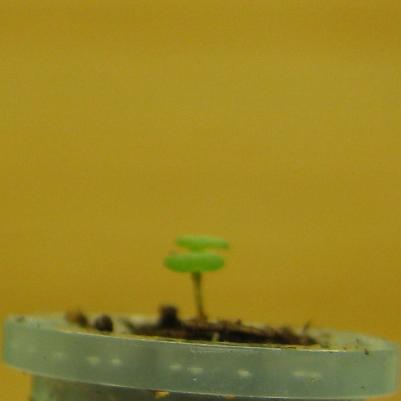

Supplement: Additional file 19 — Col-0 Side View Images for 3-D Model. Images of Col-0 captured every 10 min for 5 days from the side view for the 3-D CG model. Table S2 lists the images used as key frames in the model. [file 13007_2015_75_MOESM19_ESM.zip › side_view/side3_0061.jpg]

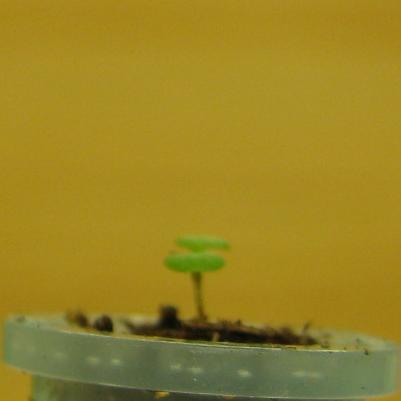

Supplement: Additional file 19 — Col-0 Side View Images for 3-D Model. Images of Col-0 captured every 10 min for 5 days from the side view for the 3-D CG model. Table S2 lists the images used as key frames in the model. [file 13007_2015_75_MOESM19_ESM.zip › side_view/side3_0062.jpg]

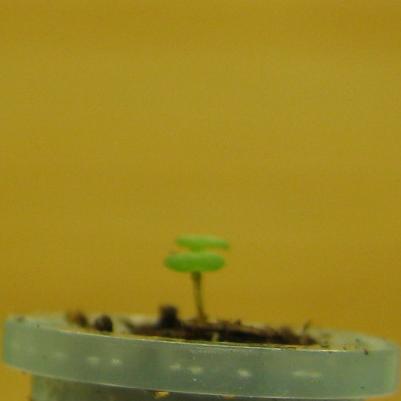

Supplement: Additional file 19 — Col-0 Side View Images for 3-D Model. Images of Col-0 captured every 10 min for 5 days from the side view for the 3-D CG model. Table S2 lists the images used as key frames in the model. [file 13007_2015_75_MOESM19_ESM.zip › side_view/side3_0063.jpg]

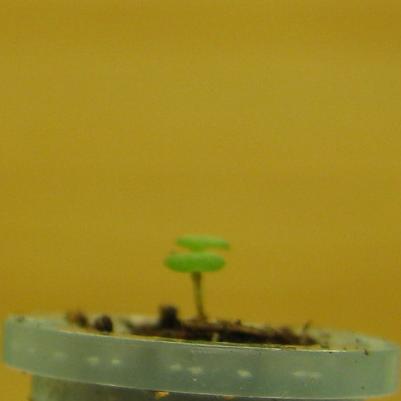

Supplement: Additional file 19 — Col-0 Side View Images for 3-D Model. Images of Col-0 captured every 10 min for 5 days from the side view for the 3-D CG model. Table S2 lists the images used as key frames in the model. [file 13007_2015_75_MOESM19_ESM.zip › side_view/side3_0064.jpg]

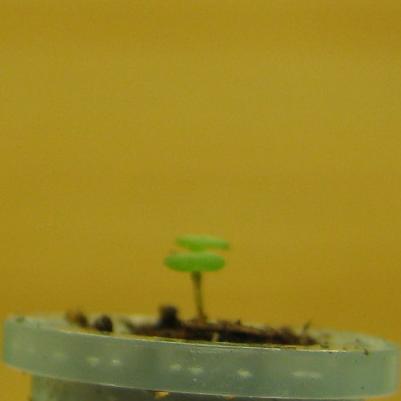

Supplement: Additional file 19 — Col-0 Side View Images for 3-D Model. Images of Col-0 captured every 10 min for 5 days from the side view for the 3-D CG model. Table S2 lists the images used as key frames in the model. [file 13007_2015_75_MOESM19_ESM.zip › side_view/side3_0065.jpg]

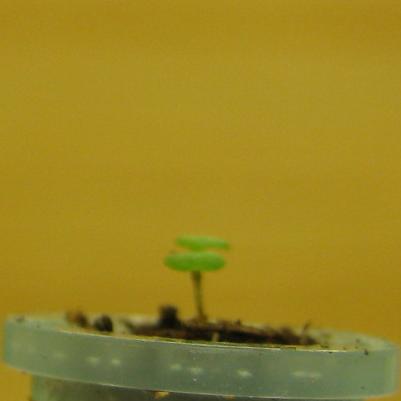

Supplement: Additional file 19 — Col-0 Side View Images for 3-D Model. Images of Col-0 captured every 10 min for 5 days from the side view for the 3-D CG model. Table S2 lists the images used as key frames in the model. [file 13007_2015_75_MOESM19_ESM.zip › side_view/side3_0066.jpg]

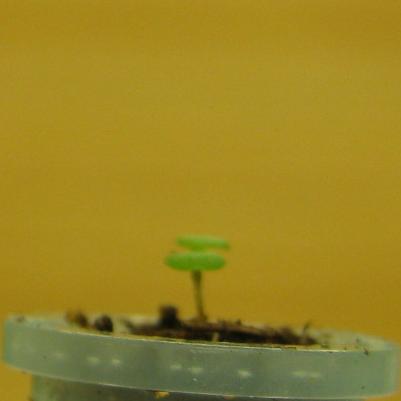

Supplement: Additional file 19 — Col-0 Side View Images for 3-D Model. Images of Col-0 captured every 10 min for 5 days from the side view for the 3-D CG model. Table S2 lists the images used as key frames in the model. [file 13007_2015_75_MOESM19_ESM.zip › side_view/side3_0067.jpg]

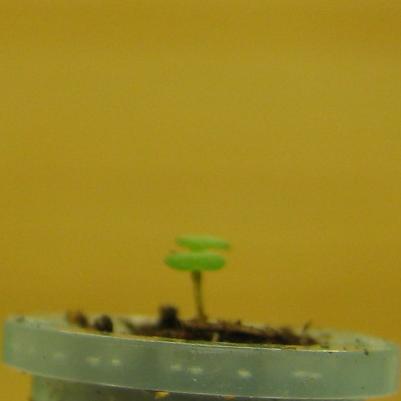

Supplement: Additional file 19 — Col-0 Side View Images for 3-D Model. Images of Col-0 captured every 10 min for 5 days from the side view for the 3-D CG model. Table S2 lists the images used as key frames in the model. [file 13007_2015_75_MOESM19_ESM.zip › side_view/side3_0068.jpg]

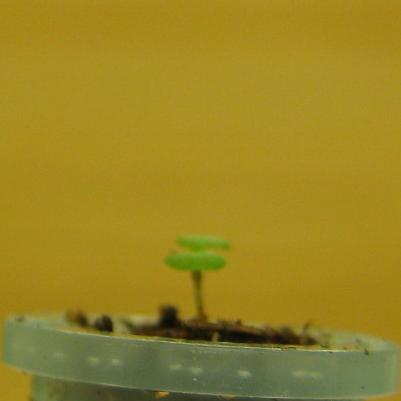

Supplement: Additional file 19 — Col-0 Side View Images for 3-D Model. Images of Col-0 captured every 10 min for 5 days from the side view for the 3-D CG model. Table S2 lists the images used as key frames in the model. [file 13007_2015_75_MOESM19_ESM.zip › side_view/side3_0069.jpg]

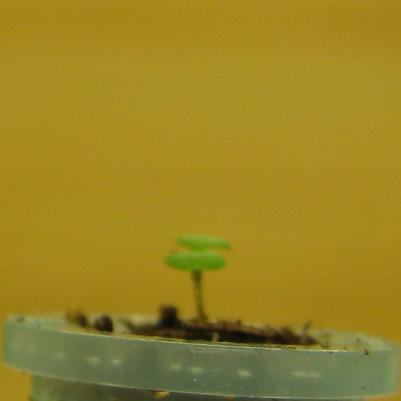

Supplement: Additional file 19 — Col-0 Side View Images for 3-D Model. Images of Col-0 captured every 10 min for 5 days from the side view for the 3-D CG model. Table S2 lists the images used as key frames in the model. [file 13007_2015_75_MOESM19_ESM.zip › side_view/side3_0070.jpg]

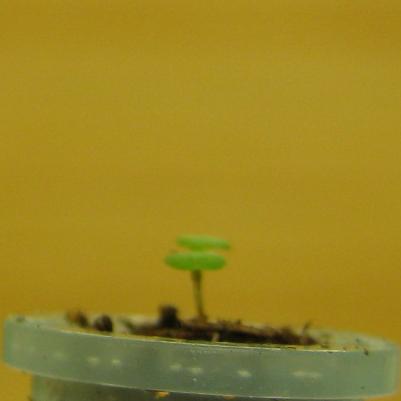

Supplement: Additional file 19 — Col-0 Side View Images for 3-D Model. Images of Col-0 captured every 10 min for 5 days from the side view for the 3-D CG model. Table S2 lists the images used as key frames in the model. [file 13007_2015_75_MOESM19_ESM.zip › side_view/side3_0071.jpg]

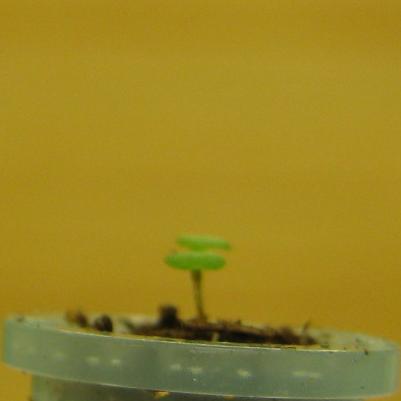

Supplement: Additional file 19 — Col-0 Side View Images for 3-D Model. Images of Col-0 captured every 10 min for 5 days from the side view for the 3-D CG model. Table S2 lists the images used as key frames in the model. [file 13007_2015_75_MOESM19_ESM.zip › side_view/side3_0072.jpg]

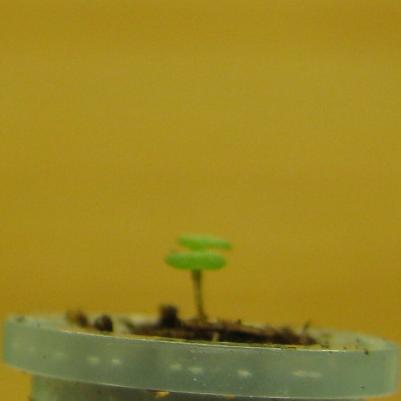

Supplement: Additional file 19 — Col-0 Side View Images for 3-D Model. Images of Col-0 captured every 10 min for 5 days from the side view for the 3-D CG model. Table S2 lists the images used as key frames in the model. [file 13007_2015_75_MOESM19_ESM.zip › side_view/side3_0073.jpg]

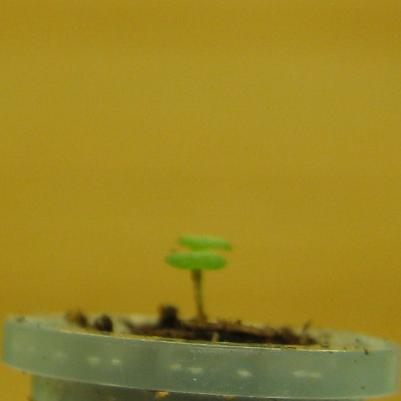

Supplement: Additional file 19 — Col-0 Side View Images for 3-D Model. Images of Col-0 captured every 10 min for 5 days from the side view for the 3-D CG model. Table S2 lists the images used as key frames in the model. [file 13007_2015_75_MOESM19_ESM.zip › side_view/side3_0074.jpg]

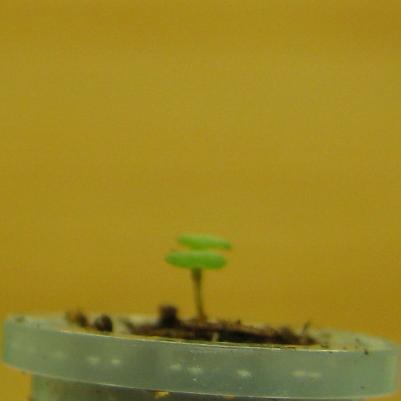

Supplement: Additional file 19 — Col-0 Side View Images for 3-D Model. Images of Col-0 captured every 10 min for 5 days from the side view for the 3-D CG model. Table S2 lists the images used as key frames in the model. [file 13007_2015_75_MOESM19_ESM.zip › side_view/side3_0075.jpg]

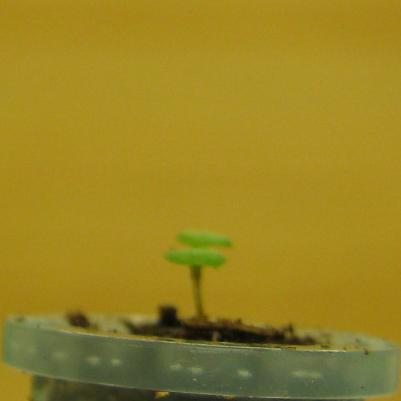

Supplement: Additional file 19 — Col-0 Side View Images for 3-D Model. Images of Col-0 captured every 10 min for 5 days from the side view for the 3-D CG model. Table S2 lists the images used as key frames in the model. [file 13007_2015_75_MOESM19_ESM.zip › side_view/side3_0076.jpg]

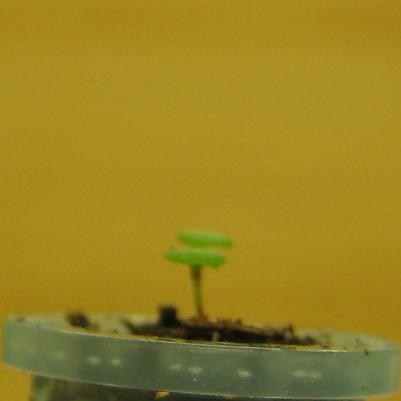

Supplement: Additional file 19 — Col-0 Side View Images for 3-D Model. Images of Col-0 captured every 10 min for 5 days from the side view for the 3-D CG model. Table S2 lists the images used as key frames in the model. [file 13007_2015_75_MOESM19_ESM.zip › side_view/side3_0077.jpg]

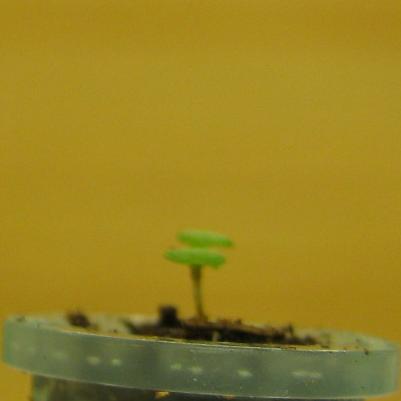

Supplement: Additional file 19 — Col-0 Side View Images for 3-D Model. Images of Col-0 captured every 10 min for 5 days from the side view for the 3-D CG model. Table S2 lists the images used as key frames in the model. [file 13007_2015_75_MOESM19_ESM.zip › side_view/side3_0078.jpg]

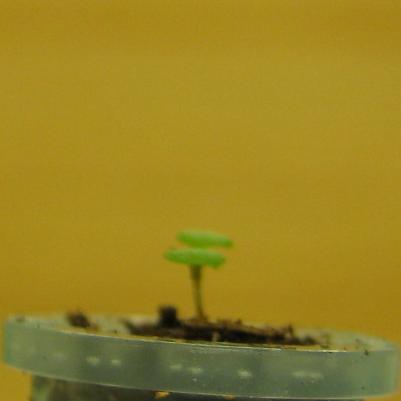

Supplement: Additional file 19 — Col-0 Side View Images for 3-D Model. Images of Col-0 captured every 10 min for 5 days from the side view for the 3-D CG model. Table S2 lists the images used as key frames in the model. [file 13007_2015_75_MOESM19_ESM.zip › side_view/side3_0079.jpg]

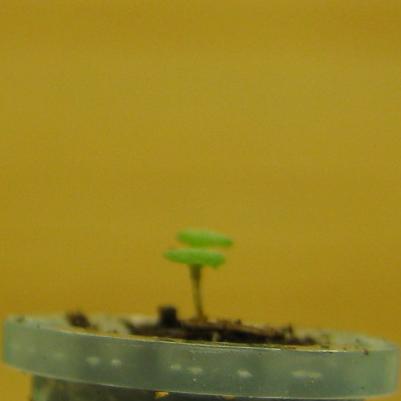

Supplement: Additional file 19 — Col-0 Side View Images for 3-D Model. Images of Col-0 captured every 10 min for 5 days from the side view for the 3-D CG model. Table S2 lists the images used as key frames in the model. [file 13007_2015_75_MOESM19_ESM.zip › side_view/side3_0080.jpg]

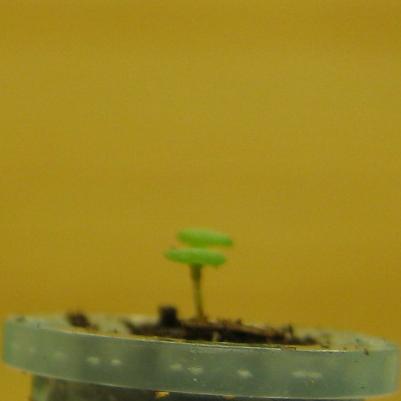

Supplement: Additional file 19 — Col-0 Side View Images for 3-D Model. Images of Col-0 captured every 10 min for 5 days from the side view for the 3-D CG model. Table S2 lists the images used as key frames in the model. [file 13007_2015_75_MOESM19_ESM.zip › side_view/side3_0081.jpg]

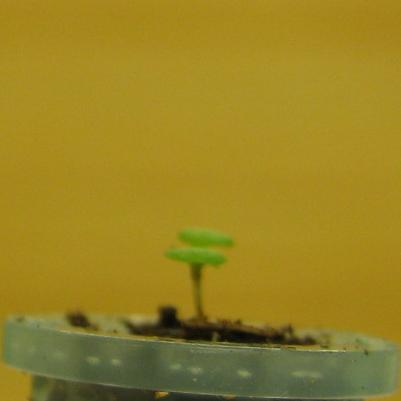

Supplement: Additional file 19 — Col-0 Side View Images for 3-D Model. Images of Col-0 captured every 10 min for 5 days from the side view for the 3-D CG model. Table S2 lists the images used as key frames in the model. [file 13007_2015_75_MOESM19_ESM.zip › side_view/side3_0082.jpg]

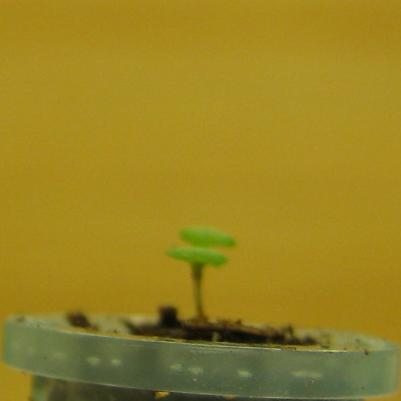

Supplement: Additional file 19 — Col-0 Side View Images for 3-D Model. Images of Col-0 captured every 10 min for 5 days from the side view for the 3-D CG model. Table S2 lists the images used as key frames in the model. [file 13007_2015_75_MOESM19_ESM.zip › side_view/side3_0083.jpg]

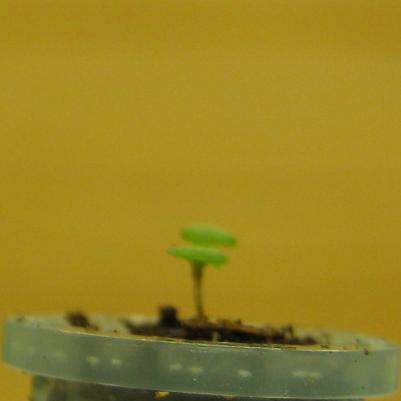

Supplement: Additional file 19 — Col-0 Side View Images for 3-D Model. Images of Col-0 captured every 10 min for 5 days from the side view for the 3-D CG model. Table S2 lists the images used as key frames in the model. [file 13007_2015_75_MOESM19_ESM.zip › side_view/side3_0084.jpg]

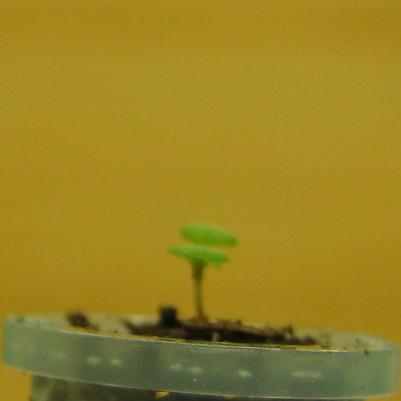

Supplement: Additional file 19 — Col-0 Side View Images for 3-D Model. Images of Col-0 captured every 10 min for 5 days from the side view for the 3-D CG model. Table S2 lists the images used as key frames in the model. [file 13007_2015_75_MOESM19_ESM.zip › side_view/side3_0085.jpg]

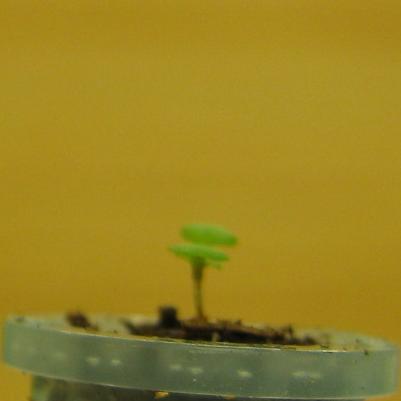

Supplement: Additional file 19 — Col-0 Side View Images for 3-D Model. Images of Col-0 captured every 10 min for 5 days from the side view for the 3-D CG model. Table S2 lists the images used as key frames in the model. [file 13007_2015_75_MOESM19_ESM.zip › side_view/side3_0086.jpg]

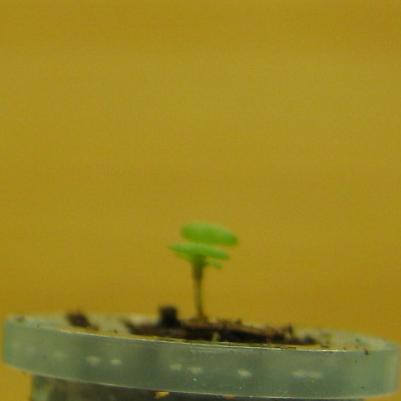

Supplement: Additional file 19 — Col-0 Side View Images for 3-D Model. Images of Col-0 captured every 10 min for 5 days from the side view for the 3-D CG model. Table S2 lists the images used as key frames in the model. [file 13007_2015_75_MOESM19_ESM.zip › side_view/side3_0087.jpg]

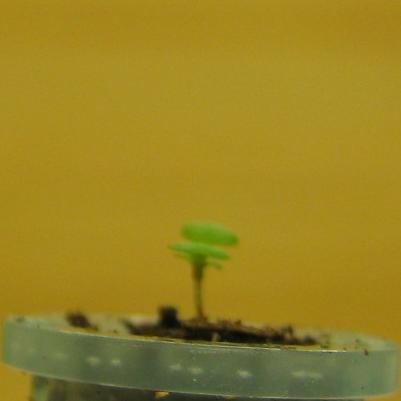

Supplement: Additional file 19 — Col-0 Side View Images for 3-D Model. Images of Col-0 captured every 10 min for 5 days from the side view for the 3-D CG model. Table S2 lists the images used as key frames in the model. [file 13007_2015_75_MOESM19_ESM.zip › side_view/side3_0088.jpg]

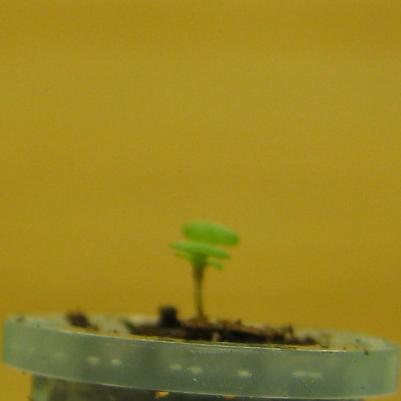

Supplement: Additional file 19 — Col-0 Side View Images for 3-D Model. Images of Col-0 captured every 10 min for 5 days from the side view for the 3-D CG model. Table S2 lists the images used as key frames in the model. [file 13007_2015_75_MOESM19_ESM.zip › side_view/side3_0089.jpg]

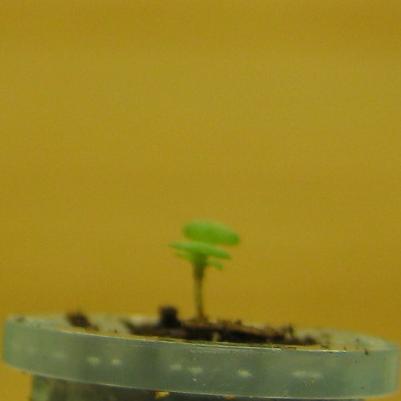

Supplement: Additional file 19 — Col-0 Side View Images for 3-D Model. Images of Col-0 captured every 10 min for 5 days from the side view for the 3-D CG model. Table S2 lists the images used as key frames in the model. [file 13007_2015_75_MOESM19_ESM.zip › side_view/side3_0090.jpg]

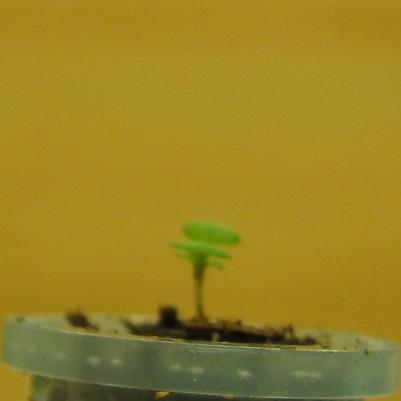

Supplement: Additional file 19 — Col-0 Side View Images for 3-D Model. Images of Col-0 captured every 10 min for 5 days from the side view for the 3-D CG model. Table S2 lists the images used as key frames in the model. [file 13007_2015_75_MOESM19_ESM.zip › side_view/side3_0091.jpg]

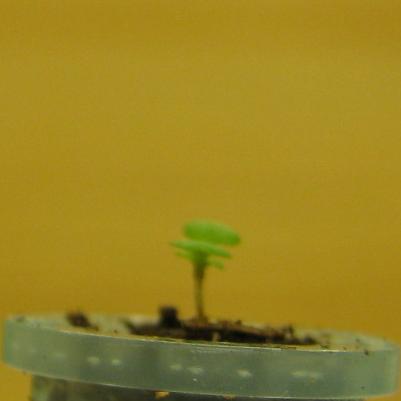

Supplement: Additional file 19 — Col-0 Side View Images for 3-D Model. Images of Col-0 captured every 10 min for 5 days from the side view for the 3-D CG model. Table S2 lists the images used as key frames in the model. [file 13007_2015_75_MOESM19_ESM.zip › side_view/side3_0092.jpg]

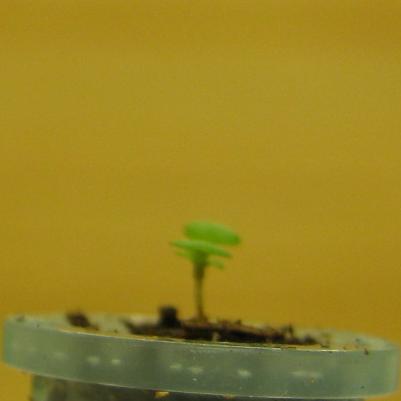

Supplement: Additional file 19 — Col-0 Side View Images for 3-D Model. Images of Col-0 captured every 10 min for 5 days from the side view for the 3-D CG model. Table S2 lists the images used as key frames in the model. [file 13007_2015_75_MOESM19_ESM.zip › side_view/side3_0093.jpg]

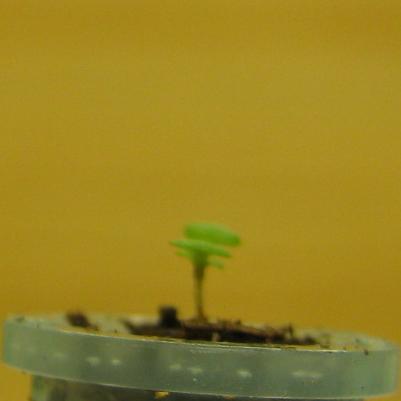

Supplement: Additional file 19 — Col-0 Side View Images for 3-D Model. Images of Col-0 captured every 10 min for 5 days from the side view for the 3-D CG model. Table S2 lists the images used as key frames in the model. [file 13007_2015_75_MOESM19_ESM.zip › side_view/side3_0094.jpg]

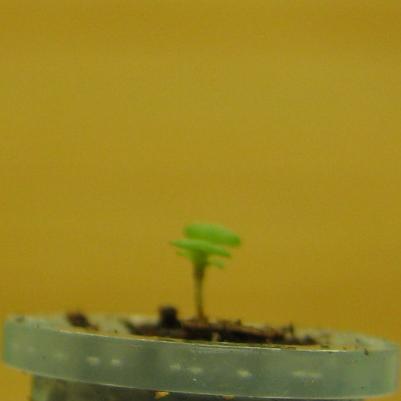

Supplement: Additional file 19 — Col-0 Side View Images for 3-D Model. Images of Col-0 captured every 10 min for 5 days from the side view for the 3-D CG model. Table S2 lists the images used as key frames in the model. [file 13007_2015_75_MOESM19_ESM.zip › side_view/side3_0095.jpg]

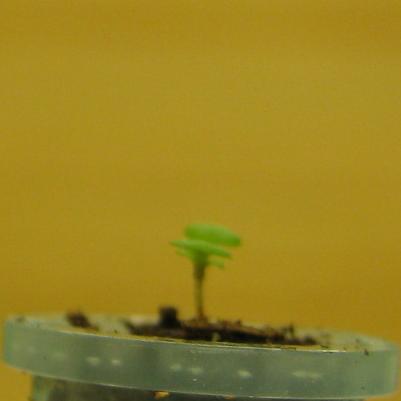

Supplement: Additional file 19 — Col-0 Side View Images for 3-D Model. Images of Col-0 captured every 10 min for 5 days from the side view for the 3-D CG model. Table S2 lists the images used as key frames in the model. [file 13007_2015_75_MOESM19_ESM.zip › side_view/side3_0096.jpg]

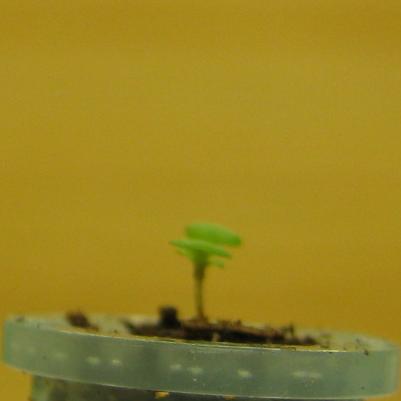

Supplement: Additional file 19 — Col-0 Side View Images for 3-D Model. Images of Col-0 captured every 10 min for 5 days from the side view for the 3-D CG model. Table S2 lists the images used as key frames in the model. [file 13007_2015_75_MOESM19_ESM.zip › side_view/side3_0097.jpg]

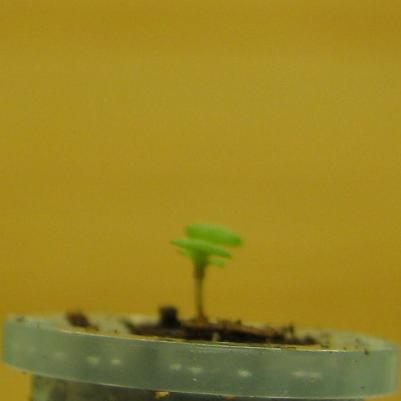

Supplement: Additional file 19 — Col-0 Side View Images for 3-D Model. Images of Col-0 captured every 10 min for 5 days from the side view for the 3-D CG model. Table S2 lists the images used as key frames in the model. [file 13007_2015_75_MOESM19_ESM.zip › side_view/side3_0098.jpg]

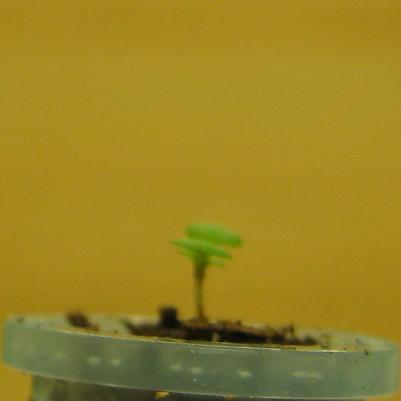

Supplement: Additional file 19 — Col-0 Side View Images for 3-D Model. Images of Col-0 captured every 10 min for 5 days from the side view for the 3-D CG model. Table S2 lists the images used as key frames in the model. [file 13007_2015_75_MOESM19_ESM.zip › side_view/side3_0099.jpg]

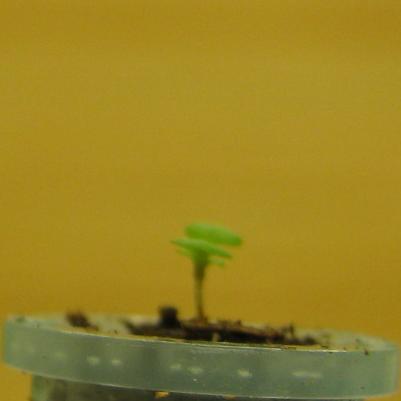

Supplement: Additional file 19 — Col-0 Side View Images for 3-D Model. Images of Col-0 captured every 10 min for 5 days from the side view for the 3-D CG model. Table S2 lists the images used as key frames in the model. [file 13007_2015_75_MOESM19_ESM.zip › side_view/side3_0100.jpg]

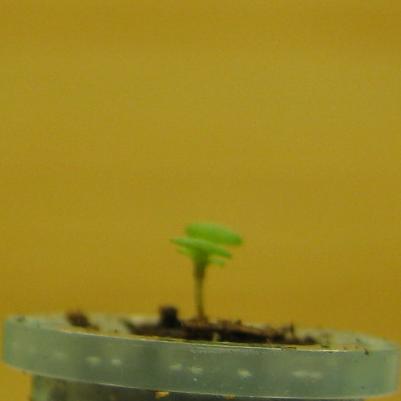

Supplement: Additional file 19 — Col-0 Side View Images for 3-D Model. Images of Col-0 captured every 10 min for 5 days from the side view for the 3-D CG model. Table S2 lists the images used as key frames in the model. [file 13007_2015_75_MOESM19_ESM.zip › side_view/side3_0101.jpg]

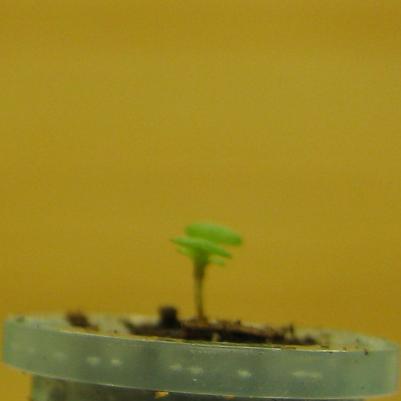

Supplement: Additional file 19 — Col-0 Side View Images for 3-D Model. Images of Col-0 captured every 10 min for 5 days from the side view for the 3-D CG model. Table S2 lists the images used as key frames in the model. [file 13007_2015_75_MOESM19_ESM.zip › side_view/side3_0102.jpg]

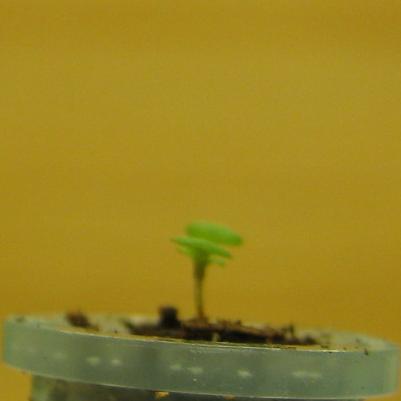

Supplement: Additional file 19 — Col-0 Side View Images for 3-D Model. Images of Col-0 captured every 10 min for 5 days from the side view for the 3-D CG model. Table S2 lists the images used as key frames in the model. [file 13007_2015_75_MOESM19_ESM.zip › side_view/side3_0103.jpg]

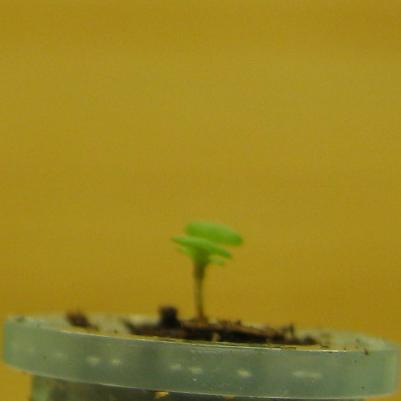

Supplement: Additional file 19 — Col-0 Side View Images for 3-D Model. Images of Col-0 captured every 10 min for 5 days from the side view for the 3-D CG model. Table S2 lists the images used as key frames in the model. [file 13007_2015_75_MOESM19_ESM.zip › side_view/side3_0104.jpg]

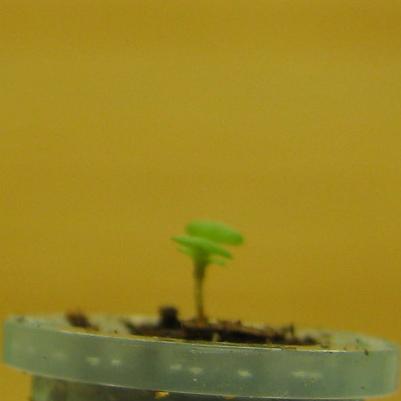

Supplement: Additional file 19 — Col-0 Side View Images for 3-D Model. Images of Col-0 captured every 10 min for 5 days from the side view for the 3-D CG model. Table S2 lists the images used as key frames in the model. [file 13007_2015_75_MOESM19_ESM.zip › side_view/side3_0105.jpg]

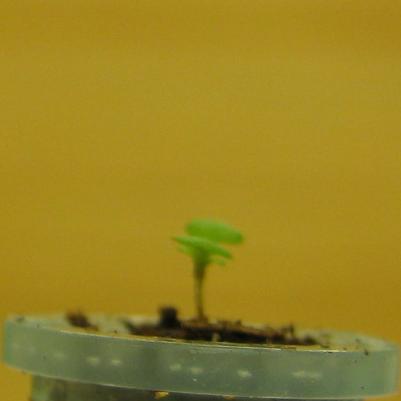

Supplement: Additional file 19 — Col-0 Side View Images for 3-D Model. Images of Col-0 captured every 10 min for 5 days from the side view for the 3-D CG model. Table S2 lists the images used as key frames in the model. [file 13007_2015_75_MOESM19_ESM.zip › side_view/side3_0106.jpg]

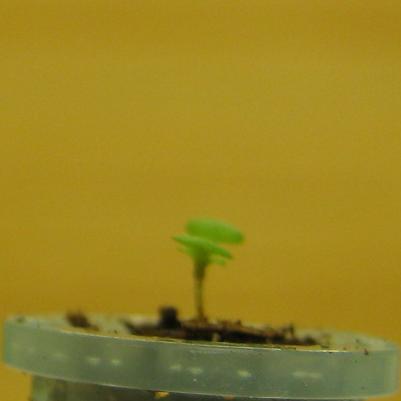

Supplement: Additional file 19 — Col-0 Side View Images for 3-D Model. Images of Col-0 captured every 10 min for 5 days from the side view for the 3-D CG model. Table S2 lists the images used as key frames in the model. [file 13007_2015_75_MOESM19_ESM.zip › side_view/side3_0107.jpg]

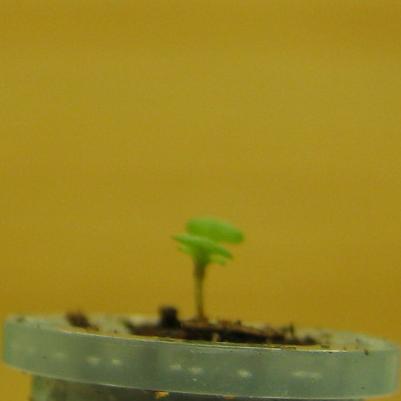

Supplement: Additional file 19 — Col-0 Side View Images for 3-D Model. Images of Col-0 captured every 10 min for 5 days from the side view for the 3-D CG model. Table S2 lists the images used as key frames in the model. [file 13007_2015_75_MOESM19_ESM.zip › side_view/side3_0108.jpg]
