# Supplementary figures and images for: TRiP: Tracking Rhythms in Plants, an automated leaf movement analysis program for circadian period estimation (part 6 of 10)
Source: Plant Methods. 2015 May 3;11:33. doi: 10.1186/s13007-015-0075-5 (PMC4445800; doi:10.1186/s13007-015-0075-5)

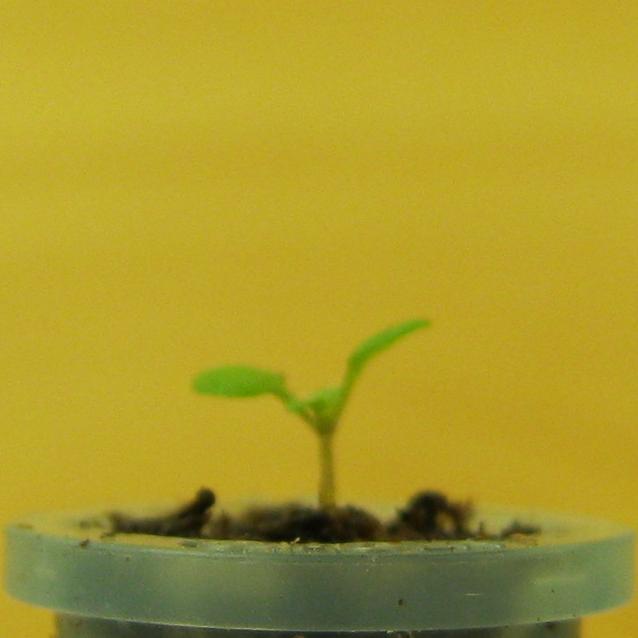

Supplement: Additional file 20 — Col-0 Front View Images for 3-D Model. Images of Col-0 captured every 10 min for 5 days from the front view for the 3-D CG model. Table S2 lists the images used as key frames in the model. [file 13007_2015_75_MOESM20_ESM.zip › front_view/side12_0130.jpg]

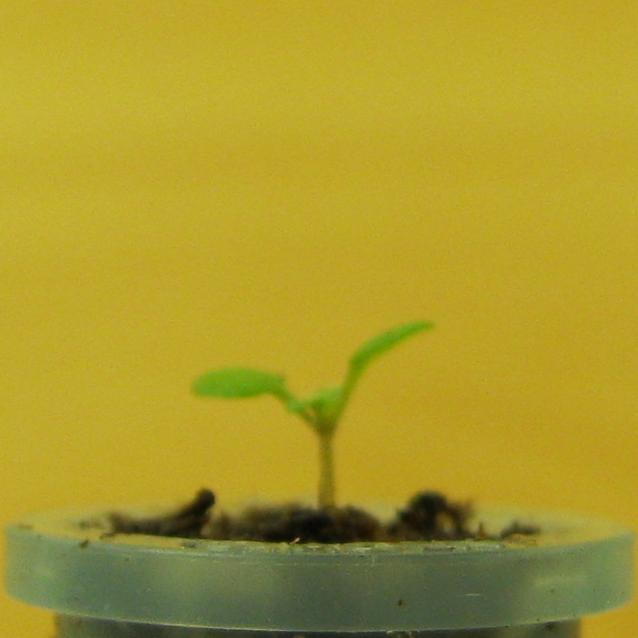

Supplement: Additional file 20 — Col-0 Front View Images for 3-D Model. Images of Col-0 captured every 10 min for 5 days from the front view for the 3-D CG model. Table S2 lists the images used as key frames in the model. [file 13007_2015_75_MOESM20_ESM.zip › front_view/side12_0131.jpg]

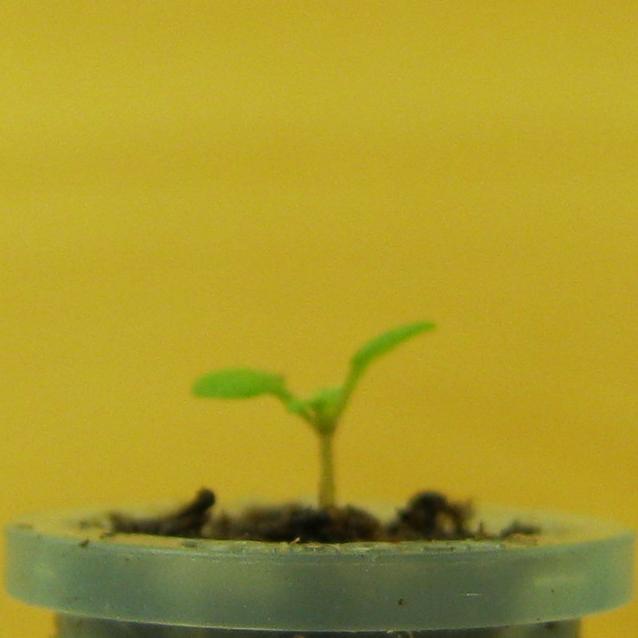

Supplement: Additional file 20 — Col-0 Front View Images for 3-D Model. Images of Col-0 captured every 10 min for 5 days from the front view for the 3-D CG model. Table S2 lists the images used as key frames in the model. [file 13007_2015_75_MOESM20_ESM.zip › front_view/side12_0132.jpg]

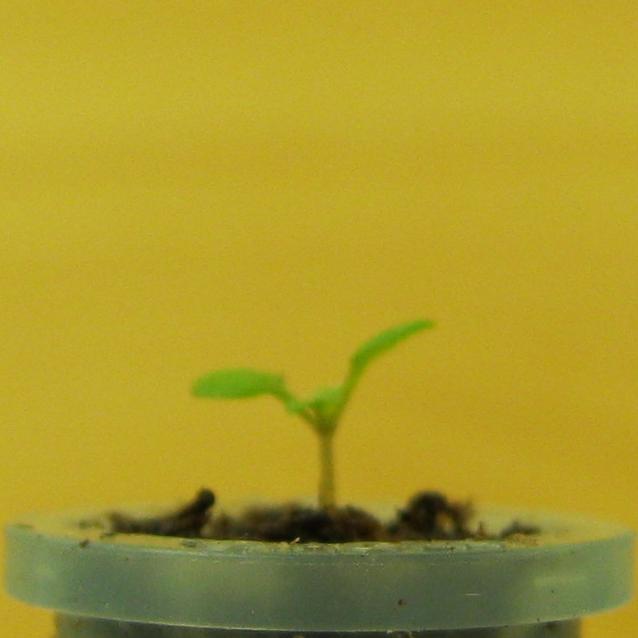

Supplement: Additional file 20 — Col-0 Front View Images for 3-D Model. Images of Col-0 captured every 10 min for 5 days from the front view for the 3-D CG model. Table S2 lists the images used as key frames in the model. [file 13007_2015_75_MOESM20_ESM.zip › front_view/side12_0133.jpg]

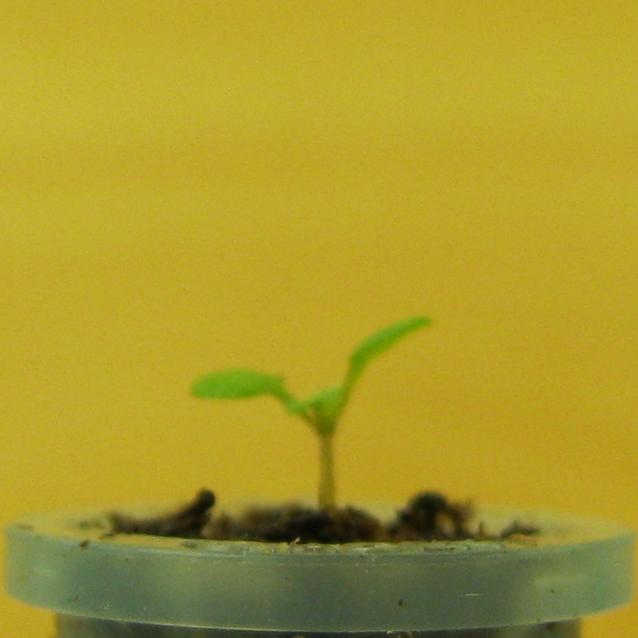

Supplement: Additional file 20 — Col-0 Front View Images for 3-D Model. Images of Col-0 captured every 10 min for 5 days from the front view for the 3-D CG model. Table S2 lists the images used as key frames in the model. [file 13007_2015_75_MOESM20_ESM.zip › front_view/side12_0134.jpg]

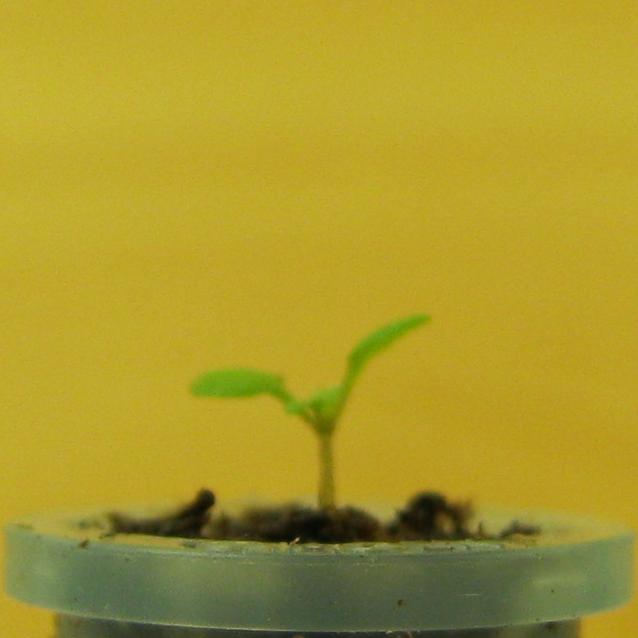

Supplement: Additional file 20 — Col-0 Front View Images for 3-D Model. Images of Col-0 captured every 10 min for 5 days from the front view for the 3-D CG model. Table S2 lists the images used as key frames in the model. [file 13007_2015_75_MOESM20_ESM.zip › front_view/side12_0135.jpg]

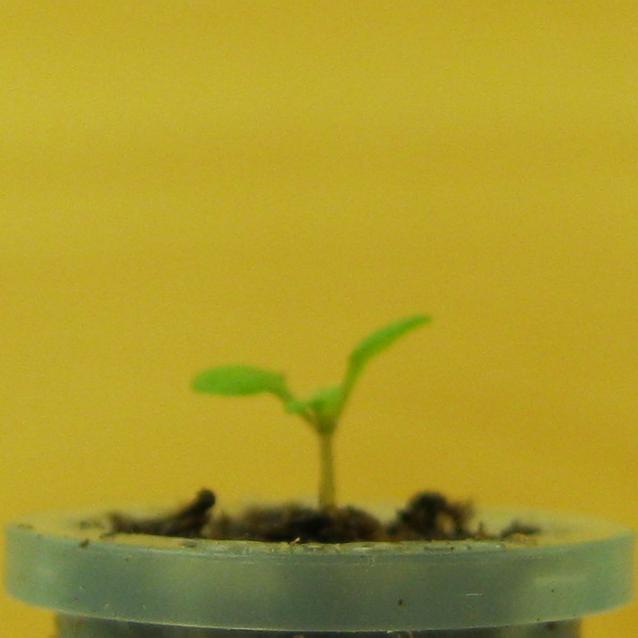

Supplement: Additional file 20 — Col-0 Front View Images for 3-D Model. Images of Col-0 captured every 10 min for 5 days from the front view for the 3-D CG model. Table S2 lists the images used as key frames in the model. [file 13007_2015_75_MOESM20_ESM.zip › front_view/side12_0136.jpg]

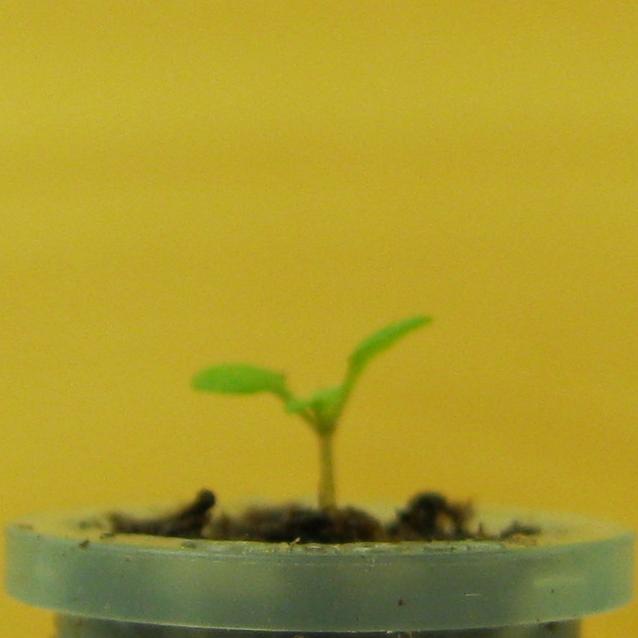

Supplement: Additional file 20 — Col-0 Front View Images for 3-D Model. Images of Col-0 captured every 10 min for 5 days from the front view for the 3-D CG model. Table S2 lists the images used as key frames in the model. [file 13007_2015_75_MOESM20_ESM.zip › front_view/side12_0137.jpg]

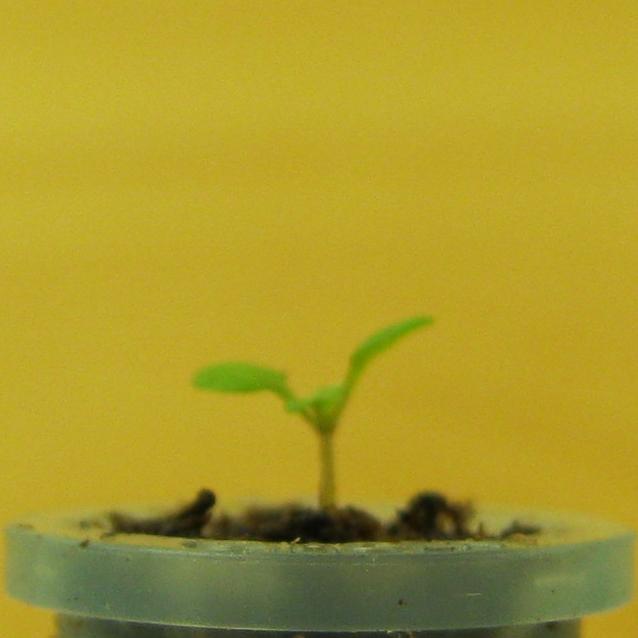

Supplement: Additional file 20 — Col-0 Front View Images for 3-D Model. Images of Col-0 captured every 10 min for 5 days from the front view for the 3-D CG model. Table S2 lists the images used as key frames in the model. [file 13007_2015_75_MOESM20_ESM.zip › front_view/side12_0138.jpg]

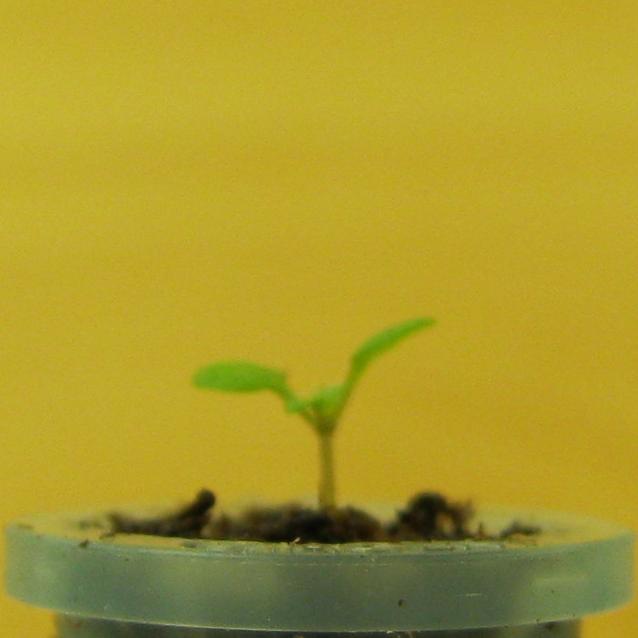

Supplement: Additional file 20 — Col-0 Front View Images for 3-D Model. Images of Col-0 captured every 10 min for 5 days from the front view for the 3-D CG model. Table S2 lists the images used as key frames in the model. [file 13007_2015_75_MOESM20_ESM.zip › front_view/side12_0139.jpg]

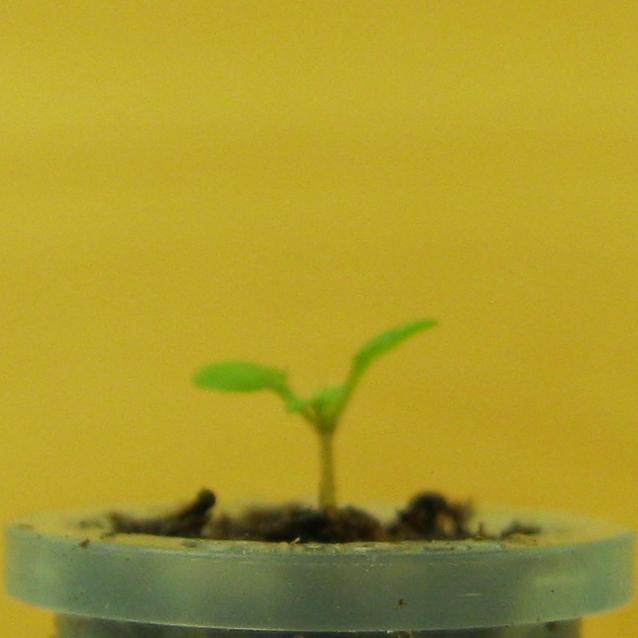

Supplement: Additional file 20 — Col-0 Front View Images for 3-D Model. Images of Col-0 captured every 10 min for 5 days from the front view for the 3-D CG model. Table S2 lists the images used as key frames in the model. [file 13007_2015_75_MOESM20_ESM.zip › front_view/side12_0140.jpg]

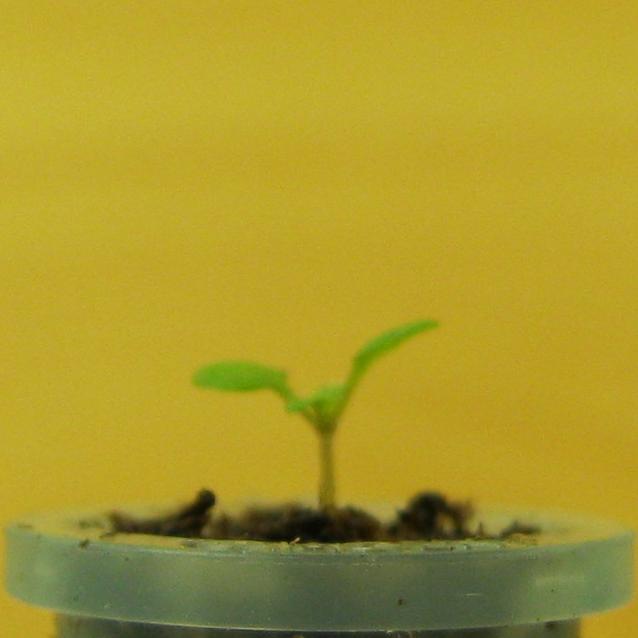

Supplement: Additional file 20 — Col-0 Front View Images for 3-D Model. Images of Col-0 captured every 10 min for 5 days from the front view for the 3-D CG model. Table S2 lists the images used as key frames in the model. [file 13007_2015_75_MOESM20_ESM.zip › front_view/side12_0141.jpg]

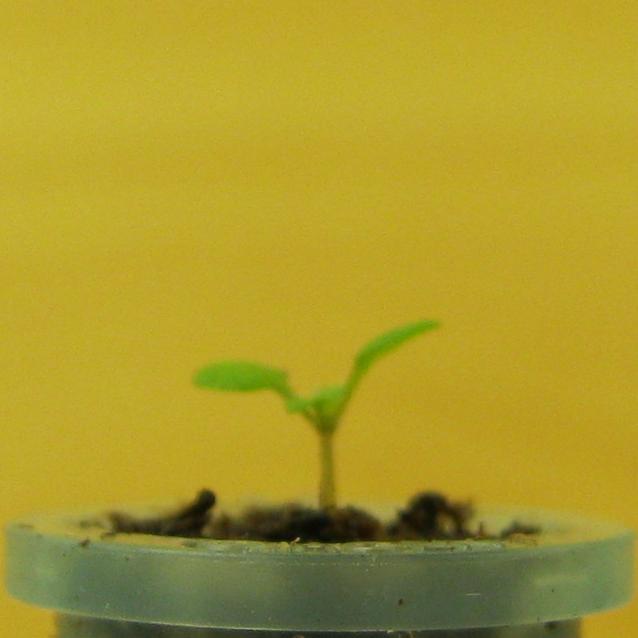

Supplement: Additional file 20 — Col-0 Front View Images for 3-D Model. Images of Col-0 captured every 10 min for 5 days from the front view for the 3-D CG model. Table S2 lists the images used as key frames in the model. [file 13007_2015_75_MOESM20_ESM.zip › front_view/side12_0142.jpg]

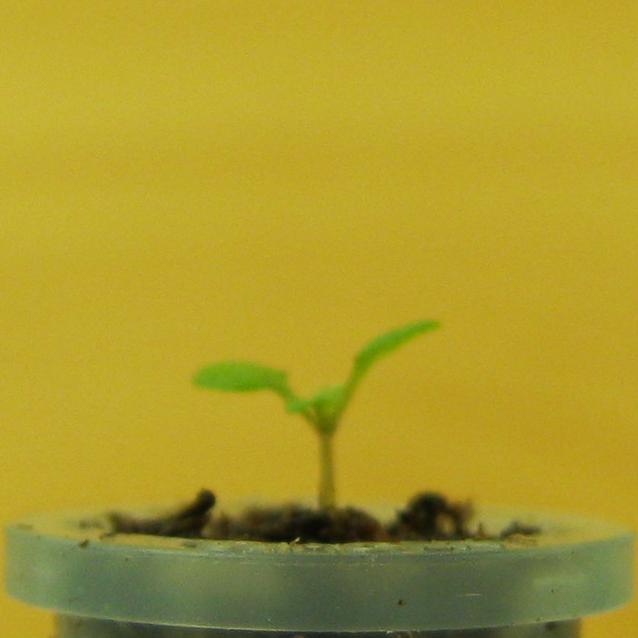

Supplement: Additional file 20 — Col-0 Front View Images for 3-D Model. Images of Col-0 captured every 10 min for 5 days from the front view for the 3-D CG model. Table S2 lists the images used as key frames in the model. [file 13007_2015_75_MOESM20_ESM.zip › front_view/side12_0143.jpg]

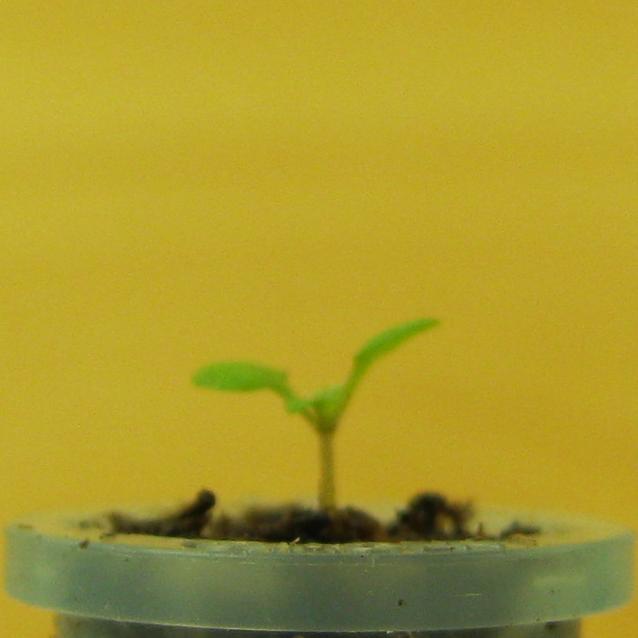

Supplement: Additional file 20 — Col-0 Front View Images for 3-D Model. Images of Col-0 captured every 10 min for 5 days from the front view for the 3-D CG model. Table S2 lists the images used as key frames in the model. [file 13007_2015_75_MOESM20_ESM.zip › front_view/side12_0144.jpg]

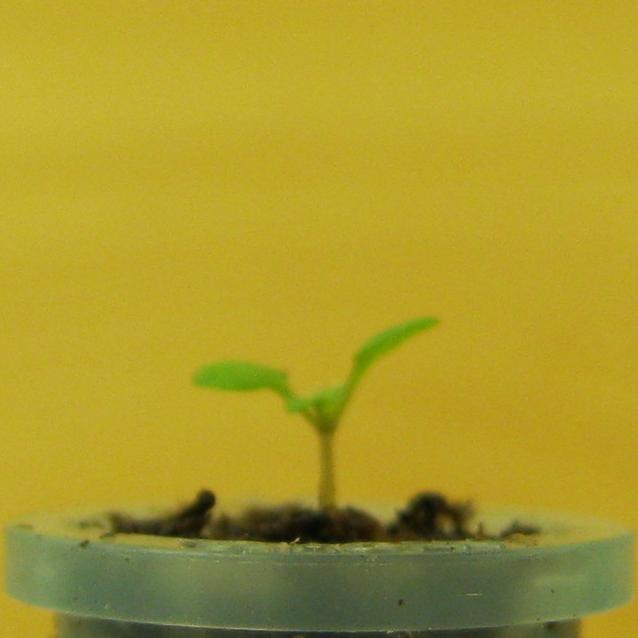

Supplement: Additional file 20 — Col-0 Front View Images for 3-D Model. Images of Col-0 captured every 10 min for 5 days from the front view for the 3-D CG model. Table S2 lists the images used as key frames in the model. [file 13007_2015_75_MOESM20_ESM.zip › front_view/side12_0145.jpg]

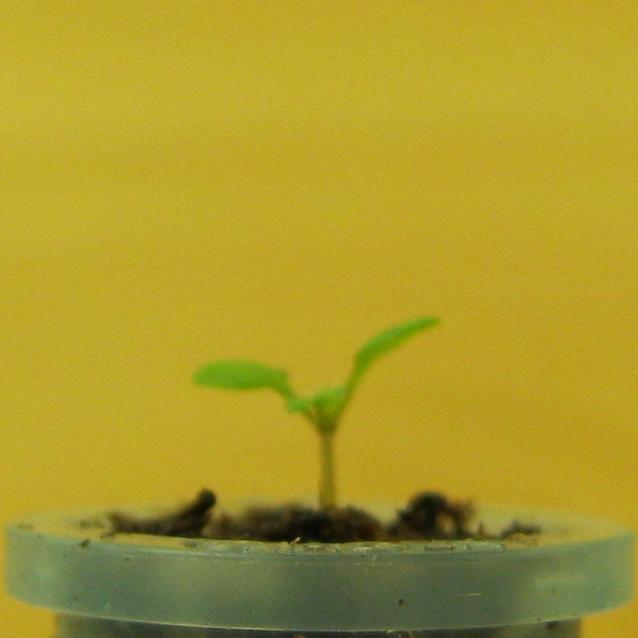

Supplement: Additional file 20 — Col-0 Front View Images for 3-D Model. Images of Col-0 captured every 10 min for 5 days from the front view for the 3-D CG model. Table S2 lists the images used as key frames in the model. [file 13007_2015_75_MOESM20_ESM.zip › front_view/side12_0146.jpg]

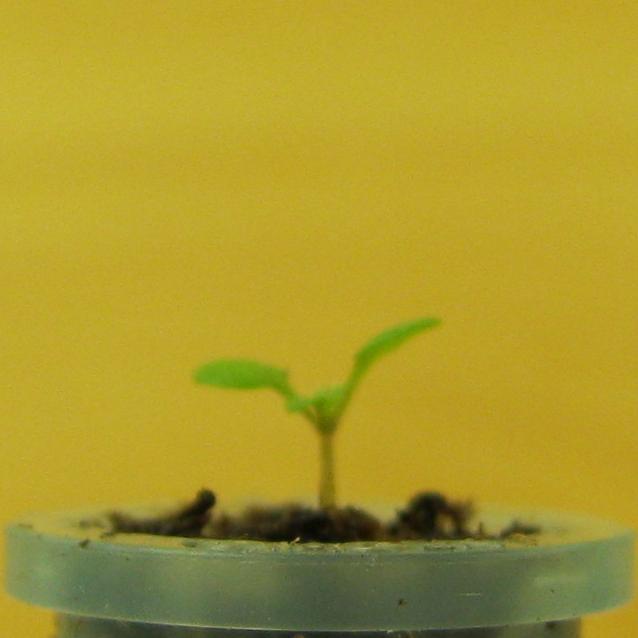

Supplement: Additional file 20 — Col-0 Front View Images for 3-D Model. Images of Col-0 captured every 10 min for 5 days from the front view for the 3-D CG model. Table S2 lists the images used as key frames in the model. [file 13007_2015_75_MOESM20_ESM.zip › front_view/side12_0147.jpg]

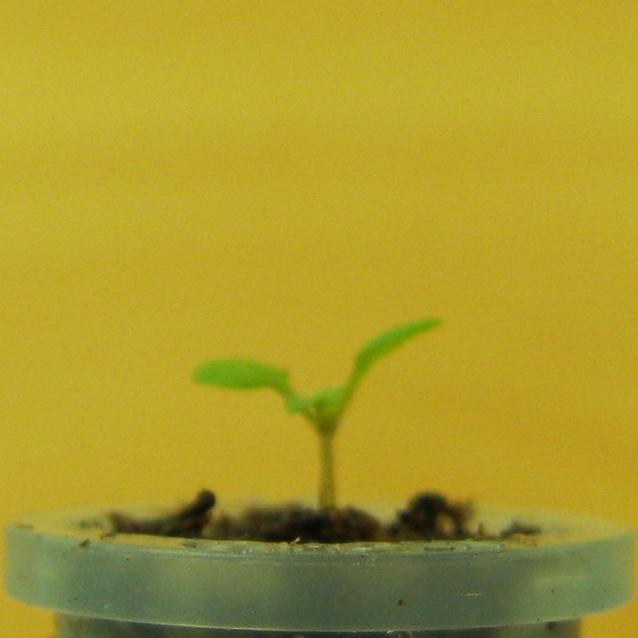

Supplement: Additional file 20 — Col-0 Front View Images for 3-D Model. Images of Col-0 captured every 10 min for 5 days from the front view for the 3-D CG model. Table S2 lists the images used as key frames in the model. [file 13007_2015_75_MOESM20_ESM.zip › front_view/side12_0148.jpg]

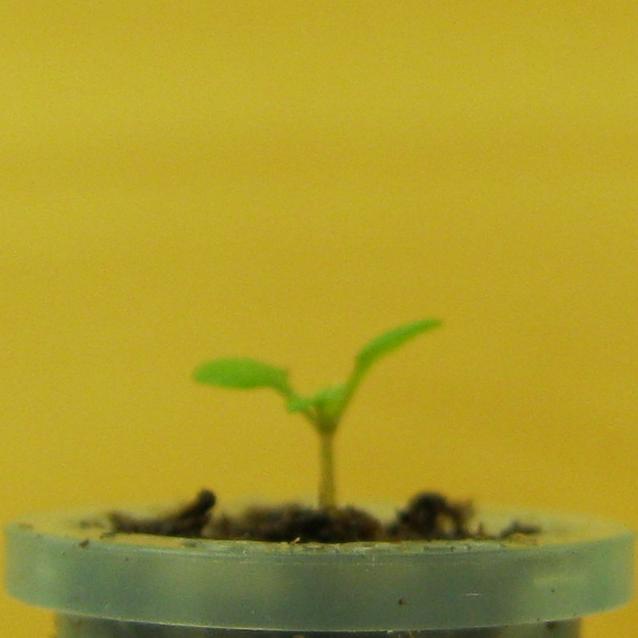

Supplement: Additional file 20 — Col-0 Front View Images for 3-D Model. Images of Col-0 captured every 10 min for 5 days from the front view for the 3-D CG model. Table S2 lists the images used as key frames in the model. [file 13007_2015_75_MOESM20_ESM.zip › front_view/side12_0149.jpg]

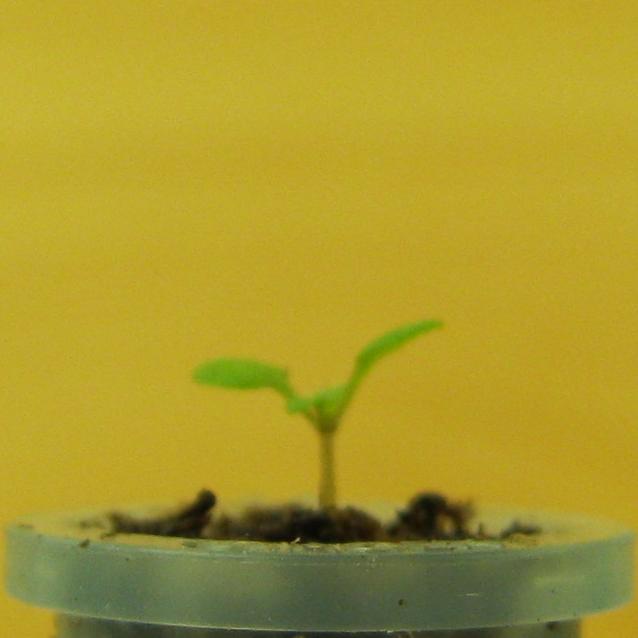

Supplement: Additional file 20 — Col-0 Front View Images for 3-D Model. Images of Col-0 captured every 10 min for 5 days from the front view for the 3-D CG model. Table S2 lists the images used as key frames in the model. [file 13007_2015_75_MOESM20_ESM.zip › front_view/side12_0150.jpg]

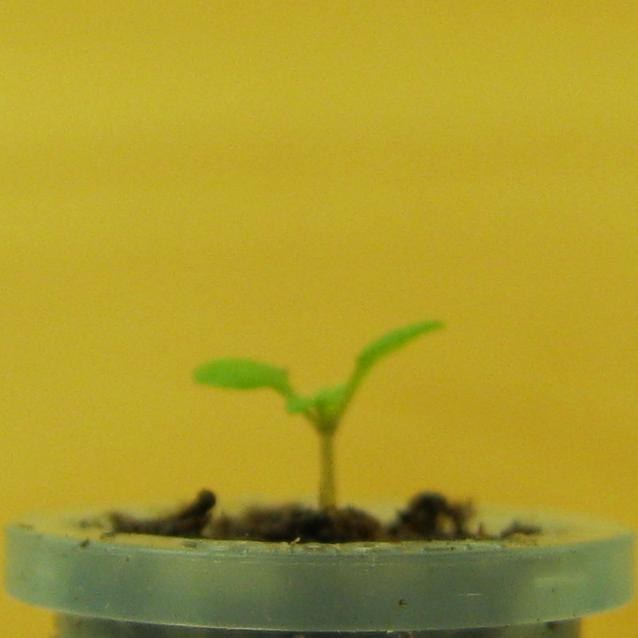

Supplement: Additional file 20 — Col-0 Front View Images for 3-D Model. Images of Col-0 captured every 10 min for 5 days from the front view for the 3-D CG model. Table S2 lists the images used as key frames in the model. [file 13007_2015_75_MOESM20_ESM.zip › front_view/side12_0151.jpg]

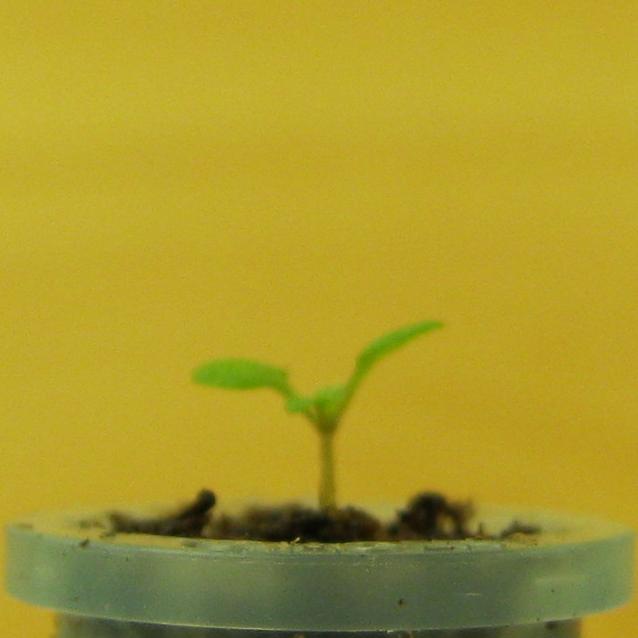

Supplement: Additional file 20 — Col-0 Front View Images for 3-D Model. Images of Col-0 captured every 10 min for 5 days from the front view for the 3-D CG model. Table S2 lists the images used as key frames in the model. [file 13007_2015_75_MOESM20_ESM.zip › front_view/side12_0152.jpg]

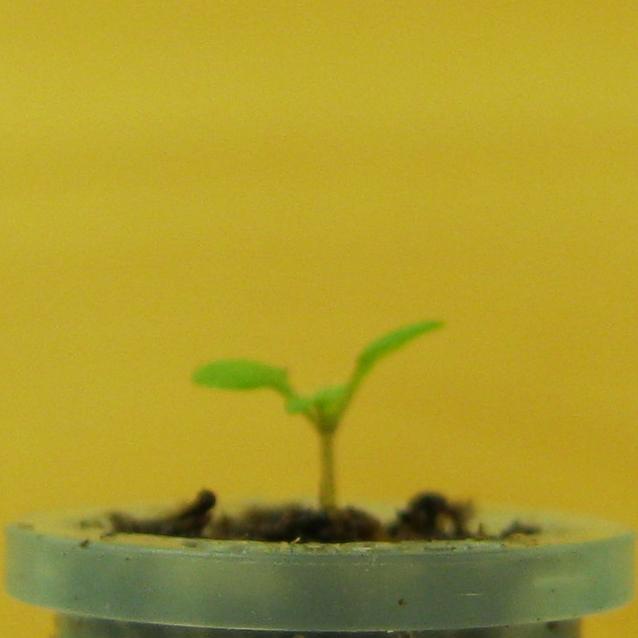

Supplement: Additional file 20 — Col-0 Front View Images for 3-D Model. Images of Col-0 captured every 10 min for 5 days from the front view for the 3-D CG model. Table S2 lists the images used as key frames in the model. [file 13007_2015_75_MOESM20_ESM.zip › front_view/side12_0153.jpg]

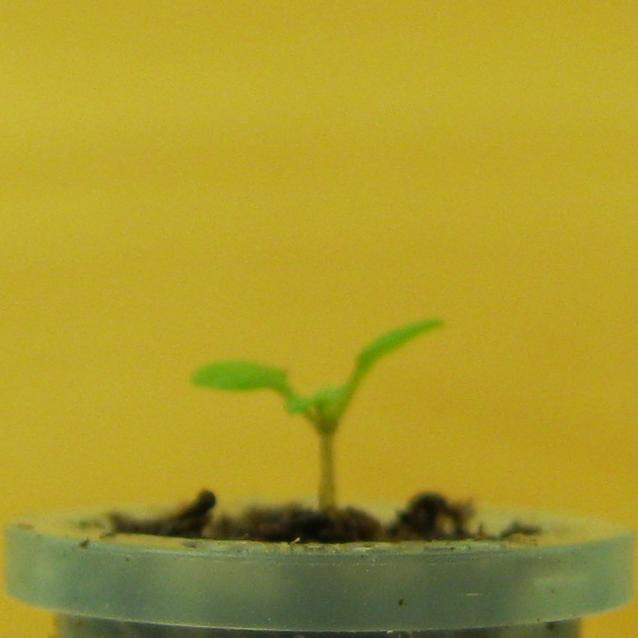

Supplement: Additional file 20 — Col-0 Front View Images for 3-D Model. Images of Col-0 captured every 10 min for 5 days from the front view for the 3-D CG model. Table S2 lists the images used as key frames in the model. [file 13007_2015_75_MOESM20_ESM.zip › front_view/side12_0154.jpg]

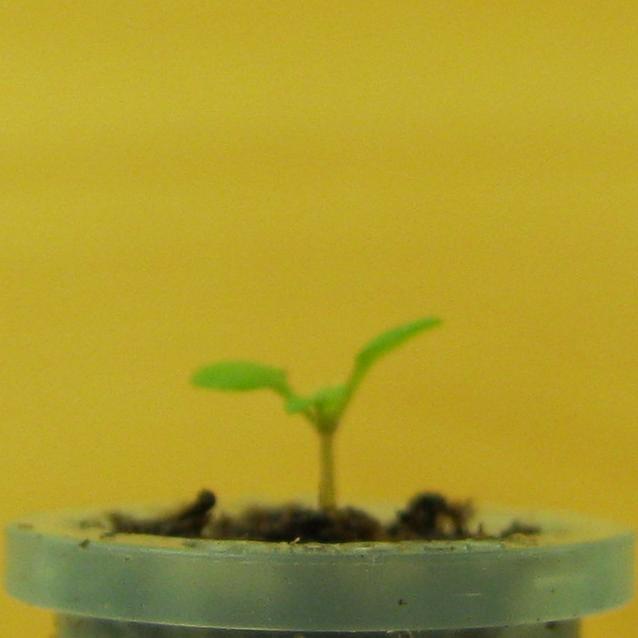

Supplement: Additional file 20 — Col-0 Front View Images for 3-D Model. Images of Col-0 captured every 10 min for 5 days from the front view for the 3-D CG model. Table S2 lists the images used as key frames in the model. [file 13007_2015_75_MOESM20_ESM.zip › front_view/side12_0155.jpg]

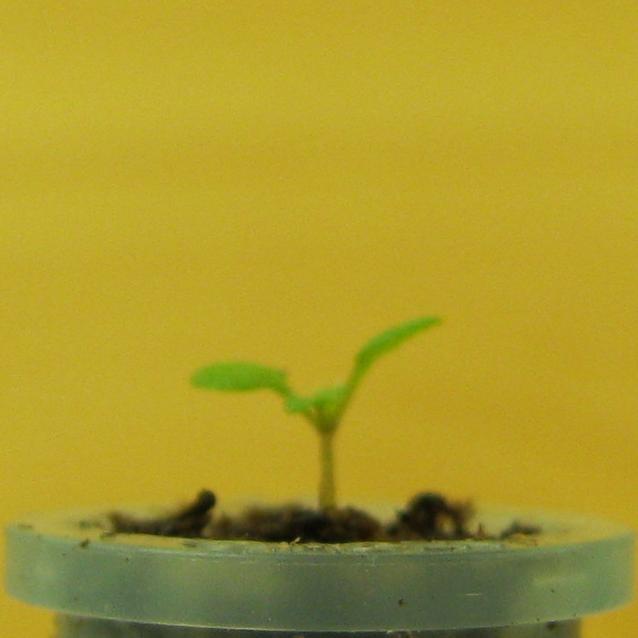

Supplement: Additional file 20 — Col-0 Front View Images for 3-D Model. Images of Col-0 captured every 10 min for 5 days from the front view for the 3-D CG model. Table S2 lists the images used as key frames in the model. [file 13007_2015_75_MOESM20_ESM.zip › front_view/side12_0156.jpg]

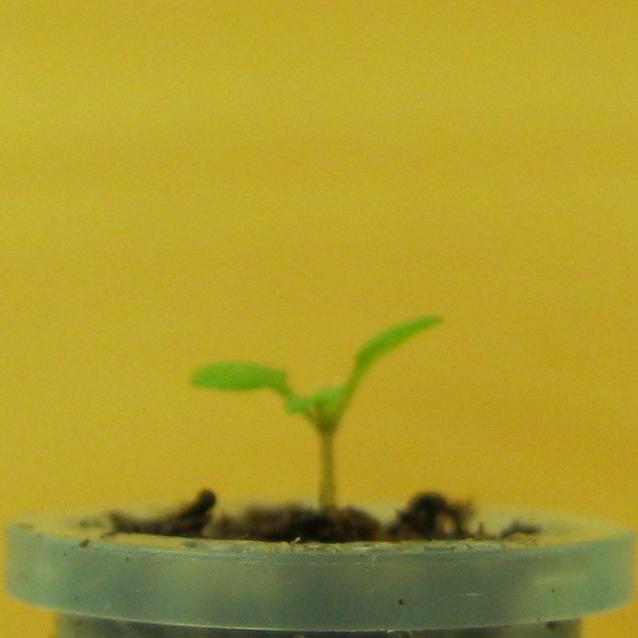

Supplement: Additional file 20 — Col-0 Front View Images for 3-D Model. Images of Col-0 captured every 10 min for 5 days from the front view for the 3-D CG model. Table S2 lists the images used as key frames in the model. [file 13007_2015_75_MOESM20_ESM.zip › front_view/side12_0157.jpg]

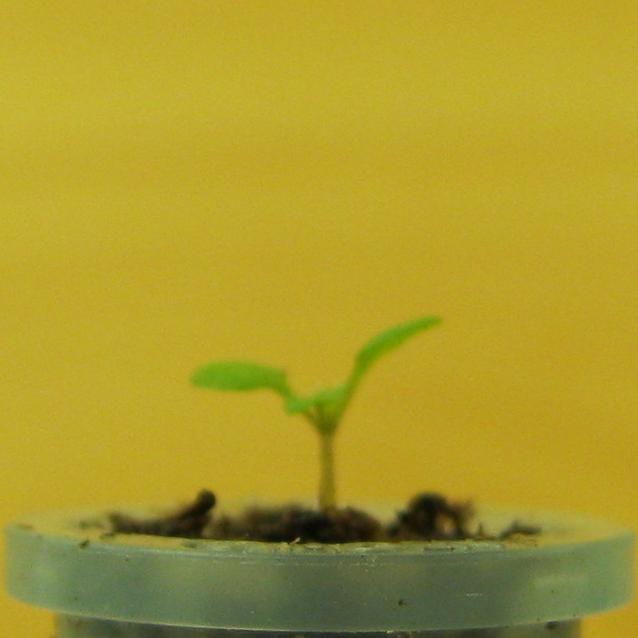

Supplement: Additional file 20 — Col-0 Front View Images for 3-D Model. Images of Col-0 captured every 10 min for 5 days from the front view for the 3-D CG model. Table S2 lists the images used as key frames in the model. [file 13007_2015_75_MOESM20_ESM.zip › front_view/side12_0158.jpg]

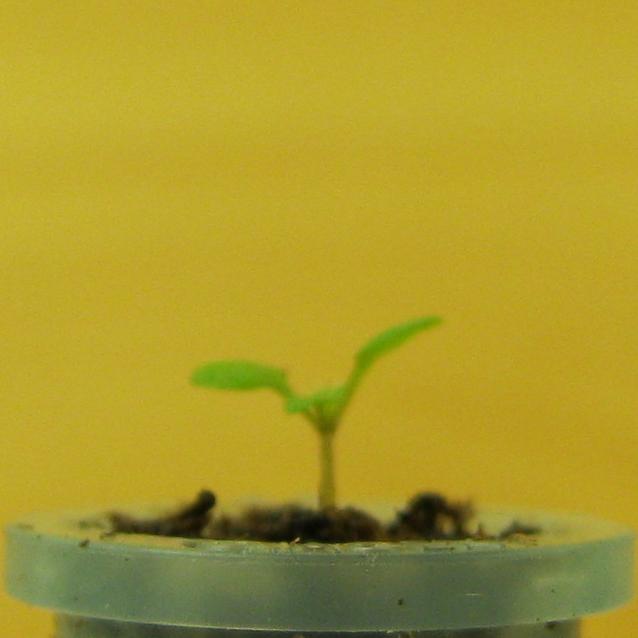

Supplement: Additional file 20 — Col-0 Front View Images for 3-D Model. Images of Col-0 captured every 10 min for 5 days from the front view for the 3-D CG model. Table S2 lists the images used as key frames in the model. [file 13007_2015_75_MOESM20_ESM.zip › front_view/side12_0159.jpg]

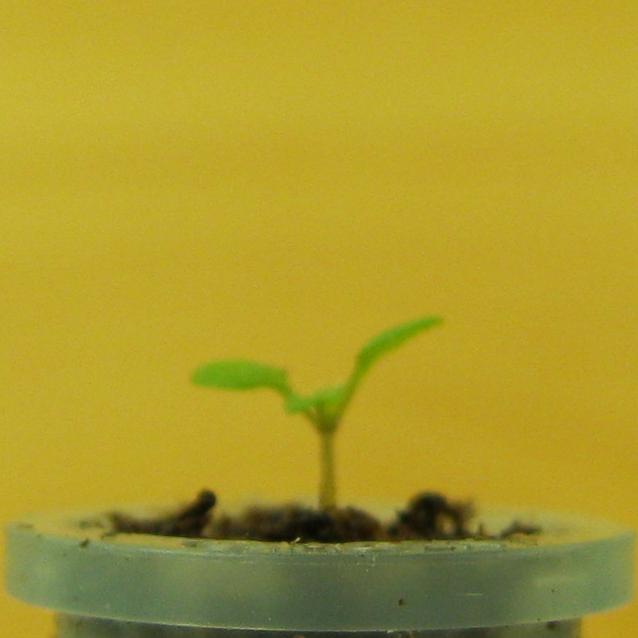

Supplement: Additional file 20 — Col-0 Front View Images for 3-D Model. Images of Col-0 captured every 10 min for 5 days from the front view for the 3-D CG model. Table S2 lists the images used as key frames in the model. [file 13007_2015_75_MOESM20_ESM.zip › front_view/side12_0160.jpg]

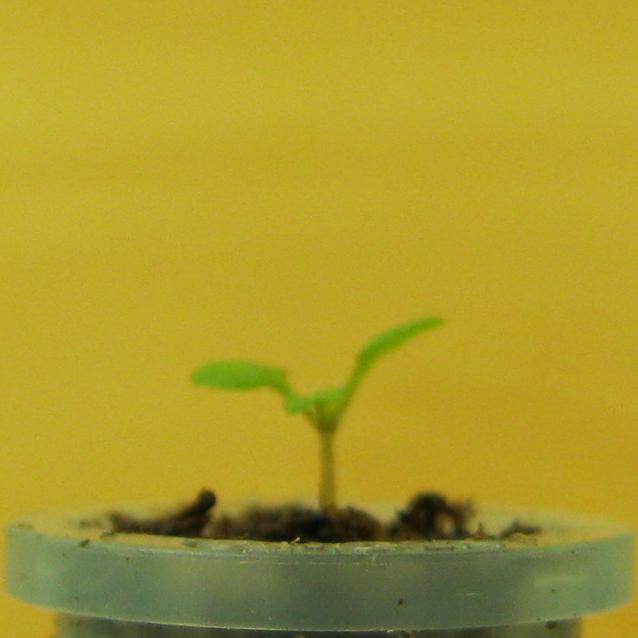

Supplement: Additional file 20 — Col-0 Front View Images for 3-D Model. Images of Col-0 captured every 10 min for 5 days from the front view for the 3-D CG model. Table S2 lists the images used as key frames in the model. [file 13007_2015_75_MOESM20_ESM.zip › front_view/side12_0161.jpg]

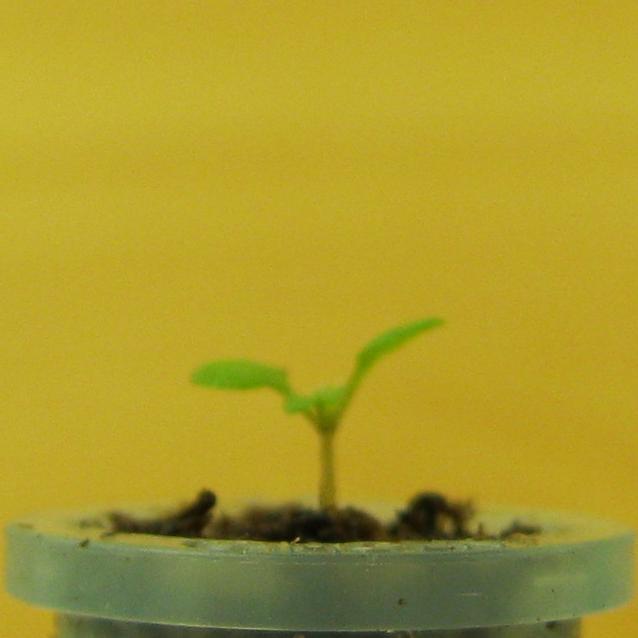

Supplement: Additional file 20 — Col-0 Front View Images for 3-D Model. Images of Col-0 captured every 10 min for 5 days from the front view for the 3-D CG model. Table S2 lists the images used as key frames in the model. [file 13007_2015_75_MOESM20_ESM.zip › front_view/side12_0162.jpg]

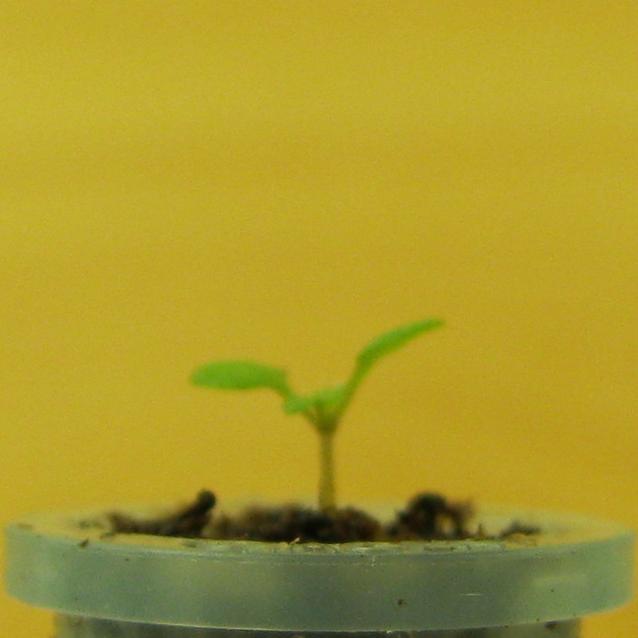

Supplement: Additional file 20 — Col-0 Front View Images for 3-D Model. Images of Col-0 captured every 10 min for 5 days from the front view for the 3-D CG model. Table S2 lists the images used as key frames in the model. [file 13007_2015_75_MOESM20_ESM.zip › front_view/side12_0163.jpg]

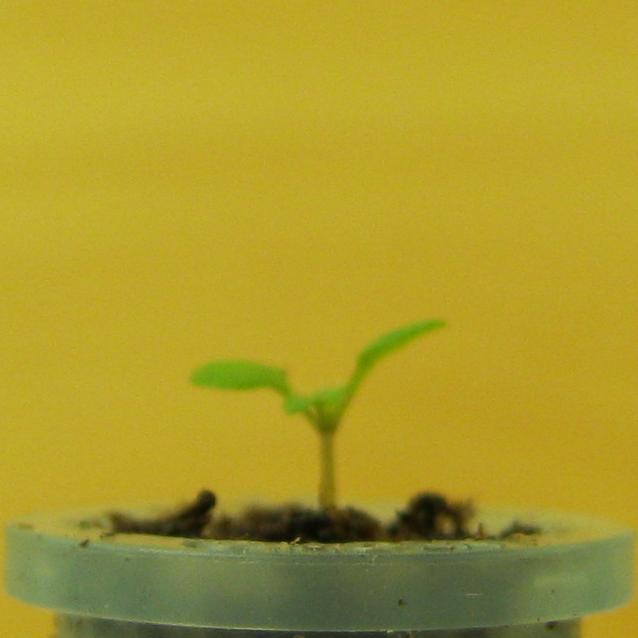

Supplement: Additional file 20 — Col-0 Front View Images for 3-D Model. Images of Col-0 captured every 10 min for 5 days from the front view for the 3-D CG model. Table S2 lists the images used as key frames in the model. [file 13007_2015_75_MOESM20_ESM.zip › front_view/side12_0164.jpg]

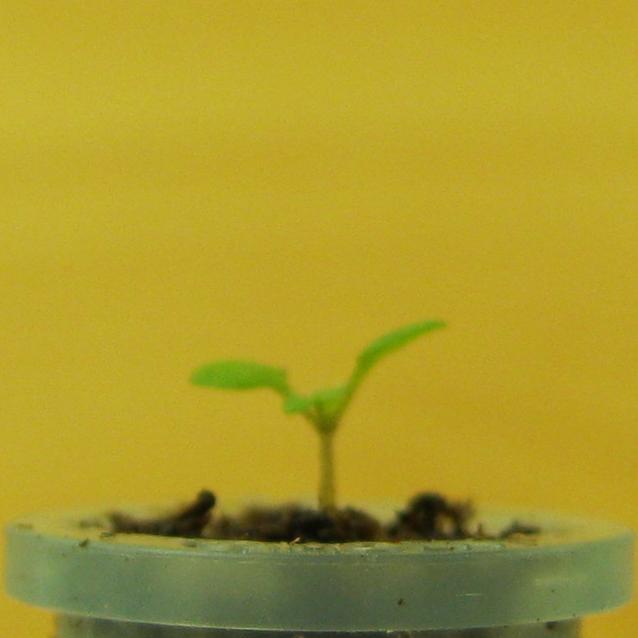

Supplement: Additional file 20 — Col-0 Front View Images for 3-D Model. Images of Col-0 captured every 10 min for 5 days from the front view for the 3-D CG model. Table S2 lists the images used as key frames in the model. [file 13007_2015_75_MOESM20_ESM.zip › front_view/side12_0165.jpg]

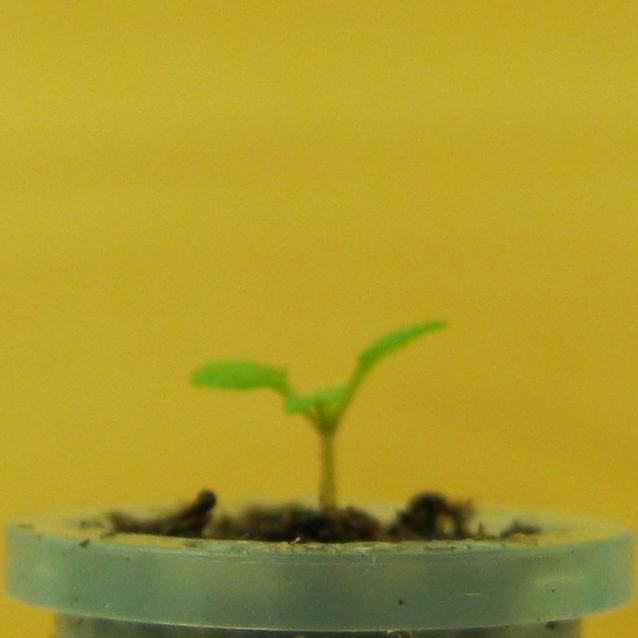

Supplement: Additional file 20 — Col-0 Front View Images for 3-D Model. Images of Col-0 captured every 10 min for 5 days from the front view for the 3-D CG model. Table S2 lists the images used as key frames in the model. [file 13007_2015_75_MOESM20_ESM.zip › front_view/side12_0166.jpg]

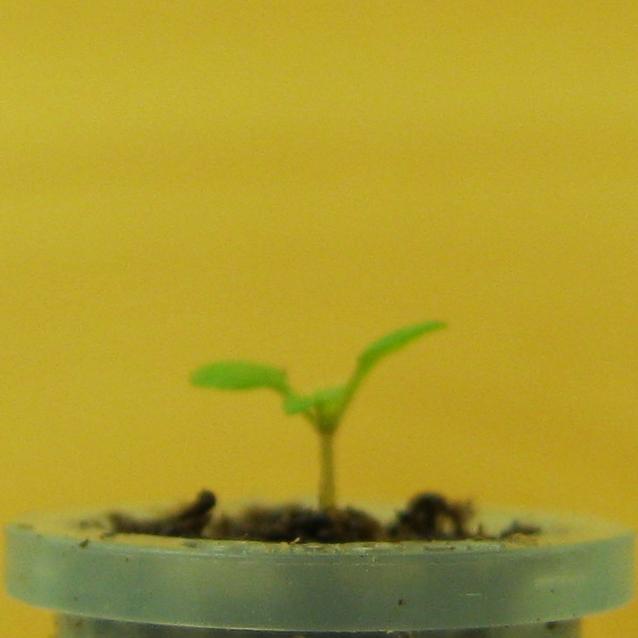

Supplement: Additional file 20 — Col-0 Front View Images for 3-D Model. Images of Col-0 captured every 10 min for 5 days from the front view for the 3-D CG model. Table S2 lists the images used as key frames in the model. [file 13007_2015_75_MOESM20_ESM.zip › front_view/side12_0167.jpg]

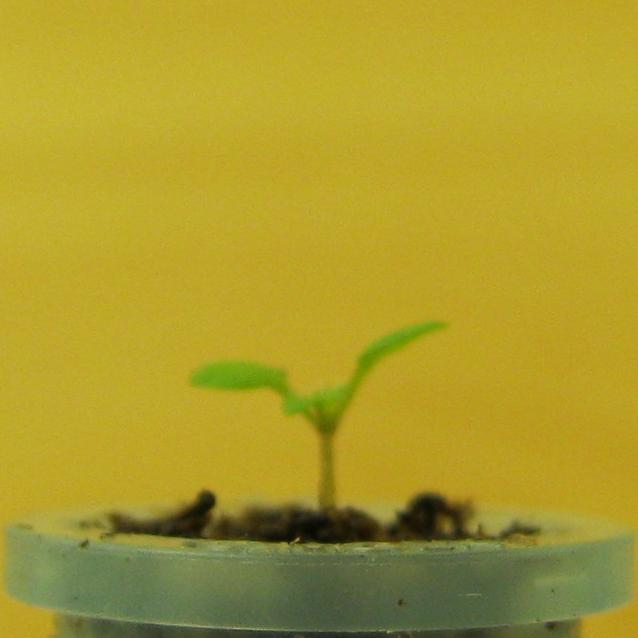

Supplement: Additional file 20 — Col-0 Front View Images for 3-D Model. Images of Col-0 captured every 10 min for 5 days from the front view for the 3-D CG model. Table S2 lists the images used as key frames in the model. [file 13007_2015_75_MOESM20_ESM.zip › front_view/side12_0168.jpg]

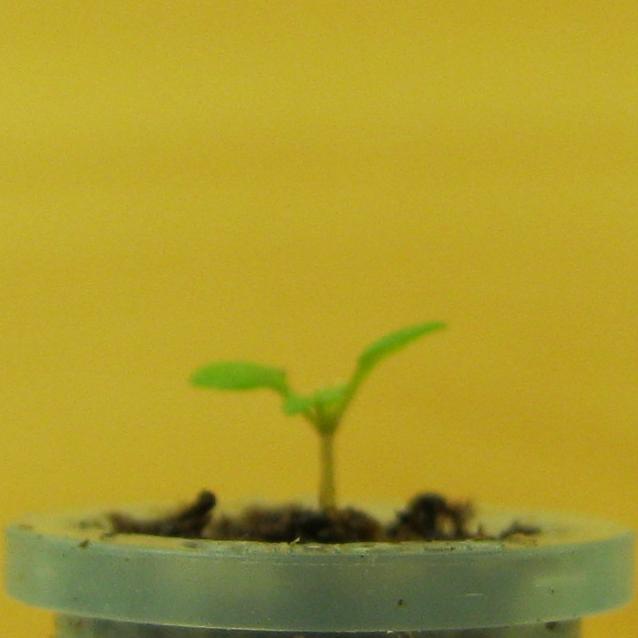

Supplement: Additional file 20 — Col-0 Front View Images for 3-D Model. Images of Col-0 captured every 10 min for 5 days from the front view for the 3-D CG model. Table S2 lists the images used as key frames in the model. [file 13007_2015_75_MOESM20_ESM.zip › front_view/side12_0169.jpg]

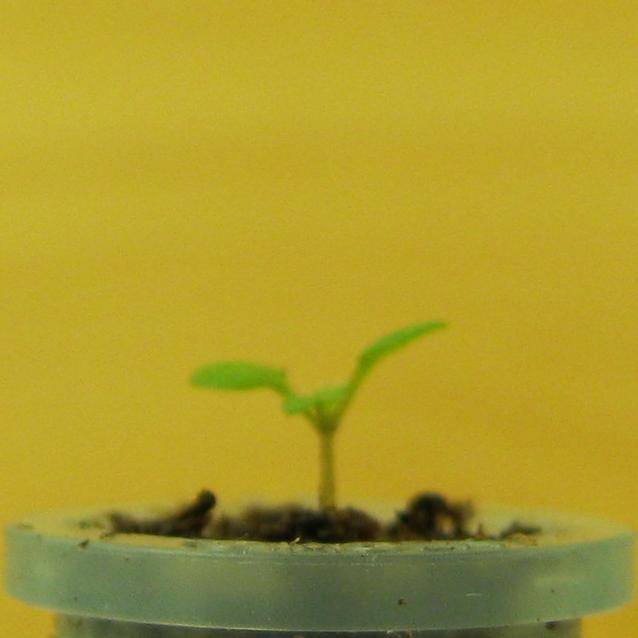

Supplement: Additional file 20 — Col-0 Front View Images for 3-D Model. Images of Col-0 captured every 10 min for 5 days from the front view for the 3-D CG model. Table S2 lists the images used as key frames in the model. [file 13007_2015_75_MOESM20_ESM.zip › front_view/side12_0170.jpg]

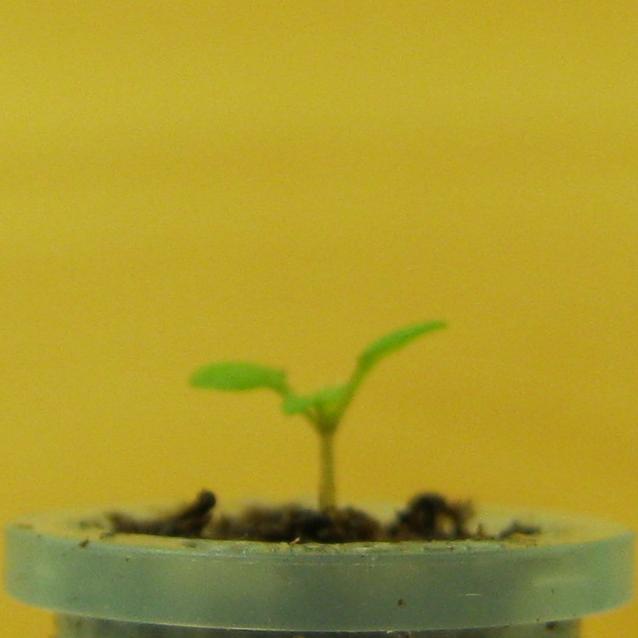

Supplement: Additional file 20 — Col-0 Front View Images for 3-D Model. Images of Col-0 captured every 10 min for 5 days from the front view for the 3-D CG model. Table S2 lists the images used as key frames in the model. [file 13007_2015_75_MOESM20_ESM.zip › front_view/side12_0171.jpg]

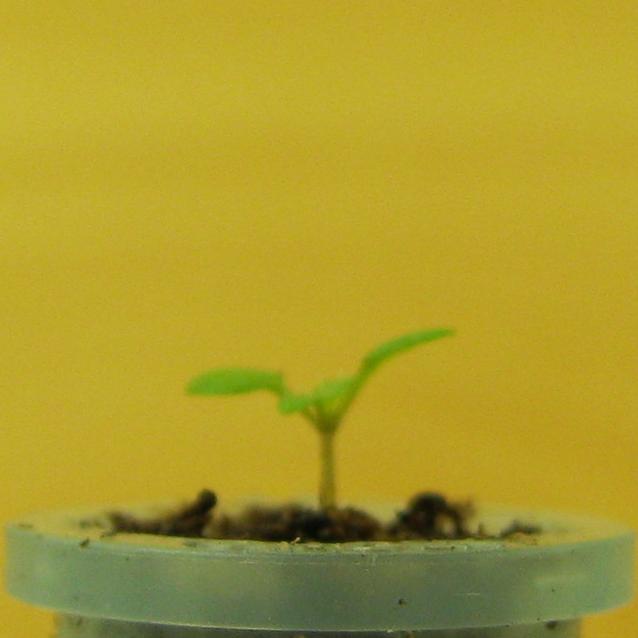

Supplement: Additional file 20 — Col-0 Front View Images for 3-D Model. Images of Col-0 captured every 10 min for 5 days from the front view for the 3-D CG model. Table S2 lists the images used as key frames in the model. [file 13007_2015_75_MOESM20_ESM.zip › front_view/side12_0172.jpg]

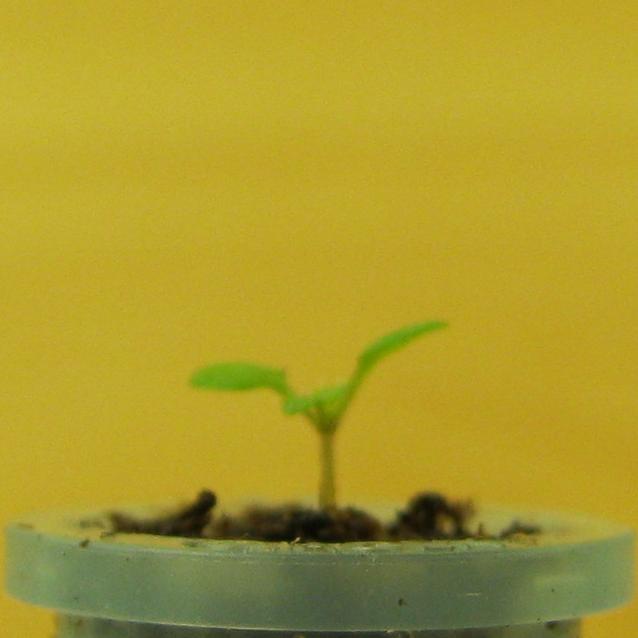

Supplement: Additional file 20 — Col-0 Front View Images for 3-D Model. Images of Col-0 captured every 10 min for 5 days from the front view for the 3-D CG model. Table S2 lists the images used as key frames in the model. [file 13007_2015_75_MOESM20_ESM.zip › front_view/side12_0173.jpg]

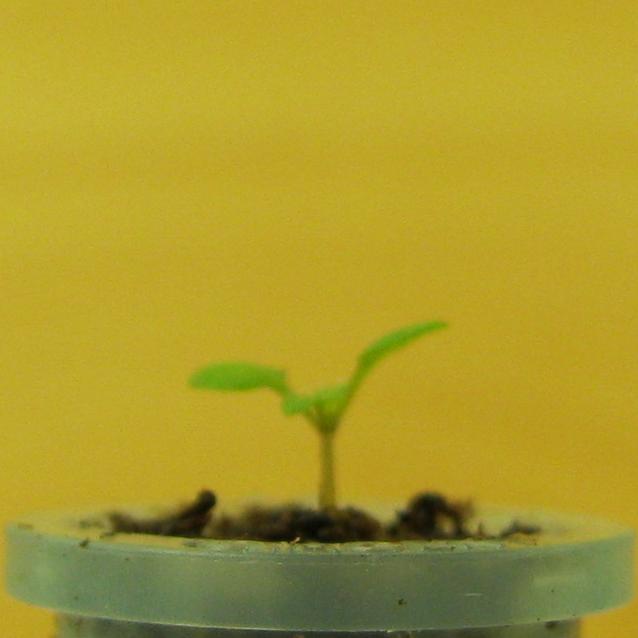

Supplement: Additional file 20 — Col-0 Front View Images for 3-D Model. Images of Col-0 captured every 10 min for 5 days from the front view for the 3-D CG model. Table S2 lists the images used as key frames in the model. [file 13007_2015_75_MOESM20_ESM.zip › front_view/side12_0174.jpg]

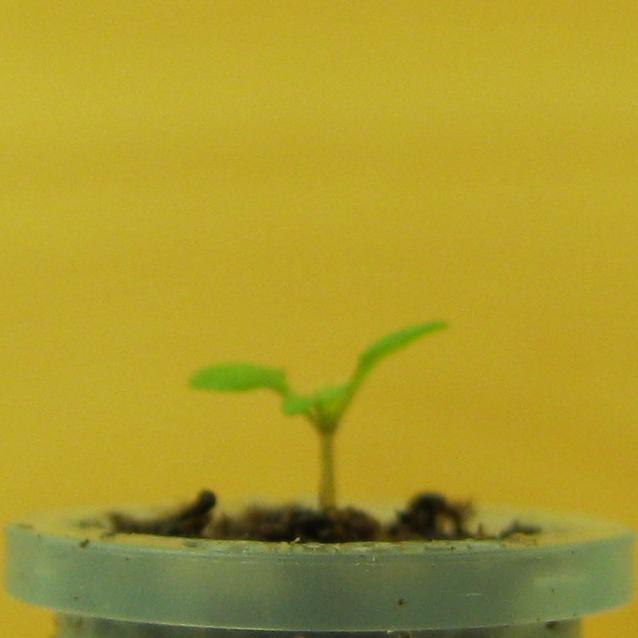

Supplement: Additional file 20 — Col-0 Front View Images for 3-D Model. Images of Col-0 captured every 10 min for 5 days from the front view for the 3-D CG model. Table S2 lists the images used as key frames in the model. [file 13007_2015_75_MOESM20_ESM.zip › front_view/side12_0175.jpg]

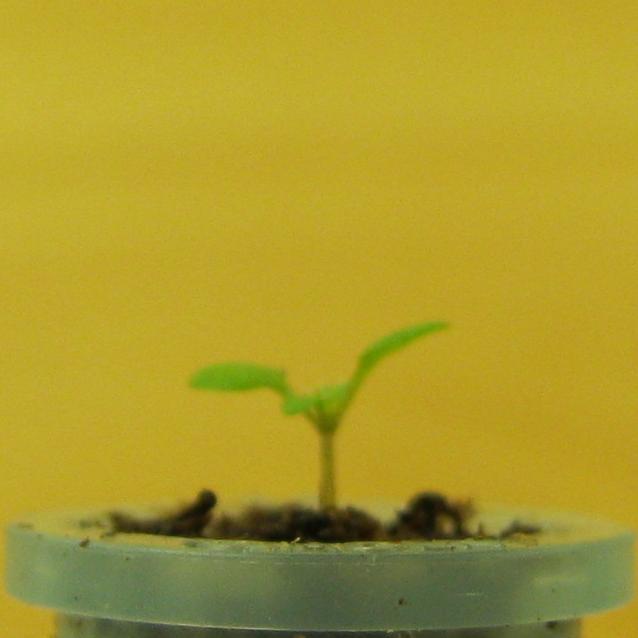

Supplement: Additional file 20 — Col-0 Front View Images for 3-D Model. Images of Col-0 captured every 10 min for 5 days from the front view for the 3-D CG model. Table S2 lists the images used as key frames in the model. [file 13007_2015_75_MOESM20_ESM.zip › front_view/side12_0176.jpg]

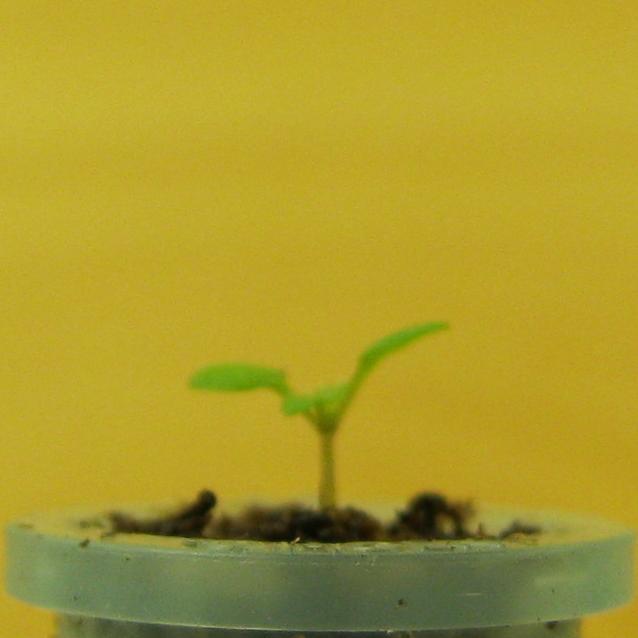

Supplement: Additional file 20 — Col-0 Front View Images for 3-D Model. Images of Col-0 captured every 10 min for 5 days from the front view for the 3-D CG model. Table S2 lists the images used as key frames in the model. [file 13007_2015_75_MOESM20_ESM.zip › front_view/side12_0177.jpg]

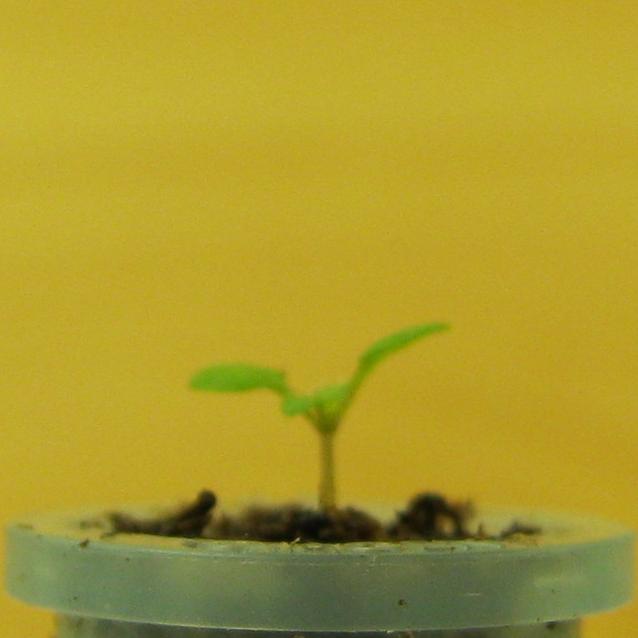

Supplement: Additional file 20 — Col-0 Front View Images for 3-D Model. Images of Col-0 captured every 10 min for 5 days from the front view for the 3-D CG model. Table S2 lists the images used as key frames in the model. [file 13007_2015_75_MOESM20_ESM.zip › front_view/side12_0178.jpg]

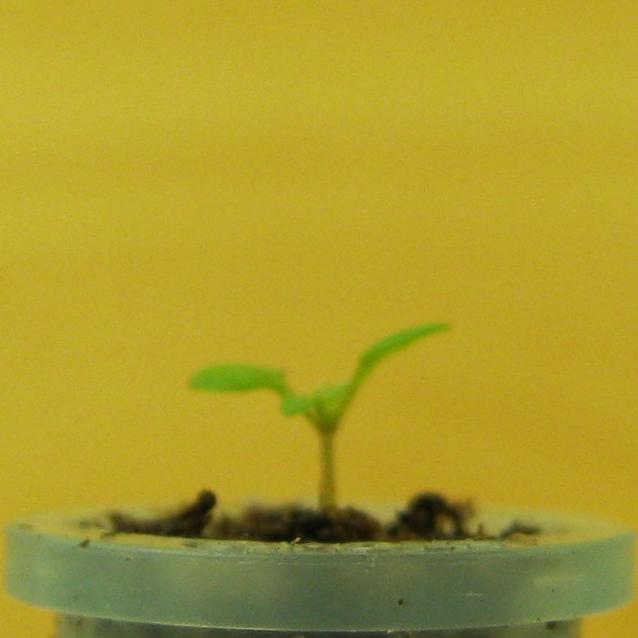

Supplement: Additional file 20 — Col-0 Front View Images for 3-D Model. Images of Col-0 captured every 10 min for 5 days from the front view for the 3-D CG model. Table S2 lists the images used as key frames in the model. [file 13007_2015_75_MOESM20_ESM.zip › front_view/side12_0179.jpg]

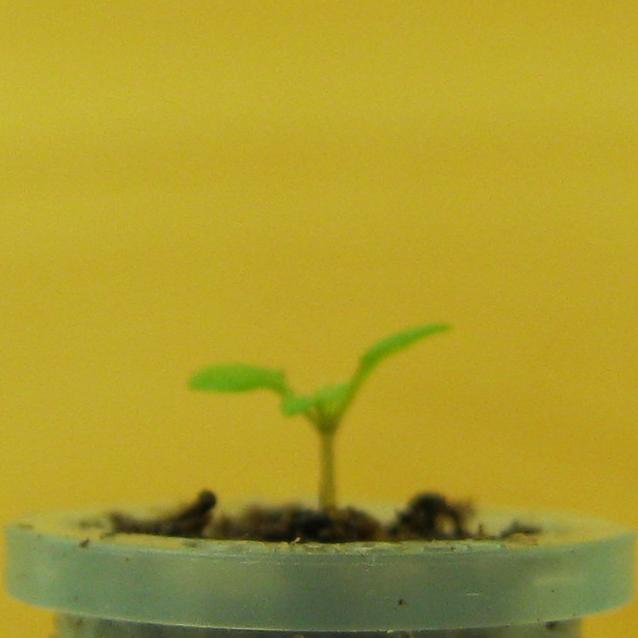

Supplement: Additional file 20 — Col-0 Front View Images for 3-D Model. Images of Col-0 captured every 10 min for 5 days from the front view for the 3-D CG model. Table S2 lists the images used as key frames in the model. [file 13007_2015_75_MOESM20_ESM.zip › front_view/side12_0180.jpg]

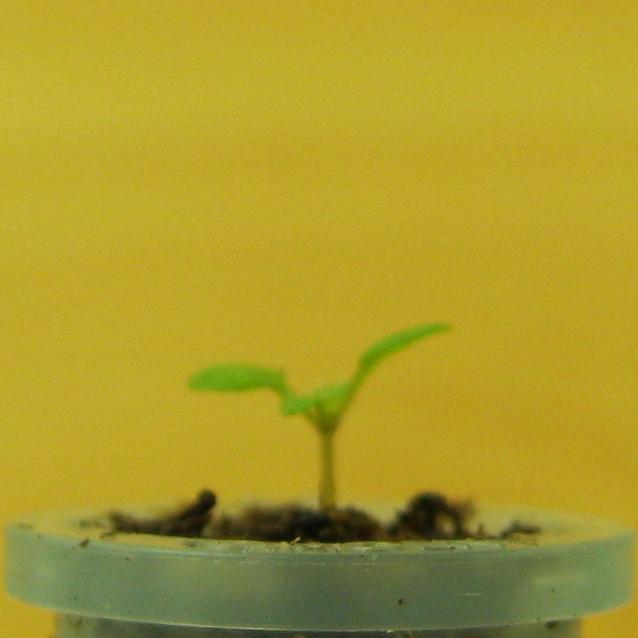

Supplement: Additional file 20 — Col-0 Front View Images for 3-D Model. Images of Col-0 captured every 10 min for 5 days from the front view for the 3-D CG model. Table S2 lists the images used as key frames in the model. [file 13007_2015_75_MOESM20_ESM.zip › front_view/side12_0181.jpg]

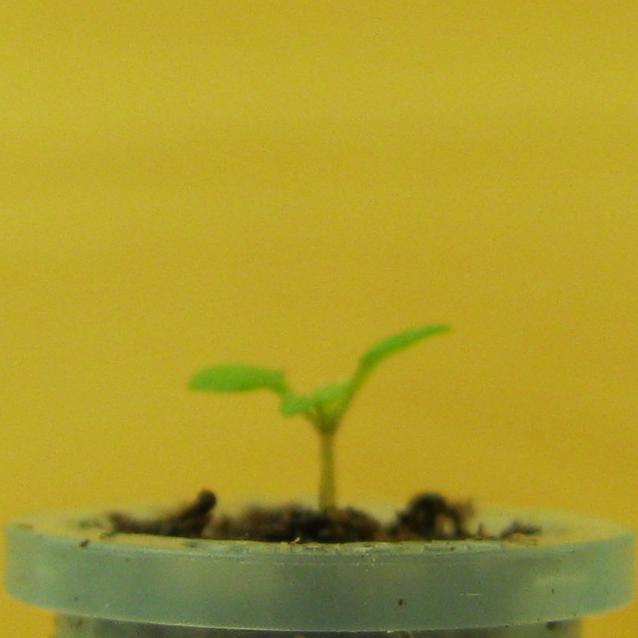

Supplement: Additional file 20 — Col-0 Front View Images for 3-D Model. Images of Col-0 captured every 10 min for 5 days from the front view for the 3-D CG model. Table S2 lists the images used as key frames in the model. [file 13007_2015_75_MOESM20_ESM.zip › front_view/side12_0182.jpg]

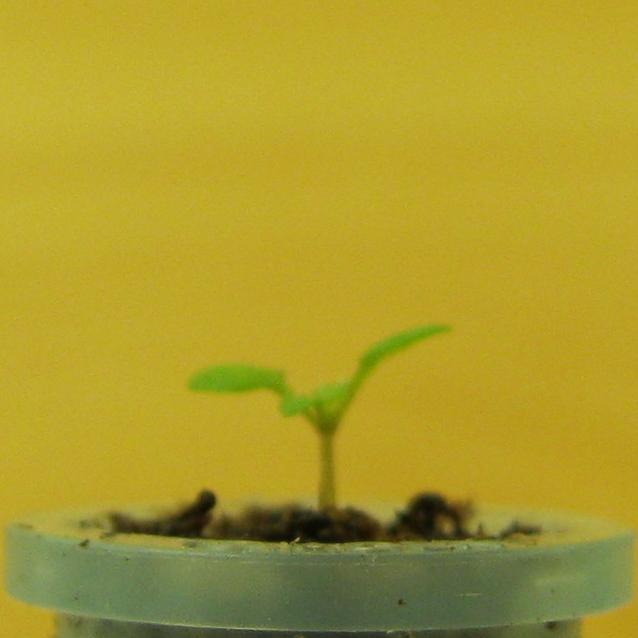

Supplement: Additional file 20 — Col-0 Front View Images for 3-D Model. Images of Col-0 captured every 10 min for 5 days from the front view for the 3-D CG model. Table S2 lists the images used as key frames in the model. [file 13007_2015_75_MOESM20_ESM.zip › front_view/side12_0183.jpg]

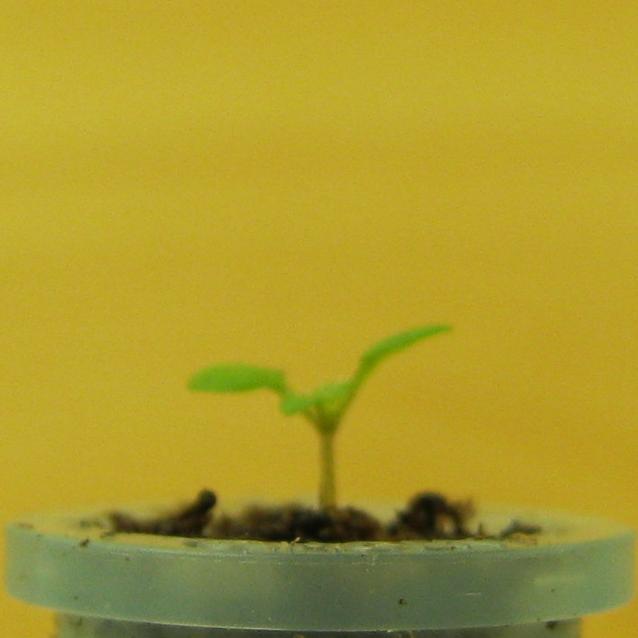

Supplement: Additional file 20 — Col-0 Front View Images for 3-D Model. Images of Col-0 captured every 10 min for 5 days from the front view for the 3-D CG model. Table S2 lists the images used as key frames in the model. [file 13007_2015_75_MOESM20_ESM.zip › front_view/side12_0184.jpg]

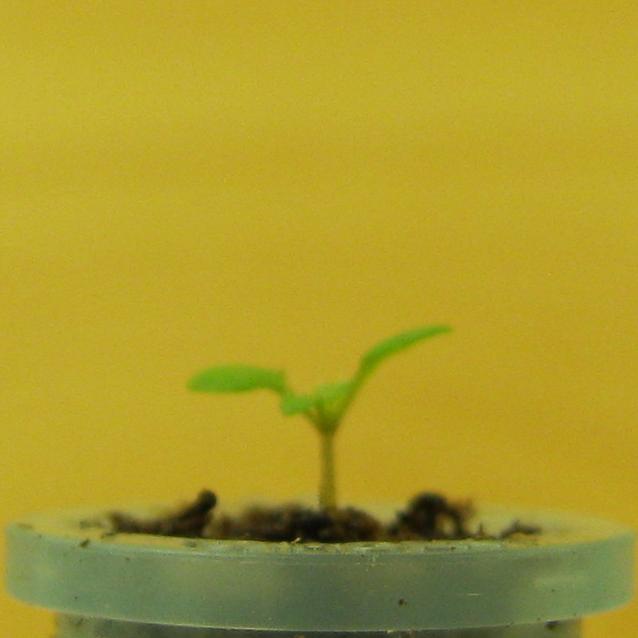

Supplement: Additional file 20 — Col-0 Front View Images for 3-D Model. Images of Col-0 captured every 10 min for 5 days from the front view for the 3-D CG model. Table S2 lists the images used as key frames in the model. [file 13007_2015_75_MOESM20_ESM.zip › front_view/side12_0185.jpg]

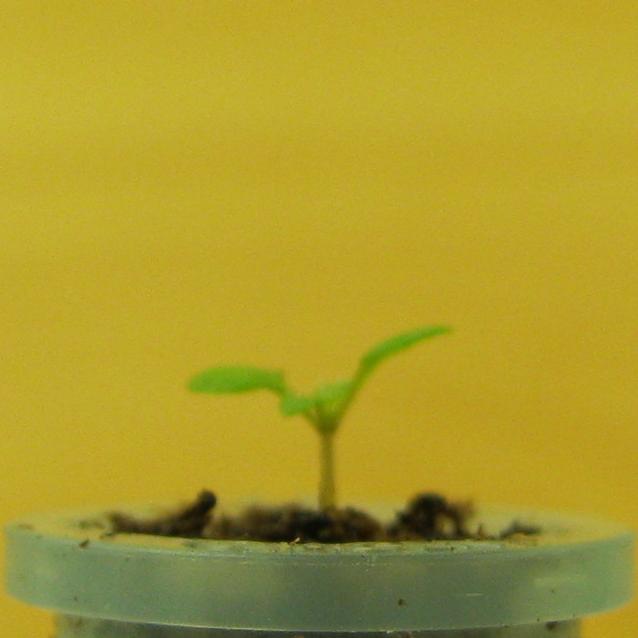

Supplement: Additional file 20 — Col-0 Front View Images for 3-D Model. Images of Col-0 captured every 10 min for 5 days from the front view for the 3-D CG model. Table S2 lists the images used as key frames in the model. [file 13007_2015_75_MOESM20_ESM.zip › front_view/side12_0186.jpg]

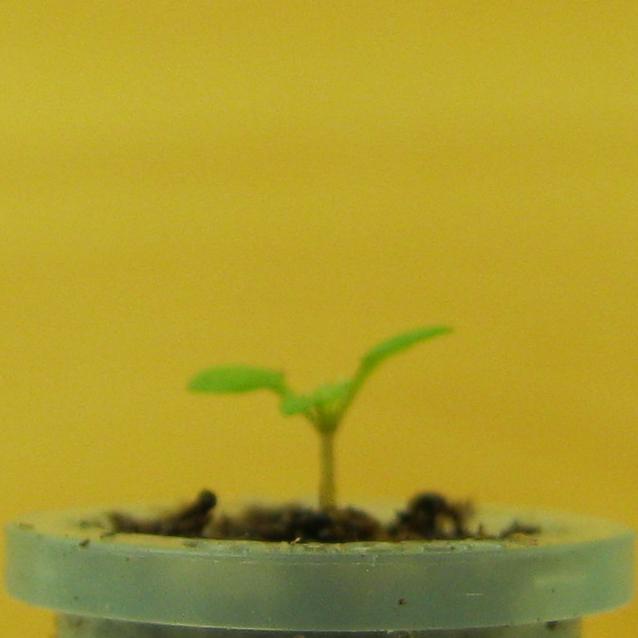

Supplement: Additional file 20 — Col-0 Front View Images for 3-D Model. Images of Col-0 captured every 10 min for 5 days from the front view for the 3-D CG model. Table S2 lists the images used as key frames in the model. [file 13007_2015_75_MOESM20_ESM.zip › front_view/side12_0187.jpg]

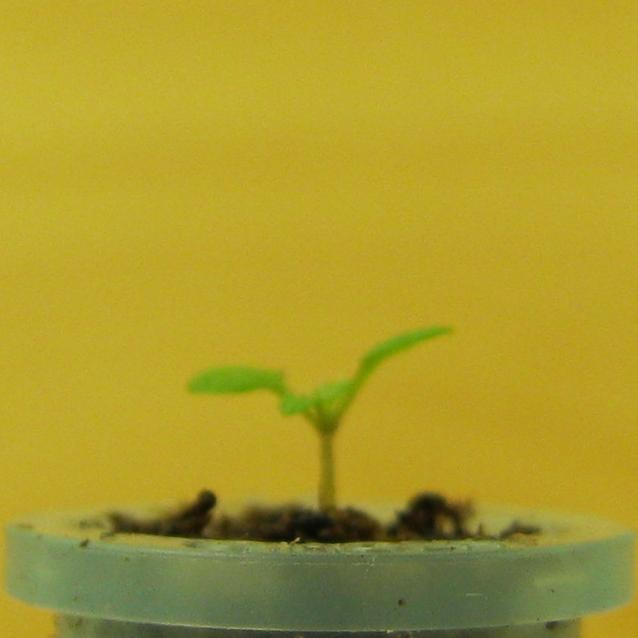

Supplement: Additional file 20 — Col-0 Front View Images for 3-D Model. Images of Col-0 captured every 10 min for 5 days from the front view for the 3-D CG model. Table S2 lists the images used as key frames in the model. [file 13007_2015_75_MOESM20_ESM.zip › front_view/side12_0188.jpg]

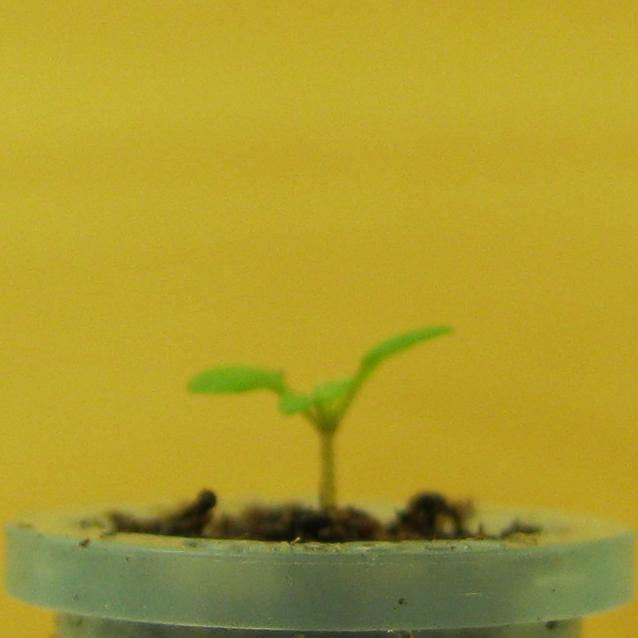

Supplement: Additional file 20 — Col-0 Front View Images for 3-D Model. Images of Col-0 captured every 10 min for 5 days from the front view for the 3-D CG model. Table S2 lists the images used as key frames in the model. [file 13007_2015_75_MOESM20_ESM.zip › front_view/side12_0189.jpg]

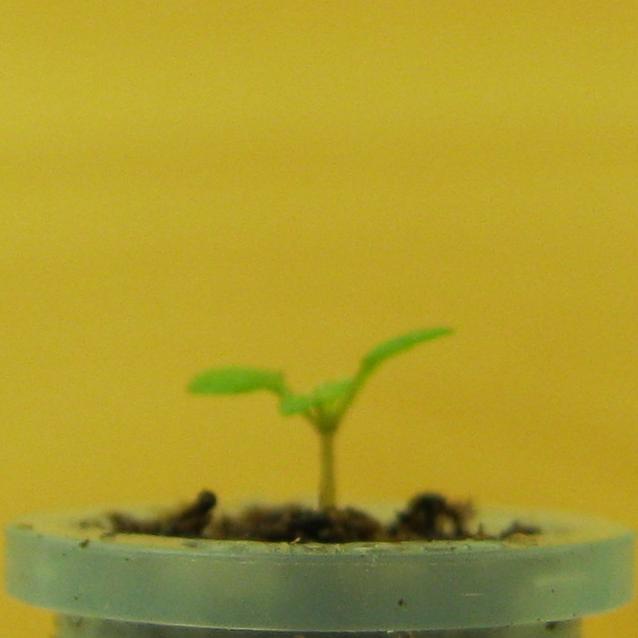

Supplement: Additional file 20 — Col-0 Front View Images for 3-D Model. Images of Col-0 captured every 10 min for 5 days from the front view for the 3-D CG model. Table S2 lists the images used as key frames in the model. [file 13007_2015_75_MOESM20_ESM.zip › front_view/side12_0190.jpg]

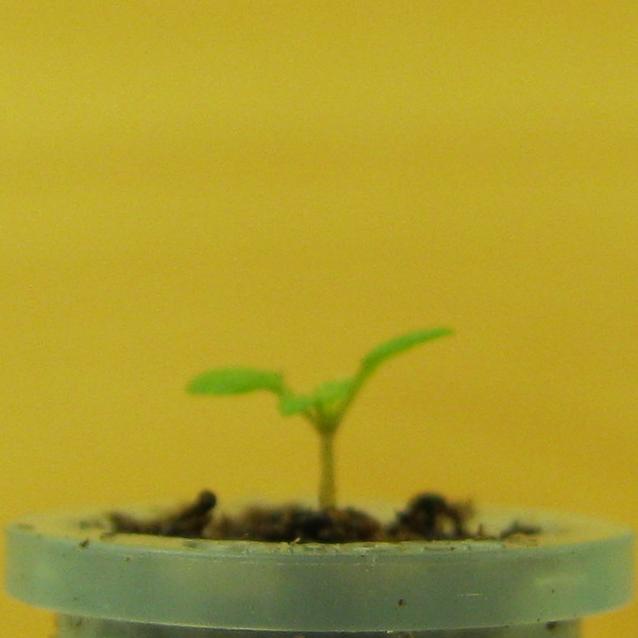

Supplement: Additional file 20 — Col-0 Front View Images for 3-D Model. Images of Col-0 captured every 10 min for 5 days from the front view for the 3-D CG model. Table S2 lists the images used as key frames in the model. [file 13007_2015_75_MOESM20_ESM.zip › front_view/side12_0191.jpg]

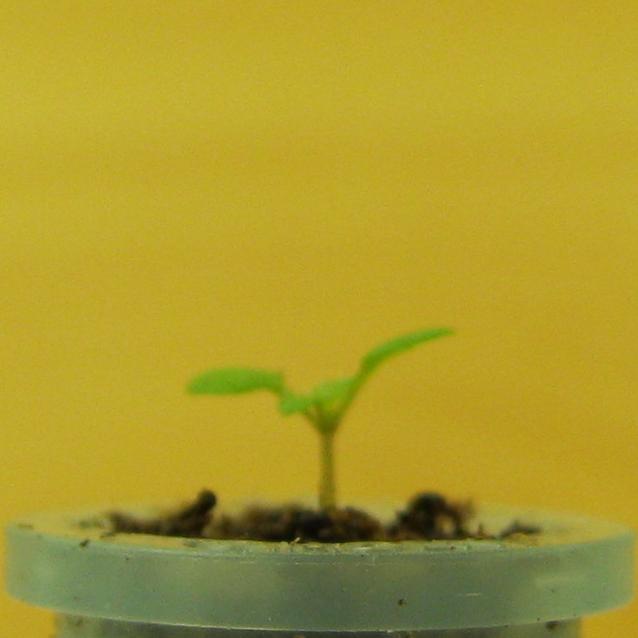

Supplement: Additional file 20 — Col-0 Front View Images for 3-D Model. Images of Col-0 captured every 10 min for 5 days from the front view for the 3-D CG model. Table S2 lists the images used as key frames in the model. [file 13007_2015_75_MOESM20_ESM.zip › front_view/side12_0192.jpg]

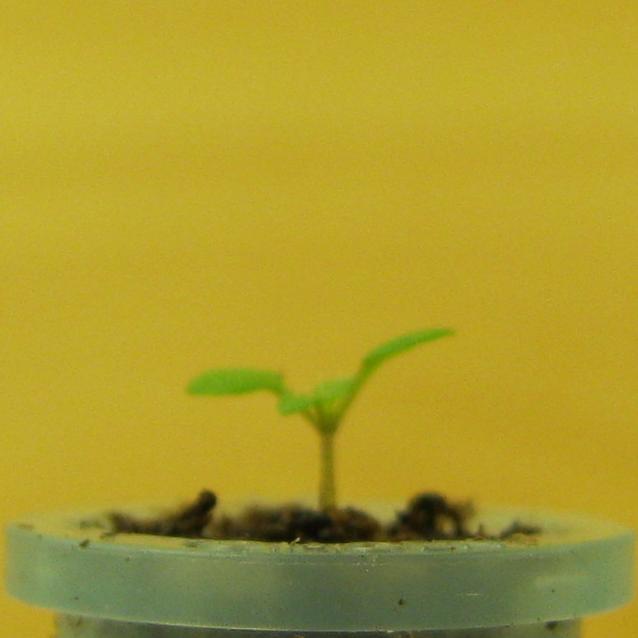

Supplement: Additional file 20 — Col-0 Front View Images for 3-D Model. Images of Col-0 captured every 10 min for 5 days from the front view for the 3-D CG model. Table S2 lists the images used as key frames in the model. [file 13007_2015_75_MOESM20_ESM.zip › front_view/side12_0193.jpg]

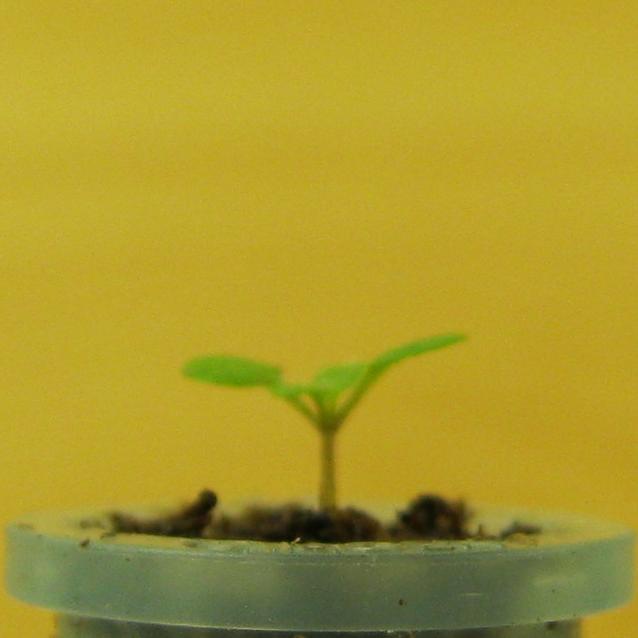

Supplement: Additional file 20 — Col-0 Front View Images for 3-D Model. Images of Col-0 captured every 10 min for 5 days from the front view for the 3-D CG model. Table S2 lists the images used as key frames in the model. [file 13007_2015_75_MOESM20_ESM.zip › front_view/side12_0194.jpg]

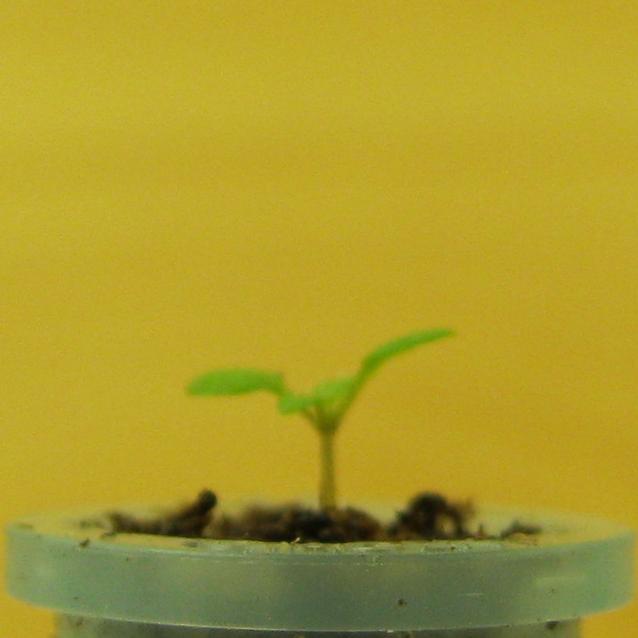

Supplement: Additional file 20 — Col-0 Front View Images for 3-D Model. Images of Col-0 captured every 10 min for 5 days from the front view for the 3-D CG model. Table S2 lists the images used as key frames in the model. [file 13007_2015_75_MOESM20_ESM.zip › front_view/side12_0195.jpg]

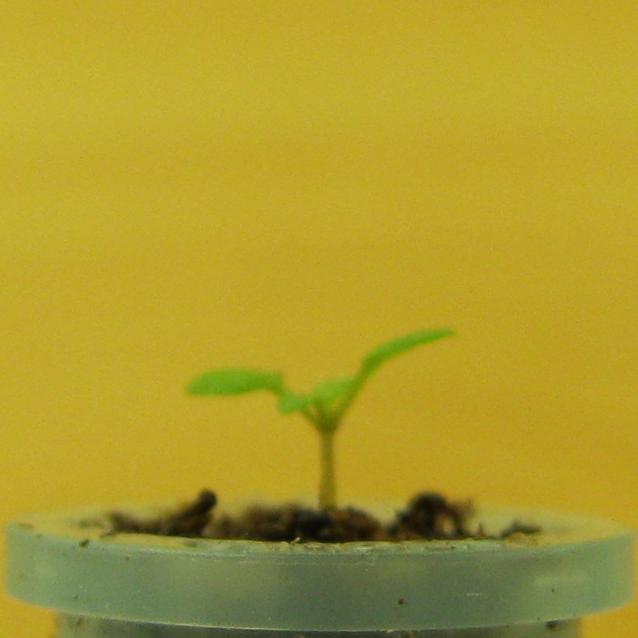

Supplement: Additional file 20 — Col-0 Front View Images for 3-D Model. Images of Col-0 captured every 10 min for 5 days from the front view for the 3-D CG model. Table S2 lists the images used as key frames in the model. [file 13007_2015_75_MOESM20_ESM.zip › front_view/side12_0196.jpg]

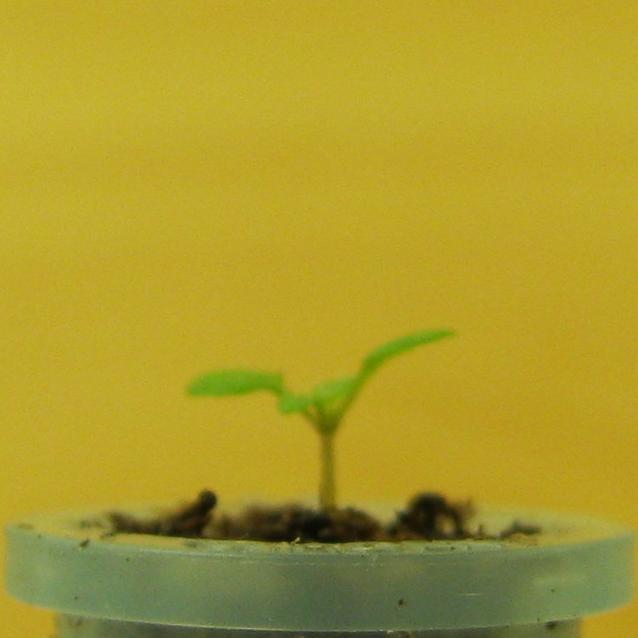

Supplement: Additional file 20 — Col-0 Front View Images for 3-D Model. Images of Col-0 captured every 10 min for 5 days from the front view for the 3-D CG model. Table S2 lists the images used as key frames in the model. [file 13007_2015_75_MOESM20_ESM.zip › front_view/side12_0197.jpg]

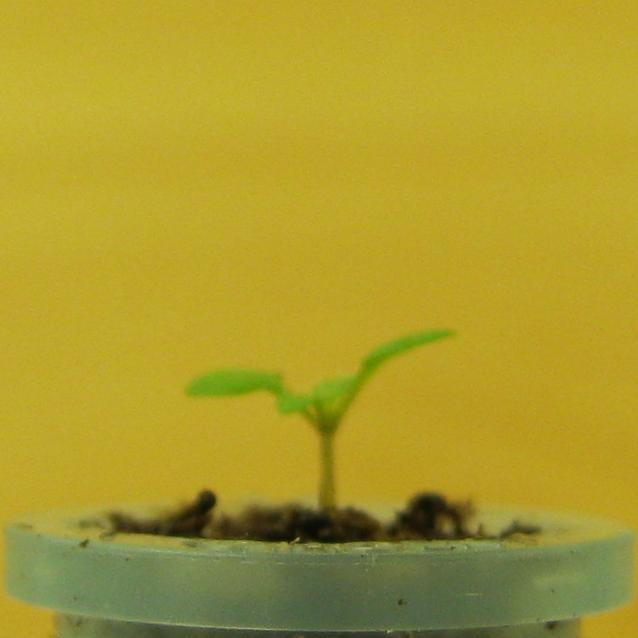

Supplement: Additional file 20 — Col-0 Front View Images for 3-D Model. Images of Col-0 captured every 10 min for 5 days from the front view for the 3-D CG model. Table S2 lists the images used as key frames in the model. [file 13007_2015_75_MOESM20_ESM.zip › front_view/side12_0198.jpg]

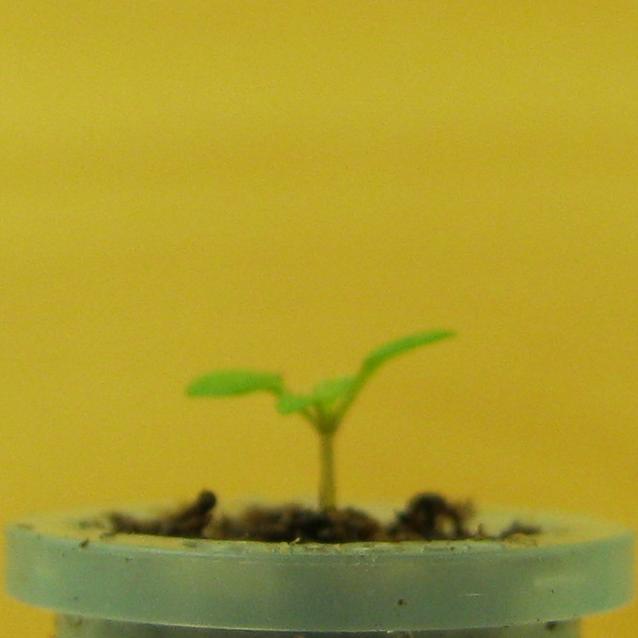

Supplement: Additional file 20 — Col-0 Front View Images for 3-D Model. Images of Col-0 captured every 10 min for 5 days from the front view for the 3-D CG model. Table S2 lists the images used as key frames in the model. [file 13007_2015_75_MOESM20_ESM.zip › front_view/side12_0199.jpg]

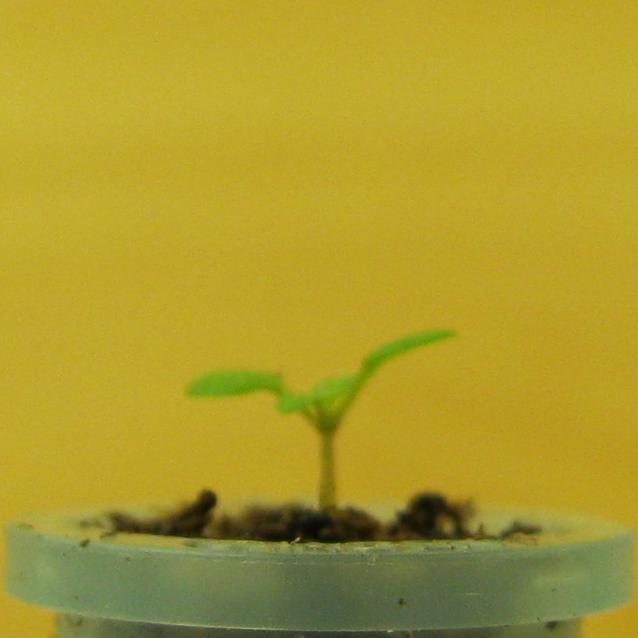

Supplement: Additional file 20 — Col-0 Front View Images for 3-D Model. Images of Col-0 captured every 10 min for 5 days from the front view for the 3-D CG model. Table S2 lists the images used as key frames in the model. [file 13007_2015_75_MOESM20_ESM.zip › front_view/side12_0200.jpg]

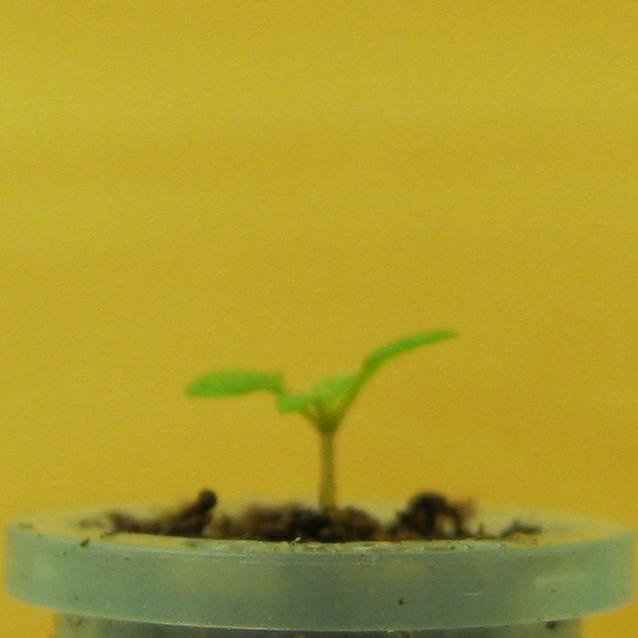

Supplement: Additional file 20 — Col-0 Front View Images for 3-D Model. Images of Col-0 captured every 10 min for 5 days from the front view for the 3-D CG model. Table S2 lists the images used as key frames in the model. [file 13007_2015_75_MOESM20_ESM.zip › front_view/side12_0201.jpg]

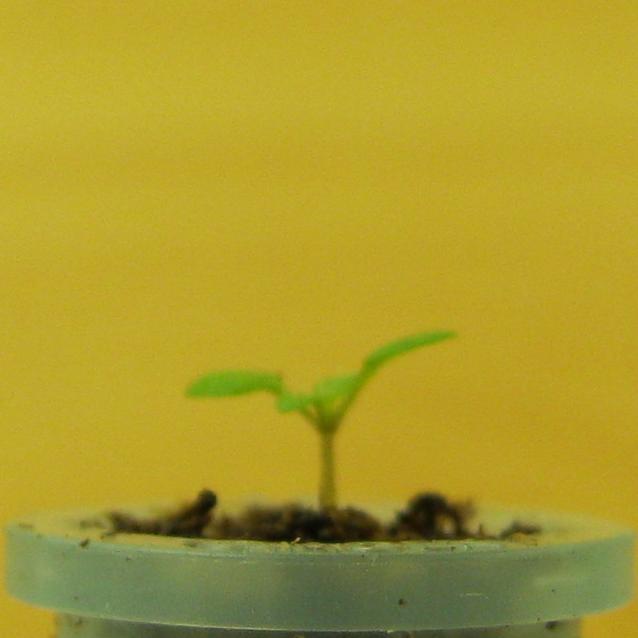

Supplement: Additional file 20 — Col-0 Front View Images for 3-D Model. Images of Col-0 captured every 10 min for 5 days from the front view for the 3-D CG model. Table S2 lists the images used as key frames in the model. [file 13007_2015_75_MOESM20_ESM.zip › front_view/side12_0202.jpg]

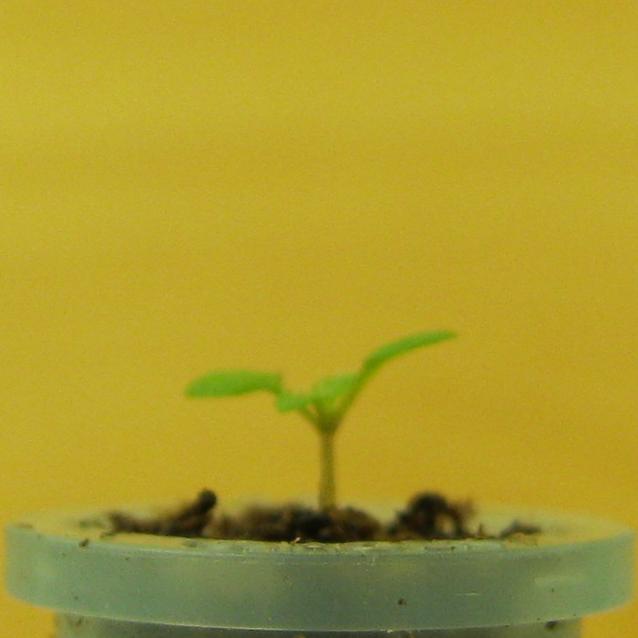

Supplement: Additional file 20 — Col-0 Front View Images for 3-D Model. Images of Col-0 captured every 10 min for 5 days from the front view for the 3-D CG model. Table S2 lists the images used as key frames in the model. [file 13007_2015_75_MOESM20_ESM.zip › front_view/side12_0203.jpg]

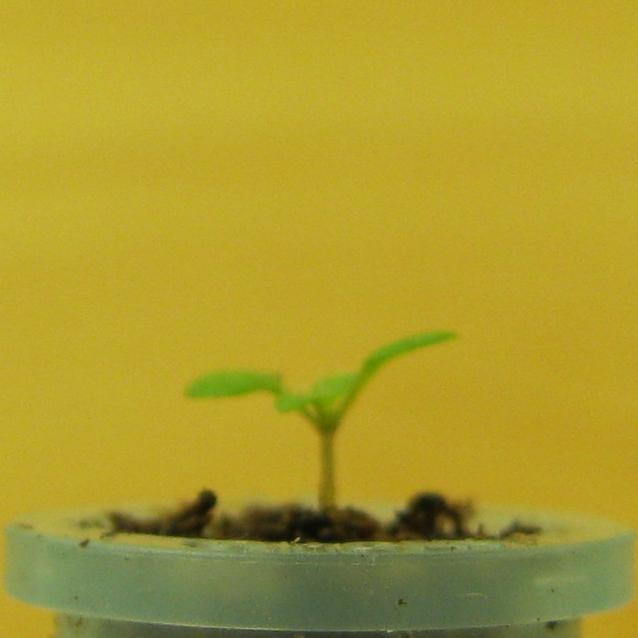

Supplement: Additional file 20 — Col-0 Front View Images for 3-D Model. Images of Col-0 captured every 10 min for 5 days from the front view for the 3-D CG model. Table S2 lists the images used as key frames in the model. [file 13007_2015_75_MOESM20_ESM.zip › front_view/side12_0204.jpg]

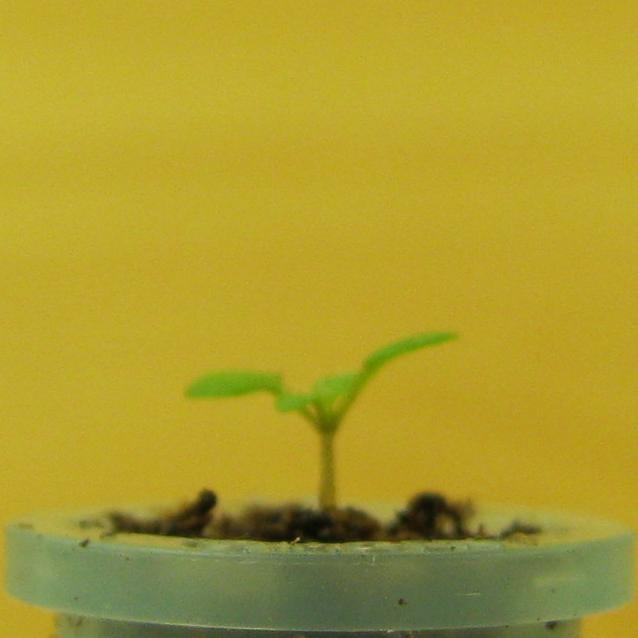

Supplement: Additional file 20 — Col-0 Front View Images for 3-D Model. Images of Col-0 captured every 10 min for 5 days from the front view for the 3-D CG model. Table S2 lists the images used as key frames in the model. [file 13007_2015_75_MOESM20_ESM.zip › front_view/side12_0205.jpg]

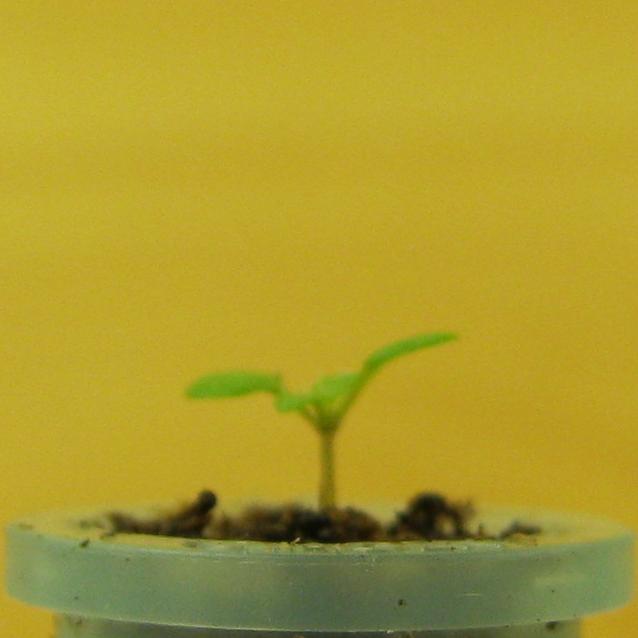

Supplement: Additional file 20 — Col-0 Front View Images for 3-D Model. Images of Col-0 captured every 10 min for 5 days from the front view for the 3-D CG model. Table S2 lists the images used as key frames in the model. [file 13007_2015_75_MOESM20_ESM.zip › front_view/side12_0206.jpg]

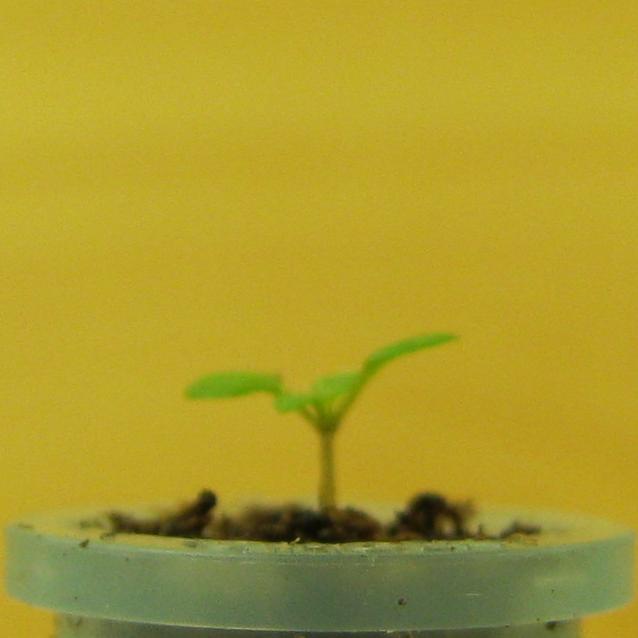

Supplement: Additional file 20 — Col-0 Front View Images for 3-D Model. Images of Col-0 captured every 10 min for 5 days from the front view for the 3-D CG model. Table S2 lists the images used as key frames in the model. [file 13007_2015_75_MOESM20_ESM.zip › front_view/side12_0207.jpg]

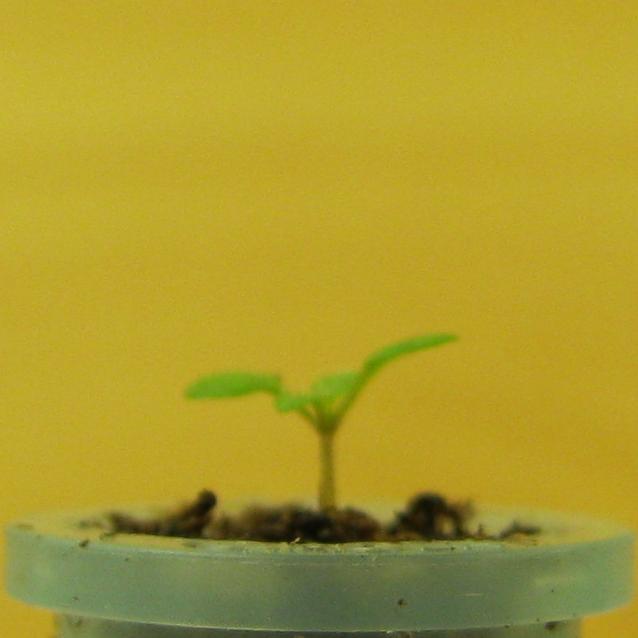

Supplement: Additional file 20 — Col-0 Front View Images for 3-D Model. Images of Col-0 captured every 10 min for 5 days from the front view for the 3-D CG model. Table S2 lists the images used as key frames in the model. [file 13007_2015_75_MOESM20_ESM.zip › front_view/side12_0208.jpg]

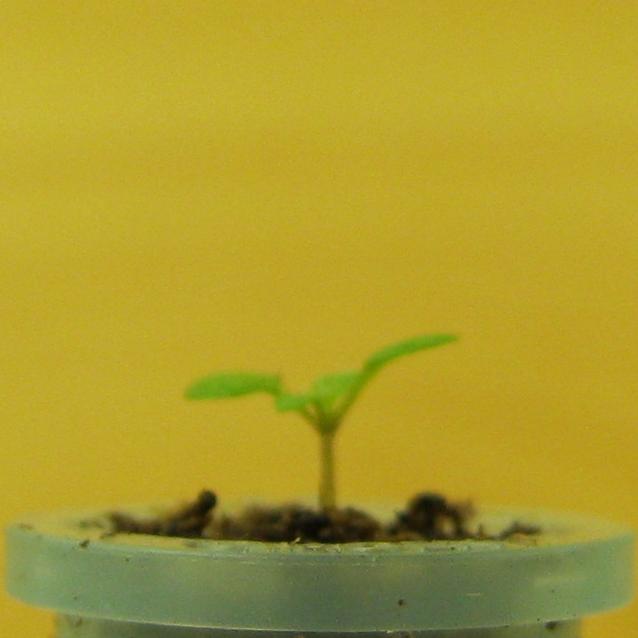

Supplement: Additional file 20 — Col-0 Front View Images for 3-D Model. Images of Col-0 captured every 10 min for 5 days from the front view for the 3-D CG model. Table S2 lists the images used as key frames in the model. [file 13007_2015_75_MOESM20_ESM.zip › front_view/side12_0209.jpg]

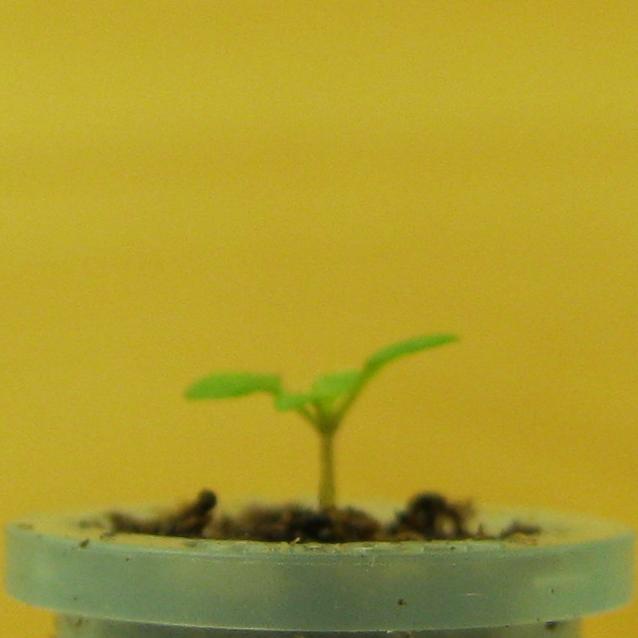

Supplement: Additional file 20 — Col-0 Front View Images for 3-D Model. Images of Col-0 captured every 10 min for 5 days from the front view for the 3-D CG model. Table S2 lists the images used as key frames in the model. [file 13007_2015_75_MOESM20_ESM.zip › front_view/side12_0210.jpg]

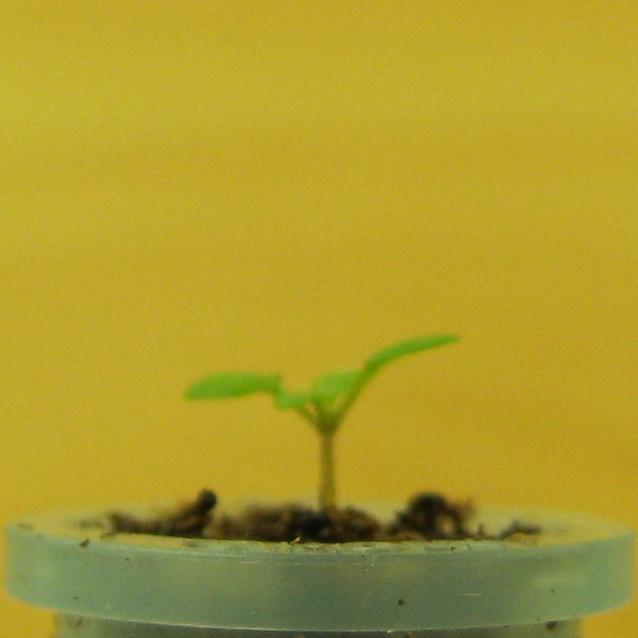

Supplement: Additional file 20 — Col-0 Front View Images for 3-D Model. Images of Col-0 captured every 10 min for 5 days from the front view for the 3-D CG model. Table S2 lists the images used as key frames in the model. [file 13007_2015_75_MOESM20_ESM.zip › front_view/side12_0211.jpg]

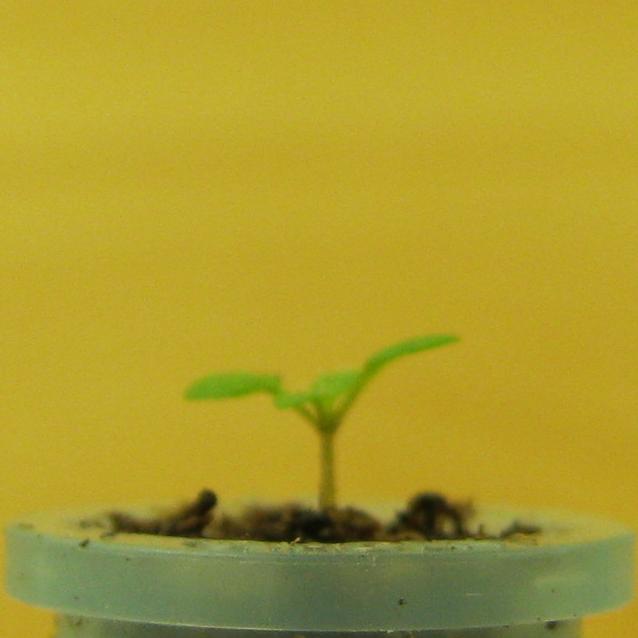

Supplement: Additional file 20 — Col-0 Front View Images for 3-D Model. Images of Col-0 captured every 10 min for 5 days from the front view for the 3-D CG model. Table S2 lists the images used as key frames in the model. [file 13007_2015_75_MOESM20_ESM.zip › front_view/side12_0212.jpg]

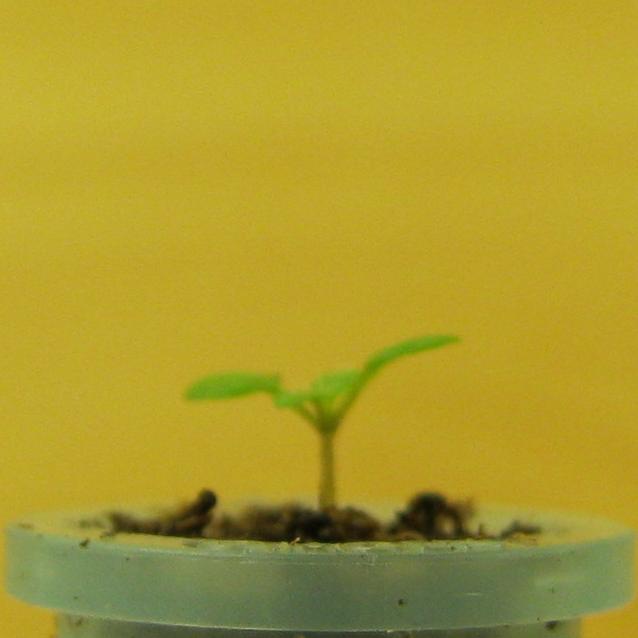

Supplement: Additional file 20 — Col-0 Front View Images for 3-D Model. Images of Col-0 captured every 10 min for 5 days from the front view for the 3-D CG model. Table S2 lists the images used as key frames in the model. [file 13007_2015_75_MOESM20_ESM.zip › front_view/side12_0213.jpg]

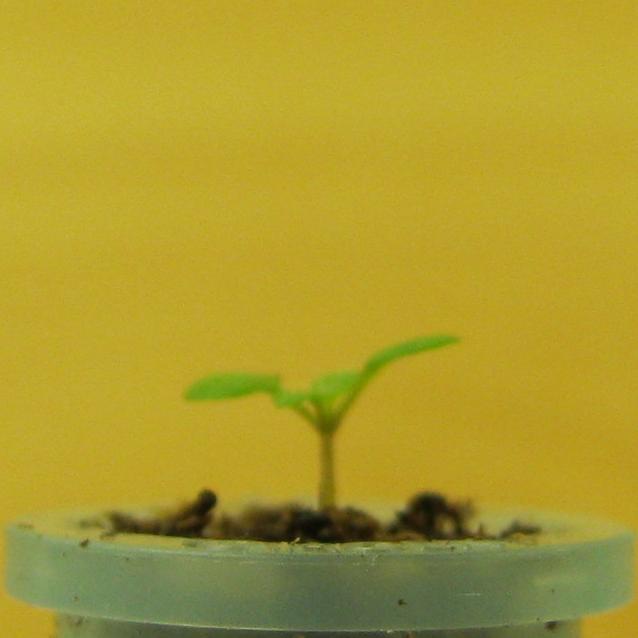

Supplement: Additional file 20 — Col-0 Front View Images for 3-D Model. Images of Col-0 captured every 10 min for 5 days from the front view for the 3-D CG model. Table S2 lists the images used as key frames in the model. [file 13007_2015_75_MOESM20_ESM.zip › front_view/side12_0214.jpg]

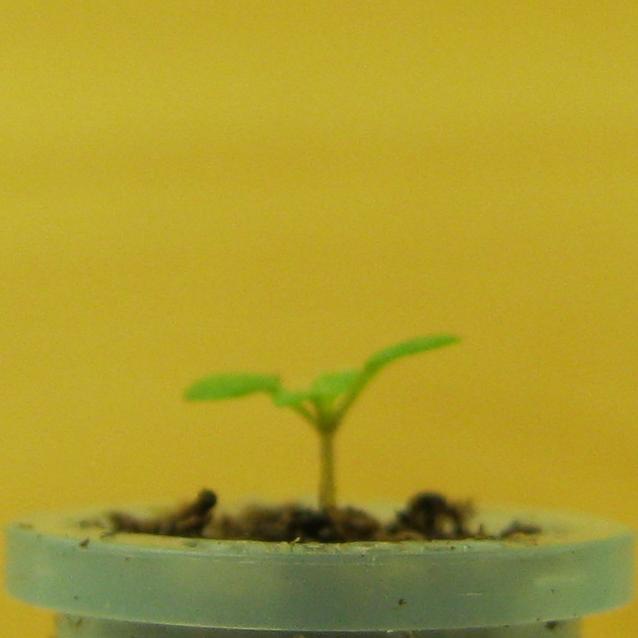

Supplement: Additional file 20 — Col-0 Front View Images for 3-D Model. Images of Col-0 captured every 10 min for 5 days from the front view for the 3-D CG model. Table S2 lists the images used as key frames in the model. [file 13007_2015_75_MOESM20_ESM.zip › front_view/side12_0215.jpg]

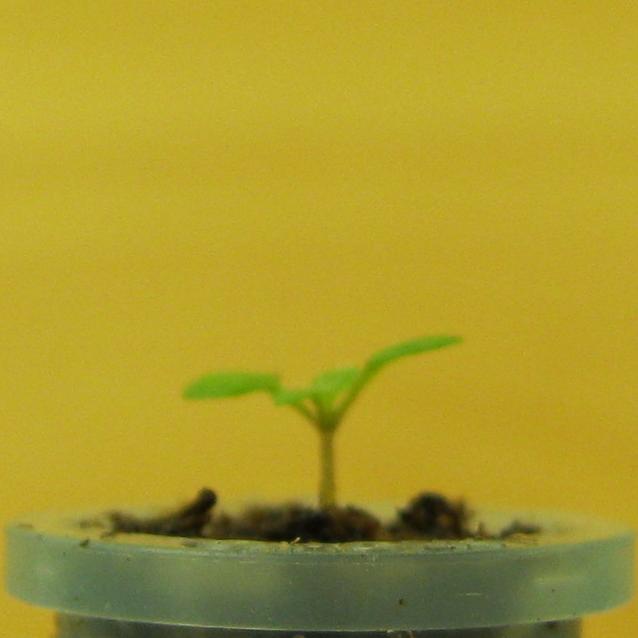

Supplement: Additional file 20 — Col-0 Front View Images for 3-D Model. Images of Col-0 captured every 10 min for 5 days from the front view for the 3-D CG model. Table S2 lists the images used as key frames in the model. [file 13007_2015_75_MOESM20_ESM.zip › front_view/side12_0216.jpg]

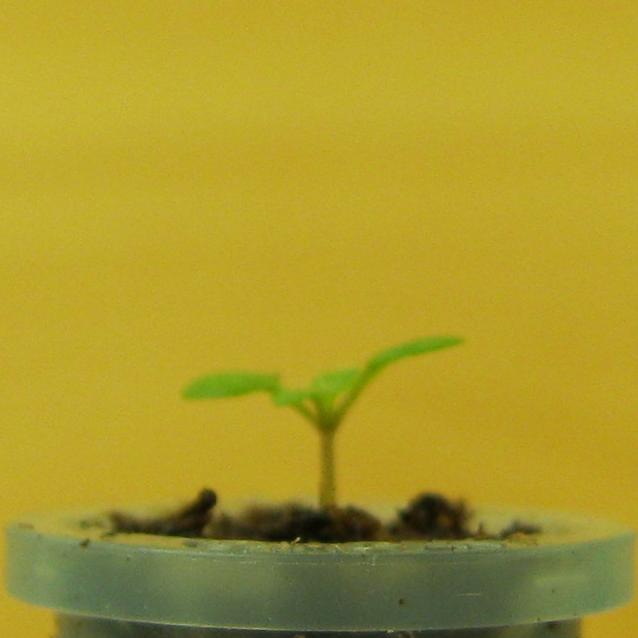

Supplement: Additional file 20 — Col-0 Front View Images for 3-D Model. Images of Col-0 captured every 10 min for 5 days from the front view for the 3-D CG model. Table S2 lists the images used as key frames in the model. [file 13007_2015_75_MOESM20_ESM.zip › front_view/side12_0217.jpg]

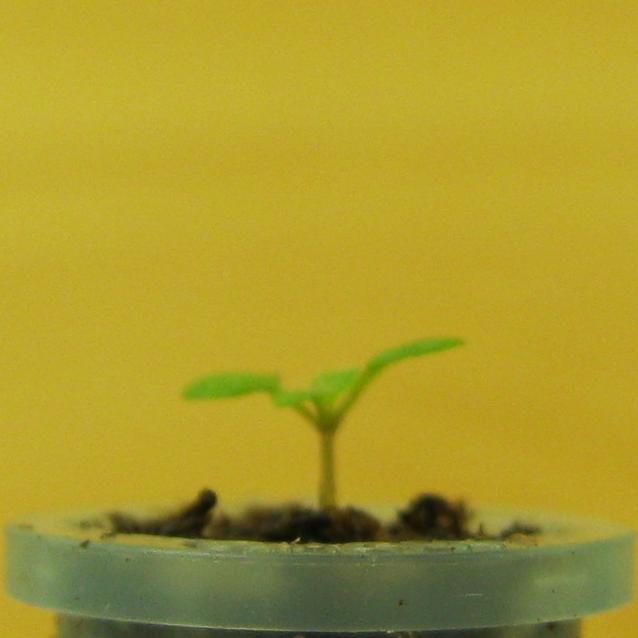

Supplement: Additional file 20 — Col-0 Front View Images for 3-D Model. Images of Col-0 captured every 10 min for 5 days from the front view for the 3-D CG model. Table S2 lists the images used as key frames in the model. [file 13007_2015_75_MOESM20_ESM.zip › front_view/side12_0218.jpg]

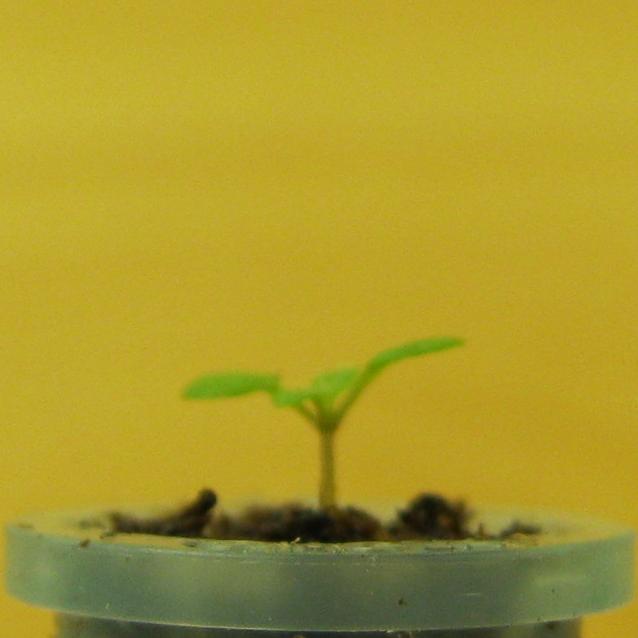

Supplement: Additional file 20 — Col-0 Front View Images for 3-D Model. Images of Col-0 captured every 10 min for 5 days from the front view for the 3-D CG model. Table S2 lists the images used as key frames in the model. [file 13007_2015_75_MOESM20_ESM.zip › front_view/side12_0219.jpg]

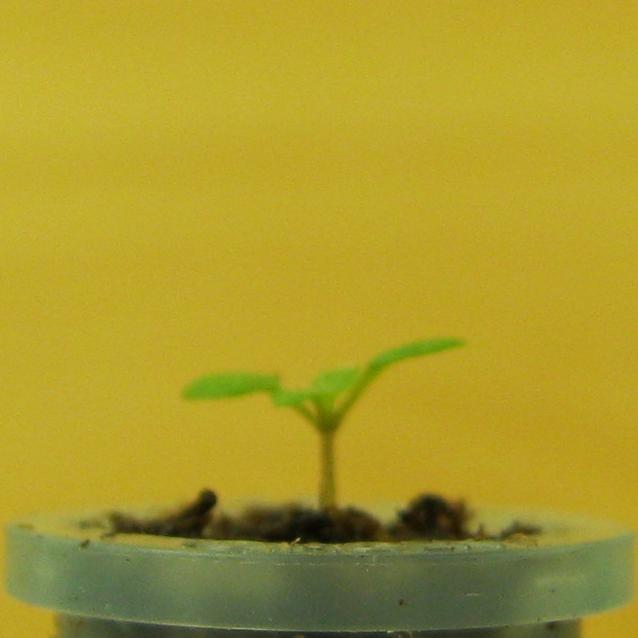

Supplement: Additional file 20 — Col-0 Front View Images for 3-D Model. Images of Col-0 captured every 10 min for 5 days from the front view for the 3-D CG model. Table S2 lists the images used as key frames in the model. [file 13007_2015_75_MOESM20_ESM.zip › front_view/side12_0220.jpg]

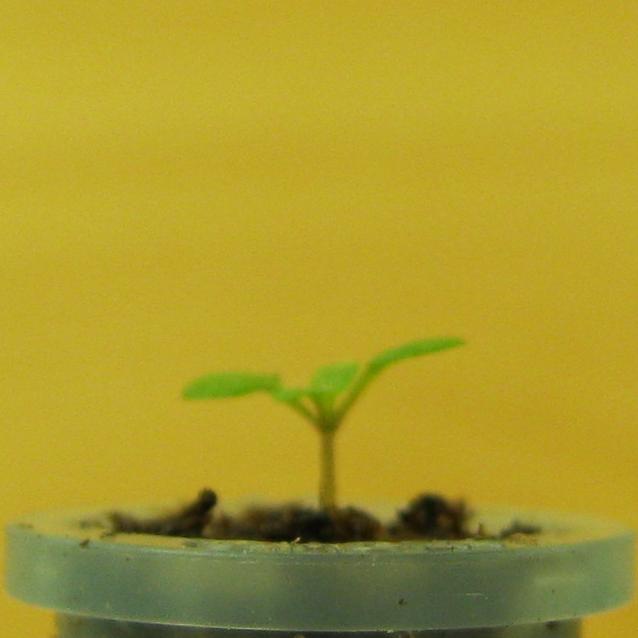

Supplement: Additional file 20 — Col-0 Front View Images for 3-D Model. Images of Col-0 captured every 10 min for 5 days from the front view for the 3-D CG model. Table S2 lists the images used as key frames in the model. [file 13007_2015_75_MOESM20_ESM.zip › front_view/side12_0221.jpg]

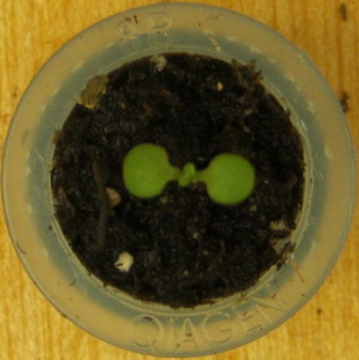

Supplement: Additional file 21 — Col-0 Top View Images for 3-D Model. First half of images of Col-0 captured every 10 min for 5 days from the top view for the 3-D CG model. Table S2 lists the images used as key frames in the model. [file 13007_2015_75_MOESM21_ESM.zip › top_view_1/top_0001.jpg]

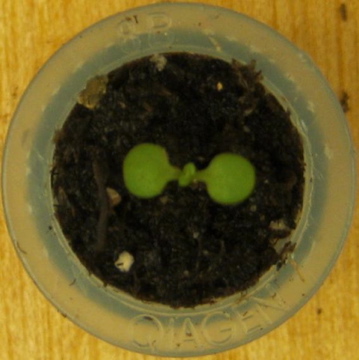

Supplement: Additional file 21 — Col-0 Top View Images for 3-D Model. First half of images of Col-0 captured every 10 min for 5 days from the top view for the 3-D CG model. Table S2 lists the images used as key frames in the model. [file 13007_2015_75_MOESM21_ESM.zip › top_view_1/top_0002.jpg]

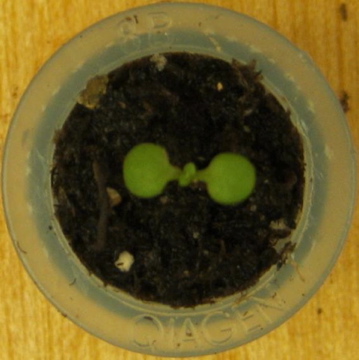

Supplement: Additional file 21 — Col-0 Top View Images for 3-D Model. First half of images of Col-0 captured every 10 min for 5 days from the top view for the 3-D CG model. Table S2 lists the images used as key frames in the model. [file 13007_2015_75_MOESM21_ESM.zip › top_view_1/top_0003.jpg]

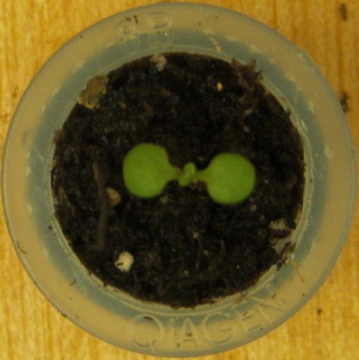

Supplement: Additional file 21 — Col-0 Top View Images for 3-D Model. First half of images of Col-0 captured every 10 min for 5 days from the top view for the 3-D CG model. Table S2 lists the images used as key frames in the model. [file 13007_2015_75_MOESM21_ESM.zip › top_view_1/top_0004.jpg]

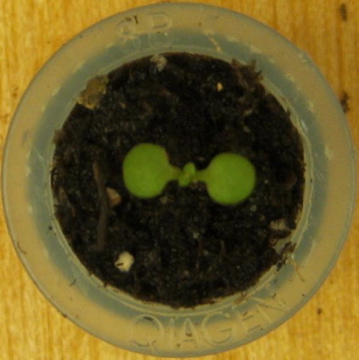

Supplement: Additional file 21 — Col-0 Top View Images for 3-D Model. First half of images of Col-0 captured every 10 min for 5 days from the top view for the 3-D CG model. Table S2 lists the images used as key frames in the model. [file 13007_2015_75_MOESM21_ESM.zip › top_view_1/top_0005.jpg]

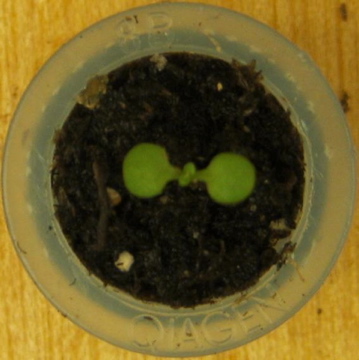

Supplement: Additional file 21 — Col-0 Top View Images for 3-D Model. First half of images of Col-0 captured every 10 min for 5 days from the top view for the 3-D CG model. Table S2 lists the images used as key frames in the model. [file 13007_2015_75_MOESM21_ESM.zip › top_view_1/top_0006.jpg]

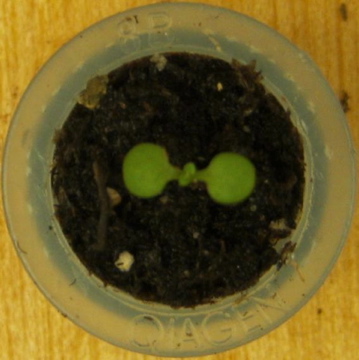

Supplement: Additional file 21 — Col-0 Top View Images for 3-D Model. First half of images of Col-0 captured every 10 min for 5 days from the top view for the 3-D CG model. Table S2 lists the images used as key frames in the model. [file 13007_2015_75_MOESM21_ESM.zip › top_view_1/top_0007.jpg]

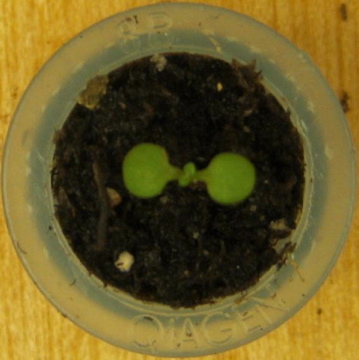

Supplement: Additional file 21 — Col-0 Top View Images for 3-D Model. First half of images of Col-0 captured every 10 min for 5 days from the top view for the 3-D CG model. Table S2 lists the images used as key frames in the model. [file 13007_2015_75_MOESM21_ESM.zip › top_view_1/top_0008.jpg]
