# Supplementary figures and images for: TRiP: Tracking Rhythms in Plants, an automated leaf movement analysis program for circadian period estimation (part 5 of 10)
Source: Plant Methods. 2015 May 3;11:33. doi: 10.1186/s13007-015-0075-5 (PMC4445800; doi:10.1186/s13007-015-0075-5)

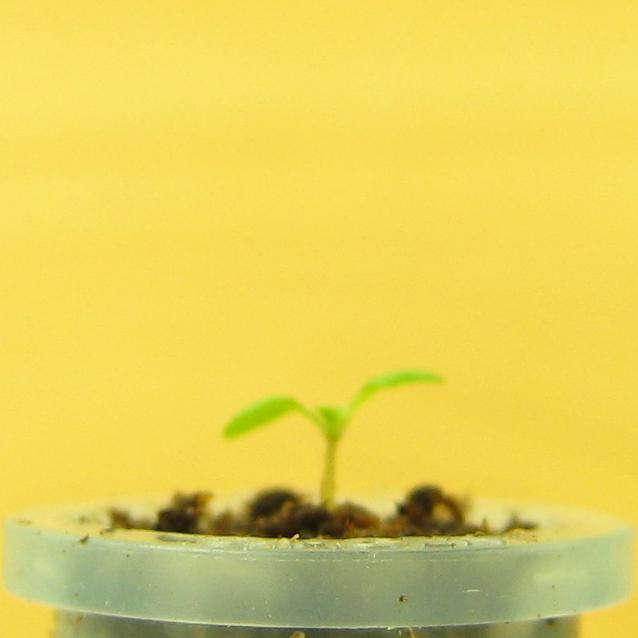

Supplement: Additional file 20 — Col-0 Front View Images for 3-D Model. Images of Col-0 captured every 10 min for 5 days from the front view for the 3-D CG model. Table S2 lists the images used as key frames in the model. [file 13007_2015_75_MOESM20_ESM.zip › front_view/side12_0009.jpg]

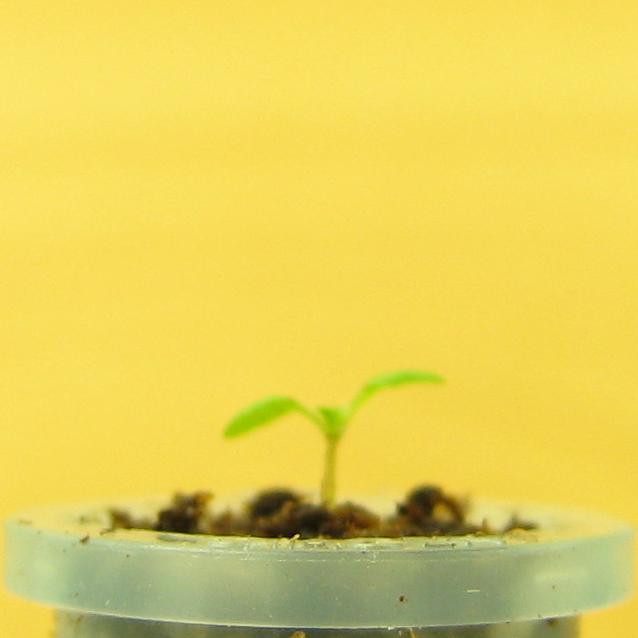

Supplement: Additional file 20 — Col-0 Front View Images for 3-D Model. Images of Col-0 captured every 10 min for 5 days from the front view for the 3-D CG model. Table S2 lists the images used as key frames in the model. [file 13007_2015_75_MOESM20_ESM.zip › front_view/side12_0010.jpg]

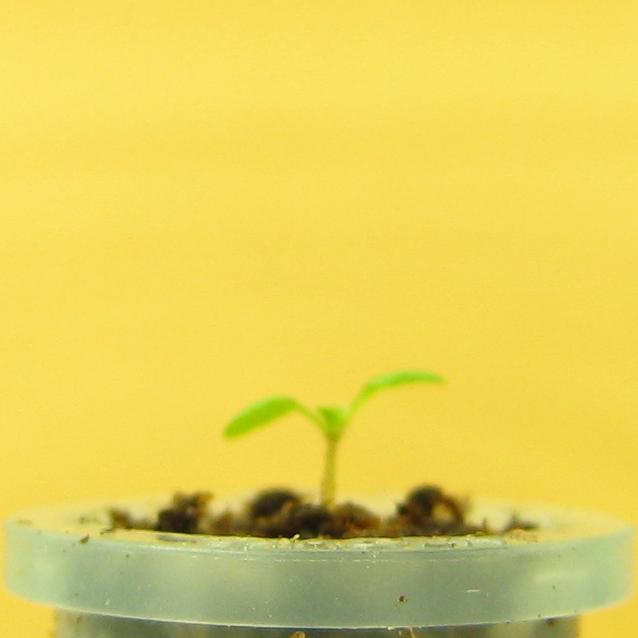

Supplement: Additional file 20 — Col-0 Front View Images for 3-D Model. Images of Col-0 captured every 10 min for 5 days from the front view for the 3-D CG model. Table S2 lists the images used as key frames in the model. [file 13007_2015_75_MOESM20_ESM.zip › front_view/side12_0011.jpg]

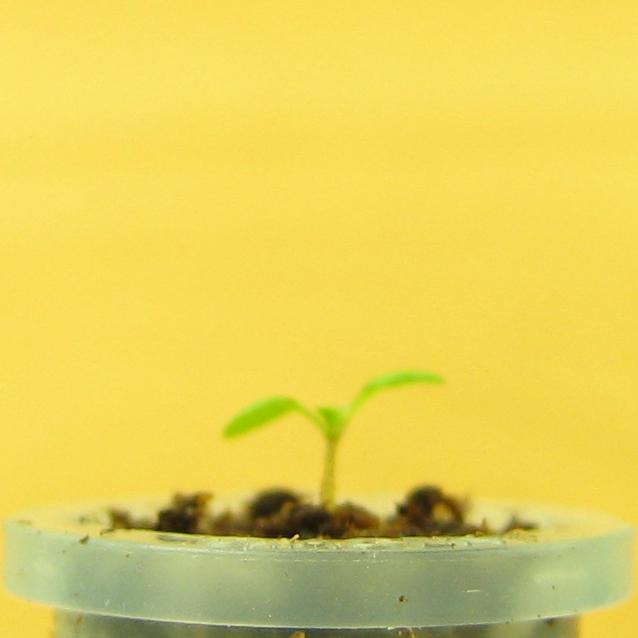

Supplement: Additional file 20 — Col-0 Front View Images for 3-D Model. Images of Col-0 captured every 10 min for 5 days from the front view for the 3-D CG model. Table S2 lists the images used as key frames in the model. [file 13007_2015_75_MOESM20_ESM.zip › front_view/side12_0012.jpg]

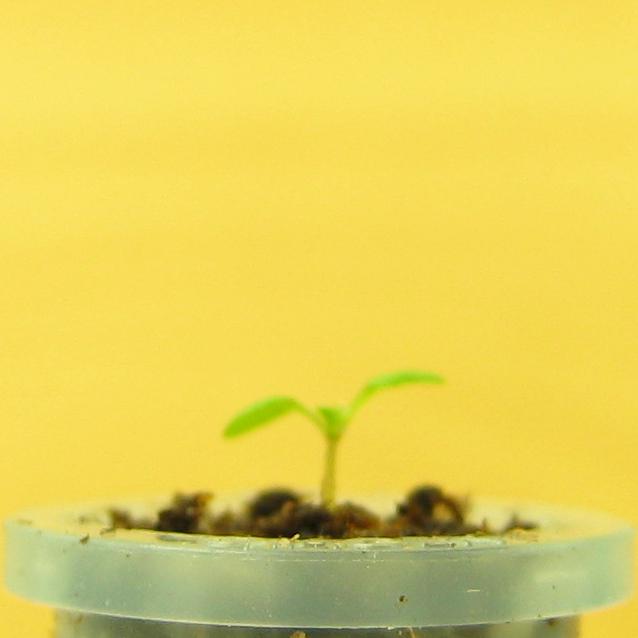

Supplement: Additional file 20 — Col-0 Front View Images for 3-D Model. Images of Col-0 captured every 10 min for 5 days from the front view for the 3-D CG model. Table S2 lists the images used as key frames in the model. [file 13007_2015_75_MOESM20_ESM.zip › front_view/side12_0013.jpg]

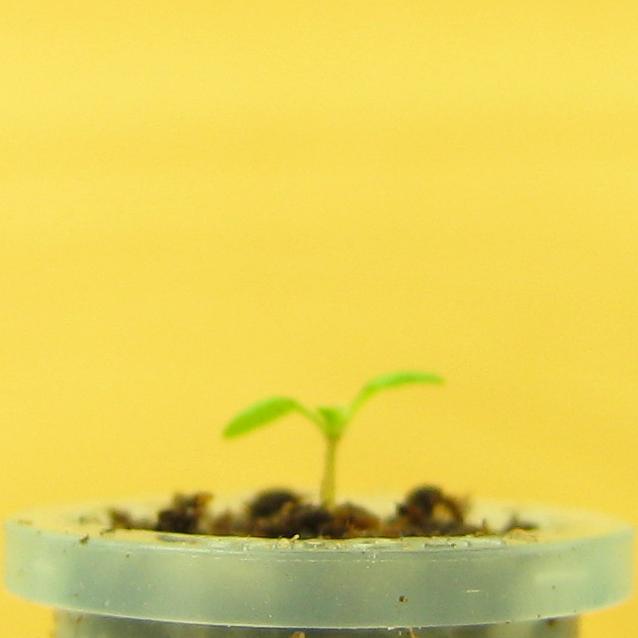

Supplement: Additional file 20 — Col-0 Front View Images for 3-D Model. Images of Col-0 captured every 10 min for 5 days from the front view for the 3-D CG model. Table S2 lists the images used as key frames in the model. [file 13007_2015_75_MOESM20_ESM.zip › front_view/side12_0014.jpg]

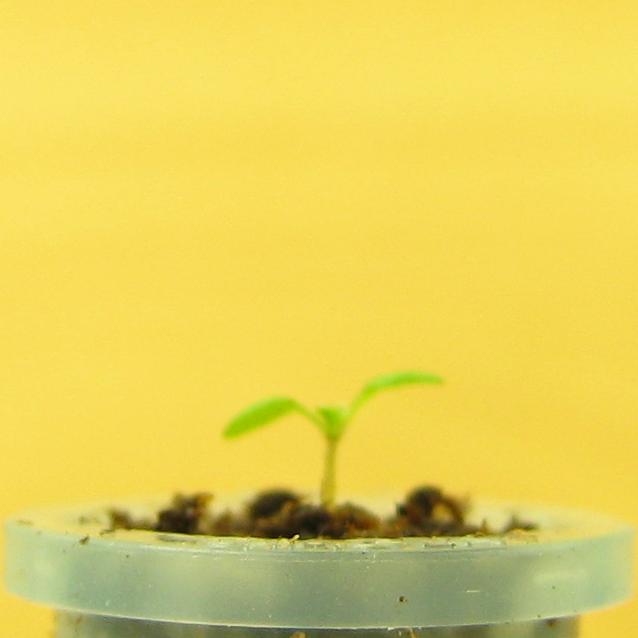

Supplement: Additional file 20 — Col-0 Front View Images for 3-D Model. Images of Col-0 captured every 10 min for 5 days from the front view for the 3-D CG model. Table S2 lists the images used as key frames in the model. [file 13007_2015_75_MOESM20_ESM.zip › front_view/side12_0015.jpg]

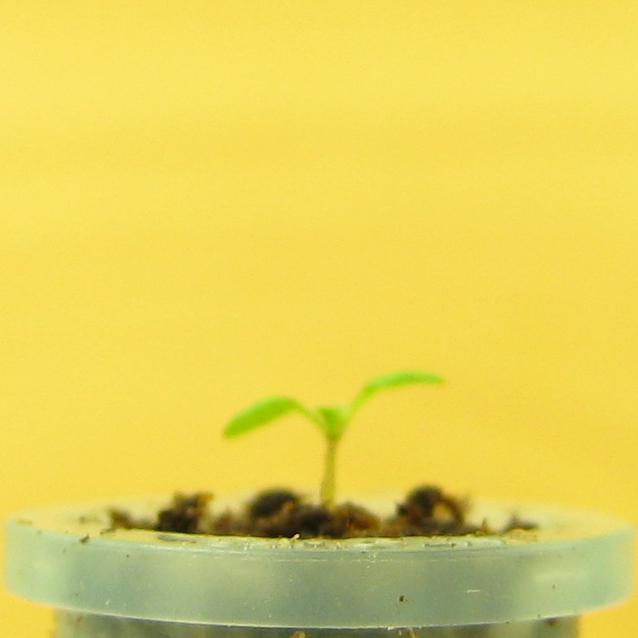

Supplement: Additional file 20 — Col-0 Front View Images for 3-D Model. Images of Col-0 captured every 10 min for 5 days from the front view for the 3-D CG model. Table S2 lists the images used as key frames in the model. [file 13007_2015_75_MOESM20_ESM.zip › front_view/side12_0016.jpg]

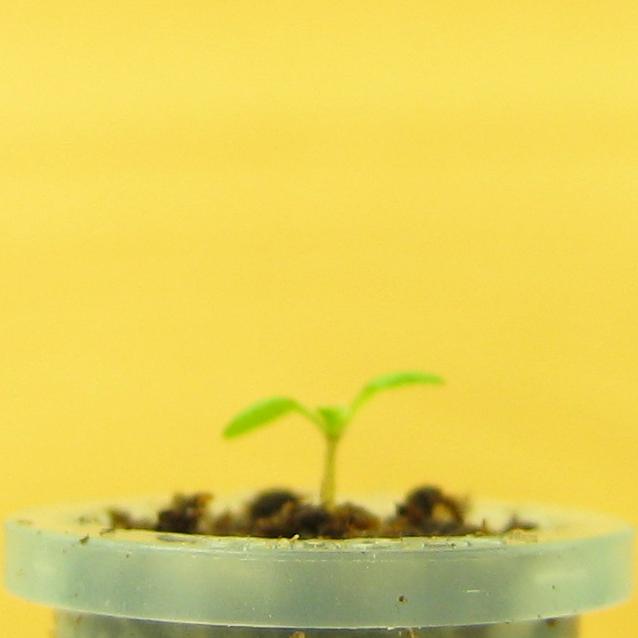

Supplement: Additional file 20 — Col-0 Front View Images for 3-D Model. Images of Col-0 captured every 10 min for 5 days from the front view for the 3-D CG model. Table S2 lists the images used as key frames in the model. [file 13007_2015_75_MOESM20_ESM.zip › front_view/side12_0017.jpg]

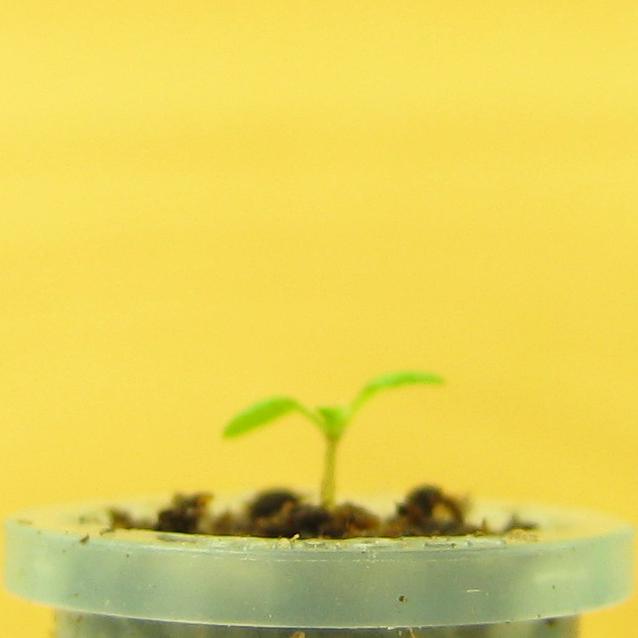

Supplement: Additional file 20 — Col-0 Front View Images for 3-D Model. Images of Col-0 captured every 10 min for 5 days from the front view for the 3-D CG model. Table S2 lists the images used as key frames in the model. [file 13007_2015_75_MOESM20_ESM.zip › front_view/side12_0018.jpg]

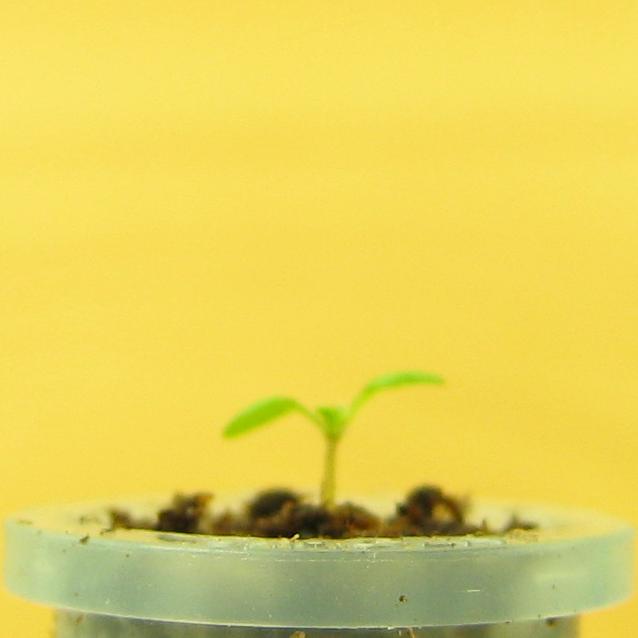

Supplement: Additional file 20 — Col-0 Front View Images for 3-D Model. Images of Col-0 captured every 10 min for 5 days from the front view for the 3-D CG model. Table S2 lists the images used as key frames in the model. [file 13007_2015_75_MOESM20_ESM.zip › front_view/side12_0019.jpg]

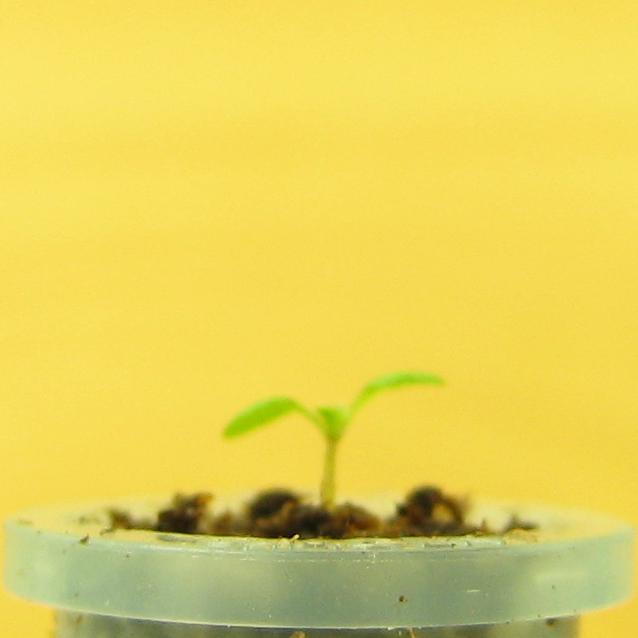

Supplement: Additional file 20 — Col-0 Front View Images for 3-D Model. Images of Col-0 captured every 10 min for 5 days from the front view for the 3-D CG model. Table S2 lists the images used as key frames in the model. [file 13007_2015_75_MOESM20_ESM.zip › front_view/side12_0020.jpg]

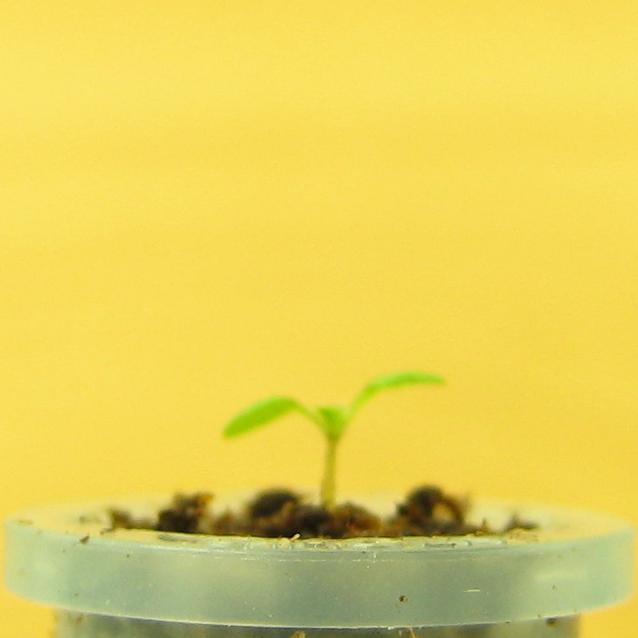

Supplement: Additional file 20 — Col-0 Front View Images for 3-D Model. Images of Col-0 captured every 10 min for 5 days from the front view for the 3-D CG model. Table S2 lists the images used as key frames in the model. [file 13007_2015_75_MOESM20_ESM.zip › front_view/side12_0021.jpg]

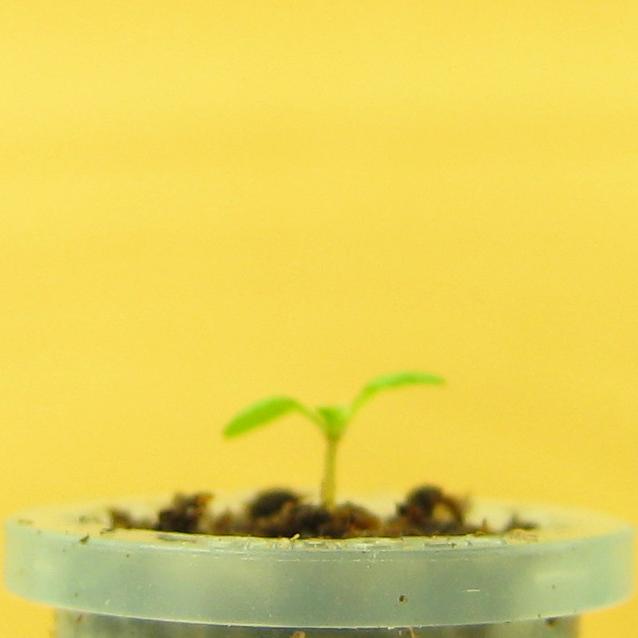

Supplement: Additional file 20 — Col-0 Front View Images for 3-D Model. Images of Col-0 captured every 10 min for 5 days from the front view for the 3-D CG model. Table S2 lists the images used as key frames in the model. [file 13007_2015_75_MOESM20_ESM.zip › front_view/side12_0022.jpg]

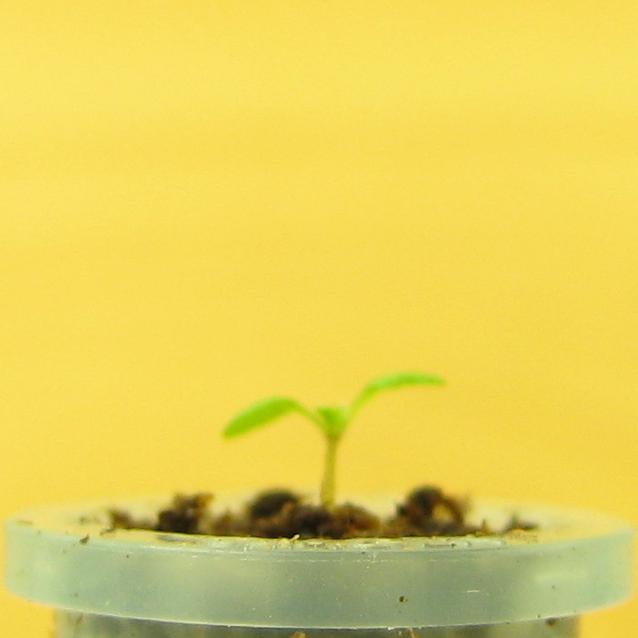

Supplement: Additional file 20 — Col-0 Front View Images for 3-D Model. Images of Col-0 captured every 10 min for 5 days from the front view for the 3-D CG model. Table S2 lists the images used as key frames in the model. [file 13007_2015_75_MOESM20_ESM.zip › front_view/side12_0023.jpg]

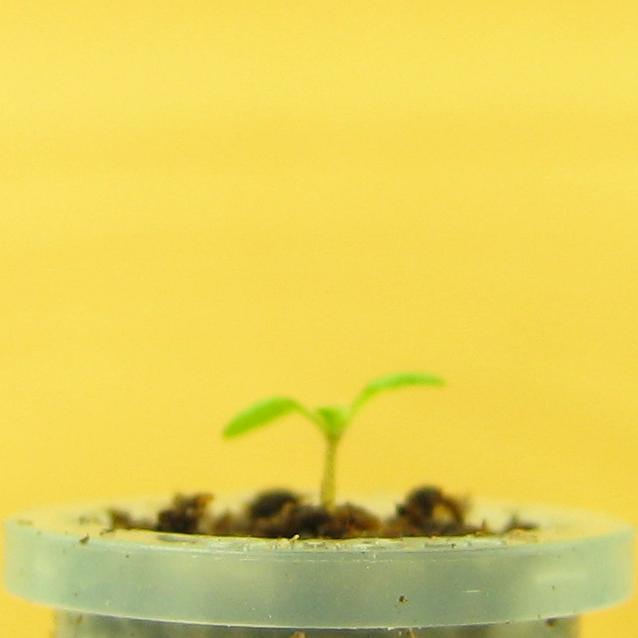

Supplement: Additional file 20 — Col-0 Front View Images for 3-D Model. Images of Col-0 captured every 10 min for 5 days from the front view for the 3-D CG model. Table S2 lists the images used as key frames in the model. [file 13007_2015_75_MOESM20_ESM.zip › front_view/side12_0024.jpg]

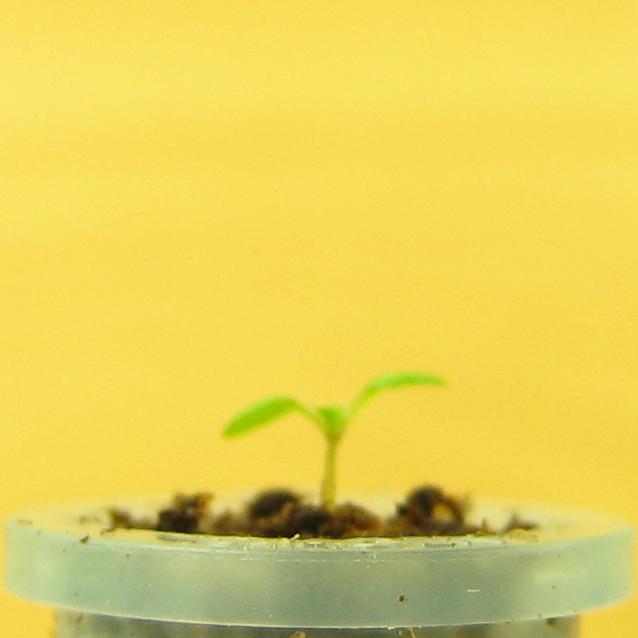

Supplement: Additional file 20 — Col-0 Front View Images for 3-D Model. Images of Col-0 captured every 10 min for 5 days from the front view for the 3-D CG model. Table S2 lists the images used as key frames in the model. [file 13007_2015_75_MOESM20_ESM.zip › front_view/side12_0025.jpg]

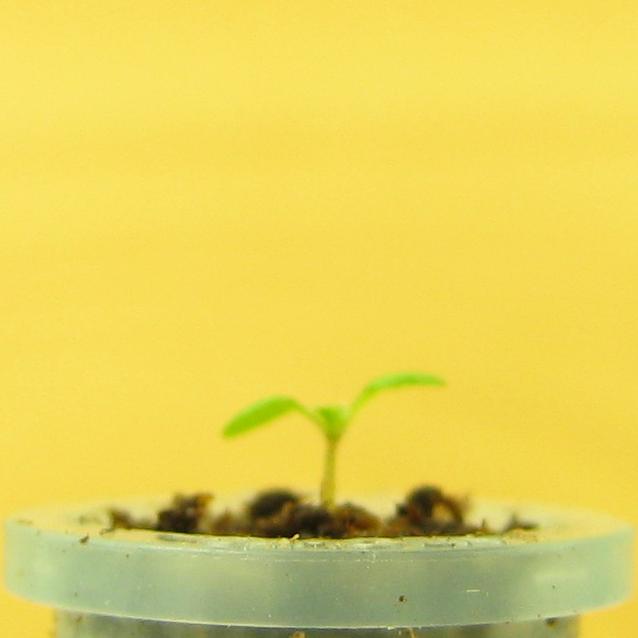

Supplement: Additional file 20 — Col-0 Front View Images for 3-D Model. Images of Col-0 captured every 10 min for 5 days from the front view for the 3-D CG model. Table S2 lists the images used as key frames in the model. [file 13007_2015_75_MOESM20_ESM.zip › front_view/side12_0026.jpg]

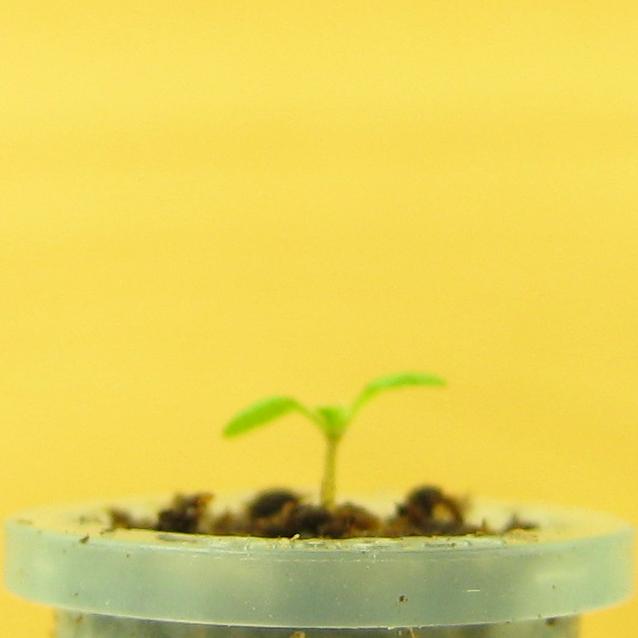

Supplement: Additional file 20 — Col-0 Front View Images for 3-D Model. Images of Col-0 captured every 10 min for 5 days from the front view for the 3-D CG model. Table S2 lists the images used as key frames in the model. [file 13007_2015_75_MOESM20_ESM.zip › front_view/side12_0027.jpg]

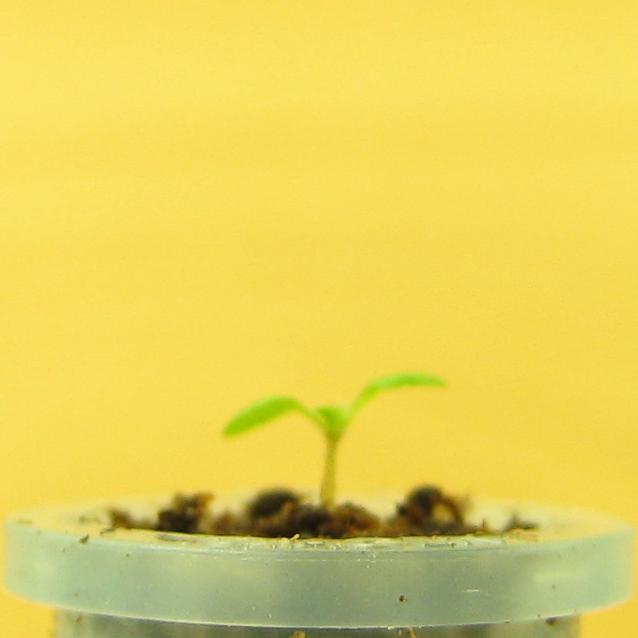

Supplement: Additional file 20 — Col-0 Front View Images for 3-D Model. Images of Col-0 captured every 10 min for 5 days from the front view for the 3-D CG model. Table S2 lists the images used as key frames in the model. [file 13007_2015_75_MOESM20_ESM.zip › front_view/side12_0028.jpg]

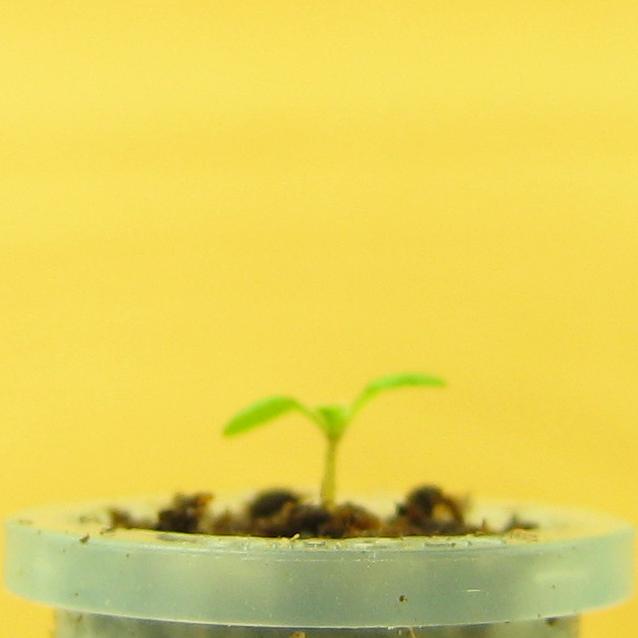

Supplement: Additional file 20 — Col-0 Front View Images for 3-D Model. Images of Col-0 captured every 10 min for 5 days from the front view for the 3-D CG model. Table S2 lists the images used as key frames in the model. [file 13007_2015_75_MOESM20_ESM.zip › front_view/side12_0029.jpg]

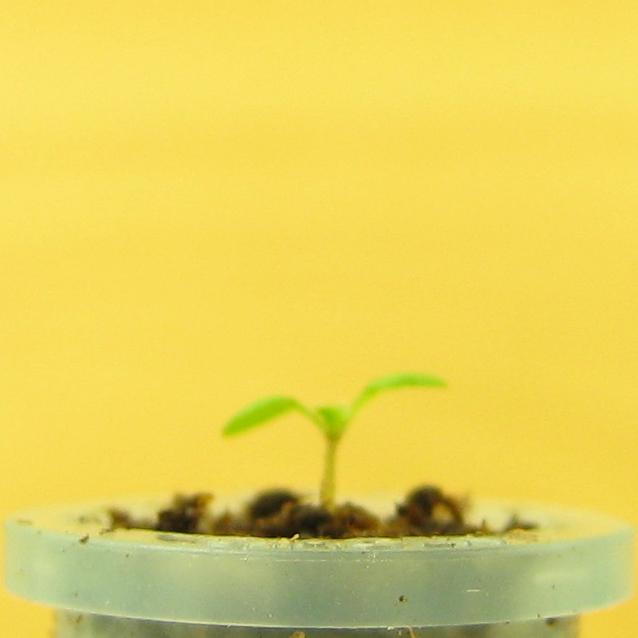

Supplement: Additional file 20 — Col-0 Front View Images for 3-D Model. Images of Col-0 captured every 10 min for 5 days from the front view for the 3-D CG model. Table S2 lists the images used as key frames in the model. [file 13007_2015_75_MOESM20_ESM.zip › front_view/side12_0030.jpg]

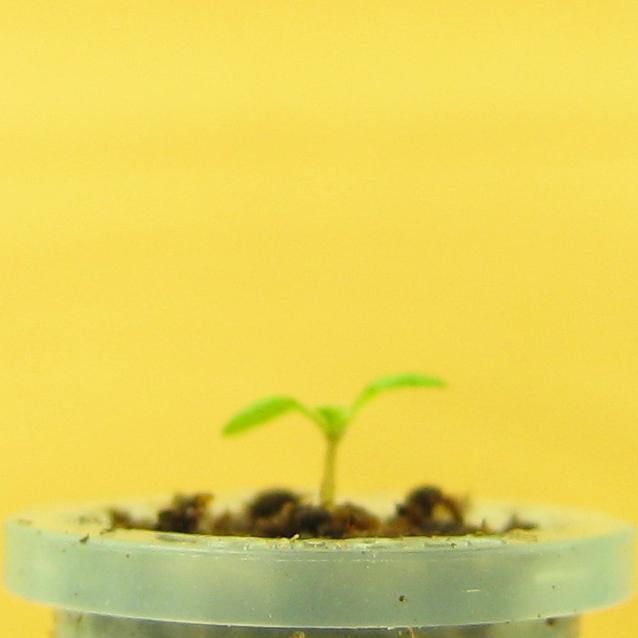

Supplement: Additional file 20 — Col-0 Front View Images for 3-D Model. Images of Col-0 captured every 10 min for 5 days from the front view for the 3-D CG model. Table S2 lists the images used as key frames in the model. [file 13007_2015_75_MOESM20_ESM.zip › front_view/side12_0031.jpg]

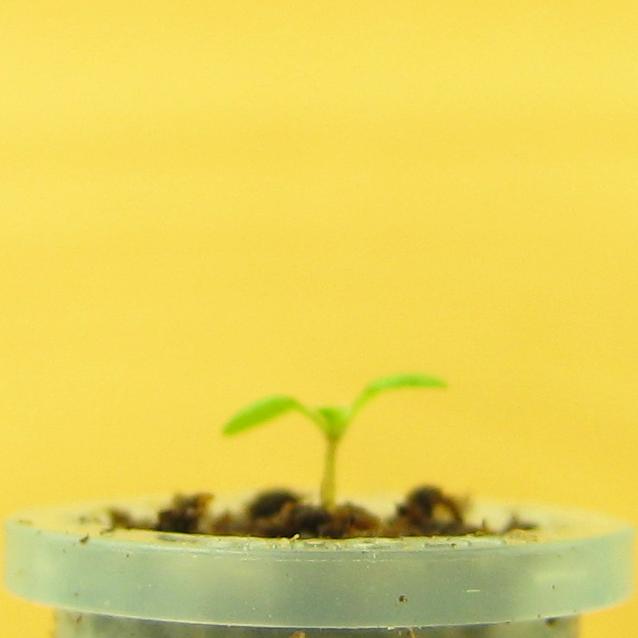

Supplement: Additional file 20 — Col-0 Front View Images for 3-D Model. Images of Col-0 captured every 10 min for 5 days from the front view for the 3-D CG model. Table S2 lists the images used as key frames in the model. [file 13007_2015_75_MOESM20_ESM.zip › front_view/side12_0032.jpg]

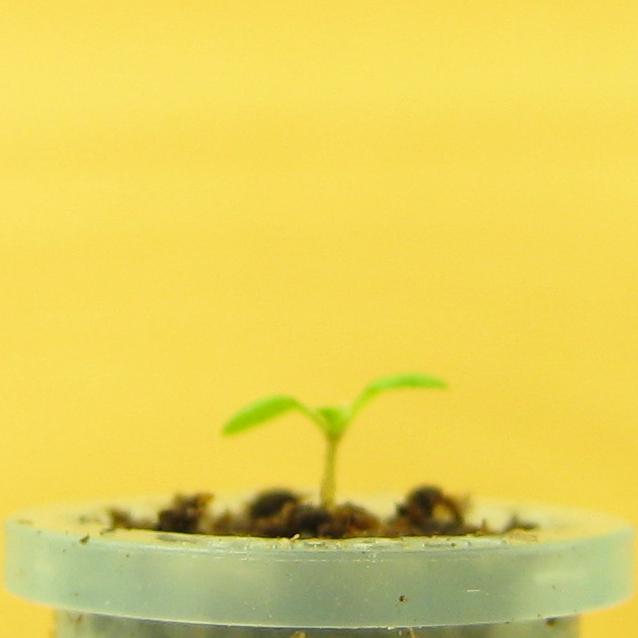

Supplement: Additional file 20 — Col-0 Front View Images for 3-D Model. Images of Col-0 captured every 10 min for 5 days from the front view for the 3-D CG model. Table S2 lists the images used as key frames in the model. [file 13007_2015_75_MOESM20_ESM.zip › front_view/side12_0033.jpg]

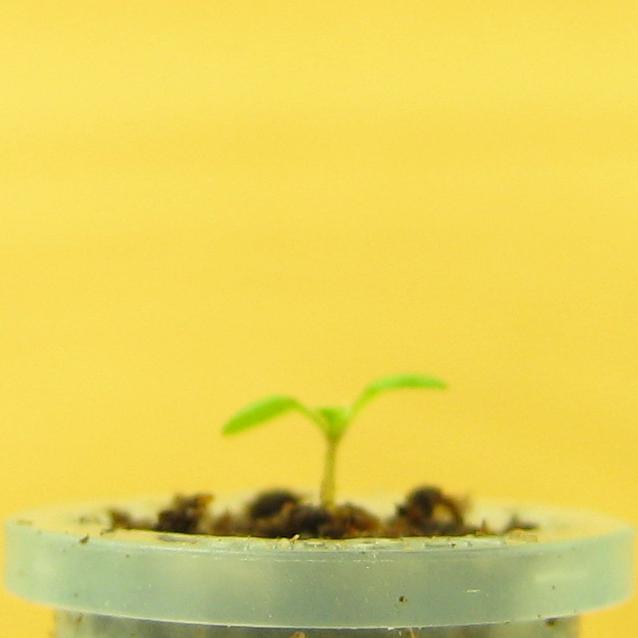

Supplement: Additional file 20 — Col-0 Front View Images for 3-D Model. Images of Col-0 captured every 10 min for 5 days from the front view for the 3-D CG model. Table S2 lists the images used as key frames in the model. [file 13007_2015_75_MOESM20_ESM.zip › front_view/side12_0034.jpg]

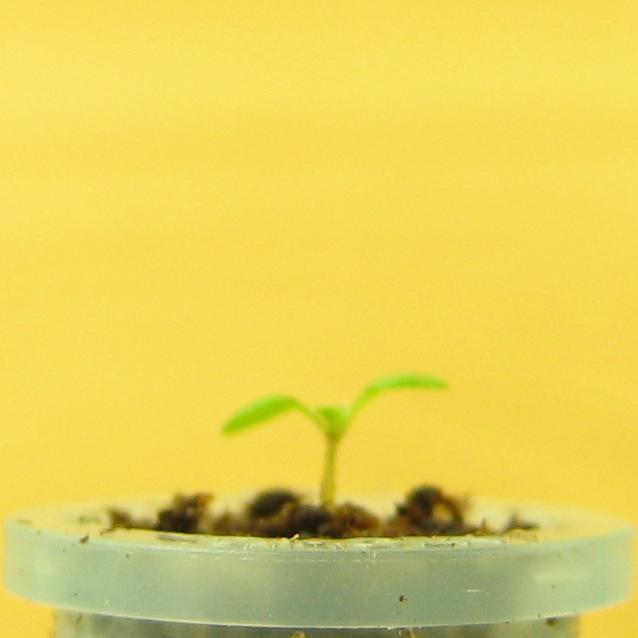

Supplement: Additional file 20 — Col-0 Front View Images for 3-D Model. Images of Col-0 captured every 10 min for 5 days from the front view for the 3-D CG model. Table S2 lists the images used as key frames in the model. [file 13007_2015_75_MOESM20_ESM.zip › front_view/side12_0035.jpg]

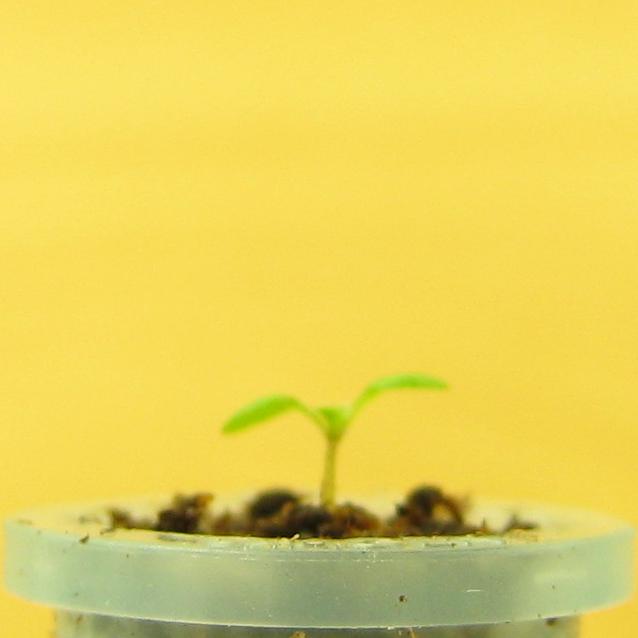

Supplement: Additional file 20 — Col-0 Front View Images for 3-D Model. Images of Col-0 captured every 10 min for 5 days from the front view for the 3-D CG model. Table S2 lists the images used as key frames in the model. [file 13007_2015_75_MOESM20_ESM.zip › front_view/side12_0036.jpg]

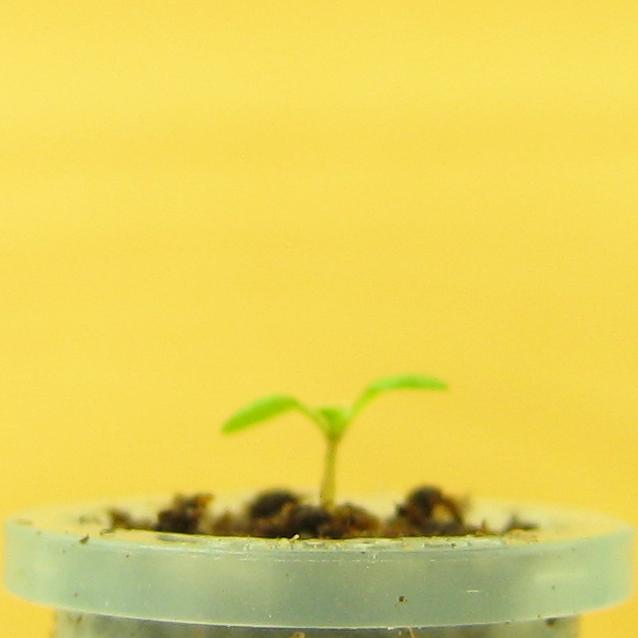

Supplement: Additional file 20 — Col-0 Front View Images for 3-D Model. Images of Col-0 captured every 10 min for 5 days from the front view for the 3-D CG model. Table S2 lists the images used as key frames in the model. [file 13007_2015_75_MOESM20_ESM.zip › front_view/side12_0037.jpg]

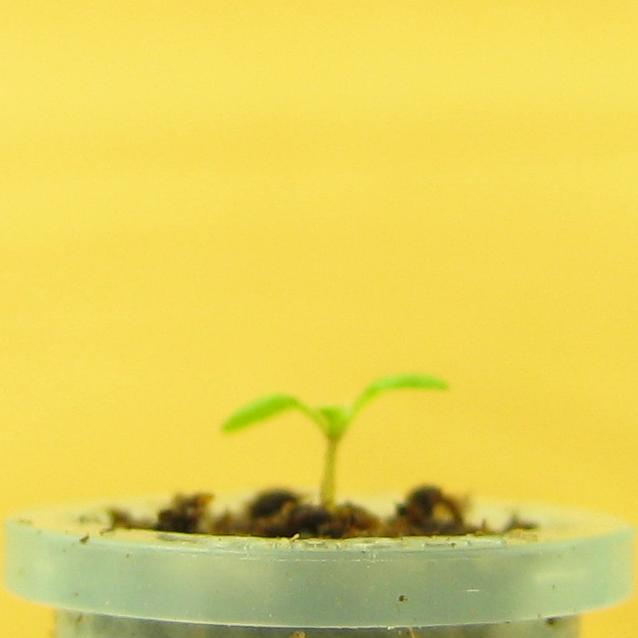

Supplement: Additional file 20 — Col-0 Front View Images for 3-D Model. Images of Col-0 captured every 10 min for 5 days from the front view for the 3-D CG model. Table S2 lists the images used as key frames in the model. [file 13007_2015_75_MOESM20_ESM.zip › front_view/side12_0038.jpg]

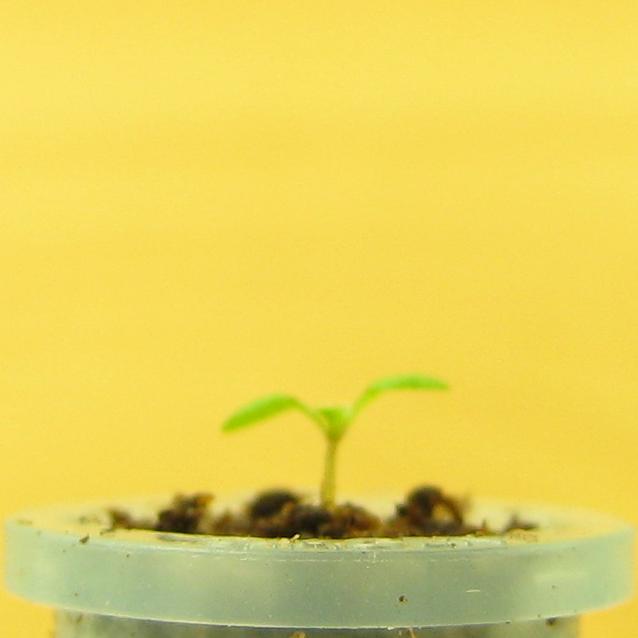

Supplement: Additional file 20 — Col-0 Front View Images for 3-D Model. Images of Col-0 captured every 10 min for 5 days from the front view for the 3-D CG model. Table S2 lists the images used as key frames in the model. [file 13007_2015_75_MOESM20_ESM.zip › front_view/side12_0039.jpg]

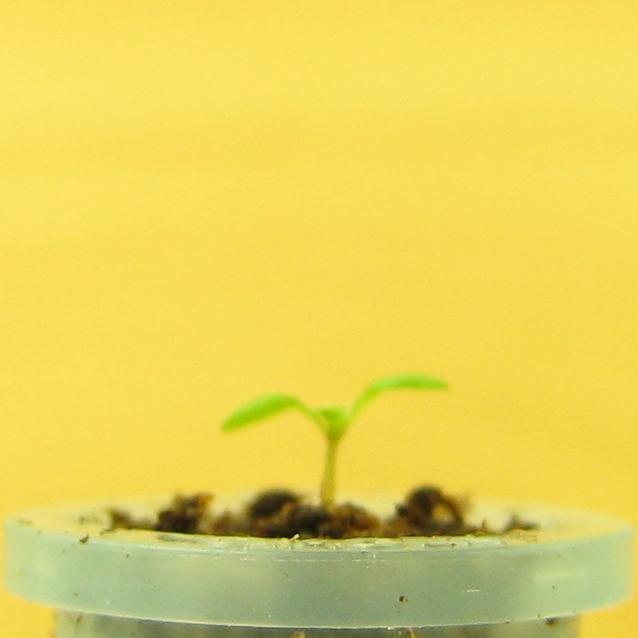

Supplement: Additional file 20 — Col-0 Front View Images for 3-D Model. Images of Col-0 captured every 10 min for 5 days from the front view for the 3-D CG model. Table S2 lists the images used as key frames in the model. [file 13007_2015_75_MOESM20_ESM.zip › front_view/side12_0040.jpg]

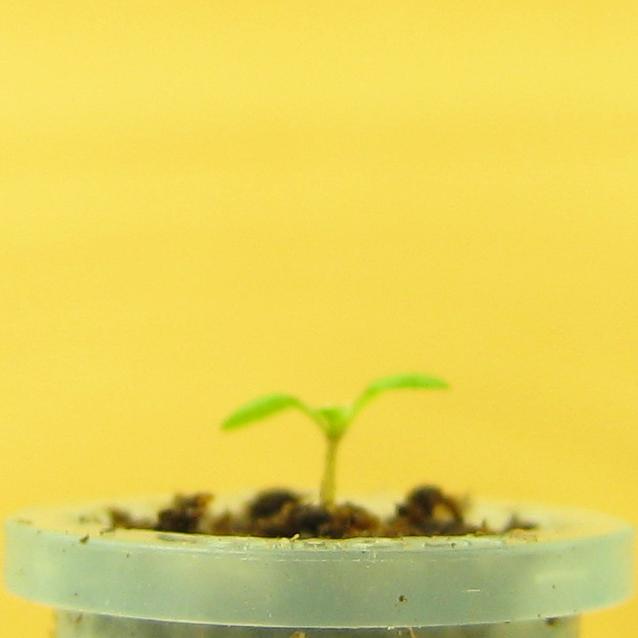

Supplement: Additional file 20 — Col-0 Front View Images for 3-D Model. Images of Col-0 captured every 10 min for 5 days from the front view for the 3-D CG model. Table S2 lists the images used as key frames in the model. [file 13007_2015_75_MOESM20_ESM.zip › front_view/side12_0041.jpg]

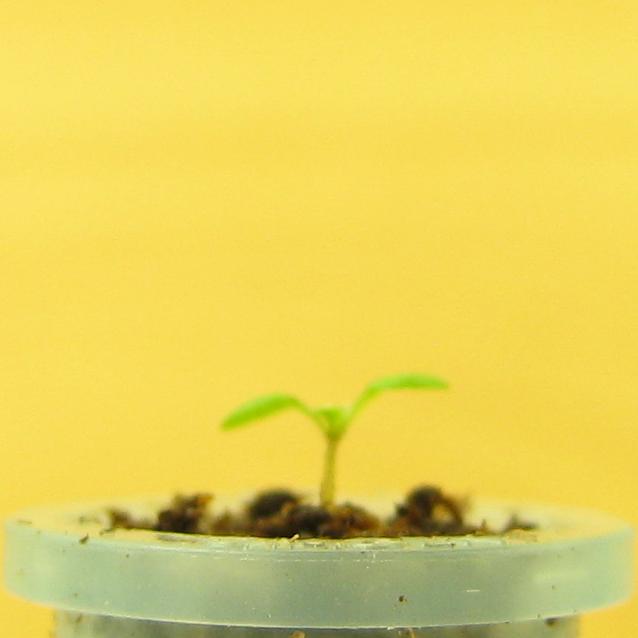

Supplement: Additional file 20 — Col-0 Front View Images for 3-D Model. Images of Col-0 captured every 10 min for 5 days from the front view for the 3-D CG model. Table S2 lists the images used as key frames in the model. [file 13007_2015_75_MOESM20_ESM.zip › front_view/side12_0042.jpg]

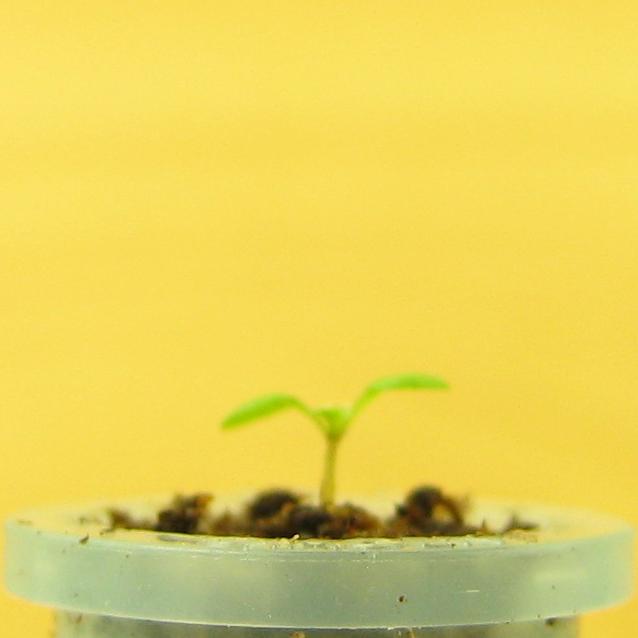

Supplement: Additional file 20 — Col-0 Front View Images for 3-D Model. Images of Col-0 captured every 10 min for 5 days from the front view for the 3-D CG model. Table S2 lists the images used as key frames in the model. [file 13007_2015_75_MOESM20_ESM.zip › front_view/side12_0043.jpg]

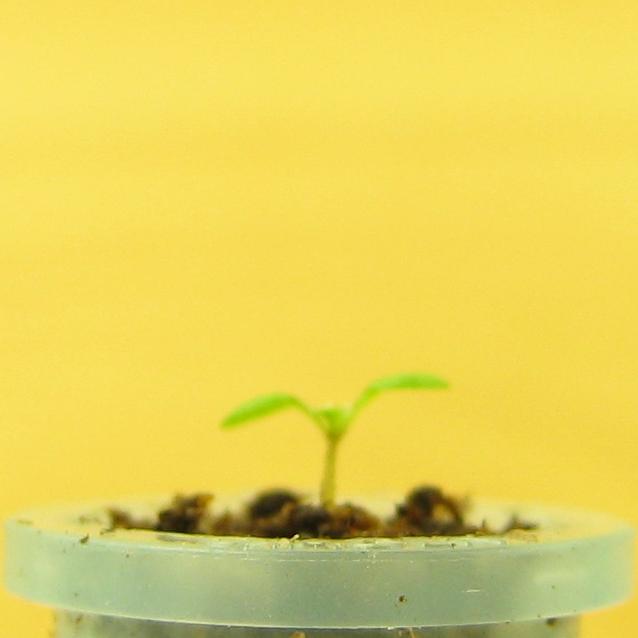

Supplement: Additional file 20 — Col-0 Front View Images for 3-D Model. Images of Col-0 captured every 10 min for 5 days from the front view for the 3-D CG model. Table S2 lists the images used as key frames in the model. [file 13007_2015_75_MOESM20_ESM.zip › front_view/side12_0044.jpg]

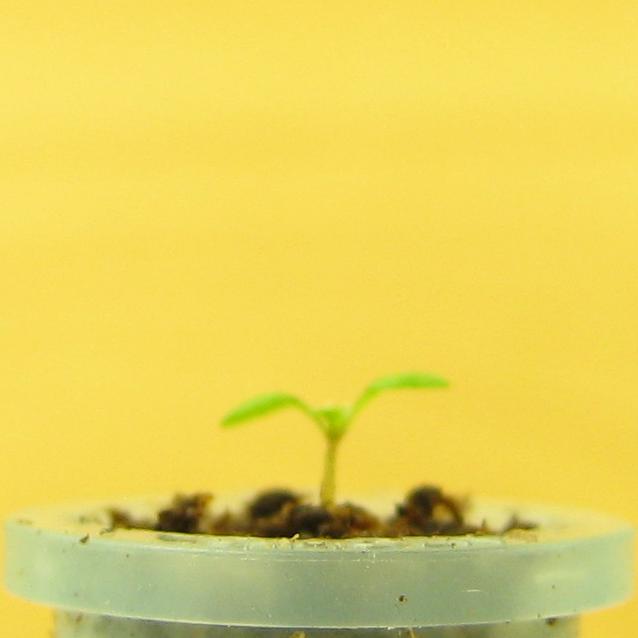

Supplement: Additional file 20 — Col-0 Front View Images for 3-D Model. Images of Col-0 captured every 10 min for 5 days from the front view for the 3-D CG model. Table S2 lists the images used as key frames in the model. [file 13007_2015_75_MOESM20_ESM.zip › front_view/side12_0045.jpg]

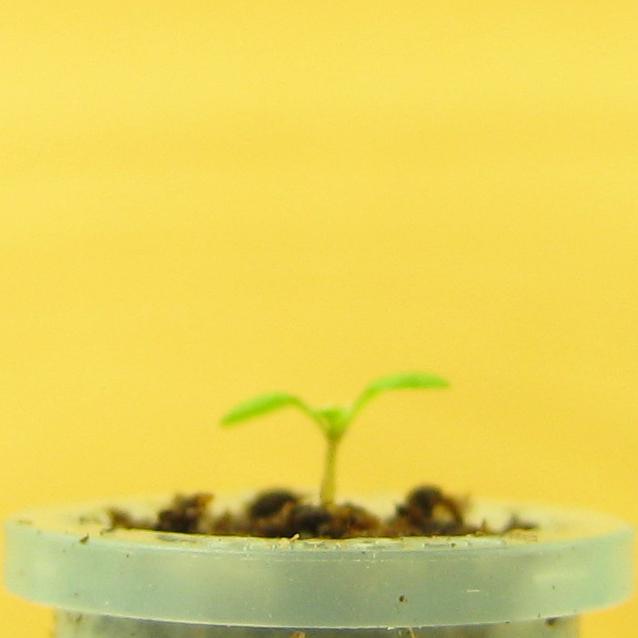

Supplement: Additional file 20 — Col-0 Front View Images for 3-D Model. Images of Col-0 captured every 10 min for 5 days from the front view for the 3-D CG model. Table S2 lists the images used as key frames in the model. [file 13007_2015_75_MOESM20_ESM.zip › front_view/side12_0046.jpg]

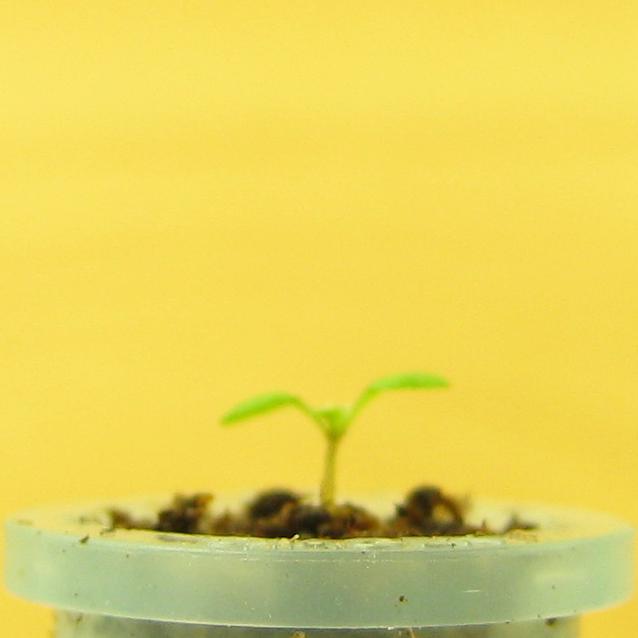

Supplement: Additional file 20 — Col-0 Front View Images for 3-D Model. Images of Col-0 captured every 10 min for 5 days from the front view for the 3-D CG model. Table S2 lists the images used as key frames in the model. [file 13007_2015_75_MOESM20_ESM.zip › front_view/side12_0047.jpg]

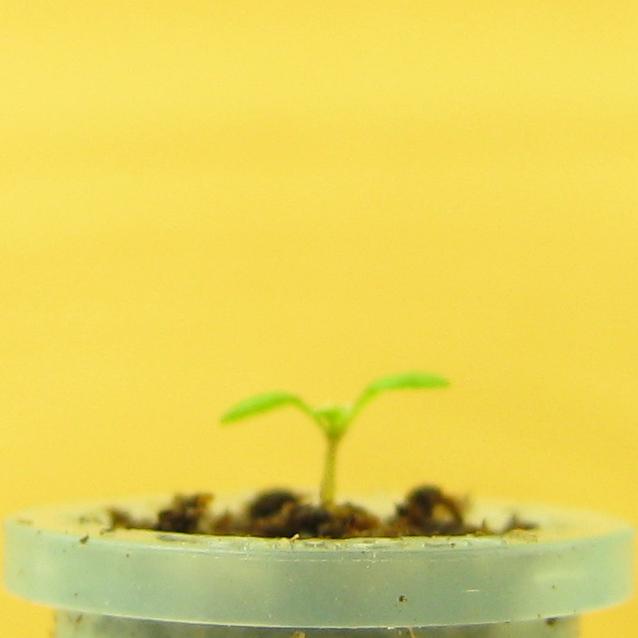

Supplement: Additional file 20 — Col-0 Front View Images for 3-D Model. Images of Col-0 captured every 10 min for 5 days from the front view for the 3-D CG model. Table S2 lists the images used as key frames in the model. [file 13007_2015_75_MOESM20_ESM.zip › front_view/side12_0048.jpg]

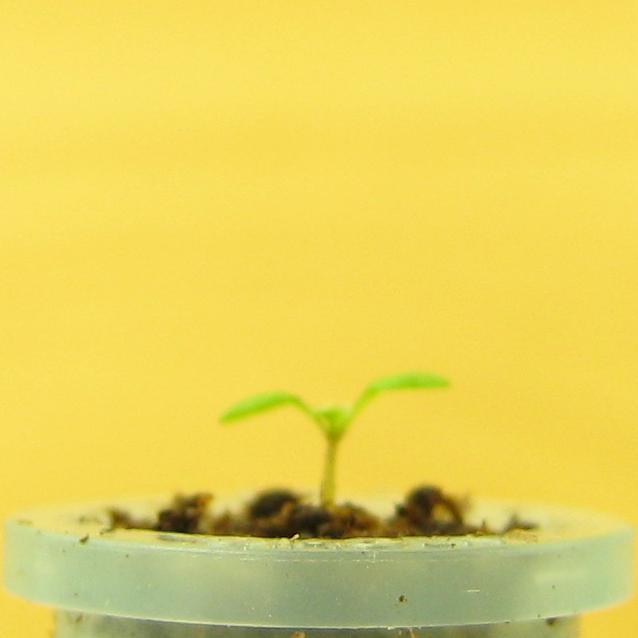

Supplement: Additional file 20 — Col-0 Front View Images for 3-D Model. Images of Col-0 captured every 10 min for 5 days from the front view for the 3-D CG model. Table S2 lists the images used as key frames in the model. [file 13007_2015_75_MOESM20_ESM.zip › front_view/side12_0049.jpg]

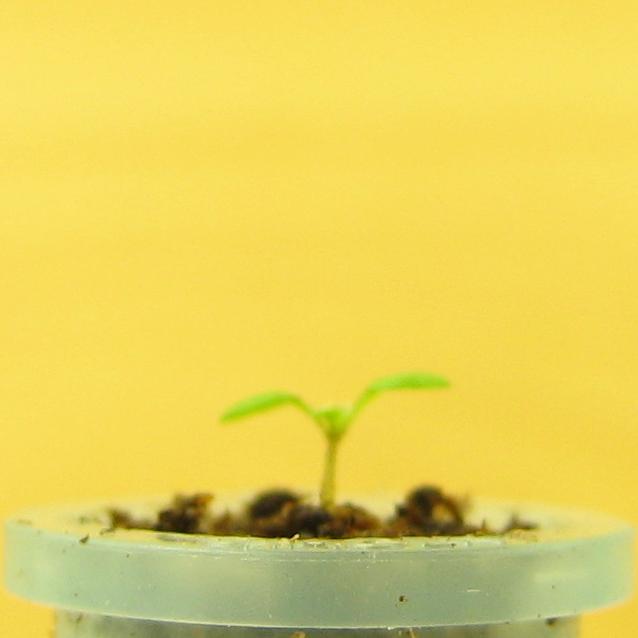

Supplement: Additional file 20 — Col-0 Front View Images for 3-D Model. Images of Col-0 captured every 10 min for 5 days from the front view for the 3-D CG model. Table S2 lists the images used as key frames in the model. [file 13007_2015_75_MOESM20_ESM.zip › front_view/side12_0050.jpg]

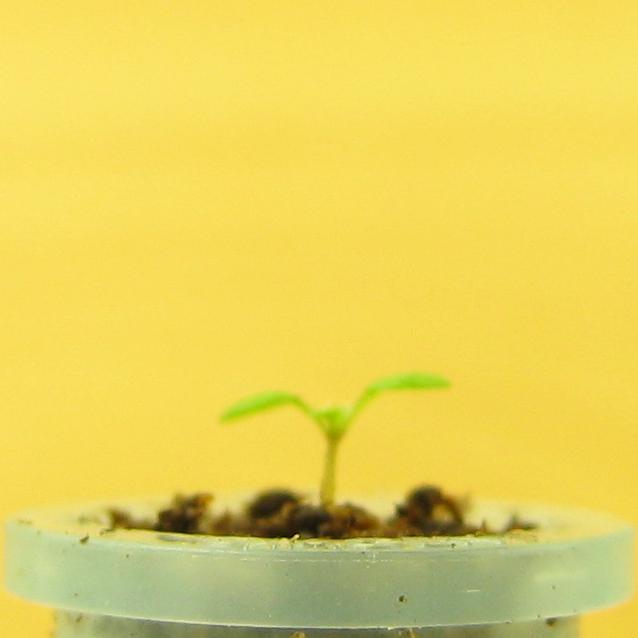

Supplement: Additional file 20 — Col-0 Front View Images for 3-D Model. Images of Col-0 captured every 10 min for 5 days from the front view for the 3-D CG model. Table S2 lists the images used as key frames in the model. [file 13007_2015_75_MOESM20_ESM.zip › front_view/side12_0051.jpg]

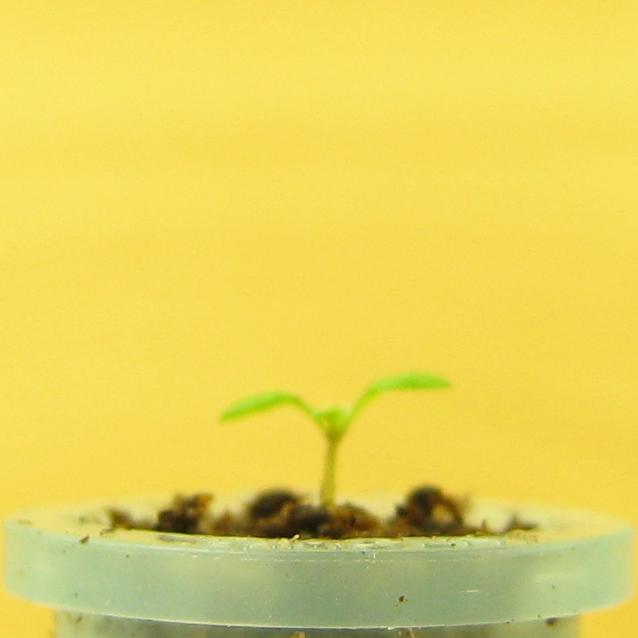

Supplement: Additional file 20 — Col-0 Front View Images for 3-D Model. Images of Col-0 captured every 10 min for 5 days from the front view for the 3-D CG model. Table S2 lists the images used as key frames in the model. [file 13007_2015_75_MOESM20_ESM.zip › front_view/side12_0052.jpg]

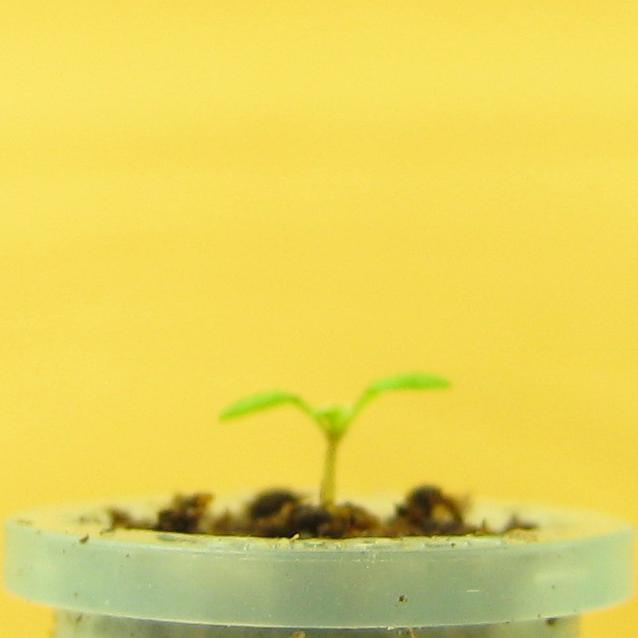

Supplement: Additional file 20 — Col-0 Front View Images for 3-D Model. Images of Col-0 captured every 10 min for 5 days from the front view for the 3-D CG model. Table S2 lists the images used as key frames in the model. [file 13007_2015_75_MOESM20_ESM.zip › front_view/side12_0053.jpg]

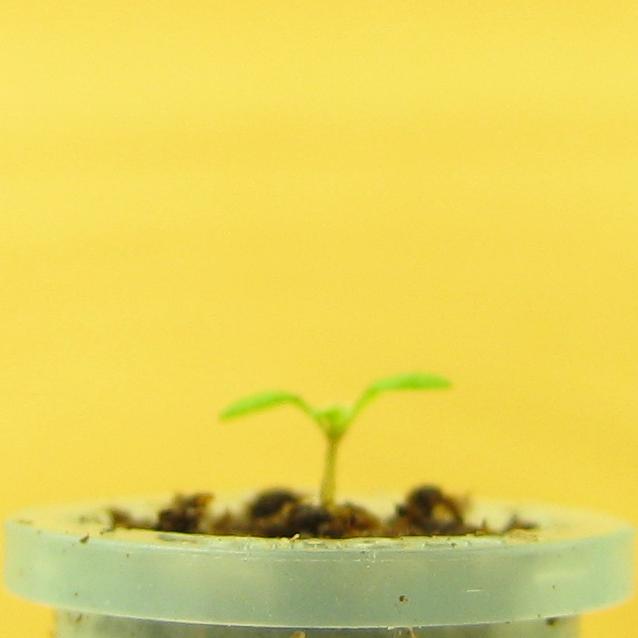

Supplement: Additional file 20 — Col-0 Front View Images for 3-D Model. Images of Col-0 captured every 10 min for 5 days from the front view for the 3-D CG model. Table S2 lists the images used as key frames in the model. [file 13007_2015_75_MOESM20_ESM.zip › front_view/side12_0054.jpg]

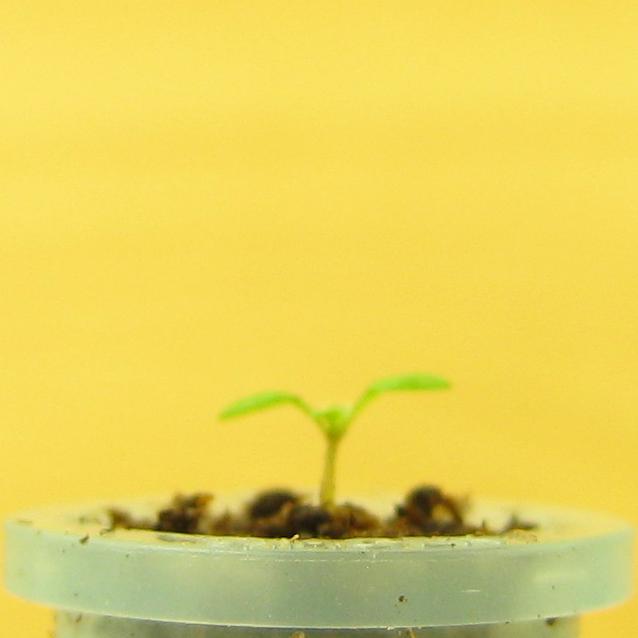

Supplement: Additional file 20 — Col-0 Front View Images for 3-D Model. Images of Col-0 captured every 10 min for 5 days from the front view for the 3-D CG model. Table S2 lists the images used as key frames in the model. [file 13007_2015_75_MOESM20_ESM.zip › front_view/side12_0055.jpg]

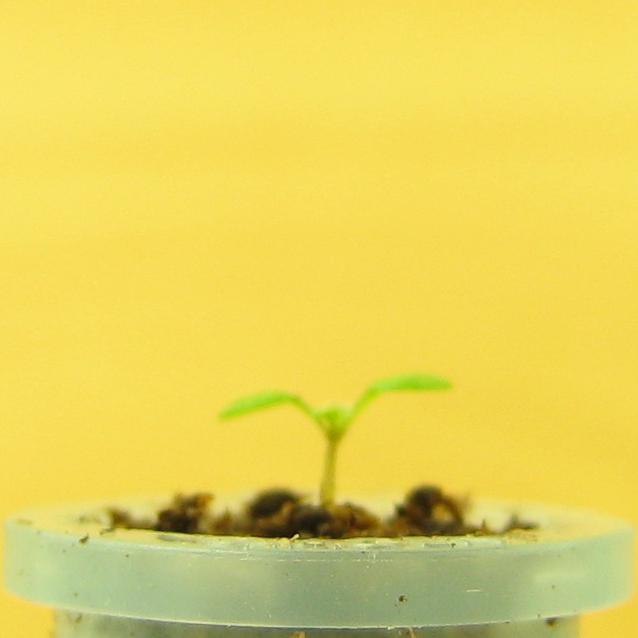

Supplement: Additional file 20 — Col-0 Front View Images for 3-D Model. Images of Col-0 captured every 10 min for 5 days from the front view for the 3-D CG model. Table S2 lists the images used as key frames in the model. [file 13007_2015_75_MOESM20_ESM.zip › front_view/side12_0056.jpg]

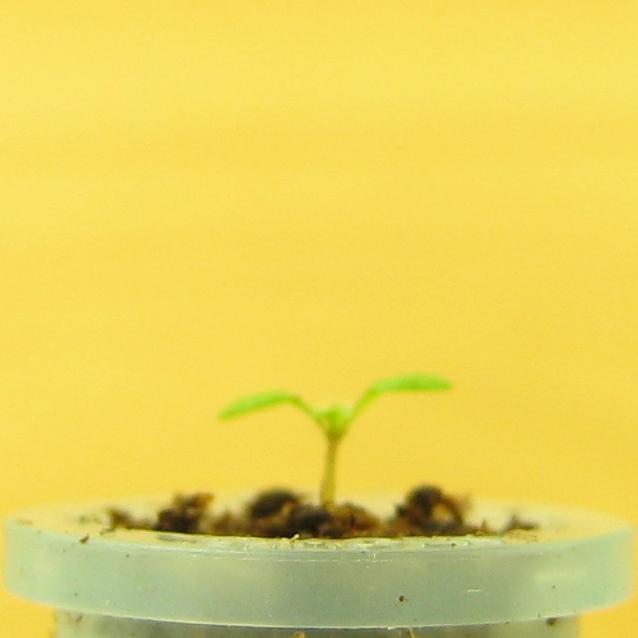

Supplement: Additional file 20 — Col-0 Front View Images for 3-D Model. Images of Col-0 captured every 10 min for 5 days from the front view for the 3-D CG model. Table S2 lists the images used as key frames in the model. [file 13007_2015_75_MOESM20_ESM.zip › front_view/side12_0057.jpg]

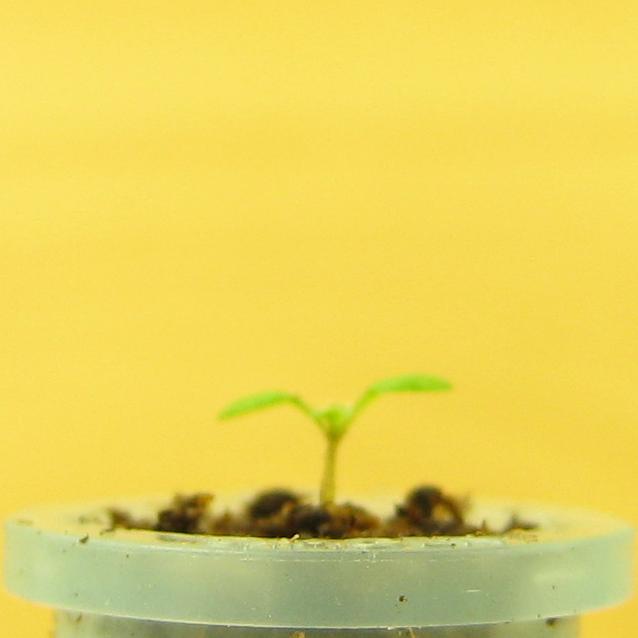

Supplement: Additional file 20 — Col-0 Front View Images for 3-D Model. Images of Col-0 captured every 10 min for 5 days from the front view for the 3-D CG model. Table S2 lists the images used as key frames in the model. [file 13007_2015_75_MOESM20_ESM.zip › front_view/side12_0058.jpg]

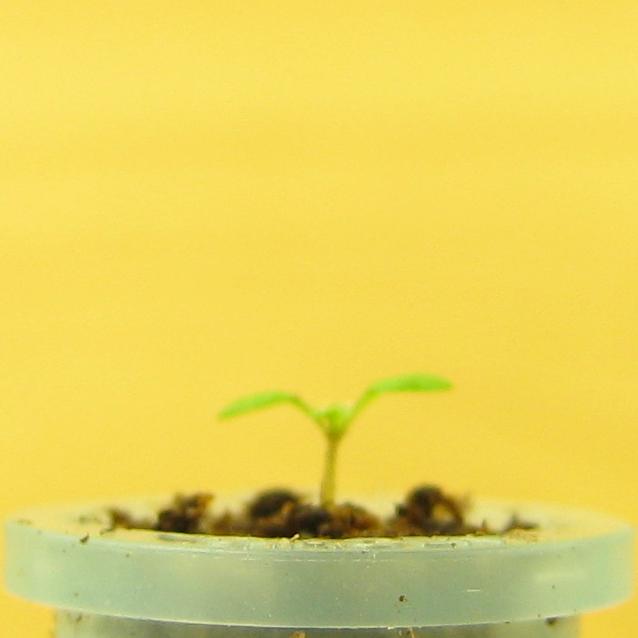

Supplement: Additional file 20 — Col-0 Front View Images for 3-D Model. Images of Col-0 captured every 10 min for 5 days from the front view for the 3-D CG model. Table S2 lists the images used as key frames in the model. [file 13007_2015_75_MOESM20_ESM.zip › front_view/side12_0059.jpg]

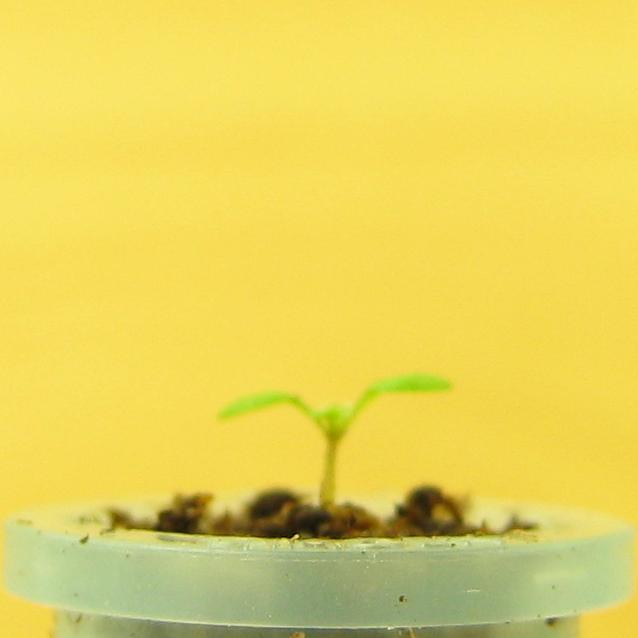

Supplement: Additional file 20 — Col-0 Front View Images for 3-D Model. Images of Col-0 captured every 10 min for 5 days from the front view for the 3-D CG model. Table S2 lists the images used as key frames in the model. [file 13007_2015_75_MOESM20_ESM.zip › front_view/side12_0060.jpg]

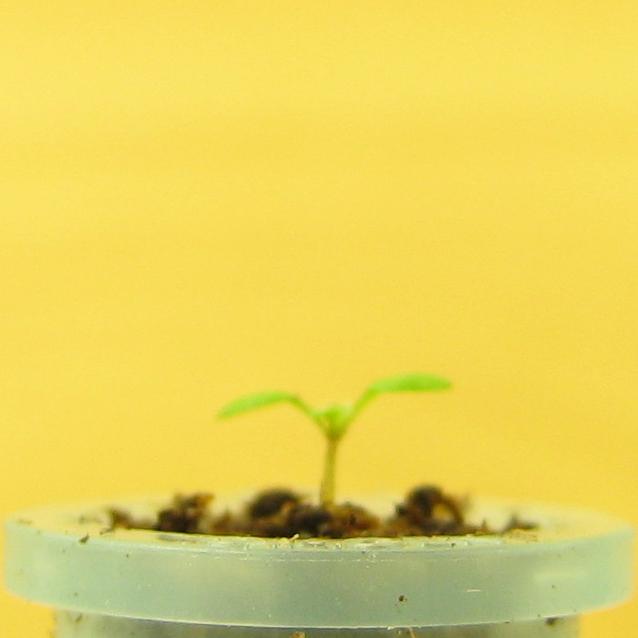

Supplement: Additional file 20 — Col-0 Front View Images for 3-D Model. Images of Col-0 captured every 10 min for 5 days from the front view for the 3-D CG model. Table S2 lists the images used as key frames in the model. [file 13007_2015_75_MOESM20_ESM.zip › front_view/side12_0061.jpg]

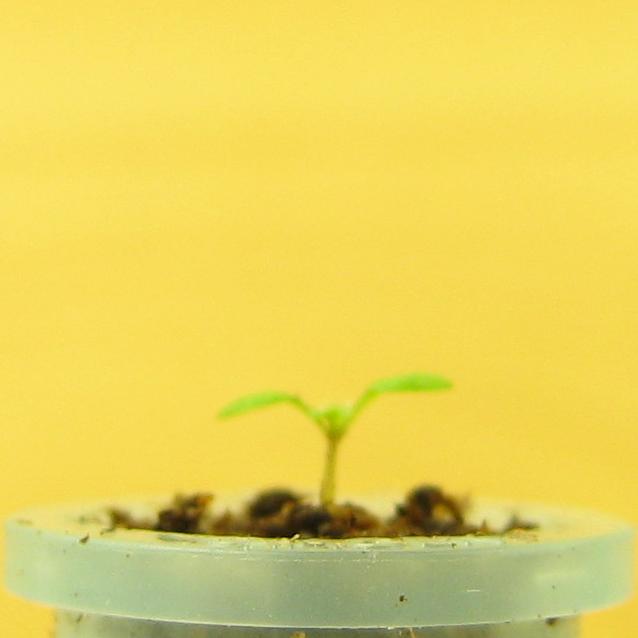

Supplement: Additional file 20 — Col-0 Front View Images for 3-D Model. Images of Col-0 captured every 10 min for 5 days from the front view for the 3-D CG model. Table S2 lists the images used as key frames in the model. [file 13007_2015_75_MOESM20_ESM.zip › front_view/side12_0062.jpg]

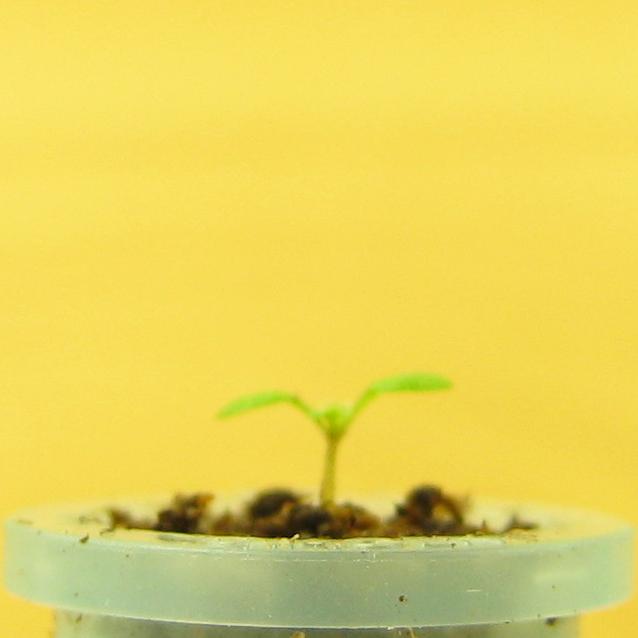

Supplement: Additional file 20 — Col-0 Front View Images for 3-D Model. Images of Col-0 captured every 10 min for 5 days from the front view for the 3-D CG model. Table S2 lists the images used as key frames in the model. [file 13007_2015_75_MOESM20_ESM.zip › front_view/side12_0063.jpg]

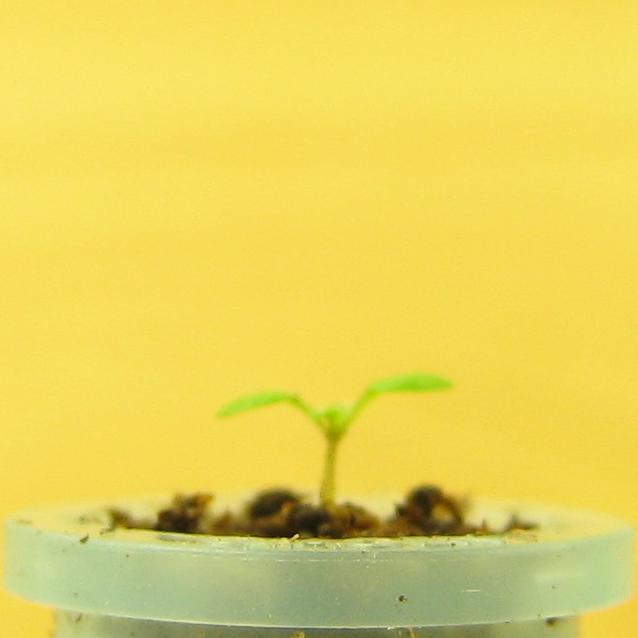

Supplement: Additional file 20 — Col-0 Front View Images for 3-D Model. Images of Col-0 captured every 10 min for 5 days from the front view for the 3-D CG model. Table S2 lists the images used as key frames in the model. [file 13007_2015_75_MOESM20_ESM.zip › front_view/side12_0064.jpg]

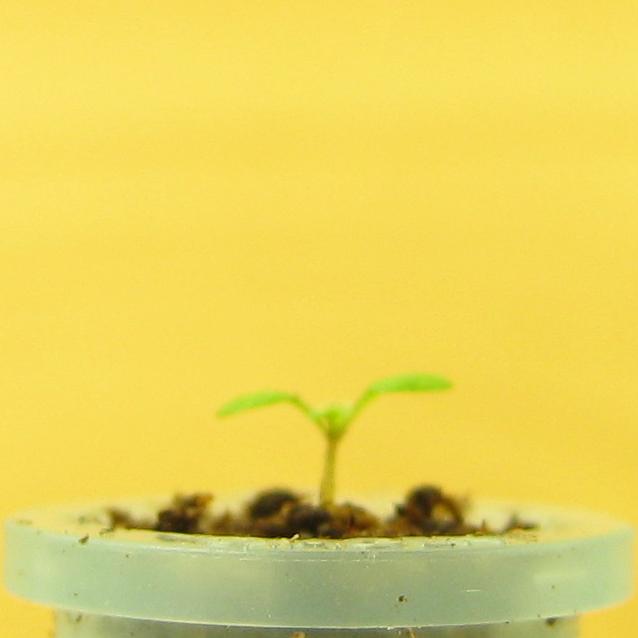

Supplement: Additional file 20 — Col-0 Front View Images for 3-D Model. Images of Col-0 captured every 10 min for 5 days from the front view for the 3-D CG model. Table S2 lists the images used as key frames in the model. [file 13007_2015_75_MOESM20_ESM.zip › front_view/side12_0065.jpg]

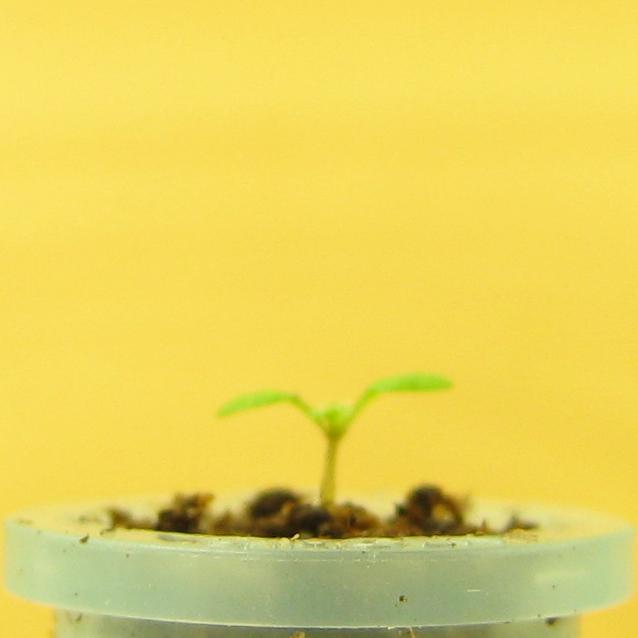

Supplement: Additional file 20 — Col-0 Front View Images for 3-D Model. Images of Col-0 captured every 10 min for 5 days from the front view for the 3-D CG model. Table S2 lists the images used as key frames in the model. [file 13007_2015_75_MOESM20_ESM.zip › front_view/side12_0066.jpg]

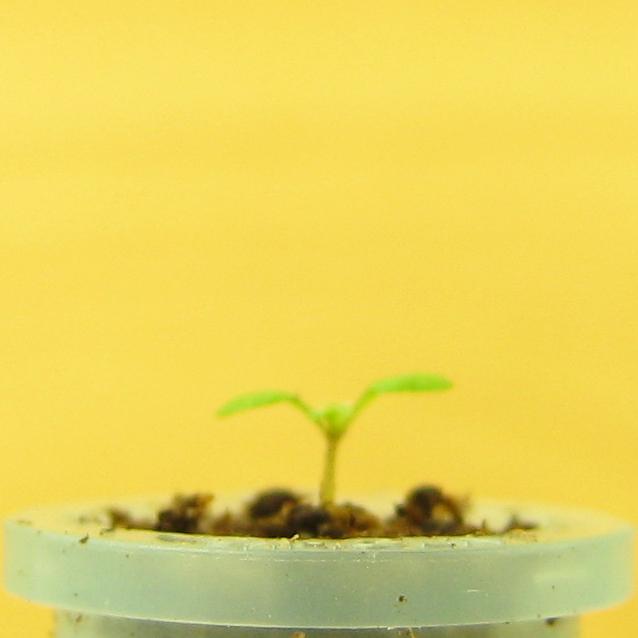

Supplement: Additional file 20 — Col-0 Front View Images for 3-D Model. Images of Col-0 captured every 10 min for 5 days from the front view for the 3-D CG model. Table S2 lists the images used as key frames in the model. [file 13007_2015_75_MOESM20_ESM.zip › front_view/side12_0067.jpg]

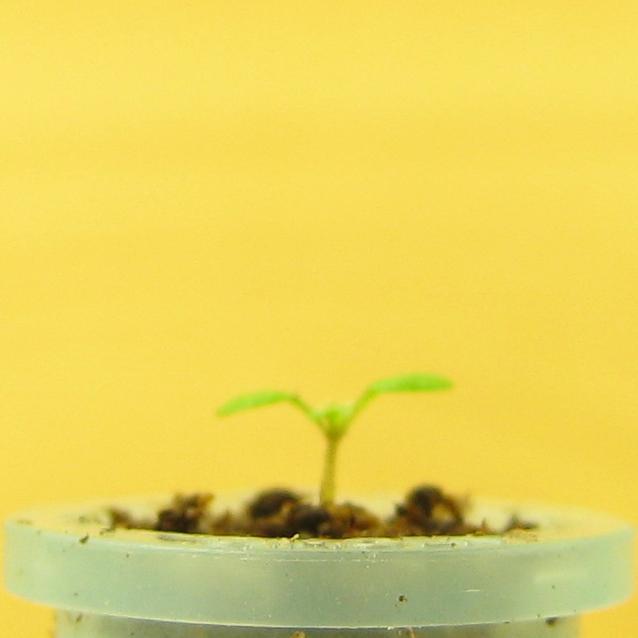

Supplement: Additional file 20 — Col-0 Front View Images for 3-D Model. Images of Col-0 captured every 10 min for 5 days from the front view for the 3-D CG model. Table S2 lists the images used as key frames in the model. [file 13007_2015_75_MOESM20_ESM.zip › front_view/side12_0068.jpg]

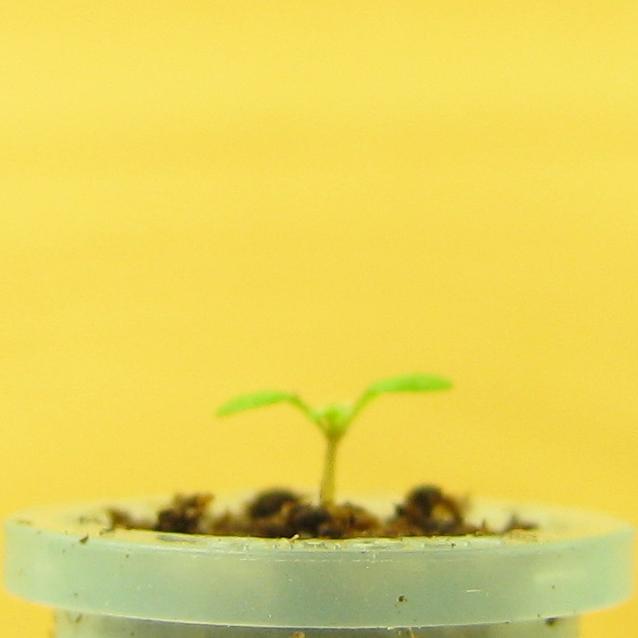

Supplement: Additional file 20 — Col-0 Front View Images for 3-D Model. Images of Col-0 captured every 10 min for 5 days from the front view for the 3-D CG model. Table S2 lists the images used as key frames in the model. [file 13007_2015_75_MOESM20_ESM.zip › front_view/side12_0069.jpg]

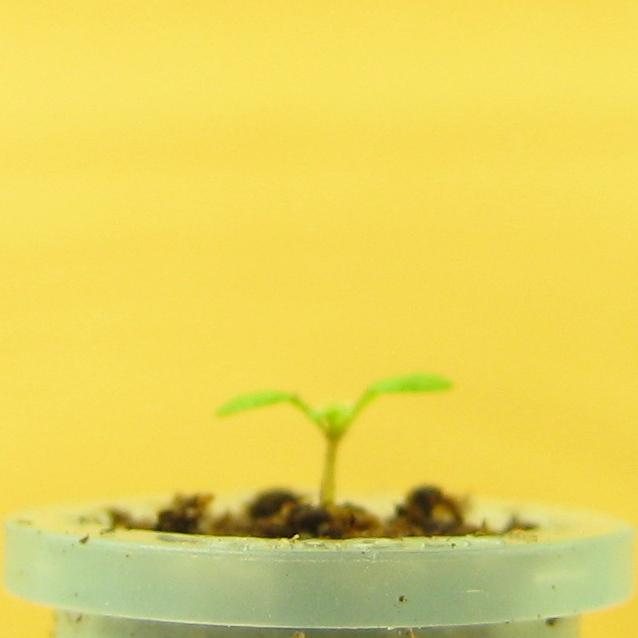

Supplement: Additional file 20 — Col-0 Front View Images for 3-D Model. Images of Col-0 captured every 10 min for 5 days from the front view for the 3-D CG model. Table S2 lists the images used as key frames in the model. [file 13007_2015_75_MOESM20_ESM.zip › front_view/side12_0070.jpg]

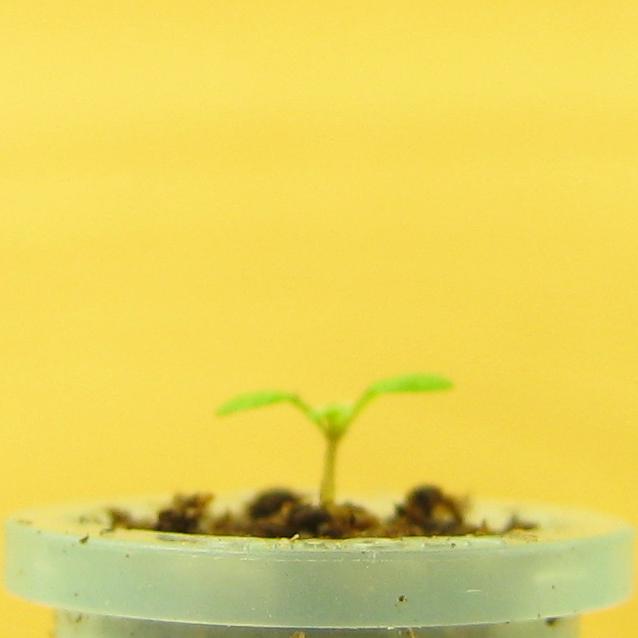

Supplement: Additional file 20 — Col-0 Front View Images for 3-D Model. Images of Col-0 captured every 10 min for 5 days from the front view for the 3-D CG model. Table S2 lists the images used as key frames in the model. [file 13007_2015_75_MOESM20_ESM.zip › front_view/side12_0071.jpg]

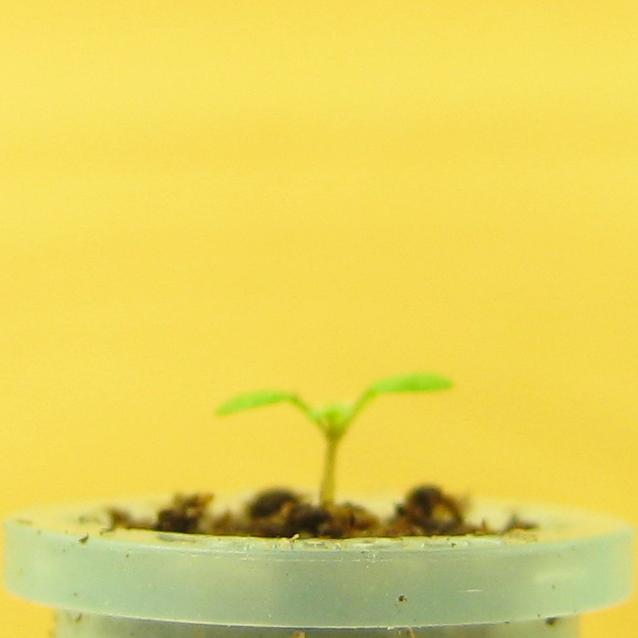

Supplement: Additional file 20 — Col-0 Front View Images for 3-D Model. Images of Col-0 captured every 10 min for 5 days from the front view for the 3-D CG model. Table S2 lists the images used as key frames in the model. [file 13007_2015_75_MOESM20_ESM.zip › front_view/side12_0072.jpg]

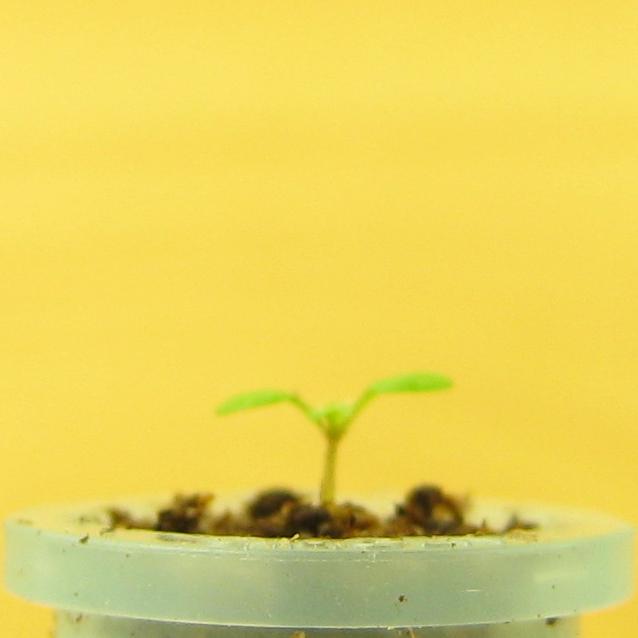

Supplement: Additional file 20 — Col-0 Front View Images for 3-D Model. Images of Col-0 captured every 10 min for 5 days from the front view for the 3-D CG model. Table S2 lists the images used as key frames in the model. [file 13007_2015_75_MOESM20_ESM.zip › front_view/side12_0073.jpg]

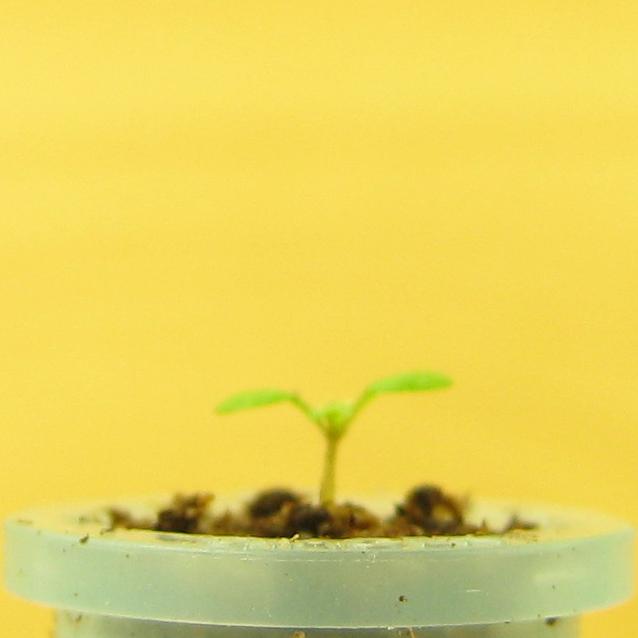

Supplement: Additional file 20 — Col-0 Front View Images for 3-D Model. Images of Col-0 captured every 10 min for 5 days from the front view for the 3-D CG model. Table S2 lists the images used as key frames in the model. [file 13007_2015_75_MOESM20_ESM.zip › front_view/side12_0074.jpg]

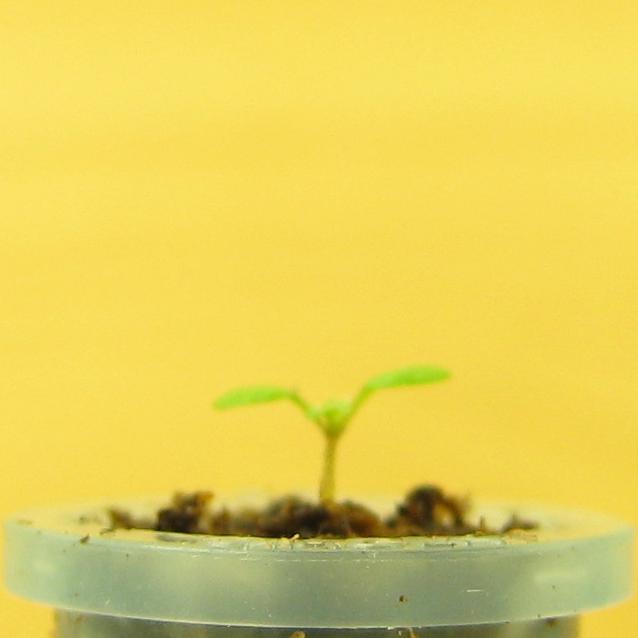

Supplement: Additional file 20 — Col-0 Front View Images for 3-D Model. Images of Col-0 captured every 10 min for 5 days from the front view for the 3-D CG model. Table S2 lists the images used as key frames in the model. [file 13007_2015_75_MOESM20_ESM.zip › front_view/side12_0075.jpg]

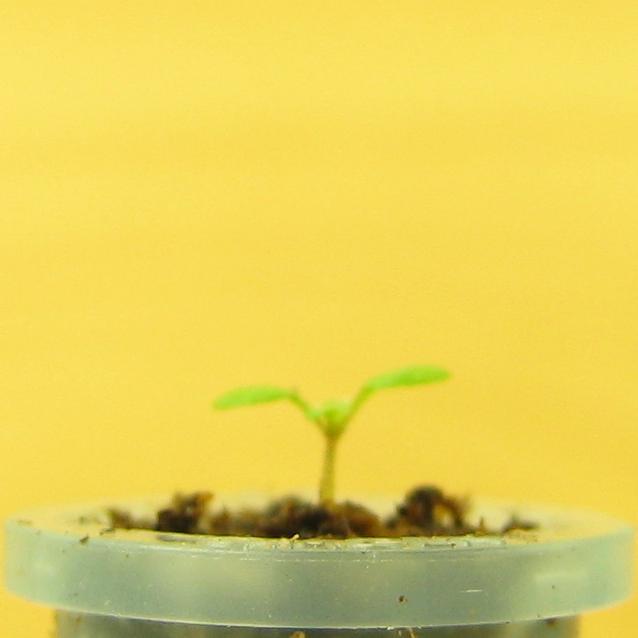

Supplement: Additional file 20 — Col-0 Front View Images for 3-D Model. Images of Col-0 captured every 10 min for 5 days from the front view for the 3-D CG model. Table S2 lists the images used as key frames in the model. [file 13007_2015_75_MOESM20_ESM.zip › front_view/side12_0076.jpg]

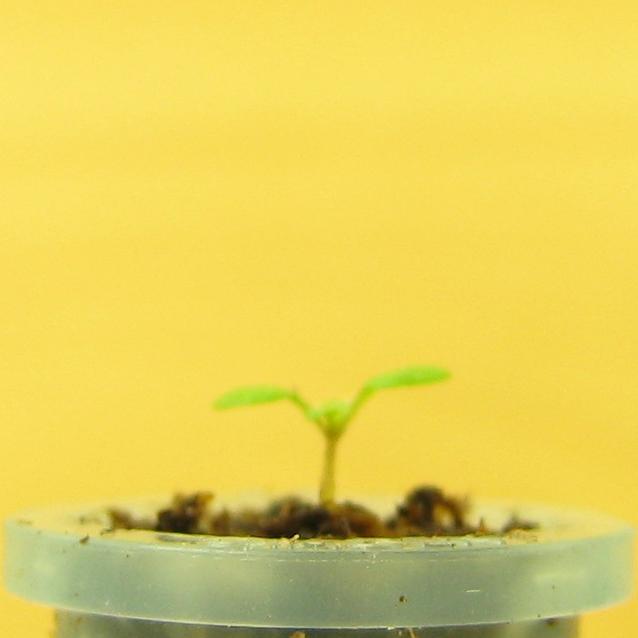

Supplement: Additional file 20 — Col-0 Front View Images for 3-D Model. Images of Col-0 captured every 10 min for 5 days from the front view for the 3-D CG model. Table S2 lists the images used as key frames in the model. [file 13007_2015_75_MOESM20_ESM.zip › front_view/side12_0077.jpg]

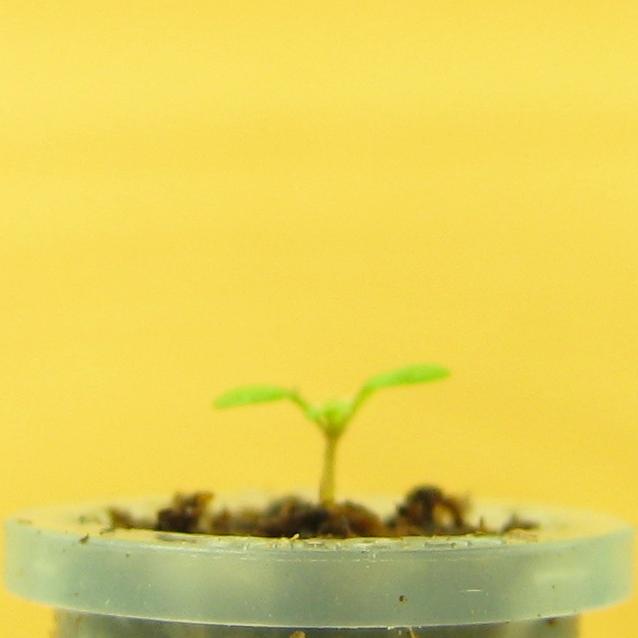

Supplement: Additional file 20 — Col-0 Front View Images for 3-D Model. Images of Col-0 captured every 10 min for 5 days from the front view for the 3-D CG model. Table S2 lists the images used as key frames in the model. [file 13007_2015_75_MOESM20_ESM.zip › front_view/side12_0078.jpg]

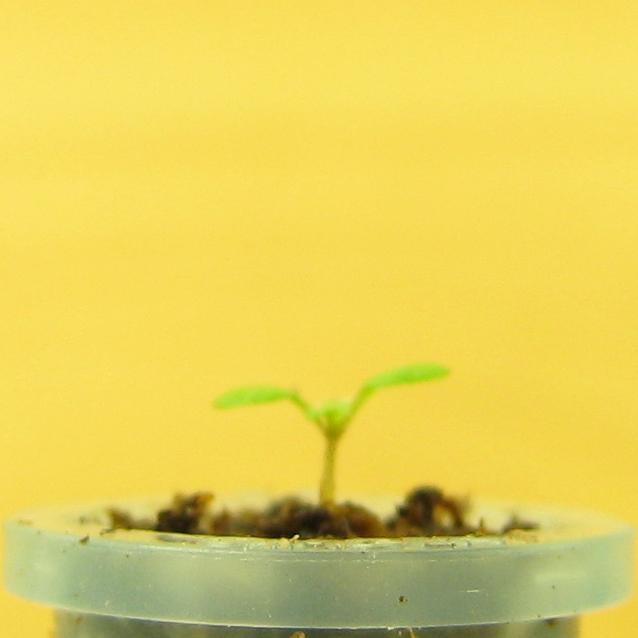

Supplement: Additional file 20 — Col-0 Front View Images for 3-D Model. Images of Col-0 captured every 10 min for 5 days from the front view for the 3-D CG model. Table S2 lists the images used as key frames in the model. [file 13007_2015_75_MOESM20_ESM.zip › front_view/side12_0079.jpg]

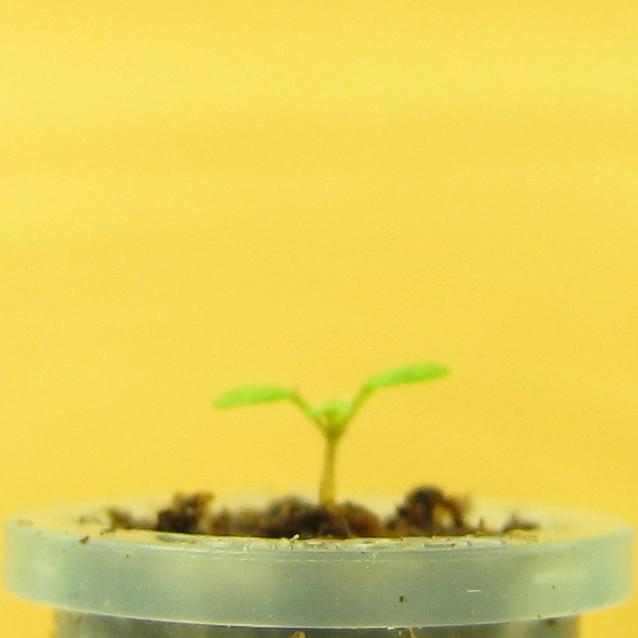

Supplement: Additional file 20 — Col-0 Front View Images for 3-D Model. Images of Col-0 captured every 10 min for 5 days from the front view for the 3-D CG model. Table S2 lists the images used as key frames in the model. [file 13007_2015_75_MOESM20_ESM.zip › front_view/side12_0080.jpg]

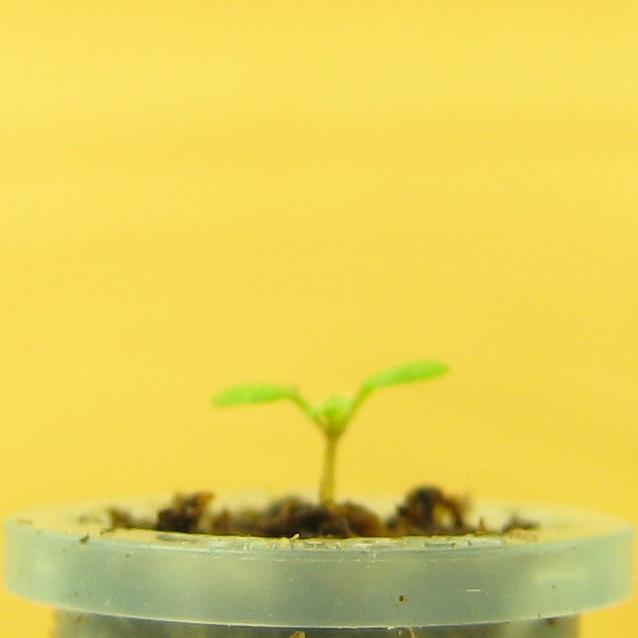

Supplement: Additional file 20 — Col-0 Front View Images for 3-D Model. Images of Col-0 captured every 10 min for 5 days from the front view for the 3-D CG model. Table S2 lists the images used as key frames in the model. [file 13007_2015_75_MOESM20_ESM.zip › front_view/side12_0081.jpg]

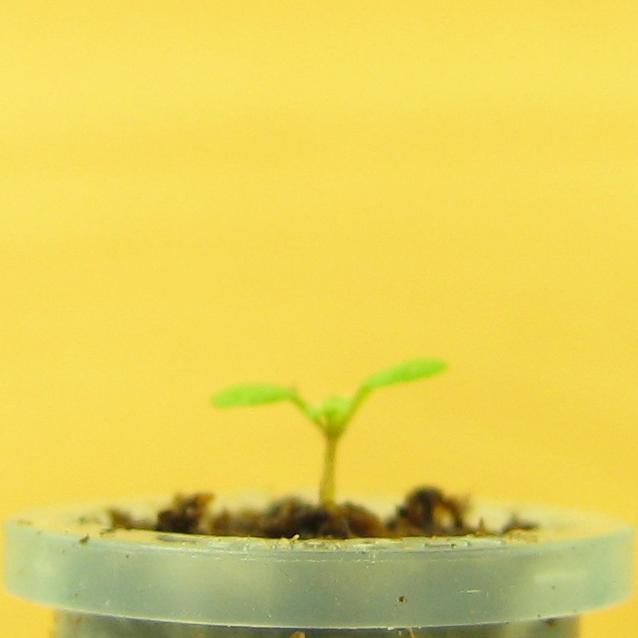

Supplement: Additional file 20 — Col-0 Front View Images for 3-D Model. Images of Col-0 captured every 10 min for 5 days from the front view for the 3-D CG model. Table S2 lists the images used as key frames in the model. [file 13007_2015_75_MOESM20_ESM.zip › front_view/side12_0082.jpg]

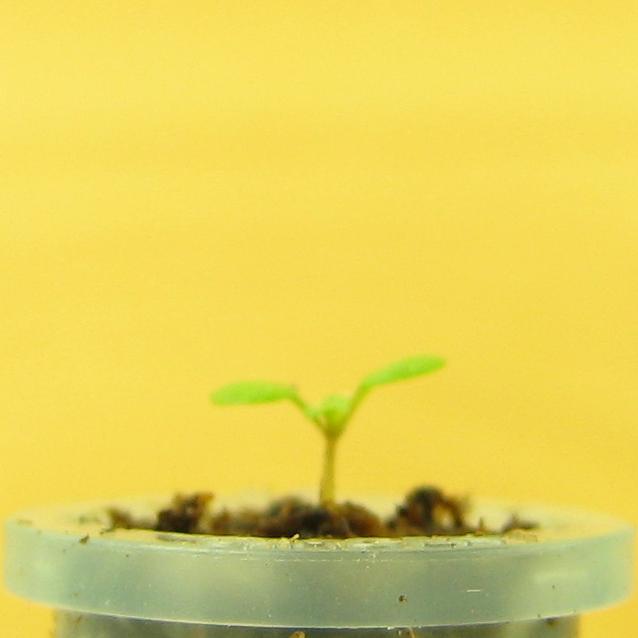

Supplement: Additional file 20 — Col-0 Front View Images for 3-D Model. Images of Col-0 captured every 10 min for 5 days from the front view for the 3-D CG model. Table S2 lists the images used as key frames in the model. [file 13007_2015_75_MOESM20_ESM.zip › front_view/side12_0083.jpg]

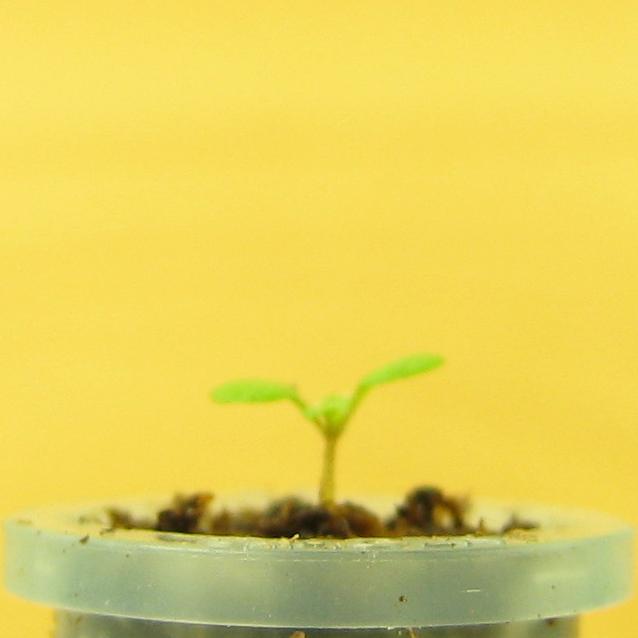

Supplement: Additional file 20 — Col-0 Front View Images for 3-D Model. Images of Col-0 captured every 10 min for 5 days from the front view for the 3-D CG model. Table S2 lists the images used as key frames in the model. [file 13007_2015_75_MOESM20_ESM.zip › front_view/side12_0084.jpg]

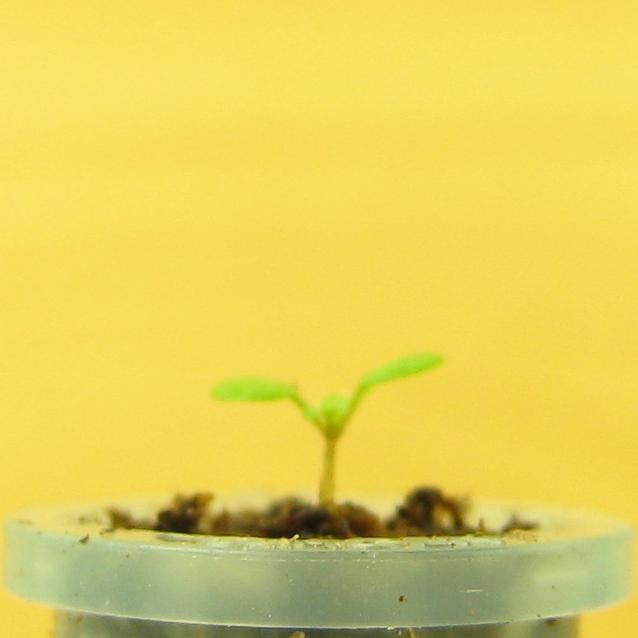

Supplement: Additional file 20 — Col-0 Front View Images for 3-D Model. Images of Col-0 captured every 10 min for 5 days from the front view for the 3-D CG model. Table S2 lists the images used as key frames in the model. [file 13007_2015_75_MOESM20_ESM.zip › front_view/side12_0085.jpg]

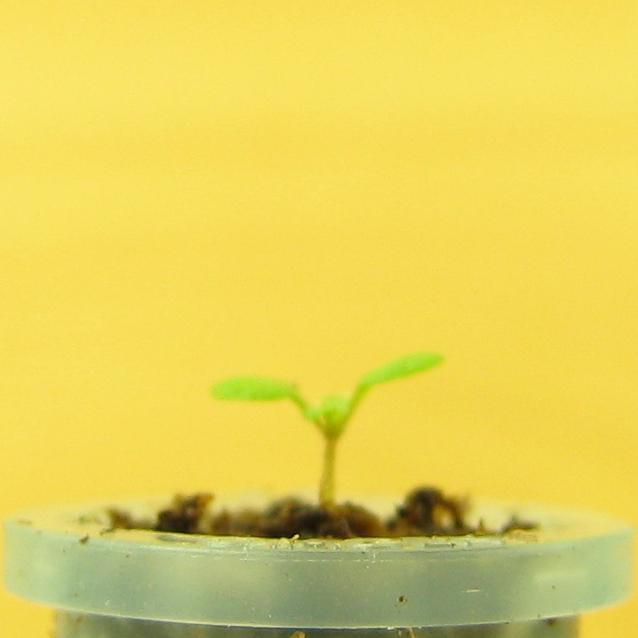

Supplement: Additional file 20 — Col-0 Front View Images for 3-D Model. Images of Col-0 captured every 10 min for 5 days from the front view for the 3-D CG model. Table S2 lists the images used as key frames in the model. [file 13007_2015_75_MOESM20_ESM.zip › front_view/side12_0086.jpg]

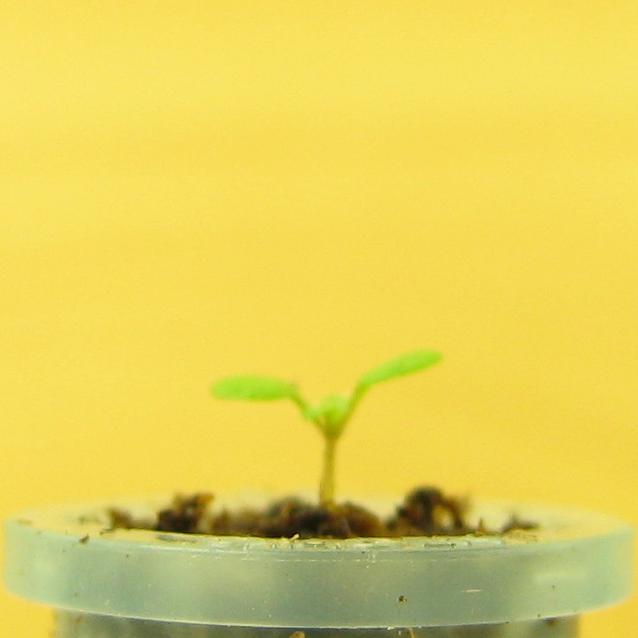

Supplement: Additional file 20 — Col-0 Front View Images for 3-D Model. Images of Col-0 captured every 10 min for 5 days from the front view for the 3-D CG model. Table S2 lists the images used as key frames in the model. [file 13007_2015_75_MOESM20_ESM.zip › front_view/side12_0087.jpg]

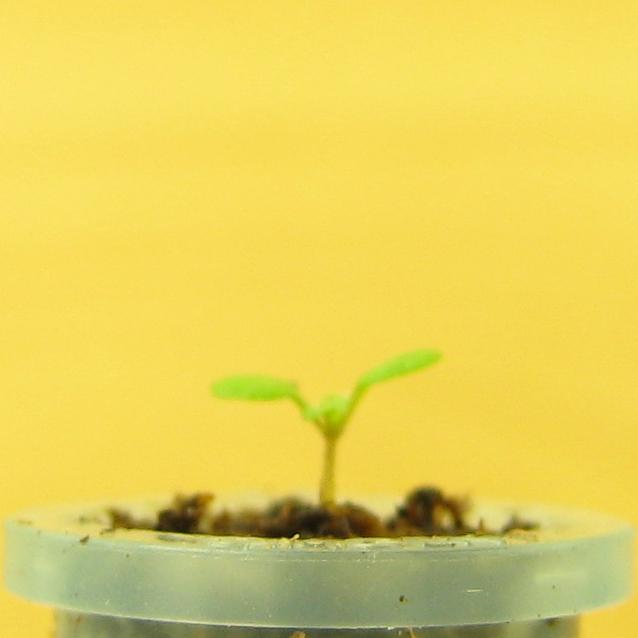

Supplement: Additional file 20 — Col-0 Front View Images for 3-D Model. Images of Col-0 captured every 10 min for 5 days from the front view for the 3-D CG model. Table S2 lists the images used as key frames in the model. [file 13007_2015_75_MOESM20_ESM.zip › front_view/side12_0088.jpg]

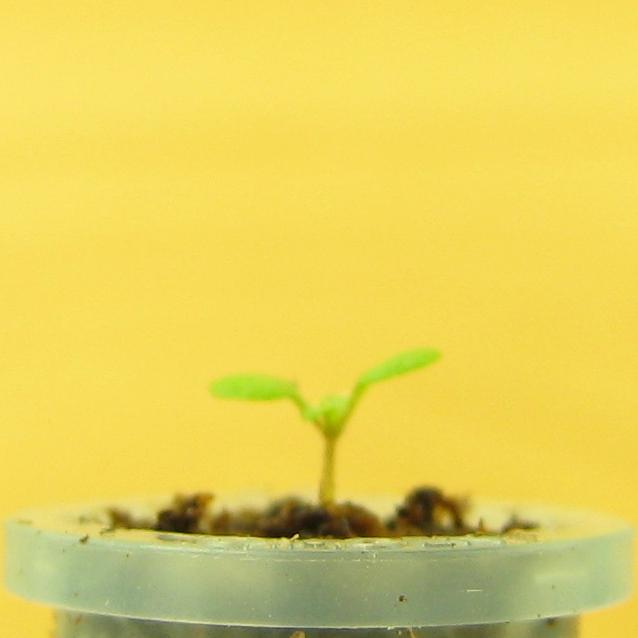

Supplement: Additional file 20 — Col-0 Front View Images for 3-D Model. Images of Col-0 captured every 10 min for 5 days from the front view for the 3-D CG model. Table S2 lists the images used as key frames in the model. [file 13007_2015_75_MOESM20_ESM.zip › front_view/side12_0089.jpg]

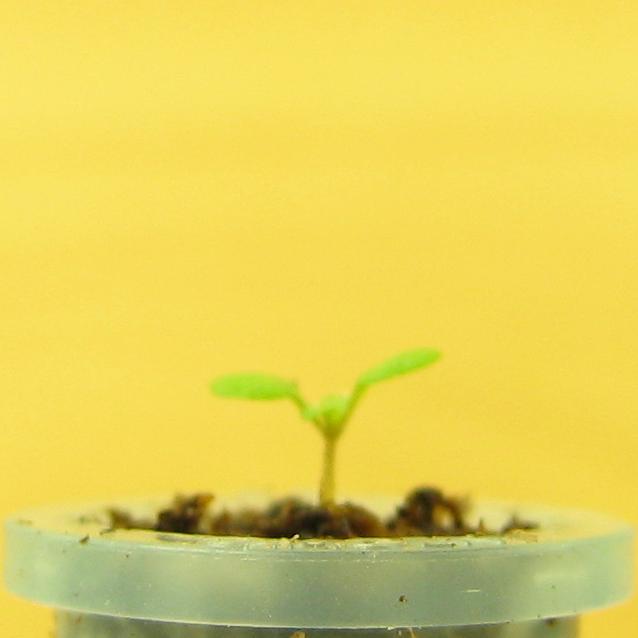

Supplement: Additional file 20 — Col-0 Front View Images for 3-D Model. Images of Col-0 captured every 10 min for 5 days from the front view for the 3-D CG model. Table S2 lists the images used as key frames in the model. [file 13007_2015_75_MOESM20_ESM.zip › front_view/side12_0090.jpg]

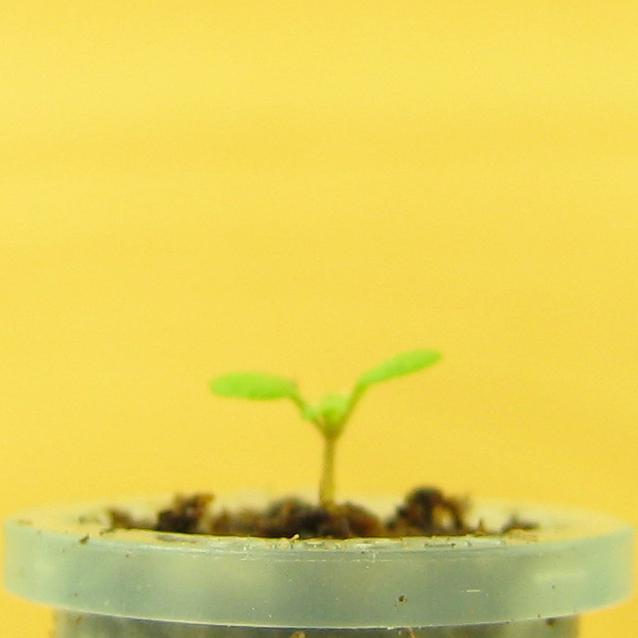

Supplement: Additional file 20 — Col-0 Front View Images for 3-D Model. Images of Col-0 captured every 10 min for 5 days from the front view for the 3-D CG model. Table S2 lists the images used as key frames in the model. [file 13007_2015_75_MOESM20_ESM.zip › front_view/side12_0091.jpg]

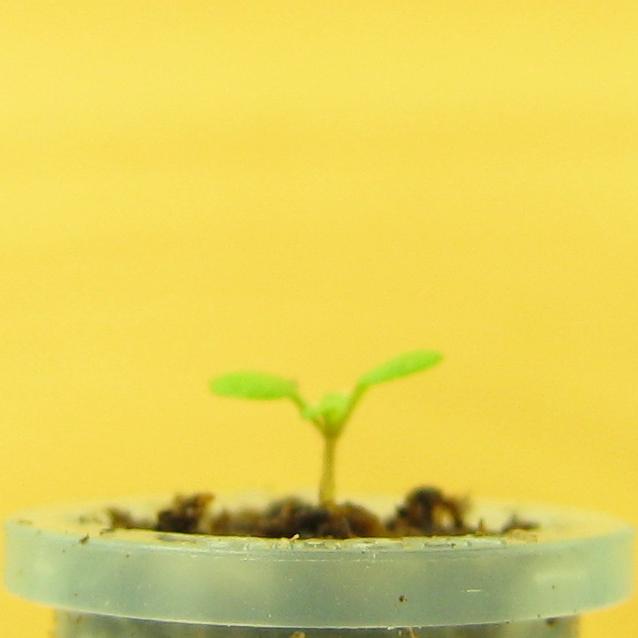

Supplement: Additional file 20 — Col-0 Front View Images for 3-D Model. Images of Col-0 captured every 10 min for 5 days from the front view for the 3-D CG model. Table S2 lists the images used as key frames in the model. [file 13007_2015_75_MOESM20_ESM.zip › front_view/side12_0092.jpg]

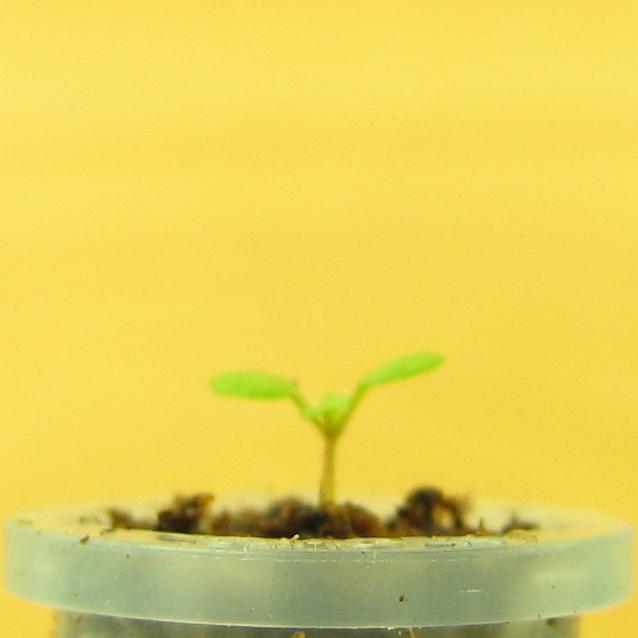

Supplement: Additional file 20 — Col-0 Front View Images for 3-D Model. Images of Col-0 captured every 10 min for 5 days from the front view for the 3-D CG model. Table S2 lists the images used as key frames in the model. [file 13007_2015_75_MOESM20_ESM.zip › front_view/side12_0093.jpg]

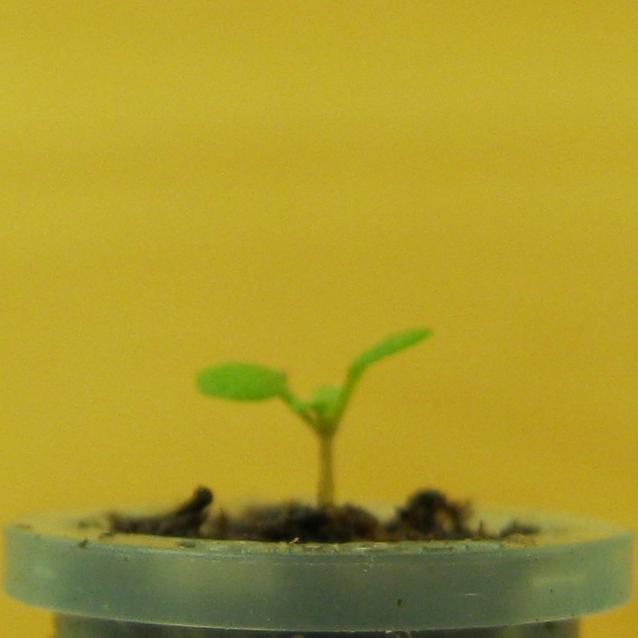

Supplement: Additional file 20 — Col-0 Front View Images for 3-D Model. Images of Col-0 captured every 10 min for 5 days from the front view for the 3-D CG model. Table S2 lists the images used as key frames in the model. [file 13007_2015_75_MOESM20_ESM.zip › front_view/side12_0094.jpg]

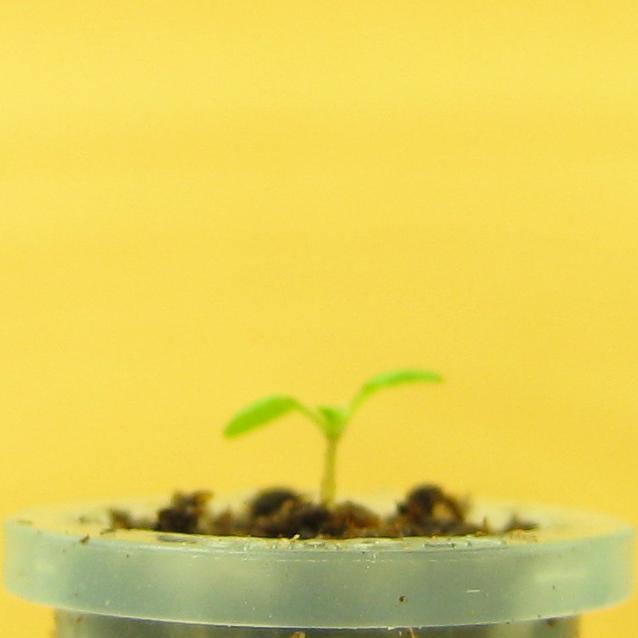

Supplement: Additional file 20 — Col-0 Front View Images for 3-D Model. Images of Col-0 captured every 10 min for 5 days from the front view for the 3-D CG model. Table S2 lists the images used as key frames in the model. [file 13007_2015_75_MOESM20_ESM.zip › front_view/side12_0095.jpg]

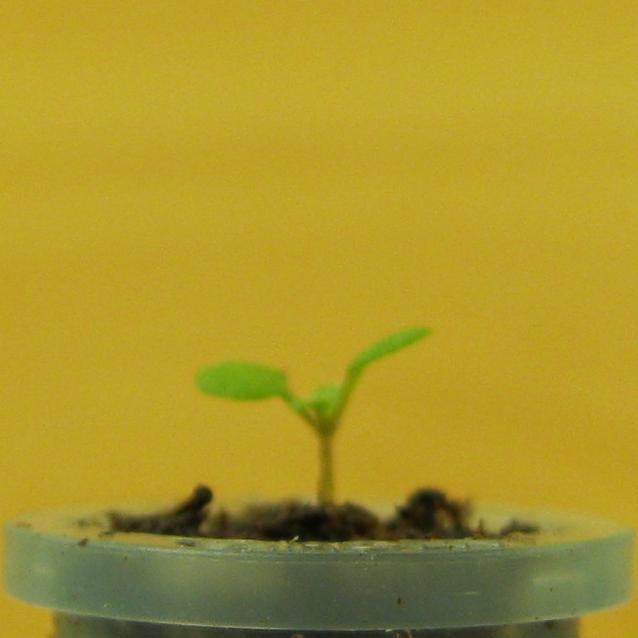

Supplement: Additional file 20 — Col-0 Front View Images for 3-D Model. Images of Col-0 captured every 10 min for 5 days from the front view for the 3-D CG model. Table S2 lists the images used as key frames in the model. [file 13007_2015_75_MOESM20_ESM.zip › front_view/side12_0117.jpg]

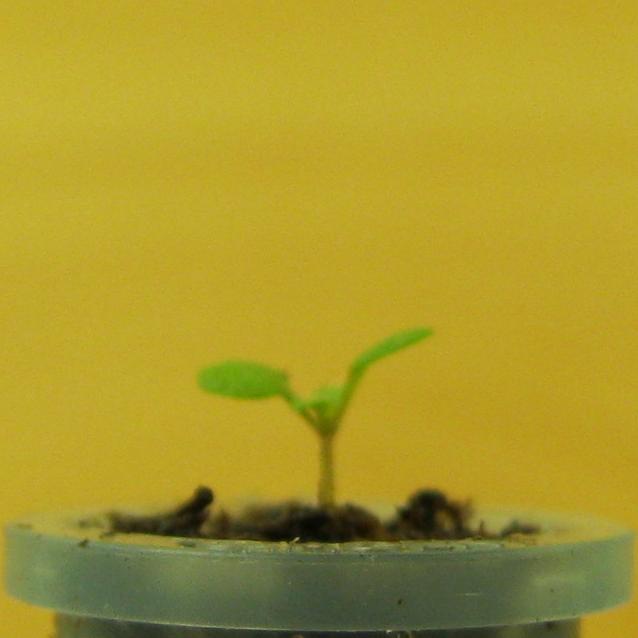

Supplement: Additional file 20 — Col-0 Front View Images for 3-D Model. Images of Col-0 captured every 10 min for 5 days from the front view for the 3-D CG model. Table S2 lists the images used as key frames in the model. [file 13007_2015_75_MOESM20_ESM.zip › front_view/side12_0118.jpg]

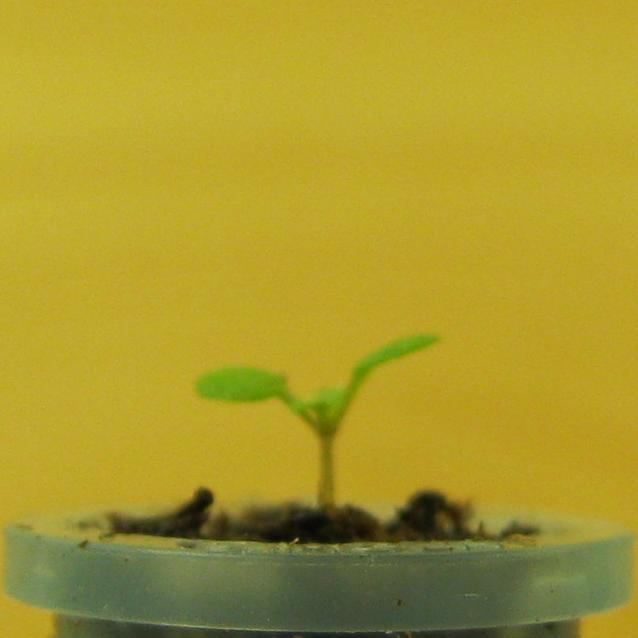

Supplement: Additional file 20 — Col-0 Front View Images for 3-D Model. Images of Col-0 captured every 10 min for 5 days from the front view for the 3-D CG model. Table S2 lists the images used as key frames in the model. [file 13007_2015_75_MOESM20_ESM.zip › front_view/side12_0119.jpg]

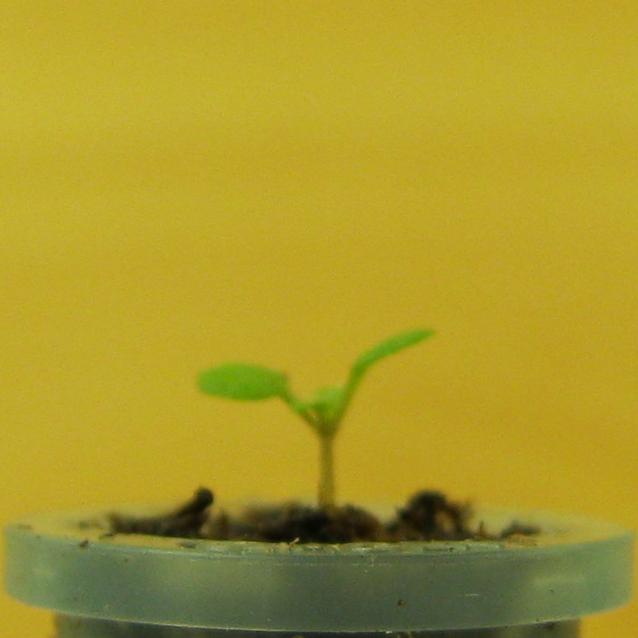

Supplement: Additional file 20 — Col-0 Front View Images for 3-D Model. Images of Col-0 captured every 10 min for 5 days from the front view for the 3-D CG model. Table S2 lists the images used as key frames in the model. [file 13007_2015_75_MOESM20_ESM.zip › front_view/side12_0120.jpg]

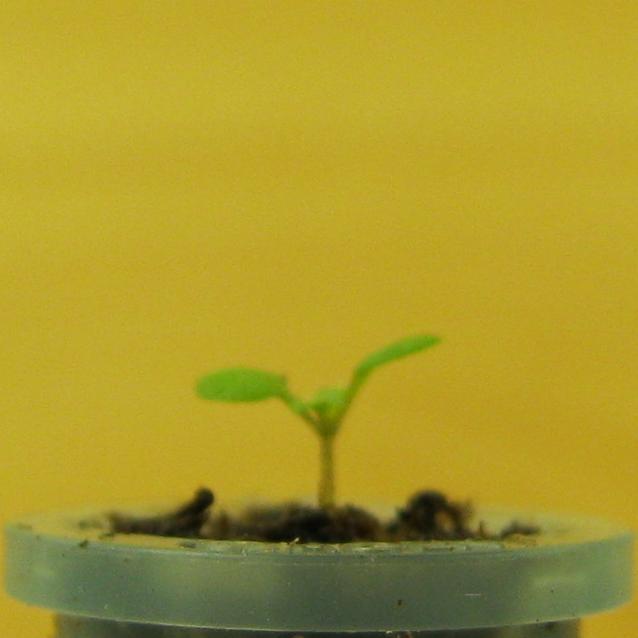

Supplement: Additional file 20 — Col-0 Front View Images for 3-D Model. Images of Col-0 captured every 10 min for 5 days from the front view for the 3-D CG model. Table S2 lists the images used as key frames in the model. [file 13007_2015_75_MOESM20_ESM.zip › front_view/side12_0121.jpg]

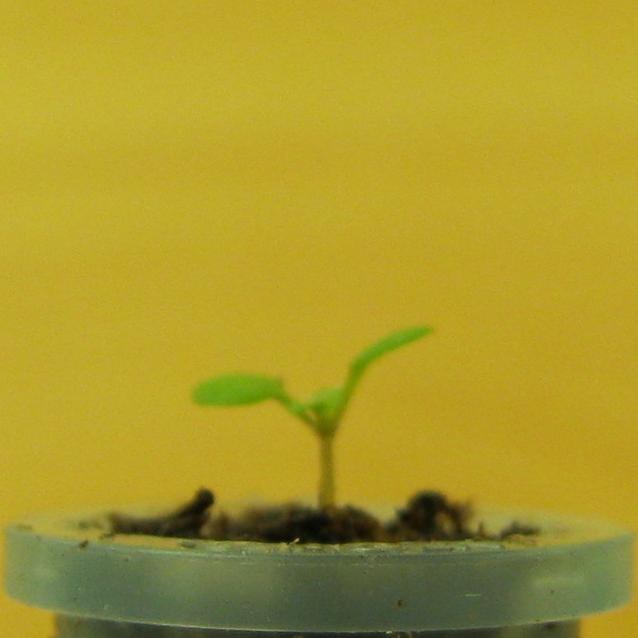

Supplement: Additional file 20 — Col-0 Front View Images for 3-D Model. Images of Col-0 captured every 10 min for 5 days from the front view for the 3-D CG model. Table S2 lists the images used as key frames in the model. [file 13007_2015_75_MOESM20_ESM.zip › front_view/side12_0122.jpg]

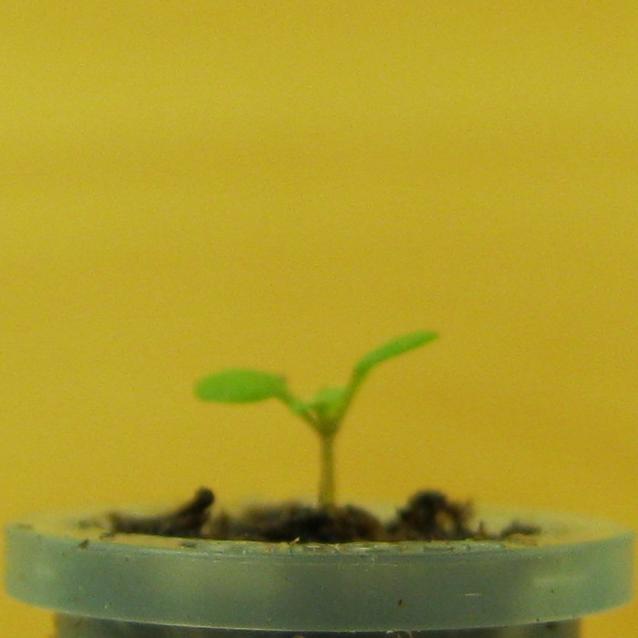

Supplement: Additional file 20 — Col-0 Front View Images for 3-D Model. Images of Col-0 captured every 10 min for 5 days from the front view for the 3-D CG model. Table S2 lists the images used as key frames in the model. [file 13007_2015_75_MOESM20_ESM.zip › front_view/side12_0123.jpg]

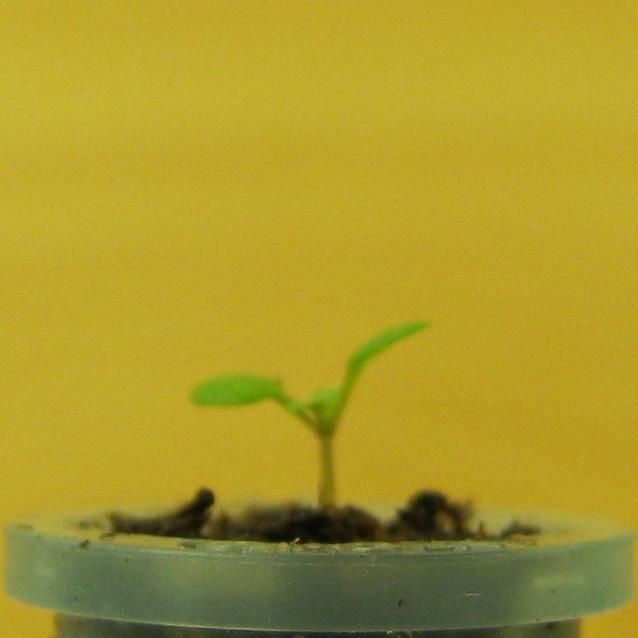

Supplement: Additional file 20 — Col-0 Front View Images for 3-D Model. Images of Col-0 captured every 10 min for 5 days from the front view for the 3-D CG model. Table S2 lists the images used as key frames in the model. [file 13007_2015_75_MOESM20_ESM.zip › front_view/side12_0124.jpg]

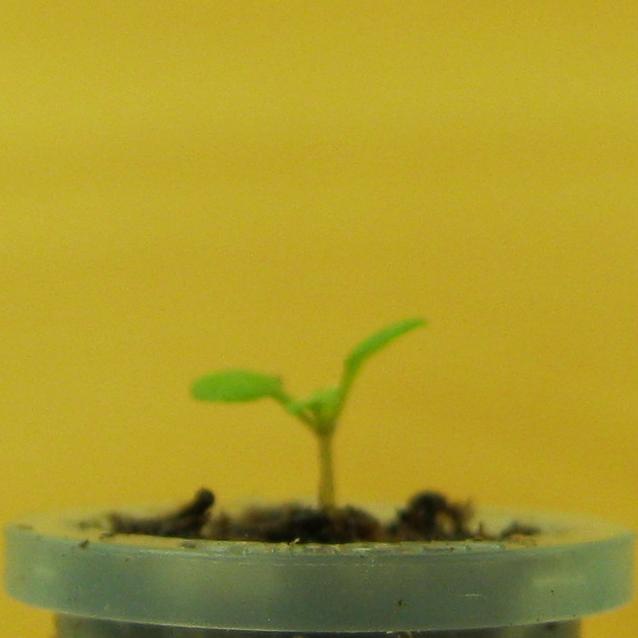

Supplement: Additional file 20 — Col-0 Front View Images for 3-D Model. Images of Col-0 captured every 10 min for 5 days from the front view for the 3-D CG model. Table S2 lists the images used as key frames in the model. [file 13007_2015_75_MOESM20_ESM.zip › front_view/side12_0125.jpg]

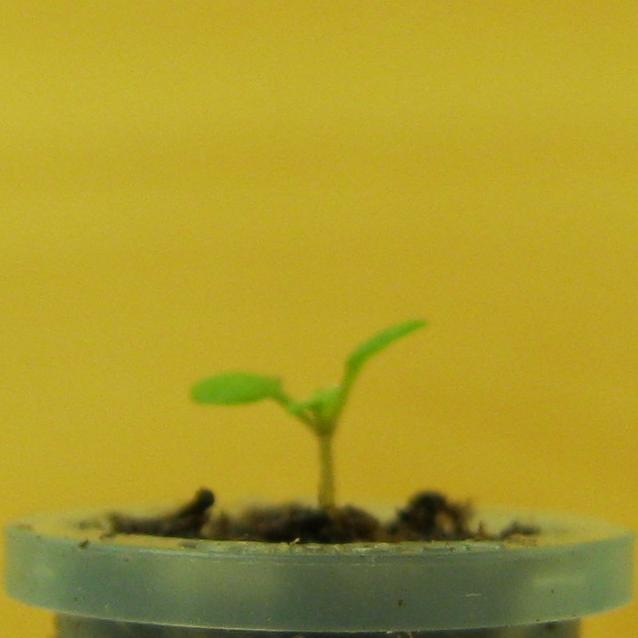

Supplement: Additional file 20 — Col-0 Front View Images for 3-D Model. Images of Col-0 captured every 10 min for 5 days from the front view for the 3-D CG model. Table S2 lists the images used as key frames in the model. [file 13007_2015_75_MOESM20_ESM.zip › front_view/side12_0126.jpg]

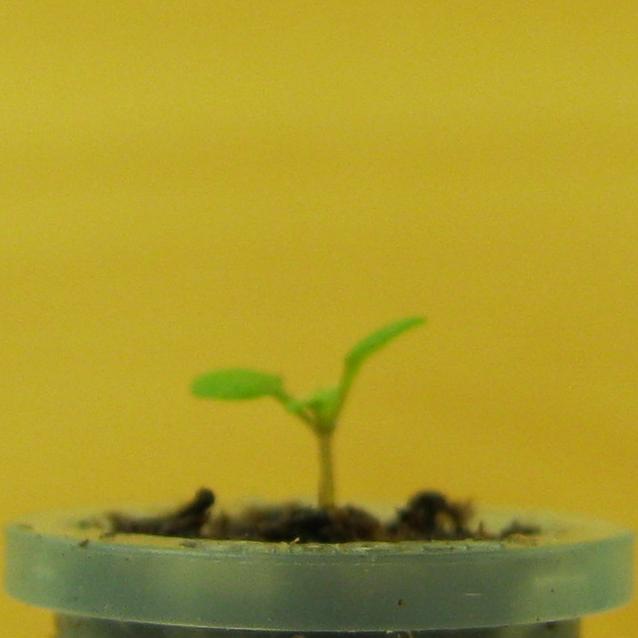

Supplement: Additional file 20 — Col-0 Front View Images for 3-D Model. Images of Col-0 captured every 10 min for 5 days from the front view for the 3-D CG model. Table S2 lists the images used as key frames in the model. [file 13007_2015_75_MOESM20_ESM.zip › front_view/side12_0127.jpg]

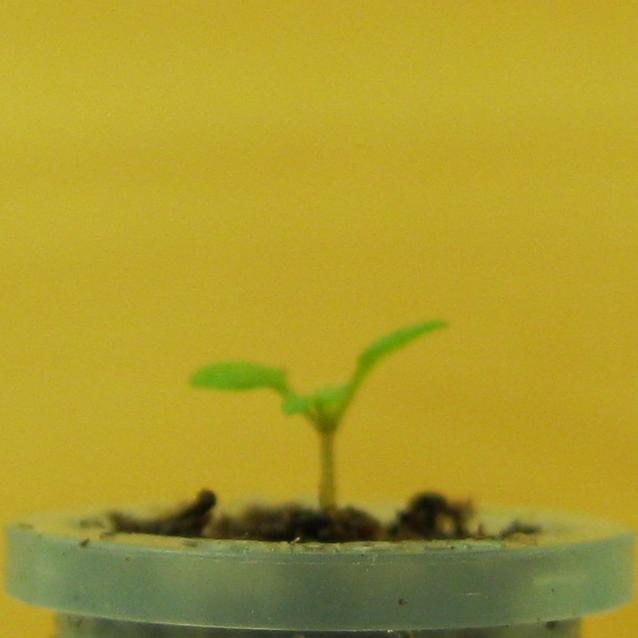

Supplement: Additional file 20 — Col-0 Front View Images for 3-D Model. Images of Col-0 captured every 10 min for 5 days from the front view for the 3-D CG model. Table S2 lists the images used as key frames in the model. [file 13007_2015_75_MOESM20_ESM.zip › front_view/side12_0128.jpg]

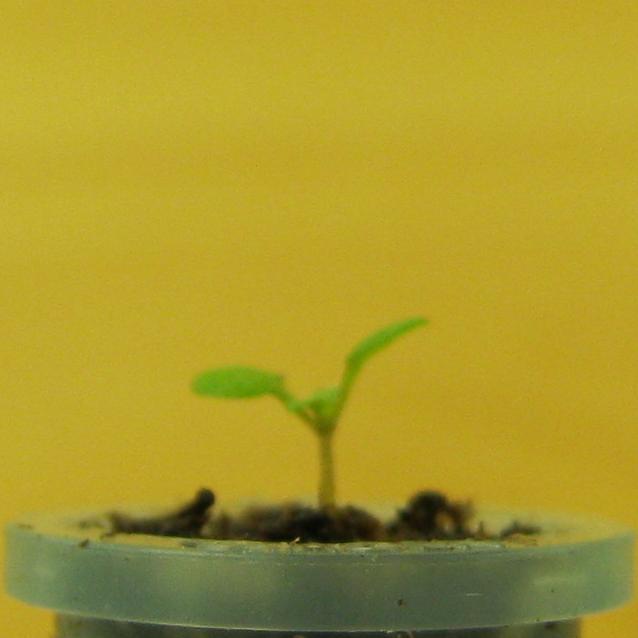

Supplement: Additional file 20 — Col-0 Front View Images for 3-D Model. Images of Col-0 captured every 10 min for 5 days from the front view for the 3-D CG model. Table S2 lists the images used as key frames in the model. [file 13007_2015_75_MOESM20_ESM.zip › front_view/side12_0129.jpg]
