# Supplementary figures and images for: TRiP: Tracking Rhythms in Plants, an automated leaf movement analysis program for circadian period estimation (part 8 of 10)
Source: Plant Methods. 2015 May 3;11:33. doi: 10.1186/s13007-015-0075-5 (PMC4445800; doi:10.1186/s13007-015-0075-5)

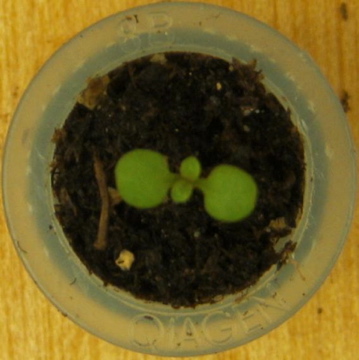

Supplement: Additional file 21 — Col-0 Top View Images for 3-D Model. First half of images of Col-0 captured every 10 min for 5 days from the top view for the 3-D CG model. Table S2 lists the images used as key frames in the model. [file 13007_2015_75_MOESM21_ESM.zip › top_view_1/top_0109.jpg]

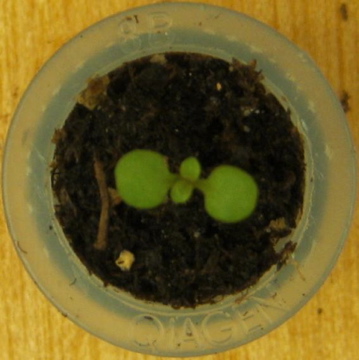

Supplement: Additional file 21 — Col-0 Top View Images for 3-D Model. First half of images of Col-0 captured every 10 min for 5 days from the top view for the 3-D CG model. Table S2 lists the images used as key frames in the model. [file 13007_2015_75_MOESM21_ESM.zip › top_view_1/top_0110.jpg]

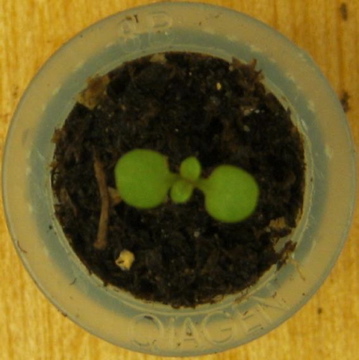

Supplement: Additional file 21 — Col-0 Top View Images for 3-D Model. First half of images of Col-0 captured every 10 min for 5 days from the top view for the 3-D CG model. Table S2 lists the images used as key frames in the model. [file 13007_2015_75_MOESM21_ESM.zip › top_view_1/top_0111.jpg]

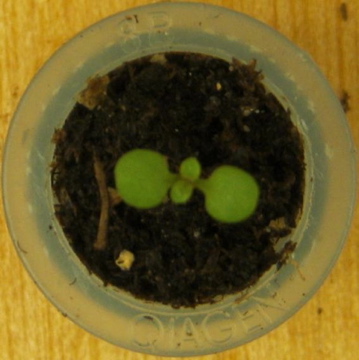

Supplement: Additional file 21 — Col-0 Top View Images for 3-D Model. First half of images of Col-0 captured every 10 min for 5 days from the top view for the 3-D CG model. Table S2 lists the images used as key frames in the model. [file 13007_2015_75_MOESM21_ESM.zip › top_view_1/top_0112.jpg]

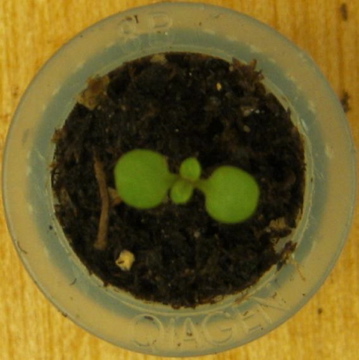

Supplement: Additional file 21 — Col-0 Top View Images for 3-D Model. First half of images of Col-0 captured every 10 min for 5 days from the top view for the 3-D CG model. Table S2 lists the images used as key frames in the model. [file 13007_2015_75_MOESM21_ESM.zip › top_view_1/top_0113.jpg]

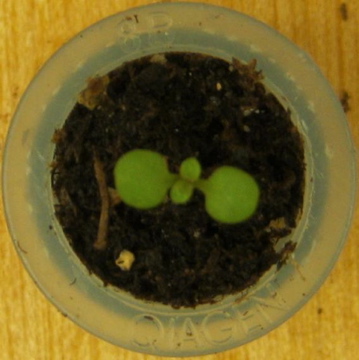

Supplement: Additional file 21 — Col-0 Top View Images for 3-D Model. First half of images of Col-0 captured every 10 min for 5 days from the top view for the 3-D CG model. Table S2 lists the images used as key frames in the model. [file 13007_2015_75_MOESM21_ESM.zip › top_view_1/top_0114.jpg]

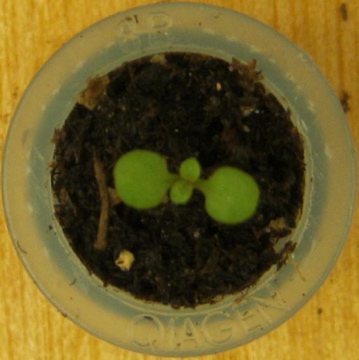

Supplement: Additional file 21 — Col-0 Top View Images for 3-D Model. First half of images of Col-0 captured every 10 min for 5 days from the top view for the 3-D CG model. Table S2 lists the images used as key frames in the model. [file 13007_2015_75_MOESM21_ESM.zip › top_view_1/top_0115.jpg]

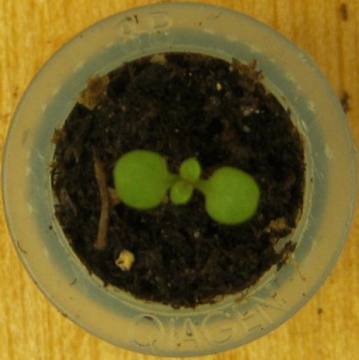

Supplement: Additional file 21 — Col-0 Top View Images for 3-D Model. First half of images of Col-0 captured every 10 min for 5 days from the top view for the 3-D CG model. Table S2 lists the images used as key frames in the model. [file 13007_2015_75_MOESM21_ESM.zip › top_view_1/top_0116.jpg]

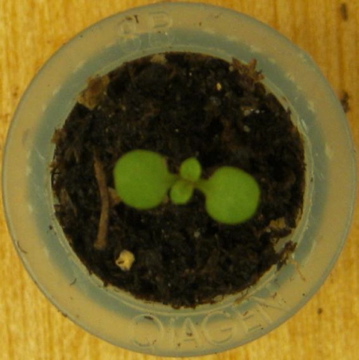

Supplement: Additional file 21 — Col-0 Top View Images for 3-D Model. First half of images of Col-0 captured every 10 min for 5 days from the top view for the 3-D CG model. Table S2 lists the images used as key frames in the model. [file 13007_2015_75_MOESM21_ESM.zip › top_view_1/top_0117.jpg]

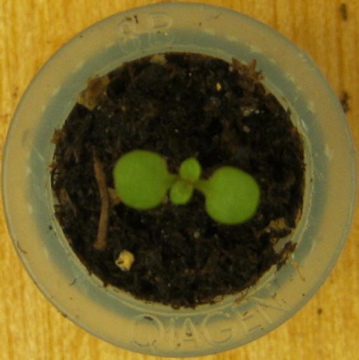

Supplement: Additional file 21 — Col-0 Top View Images for 3-D Model. First half of images of Col-0 captured every 10 min for 5 days from the top view for the 3-D CG model. Table S2 lists the images used as key frames in the model. [file 13007_2015_75_MOESM21_ESM.zip › top_view_1/top_0118.jpg]

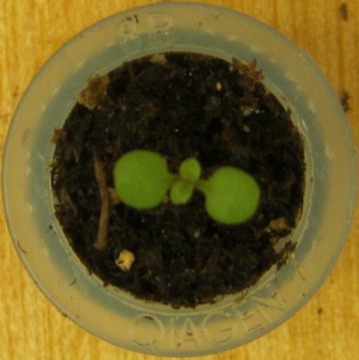

Supplement: Additional file 21 — Col-0 Top View Images for 3-D Model. First half of images of Col-0 captured every 10 min for 5 days from the top view for the 3-D CG model. Table S2 lists the images used as key frames in the model. [file 13007_2015_75_MOESM21_ESM.zip › top_view_1/top_0119.jpg]

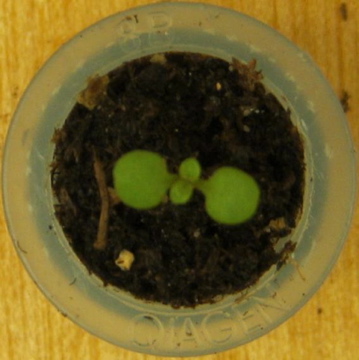

Supplement: Additional file 21 — Col-0 Top View Images for 3-D Model. First half of images of Col-0 captured every 10 min for 5 days from the top view for the 3-D CG model. Table S2 lists the images used as key frames in the model. [file 13007_2015_75_MOESM21_ESM.zip › top_view_1/top_0120.jpg]

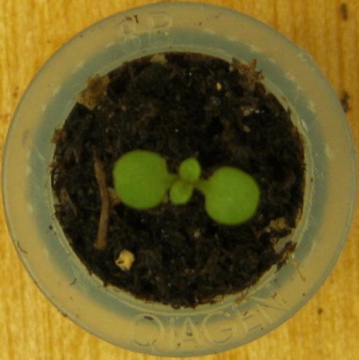

Supplement: Additional file 21 — Col-0 Top View Images for 3-D Model. First half of images of Col-0 captured every 10 min for 5 days from the top view for the 3-D CG model. Table S2 lists the images used as key frames in the model. [file 13007_2015_75_MOESM21_ESM.zip › top_view_1/top_0121.jpg]

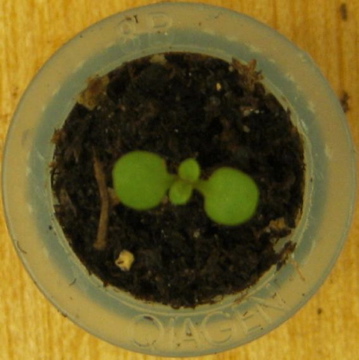

Supplement: Additional file 21 — Col-0 Top View Images for 3-D Model. First half of images of Col-0 captured every 10 min for 5 days from the top view for the 3-D CG model. Table S2 lists the images used as key frames in the model. [file 13007_2015_75_MOESM21_ESM.zip › top_view_1/top_0122.jpg]

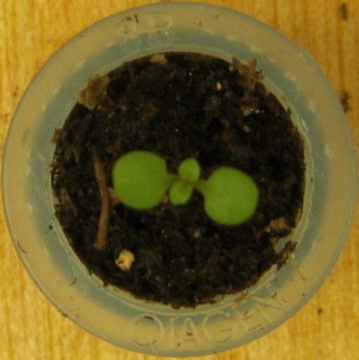

Supplement: Additional file 21 — Col-0 Top View Images for 3-D Model. First half of images of Col-0 captured every 10 min for 5 days from the top view for the 3-D CG model. Table S2 lists the images used as key frames in the model. [file 13007_2015_75_MOESM21_ESM.zip › top_view_1/top_0123.jpg]

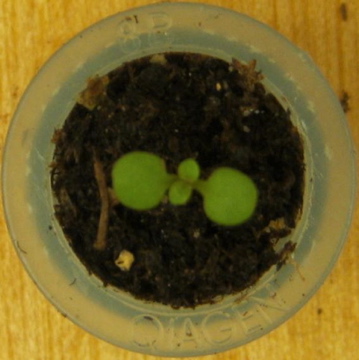

Supplement: Additional file 21 — Col-0 Top View Images for 3-D Model. First half of images of Col-0 captured every 10 min for 5 days from the top view for the 3-D CG model. Table S2 lists the images used as key frames in the model. [file 13007_2015_75_MOESM21_ESM.zip › top_view_1/top_0124.jpg]

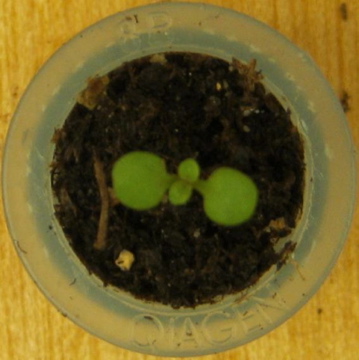

Supplement: Additional file 21 — Col-0 Top View Images for 3-D Model. First half of images of Col-0 captured every 10 min for 5 days from the top view for the 3-D CG model. Table S2 lists the images used as key frames in the model. [file 13007_2015_75_MOESM21_ESM.zip › top_view_1/top_0125.jpg]

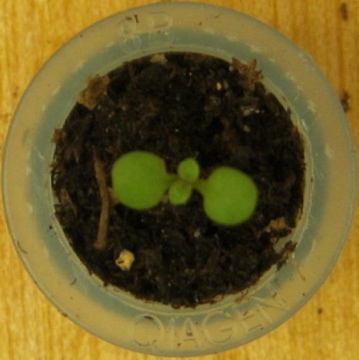

Supplement: Additional file 21 — Col-0 Top View Images for 3-D Model. First half of images of Col-0 captured every 10 min for 5 days from the top view for the 3-D CG model. Table S2 lists the images used as key frames in the model. [file 13007_2015_75_MOESM21_ESM.zip › top_view_1/top_0126.jpg]

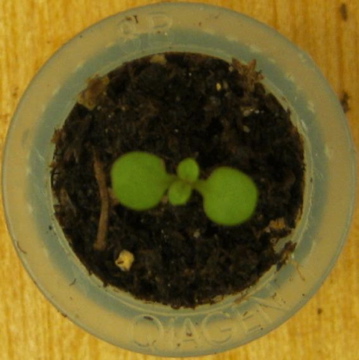

Supplement: Additional file 21 — Col-0 Top View Images for 3-D Model. First half of images of Col-0 captured every 10 min for 5 days from the top view for the 3-D CG model. Table S2 lists the images used as key frames in the model. [file 13007_2015_75_MOESM21_ESM.zip › top_view_1/top_0127.jpg]

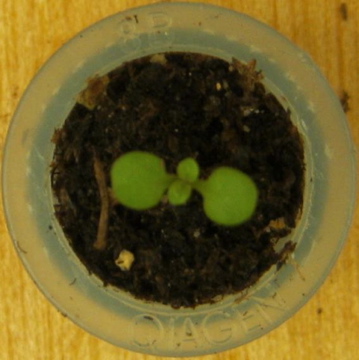

Supplement: Additional file 21 — Col-0 Top View Images for 3-D Model. First half of images of Col-0 captured every 10 min for 5 days from the top view for the 3-D CG model. Table S2 lists the images used as key frames in the model. [file 13007_2015_75_MOESM21_ESM.zip › top_view_1/top_0128.jpg]

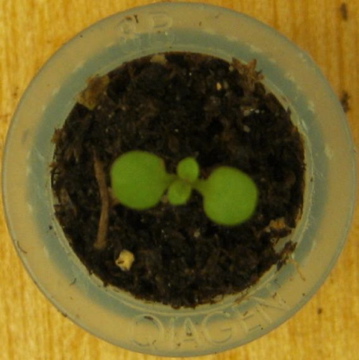

Supplement: Additional file 21 — Col-0 Top View Images for 3-D Model. First half of images of Col-0 captured every 10 min for 5 days from the top view for the 3-D CG model. Table S2 lists the images used as key frames in the model. [file 13007_2015_75_MOESM21_ESM.zip › top_view_1/top_0129.jpg]

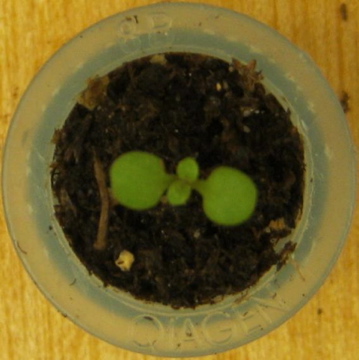

Supplement: Additional file 21 — Col-0 Top View Images for 3-D Model. First half of images of Col-0 captured every 10 min for 5 days from the top view for the 3-D CG model. Table S2 lists the images used as key frames in the model. [file 13007_2015_75_MOESM21_ESM.zip › top_view_1/top_0130.jpg]

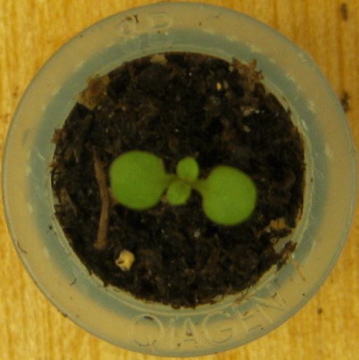

Supplement: Additional file 21 — Col-0 Top View Images for 3-D Model. First half of images of Col-0 captured every 10 min for 5 days from the top view for the 3-D CG model. Table S2 lists the images used as key frames in the model. [file 13007_2015_75_MOESM21_ESM.zip › top_view_1/top_0131.jpg]

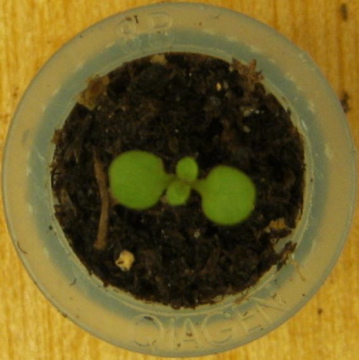

Supplement: Additional file 21 — Col-0 Top View Images for 3-D Model. First half of images of Col-0 captured every 10 min for 5 days from the top view for the 3-D CG model. Table S2 lists the images used as key frames in the model. [file 13007_2015_75_MOESM21_ESM.zip › top_view_1/top_0132.jpg]

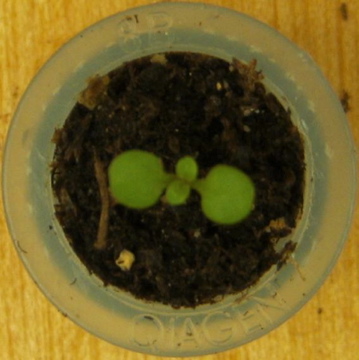

Supplement: Additional file 21 — Col-0 Top View Images for 3-D Model. First half of images of Col-0 captured every 10 min for 5 days from the top view for the 3-D CG model. Table S2 lists the images used as key frames in the model. [file 13007_2015_75_MOESM21_ESM.zip › top_view_1/top_0133.jpg]

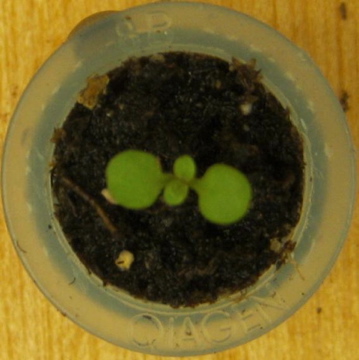

Supplement: Additional file 21 — Col-0 Top View Images for 3-D Model. First half of images of Col-0 captured every 10 min for 5 days from the top view for the 3-D CG model. Table S2 lists the images used as key frames in the model. [file 13007_2015_75_MOESM21_ESM.zip › top_view_1/top_0134.jpg]

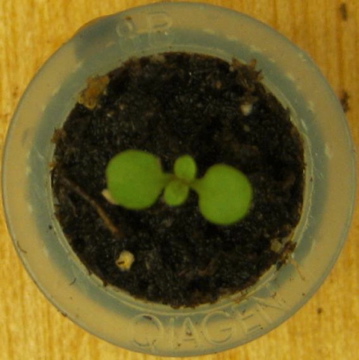

Supplement: Additional file 21 — Col-0 Top View Images for 3-D Model. First half of images of Col-0 captured every 10 min for 5 days from the top view for the 3-D CG model. Table S2 lists the images used as key frames in the model. [file 13007_2015_75_MOESM21_ESM.zip › top_view_1/top_0135.jpg]

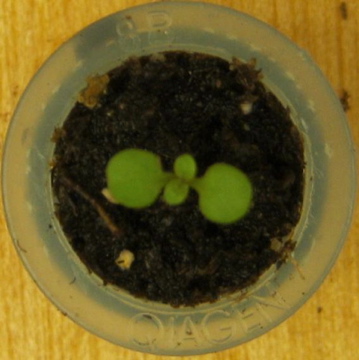

Supplement: Additional file 21 — Col-0 Top View Images for 3-D Model. First half of images of Col-0 captured every 10 min for 5 days from the top view for the 3-D CG model. Table S2 lists the images used as key frames in the model. [file 13007_2015_75_MOESM21_ESM.zip › top_view_1/top_0136.jpg]

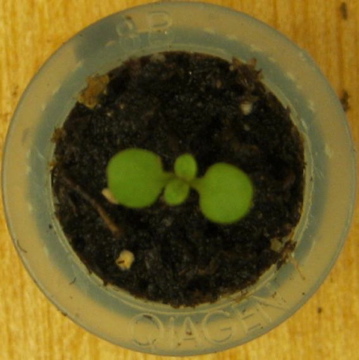

Supplement: Additional file 21 — Col-0 Top View Images for 3-D Model. First half of images of Col-0 captured every 10 min for 5 days from the top view for the 3-D CG model. Table S2 lists the images used as key frames in the model. [file 13007_2015_75_MOESM21_ESM.zip › top_view_1/top_0137.jpg]

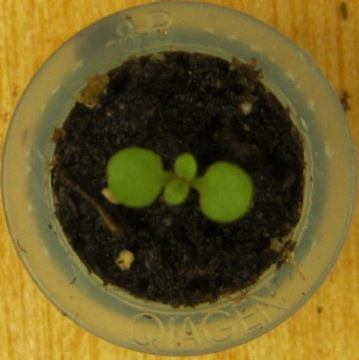

Supplement: Additional file 21 — Col-0 Top View Images for 3-D Model. First half of images of Col-0 captured every 10 min for 5 days from the top view for the 3-D CG model. Table S2 lists the images used as key frames in the model. [file 13007_2015_75_MOESM21_ESM.zip › top_view_1/top_0138.jpg]

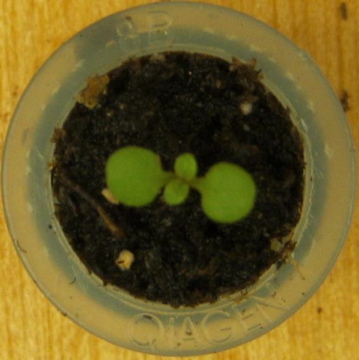

Supplement: Additional file 21 — Col-0 Top View Images for 3-D Model. First half of images of Col-0 captured every 10 min for 5 days from the top view for the 3-D CG model. Table S2 lists the images used as key frames in the model. [file 13007_2015_75_MOESM21_ESM.zip › top_view_1/top_0139.jpg]

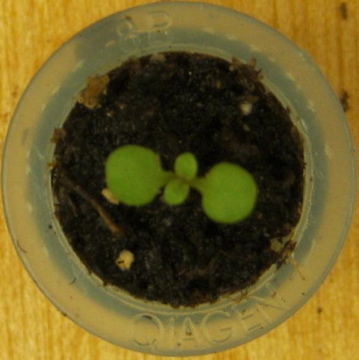

Supplement: Additional file 21 — Col-0 Top View Images for 3-D Model. First half of images of Col-0 captured every 10 min for 5 days from the top view for the 3-D CG model. Table S2 lists the images used as key frames in the model. [file 13007_2015_75_MOESM21_ESM.zip › top_view_1/top_0140.jpg]

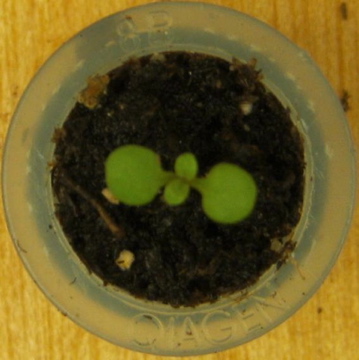

Supplement: Additional file 21 — Col-0 Top View Images for 3-D Model. First half of images of Col-0 captured every 10 min for 5 days from the top view for the 3-D CG model. Table S2 lists the images used as key frames in the model. [file 13007_2015_75_MOESM21_ESM.zip › top_view_1/top_0141.jpg]

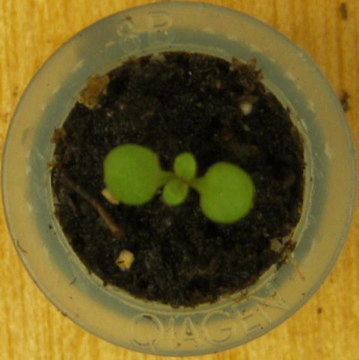

Supplement: Additional file 21 — Col-0 Top View Images for 3-D Model. First half of images of Col-0 captured every 10 min for 5 days from the top view for the 3-D CG model. Table S2 lists the images used as key frames in the model. [file 13007_2015_75_MOESM21_ESM.zip › top_view_1/top_0142.jpg]

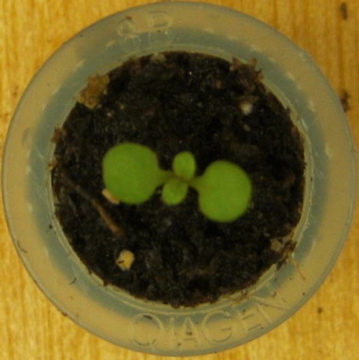

Supplement: Additional file 21 — Col-0 Top View Images for 3-D Model. First half of images of Col-0 captured every 10 min for 5 days from the top view for the 3-D CG model. Table S2 lists the images used as key frames in the model. [file 13007_2015_75_MOESM21_ESM.zip › top_view_1/top_0143.jpg]

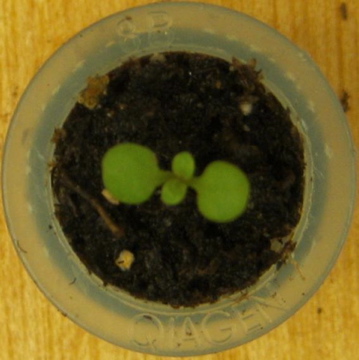

Supplement: Additional file 21 — Col-0 Top View Images for 3-D Model. First half of images of Col-0 captured every 10 min for 5 days from the top view for the 3-D CG model. Table S2 lists the images used as key frames in the model. [file 13007_2015_75_MOESM21_ESM.zip › top_view_1/top_0144.jpg]

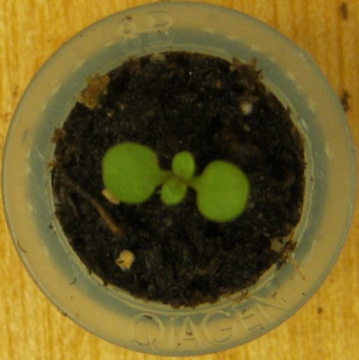

Supplement: Additional file 21 — Col-0 Top View Images for 3-D Model. First half of images of Col-0 captured every 10 min for 5 days from the top view for the 3-D CG model. Table S2 lists the images used as key frames in the model. [file 13007_2015_75_MOESM21_ESM.zip › top_view_1/top_0145.jpg]

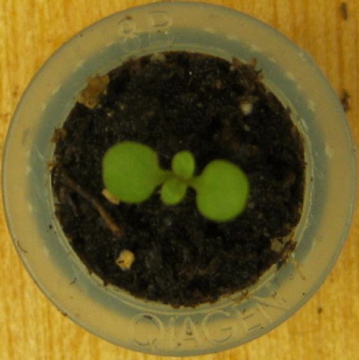

Supplement: Additional file 21 — Col-0 Top View Images for 3-D Model. First half of images of Col-0 captured every 10 min for 5 days from the top view for the 3-D CG model. Table S2 lists the images used as key frames in the model. [file 13007_2015_75_MOESM21_ESM.zip › top_view_1/top_0146.jpg]

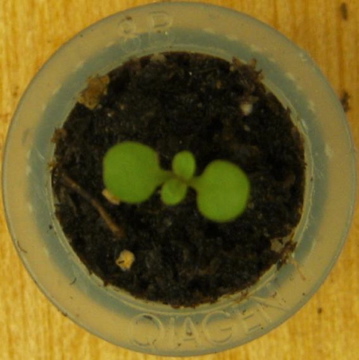

Supplement: Additional file 21 — Col-0 Top View Images for 3-D Model. First half of images of Col-0 captured every 10 min for 5 days from the top view for the 3-D CG model. Table S2 lists the images used as key frames in the model. [file 13007_2015_75_MOESM21_ESM.zip › top_view_1/top_0147.jpg]

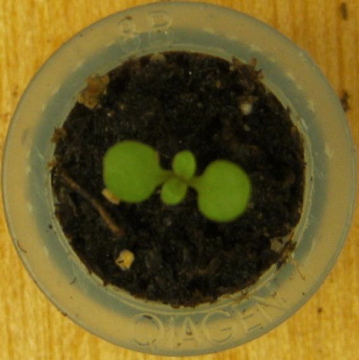

Supplement: Additional file 21 — Col-0 Top View Images for 3-D Model. First half of images of Col-0 captured every 10 min for 5 days from the top view for the 3-D CG model. Table S2 lists the images used as key frames in the model. [file 13007_2015_75_MOESM21_ESM.zip › top_view_1/top_0148.jpg]

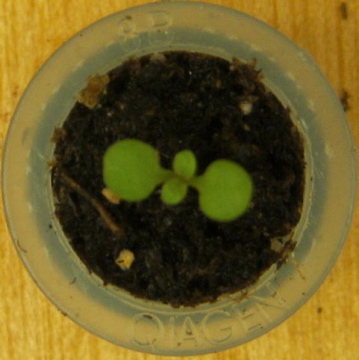

Supplement: Additional file 21 — Col-0 Top View Images for 3-D Model. First half of images of Col-0 captured every 10 min for 5 days from the top view for the 3-D CG model. Table S2 lists the images used as key frames in the model. [file 13007_2015_75_MOESM21_ESM.zip › top_view_1/top_0149.jpg]

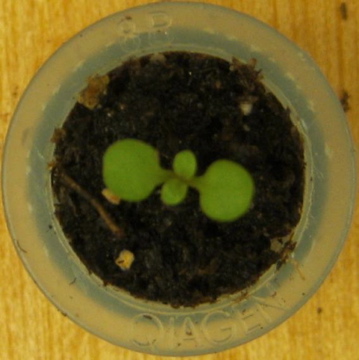

Supplement: Additional file 21 — Col-0 Top View Images for 3-D Model. First half of images of Col-0 captured every 10 min for 5 days from the top view for the 3-D CG model. Table S2 lists the images used as key frames in the model. [file 13007_2015_75_MOESM21_ESM.zip › top_view_1/top_0150.jpg]

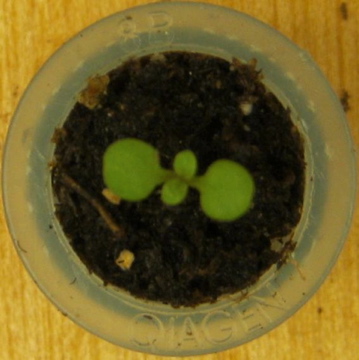

Supplement: Additional file 21 — Col-0 Top View Images for 3-D Model. First half of images of Col-0 captured every 10 min for 5 days from the top view for the 3-D CG model. Table S2 lists the images used as key frames in the model. [file 13007_2015_75_MOESM21_ESM.zip › top_view_1/top_0151.jpg]

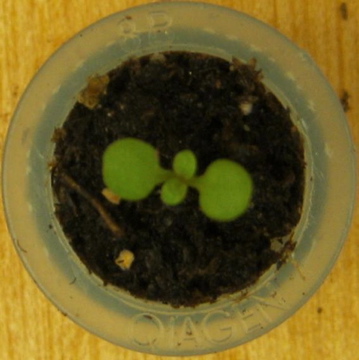

Supplement: Additional file 21 — Col-0 Top View Images for 3-D Model. First half of images of Col-0 captured every 10 min for 5 days from the top view for the 3-D CG model. Table S2 lists the images used as key frames in the model. [file 13007_2015_75_MOESM21_ESM.zip › top_view_1/top_0152.jpg]

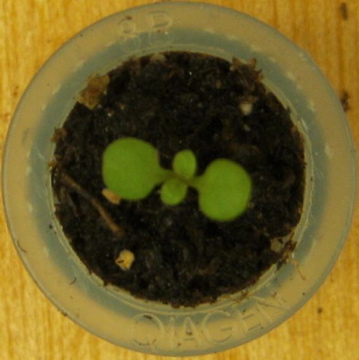

Supplement: Additional file 21 — Col-0 Top View Images for 3-D Model. First half of images of Col-0 captured every 10 min for 5 days from the top view for the 3-D CG model. Table S2 lists the images used as key frames in the model. [file 13007_2015_75_MOESM21_ESM.zip › top_view_1/top_0153.jpg]

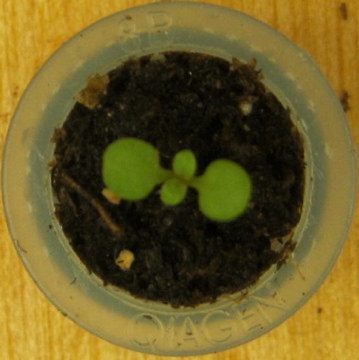

Supplement: Additional file 21 — Col-0 Top View Images for 3-D Model. First half of images of Col-0 captured every 10 min for 5 days from the top view for the 3-D CG model. Table S2 lists the images used as key frames in the model. [file 13007_2015_75_MOESM21_ESM.zip › top_view_1/top_0154.jpg]

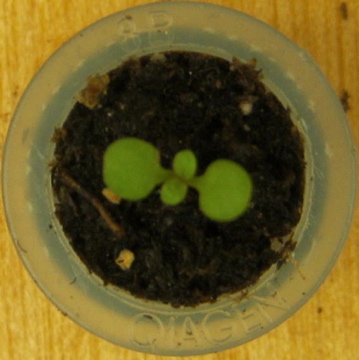

Supplement: Additional file 21 — Col-0 Top View Images for 3-D Model. First half of images of Col-0 captured every 10 min for 5 days from the top view for the 3-D CG model. Table S2 lists the images used as key frames in the model. [file 13007_2015_75_MOESM21_ESM.zip › top_view_1/top_0155.jpg]

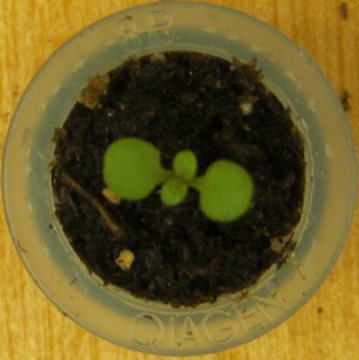

Supplement: Additional file 21 — Col-0 Top View Images for 3-D Model. First half of images of Col-0 captured every 10 min for 5 days from the top view for the 3-D CG model. Table S2 lists the images used as key frames in the model. [file 13007_2015_75_MOESM21_ESM.zip › top_view_1/top_0156.jpg]

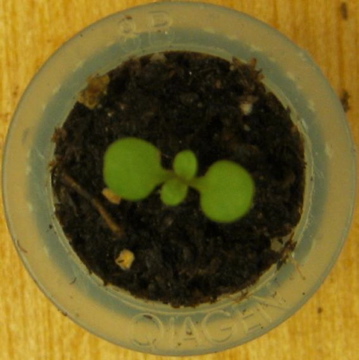

Supplement: Additional file 21 — Col-0 Top View Images for 3-D Model. First half of images of Col-0 captured every 10 min for 5 days from the top view for the 3-D CG model. Table S2 lists the images used as key frames in the model. [file 13007_2015_75_MOESM21_ESM.zip › top_view_1/top_0157.jpg]

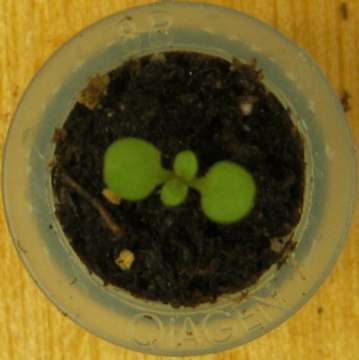

Supplement: Additional file 21 — Col-0 Top View Images for 3-D Model. First half of images of Col-0 captured every 10 min for 5 days from the top view for the 3-D CG model. Table S2 lists the images used as key frames in the model. [file 13007_2015_75_MOESM21_ESM.zip › top_view_1/top_0158.jpg]

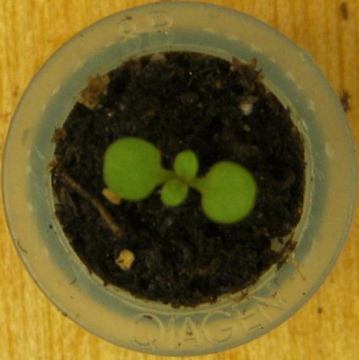

Supplement: Additional file 21 — Col-0 Top View Images for 3-D Model. First half of images of Col-0 captured every 10 min for 5 days from the top view for the 3-D CG model. Table S2 lists the images used as key frames in the model. [file 13007_2015_75_MOESM21_ESM.zip › top_view_1/top_0159.jpg]

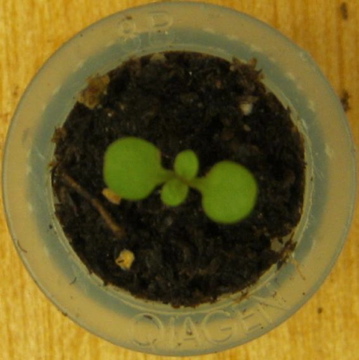

Supplement: Additional file 21 — Col-0 Top View Images for 3-D Model. First half of images of Col-0 captured every 10 min for 5 days from the top view for the 3-D CG model. Table S2 lists the images used as key frames in the model. [file 13007_2015_75_MOESM21_ESM.zip › top_view_1/top_0160.jpg]

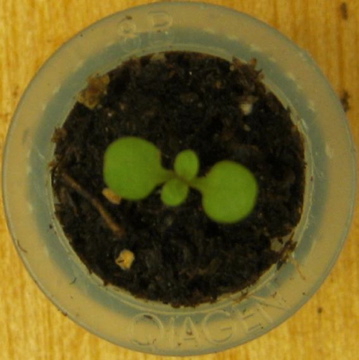

Supplement: Additional file 21 — Col-0 Top View Images for 3-D Model. First half of images of Col-0 captured every 10 min for 5 days from the top view for the 3-D CG model. Table S2 lists the images used as key frames in the model. [file 13007_2015_75_MOESM21_ESM.zip › top_view_1/top_0161.jpg]

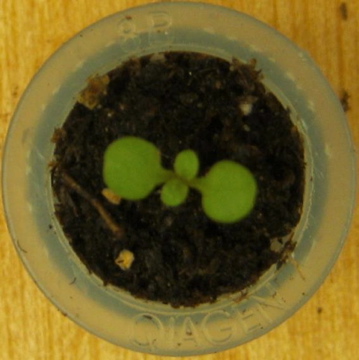

Supplement: Additional file 21 — Col-0 Top View Images for 3-D Model. First half of images of Col-0 captured every 10 min for 5 days from the top view for the 3-D CG model. Table S2 lists the images used as key frames in the model. [file 13007_2015_75_MOESM21_ESM.zip › top_view_1/top_0162.jpg]

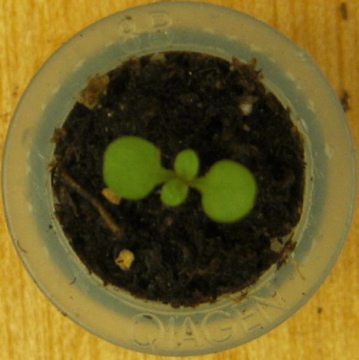

Supplement: Additional file 21 — Col-0 Top View Images for 3-D Model. First half of images of Col-0 captured every 10 min for 5 days from the top view for the 3-D CG model. Table S2 lists the images used as key frames in the model. [file 13007_2015_75_MOESM21_ESM.zip › top_view_1/top_0163.jpg]

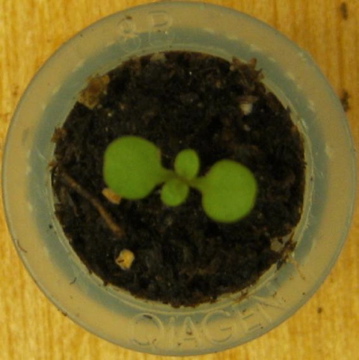

Supplement: Additional file 21 — Col-0 Top View Images for 3-D Model. First half of images of Col-0 captured every 10 min for 5 days from the top view for the 3-D CG model. Table S2 lists the images used as key frames in the model. [file 13007_2015_75_MOESM21_ESM.zip › top_view_1/top_0164.jpg]

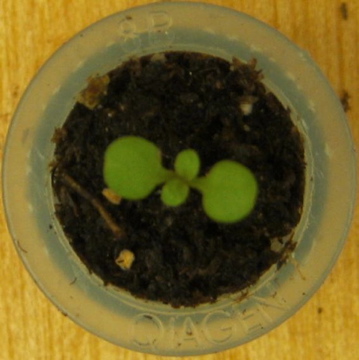

Supplement: Additional file 21 — Col-0 Top View Images for 3-D Model. First half of images of Col-0 captured every 10 min for 5 days from the top view for the 3-D CG model. Table S2 lists the images used as key frames in the model. [file 13007_2015_75_MOESM21_ESM.zip › top_view_1/top_0165.jpg]

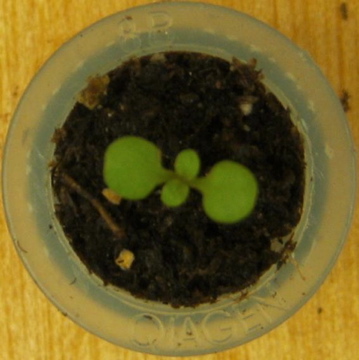

Supplement: Additional file 21 — Col-0 Top View Images for 3-D Model. First half of images of Col-0 captured every 10 min for 5 days from the top view for the 3-D CG model. Table S2 lists the images used as key frames in the model. [file 13007_2015_75_MOESM21_ESM.zip › top_view_1/top_0166.jpg]

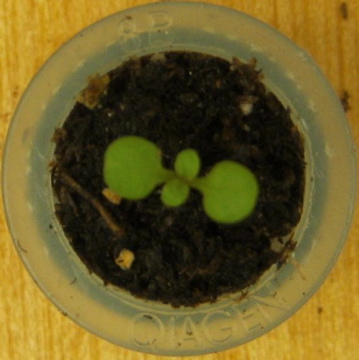

Supplement: Additional file 21 — Col-0 Top View Images for 3-D Model. First half of images of Col-0 captured every 10 min for 5 days from the top view for the 3-D CG model. Table S2 lists the images used as key frames in the model. [file 13007_2015_75_MOESM21_ESM.zip › top_view_1/top_0167.jpg]

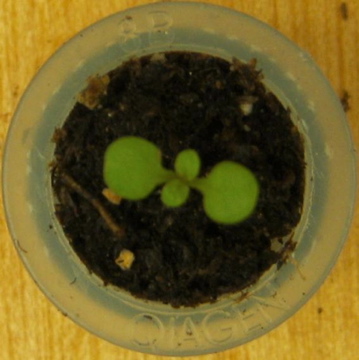

Supplement: Additional file 21 — Col-0 Top View Images for 3-D Model. First half of images of Col-0 captured every 10 min for 5 days from the top view for the 3-D CG model. Table S2 lists the images used as key frames in the model. [file 13007_2015_75_MOESM21_ESM.zip › top_view_1/top_0168.jpg]

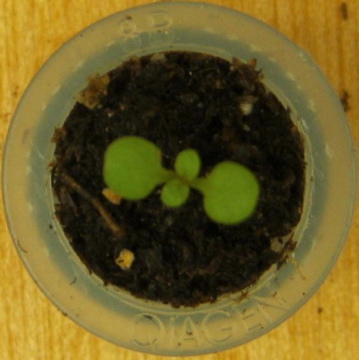

Supplement: Additional file 21 — Col-0 Top View Images for 3-D Model. First half of images of Col-0 captured every 10 min for 5 days from the top view for the 3-D CG model. Table S2 lists the images used as key frames in the model. [file 13007_2015_75_MOESM21_ESM.zip › top_view_1/top_0169.jpg]

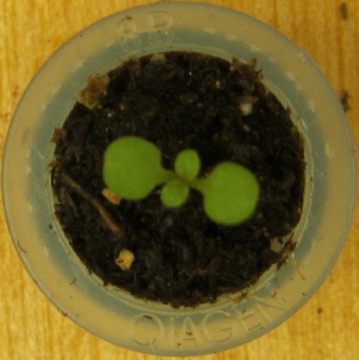

Supplement: Additional file 21 — Col-0 Top View Images for 3-D Model. First half of images of Col-0 captured every 10 min for 5 days from the top view for the 3-D CG model. Table S2 lists the images used as key frames in the model. [file 13007_2015_75_MOESM21_ESM.zip › top_view_1/top_0170.jpg]

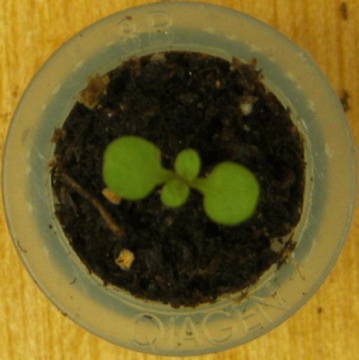

Supplement: Additional file 21 — Col-0 Top View Images for 3-D Model. First half of images of Col-0 captured every 10 min for 5 days from the top view for the 3-D CG model. Table S2 lists the images used as key frames in the model. [file 13007_2015_75_MOESM21_ESM.zip › top_view_1/top_0171.jpg]

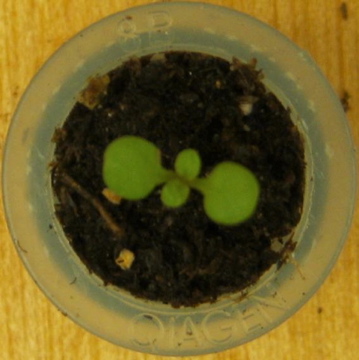

Supplement: Additional file 21 — Col-0 Top View Images for 3-D Model. First half of images of Col-0 captured every 10 min for 5 days from the top view for the 3-D CG model. Table S2 lists the images used as key frames in the model. [file 13007_2015_75_MOESM21_ESM.zip › top_view_1/top_0172.jpg]

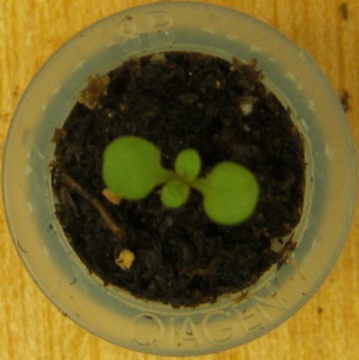

Supplement: Additional file 21 — Col-0 Top View Images for 3-D Model. First half of images of Col-0 captured every 10 min for 5 days from the top view for the 3-D CG model. Table S2 lists the images used as key frames in the model. [file 13007_2015_75_MOESM21_ESM.zip › top_view_1/top_0173.jpg]

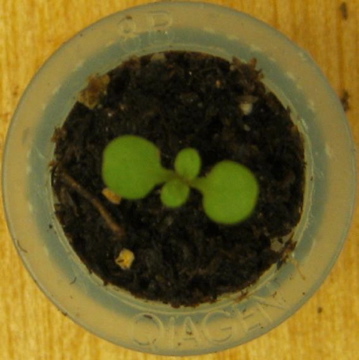

Supplement: Additional file 21 — Col-0 Top View Images for 3-D Model. First half of images of Col-0 captured every 10 min for 5 days from the top view for the 3-D CG model. Table S2 lists the images used as key frames in the model. [file 13007_2015_75_MOESM21_ESM.zip › top_view_1/top_0174.jpg]

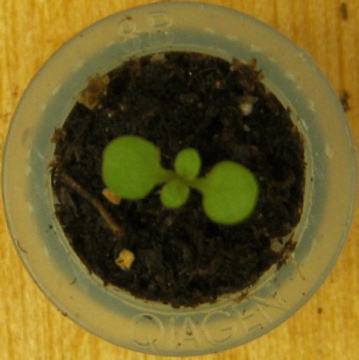

Supplement: Additional file 21 — Col-0 Top View Images for 3-D Model. First half of images of Col-0 captured every 10 min for 5 days from the top view for the 3-D CG model. Table S2 lists the images used as key frames in the model. [file 13007_2015_75_MOESM21_ESM.zip › top_view_1/top_0175.jpg]

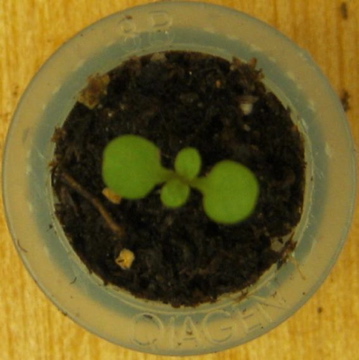

Supplement: Additional file 21 — Col-0 Top View Images for 3-D Model. First half of images of Col-0 captured every 10 min for 5 days from the top view for the 3-D CG model. Table S2 lists the images used as key frames in the model. [file 13007_2015_75_MOESM21_ESM.zip › top_view_1/top_0176.jpg]

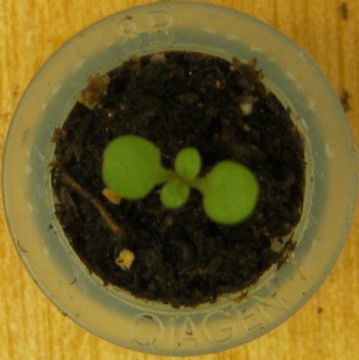

Supplement: Additional file 21 — Col-0 Top View Images for 3-D Model. First half of images of Col-0 captured every 10 min for 5 days from the top view for the 3-D CG model. Table S2 lists the images used as key frames in the model. [file 13007_2015_75_MOESM21_ESM.zip › top_view_1/top_0177.jpg]

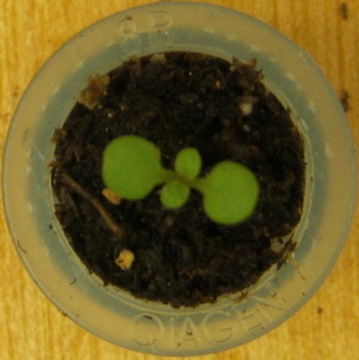

Supplement: Additional file 21 — Col-0 Top View Images for 3-D Model. First half of images of Col-0 captured every 10 min for 5 days from the top view for the 3-D CG model. Table S2 lists the images used as key frames in the model. [file 13007_2015_75_MOESM21_ESM.zip › top_view_1/top_0178.jpg]

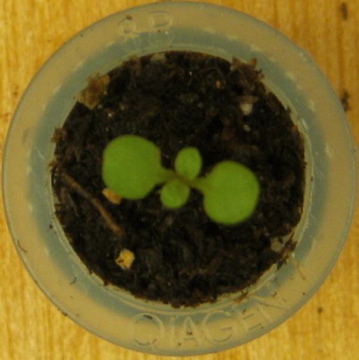

Supplement: Additional file 21 — Col-0 Top View Images for 3-D Model. First half of images of Col-0 captured every 10 min for 5 days from the top view for the 3-D CG model. Table S2 lists the images used as key frames in the model. [file 13007_2015_75_MOESM21_ESM.zip › top_view_1/top_0179.jpg]

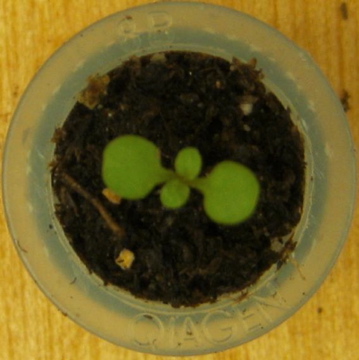

Supplement: Additional file 21 — Col-0 Top View Images for 3-D Model. First half of images of Col-0 captured every 10 min for 5 days from the top view for the 3-D CG model. Table S2 lists the images used as key frames in the model. [file 13007_2015_75_MOESM21_ESM.zip › top_view_1/top_0180.jpg]

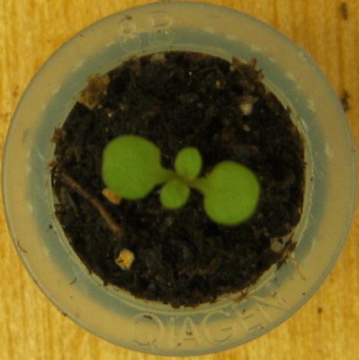

Supplement: Additional file 21 — Col-0 Top View Images for 3-D Model. First half of images of Col-0 captured every 10 min for 5 days from the top view for the 3-D CG model. Table S2 lists the images used as key frames in the model. [file 13007_2015_75_MOESM21_ESM.zip › top_view_1/top_0181.jpg]

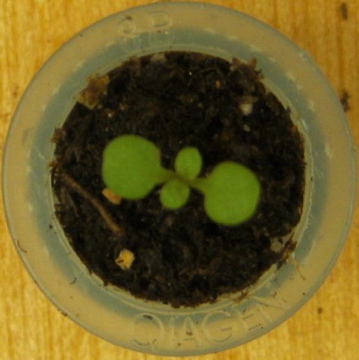

Supplement: Additional file 21 — Col-0 Top View Images for 3-D Model. First half of images of Col-0 captured every 10 min for 5 days from the top view for the 3-D CG model. Table S2 lists the images used as key frames in the model. [file 13007_2015_75_MOESM21_ESM.zip › top_view_1/top_0182.jpg]

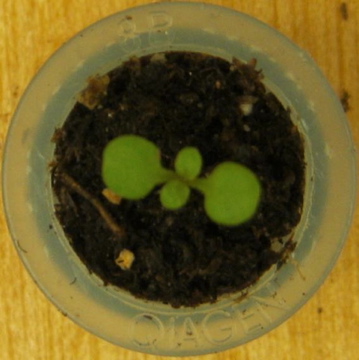

Supplement: Additional file 21 — Col-0 Top View Images for 3-D Model. First half of images of Col-0 captured every 10 min for 5 days from the top view for the 3-D CG model. Table S2 lists the images used as key frames in the model. [file 13007_2015_75_MOESM21_ESM.zip › top_view_1/top_0183.jpg]

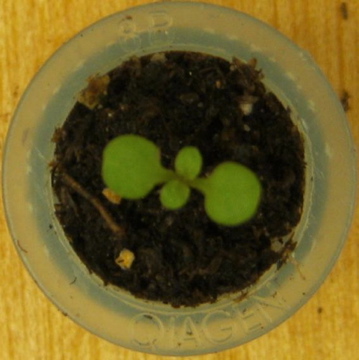

Supplement: Additional file 21 — Col-0 Top View Images for 3-D Model. First half of images of Col-0 captured every 10 min for 5 days from the top view for the 3-D CG model. Table S2 lists the images used as key frames in the model. [file 13007_2015_75_MOESM21_ESM.zip › top_view_1/top_0184.jpg]

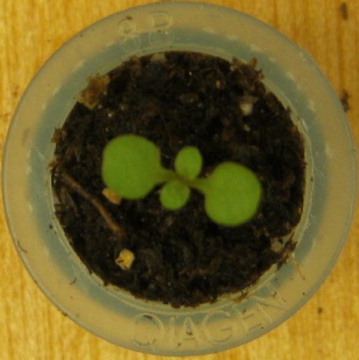

Supplement: Additional file 21 — Col-0 Top View Images for 3-D Model. First half of images of Col-0 captured every 10 min for 5 days from the top view for the 3-D CG model. Table S2 lists the images used as key frames in the model. [file 13007_2015_75_MOESM21_ESM.zip › top_view_1/top_0185.jpg]

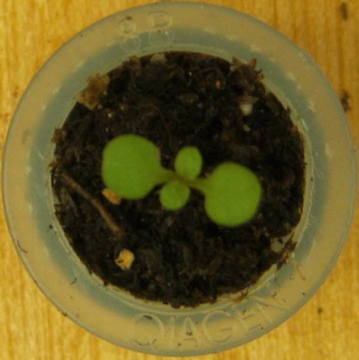

Supplement: Additional file 21 — Col-0 Top View Images for 3-D Model. First half of images of Col-0 captured every 10 min for 5 days from the top view for the 3-D CG model. Table S2 lists the images used as key frames in the model. [file 13007_2015_75_MOESM21_ESM.zip › top_view_1/top_0186.jpg]

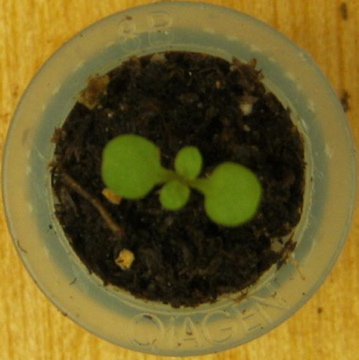

Supplement: Additional file 21 — Col-0 Top View Images for 3-D Model. First half of images of Col-0 captured every 10 min for 5 days from the top view for the 3-D CG model. Table S2 lists the images used as key frames in the model. [file 13007_2015_75_MOESM21_ESM.zip › top_view_1/top_0187.jpg]

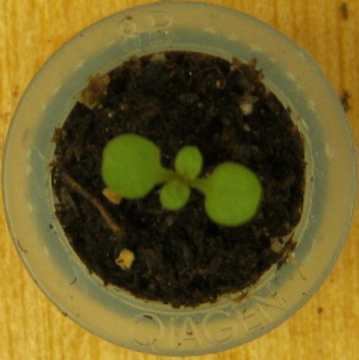

Supplement: Additional file 21 — Col-0 Top View Images for 3-D Model. First half of images of Col-0 captured every 10 min for 5 days from the top view for the 3-D CG model. Table S2 lists the images used as key frames in the model. [file 13007_2015_75_MOESM21_ESM.zip › top_view_1/top_0188.jpg]

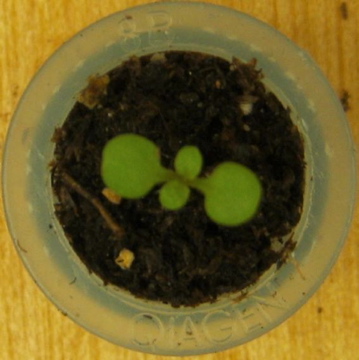

Supplement: Additional file 21 — Col-0 Top View Images for 3-D Model. First half of images of Col-0 captured every 10 min for 5 days from the top view for the 3-D CG model. Table S2 lists the images used as key frames in the model. [file 13007_2015_75_MOESM21_ESM.zip › top_view_1/top_0189.jpg]

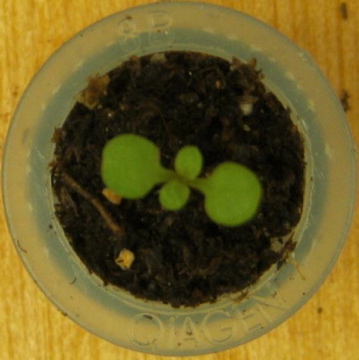

Supplement: Additional file 21 — Col-0 Top View Images for 3-D Model. First half of images of Col-0 captured every 10 min for 5 days from the top view for the 3-D CG model. Table S2 lists the images used as key frames in the model. [file 13007_2015_75_MOESM21_ESM.zip › top_view_1/top_0190.jpg]

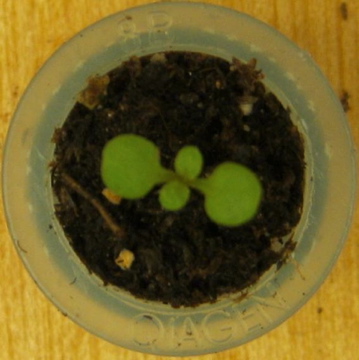

Supplement: Additional file 21 — Col-0 Top View Images for 3-D Model. First half of images of Col-0 captured every 10 min for 5 days from the top view for the 3-D CG model. Table S2 lists the images used as key frames in the model. [file 13007_2015_75_MOESM21_ESM.zip › top_view_1/top_0191.jpg]

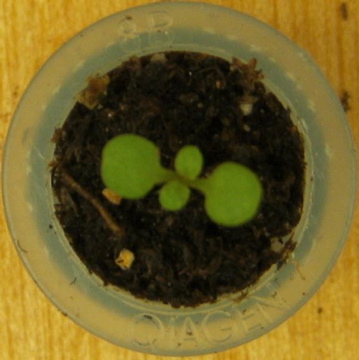

Supplement: Additional file 21 — Col-0 Top View Images for 3-D Model. First half of images of Col-0 captured every 10 min for 5 days from the top view for the 3-D CG model. Table S2 lists the images used as key frames in the model. [file 13007_2015_75_MOESM21_ESM.zip › top_view_1/top_0192.jpg]

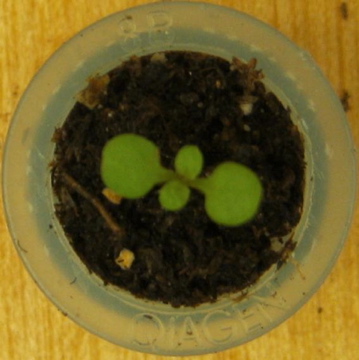

Supplement: Additional file 21 — Col-0 Top View Images for 3-D Model. First half of images of Col-0 captured every 10 min for 5 days from the top view for the 3-D CG model. Table S2 lists the images used as key frames in the model. [file 13007_2015_75_MOESM21_ESM.zip › top_view_1/top_0193.jpg]

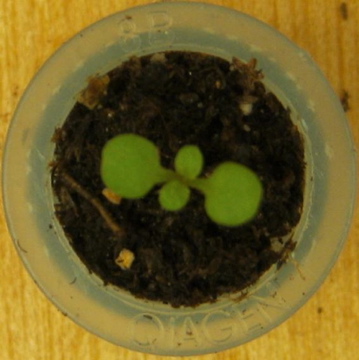

Supplement: Additional file 21 — Col-0 Top View Images for 3-D Model. First half of images of Col-0 captured every 10 min for 5 days from the top view for the 3-D CG model. Table S2 lists the images used as key frames in the model. [file 13007_2015_75_MOESM21_ESM.zip › top_view_1/top_0194.jpg]

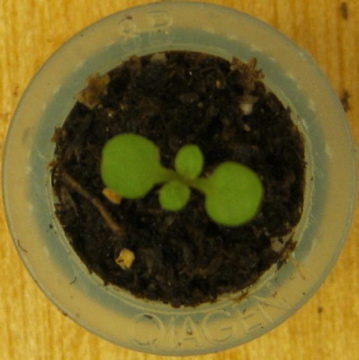

Supplement: Additional file 21 — Col-0 Top View Images for 3-D Model. First half of images of Col-0 captured every 10 min for 5 days from the top view for the 3-D CG model. Table S2 lists the images used as key frames in the model. [file 13007_2015_75_MOESM21_ESM.zip › top_view_1/top_0195.jpg]

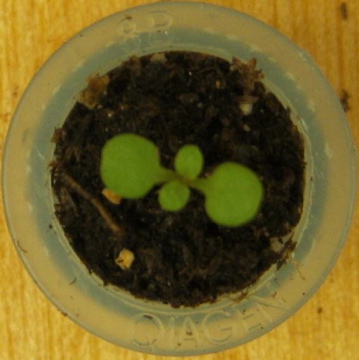

Supplement: Additional file 21 — Col-0 Top View Images for 3-D Model. First half of images of Col-0 captured every 10 min for 5 days from the top view for the 3-D CG model. Table S2 lists the images used as key frames in the model. [file 13007_2015_75_MOESM21_ESM.zip › top_view_1/top_0196.jpg]

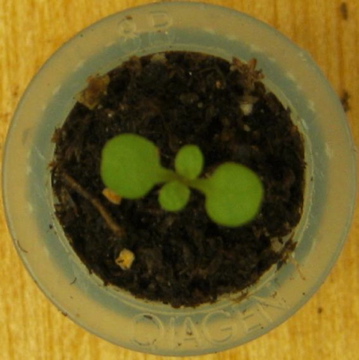

Supplement: Additional file 21 — Col-0 Top View Images for 3-D Model. First half of images of Col-0 captured every 10 min for 5 days from the top view for the 3-D CG model. Table S2 lists the images used as key frames in the model. [file 13007_2015_75_MOESM21_ESM.zip › top_view_1/top_0197.jpg]

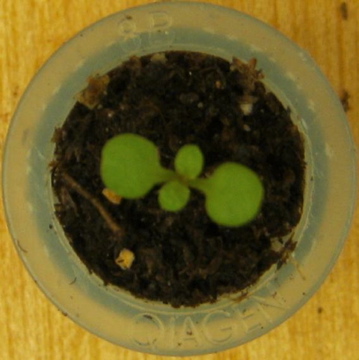

Supplement: Additional file 21 — Col-0 Top View Images for 3-D Model. First half of images of Col-0 captured every 10 min for 5 days from the top view for the 3-D CG model. Table S2 lists the images used as key frames in the model. [file 13007_2015_75_MOESM21_ESM.zip › top_view_1/top_0198.jpg]

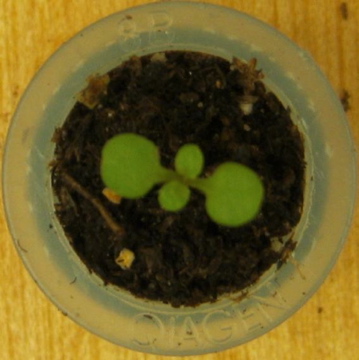

Supplement: Additional file 21 — Col-0 Top View Images for 3-D Model. First half of images of Col-0 captured every 10 min for 5 days from the top view for the 3-D CG model. Table S2 lists the images used as key frames in the model. [file 13007_2015_75_MOESM21_ESM.zip › top_view_1/top_0199.jpg]

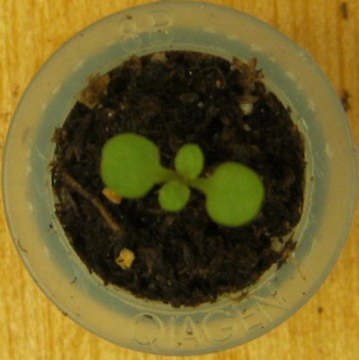

Supplement: Additional file 21 — Col-0 Top View Images for 3-D Model. First half of images of Col-0 captured every 10 min for 5 days from the top view for the 3-D CG model. Table S2 lists the images used as key frames in the model. [file 13007_2015_75_MOESM21_ESM.zip › top_view_1/top_0200.jpg]

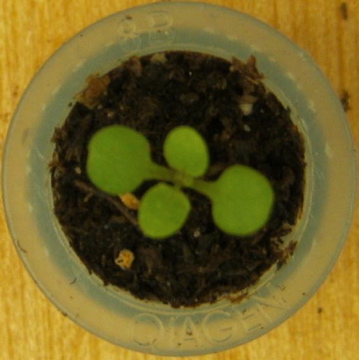

Supplement: Additional file 22 — Col-0 Top View Images for 3-D Model. Second half of images of Col-0 captured every 10 min for 5 days from the top view for the 3-D CG model. Table S2 lists the images used as key frames in the model. [file 13007_2015_75_MOESM22_ESM.zip › top_view_2/top_0346.jpg]

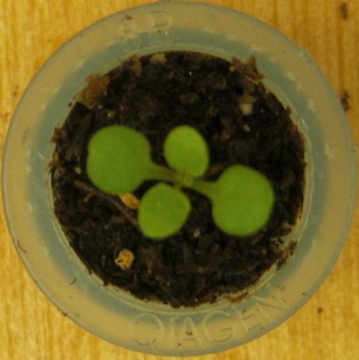

Supplement: Additional file 22 — Col-0 Top View Images for 3-D Model. Second half of images of Col-0 captured every 10 min for 5 days from the top view for the 3-D CG model. Table S2 lists the images used as key frames in the model. [file 13007_2015_75_MOESM22_ESM.zip › top_view_2/top_0347.jpg]

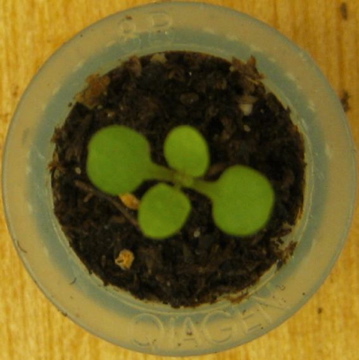

Supplement: Additional file 22 — Col-0 Top View Images for 3-D Model. Second half of images of Col-0 captured every 10 min for 5 days from the top view for the 3-D CG model. Table S2 lists the images used as key frames in the model. [file 13007_2015_75_MOESM22_ESM.zip › top_view_2/top_0348.jpg]

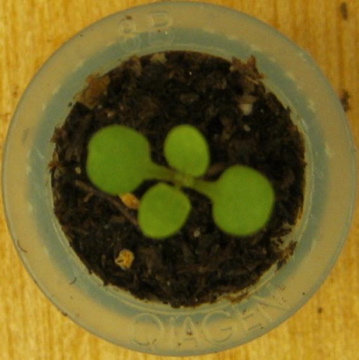

Supplement: Additional file 22 — Col-0 Top View Images for 3-D Model. Second half of images of Col-0 captured every 10 min for 5 days from the top view for the 3-D CG model. Table S2 lists the images used as key frames in the model. [file 13007_2015_75_MOESM22_ESM.zip › top_view_2/top_0349.jpg]

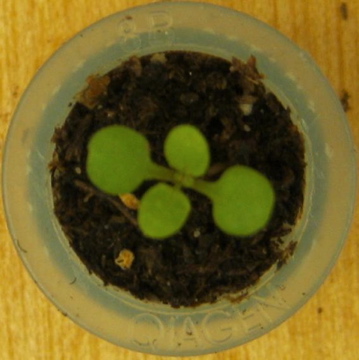

Supplement: Additional file 22 — Col-0 Top View Images for 3-D Model. Second half of images of Col-0 captured every 10 min for 5 days from the top view for the 3-D CG model. Table S2 lists the images used as key frames in the model. [file 13007_2015_75_MOESM22_ESM.zip › top_view_2/top_0350.jpg]

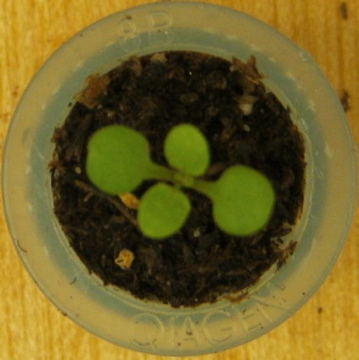

Supplement: Additional file 22 — Col-0 Top View Images for 3-D Model. Second half of images of Col-0 captured every 10 min for 5 days from the top view for the 3-D CG model. Table S2 lists the images used as key frames in the model. [file 13007_2015_75_MOESM22_ESM.zip › top_view_2/top_0351.jpg]

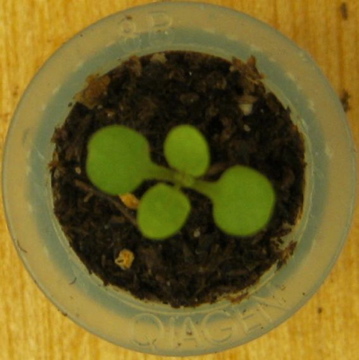

Supplement: Additional file 22 — Col-0 Top View Images for 3-D Model. Second half of images of Col-0 captured every 10 min for 5 days from the top view for the 3-D CG model. Table S2 lists the images used as key frames in the model. [file 13007_2015_75_MOESM22_ESM.zip › top_view_2/top_0352.jpg]

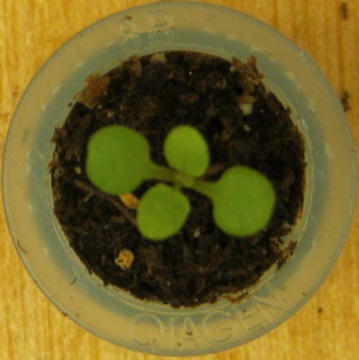

Supplement: Additional file 22 — Col-0 Top View Images for 3-D Model. Second half of images of Col-0 captured every 10 min for 5 days from the top view for the 3-D CG model. Table S2 lists the images used as key frames in the model. [file 13007_2015_75_MOESM22_ESM.zip › top_view_2/top_0353.jpg]
