# Supplementary figures and images for: TRiP: Tracking Rhythms in Plants, an automated leaf movement analysis program for circadian period estimation (part 7 of 10)
Source: Plant Methods. 2015 May 3;11:33. doi: 10.1186/s13007-015-0075-5 (PMC4445800; doi:10.1186/s13007-015-0075-5)

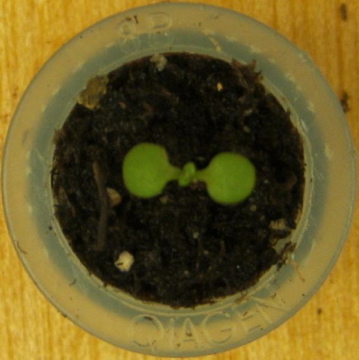

Supplement: Additional file 21 — Col-0 Top View Images for 3-D Model. First half of images of Col-0 captured every 10 min for 5 days from the top view for the 3-D CG model. Table S2 lists the images used as key frames in the model. [file 13007_2015_75_MOESM21_ESM.zip › top_view_1/top_0009.jpg]

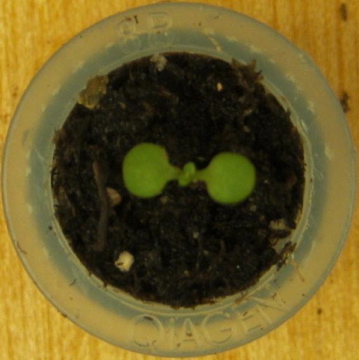

Supplement: Additional file 21 — Col-0 Top View Images for 3-D Model. First half of images of Col-0 captured every 10 min for 5 days from the top view for the 3-D CG model. Table S2 lists the images used as key frames in the model. [file 13007_2015_75_MOESM21_ESM.zip › top_view_1/top_0010.jpg]

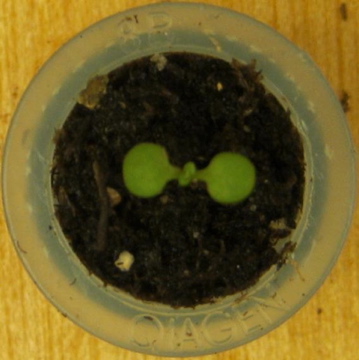

Supplement: Additional file 21 — Col-0 Top View Images for 3-D Model. First half of images of Col-0 captured every 10 min for 5 days from the top view for the 3-D CG model. Table S2 lists the images used as key frames in the model. [file 13007_2015_75_MOESM21_ESM.zip › top_view_1/top_0011.jpg]

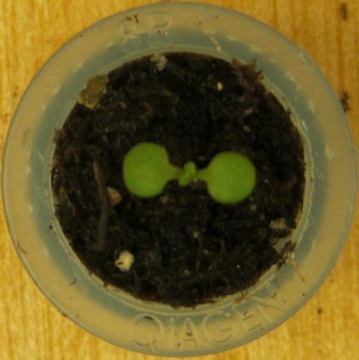

Supplement: Additional file 21 — Col-0 Top View Images for 3-D Model. First half of images of Col-0 captured every 10 min for 5 days from the top view for the 3-D CG model. Table S2 lists the images used as key frames in the model. [file 13007_2015_75_MOESM21_ESM.zip › top_view_1/top_0012.jpg]

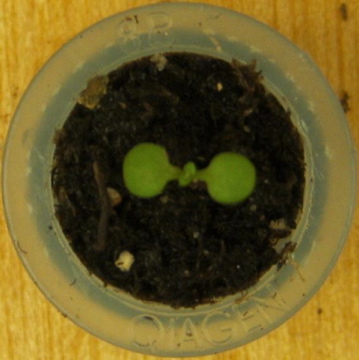

Supplement: Additional file 21 — Col-0 Top View Images for 3-D Model. First half of images of Col-0 captured every 10 min for 5 days from the top view for the 3-D CG model. Table S2 lists the images used as key frames in the model. [file 13007_2015_75_MOESM21_ESM.zip › top_view_1/top_0013.jpg]

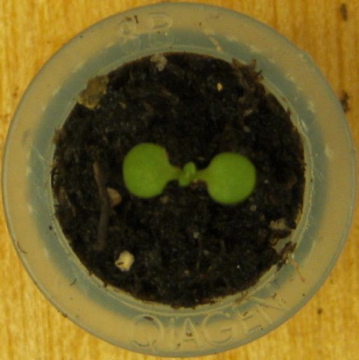

Supplement: Additional file 21 — Col-0 Top View Images for 3-D Model. First half of images of Col-0 captured every 10 min for 5 days from the top view for the 3-D CG model. Table S2 lists the images used as key frames in the model. [file 13007_2015_75_MOESM21_ESM.zip › top_view_1/top_0014.jpg]

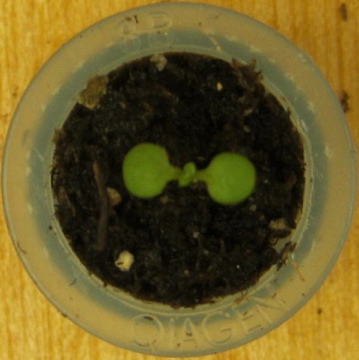

Supplement: Additional file 21 — Col-0 Top View Images for 3-D Model. First half of images of Col-0 captured every 10 min for 5 days from the top view for the 3-D CG model. Table S2 lists the images used as key frames in the model. [file 13007_2015_75_MOESM21_ESM.zip › top_view_1/top_0015.jpg]

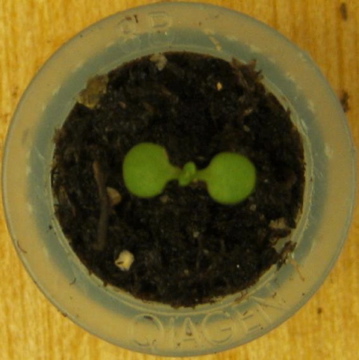

Supplement: Additional file 21 — Col-0 Top View Images for 3-D Model. First half of images of Col-0 captured every 10 min for 5 days from the top view for the 3-D CG model. Table S2 lists the images used as key frames in the model. [file 13007_2015_75_MOESM21_ESM.zip › top_view_1/top_0016.jpg]

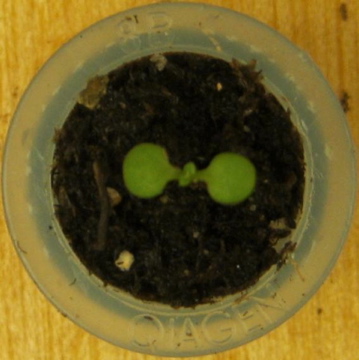

Supplement: Additional file 21 — Col-0 Top View Images for 3-D Model. First half of images of Col-0 captured every 10 min for 5 days from the top view for the 3-D CG model. Table S2 lists the images used as key frames in the model. [file 13007_2015_75_MOESM21_ESM.zip › top_view_1/top_0017.jpg]

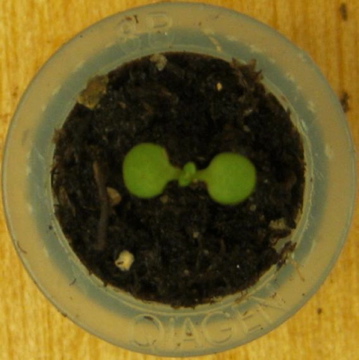

Supplement: Additional file 21 — Col-0 Top View Images for 3-D Model. First half of images of Col-0 captured every 10 min for 5 days from the top view for the 3-D CG model. Table S2 lists the images used as key frames in the model. [file 13007_2015_75_MOESM21_ESM.zip › top_view_1/top_0018.jpg]

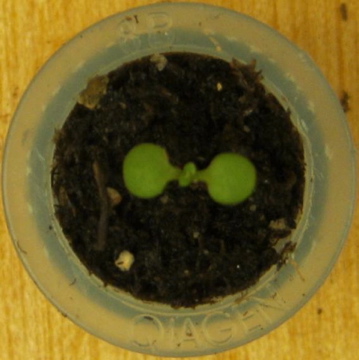

Supplement: Additional file 21 — Col-0 Top View Images for 3-D Model. First half of images of Col-0 captured every 10 min for 5 days from the top view for the 3-D CG model. Table S2 lists the images used as key frames in the model. [file 13007_2015_75_MOESM21_ESM.zip › top_view_1/top_0019.jpg]

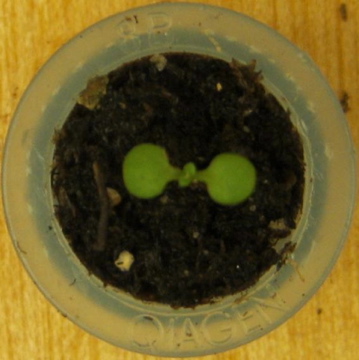

Supplement: Additional file 21 — Col-0 Top View Images for 3-D Model. First half of images of Col-0 captured every 10 min for 5 days from the top view for the 3-D CG model. Table S2 lists the images used as key frames in the model. [file 13007_2015_75_MOESM21_ESM.zip › top_view_1/top_0020.jpg]

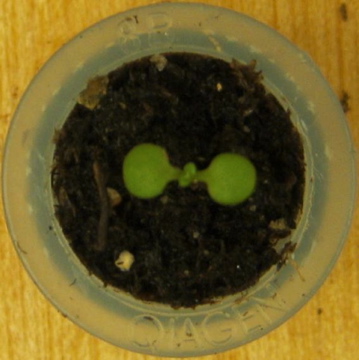

Supplement: Additional file 21 — Col-0 Top View Images for 3-D Model. First half of images of Col-0 captured every 10 min for 5 days from the top view for the 3-D CG model. Table S2 lists the images used as key frames in the model. [file 13007_2015_75_MOESM21_ESM.zip › top_view_1/top_0021.jpg]

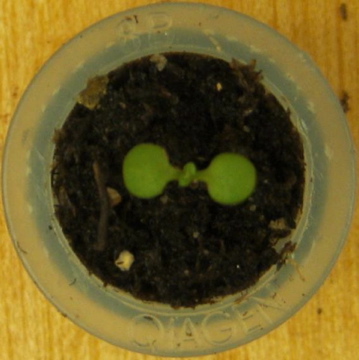

Supplement: Additional file 21 — Col-0 Top View Images for 3-D Model. First half of images of Col-0 captured every 10 min for 5 days from the top view for the 3-D CG model. Table S2 lists the images used as key frames in the model. [file 13007_2015_75_MOESM21_ESM.zip › top_view_1/top_0022.jpg]

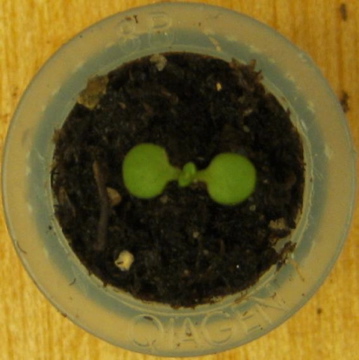

Supplement: Additional file 21 — Col-0 Top View Images for 3-D Model. First half of images of Col-0 captured every 10 min for 5 days from the top view for the 3-D CG model. Table S2 lists the images used as key frames in the model. [file 13007_2015_75_MOESM21_ESM.zip › top_view_1/top_0023.jpg]

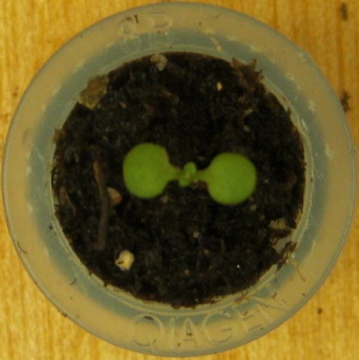

Supplement: Additional file 21 — Col-0 Top View Images for 3-D Model. First half of images of Col-0 captured every 10 min for 5 days from the top view for the 3-D CG model. Table S2 lists the images used as key frames in the model. [file 13007_2015_75_MOESM21_ESM.zip › top_view_1/top_0024.jpg]

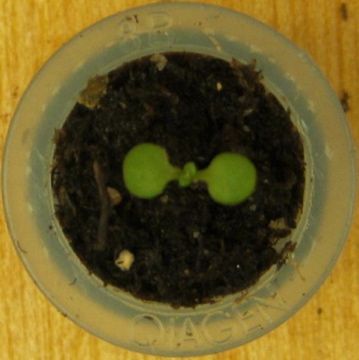

Supplement: Additional file 21 — Col-0 Top View Images for 3-D Model. First half of images of Col-0 captured every 10 min for 5 days from the top view for the 3-D CG model. Table S2 lists the images used as key frames in the model. [file 13007_2015_75_MOESM21_ESM.zip › top_view_1/top_0025.jpg]

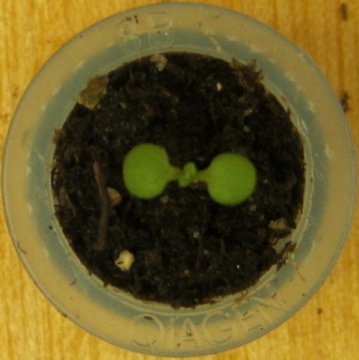

Supplement: Additional file 21 — Col-0 Top View Images for 3-D Model. First half of images of Col-0 captured every 10 min for 5 days from the top view for the 3-D CG model. Table S2 lists the images used as key frames in the model. [file 13007_2015_75_MOESM21_ESM.zip › top_view_1/top_0026.jpg]

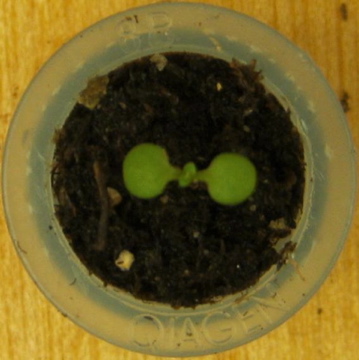

Supplement: Additional file 21 — Col-0 Top View Images for 3-D Model. First half of images of Col-0 captured every 10 min for 5 days from the top view for the 3-D CG model. Table S2 lists the images used as key frames in the model. [file 13007_2015_75_MOESM21_ESM.zip › top_view_1/top_0027.jpg]

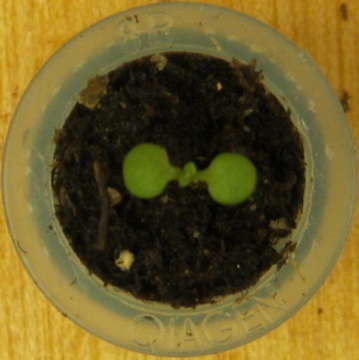

Supplement: Additional file 21 — Col-0 Top View Images for 3-D Model. First half of images of Col-0 captured every 10 min for 5 days from the top view for the 3-D CG model. Table S2 lists the images used as key frames in the model. [file 13007_2015_75_MOESM21_ESM.zip › top_view_1/top_0028.jpg]

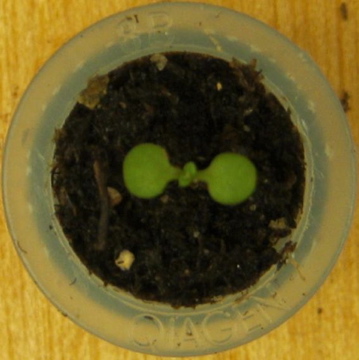

Supplement: Additional file 21 — Col-0 Top View Images for 3-D Model. First half of images of Col-0 captured every 10 min for 5 days from the top view for the 3-D CG model. Table S2 lists the images used as key frames in the model. [file 13007_2015_75_MOESM21_ESM.zip › top_view_1/top_0029.jpg]

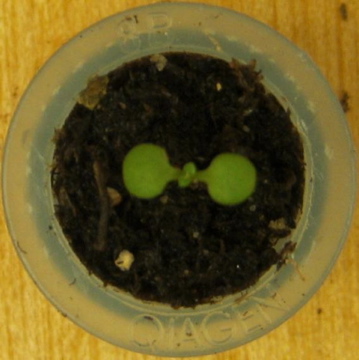

Supplement: Additional file 21 — Col-0 Top View Images for 3-D Model. First half of images of Col-0 captured every 10 min for 5 days from the top view for the 3-D CG model. Table S2 lists the images used as key frames in the model. [file 13007_2015_75_MOESM21_ESM.zip › top_view_1/top_0030.jpg]

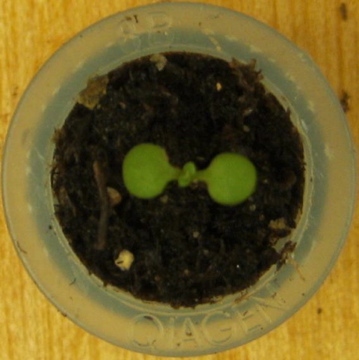

Supplement: Additional file 21 — Col-0 Top View Images for 3-D Model. First half of images of Col-0 captured every 10 min for 5 days from the top view for the 3-D CG model. Table S2 lists the images used as key frames in the model. [file 13007_2015_75_MOESM21_ESM.zip › top_view_1/top_0031.jpg]

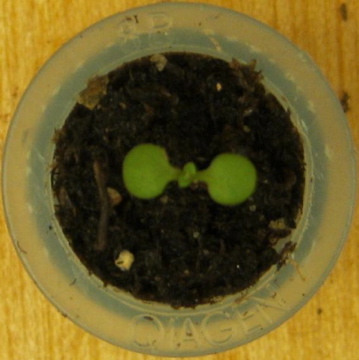

Supplement: Additional file 21 — Col-0 Top View Images for 3-D Model. First half of images of Col-0 captured every 10 min for 5 days from the top view for the 3-D CG model. Table S2 lists the images used as key frames in the model. [file 13007_2015_75_MOESM21_ESM.zip › top_view_1/top_0032.jpg]

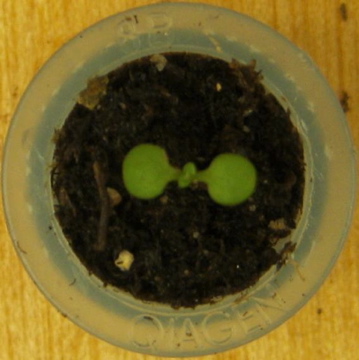

Supplement: Additional file 21 — Col-0 Top View Images for 3-D Model. First half of images of Col-0 captured every 10 min for 5 days from the top view for the 3-D CG model. Table S2 lists the images used as key frames in the model. [file 13007_2015_75_MOESM21_ESM.zip › top_view_1/top_0033.jpg]

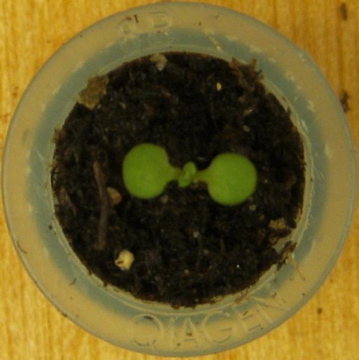

Supplement: Additional file 21 — Col-0 Top View Images for 3-D Model. First half of images of Col-0 captured every 10 min for 5 days from the top view for the 3-D CG model. Table S2 lists the images used as key frames in the model. [file 13007_2015_75_MOESM21_ESM.zip › top_view_1/top_0034.jpg]

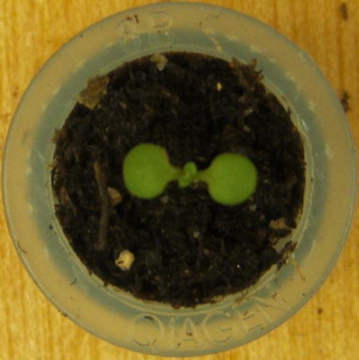

Supplement: Additional file 21 — Col-0 Top View Images for 3-D Model. First half of images of Col-0 captured every 10 min for 5 days from the top view for the 3-D CG model. Table S2 lists the images used as key frames in the model. [file 13007_2015_75_MOESM21_ESM.zip › top_view_1/top_0035.jpg]

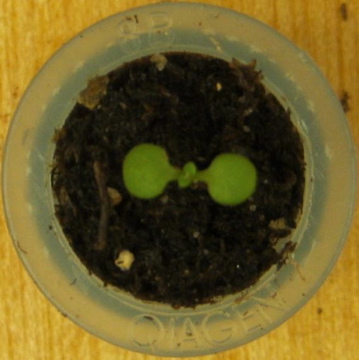

Supplement: Additional file 21 — Col-0 Top View Images for 3-D Model. First half of images of Col-0 captured every 10 min for 5 days from the top view for the 3-D CG model. Table S2 lists the images used as key frames in the model. [file 13007_2015_75_MOESM21_ESM.zip › top_view_1/top_0036.jpg]

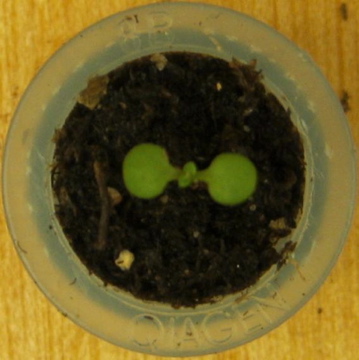

Supplement: Additional file 21 — Col-0 Top View Images for 3-D Model. First half of images of Col-0 captured every 10 min for 5 days from the top view for the 3-D CG model. Table S2 lists the images used as key frames in the model. [file 13007_2015_75_MOESM21_ESM.zip › top_view_1/top_0037.jpg]

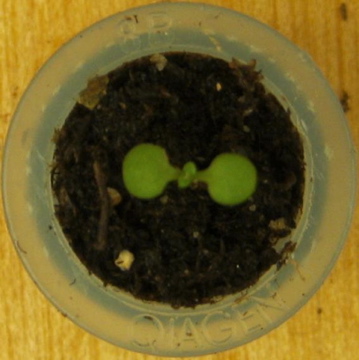

Supplement: Additional file 21 — Col-0 Top View Images for 3-D Model. First half of images of Col-0 captured every 10 min for 5 days from the top view for the 3-D CG model. Table S2 lists the images used as key frames in the model. [file 13007_2015_75_MOESM21_ESM.zip › top_view_1/top_0038.jpg]

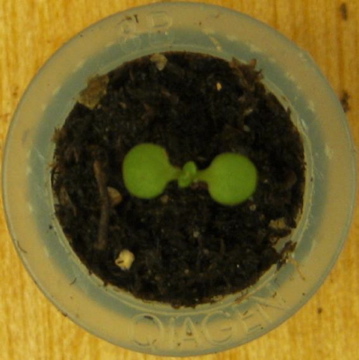

Supplement: Additional file 21 — Col-0 Top View Images for 3-D Model. First half of images of Col-0 captured every 10 min for 5 days from the top view for the 3-D CG model. Table S2 lists the images used as key frames in the model. [file 13007_2015_75_MOESM21_ESM.zip › top_view_1/top_0039.jpg]

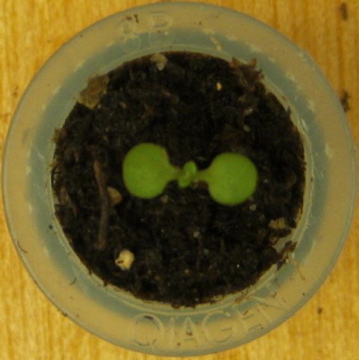

Supplement: Additional file 21 — Col-0 Top View Images for 3-D Model. First half of images of Col-0 captured every 10 min for 5 days from the top view for the 3-D CG model. Table S2 lists the images used as key frames in the model. [file 13007_2015_75_MOESM21_ESM.zip › top_view_1/top_0040.jpg]

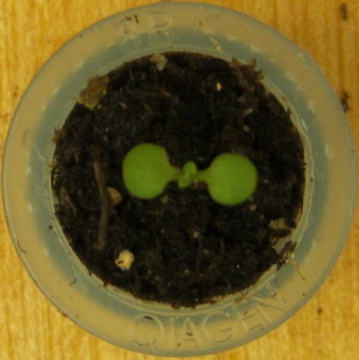

Supplement: Additional file 21 — Col-0 Top View Images for 3-D Model. First half of images of Col-0 captured every 10 min for 5 days from the top view for the 3-D CG model. Table S2 lists the images used as key frames in the model. [file 13007_2015_75_MOESM21_ESM.zip › top_view_1/top_0041.jpg]

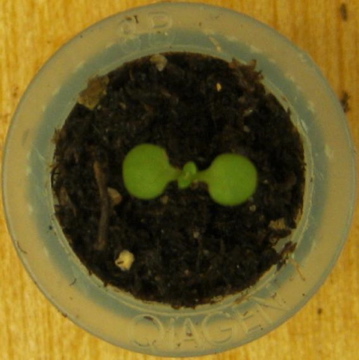

Supplement: Additional file 21 — Col-0 Top View Images for 3-D Model. First half of images of Col-0 captured every 10 min for 5 days from the top view for the 3-D CG model. Table S2 lists the images used as key frames in the model. [file 13007_2015_75_MOESM21_ESM.zip › top_view_1/top_0042.jpg]

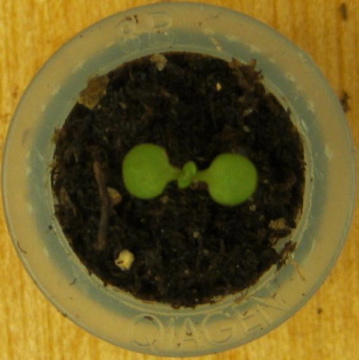

Supplement: Additional file 21 — Col-0 Top View Images for 3-D Model. First half of images of Col-0 captured every 10 min for 5 days from the top view for the 3-D CG model. Table S2 lists the images used as key frames in the model. [file 13007_2015_75_MOESM21_ESM.zip › top_view_1/top_0043.jpg]

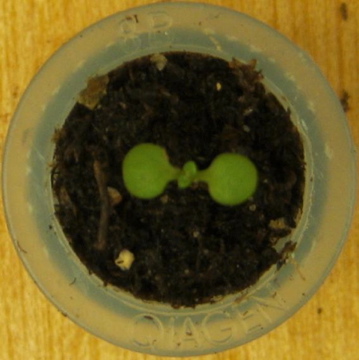

Supplement: Additional file 21 — Col-0 Top View Images for 3-D Model. First half of images of Col-0 captured every 10 min for 5 days from the top view for the 3-D CG model. Table S2 lists the images used as key frames in the model. [file 13007_2015_75_MOESM21_ESM.zip › top_view_1/top_0044.jpg]

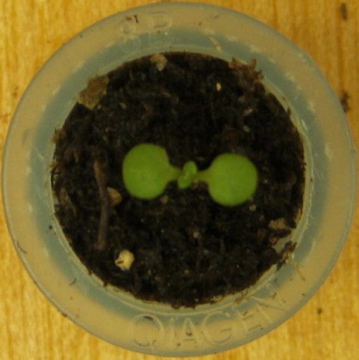

Supplement: Additional file 21 — Col-0 Top View Images for 3-D Model. First half of images of Col-0 captured every 10 min for 5 days from the top view for the 3-D CG model. Table S2 lists the images used as key frames in the model. [file 13007_2015_75_MOESM21_ESM.zip › top_view_1/top_0045.jpg]

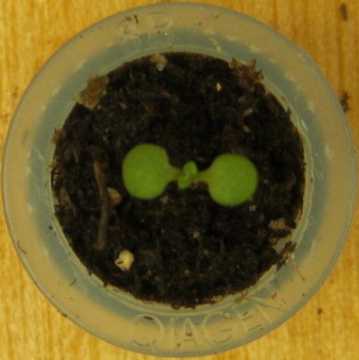

Supplement: Additional file 21 — Col-0 Top View Images for 3-D Model. First half of images of Col-0 captured every 10 min for 5 days from the top view for the 3-D CG model. Table S2 lists the images used as key frames in the model. [file 13007_2015_75_MOESM21_ESM.zip › top_view_1/top_0046.jpg]

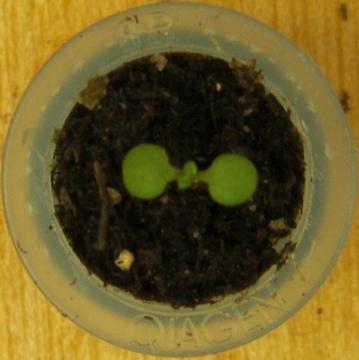

Supplement: Additional file 21 — Col-0 Top View Images for 3-D Model. First half of images of Col-0 captured every 10 min for 5 days from the top view for the 3-D CG model. Table S2 lists the images used as key frames in the model. [file 13007_2015_75_MOESM21_ESM.zip › top_view_1/top_0047.jpg]

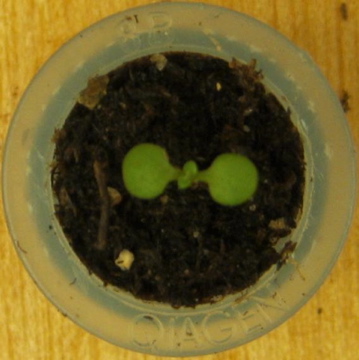

Supplement: Additional file 21 — Col-0 Top View Images for 3-D Model. First half of images of Col-0 captured every 10 min for 5 days from the top view for the 3-D CG model. Table S2 lists the images used as key frames in the model. [file 13007_2015_75_MOESM21_ESM.zip › top_view_1/top_0048.jpg]

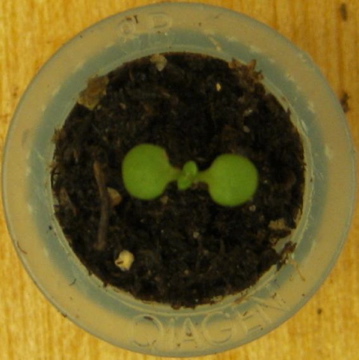

Supplement: Additional file 21 — Col-0 Top View Images for 3-D Model. First half of images of Col-0 captured every 10 min for 5 days from the top view for the 3-D CG model. Table S2 lists the images used as key frames in the model. [file 13007_2015_75_MOESM21_ESM.zip › top_view_1/top_0049.jpg]

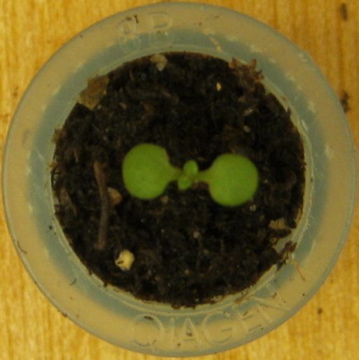

Supplement: Additional file 21 — Col-0 Top View Images for 3-D Model. First half of images of Col-0 captured every 10 min for 5 days from the top view for the 3-D CG model. Table S2 lists the images used as key frames in the model. [file 13007_2015_75_MOESM21_ESM.zip › top_view_1/top_0050.jpg]

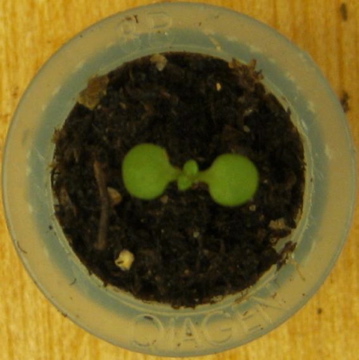

Supplement: Additional file 21 — Col-0 Top View Images for 3-D Model. First half of images of Col-0 captured every 10 min for 5 days from the top view for the 3-D CG model. Table S2 lists the images used as key frames in the model. [file 13007_2015_75_MOESM21_ESM.zip › top_view_1/top_0051.jpg]

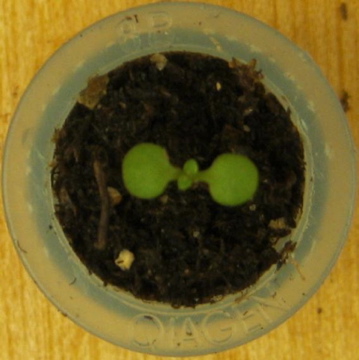

Supplement: Additional file 21 — Col-0 Top View Images for 3-D Model. First half of images of Col-0 captured every 10 min for 5 days from the top view for the 3-D CG model. Table S2 lists the images used as key frames in the model. [file 13007_2015_75_MOESM21_ESM.zip › top_view_1/top_0052.jpg]

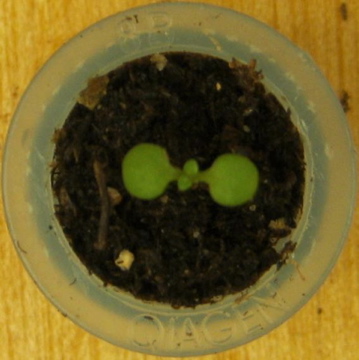

Supplement: Additional file 21 — Col-0 Top View Images for 3-D Model. First half of images of Col-0 captured every 10 min for 5 days from the top view for the 3-D CG model. Table S2 lists the images used as key frames in the model. [file 13007_2015_75_MOESM21_ESM.zip › top_view_1/top_0053.jpg]

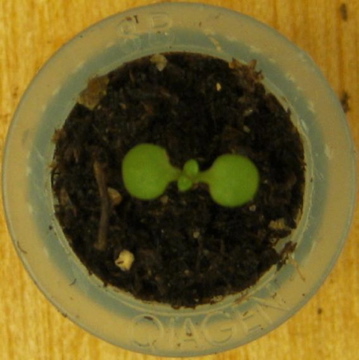

Supplement: Additional file 21 — Col-0 Top View Images for 3-D Model. First half of images of Col-0 captured every 10 min for 5 days from the top view for the 3-D CG model. Table S2 lists the images used as key frames in the model. [file 13007_2015_75_MOESM21_ESM.zip › top_view_1/top_0054.jpg]

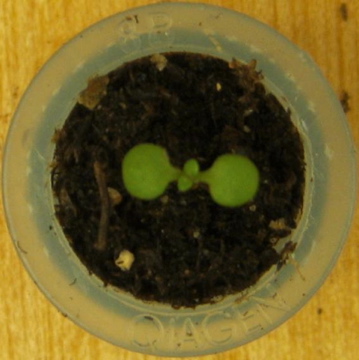

Supplement: Additional file 21 — Col-0 Top View Images for 3-D Model. First half of images of Col-0 captured every 10 min for 5 days from the top view for the 3-D CG model. Table S2 lists the images used as key frames in the model. [file 13007_2015_75_MOESM21_ESM.zip › top_view_1/top_0055.jpg]

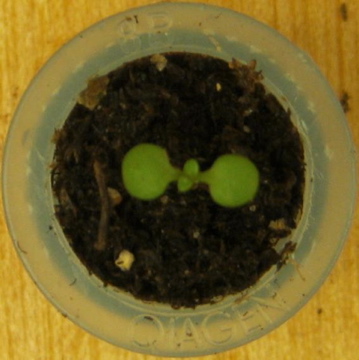

Supplement: Additional file 21 — Col-0 Top View Images for 3-D Model. First half of images of Col-0 captured every 10 min for 5 days from the top view for the 3-D CG model. Table S2 lists the images used as key frames in the model. [file 13007_2015_75_MOESM21_ESM.zip › top_view_1/top_0056.jpg]

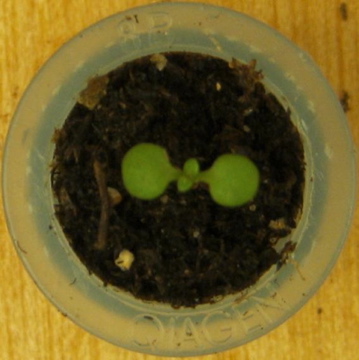

Supplement: Additional file 21 — Col-0 Top View Images for 3-D Model. First half of images of Col-0 captured every 10 min for 5 days from the top view for the 3-D CG model. Table S2 lists the images used as key frames in the model. [file 13007_2015_75_MOESM21_ESM.zip › top_view_1/top_0057.jpg]

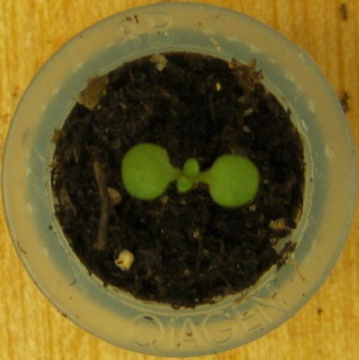

Supplement: Additional file 21 — Col-0 Top View Images for 3-D Model. First half of images of Col-0 captured every 10 min for 5 days from the top view for the 3-D CG model. Table S2 lists the images used as key frames in the model. [file 13007_2015_75_MOESM21_ESM.zip › top_view_1/top_0058.jpg]

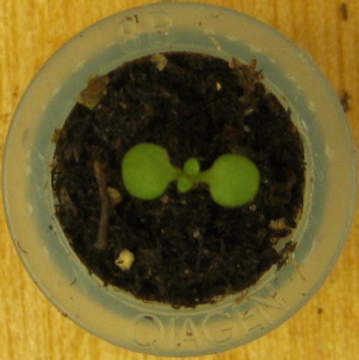

Supplement: Additional file 21 — Col-0 Top View Images for 3-D Model. First half of images of Col-0 captured every 10 min for 5 days from the top view for the 3-D CG model. Table S2 lists the images used as key frames in the model. [file 13007_2015_75_MOESM21_ESM.zip › top_view_1/top_0059.jpg]

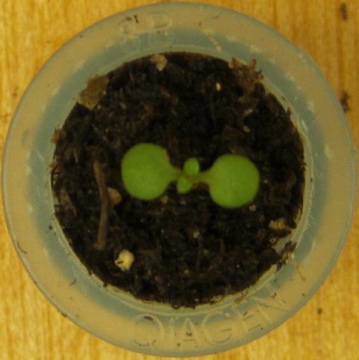

Supplement: Additional file 21 — Col-0 Top View Images for 3-D Model. First half of images of Col-0 captured every 10 min for 5 days from the top view for the 3-D CG model. Table S2 lists the images used as key frames in the model. [file 13007_2015_75_MOESM21_ESM.zip › top_view_1/top_0060.jpg]

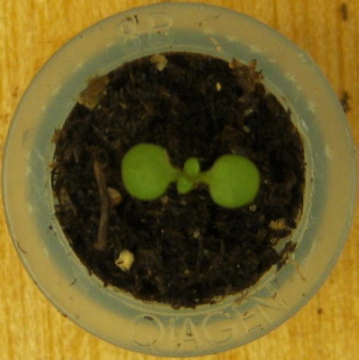

Supplement: Additional file 21 — Col-0 Top View Images for 3-D Model. First half of images of Col-0 captured every 10 min for 5 days from the top view for the 3-D CG model. Table S2 lists the images used as key frames in the model. [file 13007_2015_75_MOESM21_ESM.zip › top_view_1/top_0061.jpg]

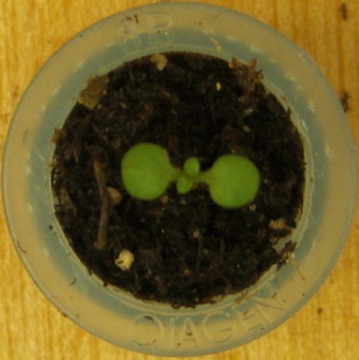

Supplement: Additional file 21 — Col-0 Top View Images for 3-D Model. First half of images of Col-0 captured every 10 min for 5 days from the top view for the 3-D CG model. Table S2 lists the images used as key frames in the model. [file 13007_2015_75_MOESM21_ESM.zip › top_view_1/top_0062.jpg]

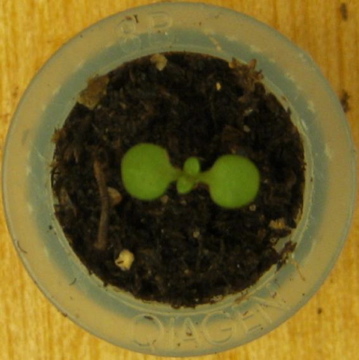

Supplement: Additional file 21 — Col-0 Top View Images for 3-D Model. First half of images of Col-0 captured every 10 min for 5 days from the top view for the 3-D CG model. Table S2 lists the images used as key frames in the model. [file 13007_2015_75_MOESM21_ESM.zip › top_view_1/top_0063.jpg]

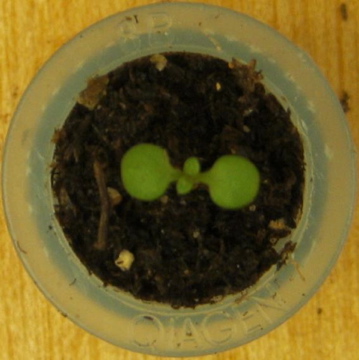

Supplement: Additional file 21 — Col-0 Top View Images for 3-D Model. First half of images of Col-0 captured every 10 min for 5 days from the top view for the 3-D CG model. Table S2 lists the images used as key frames in the model. [file 13007_2015_75_MOESM21_ESM.zip › top_view_1/top_0064.jpg]

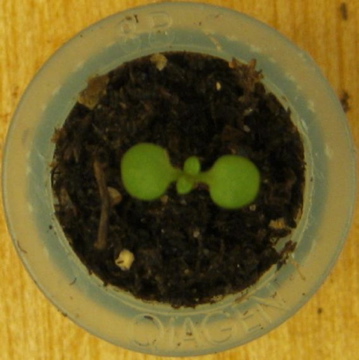

Supplement: Additional file 21 — Col-0 Top View Images for 3-D Model. First half of images of Col-0 captured every 10 min for 5 days from the top view for the 3-D CG model. Table S2 lists the images used as key frames in the model. [file 13007_2015_75_MOESM21_ESM.zip › top_view_1/top_0065.jpg]

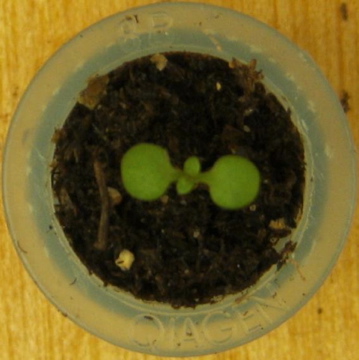

Supplement: Additional file 21 — Col-0 Top View Images for 3-D Model. First half of images of Col-0 captured every 10 min for 5 days from the top view for the 3-D CG model. Table S2 lists the images used as key frames in the model. [file 13007_2015_75_MOESM21_ESM.zip › top_view_1/top_0066.jpg]

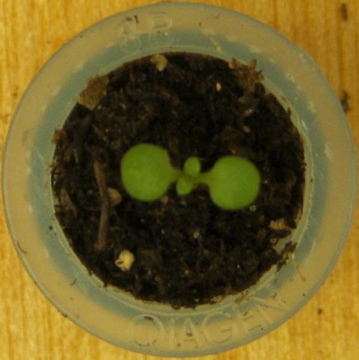

Supplement: Additional file 21 — Col-0 Top View Images for 3-D Model. First half of images of Col-0 captured every 10 min for 5 days from the top view for the 3-D CG model. Table S2 lists the images used as key frames in the model. [file 13007_2015_75_MOESM21_ESM.zip › top_view_1/top_0067.jpg]

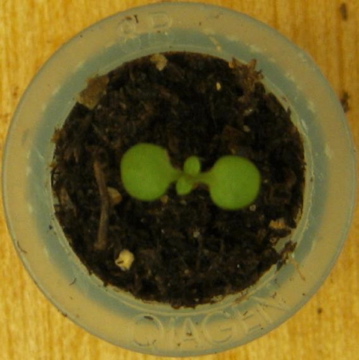

Supplement: Additional file 21 — Col-0 Top View Images for 3-D Model. First half of images of Col-0 captured every 10 min for 5 days from the top view for the 3-D CG model. Table S2 lists the images used as key frames in the model. [file 13007_2015_75_MOESM21_ESM.zip › top_view_1/top_0068.jpg]

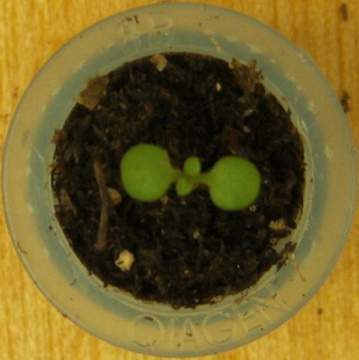

Supplement: Additional file 21 — Col-0 Top View Images for 3-D Model. First half of images of Col-0 captured every 10 min for 5 days from the top view for the 3-D CG model. Table S2 lists the images used as key frames in the model. [file 13007_2015_75_MOESM21_ESM.zip › top_view_1/top_0069.jpg]

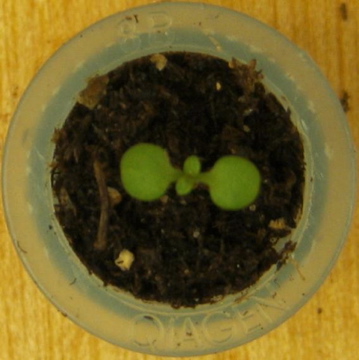

Supplement: Additional file 21 — Col-0 Top View Images for 3-D Model. First half of images of Col-0 captured every 10 min for 5 days from the top view for the 3-D CG model. Table S2 lists the images used as key frames in the model. [file 13007_2015_75_MOESM21_ESM.zip › top_view_1/top_0070.jpg]

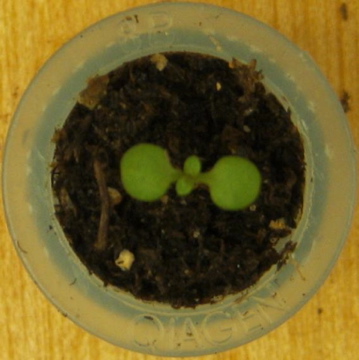

Supplement: Additional file 21 — Col-0 Top View Images for 3-D Model. First half of images of Col-0 captured every 10 min for 5 days from the top view for the 3-D CG model. Table S2 lists the images used as key frames in the model. [file 13007_2015_75_MOESM21_ESM.zip › top_view_1/top_0071.jpg]

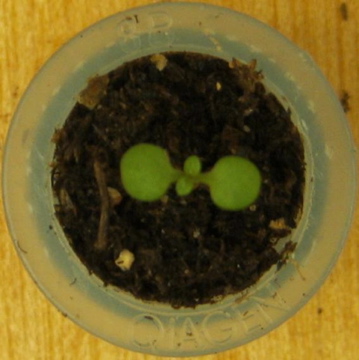

Supplement: Additional file 21 — Col-0 Top View Images for 3-D Model. First half of images of Col-0 captured every 10 min for 5 days from the top view for the 3-D CG model. Table S2 lists the images used as key frames in the model. [file 13007_2015_75_MOESM21_ESM.zip › top_view_1/top_0072.jpg]

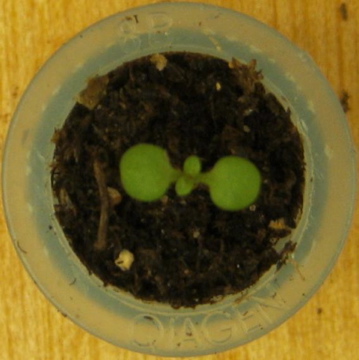

Supplement: Additional file 21 — Col-0 Top View Images for 3-D Model. First half of images of Col-0 captured every 10 min for 5 days from the top view for the 3-D CG model. Table S2 lists the images used as key frames in the model. [file 13007_2015_75_MOESM21_ESM.zip › top_view_1/top_0073.jpg]

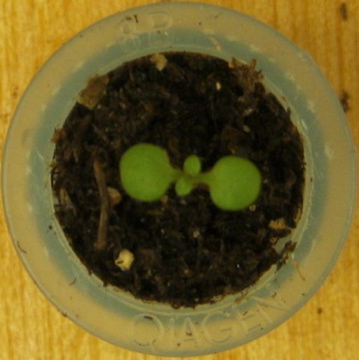

Supplement: Additional file 21 — Col-0 Top View Images for 3-D Model. First half of images of Col-0 captured every 10 min for 5 days from the top view for the 3-D CG model. Table S2 lists the images used as key frames in the model. [file 13007_2015_75_MOESM21_ESM.zip › top_view_1/top_0074.jpg]

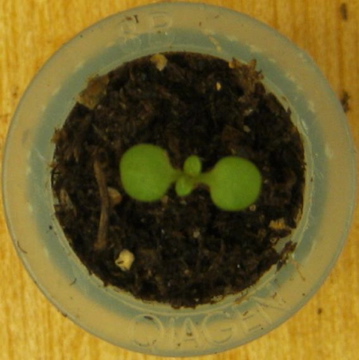

Supplement: Additional file 21 — Col-0 Top View Images for 3-D Model. First half of images of Col-0 captured every 10 min for 5 days from the top view for the 3-D CG model. Table S2 lists the images used as key frames in the model. [file 13007_2015_75_MOESM21_ESM.zip › top_view_1/top_0075.jpg]

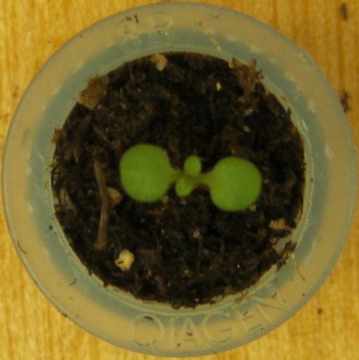

Supplement: Additional file 21 — Col-0 Top View Images for 3-D Model. First half of images of Col-0 captured every 10 min for 5 days from the top view for the 3-D CG model. Table S2 lists the images used as key frames in the model. [file 13007_2015_75_MOESM21_ESM.zip › top_view_1/top_0076.jpg]

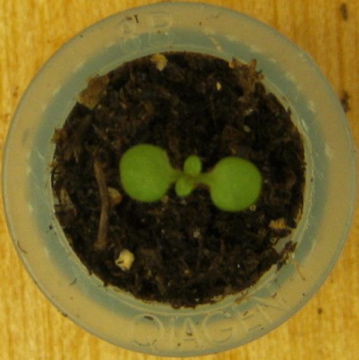

Supplement: Additional file 21 — Col-0 Top View Images for 3-D Model. First half of images of Col-0 captured every 10 min for 5 days from the top view for the 3-D CG model. Table S2 lists the images used as key frames in the model. [file 13007_2015_75_MOESM21_ESM.zip › top_view_1/top_0077.jpg]

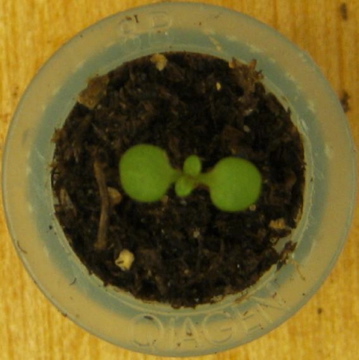

Supplement: Additional file 21 — Col-0 Top View Images for 3-D Model. First half of images of Col-0 captured every 10 min for 5 days from the top view for the 3-D CG model. Table S2 lists the images used as key frames in the model. [file 13007_2015_75_MOESM21_ESM.zip › top_view_1/top_0078.jpg]

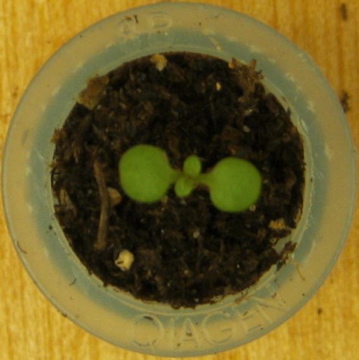

Supplement: Additional file 21 — Col-0 Top View Images for 3-D Model. First half of images of Col-0 captured every 10 min for 5 days from the top view for the 3-D CG model. Table S2 lists the images used as key frames in the model. [file 13007_2015_75_MOESM21_ESM.zip › top_view_1/top_0079.jpg]

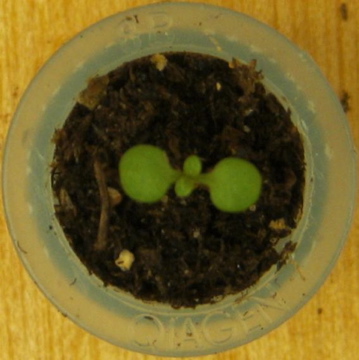

Supplement: Additional file 21 — Col-0 Top View Images for 3-D Model. First half of images of Col-0 captured every 10 min for 5 days from the top view for the 3-D CG model. Table S2 lists the images used as key frames in the model. [file 13007_2015_75_MOESM21_ESM.zip › top_view_1/top_0080.jpg]

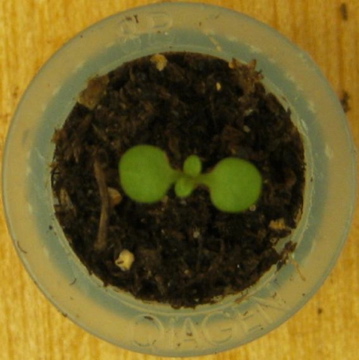

Supplement: Additional file 21 — Col-0 Top View Images for 3-D Model. First half of images of Col-0 captured every 10 min for 5 days from the top view for the 3-D CG model. Table S2 lists the images used as key frames in the model. [file 13007_2015_75_MOESM21_ESM.zip › top_view_1/top_0081.jpg]

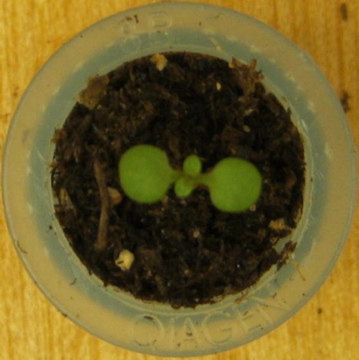

Supplement: Additional file 21 — Col-0 Top View Images for 3-D Model. First half of images of Col-0 captured every 10 min for 5 days from the top view for the 3-D CG model. Table S2 lists the images used as key frames in the model. [file 13007_2015_75_MOESM21_ESM.zip › top_view_1/top_0082.jpg]

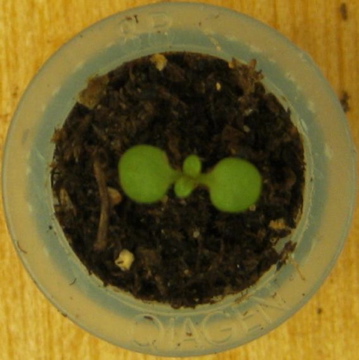

Supplement: Additional file 21 — Col-0 Top View Images for 3-D Model. First half of images of Col-0 captured every 10 min for 5 days from the top view for the 3-D CG model. Table S2 lists the images used as key frames in the model. [file 13007_2015_75_MOESM21_ESM.zip › top_view_1/top_0083.jpg]

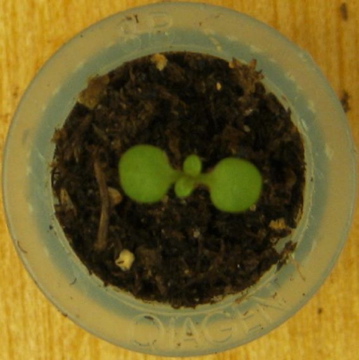

Supplement: Additional file 21 — Col-0 Top View Images for 3-D Model. First half of images of Col-0 captured every 10 min for 5 days from the top view for the 3-D CG model. Table S2 lists the images used as key frames in the model. [file 13007_2015_75_MOESM21_ESM.zip › top_view_1/top_0084.jpg]

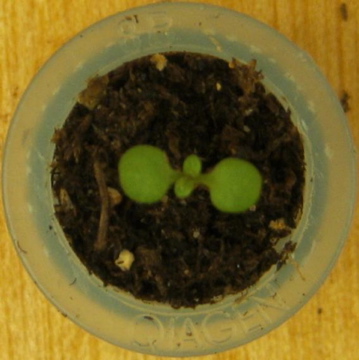

Supplement: Additional file 21 — Col-0 Top View Images for 3-D Model. First half of images of Col-0 captured every 10 min for 5 days from the top view for the 3-D CG model. Table S2 lists the images used as key frames in the model. [file 13007_2015_75_MOESM21_ESM.zip › top_view_1/top_0085.jpg]

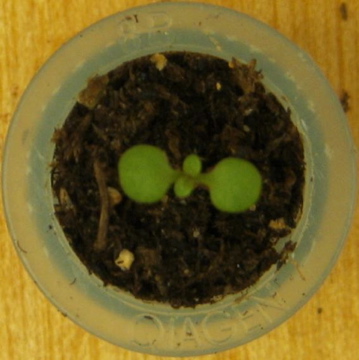

Supplement: Additional file 21 — Col-0 Top View Images for 3-D Model. First half of images of Col-0 captured every 10 min for 5 days from the top view for the 3-D CG model. Table S2 lists the images used as key frames in the model. [file 13007_2015_75_MOESM21_ESM.zip › top_view_1/top_0086.jpg]

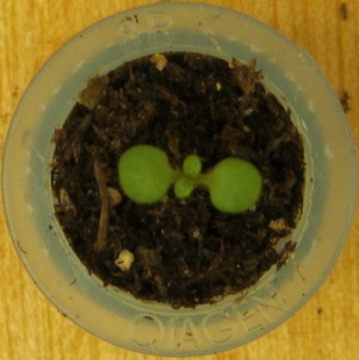

Supplement: Additional file 21 — Col-0 Top View Images for 3-D Model. First half of images of Col-0 captured every 10 min for 5 days from the top view for the 3-D CG model. Table S2 lists the images used as key frames in the model. [file 13007_2015_75_MOESM21_ESM.zip › top_view_1/top_0087.jpg]

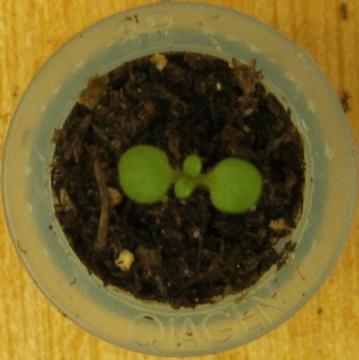

Supplement: Additional file 21 — Col-0 Top View Images for 3-D Model. First half of images of Col-0 captured every 10 min for 5 days from the top view for the 3-D CG model. Table S2 lists the images used as key frames in the model. [file 13007_2015_75_MOESM21_ESM.zip › top_view_1/top_0088.jpg]

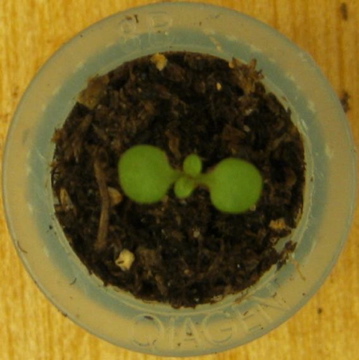

Supplement: Additional file 21 — Col-0 Top View Images for 3-D Model. First half of images of Col-0 captured every 10 min for 5 days from the top view for the 3-D CG model. Table S2 lists the images used as key frames in the model. [file 13007_2015_75_MOESM21_ESM.zip › top_view_1/top_0089.jpg]

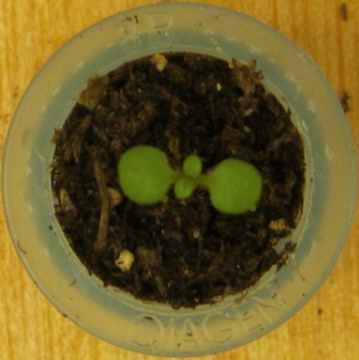

Supplement: Additional file 21 — Col-0 Top View Images for 3-D Model. First half of images of Col-0 captured every 10 min for 5 days from the top view for the 3-D CG model. Table S2 lists the images used as key frames in the model. [file 13007_2015_75_MOESM21_ESM.zip › top_view_1/top_0090.jpg]

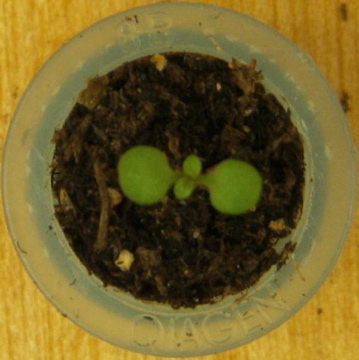

Supplement: Additional file 21 — Col-0 Top View Images for 3-D Model. First half of images of Col-0 captured every 10 min for 5 days from the top view for the 3-D CG model. Table S2 lists the images used as key frames in the model. [file 13007_2015_75_MOESM21_ESM.zip › top_view_1/top_0091.jpg]

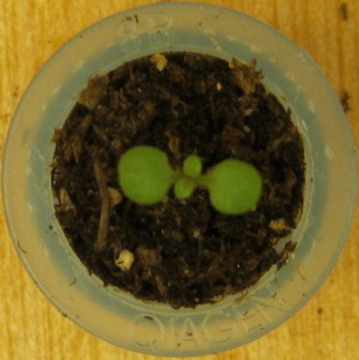

Supplement: Additional file 21 — Col-0 Top View Images for 3-D Model. First half of images of Col-0 captured every 10 min for 5 days from the top view for the 3-D CG model. Table S2 lists the images used as key frames in the model. [file 13007_2015_75_MOESM21_ESM.zip › top_view_1/top_0092.jpg]

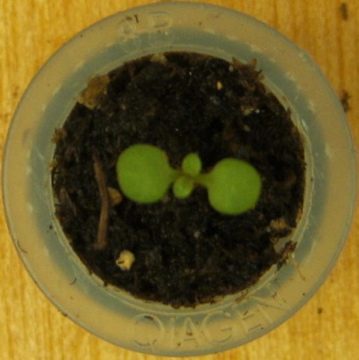

Supplement: Additional file 21 — Col-0 Top View Images for 3-D Model. First half of images of Col-0 captured every 10 min for 5 days from the top view for the 3-D CG model. Table S2 lists the images used as key frames in the model. [file 13007_2015_75_MOESM21_ESM.zip › top_view_1/top_0093.jpg]

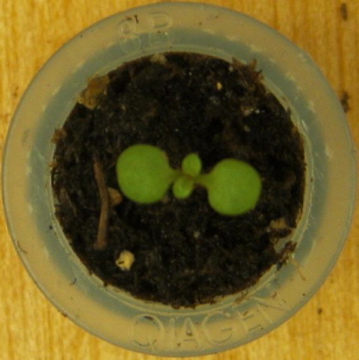

Supplement: Additional file 21 — Col-0 Top View Images for 3-D Model. First half of images of Col-0 captured every 10 min for 5 days from the top view for the 3-D CG model. Table S2 lists the images used as key frames in the model. [file 13007_2015_75_MOESM21_ESM.zip › top_view_1/top_0094.jpg]

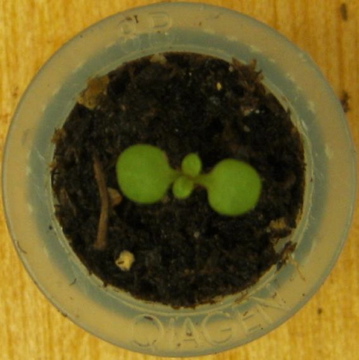

Supplement: Additional file 21 — Col-0 Top View Images for 3-D Model. First half of images of Col-0 captured every 10 min for 5 days from the top view for the 3-D CG model. Table S2 lists the images used as key frames in the model. [file 13007_2015_75_MOESM21_ESM.zip › top_view_1/top_0095.jpg]

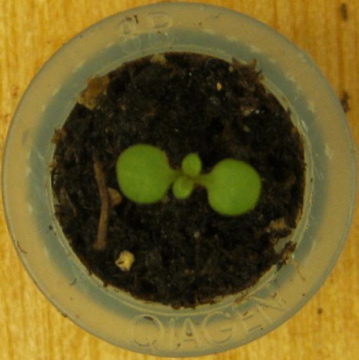

Supplement: Additional file 21 — Col-0 Top View Images for 3-D Model. First half of images of Col-0 captured every 10 min for 5 days from the top view for the 3-D CG model. Table S2 lists the images used as key frames in the model. [file 13007_2015_75_MOESM21_ESM.zip › top_view_1/top_0096.jpg]

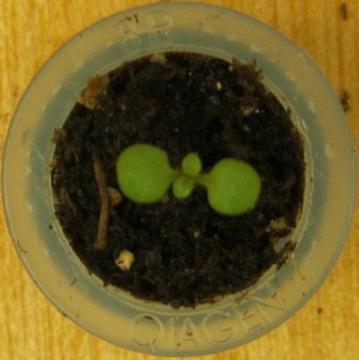

Supplement: Additional file 21 — Col-0 Top View Images for 3-D Model. First half of images of Col-0 captured every 10 min for 5 days from the top view for the 3-D CG model. Table S2 lists the images used as key frames in the model. [file 13007_2015_75_MOESM21_ESM.zip › top_view_1/top_0097.jpg]

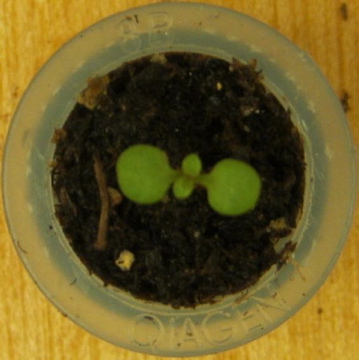

Supplement: Additional file 21 — Col-0 Top View Images for 3-D Model. First half of images of Col-0 captured every 10 min for 5 days from the top view for the 3-D CG model. Table S2 lists the images used as key frames in the model. [file 13007_2015_75_MOESM21_ESM.zip › top_view_1/top_0098.jpg]

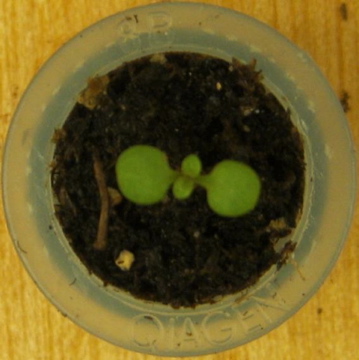

Supplement: Additional file 21 — Col-0 Top View Images for 3-D Model. First half of images of Col-0 captured every 10 min for 5 days from the top view for the 3-D CG model. Table S2 lists the images used as key frames in the model. [file 13007_2015_75_MOESM21_ESM.zip › top_view_1/top_0099.jpg]

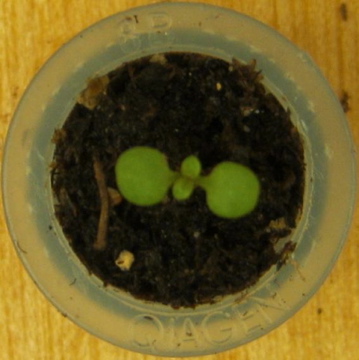

Supplement: Additional file 21 — Col-0 Top View Images for 3-D Model. First half of images of Col-0 captured every 10 min for 5 days from the top view for the 3-D CG model. Table S2 lists the images used as key frames in the model. [file 13007_2015_75_MOESM21_ESM.zip › top_view_1/top_0100.jpg]

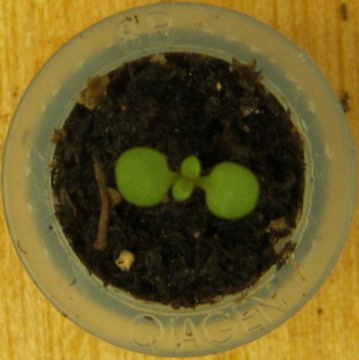

Supplement: Additional file 21 — Col-0 Top View Images for 3-D Model. First half of images of Col-0 captured every 10 min for 5 days from the top view for the 3-D CG model. Table S2 lists the images used as key frames in the model. [file 13007_2015_75_MOESM21_ESM.zip › top_view_1/top_0101.jpg]

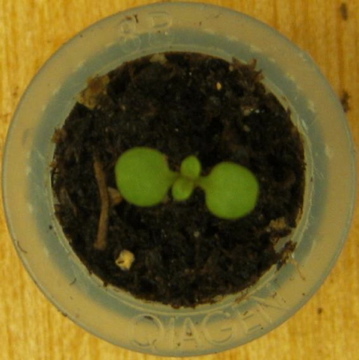

Supplement: Additional file 21 — Col-0 Top View Images for 3-D Model. First half of images of Col-0 captured every 10 min for 5 days from the top view for the 3-D CG model. Table S2 lists the images used as key frames in the model. [file 13007_2015_75_MOESM21_ESM.zip › top_view_1/top_0102.jpg]

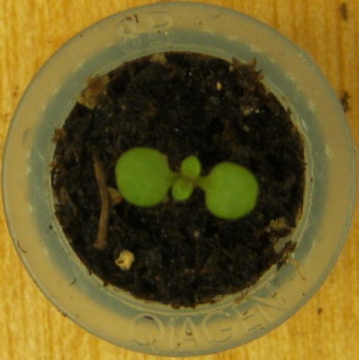

Supplement: Additional file 21 — Col-0 Top View Images for 3-D Model. First half of images of Col-0 captured every 10 min for 5 days from the top view for the 3-D CG model. Table S2 lists the images used as key frames in the model. [file 13007_2015_75_MOESM21_ESM.zip › top_view_1/top_0103.jpg]

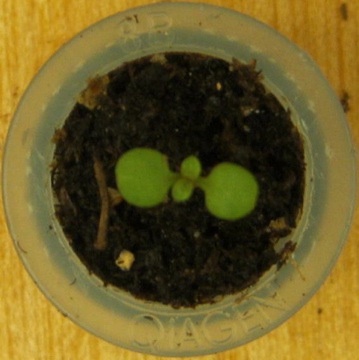

Supplement: Additional file 21 — Col-0 Top View Images for 3-D Model. First half of images of Col-0 captured every 10 min for 5 days from the top view for the 3-D CG model. Table S2 lists the images used as key frames in the model. [file 13007_2015_75_MOESM21_ESM.zip › top_view_1/top_0104.jpg]

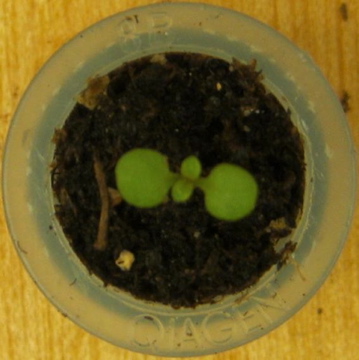

Supplement: Additional file 21 — Col-0 Top View Images for 3-D Model. First half of images of Col-0 captured every 10 min for 5 days from the top view for the 3-D CG model. Table S2 lists the images used as key frames in the model. [file 13007_2015_75_MOESM21_ESM.zip › top_view_1/top_0105.jpg]

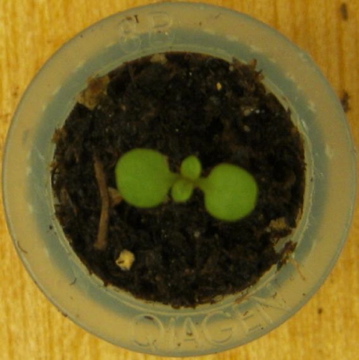

Supplement: Additional file 21 — Col-0 Top View Images for 3-D Model. First half of images of Col-0 captured every 10 min for 5 days from the top view for the 3-D CG model. Table S2 lists the images used as key frames in the model. [file 13007_2015_75_MOESM21_ESM.zip › top_view_1/top_0106.jpg]

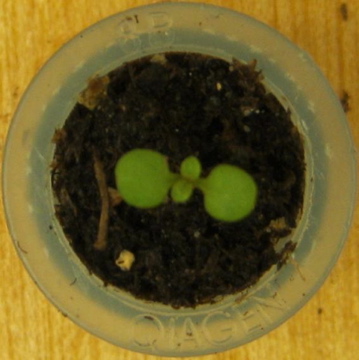

Supplement: Additional file 21 — Col-0 Top View Images for 3-D Model. First half of images of Col-0 captured every 10 min for 5 days from the top view for the 3-D CG model. Table S2 lists the images used as key frames in the model. [file 13007_2015_75_MOESM21_ESM.zip › top_view_1/top_0107.jpg]

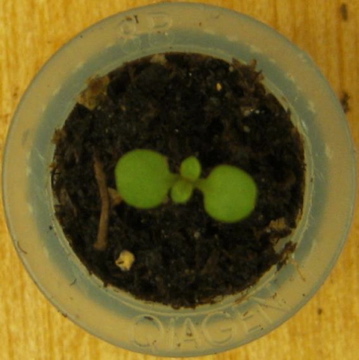

Supplement: Additional file 21 — Col-0 Top View Images for 3-D Model. First half of images of Col-0 captured every 10 min for 5 days from the top view for the 3-D CG model. Table S2 lists the images used as key frames in the model. [file 13007_2015_75_MOESM21_ESM.zip › top_view_1/top_0108.jpg]
