# Supplementary figures and images for: TRiP: Tracking Rhythms in Plants, an automated leaf movement analysis program for circadian period estimation (part 10 of 10)
Source: Plant Methods. 2015 May 3;11:33. doi: 10.1186/s13007-015-0075-5 (PMC4445800; doi:10.1186/s13007-015-0075-5)

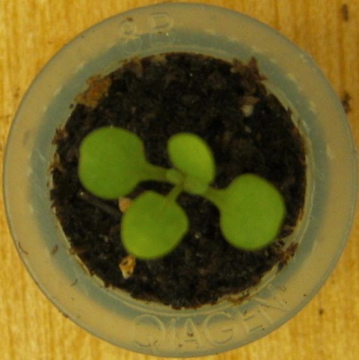

Supplement: Additional file 22 — Col-0 Top View Images for 3-D Model. Second half of images of Col-0 captured every 10 min for 5 days from the top view for the 3-D CG model. Table S2 lists the images used as key frames in the model. [file 13007_2015_75_MOESM22_ESM.zip › top_view_2/top_0454.jpg]

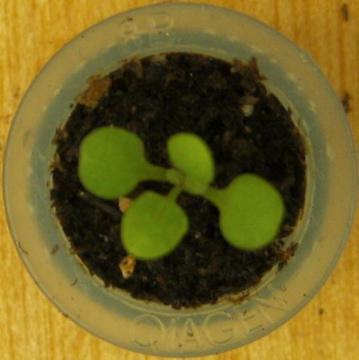

Supplement: Additional file 22 — Col-0 Top View Images for 3-D Model. Second half of images of Col-0 captured every 10 min for 5 days from the top view for the 3-D CG model. Table S2 lists the images used as key frames in the model. [file 13007_2015_75_MOESM22_ESM.zip › top_view_2/top_0455.jpg]

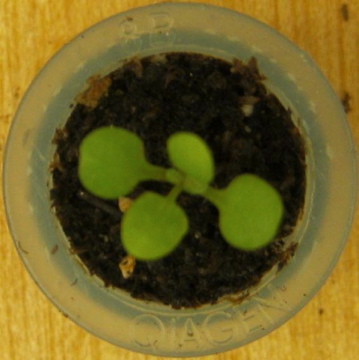

Supplement: Additional file 22 — Col-0 Top View Images for 3-D Model. Second half of images of Col-0 captured every 10 min for 5 days from the top view for the 3-D CG model. Table S2 lists the images used as key frames in the model. [file 13007_2015_75_MOESM22_ESM.zip › top_view_2/top_0456.jpg]

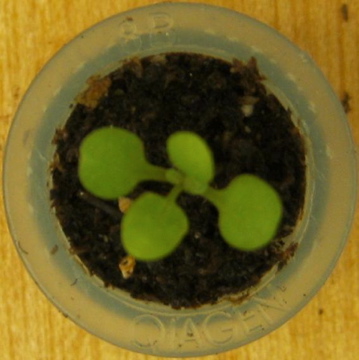

Supplement: Additional file 22 — Col-0 Top View Images for 3-D Model. Second half of images of Col-0 captured every 10 min for 5 days from the top view for the 3-D CG model. Table S2 lists the images used as key frames in the model. [file 13007_2015_75_MOESM22_ESM.zip › top_view_2/top_0457.jpg]

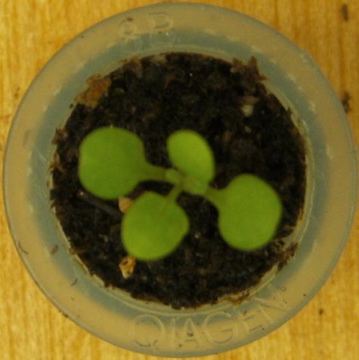

Supplement: Additional file 22 — Col-0 Top View Images for 3-D Model. Second half of images of Col-0 captured every 10 min for 5 days from the top view for the 3-D CG model. Table S2 lists the images used as key frames in the model. [file 13007_2015_75_MOESM22_ESM.zip › top_view_2/top_0458.jpg]

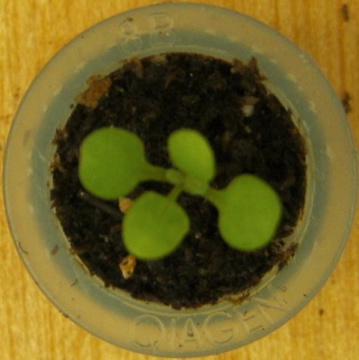

Supplement: Additional file 22 — Col-0 Top View Images for 3-D Model. Second half of images of Col-0 captured every 10 min for 5 days from the top view for the 3-D CG model. Table S2 lists the images used as key frames in the model. [file 13007_2015_75_MOESM22_ESM.zip › top_view_2/top_0459.jpg]

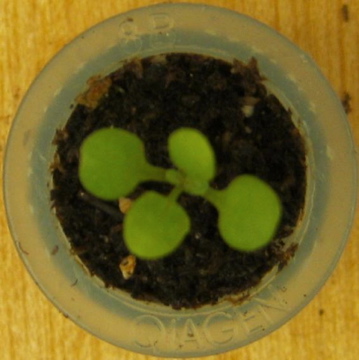

Supplement: Additional file 22 — Col-0 Top View Images for 3-D Model. Second half of images of Col-0 captured every 10 min for 5 days from the top view for the 3-D CG model. Table S2 lists the images used as key frames in the model. [file 13007_2015_75_MOESM22_ESM.zip › top_view_2/top_0460.jpg]

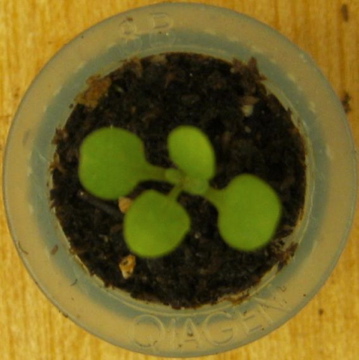

Supplement: Additional file 22 — Col-0 Top View Images for 3-D Model. Second half of images of Col-0 captured every 10 min for 5 days from the top view for the 3-D CG model. Table S2 lists the images used as key frames in the model. [file 13007_2015_75_MOESM22_ESM.zip › top_view_2/top_0461.jpg]

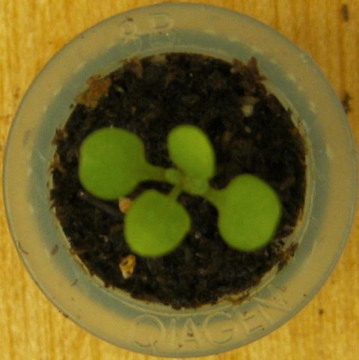

Supplement: Additional file 22 — Col-0 Top View Images for 3-D Model. Second half of images of Col-0 captured every 10 min for 5 days from the top view for the 3-D CG model. Table S2 lists the images used as key frames in the model. [file 13007_2015_75_MOESM22_ESM.zip › top_view_2/top_0462.jpg]

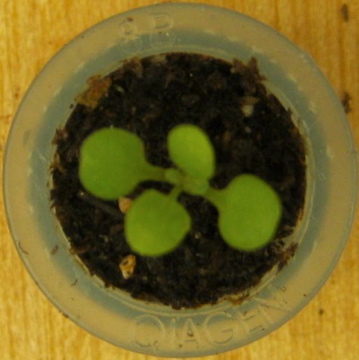

Supplement: Additional file 22 — Col-0 Top View Images for 3-D Model. Second half of images of Col-0 captured every 10 min for 5 days from the top view for the 3-D CG model. Table S2 lists the images used as key frames in the model. [file 13007_2015_75_MOESM22_ESM.zip › top_view_2/top_0463.jpg]

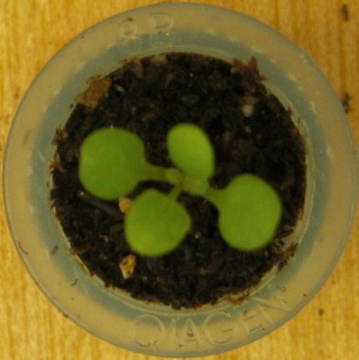

Supplement: Additional file 22 — Col-0 Top View Images for 3-D Model. Second half of images of Col-0 captured every 10 min for 5 days from the top view for the 3-D CG model. Table S2 lists the images used as key frames in the model. [file 13007_2015_75_MOESM22_ESM.zip › top_view_2/top_0464.jpg]

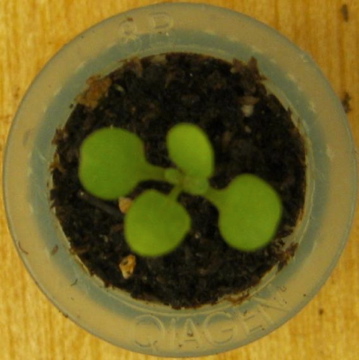

Supplement: Additional file 22 — Col-0 Top View Images for 3-D Model. Second half of images of Col-0 captured every 10 min for 5 days from the top view for the 3-D CG model. Table S2 lists the images used as key frames in the model. [file 13007_2015_75_MOESM22_ESM.zip › top_view_2/top_0465.jpg]

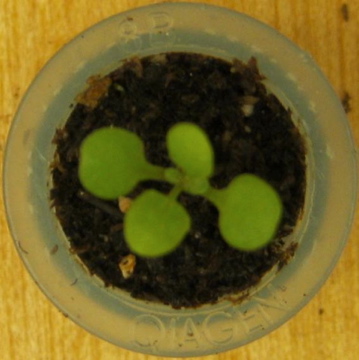

Supplement: Additional file 22 — Col-0 Top View Images for 3-D Model. Second half of images of Col-0 captured every 10 min for 5 days from the top view for the 3-D CG model. Table S2 lists the images used as key frames in the model. [file 13007_2015_75_MOESM22_ESM.zip › top_view_2/top_0466.jpg]

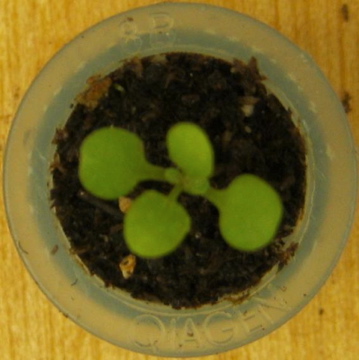

Supplement: Additional file 22 — Col-0 Top View Images for 3-D Model. Second half of images of Col-0 captured every 10 min for 5 days from the top view for the 3-D CG model. Table S2 lists the images used as key frames in the model. [file 13007_2015_75_MOESM22_ESM.zip › top_view_2/top_0467.jpg]

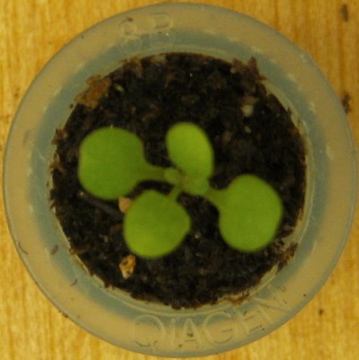

Supplement: Additional file 22 — Col-0 Top View Images for 3-D Model. Second half of images of Col-0 captured every 10 min for 5 days from the top view for the 3-D CG model. Table S2 lists the images used as key frames in the model. [file 13007_2015_75_MOESM22_ESM.zip › top_view_2/top_0468.jpg]

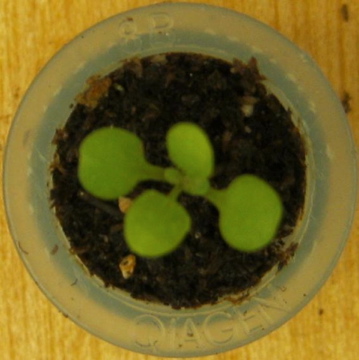

Supplement: Additional file 22 — Col-0 Top View Images for 3-D Model. Second half of images of Col-0 captured every 10 min for 5 days from the top view for the 3-D CG model. Table S2 lists the images used as key frames in the model. [file 13007_2015_75_MOESM22_ESM.zip › top_view_2/top_0469.jpg]

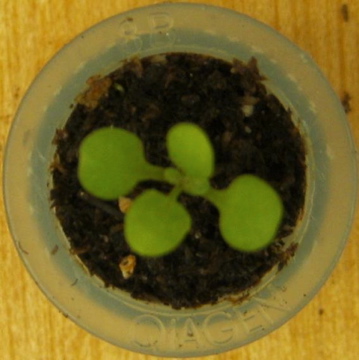

Supplement: Additional file 22 — Col-0 Top View Images for 3-D Model. Second half of images of Col-0 captured every 10 min for 5 days from the top view for the 3-D CG model. Table S2 lists the images used as key frames in the model. [file 13007_2015_75_MOESM22_ESM.zip › top_view_2/top_0470.jpg]

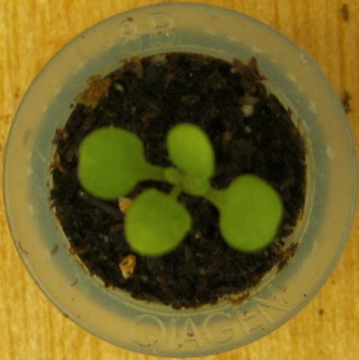

Supplement: Additional file 22 — Col-0 Top View Images for 3-D Model. Second half of images of Col-0 captured every 10 min for 5 days from the top view for the 3-D CG model. Table S2 lists the images used as key frames in the model. [file 13007_2015_75_MOESM22_ESM.zip › top_view_2/top_0471.jpg]

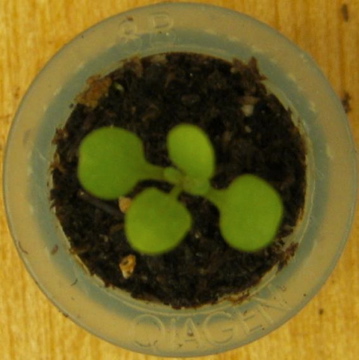

Supplement: Additional file 22 — Col-0 Top View Images for 3-D Model. Second half of images of Col-0 captured every 10 min for 5 days from the top view for the 3-D CG model. Table S2 lists the images used as key frames in the model. [file 13007_2015_75_MOESM22_ESM.zip › top_view_2/top_0472.jpg]

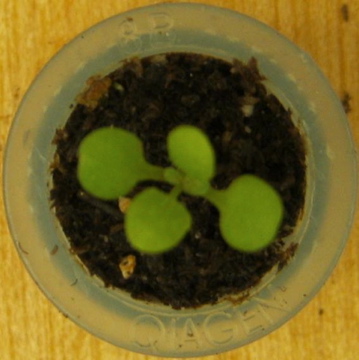

Supplement: Additional file 22 — Col-0 Top View Images for 3-D Model. Second half of images of Col-0 captured every 10 min for 5 days from the top view for the 3-D CG model. Table S2 lists the images used as key frames in the model. [file 13007_2015_75_MOESM22_ESM.zip › top_view_2/top_0473.jpg]

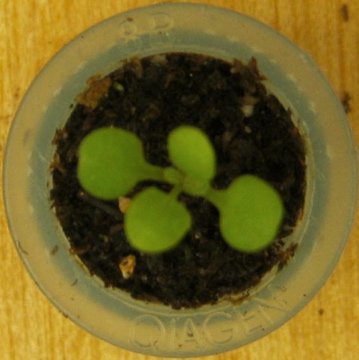

Supplement: Additional file 22 — Col-0 Top View Images for 3-D Model. Second half of images of Col-0 captured every 10 min for 5 days from the top view for the 3-D CG model. Table S2 lists the images used as key frames in the model. [file 13007_2015_75_MOESM22_ESM.zip › top_view_2/top_0474.jpg]

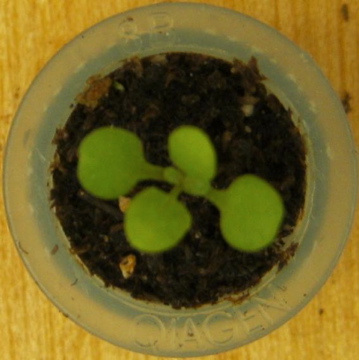

Supplement: Additional file 22 — Col-0 Top View Images for 3-D Model. Second half of images of Col-0 captured every 10 min for 5 days from the top view for the 3-D CG model. Table S2 lists the images used as key frames in the model. [file 13007_2015_75_MOESM22_ESM.zip › top_view_2/top_0475.jpg]

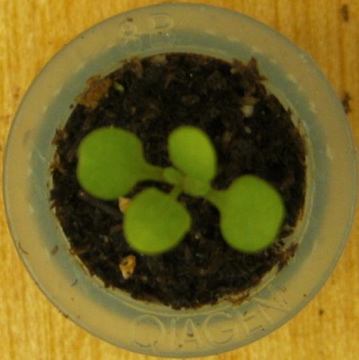

Supplement: Additional file 22 — Col-0 Top View Images for 3-D Model. Second half of images of Col-0 captured every 10 min for 5 days from the top view for the 3-D CG model. Table S2 lists the images used as key frames in the model. [file 13007_2015_75_MOESM22_ESM.zip › top_view_2/top_0476.jpg]

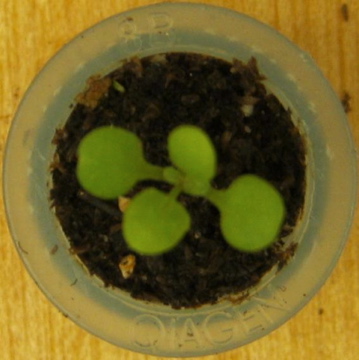

Supplement: Additional file 22 — Col-0 Top View Images for 3-D Model. Second half of images of Col-0 captured every 10 min for 5 days from the top view for the 3-D CG model. Table S2 lists the images used as key frames in the model. [file 13007_2015_75_MOESM22_ESM.zip › top_view_2/top_0477.jpg]

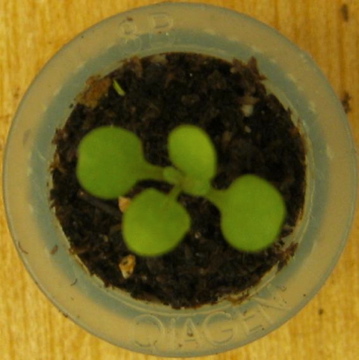

Supplement: Additional file 22 — Col-0 Top View Images for 3-D Model. Second half of images of Col-0 captured every 10 min for 5 days from the top view for the 3-D CG model. Table S2 lists the images used as key frames in the model. [file 13007_2015_75_MOESM22_ESM.zip › top_view_2/top_0478.jpg]

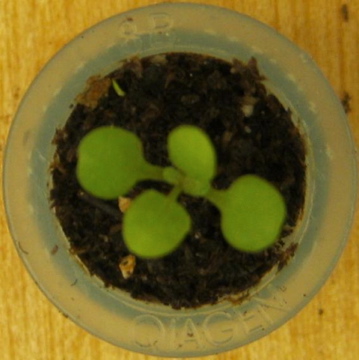

Supplement: Additional file 22 — Col-0 Top View Images for 3-D Model. Second half of images of Col-0 captured every 10 min for 5 days from the top view for the 3-D CG model. Table S2 lists the images used as key frames in the model. [file 13007_2015_75_MOESM22_ESM.zip › top_view_2/top_0479.jpg]

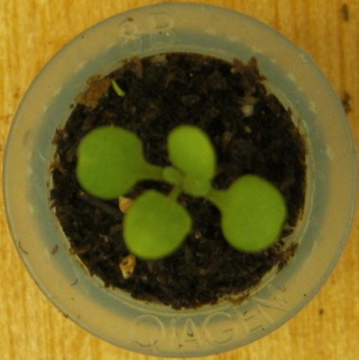

Supplement: Additional file 22 — Col-0 Top View Images for 3-D Model. Second half of images of Col-0 captured every 10 min for 5 days from the top view for the 3-D CG model. Table S2 lists the images used as key frames in the model. [file 13007_2015_75_MOESM22_ESM.zip › top_view_2/top_0480.jpg]

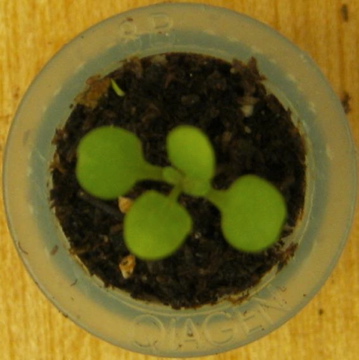

Supplement: Additional file 22 — Col-0 Top View Images for 3-D Model. Second half of images of Col-0 captured every 10 min for 5 days from the top view for the 3-D CG model. Table S2 lists the images used as key frames in the model. [file 13007_2015_75_MOESM22_ESM.zip › top_view_2/top_0481.jpg]

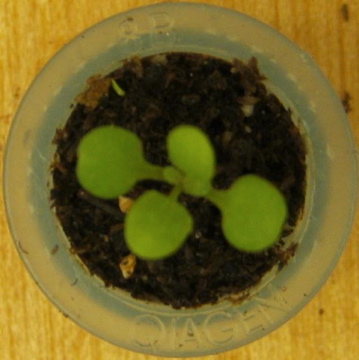

Supplement: Additional file 22 — Col-0 Top View Images for 3-D Model. Second half of images of Col-0 captured every 10 min for 5 days from the top view for the 3-D CG model. Table S2 lists the images used as key frames in the model. [file 13007_2015_75_MOESM22_ESM.zip › top_view_2/top_0482.jpg]

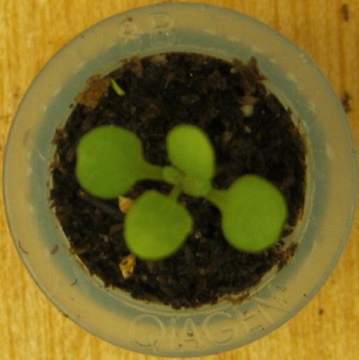

Supplement: Additional file 22 — Col-0 Top View Images for 3-D Model. Second half of images of Col-0 captured every 10 min for 5 days from the top view for the 3-D CG model. Table S2 lists the images used as key frames in the model. [file 13007_2015_75_MOESM22_ESM.zip › top_view_2/top_0483.jpg]

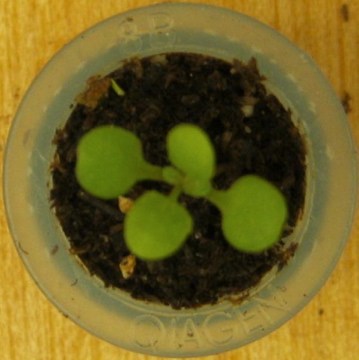

Supplement: Additional file 22 — Col-0 Top View Images for 3-D Model. Second half of images of Col-0 captured every 10 min for 5 days from the top view for the 3-D CG model. Table S2 lists the images used as key frames in the model. [file 13007_2015_75_MOESM22_ESM.zip › top_view_2/top_0484.jpg]

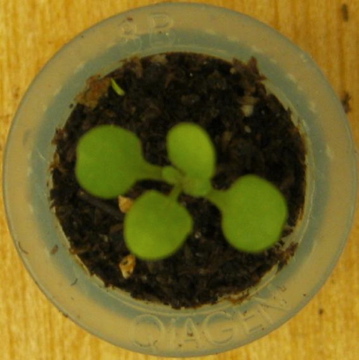

Supplement: Additional file 22 — Col-0 Top View Images for 3-D Model. Second half of images of Col-0 captured every 10 min for 5 days from the top view for the 3-D CG model. Table S2 lists the images used as key frames in the model. [file 13007_2015_75_MOESM22_ESM.zip › top_view_2/top_0485.jpg]

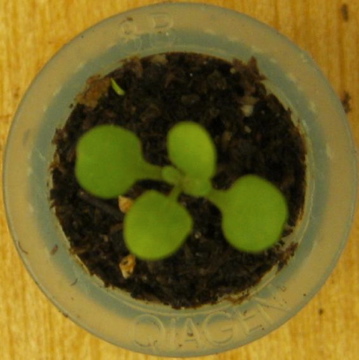

Supplement: Additional file 22 — Col-0 Top View Images for 3-D Model. Second half of images of Col-0 captured every 10 min for 5 days from the top view for the 3-D CG model. Table S2 lists the images used as key frames in the model. [file 13007_2015_75_MOESM22_ESM.zip › top_view_2/top_0486.jpg]

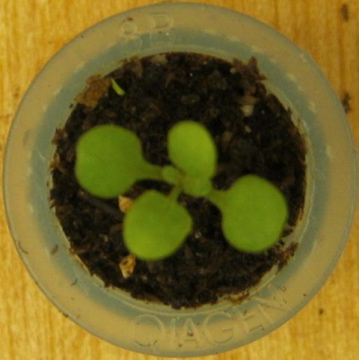

Supplement: Additional file 22 — Col-0 Top View Images for 3-D Model. Second half of images of Col-0 captured every 10 min for 5 days from the top view for the 3-D CG model. Table S2 lists the images used as key frames in the model. [file 13007_2015_75_MOESM22_ESM.zip › top_view_2/top_0487.jpg]

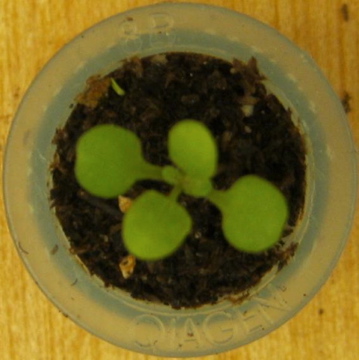

Supplement: Additional file 22 — Col-0 Top View Images for 3-D Model. Second half of images of Col-0 captured every 10 min for 5 days from the top view for the 3-D CG model. Table S2 lists the images used as key frames in the model. [file 13007_2015_75_MOESM22_ESM.zip › top_view_2/top_0488.jpg]

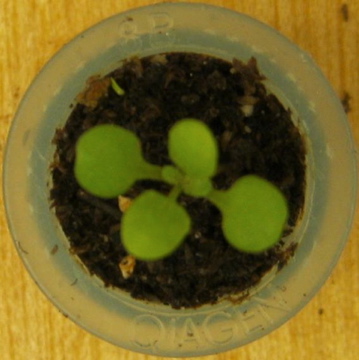

Supplement: Additional file 22 — Col-0 Top View Images for 3-D Model. Second half of images of Col-0 captured every 10 min for 5 days from the top view for the 3-D CG model. Table S2 lists the images used as key frames in the model. [file 13007_2015_75_MOESM22_ESM.zip › top_view_2/top_0489.jpg]

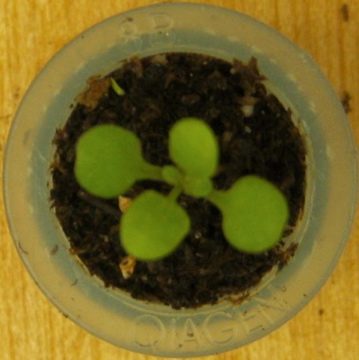

Supplement: Additional file 22 — Col-0 Top View Images for 3-D Model. Second half of images of Col-0 captured every 10 min for 5 days from the top view for the 3-D CG model. Table S2 lists the images used as key frames in the model. [file 13007_2015_75_MOESM22_ESM.zip › top_view_2/top_0490.jpg]

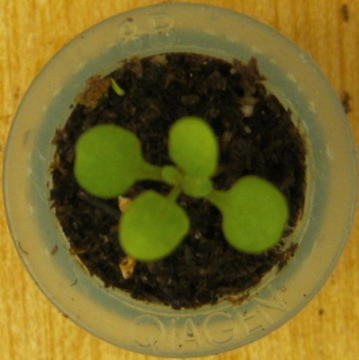

Supplement: Additional file 22 — Col-0 Top View Images for 3-D Model. Second half of images of Col-0 captured every 10 min for 5 days from the top view for the 3-D CG model. Table S2 lists the images used as key frames in the model. [file 13007_2015_75_MOESM22_ESM.zip › top_view_2/top_0491.jpg]

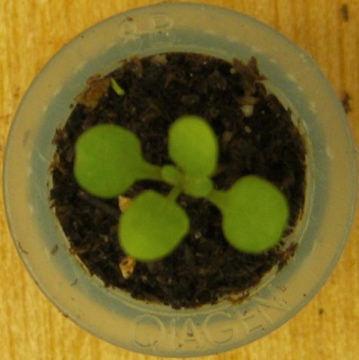

Supplement: Additional file 22 — Col-0 Top View Images for 3-D Model. Second half of images of Col-0 captured every 10 min for 5 days from the top view for the 3-D CG model. Table S2 lists the images used as key frames in the model. [file 13007_2015_75_MOESM22_ESM.zip › top_view_2/top_0492.jpg]

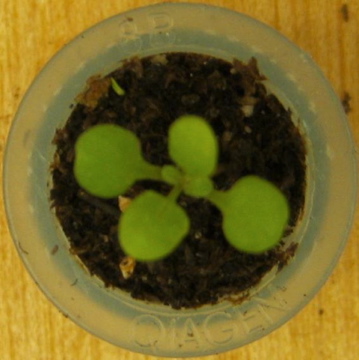

Supplement: Additional file 22 — Col-0 Top View Images for 3-D Model. Second half of images of Col-0 captured every 10 min for 5 days from the top view for the 3-D CG model. Table S2 lists the images used as key frames in the model. [file 13007_2015_75_MOESM22_ESM.zip › top_view_2/top_0493.jpg]

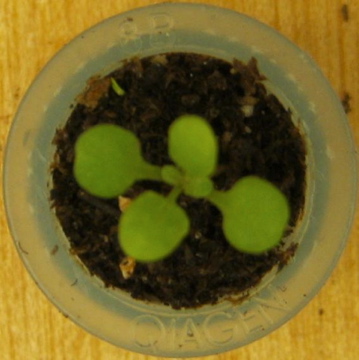

Supplement: Additional file 22 — Col-0 Top View Images for 3-D Model. Second half of images of Col-0 captured every 10 min for 5 days from the top view for the 3-D CG model. Table S2 lists the images used as key frames in the model. [file 13007_2015_75_MOESM22_ESM.zip › top_view_2/top_0494.jpg]

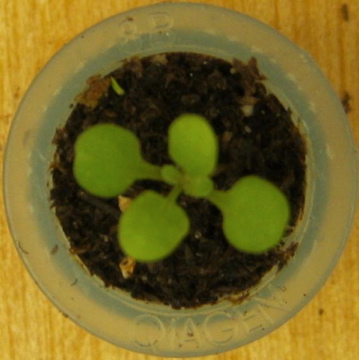

Supplement: Additional file 22 — Col-0 Top View Images for 3-D Model. Second half of images of Col-0 captured every 10 min for 5 days from the top view for the 3-D CG model. Table S2 lists the images used as key frames in the model. [file 13007_2015_75_MOESM22_ESM.zip › top_view_2/top_0495.jpg]

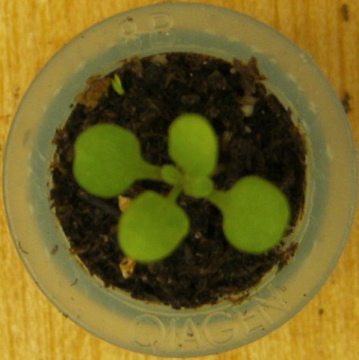

Supplement: Additional file 22 — Col-0 Top View Images for 3-D Model. Second half of images of Col-0 captured every 10 min for 5 days from the top view for the 3-D CG model. Table S2 lists the images used as key frames in the model. [file 13007_2015_75_MOESM22_ESM.zip › top_view_2/top_0496.jpg]

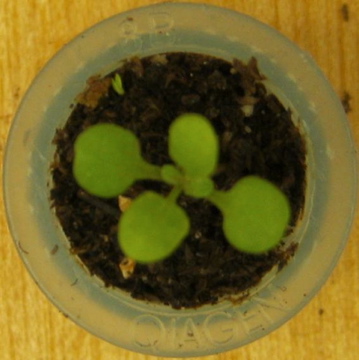

Supplement: Additional file 22 — Col-0 Top View Images for 3-D Model. Second half of images of Col-0 captured every 10 min for 5 days from the top view for the 3-D CG model. Table S2 lists the images used as key frames in the model. [file 13007_2015_75_MOESM22_ESM.zip › top_view_2/top_0497.jpg]

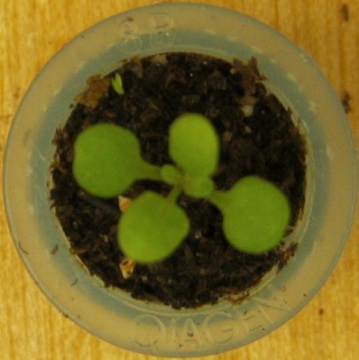

Supplement: Additional file 22 — Col-0 Top View Images for 3-D Model. Second half of images of Col-0 captured every 10 min for 5 days from the top view for the 3-D CG model. Table S2 lists the images used as key frames in the model. [file 13007_2015_75_MOESM22_ESM.zip › top_view_2/top_0498.jpg]

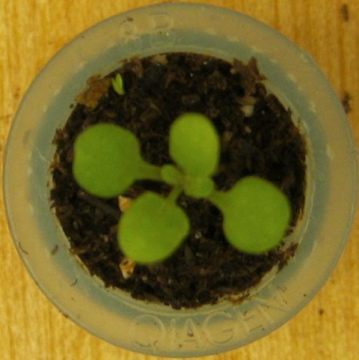

Supplement: Additional file 22 — Col-0 Top View Images for 3-D Model. Second half of images of Col-0 captured every 10 min for 5 days from the top view for the 3-D CG model. Table S2 lists the images used as key frames in the model. [file 13007_2015_75_MOESM22_ESM.zip › top_view_2/top_0499.jpg]

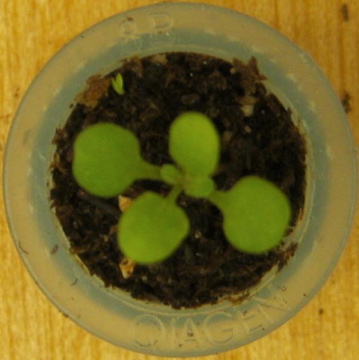

Supplement: Additional file 22 — Col-0 Top View Images for 3-D Model. Second half of images of Col-0 captured every 10 min for 5 days from the top view for the 3-D CG model. Table S2 lists the images used as key frames in the model. [file 13007_2015_75_MOESM22_ESM.zip › top_view_2/top_0500.jpg]

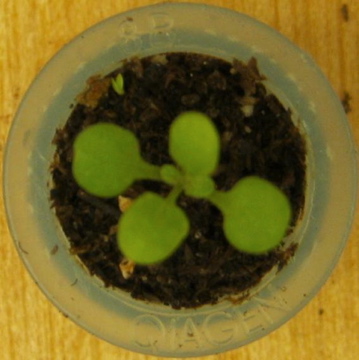

Supplement: Additional file 22 — Col-0 Top View Images for 3-D Model. Second half of images of Col-0 captured every 10 min for 5 days from the top view for the 3-D CG model. Table S2 lists the images used as key frames in the model. [file 13007_2015_75_MOESM22_ESM.zip › top_view_2/top_0501.jpg]

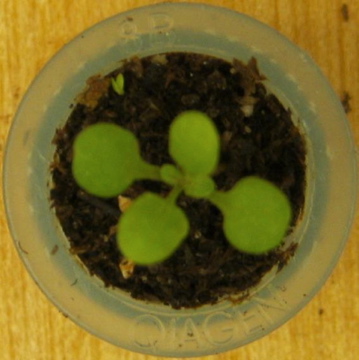

Supplement: Additional file 22 — Col-0 Top View Images for 3-D Model. Second half of images of Col-0 captured every 10 min for 5 days from the top view for the 3-D CG model. Table S2 lists the images used as key frames in the model. [file 13007_2015_75_MOESM22_ESM.zip › top_view_2/top_0502.jpg]

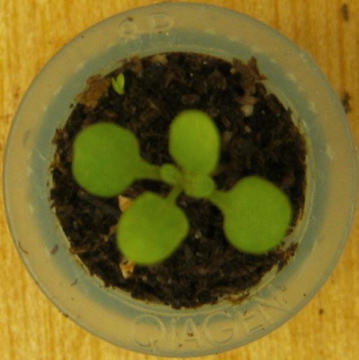

Supplement: Additional file 22 — Col-0 Top View Images for 3-D Model. Second half of images of Col-0 captured every 10 min for 5 days from the top view for the 3-D CG model. Table S2 lists the images used as key frames in the model. [file 13007_2015_75_MOESM22_ESM.zip › top_view_2/top_0503.jpg]

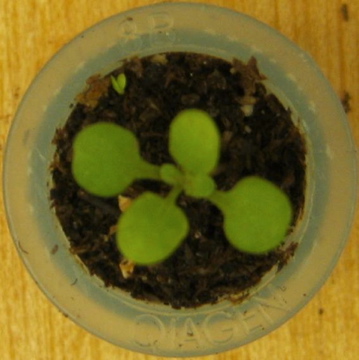

Supplement: Additional file 22 — Col-0 Top View Images for 3-D Model. Second half of images of Col-0 captured every 10 min for 5 days from the top view for the 3-D CG model. Table S2 lists the images used as key frames in the model. [file 13007_2015_75_MOESM22_ESM.zip › top_view_2/top_0504.jpg]

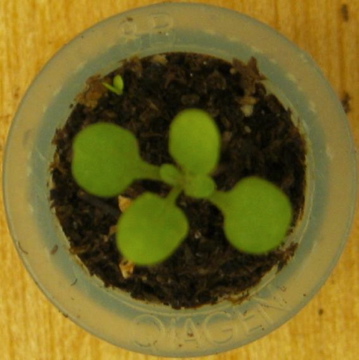

Supplement: Additional file 22 — Col-0 Top View Images for 3-D Model. Second half of images of Col-0 captured every 10 min for 5 days from the top view for the 3-D CG model. Table S2 lists the images used as key frames in the model. [file 13007_2015_75_MOESM22_ESM.zip › top_view_2/top_0505.jpg]

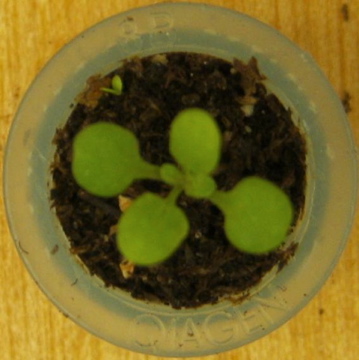

Supplement: Additional file 22 — Col-0 Top View Images for 3-D Model. Second half of images of Col-0 captured every 10 min for 5 days from the top view for the 3-D CG model. Table S2 lists the images used as key frames in the model. [file 13007_2015_75_MOESM22_ESM.zip › top_view_2/top_0506.jpg]

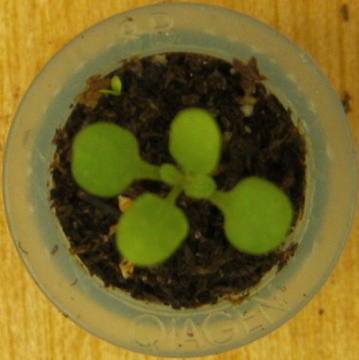

Supplement: Additional file 22 — Col-0 Top View Images for 3-D Model. Second half of images of Col-0 captured every 10 min for 5 days from the top view for the 3-D CG model. Table S2 lists the images used as key frames in the model. [file 13007_2015_75_MOESM22_ESM.zip › top_view_2/top_0507.jpg]

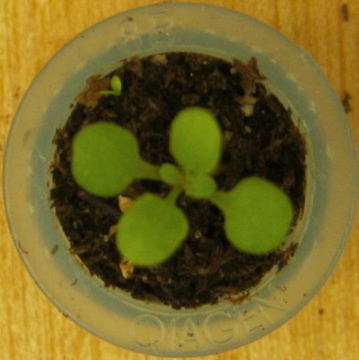

Supplement: Additional file 22 — Col-0 Top View Images for 3-D Model. Second half of images of Col-0 captured every 10 min for 5 days from the top view for the 3-D CG model. Table S2 lists the images used as key frames in the model. [file 13007_2015_75_MOESM22_ESM.zip › top_view_2/top_0508.jpg]

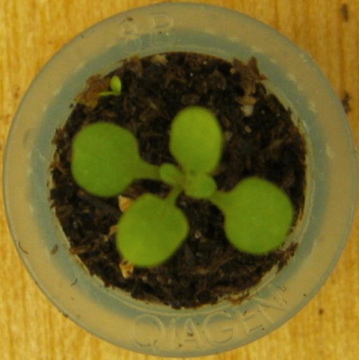

Supplement: Additional file 22 — Col-0 Top View Images for 3-D Model. Second half of images of Col-0 captured every 10 min for 5 days from the top view for the 3-D CG model. Table S2 lists the images used as key frames in the model. [file 13007_2015_75_MOESM22_ESM.zip › top_view_2/top_0509.jpg]

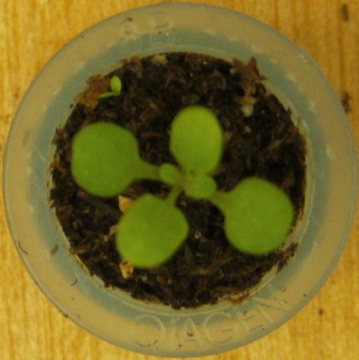

Supplement: Additional file 22 — Col-0 Top View Images for 3-D Model. Second half of images of Col-0 captured every 10 min for 5 days from the top view for the 3-D CG model. Table S2 lists the images used as key frames in the model. [file 13007_2015_75_MOESM22_ESM.zip › top_view_2/top_0510.jpg]

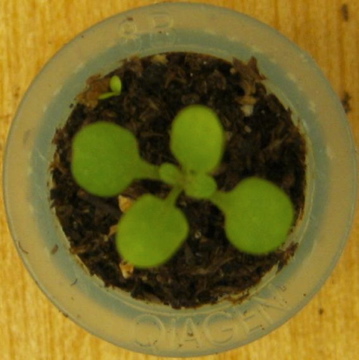

Supplement: Additional file 22 — Col-0 Top View Images for 3-D Model. Second half of images of Col-0 captured every 10 min for 5 days from the top view for the 3-D CG model. Table S2 lists the images used as key frames in the model. [file 13007_2015_75_MOESM22_ESM.zip › top_view_2/top_0511.jpg]

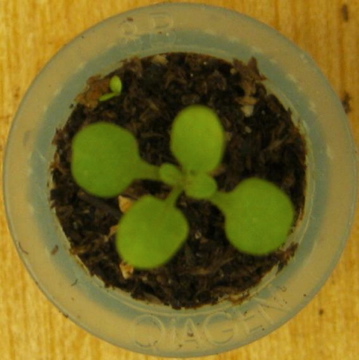

Supplement: Additional file 22 — Col-0 Top View Images for 3-D Model. Second half of images of Col-0 captured every 10 min for 5 days from the top view for the 3-D CG model. Table S2 lists the images used as key frames in the model. [file 13007_2015_75_MOESM22_ESM.zip › top_view_2/top_0512.jpg]

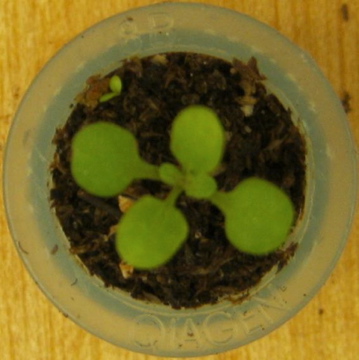

Supplement: Additional file 22 — Col-0 Top View Images for 3-D Model. Second half of images of Col-0 captured every 10 min for 5 days from the top view for the 3-D CG model. Table S2 lists the images used as key frames in the model. [file 13007_2015_75_MOESM22_ESM.zip › top_view_2/top_0513.jpg]

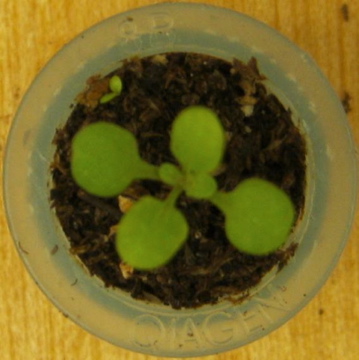

Supplement: Additional file 22 — Col-0 Top View Images for 3-D Model. Second half of images of Col-0 captured every 10 min for 5 days from the top view for the 3-D CG model. Table S2 lists the images used as key frames in the model. [file 13007_2015_75_MOESM22_ESM.zip › top_view_2/top_0514.jpg]

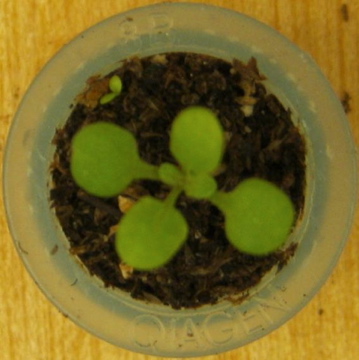

Supplement: Additional file 22 — Col-0 Top View Images for 3-D Model. Second half of images of Col-0 captured every 10 min for 5 days from the top view for the 3-D CG model. Table S2 lists the images used as key frames in the model. [file 13007_2015_75_MOESM22_ESM.zip › top_view_2/top_0515.jpg]

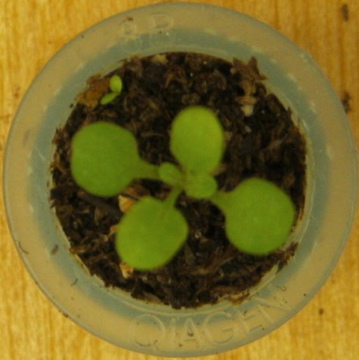

Supplement: Additional file 22 — Col-0 Top View Images for 3-D Model. Second half of images of Col-0 captured every 10 min for 5 days from the top view for the 3-D CG model. Table S2 lists the images used as key frames in the model. [file 13007_2015_75_MOESM22_ESM.zip › top_view_2/top_0516.jpg]

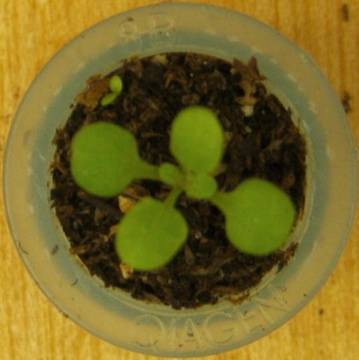

Supplement: Additional file 22 — Col-0 Top View Images for 3-D Model. Second half of images of Col-0 captured every 10 min for 5 days from the top view for the 3-D CG model. Table S2 lists the images used as key frames in the model. [file 13007_2015_75_MOESM22_ESM.zip › top_view_2/top_0517.jpg]

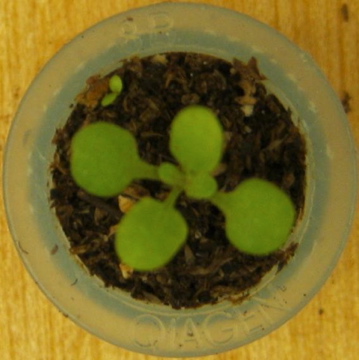

Supplement: Additional file 22 — Col-0 Top View Images for 3-D Model. Second half of images of Col-0 captured every 10 min for 5 days from the top view for the 3-D CG model. Table S2 lists the images used as key frames in the model. [file 13007_2015_75_MOESM22_ESM.zip › top_view_2/top_0518.jpg]

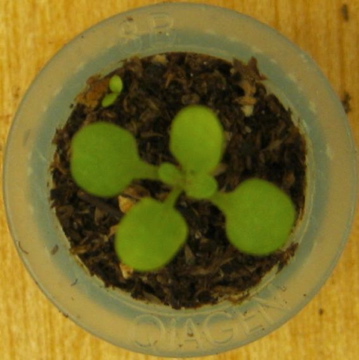

Supplement: Additional file 22 — Col-0 Top View Images for 3-D Model. Second half of images of Col-0 captured every 10 min for 5 days from the top view for the 3-D CG model. Table S2 lists the images used as key frames in the model. [file 13007_2015_75_MOESM22_ESM.zip › top_view_2/top_0519.jpg]

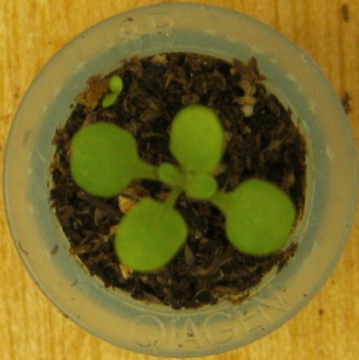

Supplement: Additional file 22 — Col-0 Top View Images for 3-D Model. Second half of images of Col-0 captured every 10 min for 5 days from the top view for the 3-D CG model. Table S2 lists the images used as key frames in the model. [file 13007_2015_75_MOESM22_ESM.zip › top_view_2/top_0520.jpg]

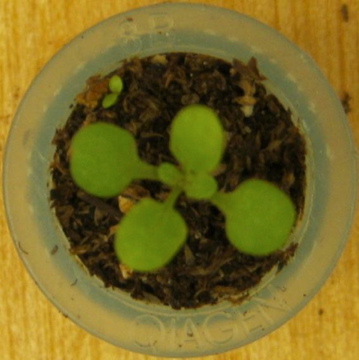

Supplement: Additional file 22 — Col-0 Top View Images for 3-D Model. Second half of images of Col-0 captured every 10 min for 5 days from the top view for the 3-D CG model. Table S2 lists the images used as key frames in the model. [file 13007_2015_75_MOESM22_ESM.zip › top_view_2/top_0521.jpg]

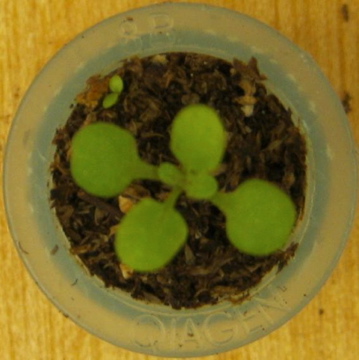

Supplement: Additional file 22 — Col-0 Top View Images for 3-D Model. Second half of images of Col-0 captured every 10 min for 5 days from the top view for the 3-D CG model. Table S2 lists the images used as key frames in the model. [file 13007_2015_75_MOESM22_ESM.zip › top_view_2/top_0522.jpg]

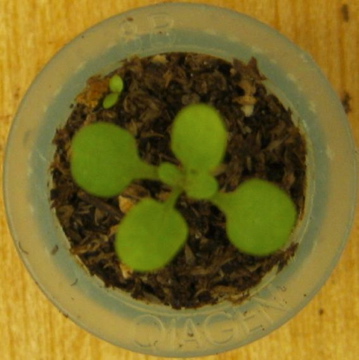

Supplement: Additional file 22 — Col-0 Top View Images for 3-D Model. Second half of images of Col-0 captured every 10 min for 5 days from the top view for the 3-D CG model. Table S2 lists the images used as key frames in the model. [file 13007_2015_75_MOESM22_ESM.zip › top_view_2/top_0523.jpg]

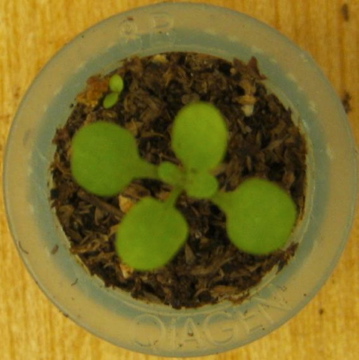

Supplement: Additional file 22 — Col-0 Top View Images for 3-D Model. Second half of images of Col-0 captured every 10 min for 5 days from the top view for the 3-D CG model. Table S2 lists the images used as key frames in the model. [file 13007_2015_75_MOESM22_ESM.zip › top_view_2/top_0524.jpg]

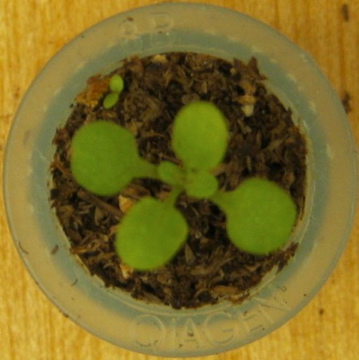

Supplement: Additional file 22 — Col-0 Top View Images for 3-D Model. Second half of images of Col-0 captured every 10 min for 5 days from the top view for the 3-D CG model. Table S2 lists the images used as key frames in the model. [file 13007_2015_75_MOESM22_ESM.zip › top_view_2/top_0525.jpg]

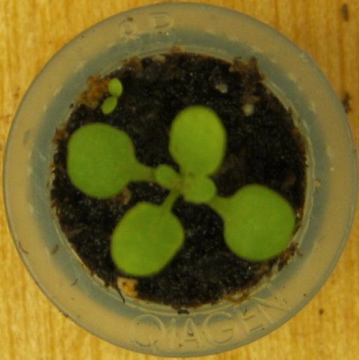

Supplement: Additional file 22 — Col-0 Top View Images for 3-D Model. Second half of images of Col-0 captured every 10 min for 5 days from the top view for the 3-D CG model. Table S2 lists the images used as key frames in the model. [file 13007_2015_75_MOESM22_ESM.zip › top_view_2/top_0526.jpg]

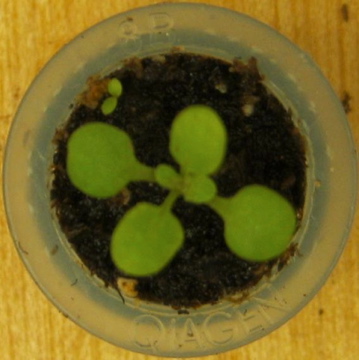

Supplement: Additional file 22 — Col-0 Top View Images for 3-D Model. Second half of images of Col-0 captured every 10 min for 5 days from the top view for the 3-D CG model. Table S2 lists the images used as key frames in the model. [file 13007_2015_75_MOESM22_ESM.zip › top_view_2/top_0527.jpg]

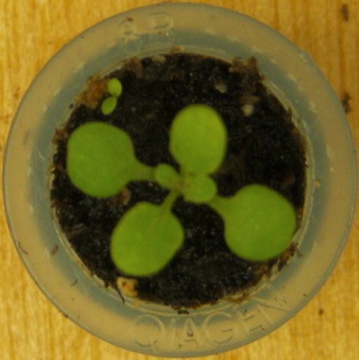

Supplement: Additional file 22 — Col-0 Top View Images for 3-D Model. Second half of images of Col-0 captured every 10 min for 5 days from the top view for the 3-D CG model. Table S2 lists the images used as key frames in the model. [file 13007_2015_75_MOESM22_ESM.zip › top_view_2/top_0528.jpg]

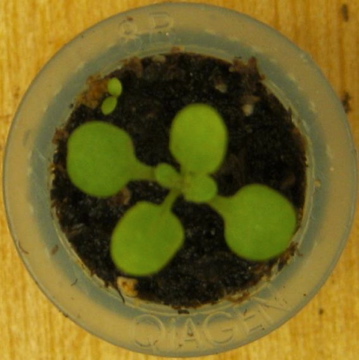

Supplement: Additional file 22 — Col-0 Top View Images for 3-D Model. Second half of images of Col-0 captured every 10 min for 5 days from the top view for the 3-D CG model. Table S2 lists the images used as key frames in the model. [file 13007_2015_75_MOESM22_ESM.zip › top_view_2/top_0529.jpg]

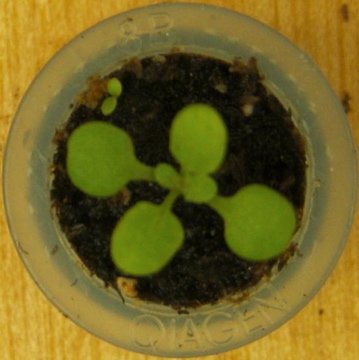

Supplement: Additional file 22 — Col-0 Top View Images for 3-D Model. Second half of images of Col-0 captured every 10 min for 5 days from the top view for the 3-D CG model. Table S2 lists the images used as key frames in the model. [file 13007_2015_75_MOESM22_ESM.zip › top_view_2/top_0530.jpg]

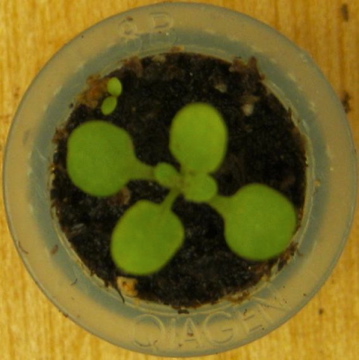

Supplement: Additional file 22 — Col-0 Top View Images for 3-D Model. Second half of images of Col-0 captured every 10 min for 5 days from the top view for the 3-D CG model. Table S2 lists the images used as key frames in the model. [file 13007_2015_75_MOESM22_ESM.zip › top_view_2/top_0531.jpg]

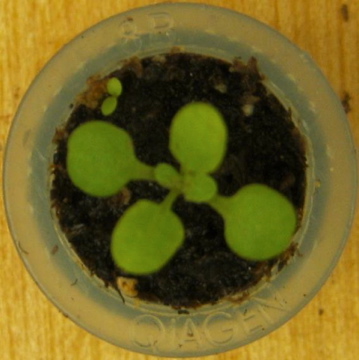

Supplement: Additional file 22 — Col-0 Top View Images for 3-D Model. Second half of images of Col-0 captured every 10 min for 5 days from the top view for the 3-D CG model. Table S2 lists the images used as key frames in the model. [file 13007_2015_75_MOESM22_ESM.zip › top_view_2/top_0532.jpg]

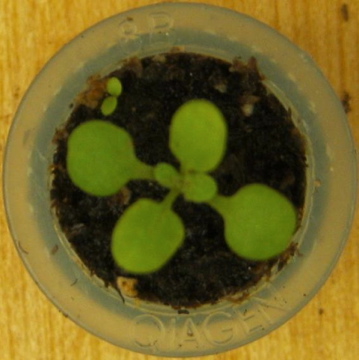

Supplement: Additional file 22 — Col-0 Top View Images for 3-D Model. Second half of images of Col-0 captured every 10 min for 5 days from the top view for the 3-D CG model. Table S2 lists the images used as key frames in the model. [file 13007_2015_75_MOESM22_ESM.zip › top_view_2/top_0533.jpg]

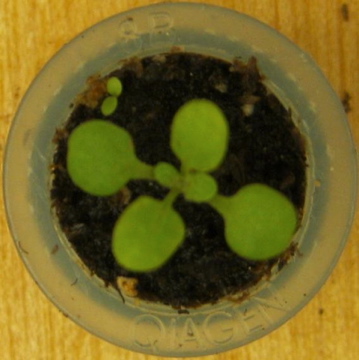

Supplement: Additional file 22 — Col-0 Top View Images for 3-D Model. Second half of images of Col-0 captured every 10 min for 5 days from the top view for the 3-D CG model. Table S2 lists the images used as key frames in the model. [file 13007_2015_75_MOESM22_ESM.zip › top_view_2/top_0534.jpg]

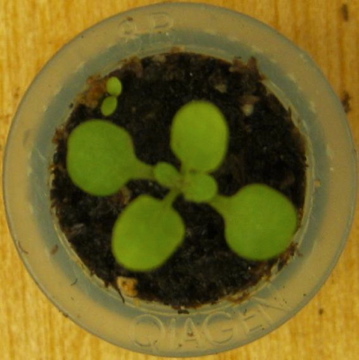

Supplement: Additional file 22 — Col-0 Top View Images for 3-D Model. Second half of images of Col-0 captured every 10 min for 5 days from the top view for the 3-D CG model. Table S2 lists the images used as key frames in the model. [file 13007_2015_75_MOESM22_ESM.zip › top_view_2/top_0535.jpg]

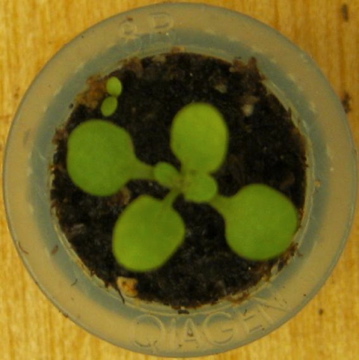

Supplement: Additional file 22 — Col-0 Top View Images for 3-D Model. Second half of images of Col-0 captured every 10 min for 5 days from the top view for the 3-D CG model. Table S2 lists the images used as key frames in the model. [file 13007_2015_75_MOESM22_ESM.zip › top_view_2/top_0536.jpg]

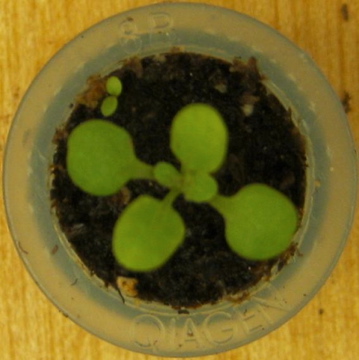

Supplement: Additional file 22 — Col-0 Top View Images for 3-D Model. Second half of images of Col-0 captured every 10 min for 5 days from the top view for the 3-D CG model. Table S2 lists the images used as key frames in the model. [file 13007_2015_75_MOESM22_ESM.zip › top_view_2/top_0537.jpg]

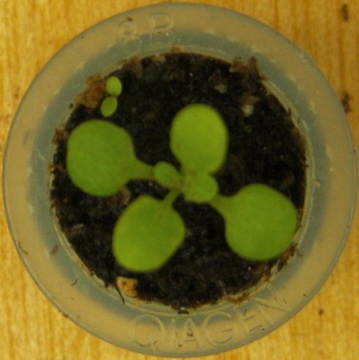

Supplement: Additional file 22 — Col-0 Top View Images for 3-D Model. Second half of images of Col-0 captured every 10 min for 5 days from the top view for the 3-D CG model. Table S2 lists the images used as key frames in the model. [file 13007_2015_75_MOESM22_ESM.zip › top_view_2/top_0538.jpg]

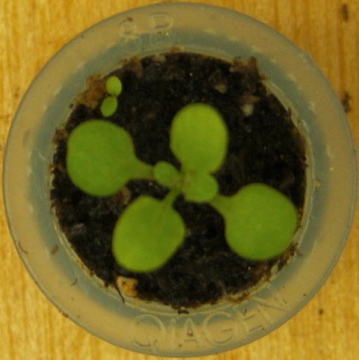

Supplement: Additional file 22 — Col-0 Top View Images for 3-D Model. Second half of images of Col-0 captured every 10 min for 5 days from the top view for the 3-D CG model. Table S2 lists the images used as key frames in the model. [file 13007_2015_75_MOESM22_ESM.zip › top_view_2/top_0539.jpg]

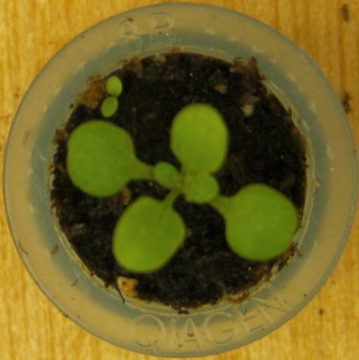

Supplement: Additional file 22 — Col-0 Top View Images for 3-D Model. Second half of images of Col-0 captured every 10 min for 5 days from the top view for the 3-D CG model. Table S2 lists the images used as key frames in the model. [file 13007_2015_75_MOESM22_ESM.zip › top_view_2/top_0540.jpg]

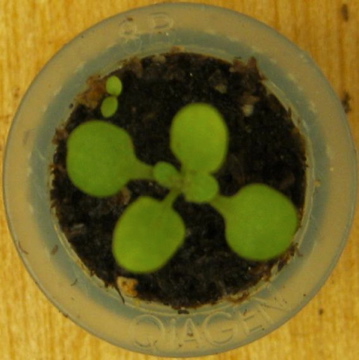

Supplement: Additional file 22 — Col-0 Top View Images for 3-D Model. Second half of images of Col-0 captured every 10 min for 5 days from the top view for the 3-D CG model. Table S2 lists the images used as key frames in the model. [file 13007_2015_75_MOESM22_ESM.zip › top_view_2/top_0541.jpg]

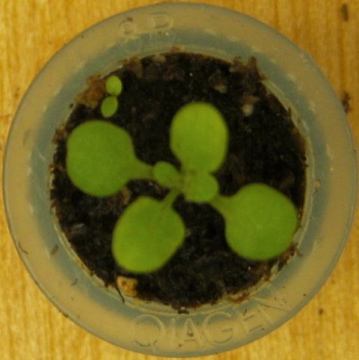

Supplement: Additional file 22 — Col-0 Top View Images for 3-D Model. Second half of images of Col-0 captured every 10 min for 5 days from the top view for the 3-D CG model. Table S2 lists the images used as key frames in the model. [file 13007_2015_75_MOESM22_ESM.zip › top_view_2/top_0542.jpg]

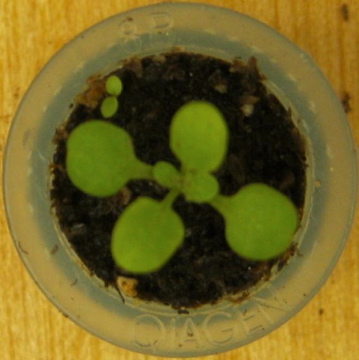

Supplement: Additional file 22 — Col-0 Top View Images for 3-D Model. Second half of images of Col-0 captured every 10 min for 5 days from the top view for the 3-D CG model. Table S2 lists the images used as key frames in the model. [file 13007_2015_75_MOESM22_ESM.zip › top_view_2/top_0543.jpg]

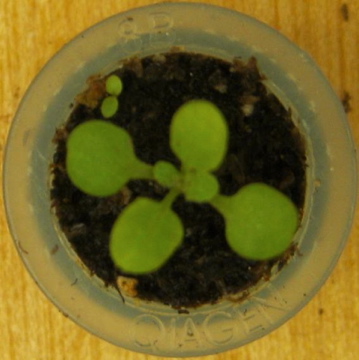

Supplement: Additional file 22 — Col-0 Top View Images for 3-D Model. Second half of images of Col-0 captured every 10 min for 5 days from the top view for the 3-D CG model. Table S2 lists the images used as key frames in the model. [file 13007_2015_75_MOESM22_ESM.zip › top_view_2/top_0544.jpg]

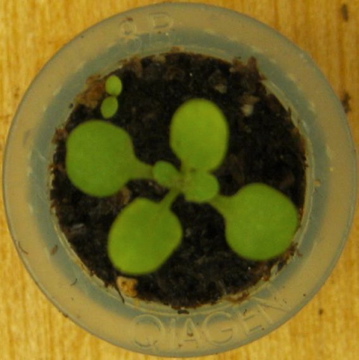

Supplement: Additional file 22 — Col-0 Top View Images for 3-D Model. Second half of images of Col-0 captured every 10 min for 5 days from the top view for the 3-D CG model. Table S2 lists the images used as key frames in the model. [file 13007_2015_75_MOESM22_ESM.zip › top_view_2/top_0545.jpg]
