# Supplementary figures and images for: TRiP: Tracking Rhythms in Plants, an automated leaf movement analysis program for circadian period estimation (part 9 of 10)
Source: Plant Methods. 2015 May 3;11:33. doi: 10.1186/s13007-015-0075-5 (PMC4445800; doi:10.1186/s13007-015-0075-5)

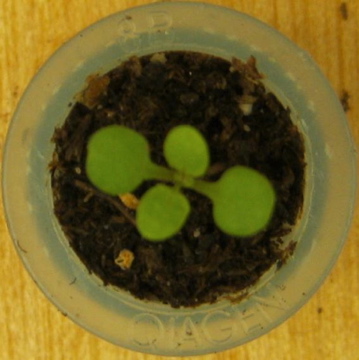

Supplement: Additional file 22 — Col-0 Top View Images for 3-D Model. Second half of images of Col-0 captured every 10 min for 5 days from the top view for the 3-D CG model. Table S2 lists the images used as key frames in the model. [file 13007_2015_75_MOESM22_ESM.zip › top_view_2/top_0354.jpg]

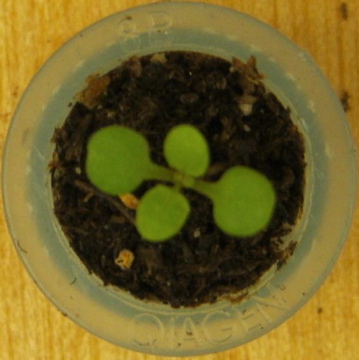

Supplement: Additional file 22 — Col-0 Top View Images for 3-D Model. Second half of images of Col-0 captured every 10 min for 5 days from the top view for the 3-D CG model. Table S2 lists the images used as key frames in the model. [file 13007_2015_75_MOESM22_ESM.zip › top_view_2/top_0355.jpg]

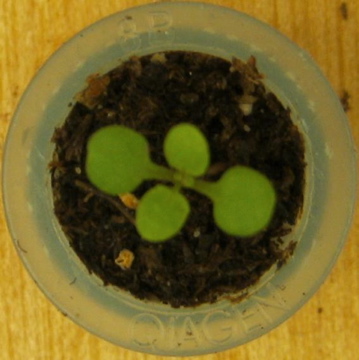

Supplement: Additional file 22 — Col-0 Top View Images for 3-D Model. Second half of images of Col-0 captured every 10 min for 5 days from the top view for the 3-D CG model. Table S2 lists the images used as key frames in the model. [file 13007_2015_75_MOESM22_ESM.zip › top_view_2/top_0356.jpg]

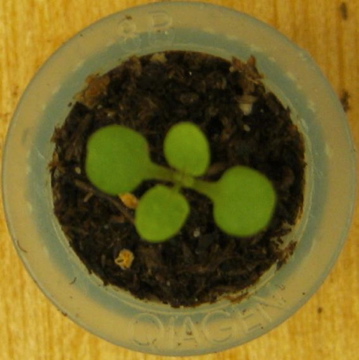

Supplement: Additional file 22 — Col-0 Top View Images for 3-D Model. Second half of images of Col-0 captured every 10 min for 5 days from the top view for the 3-D CG model. Table S2 lists the images used as key frames in the model. [file 13007_2015_75_MOESM22_ESM.zip › top_view_2/top_0357.jpg]

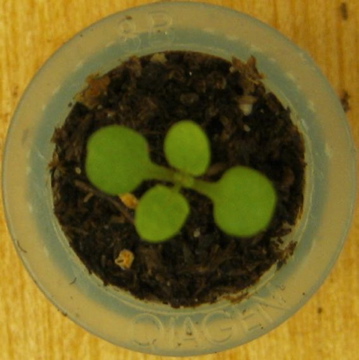

Supplement: Additional file 22 — Col-0 Top View Images for 3-D Model. Second half of images of Col-0 captured every 10 min for 5 days from the top view for the 3-D CG model. Table S2 lists the images used as key frames in the model. [file 13007_2015_75_MOESM22_ESM.zip › top_view_2/top_0358.jpg]

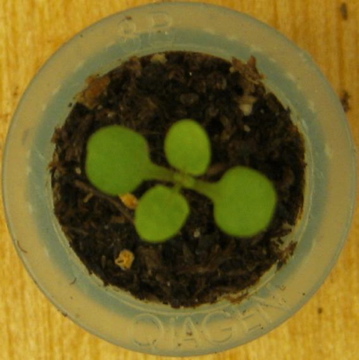

Supplement: Additional file 22 — Col-0 Top View Images for 3-D Model. Second half of images of Col-0 captured every 10 min for 5 days from the top view for the 3-D CG model. Table S2 lists the images used as key frames in the model. [file 13007_2015_75_MOESM22_ESM.zip › top_view_2/top_0359.jpg]

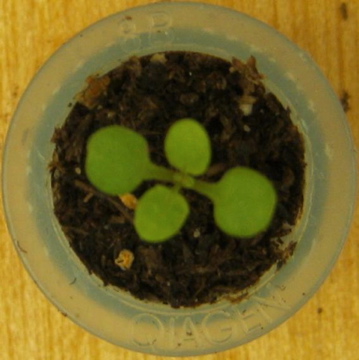

Supplement: Additional file 22 — Col-0 Top View Images for 3-D Model. Second half of images of Col-0 captured every 10 min for 5 days from the top view for the 3-D CG model. Table S2 lists the images used as key frames in the model. [file 13007_2015_75_MOESM22_ESM.zip › top_view_2/top_0360.jpg]

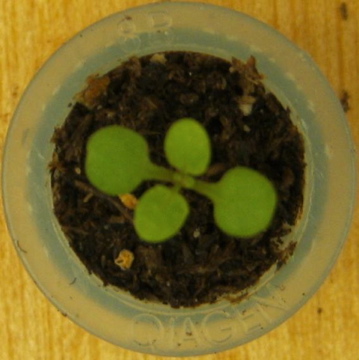

Supplement: Additional file 22 — Col-0 Top View Images for 3-D Model. Second half of images of Col-0 captured every 10 min for 5 days from the top view for the 3-D CG model. Table S2 lists the images used as key frames in the model. [file 13007_2015_75_MOESM22_ESM.zip › top_view_2/top_0361.jpg]

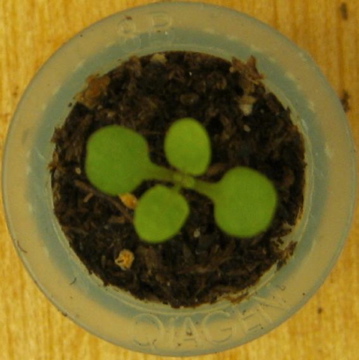

Supplement: Additional file 22 — Col-0 Top View Images for 3-D Model. Second half of images of Col-0 captured every 10 min for 5 days from the top view for the 3-D CG model. Table S2 lists the images used as key frames in the model. [file 13007_2015_75_MOESM22_ESM.zip › top_view_2/top_0362.jpg]

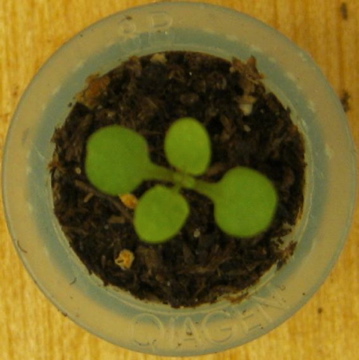

Supplement: Additional file 22 — Col-0 Top View Images for 3-D Model. Second half of images of Col-0 captured every 10 min for 5 days from the top view for the 3-D CG model. Table S2 lists the images used as key frames in the model. [file 13007_2015_75_MOESM22_ESM.zip › top_view_2/top_0363.jpg]

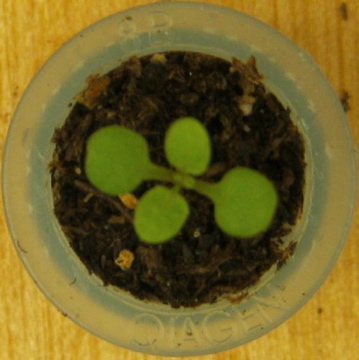

Supplement: Additional file 22 — Col-0 Top View Images for 3-D Model. Second half of images of Col-0 captured every 10 min for 5 days from the top view for the 3-D CG model. Table S2 lists the images used as key frames in the model. [file 13007_2015_75_MOESM22_ESM.zip › top_view_2/top_0364.jpg]

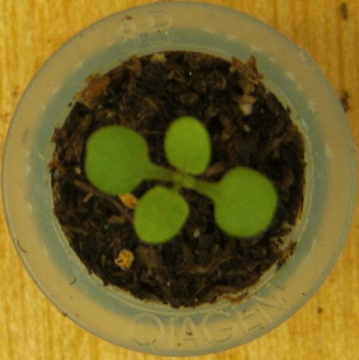

Supplement: Additional file 22 — Col-0 Top View Images for 3-D Model. Second half of images of Col-0 captured every 10 min for 5 days from the top view for the 3-D CG model. Table S2 lists the images used as key frames in the model. [file 13007_2015_75_MOESM22_ESM.zip › top_view_2/top_0365.jpg]

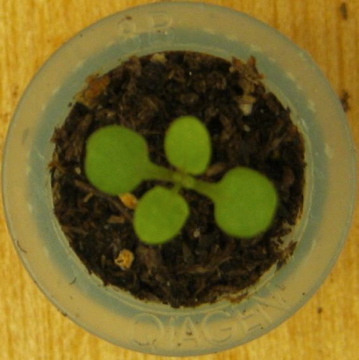

Supplement: Additional file 22 — Col-0 Top View Images for 3-D Model. Second half of images of Col-0 captured every 10 min for 5 days from the top view for the 3-D CG model. Table S2 lists the images used as key frames in the model. [file 13007_2015_75_MOESM22_ESM.zip › top_view_2/top_0366.jpg]

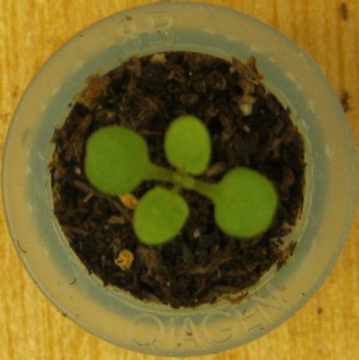

Supplement: Additional file 22 — Col-0 Top View Images for 3-D Model. Second half of images of Col-0 captured every 10 min for 5 days from the top view for the 3-D CG model. Table S2 lists the images used as key frames in the model. [file 13007_2015_75_MOESM22_ESM.zip › top_view_2/top_0367.jpg]

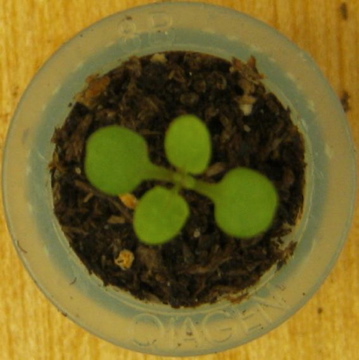

Supplement: Additional file 22 — Col-0 Top View Images for 3-D Model. Second half of images of Col-0 captured every 10 min for 5 days from the top view for the 3-D CG model. Table S2 lists the images used as key frames in the model. [file 13007_2015_75_MOESM22_ESM.zip › top_view_2/top_0368.jpg]

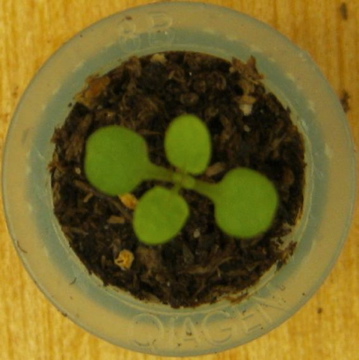

Supplement: Additional file 22 — Col-0 Top View Images for 3-D Model. Second half of images of Col-0 captured every 10 min for 5 days from the top view for the 3-D CG model. Table S2 lists the images used as key frames in the model. [file 13007_2015_75_MOESM22_ESM.zip › top_view_2/top_0369.jpg]

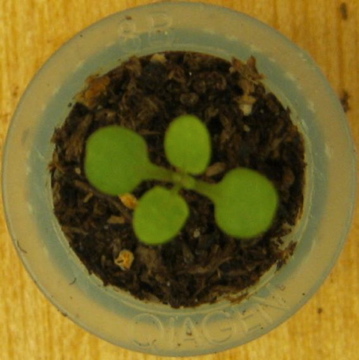

Supplement: Additional file 22 — Col-0 Top View Images for 3-D Model. Second half of images of Col-0 captured every 10 min for 5 days from the top view for the 3-D CG model. Table S2 lists the images used as key frames in the model. [file 13007_2015_75_MOESM22_ESM.zip › top_view_2/top_0370.jpg]

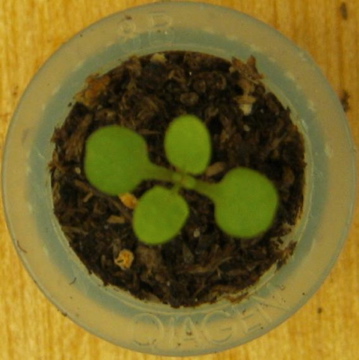

Supplement: Additional file 22 — Col-0 Top View Images for 3-D Model. Second half of images of Col-0 captured every 10 min for 5 days from the top view for the 3-D CG model. Table S2 lists the images used as key frames in the model. [file 13007_2015_75_MOESM22_ESM.zip › top_view_2/top_0371.jpg]

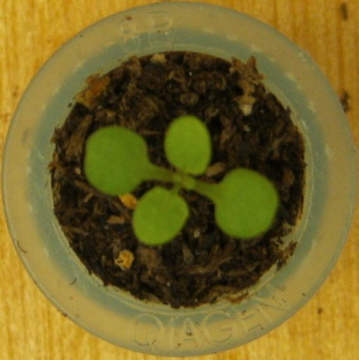

Supplement: Additional file 22 — Col-0 Top View Images for 3-D Model. Second half of images of Col-0 captured every 10 min for 5 days from the top view for the 3-D CG model. Table S2 lists the images used as key frames in the model. [file 13007_2015_75_MOESM22_ESM.zip › top_view_2/top_0372.jpg]

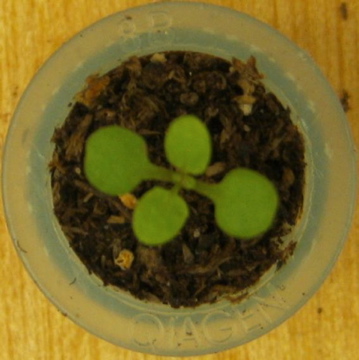

Supplement: Additional file 22 — Col-0 Top View Images for 3-D Model. Second half of images of Col-0 captured every 10 min for 5 days from the top view for the 3-D CG model. Table S2 lists the images used as key frames in the model. [file 13007_2015_75_MOESM22_ESM.zip › top_view_2/top_0373.jpg]

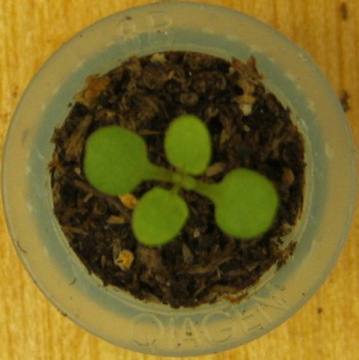

Supplement: Additional file 22 — Col-0 Top View Images for 3-D Model. Second half of images of Col-0 captured every 10 min for 5 days from the top view for the 3-D CG model. Table S2 lists the images used as key frames in the model. [file 13007_2015_75_MOESM22_ESM.zip › top_view_2/top_0374.jpg]

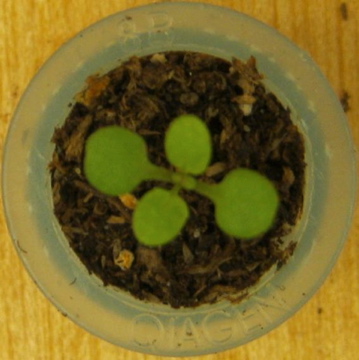

Supplement: Additional file 22 — Col-0 Top View Images for 3-D Model. Second half of images of Col-0 captured every 10 min for 5 days from the top view for the 3-D CG model. Table S2 lists the images used as key frames in the model. [file 13007_2015_75_MOESM22_ESM.zip › top_view_2/top_0375.jpg]

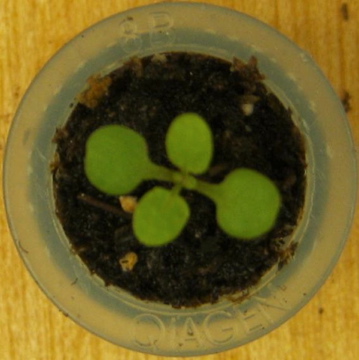

Supplement: Additional file 22 — Col-0 Top View Images for 3-D Model. Second half of images of Col-0 captured every 10 min for 5 days from the top view for the 3-D CG model. Table S2 lists the images used as key frames in the model. [file 13007_2015_75_MOESM22_ESM.zip › top_view_2/top_0376.jpg]

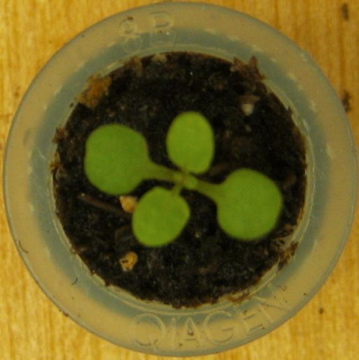

Supplement: Additional file 22 — Col-0 Top View Images for 3-D Model. Second half of images of Col-0 captured every 10 min for 5 days from the top view for the 3-D CG model. Table S2 lists the images used as key frames in the model. [file 13007_2015_75_MOESM22_ESM.zip › top_view_2/top_0377.jpg]

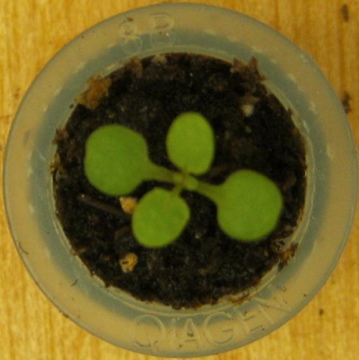

Supplement: Additional file 22 — Col-0 Top View Images for 3-D Model. Second half of images of Col-0 captured every 10 min for 5 days from the top view for the 3-D CG model. Table S2 lists the images used as key frames in the model. [file 13007_2015_75_MOESM22_ESM.zip › top_view_2/top_0378.jpg]

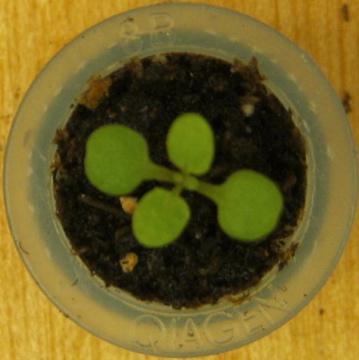

Supplement: Additional file 22 — Col-0 Top View Images for 3-D Model. Second half of images of Col-0 captured every 10 min for 5 days from the top view for the 3-D CG model. Table S2 lists the images used as key frames in the model. [file 13007_2015_75_MOESM22_ESM.zip › top_view_2/top_0379.jpg]

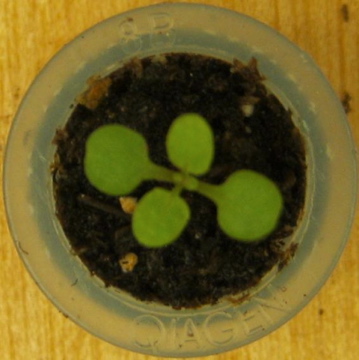

Supplement: Additional file 22 — Col-0 Top View Images for 3-D Model. Second half of images of Col-0 captured every 10 min for 5 days from the top view for the 3-D CG model. Table S2 lists the images used as key frames in the model. [file 13007_2015_75_MOESM22_ESM.zip › top_view_2/top_0380.jpg]

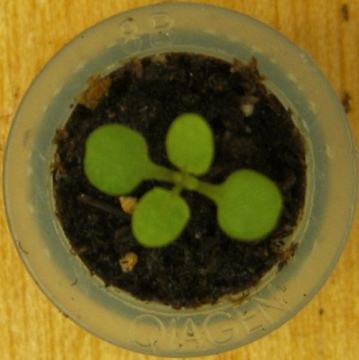

Supplement: Additional file 22 — Col-0 Top View Images for 3-D Model. Second half of images of Col-0 captured every 10 min for 5 days from the top view for the 3-D CG model. Table S2 lists the images used as key frames in the model. [file 13007_2015_75_MOESM22_ESM.zip › top_view_2/top_0381.jpg]

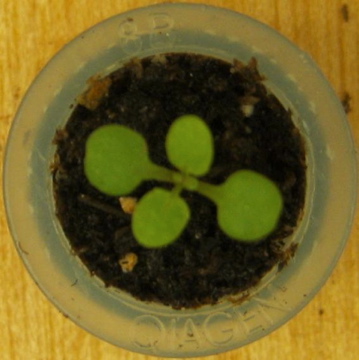

Supplement: Additional file 22 — Col-0 Top View Images for 3-D Model. Second half of images of Col-0 captured every 10 min for 5 days from the top view for the 3-D CG model. Table S2 lists the images used as key frames in the model. [file 13007_2015_75_MOESM22_ESM.zip › top_view_2/top_0382.jpg]

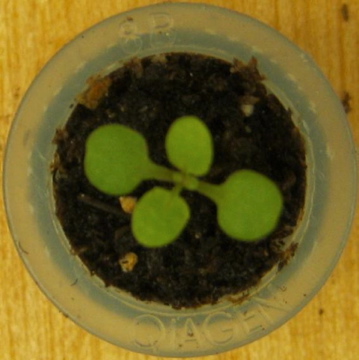

Supplement: Additional file 22 — Col-0 Top View Images for 3-D Model. Second half of images of Col-0 captured every 10 min for 5 days from the top view for the 3-D CG model. Table S2 lists the images used as key frames in the model. [file 13007_2015_75_MOESM22_ESM.zip › top_view_2/top_0383.jpg]

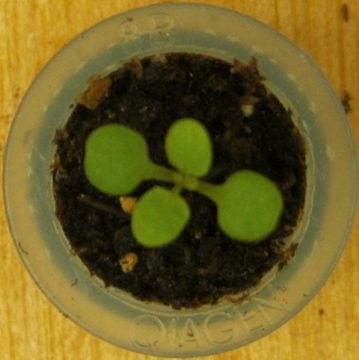

Supplement: Additional file 22 — Col-0 Top View Images for 3-D Model. Second half of images of Col-0 captured every 10 min for 5 days from the top view for the 3-D CG model. Table S2 lists the images used as key frames in the model. [file 13007_2015_75_MOESM22_ESM.zip › top_view_2/top_0384.jpg]

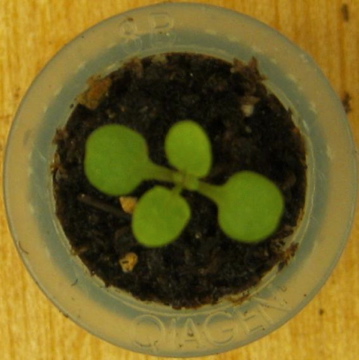

Supplement: Additional file 22 — Col-0 Top View Images for 3-D Model. Second half of images of Col-0 captured every 10 min for 5 days from the top view for the 3-D CG model. Table S2 lists the images used as key frames in the model. [file 13007_2015_75_MOESM22_ESM.zip › top_view_2/top_0385.jpg]

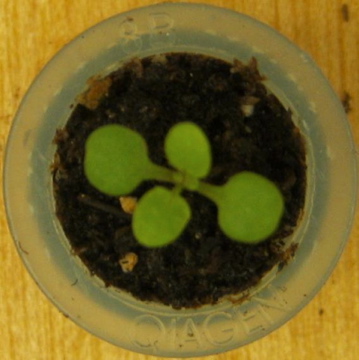

Supplement: Additional file 22 — Col-0 Top View Images for 3-D Model. Second half of images of Col-0 captured every 10 min for 5 days from the top view for the 3-D CG model. Table S2 lists the images used as key frames in the model. [file 13007_2015_75_MOESM22_ESM.zip › top_view_2/top_0386.jpg]

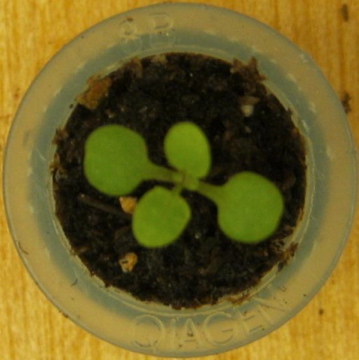

Supplement: Additional file 22 — Col-0 Top View Images for 3-D Model. Second half of images of Col-0 captured every 10 min for 5 days from the top view for the 3-D CG model. Table S2 lists the images used as key frames in the model. [file 13007_2015_75_MOESM22_ESM.zip › top_view_2/top_0387.jpg]

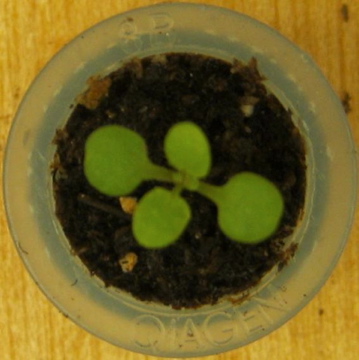

Supplement: Additional file 22 — Col-0 Top View Images for 3-D Model. Second half of images of Col-0 captured every 10 min for 5 days from the top view for the 3-D CG model. Table S2 lists the images used as key frames in the model. [file 13007_2015_75_MOESM22_ESM.zip › top_view_2/top_0388.jpg]

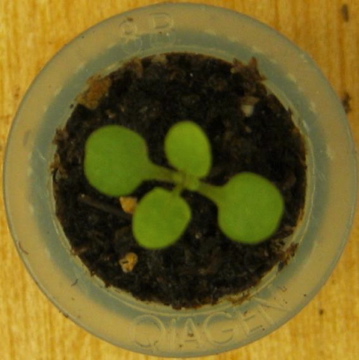

Supplement: Additional file 22 — Col-0 Top View Images for 3-D Model. Second half of images of Col-0 captured every 10 min for 5 days from the top view for the 3-D CG model. Table S2 lists the images used as key frames in the model. [file 13007_2015_75_MOESM22_ESM.zip › top_view_2/top_0389.jpg]

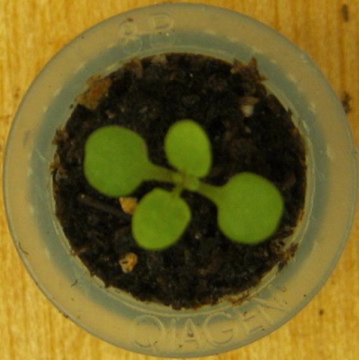

Supplement: Additional file 22 — Col-0 Top View Images for 3-D Model. Second half of images of Col-0 captured every 10 min for 5 days from the top view for the 3-D CG model. Table S2 lists the images used as key frames in the model. [file 13007_2015_75_MOESM22_ESM.zip › top_view_2/top_0390.jpg]

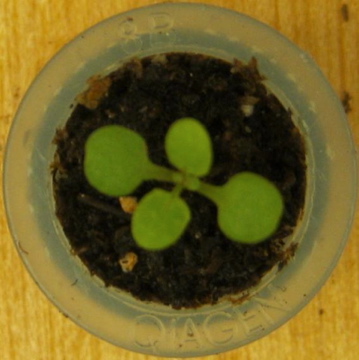

Supplement: Additional file 22 — Col-0 Top View Images for 3-D Model. Second half of images of Col-0 captured every 10 min for 5 days from the top view for the 3-D CG model. Table S2 lists the images used as key frames in the model. [file 13007_2015_75_MOESM22_ESM.zip › top_view_2/top_0391.jpg]

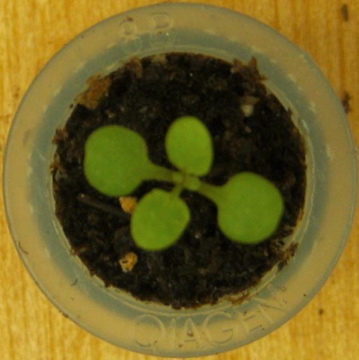

Supplement: Additional file 22 — Col-0 Top View Images for 3-D Model. Second half of images of Col-0 captured every 10 min for 5 days from the top view for the 3-D CG model. Table S2 lists the images used as key frames in the model. [file 13007_2015_75_MOESM22_ESM.zip › top_view_2/top_0392.jpg]

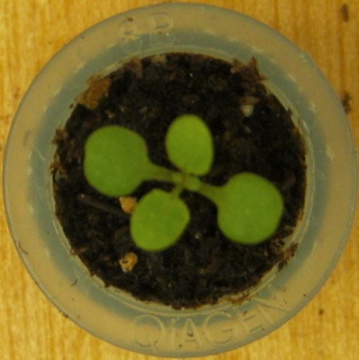

Supplement: Additional file 22 — Col-0 Top View Images for 3-D Model. Second half of images of Col-0 captured every 10 min for 5 days from the top view for the 3-D CG model. Table S2 lists the images used as key frames in the model. [file 13007_2015_75_MOESM22_ESM.zip › top_view_2/top_0393.jpg]

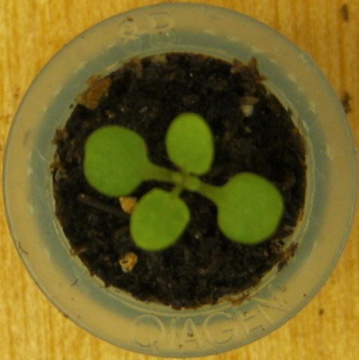

Supplement: Additional file 22 — Col-0 Top View Images for 3-D Model. Second half of images of Col-0 captured every 10 min for 5 days from the top view for the 3-D CG model. Table S2 lists the images used as key frames in the model. [file 13007_2015_75_MOESM22_ESM.zip › top_view_2/top_0394.jpg]

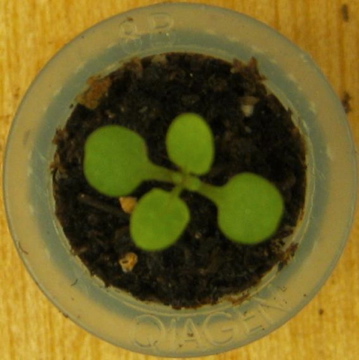

Supplement: Additional file 22 — Col-0 Top View Images for 3-D Model. Second half of images of Col-0 captured every 10 min for 5 days from the top view for the 3-D CG model. Table S2 lists the images used as key frames in the model. [file 13007_2015_75_MOESM22_ESM.zip › top_view_2/top_0395.jpg]

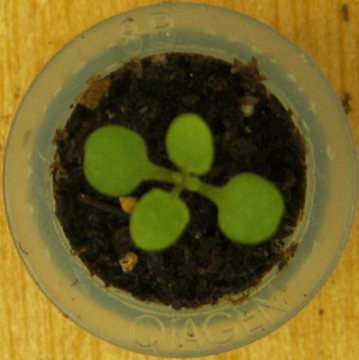

Supplement: Additional file 22 — Col-0 Top View Images for 3-D Model. Second half of images of Col-0 captured every 10 min for 5 days from the top view for the 3-D CG model. Table S2 lists the images used as key frames in the model. [file 13007_2015_75_MOESM22_ESM.zip › top_view_2/top_0396.jpg]

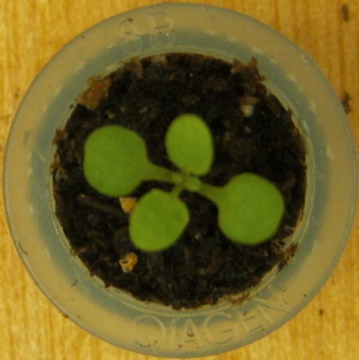

Supplement: Additional file 22 — Col-0 Top View Images for 3-D Model. Second half of images of Col-0 captured every 10 min for 5 days from the top view for the 3-D CG model. Table S2 lists the images used as key frames in the model. [file 13007_2015_75_MOESM22_ESM.zip › top_view_2/top_0397.jpg]

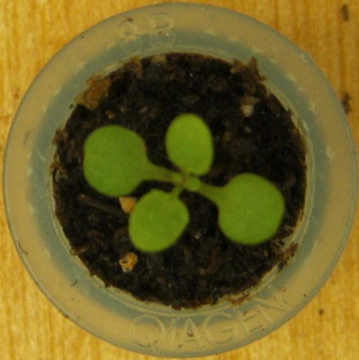

Supplement: Additional file 22 — Col-0 Top View Images for 3-D Model. Second half of images of Col-0 captured every 10 min for 5 days from the top view for the 3-D CG model. Table S2 lists the images used as key frames in the model. [file 13007_2015_75_MOESM22_ESM.zip › top_view_2/top_0398.jpg]

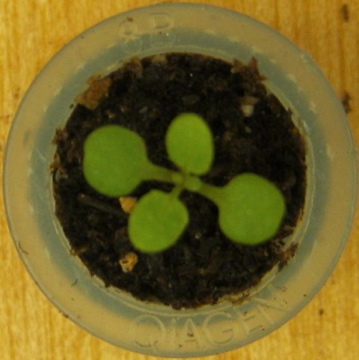

Supplement: Additional file 22 — Col-0 Top View Images for 3-D Model. Second half of images of Col-0 captured every 10 min for 5 days from the top view for the 3-D CG model. Table S2 lists the images used as key frames in the model. [file 13007_2015_75_MOESM22_ESM.zip › top_view_2/top_0399.jpg]

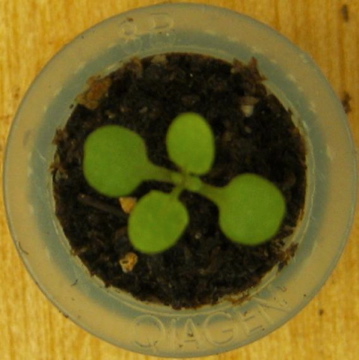

Supplement: Additional file 22 — Col-0 Top View Images for 3-D Model. Second half of images of Col-0 captured every 10 min for 5 days from the top view for the 3-D CG model. Table S2 lists the images used as key frames in the model. [file 13007_2015_75_MOESM22_ESM.zip › top_view_2/top_0400.jpg]

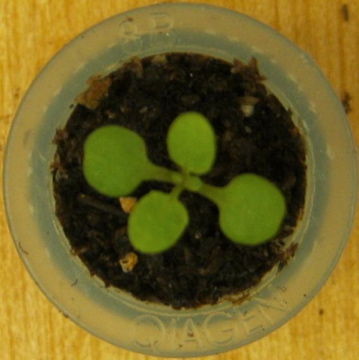

Supplement: Additional file 22 — Col-0 Top View Images for 3-D Model. Second half of images of Col-0 captured every 10 min for 5 days from the top view for the 3-D CG model. Table S2 lists the images used as key frames in the model. [file 13007_2015_75_MOESM22_ESM.zip › top_view_2/top_0401.jpg]

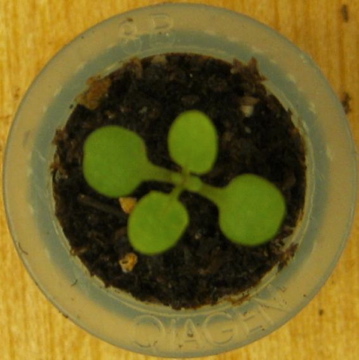

Supplement: Additional file 22 — Col-0 Top View Images for 3-D Model. Second half of images of Col-0 captured every 10 min for 5 days from the top view for the 3-D CG model. Table S2 lists the images used as key frames in the model. [file 13007_2015_75_MOESM22_ESM.zip › top_view_2/top_0402.jpg]

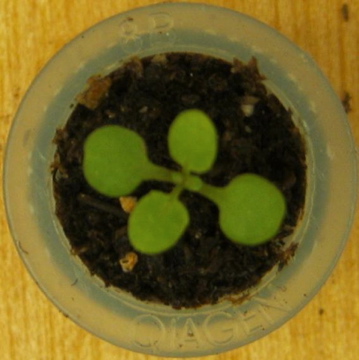

Supplement: Additional file 22 — Col-0 Top View Images for 3-D Model. Second half of images of Col-0 captured every 10 min for 5 days from the top view for the 3-D CG model. Table S2 lists the images used as key frames in the model. [file 13007_2015_75_MOESM22_ESM.zip › top_view_2/top_0403.jpg]

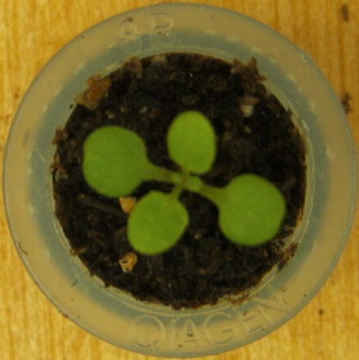

Supplement: Additional file 22 — Col-0 Top View Images for 3-D Model. Second half of images of Col-0 captured every 10 min for 5 days from the top view for the 3-D CG model. Table S2 lists the images used as key frames in the model. [file 13007_2015_75_MOESM22_ESM.zip › top_view_2/top_0404.jpg]

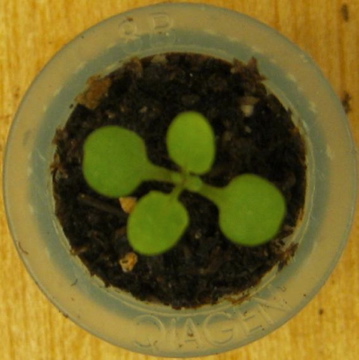

Supplement: Additional file 22 — Col-0 Top View Images for 3-D Model. Second half of images of Col-0 captured every 10 min for 5 days from the top view for the 3-D CG model. Table S2 lists the images used as key frames in the model. [file 13007_2015_75_MOESM22_ESM.zip › top_view_2/top_0405.jpg]

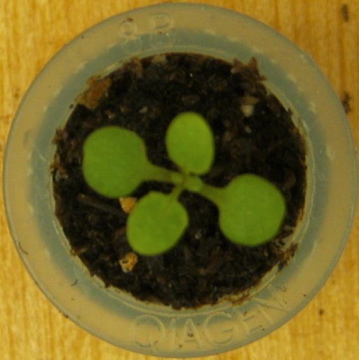

Supplement: Additional file 22 — Col-0 Top View Images for 3-D Model. Second half of images of Col-0 captured every 10 min for 5 days from the top view for the 3-D CG model. Table S2 lists the images used as key frames in the model. [file 13007_2015_75_MOESM22_ESM.zip › top_view_2/top_0406.jpg]

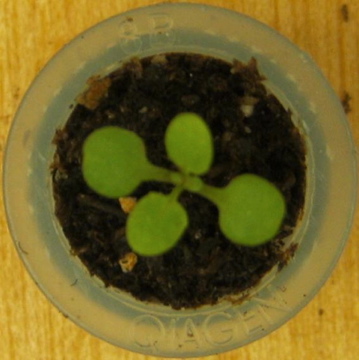

Supplement: Additional file 22 — Col-0 Top View Images for 3-D Model. Second half of images of Col-0 captured every 10 min for 5 days from the top view for the 3-D CG model. Table S2 lists the images used as key frames in the model. [file 13007_2015_75_MOESM22_ESM.zip › top_view_2/top_0407.jpg]

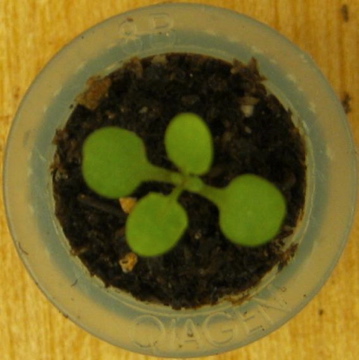

Supplement: Additional file 22 — Col-0 Top View Images for 3-D Model. Second half of images of Col-0 captured every 10 min for 5 days from the top view for the 3-D CG model. Table S2 lists the images used as key frames in the model. [file 13007_2015_75_MOESM22_ESM.zip › top_view_2/top_0408.jpg]

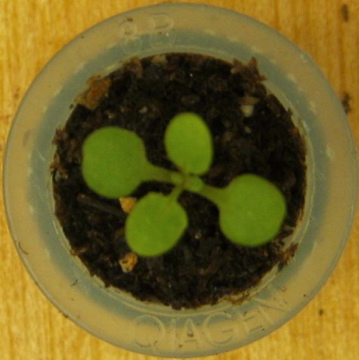

Supplement: Additional file 22 — Col-0 Top View Images for 3-D Model. Second half of images of Col-0 captured every 10 min for 5 days from the top view for the 3-D CG model. Table S2 lists the images used as key frames in the model. [file 13007_2015_75_MOESM22_ESM.zip › top_view_2/top_0409.jpg]

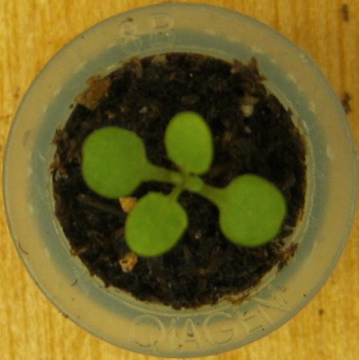

Supplement: Additional file 22 — Col-0 Top View Images for 3-D Model. Second half of images of Col-0 captured every 10 min for 5 days from the top view for the 3-D CG model. Table S2 lists the images used as key frames in the model. [file 13007_2015_75_MOESM22_ESM.zip › top_view_2/top_0410.jpg]

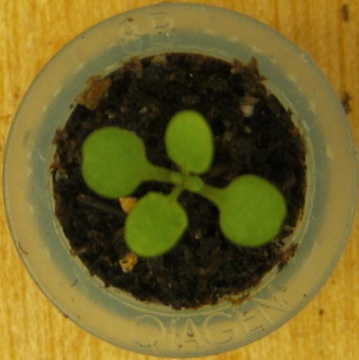

Supplement: Additional file 22 — Col-0 Top View Images for 3-D Model. Second half of images of Col-0 captured every 10 min for 5 days from the top view for the 3-D CG model. Table S2 lists the images used as key frames in the model. [file 13007_2015_75_MOESM22_ESM.zip › top_view_2/top_0411.jpg]

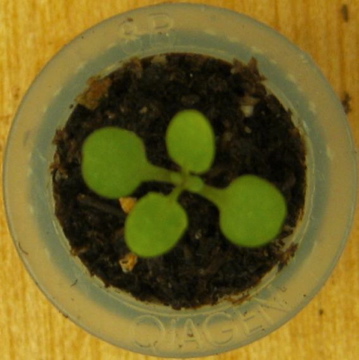

Supplement: Additional file 22 — Col-0 Top View Images for 3-D Model. Second half of images of Col-0 captured every 10 min for 5 days from the top view for the 3-D CG model. Table S2 lists the images used as key frames in the model. [file 13007_2015_75_MOESM22_ESM.zip › top_view_2/top_0412.jpg]

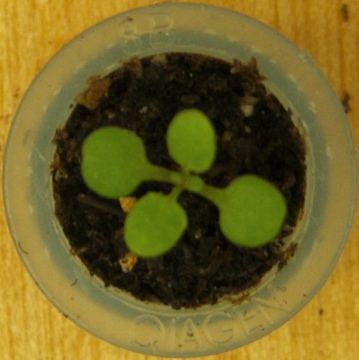

Supplement: Additional file 22 — Col-0 Top View Images for 3-D Model. Second half of images of Col-0 captured every 10 min for 5 days from the top view for the 3-D CG model. Table S2 lists the images used as key frames in the model. [file 13007_2015_75_MOESM22_ESM.zip › top_view_2/top_0413.jpg]

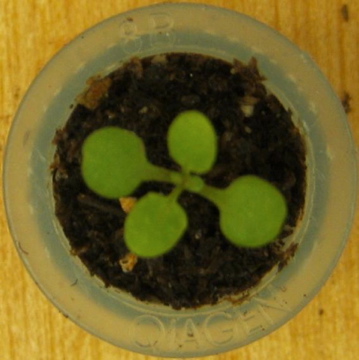

Supplement: Additional file 22 — Col-0 Top View Images for 3-D Model. Second half of images of Col-0 captured every 10 min for 5 days from the top view for the 3-D CG model. Table S2 lists the images used as key frames in the model. [file 13007_2015_75_MOESM22_ESM.zip › top_view_2/top_0414.jpg]

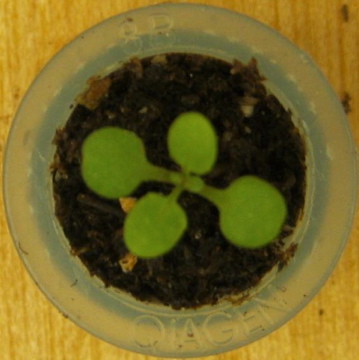

Supplement: Additional file 22 — Col-0 Top View Images for 3-D Model. Second half of images of Col-0 captured every 10 min for 5 days from the top view for the 3-D CG model. Table S2 lists the images used as key frames in the model. [file 13007_2015_75_MOESM22_ESM.zip › top_view_2/top_0415.jpg]

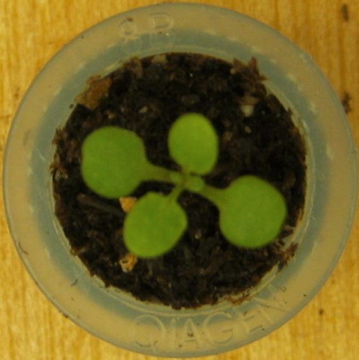

Supplement: Additional file 22 — Col-0 Top View Images for 3-D Model. Second half of images of Col-0 captured every 10 min for 5 days from the top view for the 3-D CG model. Table S2 lists the images used as key frames in the model. [file 13007_2015_75_MOESM22_ESM.zip › top_view_2/top_0416.jpg]

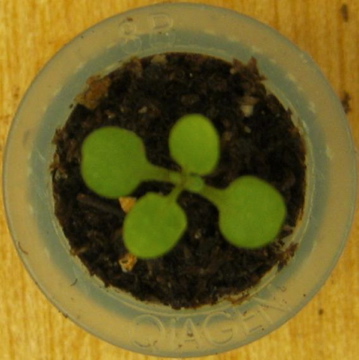

Supplement: Additional file 22 — Col-0 Top View Images for 3-D Model. Second half of images of Col-0 captured every 10 min for 5 days from the top view for the 3-D CG model. Table S2 lists the images used as key frames in the model. [file 13007_2015_75_MOESM22_ESM.zip › top_view_2/top_0417.jpg]

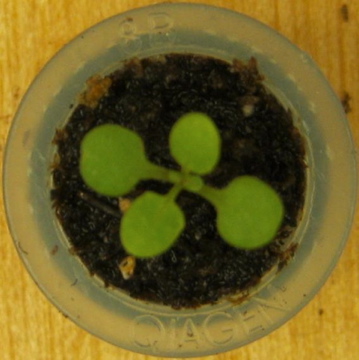

Supplement: Additional file 22 — Col-0 Top View Images for 3-D Model. Second half of images of Col-0 captured every 10 min for 5 days from the top view for the 3-D CG model. Table S2 lists the images used as key frames in the model. [file 13007_2015_75_MOESM22_ESM.zip › top_view_2/top_0418.jpg]

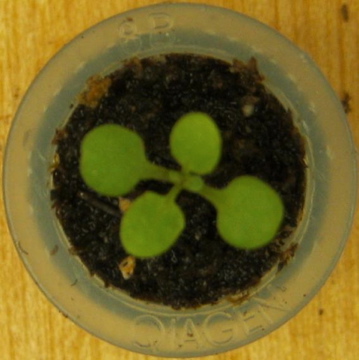

Supplement: Additional file 22 — Col-0 Top View Images for 3-D Model. Second half of images of Col-0 captured every 10 min for 5 days from the top view for the 3-D CG model. Table S2 lists the images used as key frames in the model. [file 13007_2015_75_MOESM22_ESM.zip › top_view_2/top_0419.jpg]

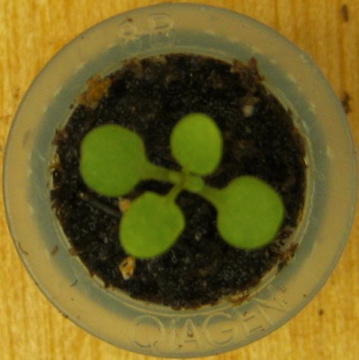

Supplement: Additional file 22 — Col-0 Top View Images for 3-D Model. Second half of images of Col-0 captured every 10 min for 5 days from the top view for the 3-D CG model. Table S2 lists the images used as key frames in the model. [file 13007_2015_75_MOESM22_ESM.zip › top_view_2/top_0420.jpg]

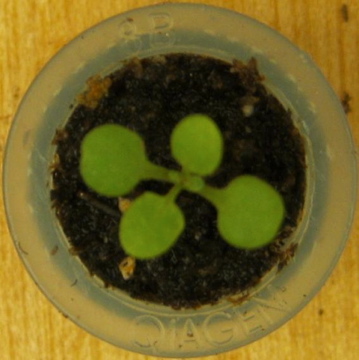

Supplement: Additional file 22 — Col-0 Top View Images for 3-D Model. Second half of images of Col-0 captured every 10 min for 5 days from the top view for the 3-D CG model. Table S2 lists the images used as key frames in the model. [file 13007_2015_75_MOESM22_ESM.zip › top_view_2/top_0421.jpg]

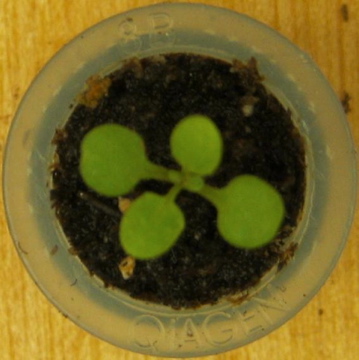

Supplement: Additional file 22 — Col-0 Top View Images for 3-D Model. Second half of images of Col-0 captured every 10 min for 5 days from the top view for the 3-D CG model. Table S2 lists the images used as key frames in the model. [file 13007_2015_75_MOESM22_ESM.zip › top_view_2/top_0422.jpg]

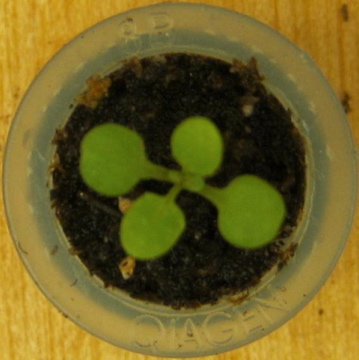

Supplement: Additional file 22 — Col-0 Top View Images for 3-D Model. Second half of images of Col-0 captured every 10 min for 5 days from the top view for the 3-D CG model. Table S2 lists the images used as key frames in the model. [file 13007_2015_75_MOESM22_ESM.zip › top_view_2/top_0423.jpg]

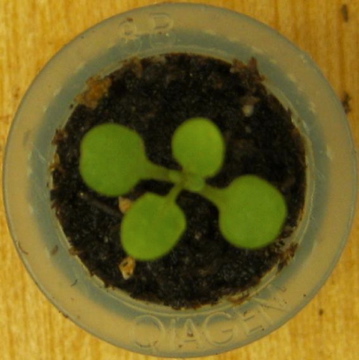

Supplement: Additional file 22 — Col-0 Top View Images for 3-D Model. Second half of images of Col-0 captured every 10 min for 5 days from the top view for the 3-D CG model. Table S2 lists the images used as key frames in the model. [file 13007_2015_75_MOESM22_ESM.zip › top_view_2/top_0424.jpg]

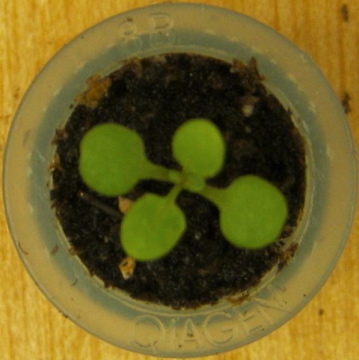

Supplement: Additional file 22 — Col-0 Top View Images for 3-D Model. Second half of images of Col-0 captured every 10 min for 5 days from the top view for the 3-D CG model. Table S2 lists the images used as key frames in the model. [file 13007_2015_75_MOESM22_ESM.zip › top_view_2/top_0425.jpg]

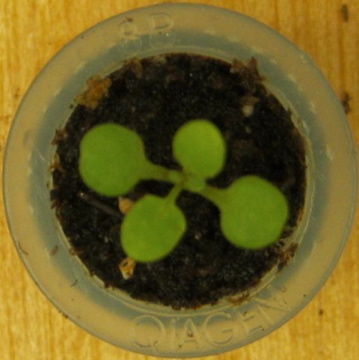

Supplement: Additional file 22 — Col-0 Top View Images for 3-D Model. Second half of images of Col-0 captured every 10 min for 5 days from the top view for the 3-D CG model. Table S2 lists the images used as key frames in the model. [file 13007_2015_75_MOESM22_ESM.zip › top_view_2/top_0426.jpg]

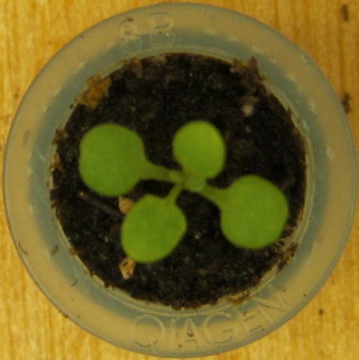

Supplement: Additional file 22 — Col-0 Top View Images for 3-D Model. Second half of images of Col-0 captured every 10 min for 5 days from the top view for the 3-D CG model. Table S2 lists the images used as key frames in the model. [file 13007_2015_75_MOESM22_ESM.zip › top_view_2/top_0427.jpg]

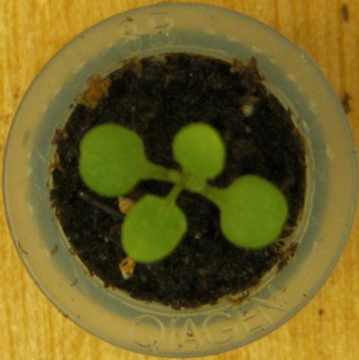

Supplement: Additional file 22 — Col-0 Top View Images for 3-D Model. Second half of images of Col-0 captured every 10 min for 5 days from the top view for the 3-D CG model. Table S2 lists the images used as key frames in the model. [file 13007_2015_75_MOESM22_ESM.zip › top_view_2/top_0428.jpg]

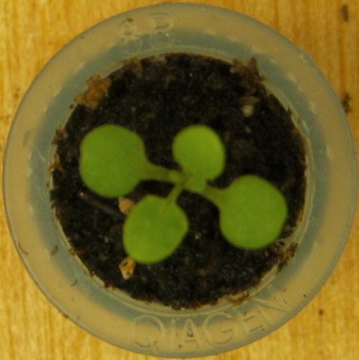

Supplement: Additional file 22 — Col-0 Top View Images for 3-D Model. Second half of images of Col-0 captured every 10 min for 5 days from the top view for the 3-D CG model. Table S2 lists the images used as key frames in the model. [file 13007_2015_75_MOESM22_ESM.zip › top_view_2/top_0429.jpg]

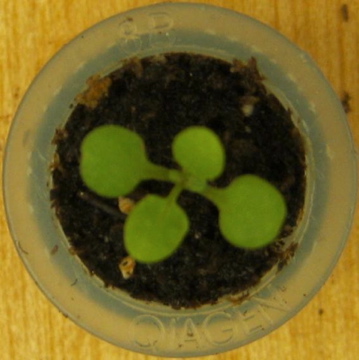

Supplement: Additional file 22 — Col-0 Top View Images for 3-D Model. Second half of images of Col-0 captured every 10 min for 5 days from the top view for the 3-D CG model. Table S2 lists the images used as key frames in the model. [file 13007_2015_75_MOESM22_ESM.zip › top_view_2/top_0430.jpg]

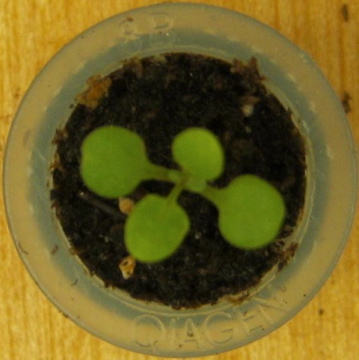

Supplement: Additional file 22 — Col-0 Top View Images for 3-D Model. Second half of images of Col-0 captured every 10 min for 5 days from the top view for the 3-D CG model. Table S2 lists the images used as key frames in the model. [file 13007_2015_75_MOESM22_ESM.zip › top_view_2/top_0431.jpg]

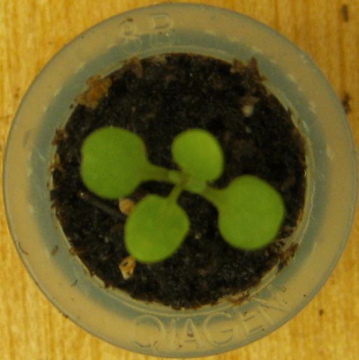

Supplement: Additional file 22 — Col-0 Top View Images for 3-D Model. Second half of images of Col-0 captured every 10 min for 5 days from the top view for the 3-D CG model. Table S2 lists the images used as key frames in the model. [file 13007_2015_75_MOESM22_ESM.zip › top_view_2/top_0432.jpg]

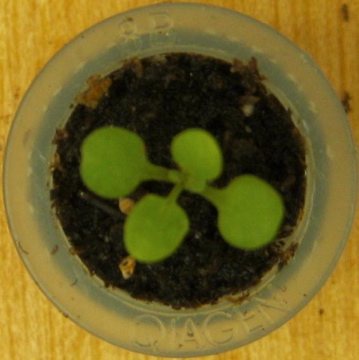

Supplement: Additional file 22 — Col-0 Top View Images for 3-D Model. Second half of images of Col-0 captured every 10 min for 5 days from the top view for the 3-D CG model. Table S2 lists the images used as key frames in the model. [file 13007_2015_75_MOESM22_ESM.zip › top_view_2/top_0433.jpg]

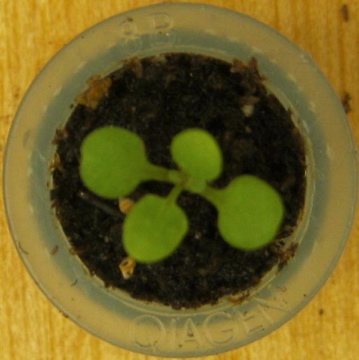

Supplement: Additional file 22 — Col-0 Top View Images for 3-D Model. Second half of images of Col-0 captured every 10 min for 5 days from the top view for the 3-D CG model. Table S2 lists the images used as key frames in the model. [file 13007_2015_75_MOESM22_ESM.zip › top_view_2/top_0434.jpg]

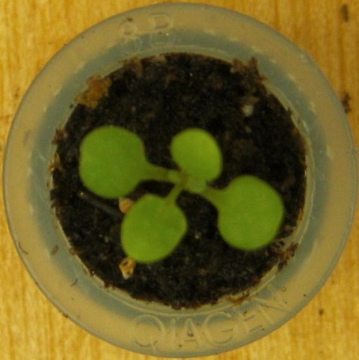

Supplement: Additional file 22 — Col-0 Top View Images for 3-D Model. Second half of images of Col-0 captured every 10 min for 5 days from the top view for the 3-D CG model. Table S2 lists the images used as key frames in the model. [file 13007_2015_75_MOESM22_ESM.zip › top_view_2/top_0435.jpg]

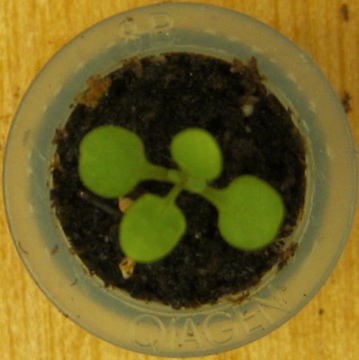

Supplement: Additional file 22 — Col-0 Top View Images for 3-D Model. Second half of images of Col-0 captured every 10 min for 5 days from the top view for the 3-D CG model. Table S2 lists the images used as key frames in the model. [file 13007_2015_75_MOESM22_ESM.zip › top_view_2/top_0436.jpg]

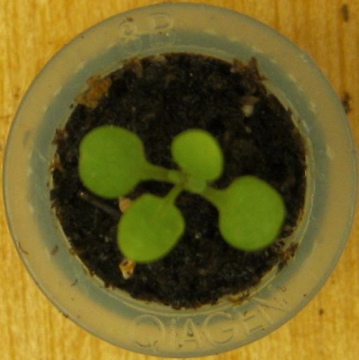

Supplement: Additional file 22 — Col-0 Top View Images for 3-D Model. Second half of images of Col-0 captured every 10 min for 5 days from the top view for the 3-D CG model. Table S2 lists the images used as key frames in the model. [file 13007_2015_75_MOESM22_ESM.zip › top_view_2/top_0437.jpg]

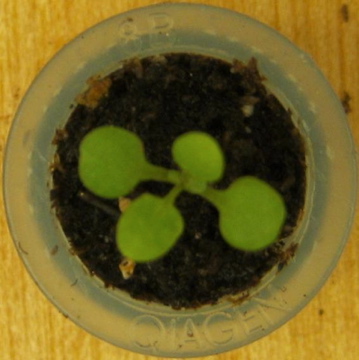

Supplement: Additional file 22 — Col-0 Top View Images for 3-D Model. Second half of images of Col-0 captured every 10 min for 5 days from the top view for the 3-D CG model. Table S2 lists the images used as key frames in the model. [file 13007_2015_75_MOESM22_ESM.zip › top_view_2/top_0438.jpg]

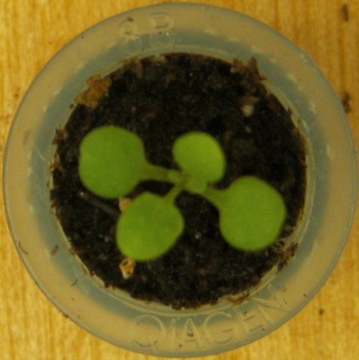

Supplement: Additional file 22 — Col-0 Top View Images for 3-D Model. Second half of images of Col-0 captured every 10 min for 5 days from the top view for the 3-D CG model. Table S2 lists the images used as key frames in the model. [file 13007_2015_75_MOESM22_ESM.zip › top_view_2/top_0439.jpg]

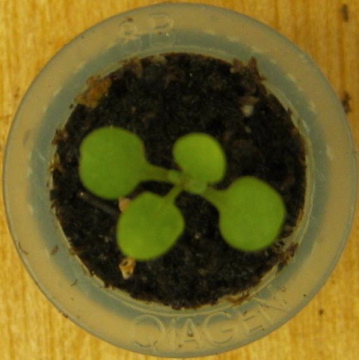

Supplement: Additional file 22 — Col-0 Top View Images for 3-D Model. Second half of images of Col-0 captured every 10 min for 5 days from the top view for the 3-D CG model. Table S2 lists the images used as key frames in the model. [file 13007_2015_75_MOESM22_ESM.zip › top_view_2/top_0440.jpg]

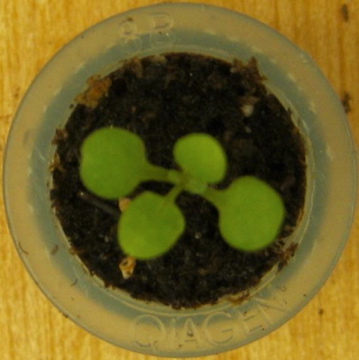

Supplement: Additional file 22 — Col-0 Top View Images for 3-D Model. Second half of images of Col-0 captured every 10 min for 5 days from the top view for the 3-D CG model. Table S2 lists the images used as key frames in the model. [file 13007_2015_75_MOESM22_ESM.zip › top_view_2/top_0441.jpg]

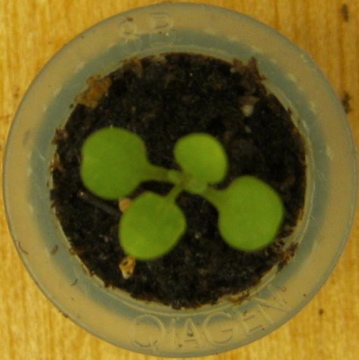

Supplement: Additional file 22 — Col-0 Top View Images for 3-D Model. Second half of images of Col-0 captured every 10 min for 5 days from the top view for the 3-D CG model. Table S2 lists the images used as key frames in the model. [file 13007_2015_75_MOESM22_ESM.zip › top_view_2/top_0442.jpg]

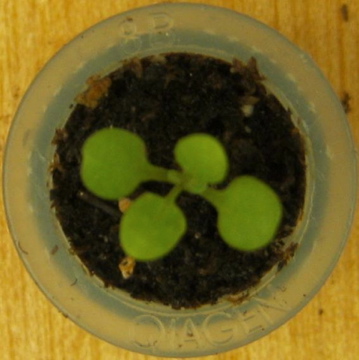

Supplement: Additional file 22 — Col-0 Top View Images for 3-D Model. Second half of images of Col-0 captured every 10 min for 5 days from the top view for the 3-D CG model. Table S2 lists the images used as key frames in the model. [file 13007_2015_75_MOESM22_ESM.zip › top_view_2/top_0443.jpg]

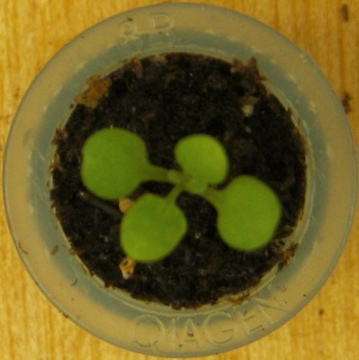

Supplement: Additional file 22 — Col-0 Top View Images for 3-D Model. Second half of images of Col-0 captured every 10 min for 5 days from the top view for the 3-D CG model. Table S2 lists the images used as key frames in the model. [file 13007_2015_75_MOESM22_ESM.zip › top_view_2/top_0444.jpg]

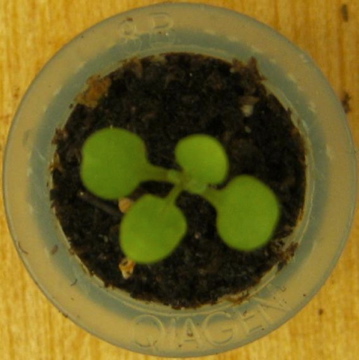

Supplement: Additional file 22 — Col-0 Top View Images for 3-D Model. Second half of images of Col-0 captured every 10 min for 5 days from the top view for the 3-D CG model. Table S2 lists the images used as key frames in the model. [file 13007_2015_75_MOESM22_ESM.zip › top_view_2/top_0445.jpg]

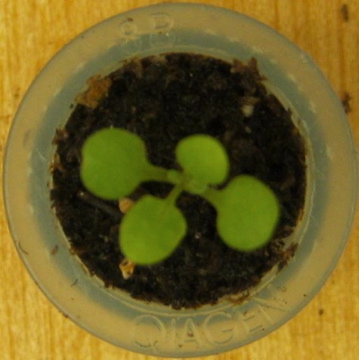

Supplement: Additional file 22 — Col-0 Top View Images for 3-D Model. Second half of images of Col-0 captured every 10 min for 5 days from the top view for the 3-D CG model. Table S2 lists the images used as key frames in the model. [file 13007_2015_75_MOESM22_ESM.zip › top_view_2/top_0446.jpg]

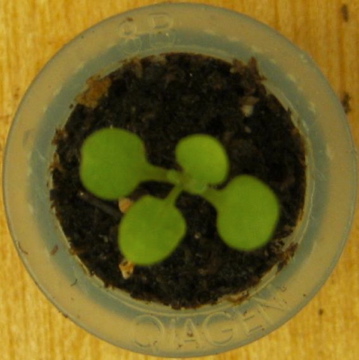

Supplement: Additional file 22 — Col-0 Top View Images for 3-D Model. Second half of images of Col-0 captured every 10 min for 5 days from the top view for the 3-D CG model. Table S2 lists the images used as key frames in the model. [file 13007_2015_75_MOESM22_ESM.zip › top_view_2/top_0447.jpg]

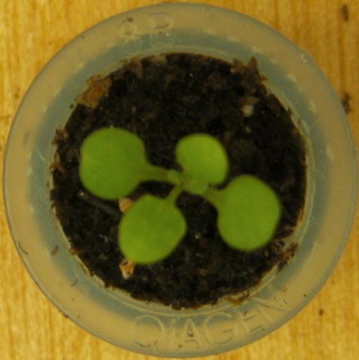

Supplement: Additional file 22 — Col-0 Top View Images for 3-D Model. Second half of images of Col-0 captured every 10 min for 5 days from the top view for the 3-D CG model. Table S2 lists the images used as key frames in the model. [file 13007_2015_75_MOESM22_ESM.zip › top_view_2/top_0448.jpg]

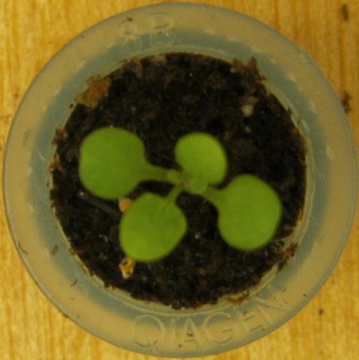

Supplement: Additional file 22 — Col-0 Top View Images for 3-D Model. Second half of images of Col-0 captured every 10 min for 5 days from the top view for the 3-D CG model. Table S2 lists the images used as key frames in the model. [file 13007_2015_75_MOESM22_ESM.zip › top_view_2/top_0449.jpg]

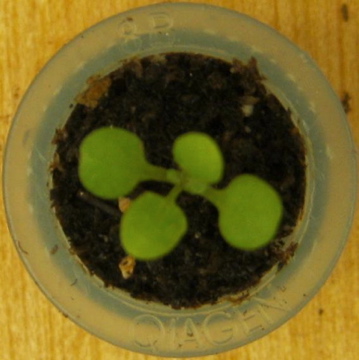

Supplement: Additional file 22 — Col-0 Top View Images for 3-D Model. Second half of images of Col-0 captured every 10 min for 5 days from the top view for the 3-D CG model. Table S2 lists the images used as key frames in the model. [file 13007_2015_75_MOESM22_ESM.zip › top_view_2/top_0450.jpg]

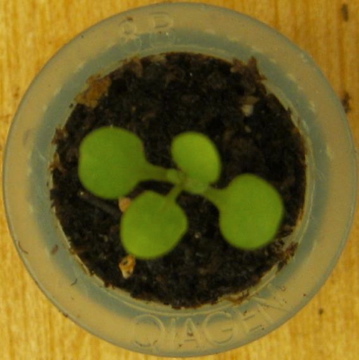

Supplement: Additional file 22 — Col-0 Top View Images for 3-D Model. Second half of images of Col-0 captured every 10 min for 5 days from the top view for the 3-D CG model. Table S2 lists the images used as key frames in the model. [file 13007_2015_75_MOESM22_ESM.zip › top_view_2/top_0451.jpg]

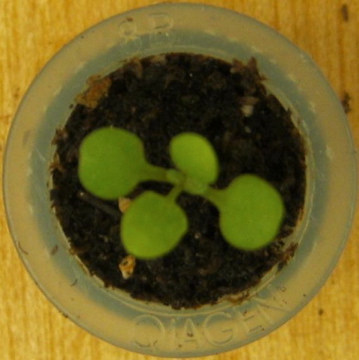

Supplement: Additional file 22 — Col-0 Top View Images for 3-D Model. Second half of images of Col-0 captured every 10 min for 5 days from the top view for the 3-D CG model. Table S2 lists the images used as key frames in the model. [file 13007_2015_75_MOESM22_ESM.zip › top_view_2/top_0452.jpg]

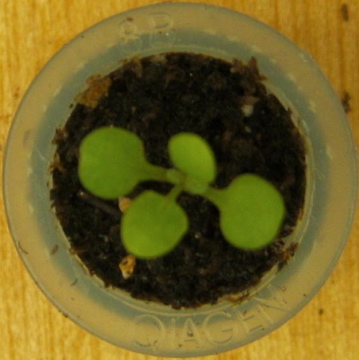

Supplement: Additional file 22 — Col-0 Top View Images for 3-D Model. Second half of images of Col-0 captured every 10 min for 5 days from the top view for the 3-D CG model. Table S2 lists the images used as key frames in the model. [file 13007_2015_75_MOESM22_ESM.zip › top_view_2/top_0453.jpg]
